# Data platforms for open life sciences–A systematic analysis of management instruments

**DOI:** 10.1371/journal.pone.0276204

**Published:** 2022-10-25

**Authors:** Daniel Laufs, Mareike Peters, Carsten Schultz

**Affiliations:** Technology Management Research Group, Faculty of Business, Economics and Social Sciences, Kiel University, Kiel, SH, Germany; Nazarbayev University, KAZAKHSTAN

## Abstract

Open data platforms are interfaces between data demand of and supply from their users. Yet, data platform providers frequently struggle to aggregate data to suit their users’ needs and to establish a high intensity of data exchange in a collaborative environment. Here, using open life science data platforms as an example for a diverse data structure, we systematically categorize these platforms based on their technology intermediation and the range of domains they cover to derive general and specific success factors for their management instruments. Our qualitative content analysis is based on 39 in-depth interviews with experts employed by data platforms and external stakeholders. We thus complement peer initiatives which focus solely on data quality, by additionally highlighting the data platforms’ role to enable data utilization for innovative output. Based on our analysis, we propose a clearly structured and detailed guideline for seven management instruments. This guideline helps to establish and operationalize data platforms and to best exploit the data provided. Our findings support further exploitation of the open innovation potential in the life sciences and beyond.

## Introduction

Open Science approaches in life sciences facilitate the recombination of knowledge through interdisciplinary collaboration and support the exchange of ideas, experiences, and resources [1]. Data platforms (DPs) like *Metabolights*, *UniProt and figshare* support the shared use of data. However, their users frequently struggle utilizing their data stocks for large-scale analyses [2]. Our exploratory study aims to provide a framework of relevant DP’s management instruments in Open Life Science that may help to overcome the identified challenges.

Open Science comprises transparent and accessible data and knowledge shared and developed through collaborative networks [1]. DPs are organized collections of data stored and accessed electronically. In contrast to private DPs storing data from organizations’ internal research results or hospitals’ patient registries, this study focuses on open DPs. These open DPs facilitate data transfers taking the central role of intermediaries between external users [3–6], functioning as interfaces between data supply and demand. Regarding the DP users’ role as data supplier, their motivation to share data on a specific DP is critical for its success. Regarding the DP users’ role as data consumers, the data’s accessibility, characteristics, and structure on the DP are critical. In heterogeneous scientific domains, DPs link data and users beyond distinct application fields. Especially in the life sciences, researchers struggle to make their contributions reproducible and sharable, due to increasing volumes of high-throughput multimodal data and challenges in generating and utilizing robust and scalable data to enable large-scale analyses [2]. Hence, open DPs frequently experience difficulties to access the relevant volume of comprised data, to establish a high intensity of data exchange, and to create a collaborative environment for their users. Building on prior research and an extensive qualitative study, our study reveals management structures and activities that determine efficient data exchange on DPs within the life sciences to resolve these difficulties. It offers a guideline to successfully establish and operate DPs to increase the diffusion of the DP in the scientific community and to exploit the data provided to the best extent.

The increasing data demands from life sciences DPs and the challenges inherent in their successful operation, call for a systematic analysis of the required management instruments. We focus on DPs’ management instruments that are central to the implementation of data-related services driving open life sciences. Based on classification of existing DPs and 39 in-depth expert interviews, we describe relevant management instruments and activities which comprise formal requirements, rules, and guidelines, and ensure effective and efficient data exchange [7]. We structure this manuscript as follows: after introducing the current state of the art of DP management and existing guidelines in the life sciences, we distinguish DPs from other platform types, and we analyze management instruments for three data platform types based on a qualitative content analysis of the interview data. We discuss categorical differences between seven management instruments, and highlight practical implications for DP governance and management fostering data exchange in Open Science.

## Managing open data platforms in the life sciences

In this chapter, we introduce open DPs in the life sciences and highlight data management challenges of DP providers. We summarize DP roles and present approaches to managing life sciences data platforms including additional DP services and tools. We then turn to general data management guidelines.

### Data platforms in the life sciences

DPs in the life sciences have a long history of an increasing range of applications, expanded functions and improved management structures. DPs evolved from printed data collections like the *Atlas of Protein Sequence and Structure*, 1965 [8, 9]. Also in the 1960s, early examples for computer-assisted digitalized data collections were established and maintained, like the *Cambridge Structural Database* by *Cambridge Crystallographic Data Centre* (*CCDC*) which contains all published organic and metal-organic small-molecule crystal structures [10]. In 1971, the *Protein Data Bank* was introduced in *Nature New Biology* as repository system for protein crystallographic data, “making machine readable data available on magnetic tape free of charge” [11]. This joint venture by the UK *CCDC* and the US *Brookhaven National Laboratory* maintained identical files on both continents. In 1984, the US *National Biomedical Research Foundation* introduced the *Protein Information Resource* (*PIR*) as an integrated public bioinformatics resource [12]. Accessed by telephone line, it was the first online database system available for interrogation by remote computers [13]. Brazma et al. pioneered minimum information standards for recording and reporting microarray-based gene expression data, that have later been transferred to other research fields [14]. The *European Bioinformatic Institute’s “Ensembl Genome Database Project”* from 1999 is an early example of the co-development of a centralized, comprehensive genome informatics resource, integrating experimental and reference data from multiple providers for use by researchers like geneticists and molecular biologists [15]. Recent enhancement of the *Ensembl* annotation and processing methods resulted in the *Rapid Release Platform* and accelerated the pace of genome annotation. Consolidations increase the strength of individual platforms, for example *UniProt* created by combining the *Swiss-Prot*, *TrEMBL* and *PIR-PSD* databases as universal central protein data resource [16]. Bioinformatics workflow management systems, like the *Galaxy Platform*, offer complementary tools providing open-source workflows within the life sciences for scientists who do not possess sufficient programming or systems administration experience [17].

Today, a large variety of life science DPs exist within and across all sub-disciplines [18] categorizing data like biodiversity patterns (e.g., *Global Biodiversity Information Facility’s infrastructure*), protein sequences (e.g., *UniProt*), enzymes (e.g., *BRENDA*), nucleic acids (e.g., *GenBank*), and molecular structures (e.g., *Cambridge Structural Database*). The data structure differs according to the data type, for example, distinguishing between sequences (like DNA, RNA, and amino acid sequences), graphs (like metabolic pathways, gene regulatory networks, and taxonomies), 3D structures (like molecule structures), temporal data (like cellular response to external changes or evolutionary biology), and mathematic models (like parameter estimation and testing of statistical models of biological systems and datasets) [19]. To uncover scientific matters, like DNA genomes and molecular pathways, and to translate them into new concrete application fields, researchers and industrial actors from various disciplines require heterogeneous original data. Their efficient exploitation is one of the major obstacles in big data analytics [20]. In addition, life science DPs are critical for ensuring the reproducibility and integrity of the entire life sciences domain [21] but many DPs are supported by short-term grants, and there is little coordination of funding across these resources [22]. To harmonize multimodal data for heterogenous users, professionalization of management instruments by life sciences platform providers is needed [2].

Users from various disciplines have different demands, and sufficient data exploitation requires individualized solutions and complementary services, challenging the existing platform providers’ structures [23, 24]. Logistical and technical challenges to discover, query, and integrate heterogenous syntaxes, structures, formats, and biomedical entity notations cause implementation challenges, and result in stand-alone data sources that are not interlinked with other resources, that use unpublished schemas with minimal reuse or mappings, and that may have elements not useful for data integration from a biomedical perspective [25]. The existence of several isolated, heterogeneous data sources causes high variance in formats, syntaxes and schemas [26], limits data sharing, and creates uncertainty among potential users. These challenges escalate, since data volume increases rapidly and, therefore, the solutions to store, analyze and publish standardized data increase in complexity [27, 28]. Intelligent DP structures need to be developed to foster vigorous data exchange which goes beyond single disciplines, enabling interdisciplinary collaboration.

### Existing approaches to managing life sciences data platforms

Platforms are keystones in the process of data identification, access, management, analysis and use. Some platforms play the role as data repositories, a data storage entity, where data associated with a previously conducted research study or a publication is placed for analytical or reporting purposes. Many journals require authors to share data of a study in an appropriate repository for publication. Most often, users must meet these repositories’ data deposition criteria, like minimum information check-lists, standard ontologies or vocabularies, to enable researchers to replicate the analysis or to reuse the data in new investigations. *UniProt*, for example, additionally encourages authors to provide a long textual description on the data submitted [29]. Some data repositories offer services like a limited data access for external users. *Zenodo*, as an example for a generic platform, allows the deposition of restricted files with the ability to share access with other users if certain requirements are met [30]. Compared to these repositories, platforms can go beyond data storage and have additional functionalities, such as collaborative spaces or analytics tools to promote data sharing, use and recombination. Open DPs are multi-sided and most sensitive to management instruments because of their intense user interactions [31]. Sharing data from their contributors and linking external resources, such as data from repositories, open DPs must implement new functionalities, like control mechanisms for automatized data upload, to prevent platform abuse at the cost of increasing data management complexity. Some databases build on decentralized entries from one or several data repositories, and they feature highly processed and curated data, summarize complex or unstructured information, and update and change their structure over time [32–34]. As such, platforms are of particular importance in Open Science.

Research infrastructures can go beyond data platform management. The pan-European research infrastructure *ELIXIR*, for example, states to unite Europe’s leading life science organizations in managing and safeguarding the increasing data volume being generated by publicly funded research [35]. Among other services, *ELIXIR* includes an entire platform environment that provides robust, long-term data resources within a coordinated, scalable and connected data ecosystem [35, 36]. Embedded within the *ELIXIR* infrastructure, this platform environment is an example providing advanced functionalities and tools. In order to find, register and benchmark software tools, the *ELIXIR Tools Platform* supports users to access, analyze and integrate data to drive scientific discoveries across the life sciences. The *ELIXIR Training Platform* strengthens user competences in DP and Tools Platform usage.

Researchers and funders accredit DPs’ importance in open data exchange, as DPs provide complex services and tools for better data management and stewardship within the life sciences. DPs’ improved scalability and high level of formalization of management mechanisms help to exploit vast data sets [37]. Digitalization and increasingly accessible computer systems have yielded new methods that allow efficient exploration and automated processing that improve data quality. Semantic concept schemas, for instance, contribute to a better description of statistical conclusions from data analyses [38]. DP managers put great effort into recombining datasets on platforms like the *RIKEN MetaDatabase* for healthcare and life sciences of linked open data [39] or the *Life Sciences Linked Open Data Cloud* [26]. For better analyses in cancer research, for example, a reproducible pharmacogenomic analysis workflow combines existing pharmacological and molecular profiles into one data object [40]. Software improvements in data harmonization enable user-friendly data utilization in Open Science in concrete application fields, like electrophysiology in neuroscience [41]. As of today, several organizations exist to improve IT-based research and services within natural sciences, like the *European Bioinformatics Institute* (*EMBL-EBI*). The European-Commission-funded scholarly participatory communication infrastructure *OpenAIRE* enables proper Open Science dissemination and training and operating technical services required to monitor and facilitate publishing trends and research impact across disciplinary boundaries [42]. *FAIRDOM* is another open consortium of services for research data management across disciplines providing an open source software platform in particular relevant in the field of Systems Biology [43].

### Guidelines for data management and stewardship in Open Science

Technical services alone do not guarantee the successful implementation of DPs. Hence, several European-funded initiatives and guidelines support researchers in the development of data management plans to correctly handle open data management and stewardship. The *European Research Council* enforces universal data sharing principles for their grantees [44]. Their guideline on data management plans for Open Science data requires sufficient dataset and protocol description, standards, persistent identifiers for datasets, information on curation and preservation methodology, and grantees’ data sharing methods. Management plans enforce *FAIR Principles* that ensure data findability, accessibility, interoperability and reusability [45]. Data stewardship focuses on tactical coordination and implementation responsible for establishing data quality metrics and other proper data management requirements to consistently provide easily accessible high-quality data. The *Data Stewardship Wizard* is a tool that practically supports researchers in creating useful data management plans [46]. Formal guidelines, like the *TRUST Principles*, support a platform`s trustworthiness [47]. They provide a framework to ensure that DPs are reliable and capable of appropriately managing the data they contain, offering sufficient transparency, responsibility, user focus technology and sustainability.

Despite all these efforts, data platforms in the life sciences still face the challenge of developing a holistic approach to platform management for their specific platform. Therefore, the focus of our study is the development of a framework for the holistic management of data platforms in the life sciences. We build on existing approaches in the life sciences and extend them against the background of our empirical observations and the broad management research on the governance and design of digital platforms in other scientific fields and industries [48].

## Materials and methods

**Fig 1:**
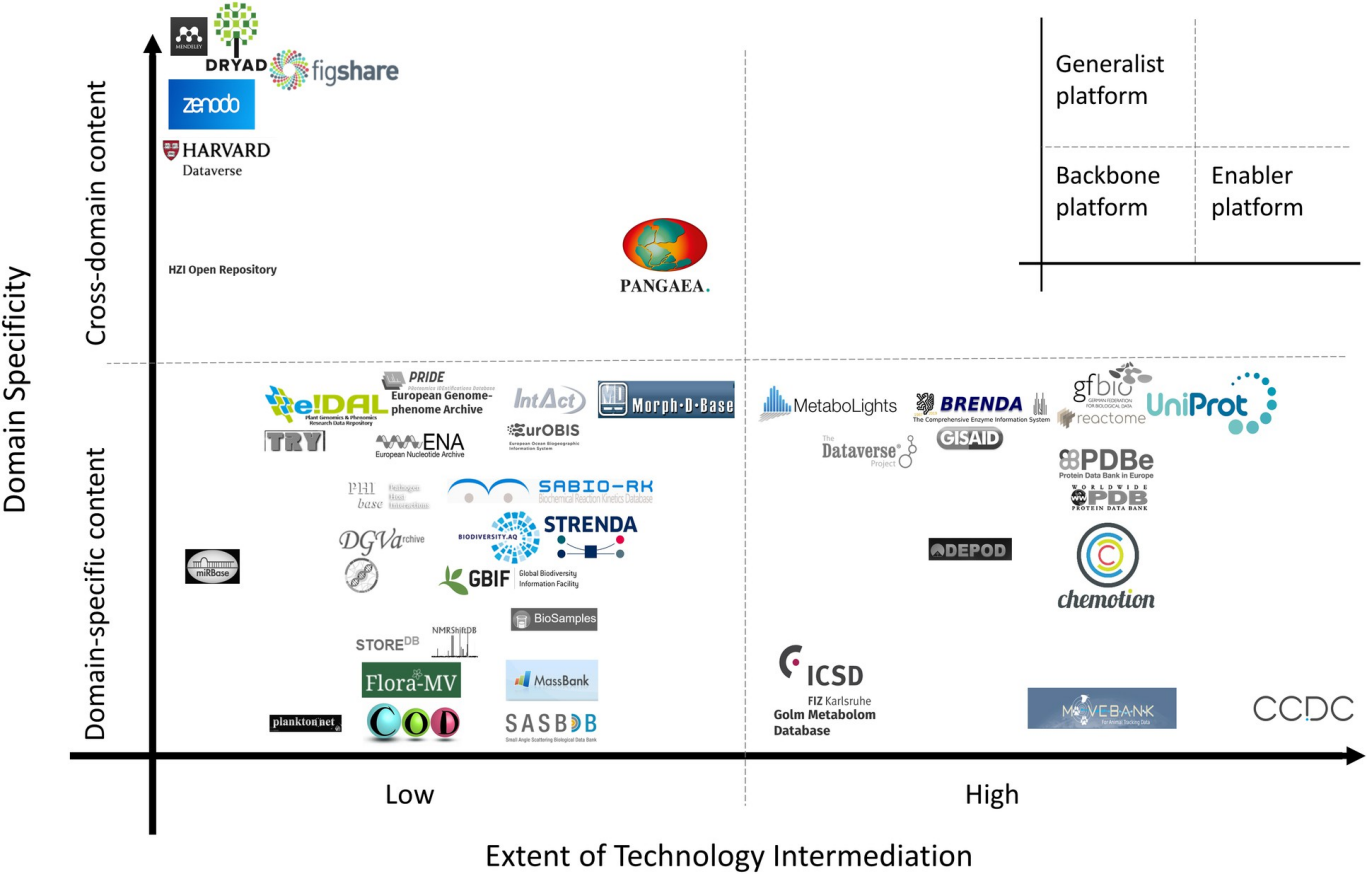


For our inductive qualitative study, we apply content analysis to primary textual data [49] to capture and gain insights from the meanings given to organizational phenomena, deriving themes that impact successful data platform management [50]. We investigate 223 platforms in the life sciences (see S1 Table) and complement our analysis of 39 qualitative in-depth interviews with experts from 22 DPs with secondary data from web pages and platform descriptions (for additional information on platforms and interviews, see S1–S3 Tables and for the interview transcription guideline, see S1 File). We approach the management instruments of DPs in two steps: First, we identify different types of platforms in the life sciences and categorize the DPs according to a 2x2 matrix in the dimensions “Extent of Technology Intermediation” (ETI) and “Domain Specificity” (DS) (see Fig 1 and Chapter Data platform types”). Second, we conduct a qualitative content analysis of the semi-structured interviews, to reveal the data sharing process and the management instruments that support the platforms’ success (see Table 2). First order concepts are summarized in the observed structures and activities aggregated to seven dimensions of effective DP management in the life sciences that determine successful data exchange.

The ETI shows the technology’s influence on the transaction process, and can be defined as the deployment of a software platform and its various digital tools as an intermediary that manages and coordinates the exchange between network actors [51]. The ETI plays an important role in choosing the right management instruments, since the more ambitious a platform is on a technical level, the more management challenges it may encounter [52]. The higher the number of the following features a DP offers, and the more advanced its tools, the higher its ETI: up- and download of data, (meta) data standards, data visualization, automatic checks of data with underlying data models, linkages to further resources, download tools, and beta stage tools.

The DS considers the scope of the life science domains that are covered, reflecting the heterogeneity of the included data. Research shows that data sharing practices [53] and user needs [54] depend on the scientific domain, and each domain may thus need specific management instruments. When DPs support only one or a few specific data types, there is a high DS value, while the DS value is low when users can upload different kinds of data and formats.

The following selection criteria were applied to identify relevant DPs for the interviews: All analyzed platforms are active in the life sciences, are publicly available (web pages, repositories, etc.), are not solely patient and material registries, and are not considered as “institute-only” DPs, or as internal project or consortium platforms. We also exclude databases that focus exclusively on mediation between platforms. Based on the selection criteria, we invited 45 suitable DP experts for interviews. 39 representatives from 22 DPs responded positively and took part in this study after verbal consent was informed. The representatives, which include researchers, senior managers, and advisory board members, cover a broad spectrum of users and DP operators from academia and industry. Their broad scope of domains within the life sciences allowed the identification of similarities and differences between the DPs. The interviews include management-related questions regarding the DPs’ specific history, core offerings, data transfer, allocation of responsibilities, decision-making processes, goals, challenges, and user groups, as well as governance-related questions on rules and requirements, functionalities, data reusability, incentives, trust and research culture (compare S2 File. Interview guideline and S3 File anonymized interviews).

### Results

In the result section, we distinguish DPs from other platform types and categorize them accordingly. Based on the interviews, we introduce the management instruments as aggregated dimensions of the coding process and present differences between DP types.

### Data platform types

223 platforms have been identified within life sciences and have been distinguished based on the parties they connect: users and data stocks, like repositories. *Data platforms* (1) enable users to upload their own data and download data from other users. Their core contribution is to store, publish, discover and give access to scientific data. Some DPs offer auxiliary tools and services, like data curation. In contrast, *Distributor platforms* (2) allow users to upload data and distribute it to other platforms, for example *GFBio*, that mediates data between the scientific community and various domain-specific data platforms [55]. *Aggregator platforms* (3) make extracted data from literature available to users without the possibility for users to upload data themselves, like *PharmGKB*, which curates knowledge on the impact of genetic variation on drug response. *Umbrella platforms* (4) centralize various cooperating platforms’ data stocks like the *Dataverse Project* [56] hosting multiple virtual archives (called *Dataverse collections*). Also, platform environments like the one within the ELIXIR infrastructure [35] provide an integrated view of several DPs, like in this case *Uniprot* [16] and *BRENDA* [57]. In our analysis, we focus on platforms that are considered as DPs since they mediate between user groups and are, thus, most sensitive to management instruments [31]. The 45 identified DPs (see S1 Table) were categorized using a developed 2x2 matrix based on the introduced dimensions ETI and DS (Fig 1); and three DP types were derived.

We differentiate between *Backbone*, *Generalist* and *Enabler* DP types (Fig 1) which offer different functions depending on the users’ needs. *Backbone* platforms (BP, low ETI, high DS) provide domain-specific communities with a central infrastructure for domain-specific data. BPs ensure that data meet certain criteria, they check data partially automatically, and they interlink data sets with other sources. *Generalist* platforms (GP, low ETI, low DS) offer the core service of uploading, storing, publishing, and discovering data with a minimal set of meta data standards, without being limited to specific domains. GPs can provide organizations with a solution that prevents them from using different DPs while enabling cross-domain data sharing. *Enabler* platforms (EP, high ETI, high DS) offer the scientific community both standardized and interlinked data, as well as tools for direct analysis of the data on the platform. Tools range from beta stages, word maps and simulations to complex and comprehensive analyses that can even include additional data from outside the platform. A fourth type (high ETI, low DS) would offer cross-domain advanced analysis tools directly on the platform, but does not exist in our data set. We mostly identified BPs (24), followed by EPs (14) and GPs (7).

### Management instruments

A qualitative content analysis of 39 interviews with 22 DP experts, supplemented with data from desk research, reveals seven management instruments that determine the success of DPs (Table 1). In the following section, we present and analyze these instruments based on the experts’ reflections on their own management activities and their observations within the field.

**Table 1:** Summary of data platforms’ management instruments deployed during the qualitative content analysis. The interviews reveal a difference in the identified management instruments for each data platform type.

Management instruments	Determinants: Observed structures and activities (examples / explanations)	Factor / Sub-unit	Generalist platforms	Backbone platform	Enabler platforms
**Organizational structure**	**Size** (Pool of DP employees measured in full time equivalents (FTE))	FTE Mean value / Median	17.0 / 15.0	4.4 / 2.1	27.4 / 8.0
		FTE Minimum / Maximum	4.0 / 40.0	1.0 / 27.0	4.5 / 80.0
	**Hierarchy** (Arrangement of employees: High vs. low direct vertical coordination)	1 Team / 2 Teams / > 2 Teams	20% / 20% / 60%	90% / 0% / 10%	43% / 29% / 29%
	**Team diversity** (Discipline background)	Computer science background	High	Medium	High
		Domain-specific background	Low	High	High
		Business administration, marketing and design, HR, diverse background	High	Low	Medium
	**Boards and committees** (Existence of advisory board, steering committee)		40%	30%	43%
**Technological infrastructure**	**State-of-the-art** (Response to challenges related to technological change)	Core Infrastructure	Storage space; covering individual use cases	Standardizing or expanding (meta) data; data duplication	Scaling tools with data evolvement, data complexity
	**Auxiliary tools** (Beta-stages, word maps, comprehensive tools)		No	No	Yes
	**Requirements for data upload** (Data size, format, (meta) data standards)	Fulfilling data standards	20%	80%	71%
		Fulfilling high meta data standards	20%	70%	100%
		Storage limitation	40%	0%	0%
		Any data format	80%	10%	0%
**Quality management**	**Manual curation** (Extent of platform intervention, plausibility, format checks)		60%	30%	71%
	**Automatic checks** (Scope of underlying data model)		40% low scope (meta data fields), 20% medium to high scope, 40% no automatic checks	80% medium to high scope (meta data and data checks), 20% no automatic checks	86% medium to high scope (meta data and data checks), 14% no automatic checks
	**External quality control** (data acceptance from peer-reviewed articles only)		0%	20%	29%
**Trust and credibility**	**Who favors property rights and terms of use?** (Availability of online documents)		Rather data user and author	Rather data user and author	Rather author or platform
	**Certifications** (External rewards and assessments like “CoreTrustSeal”)		20%	0%	43%
**Incentives (to motivate for data upload)**	**Recognition** (By other researchers providing measures like rankings or DOIs for citations)	Ranking	80%	0	0
		Providing DOI	100%	60%	57%
	**Platform outreaches** (Cooperation, directly addressed target groups)	Target group: Researchers	Medium scope	High scope	Low scope
		Target group: Universities, libraries and other institutions	High scope: Partnering and cooperating with institutions, targeting libraries, conferences	Low scope: Conferences and related events	High scope: Conferences and related events
		Target group: Industry	Low scope	-	Low scope
		Target group: Journals	High scope: Recommendations from journals	High scope: Pub-lishing articles, recommendations from journals	Medium scope: Recommendations from journals
	**Platform disclosure** (General information, statistics, publishing names)	Platform statistics	60%	70%	57%
		Publishing data supplier name	100%	40%	43%
		Direct contact possibility to data supplier	80%	40%	14%
		Linkage to publication(s) within data set	40%	50%	43%
		Data set statistics	80%	0	0
		Long term data availability statement	20%	0	0
	**External incentives** (Open Science policies of publishers /and funders)		High	High	High
**Openness**	**License agreements** (Taking pre-defined Licenses or offering inhouse Licenses, impact of Licenses, Licenses valid for whole platform or data set online)	Apache 2.0 License	0%	0%	14%
		CC for each data set	60%	30%	14%
		CC0 License	40%	0%	0%
		CC BY License	0%	30%	43%
		CC BY-NC License (or comparable)	0%	20%	14%
		No specified license	0	10%	0%
		Personal permission (data uploader remains owner)	0	10%	0%
		Ownership of data is transferred to platform	0	0%	14%
	**Involvement** (of the community in processes and procedures)	Surveys and workshops	Often	Rare	Medium
		Other forms of involvement	Ambassador programs	-	Events
	**Services for payment** (Analytical service, workshops)		0%	0%	14%
**Financing model**	**Revenues** (Licensing, memberships, fee for data depositions)	Research grants and project funding (Specific for data management)	60%	80%	86%
		Institutional funding (Hosting, employees, operation)	80%	80%	57%
		Licensing (Industry, academia, consortium)	40%	0%	29%
		Membership fee (Individual, consortium, data depositions)	20%	20%	14%

The interviewees consider the adoption rate, the number of data sets, and the usage numbers as measures of success. Successful management instruments include a clear organizational structure, a supportive technological infrastructure, and proper quality management. The DP must also appear trustworthy and open, offer incentives to its users, and provide a sustainable financing model. Regarding the organizational structure, the DPs differ in size, hierarchies, team diversity and the additional formal involvement of boards and committees. Structures are especially important for DPs that have a large community with diverse content. The organizational structure must remain dynamic to face changing environments.

Based on the insights from the expert interviews, we believe that a supportive technological infrastructure increases the platforms’ success. As is the case in other disciplines, state-of-the-art tools, including mechanisms for optimal standardization and structure without the risk of losing important content, deeper and more specific analyses, and links between data, are important for new data, but also for maintenance of the existing data pool [58, 59]. Concrete technologies include text mining and artificial intelligence approaches [60, 61] for both data management and for users’ search tools. All data provided must meet the various users’ criteria for easy data findability, accessibility, interoperability, and reusability, to allow for use for their specific needs [45]. These needs are especially diverse within the life sciences, and it is therefore important to find a common base for data exchange. DP user rules on data upload, meeting proper scientific practices and prevailing norms, for instance, are extremely important, and their communication and enforcement must consequently be ensured. Clearly communicated data standards are therefore necessary to motivate data uploads, during which process auxiliary tools support the fulfilling of the upload requirements.

An effective quality management system must ensure that user guidelines, such as complying with data upload requirements and the correct scientific usage of data, are developed, enforced, and monitored. According to the interviewees, this measure should, however, remain user-friendly rather than create an additional burden that discourages users from uploading data. The experts highlight constant maintenance services, including automated checks and manual curation, as additional operational management tasks which ensure high data quality. Some DPs further integrate external quality controls by only publishing data from peer-reviewed articles.

To achieve a high level of trust and credibility, property rights must be distributed well. Common ways to do so are licenses or standardized digital documents that can be signed online. Process transparency must be ensured at all times by, for example, providing information on data sources. When users experience the platform and data as transparent, data transactions take place. Certifications awarded by external assessments and reviewing authorities also signal trust, credibility and proficiency, and motivate researchers to upload data.

We distinguish between internal and external incentives for the DP users motivating for data sharing. Researchers are the main data contributors. Internally, many platforms establish incentive schemes mainly related to improved recognition, which is a major motivational factor for researchers [62]. By offering a DOI, for instance, these researchers can be cited in and indirectly contribute to external studies. The interviewees also mention rankings by, for example, highlighting highly active data contributors. Further motivation for new entrants includes platform disclosure of contributors, partners, and supporters and actively promoting its outreach. All DPs rely on external incentives, including obligations to publishers and funders to follow Open Science policies, including publishing data in open access databases.

The platform must advertise and prove its open-access format and transparency to its users. As such, license agreements like the CC License Family or Apache License build a platform usage framework, and the choice of general licensing conditions for either the entire platform or for individual data sets also influences data reusability. Involving its users and uplifting a community creates mutual commitment and strengthens the DP. The DP’s management must therefore allow its users direct involvement and respond to their needs and suggestions. Furthermore, expanded services, such as links beyond the individual database, signal openness and may also support users to easily exploit further databases. In addition, transparency is of increased importance for data users as it is required by funding agencies, policy makers and other stakeholders, including researchers and publishers.

Finally, the financing model must be sustainable. For smaller platforms related to single research projects, specifically, maintenance is often only guaranteed for a short period after the project’s finalization. The interviewees state that research driven platforms, especially, fail to focus on the necessary revenue options for a database’s continuation. Such revenue possibilities include licensing and membership models, institutional financing, and research grants. Nevertheless, funded platforms must carefully balance their revenue or funding sources, as this may impact their autonomy or strategic positioning, contradicting Open Science policies. Research funders have identified the challenges in platform sustainability due to financial restrictions, and form alliances like the *Global Biodata Coalition* that mainly focuses on core data to better coordinate and share approaches to the efficient management and growth of freely available biodata resources [63]. These alliances ensure long-term financial aid for a global biodata infrastructure and support global core resources that are crucial for sustaining the broader infrastructure.

The interviews also reveal the differences between the DP types regarding utilized management instruments (Table 2). BPs offer high volumes of domain-specific data and address the specific needs of communities by enabling data intensive analyses. Having the lowest average level of resources, they rely on citations and often engage in open access formats. EPs are especially user friendly, as they create a centralized location for standardized data and feature a wide range of additional functions at the cost of personnel intensity. GPs comprise vast data and user diversity at the cost of standardization and technological involvement. They reach a broad pool of users and allow for the utilization of diverse datasets without connecting to other DPs (more details in the S4 Table and S4 File).

**Table 2:** Comparisons of management instruments.

Management instruments	Determinants: Observed structures and activities (examples / explanations)	Differences between platform types
**Organizational structure**	**Size** (Pool of DP employees measured in full time equivalents (FTE))	On average, EPs have the largest pool of employees, followed by GPs and BPs. By offering tools, EPs depend on personnel intensive work, like creating tools and maintaining corresponding data quality. GPs cover a wide scope of scientific domains resulting in a diverse user community, as well as high data volumes and use cases. BPs cover certain use cases in a specific domain with comparably low FTE values.
	**Hierarchy** (Arrangement of employees: High vs. low direct vertical coordination)	In general, the higher the number of FTEs of a DP, the more likely it is that two or more teams exist, requiring more extensive vertical and horizontal coordination in the organization. While GPs often consist of more than two teams, BPs usually consist of one team only.
	**Team diversity** (Disciplinary background)	All DPs rely on personnel with IT and computer science backgrounds to set up, maintain and advance the platform. The educational background of DPs personnel with high domain specificity (BP, EP) is often related to the domain-specific background. GPs favor a diverse educational background, as there is no anchoring within one particular domain. Employees with a diverse background signal a broader knowledge frontier, enabling the recombination of cross-domain knowledge, which is vital for innovative governance models.
	**Boards and committees** (Existence of advisory board, steering committee)	The existence of advisory boards and steering committees is comparable between all DP types, and smaller BPs also have such entities.
**Technological infrastructure**	**State-of-the-art** (Response to challenges related to technological change)	EPs face challenges regarding the ambidexterity of simultaneously increasing data complexity and offering tools to users. Adapting existing tools to evolving data sets remains especially challenging.
		For BPs, the degree of transparency remains important, as they facilitate data exchanges in specific data domains by functioning as a major storage space. Current challenges experienced by BPs comprise standardizing and expanding (meta) data, as well as data duplication.
		The technologies of GPs are designed for metadata and data of many or even all domains, so that the platform can be linked to other (external) organizations. Thus, the architecture must be designed to link the scattered components of the infrastructure (e.g., from researchers, libraries, and journals) making APIs the key gateway technology for GPs. Challenges include storage space, covering individual use cases, and the identification of tools that enable data comparison (for a shift towards higher technology involvement).
	**Auxiliary tools** (Beta-stages, word maps, comprehensive tools)	EPs make it possible to directly discover data on the platform using tools. Data must be available in a standardized form to best apply data exploitation tools. For EPs, a crucial success factor is the extent to which additional functions and services relate directly to the semantic dimension of its data. As GPs and BPs rarely offer facilitating tools for analysis, easy usability and accessibility of the platform is mainly considered beneficial to the users.
	**Requirements for data upload** (Data size, format, (meta) data standards)	EPs and BPs are characterized by extensive (meta) data upload requirements, limiting the data format to upload. GPs have lower (meta) data requirements, mostly allowing for any data format, while storage limitations can apply.
**Quality management**	**Manual curation** (Extent of platform intervention, plausibility, format checks)	There are rather strict upload requirements for EPs, ensuring higher data quality and allowing for the use of tools to analyze standardized data. BPs facilitate the standardization of domain-specific data, which is linked to the perceived data quality. Quality can be ensured by manual curation, automatic checks, and outsourcing. Manual curation does, however, become time consuming with increasing data volumes. Since BPs have particularly low FTE values, they depend on increasing automation of data upload controls, which simultaneously enforces standardization and data quality checks. One third of the EPs and 20% of the BPs interviewed only allowed data from peer-reviewed papers that enhanced the data quality. GPs face challenges regarding the data quality offered. It is particularly noticeable that GPs with no domain restriction enforce lower metadata standards, and often have no data standards at all. A DP does not necessarily have to offer high quality data if it is not the decisive criterion for the user group.
	**Automatic checks** (Scope of underlying data model)	
	**External quality control** (Data accepted from peer-reviewed articles only)	
**Trust and credibility**	**Who favors property rights and terms of use** (Availability of online documents)	The property rights favor the author when the author has free choice about the specified License, or if the License enforces citations of the data. The data user is favored when no citation of used data is needed. The property rights favor the platform when the ownership is transferred to the platform. The property rights of EPs favor the author (need to cite) or the platform itself (transferring ownership rights). In the case of GPs and BPs, property rights mostly favor the data user (choice of License) and author (need to cite).
	**Certifications** (External rewards and assessments, like “CoreTrustSeal”)	Certifications are mostly received by EPs, followed by GPs. Interviewees mostly cite certifications as indicators of institutional-based trust. No BP in our analysis has been certified.
**Incentives (Motivation for data upload)**	**Recognition** (By other researchers providing measures like rankings or DOIs for citations)	A dataset becomes citable by providing a DOI. A DOI is an incentive for data suppliers, as it acknowledges the work. In our analysis, most GPs already provide DOIs and publish rankings that enable better recognition of the data supplier on their website. In comparison, not all BPs and EPs provide DOIs.
	**Platform outreaches** (Cooperation, directly addressed target groups)	All DPs attract new users from journal recommendations. EPs specifically, frequently advertise their high functionalities. Due to scare resources, BPs reach out to their users at scientific conferences and presentations, and by publishing research articles. Users of GPs require diverse data for their analyses, and therefore cooperate with institutions such as universities and libraries.
	**Platform disclosure** (General information, statistics, publishing names)	GPs follow a strong platform disclosure strategy by publishing data set statistics and the name of the data provider, and by offering contact possibilities, like posting it on the main page and showing statistics for individual data sets. One GP within the sample provides a long-term data availability statement. EPs disclose the least amount of information about the data (e.g., statistics) and authors (e.g., name and contact possibilities) on the platform. The BPs disclose more information regarding contact possibilities.
	**External incentives** (Open Science policies of publishers and funders)	No differences of external incentives between DP types have been observed. All DPs benefit from external incentives, like Open Science policies of publishers and funders.
**Openness**	**License agreements** (Using pre-defined Licenses or offering inhouse Licenses, impact of Licenses, Licenses valid for whole platform or data set online)	The License agreements of EPs vary in scope impact. Regarding the scope, Licenses may cover the whole platform or individual data sets only. Regarding the impact, EPs show all identified License agreements. BPs mainly comprise CC BY and CC BY-NC Licenses. It follows that data suppliers and the DP are cited by researchers when reusing them. Despite the absence of a DP citation index, citations of used data promote the work of the DP in the scientific community. For GPs, it is noticeable that either the users themselves can determine the CC-License or the entire platform is subject to the CC0 License.
	**Involvement** (Of the community in processes and procedures)	Based on available resources, different DPs perform different tasks in community involvement. GPs conduct most surveys and workshops, followed by EPs. Another form of involvement is an ambassador program in which users can actively participate. EPs also offer further services, like tutorials, and events, such as training, to their communities. When EPs publish blog articles or spread information about news and events, they also act as a social space for the scientific community, offering users a communication platform. At this point, they can use the opportunity to actively participate in the discussion, influencing processes and procedures, and respond to their communities’ needs. Most BPs occasionally offer workshops, and then only case-based ones.
	**Services for payment** (Analytical service, workshops)	One EP offers analytical services for data payment.
**Financing model**	**Revenues** (Licensing, memberships, fee for data deposition)	The biggest share of DPs receives research grants, and most research projects include database funding. Funding is especially important for BPs, as their users (mostly researchers) show the least willingness to contribute financially. Most DPs have received public funding in the early development stages. GPs are often beyond the initial (public) funding phase. In some cases, organizations follow up on public funding after their expiration. This has been observed for most BPs, but is also the usual procedure for the other types’ platforms. Institutional funding often enables BPs to extend their limited resources and develop into EPs or DPs. Licensing for additional services is most common for GPs, as their users are willing to pay for additional services, like standardized data. None of the analyzed BPs use Licensing models. Membership fees are rare within the sample, as open DPs have been the focus. Yet, such models do arise with the progression of DPs.

## Discussion

Our exploratory research is the first in-depth study to reveal management structures and activities that determine efficient data exchange on DPs within the life sciences. It adds to the current literature by improving the establishment and operation of DPs to increase the diffusion of the DP and to better exploit the data provided. A successful DP satisfies the needs of the community and, in turn, the value of a platform increases with the number of their users. These network effects on the one hand directly increase the value of the DP due to a larger volume of shared data. On the other hand, indirect network effects result from an enlargement of the offered services of the DP providers or associated service providers, because both have stronger incentives to offer specific and valuable analysis tools [64, 65]. Therefore, it is important to motivate the community by being open, credible, and trustworthy, and by offering incentives for data uploads and guaranteeing high data quality. These findings are in line with the call for research policies that better incentivize data sharing [66] to overcome existing barriers to effective data sharing and preservation [67].

The value of DPs for its users, and thus a DP`s success, also increases with a higher reuse of stored data. License agreements, rules and requirements, including scientific practices and prevailing norms, build a formal structure determining the extent to which data may be reused. To increase data exchanges with other DPs, a supportive, open organizational structure and the latest state-of-the-art technologies are needed. Consequently, quality management should ensure the interlinking of platforms and (standardized) data, as well as following FAIR principles for DP success [45]. In accordance with Wilkinson et al., we support the importance of a transparent evaluation framework to promote digital resource fairness, which assists data stewards [68]. Roadmaps for scientific publishers [69] and scholarly data repositories [70] may support the implementation of proper data citation.

Beyond the importance of individual management instruments for increasing the performance of the platform, the results show the relevance of a holistic approach. We suggest seven management instruments that have potentially not only additive but also multiplicative effects: Organizational structure, Technological infrastructure, Quality management, Trust and credibility, Incentives, Openness, and Financing model. The manifestation of each of the seven dimensions determines the choice and effect of other elements. Thus, for instance, a strong organizational centralization and adequate resource base of the platform management goes hand in hand with an expansion of the quality management. Efficient quality management requires an efficient infrastructure and at the same time increases trust in the platform. Trust, as a key determinant of performance, is in turn dependent on the choice of the incentive system, the level of openness, and the financing model. We believe that it is this ability of a DP`s management to think in such systemic terms that distinguishes successful platforms from unsuccessful ones.

In the dynamic life science environment the nature, size and heterogeneity of relevant data, as well as the user demands and regulatory requirements, are continuously changing [20, 71, 72]. Therefore, management instruments must adopt technological advances and DP managers should remain flexible and versatile to quickly adapt to environmental changes and user demands by adjusting their existing resources. Constant screening of the environment (e.g., competitors’ activities), a re-evaluation of its potential impact on the database, and actions to react, sharpen DPs’ dynamic capabilities [73].

Although the seven management instruments (Table 1) apply to all DPs, there are different success factors for the DP types, and individual strategies can be derived for each (Table 2). Compared to other DP types, standardization enforcement is simpler for BPs, since they have the narrowest focus. BPs can develop profound scientific expertise within their niche and apply domain-specific technologies enabling them to provide high quality data. Metadata, in particular, needs to meet domain-specific standards [45]. On the downside, BPs often face short funding cycles and need to rapidly establish themselves within their respective community, emphasizing their importance to be openly accessible, and ensuring longevity. EPs create a central location for standardized data and lower various transaction costs. They can connect their communities through extensive services and provide additional technologies that standardize and interlink data. These services require high levels of personnel involvement, leading to higher costs. EPs create switching costs for their users and tend to lean towards License-based models, with more restrictive models at the cost of openness. GPs are of definite value within the life sciences if they reach a broad community and provide diverse datasets. We therefore observe a strong platform disclosure strategy, as GPs often highlight names and affiliations of their data suppliers. They enable cross-domain data recombination. In addition, they reduce multi-homing costs for their communities providing access to several data sets. GPs must be able to connect to several technical infrastructures and rely heavily on the latest state-of-the-art data management tools and IT specialists for execution. Consequently, they cannot provide profound scientific expertise, and should rather outsource quality management and cooperate with other platforms. Despite comprising diverse datasets, GPs should also concentrate their resources, for example, by focusing on a specific customer group, application field, or scientific area to create additional value for their users.

Finally, we turn to trends we observed for DPs. First, anticipating the future publishing routines of a “publish first, curate later” approach [74], we presume that user communities will become increasingly powerful. This approach integrates community feedback, emphasizing the importance of transparency, peer-mediated improvement, and post-publication appraisal of scientific data. It therefore highlights the importance of user involvement, not only in simple data uploading, but also in co-creating platform management structures. Second, the trend of increasing the number of databases fuels the competition for additional users and data. In addition, the declining of brand and company loyalty in other industries [75–77] may also increase users’ willingness to change platforms in the sciences domain, thus emphasizing the importance of user retention through qualitative services and fair contracts. Third, to this day, despite existing guidelines and principles, we observe challenges in the practical enforcement of universal data standards, specifically, among different BPs. Domain-specific BPs often optimize conditions for their individual niche and user group they originally serve [45], hindering new entrants from utilizing the platform for (meta) data analyses. In addition, their individual data rules and requirements may hamper easy data upload and usage, as users must first acquire each DP’s individual operation guidelines. Thus, building on what has been observed in narrower industries [78], especially in the life sciences there will be an ongoing need for ambidexterity of DP providers to address specific user groups’ needs and to reach a diverse audience at the cost of specificity.

## Conclusions

We contribute to the discussion on the increasing importance of DPs in the life sciences. As intermediaries, DPs form a bottleneck and can significantly influence the scientific impact of efficient data utilization. Our study integrates a management and governance perspective that reveals several implications for research and data management.

Our selection criteria for DPs are in line with those by the *ELIXIR Core Data Resources* based on the DPs’ scientific quality, their communities, service quality, governance infrastructure, and their impact [79]. These resources provide a fundamental infrastructure for the life sciences and the long-term preservation of biological data [80].

The research design, as a cross-case study, reflects the current state of the examined DPs in the life sciences. Sadiq and Indulska [81] state that “the relationship between data quality, intention to use, and the effective use of data remains unexplored in academic literature.” We address this research gap and provide a DP management guideline for the life sciences. We extend the existing TRUST Principles [47] by adding further dimensions of management instruments, such as the organizational structure, openness, incentives and financial aspects. Further research could use this list of required competences and could monitor the platform development over time. Such longitudinal analysis may allow researchers to determine the actual impact of single management instruments and their combination on DP success. Our framework of seven management instruments describes the determinants of successful data platforms, and provides a starting point for the measurement of a DP`s management capabilities for such an empirical analysis.

Turning to the managerial implications, we find that awareness of the platform type and understanding its implication is the first step in effective DP management. The three identified data platform types, which are based on the dimensions “extent of technology intermediation” and “domain specificity”, can be applied to further platforms outside of our sample. GPs must, for example, be able to connect to several technical infrastructures, such as those at universities and journals and, to this purpose, invest in keeping their technology at state-of-the-art levels. Increasing the automation of data upload controls, which simultaneously enforce standardization and platform rules, allows users, especially BPs, to react to resource constraints. By offering technical tools, EPs have the unique opportunity to simultaneously create incentives and build trust with users. Our guideline to structure and to operate DPs offers a holistic approach for DP managers. An application of our guideline of management instruments, including activities for successful data exchange, supports the further exploitation of the open life science’s potential.

## Supporting information

S1 Table Overview of platforms. (DOCX)

S2 Table Overview of collected characteristics and values. (DOCX)

S3 Table Additional information on interviews. (DOCX)

S4 Table Definition table of applied terms and concepts. (DOCX)

S1 File Interview transcription guideline. (DOCX)

S2 File Interview guideline. (DOCX)

S3 File Interview transcripts (anonymized). (PDF)

S4 File Detailed information on data platform types. (DOCX)
