# Supplementary material for: Data platforms for open life sciences–A systematic analysis of management instruments
Source: PLoS One. 2022 Oct 25;17(10):e0276204. doi: 10.1371/journal.pone.0276204 (PMC9595524; doi:10.1371/journal.pone.0276204)
Supplement: S1 Table — (DOCX) [file pone.0276204.s001.docx]

# S1. Table. Overview of platforms

| **Platform ID** | **Platform name** | **URL [last retrieved on 06.07.2022]** |
| --- | --- | --- |
| 1 | AgeGuess Database | https://www.nature.com/articles/s41597-019-0245-9 |
| 2 | Aligned Ribosomal Complexes Database | http://darcsite.genzentrum.lmu.de/darc/ |
| 3 | ArrayExpress | https://www.ebi.ac.uk/arrayexpress/ |
| 4 | ARS Antibiotika-Resistenz-Surveillance | https://www.re3data.org/repository/r3d100010398 |
| 5 | BacDIVE | https://bacdive.dsmz.de |
| 6 | BExIS Biodiversity Exploratories Information System | https://www.bexis.uni-jena.de/Login/Account.aspx |
| 7 | bigg (by University of California) | http://bigg.ucsd.edu |
| 8 | BioCyc | https://biocyc.org/ |
| 9 | Biogrid | https://thebiogrid.org |
| 10 | Biological Collection Access Service for Europe (BioCASE) | https://www.biocase.org/ |
| 11 | BioModels Database | http://www.ebi.ac.uk/biomodels-main/ |
| 12 | *BioSD** | https://www.ebi.ac.uk/biosamples/ |
| 13 | **BRENDA*** | https://www.brenda-enzymes.org/ |
| 14 | **Cambridge Structural Database*** | https://www.ccdc.cam.ac.uk/solutions/csd-system/components/csd/ |
| 15 | CAMPR3: a database on sequences, structures and signatures of antimicrobial peptides | https://academic.oup.com/nar/article/44/D1/D1094/2502595 |
| 16 | Cancer Imaging Archive | https://www.cancerimagingarchive.net/about-the-cancer-imaging-archive-tcia/# |
| 17 | Cataloge of life | http://www.catalogueoflife.org |
| 18 | Cellfinder | http://cellfinder.org |
| 19 | Center for Open Science | https://cos.io/contact/ |
| 20 | Center for Open Science / Open Science Framework (OSF) | https://osf.io |
| 21 | Central database, communication platform and data synthesis of the research unit KiLi | https://gepris.dfg.de/gepris/projekt/163561546?language=en |
| 22 | CentraXX Bio (by IQVIA) | https://www.kairos.de/produkte/centraxx-bio/ |
| 23 | ChEMBL - Neglected Tropical Disease | https://www.ebi.ac.uk/chembl/ |
| 24 | Chemical Entities of Biological Interest | https://www.ebi.ac.uk/chebi/aboutChebiForward.do |
| 25 | **Chemotion*** | https://chemotion.net |
| 26 | Chemspider | http://www.chemspider.com |
| 27 | Collaborative resource for the Bacillus community. | http://subtiwiki.uni-goettingen.de |
| 28 | Cologne Open Science - Wissenschaft weltweit vernetzen | https://cos.bibl.th-koeln.de/home |
| 29 | Combase | https://www.combase.cc/index.php/en/contact-us |
| 30 | Comprehensive Enzyme Information System | https://www.brenda-enzymes.org |
| 31 | CORUM – The Comprehensive Resource of Mammalian protein complexes | https://clarivate.com/webofsciencegroup/master-data-repository-list/ |
| 32 | Cotton Database Resources | https://www.cottongen.org/ |
| 33 | **Crystallography Open Database*** | https://www.crystallography.net/cod/index.php |
| 34 | *Database of Genomic Variants archive** | https://www.ebi.ac.uk/dgva/ |
| 35 | Database of RNA interactions in post-transcriptional regulation | https://dorina.mdc-berlin.de/regulators |
| 36 | DataOne | https://www.dataone.org |
| 37 | Dataverse | https://dataverse.org |
| 38 | *DEPOD (DEPhOsphorylation Database)** | http://depod.bioss.uni-freiburg.de |
| 39 | DESY Publication Database | https://bib-pubdb1.desy.de/youraccount/login?ln=en&amp;referer=https%3A%2F%2Fbib-pubdb1.desy.de%2Fsubmit%3Fln%3Den |
| 40 | Deutsches Biobankenregister | http://www.biobanken.de |
| 41 | Deutsches Klimarechenzentrum | https://www.dkrz.de/en/dkrz-partner-for-climate-research?set_language=en |
| 42 | Deutsches Register Klinischer Studien | https://www.drks.de/drks_web/ |
| 43 | DGVarchive | https://www.ebi.ac.uk/dgva/ |
| 44 | DNA Data Bank of Japan | https://www.ddbj.nig.ac.jp/submission-e.html |
| 45 | Dokumentenserver Klimawandel | http://edoc.sub.uni-hamburg.de/klimawandel/home |
| 46 | doRiNA | https://dorina.mdc-berlin.de |
| 47 | DRAMP 2.0 | https://www.nature.com/articles/s41597-019-0154-y |
| 48 | DrugBank | https://www.drugbank.ca |
| 49 | Drugbank plus | https://www.drugbankplus.com/?_ga=2.135955767.742706252.1575898367-335313615.1575898367 |
| 50 | Drug-related information: SuperTarget | http://bioinformatics.charite.de/supertarget/ |
| 51 | **Dryad Digital Repository*** | https://datadryad.org/stash |
| 52 | Duraspace | https://duraspace.org/dspace/ |
| 53 | EcoCyc | https://ecocyc.org/ |
| 54 | EDI Data Portal | https://portal.edirepository.org/nis/contact.jsp |
| 55 | *EGA European Genome-Phenome Archive** | https://www.ebi.ac.uk/ega/submission#Key_steps_for_all_EGA_submissions |
| 56 | Elixir | https://elixir-europe.org/platforms/data/core-data-resources |
| 57 | EMDataResource | https://www.emdataresource.org/deposit.html |
| 58 | *ENA European Nucleotide Archive** | https://www.ebi.ac.uk/ena |
| 59 | Ensemble (by EBI) | https://www.ensembl.org/index.html |
| 60 | Environmental Information Data Centre | http://eidc.ceh.ac.uk |
| 61 | Essential Biodiversity Variables | https://geobon.org/about/get-involved/ |
| 62 | Establishment of the Biopolis Dresden Imaging Platform (BioDIP) | https://gepris.dfg.de/gepris/projekt/214014982 |
| 63 | EuPathDB (Eukaryotic Pathogen Database Resources) | https://eupathdb.org/eupathdb/ |
| 64 | Eurobis | http://www.eurobis.org/data_access_services |
| 65 | EUROCarbDB | https://bio.tools/eurocarbdb |
| 66 | European Bioinformatics Institute | https://www.ebi.ac.uk/ |
| 67 | European Chemical Biology Database | ecbd@ebi.ac.uk |
| 68 | European Open Science Cloud | https://eosc-portal.eu/ |
| 69 | *European Vegetation Archive (Eva)** | http://euroveg.org/eva-database |
| 70 | Exposome-Explorer | http://exposome-explorer.iarc.fr |
| 71 | EyeMoviePedia | http://www.eyemoviepedia.com |
| 72 | Fairdom | https://fairdomhub.org |
| 73 | FAIRDOM-SEEK | https://seek4science.org/about_us.html |
| 74 | **Figshare repository*** | https://figshare.com/about |
| 75 | Flechten Lichen Metabolites | https://www.nature.com/articles/s41597-019-0305-1.pdf |
| 76 | Flora von Bayern | https://wiki.bayernflora.de/web/Hauptseite |
| 77 | ForestPlots.net | https://www.forestplots.net |
| 78 | GBOL (German Barcode Of Life) | https://www.bolgermany.de |
| 79 | Genbank | https://www.ncbi.nlm.nih.gov/genbank/ |
| 80 | Gene Ontology | http://geneontology.org |
| 81 | GeneDB | https://www.genedb.org |
| 82 | General Unrestricted Structure-Activity Relationships "GUSAR" | http://genexplain.com/gusar/ |
| 83 | GenomeRNAi | http://www.genomernai.org |
| 84 | Genomic Expression Archive (GEA) | http://www.genomernai.org |
| 85 | GeoReM | http://georem.mpch-mainz.gwdg.de |
| 86 | Geosciences Collection Access Service | http://geocase.eu/access |
| 87 | German Network for Bioinformatics Infrastructure (de.nbi) | https://www.denbi.de/ |
| 88 | Gesellschaft für wissenschaftliche Datenverarbeitung mbH Göttingen | https://www.gwdg.de/ |
| 89 | *GFBio** | https://www.gfbio.org |
| 90 | GGBN Wiki | <https://wiki.ggbn.org/ggbn/Join_the_Network> |
| 91 | *GISAID EpiFlu database** | https://www.gisaid.org/ |
| 92 | **Global Biodiversity Information Facility*** | https://www.gbif.org |
| 93 | **Global Index of Vegetation-Plot Databases*** | http://www.givd.info |
| 94 | GlyTouCan | https://glytoucan.org |
| 95 | *Golm Metabolome Database** | http://gmd.mpimp-golm.mpg.de |
| 96 | GWAS Central | https://www.gwascentral.org |
| 97 | **Harvard Dataverse*** | https://dataverse.harvard.edu/ |
| 98 | heiDATA Dataverse Network | https://clarivate.com/webofsciencegroup/master-data-repository-list/ |
| 99 | *Helmholtz-Zentrum für Infektionsforschung Open Repository** | https://repository.helmholtz-hzi.de/pages/submitting-content |
| 100 | HENA, heterogeneous network-based data set for Alzheimer’s disease | https://github.com/esugis/hena |
| 101 | HeRBi - Helmholtz Repository of Bioparts | http://www.herbi.kit.edu/57.php |
| 102 | High Throughput Experimental Materials Database (HTEM DB) | https://www.nature.com/articles/sdata201853 |
| 103 | Human cerebrospinal fluid metabolome database | http://www.csfmetabolome.ca |
| 104 | Human Metabolome Database | http://www.hmdb.ca |
| 105 | Human protein-protein interaction network database search | http://artemis.mdc-berlin.de/y2h_network/ppi_search.php |
| 106 | Human Urine Metabolom | http://www.urinemetabolome.ca |
| 107 | HumanPSD | http://genexplain.com/humanpsd/ |
| 108 | ICGC Data Portal | https://dcc.icgc.org/team |
| 109 | i-Marine | http://www.i-marine.eu |
| 110 | Immuno Polymorphism Database | https://www.ebi.ac.uk/ipd/ |
| 111 | Informations-Infrastruktur-Projekt (INF) | https://gepris.dfg.de/gepris/projekt/269768312?context=projekt&task=showDetail&id=269768312& |
| 112 | **Inorganic crystal structures database ICSD*** | https://icsd.products.fiz-karlsruhe.de |
| 113 | INSDC USA by the National Center for Biotechnology Information | https://www.ncbi.nlm.nih.gov/home/about/mission/ |
| 114 | *Intact** | http://www.ebi.ac.uk/intact/ |
| 115 | Intenz | https://www.ebi.ac.uk/intenz/ |
| 116 | International Center for Diffraction Data | http://www.icdd.com |
| 117 | International Mouse Phenotyping Consortium | https://www.mousephenotype.org/ |
| 118 | International Nucleotide Sequence Database Collaboration | https://www.insdc.org/ |
| 119 | International Nucleotide Sequence Database Collaboration (INSDC | http://www.insdc.org |
| 120 | International Union of Basic and Clinical Pharmacology Guide to Pharmacology | https://www.guidetopharmacology.org |
| 121 | InterPro | https://www.ebi.ac.uk/interpro/ |
| 122 | JuSER | https://juser.fz-juelich.de/?ln=de |
| 123 | Kalium 2.0 | https://www.nature.com/articles/s41597-019-0074-x |
| 124 | Kent Data Repository | https://data.kent.ac.uk/ |
| 125 | KiMoSys | https://www.kimosys.org |
| 126 | KITopen | https://www.bibliothek.kit.edu/cms/kitopen.php |
| 127 | Knowledge Network for Biocomplexity | https://knb.ecoinformatics.org/about |
| 128 | Knowledge portal LSBTI² | https://wissensportal-lsbti.de/ |
| 129 | Kyoto Encyclopedia of Genes and Genomes | https://www.genome.jp/kegg/ |
| 130 | LAMP2: A Database Linking Antimicrobial Peptides | https://journals.plos.org/plosone/article?id=10.1371/journal.pone.0066557 |
| 131 | Leibniz Institute DSMZ-German Collection of Microorganisms and Cell Cultures | https://www.dsmz.de/ |
| 132 | Leipzig Health Atlas | https://www.health-atlas.de/ |
| 133 | LIVIVO - The Search Portal for Life Sciences | https://www.livivo.de |
| 134 | Locus Specific Databases | https://grenada.lumc.nl/LSDB_list/lsdbs |
| 135 | Machine Learning for Pharmaceutical Discovery and Synthesis Consortium | <https://mlpds.mit.edu/> |
| 136 | Mannheim Research Data Repository | https://madata.bib.uni-mannheim.de |
| 137 | Marine Ecological GenomiX (megx) | http://mb3is.megx.net |
| 138 | Mass Spectrometry Interactive Virtual Environment | https://massive.ucsd.edu/ProteoSAFe/static/massive.jsp |
| 139 | Mass Spectrometry Platform | https://gepris.dfg.de/gepris/projekt/239825530 |
| 140 | Mass Spectrometry Platform (for lipid, protein, & glycoconjugate analyses) | https://gepris.dfg.de/gepris/projekt/239825530?context=projekt&task=showDetail&id=239825530& |
| 141 | **MassBank*** | http://www.massbank.jp/ |
| 142 | Mcule | https://mcule.com/ |
| 143 | *Mendeley Data** | https://data.mendeley.com/ |
| 144 | **MetaboLights*** | http://www.ebi.ac.uk/metabolights |
| 145 | MetaCyc | https://metacyc.org |
| 146 | MG-RAST | https://www.mg-rast.org |
| 147 | *microRNA database "miRBase"** | http://www.mirbase.org |
| 148 | "Miriam" - Minimal Information Required in the Annotation of Models | http://co.mbine.org/standards/miriam |
| 149 | **MorphDBase*** | https://www.morphdbase.de |
| 150 | **Movebank Data Repository*** | https://www.datarepository.movebank.org |
| 151 | National Decade against Cancer (of Germany) | https://www.dekade-gegen-krebs.de/en/home/home_node.html |
| 152 | Nationale Forschungsplattform für Zoonosen | https://www.zoonosen.net |
| 153 | Nationale Kohorte | http://nako.de/ |
| 154 | NERC Data Center | https://nerc.ukri.org/ |
| 155 | Network Data Exchange (NDEx) repository | https://home.ndexbio.org/about-ndex/ |
| 156 | Nextprot | https://www.nextprot.org/ |
| 157 | *NMRshiftDB** | http://nmrshiftdb.nmr.uni-koeln.de |
| 158 | *Ocean Biodeiversity Information System (OBIS)** | https://obis.org |
| 159 | OceanRep (GEOMAR) | https://oceanrep.geomar.de/ |
| 160 | Online Mendelian Inheritance in Man (OMIM) | https://www.omim.org |
| 161 | openBIS LIMS-ELN | https://csb.ethz.ch/tools/software/openbis-lims-eln.html |
| 162 | **PANGAEA. Data Publisher for Earth & Environmental Science*** | https://www.pangaea.de |
| 163 | Panorama Public | https://panoramaweb.org/project/Panorama%20Public/begin.view? |
| 164 | PASS & PharmaExpert | http://genexplain.com/pass/ |
| 165 | Pathogen Host Interactions "PHI-base" | http://www.phi-base.org |
| 166 | PeptideAtlas | http://www.peptideatlas.org/ |
| 167 | PharmaExpert | http://genexplain.com/pharmaexpert/ |
| 168 | PharmGKB | https://www.pharmgkb.org |
| 169 | *Planktonnet** | https://planktonnet.awi.de/#content |
| 170 | **Plant Genomics and Phenomics Research Data Repository*** | https://edal-pgp.ipk-gatersleben.de/whatis.html |
| 171 | Plant Metabolic Networks | https://www.plantcyc.org/feedback/data-submission |
| 172 | PomBase | https://www.pombase.org |
| 173 | Portal of Medical Data Models | https://medical-data-models.org/ |
| 174 | PRODORIC | http://www.prodoric.de/index.php?index=1 |
| 175 | *Protein Data Bank in Europe (PDBe by EBI)** | <http://www.ebi.ac.uk/pdbe/> |
| 176 | Protein Families (Pfam by EBI) | https://pfam.xfam.org/ |
| 177 | Protein-chemical Interactions | http://pcidb.russelllab.org |
| 178 | Proteomic platform | https://gepris.dfg.de/gepris/projekt/280720994 |
| 179 | *PRoteomics IDEntifications Database (PRIDE by EBI)** | http://www.ebi.ac.uk/pride |
| 180 | Prototypic small molecule database for bronchoalveolar lavage-based metabolomics | https://static-content.springer.com/esm/art%3A10.1038%2Fsdata.2018.60/MediaObjects/41597_2018_BFsdata201860_MOESM75_ESM.zip |
| 181 | Pseudobase | http://www.ekevanbatenburg.nl/PKBASE/PKBABOUT.HTML |
| 182 | Pubchem | https://pubchem.ncbi.nlm.nih.gov |
| 183 | QM-sym, a symmetrized quantum chemistry database of 135 kilo molecules | https://www.nature.com/articles/s41597-019-0237-9 |
| 184 | RADAR | https://ub.fau.de/forschen/daten-software-forschung/radar-datenplattform/ |
| 185 | RAMEDIS Rare Metabolic Diseases Database | https://agbi.techfak.uni-bielefeld.de/ramedis/htdocs/eng/index.php |
| 186 | *Reactome ** | https://reactome.org |
| 187 | Reference Sequence Database "RefSeq" (by NCBI) | https://www.ncbi.nlm.nih.gov/refseq/ |
| 188 | Repository Search / Repositorien Finder | https://v2.sherpa.ac.uk/cgi/search/repository/basic?screen=Search&repository_name-auto_merge=ANY&repository_name-auto=data&order=preferred_name&_action_search=Search |
| 189 | Research Collaboratory for Structural Bioinformatics PDB (RCSB PBD) | https://www.rcsb.org/pages/about-us/index |
| 190 | Rfam | https://rfam.xfam.org/ |
| 191 | *Rhea** | https://www.rhea-db.org |
| 192 | RIKEN MetaDatabase for healthcare and life sciences | https://ideas.repec.org/a/igg/jswis0/v14y2018i1p140-164.html |
| 193 | **Sabio RK*** | http://sabio.h-its.org/layouts/content/about.gsp |
| 194 | SATPdb: a database of structurally annotated therapeutic peptides | https://academic.oup.com/nar/article/44/D1/D1119/2502618 |
| 195 | **SCAR Antarctic Biodiversity Portal*** | https://www.biodiversity.aq/ |
| 196 | Science Europe AISBL | https://scienceeurope.org/our-priorities/research-data/ |
| 197 | Sciences Linked Open Data Cloud | https://www.nature.com/articles/s41746-019-0162-5#article-info |
| 198 | Sequence Read Archive (by DDBJ) | https://www.ddbj.nig.ac.jp/index-e.html |
| 199 | Serum Metabolome | http://www.serummetabolome.ca |
| 200 | Share it - Open Access & Forschungsdaten-Repositorium der Hochschulbibliotheken in Sachsen-Anhalt | https://opendata.uni-halle.de |
| 201 | Signaling Pathways Project, an integrated ‘omics knowledgebase for mammalian cellular signaling pathways | https://www.nature.com/articles/s41597-019-0193-4 |
| 202 | **Small Angle Scattering Biological Data Bank*** | https://clarivate.com/webofsciencegroup/master-data-repository-list/ |
| 203 | STITCH | http://stitch.embl.de/cgi/about.pl?UserId=EoDiaj1mTpGv&sessionId=d2x0a6gsdIdS&footer_active_subpage=content |
| 204 | *StoreDB** | https://www.storedb.org/store_v3/ |
| 205 | **Strenda*** | https://www.beilstein-strenda-db.org/strenda/ |
| 206 | STRING | https://www.re3data.org/search?query=&countries%5B%5D=DEU&providerTypes%5B%5D=dataProvider&types%5B%5D=disciplinary&subjects%5B%5D=2%20Life%20Sciences |
| 207 | StudyForrest | http://studyforrest.org/about.html |
| 208 | Subnational Human Development Database | https://globaldatalab.org/shdi/shdi/ |
| 209 | SuperDRUG2 | http://cheminfo.charite.de/superdrug2/ |
| 210 | Technologie- und Methodenplattform für die vernetzte medizinische Forschung e.V (tmf) | https://www.forschungsdaten.info/wissenschaftsbereiche/lebenswissenschaften/medizinische-verbundforschung/ |
| 211 | Technologisches Innovationssystem - Infrastruktur Provider | https://www.biocase.org/whats_biocase/index.shtml https://www.bolgermany.de/wp/startseite/links/ |
| 212 | Transfac | http://genexplain.com/transfac/ |
| 213 | Translational Skin Disease Platform (Z04) | https://gepris.dfg.de/gepris/projekt/340075938 |
| 214 | Transpath | http://genexplain.com/transpath/ |
| 215 | *TRY (Plant Trait Database)** | https://www.try-db.org/TryWeb/Home.php |
| 216 | U-Index, a dataset and an impact metric for informatics tools and databases | https://www.nature.com/articles/sdata201843 |
| 217 | **Uniprot*** | https://www.uniprot.org |
| 218 | Viertuelles Herbarium Deutschland | http://vh.gbif.de/vh/ |
| 219 | wikidata | http://www.chemspider.com/DatasourceDetails.aspx?id=923 |
| 220 | *Worldwide Protein Data Bank (wwPDB)** | https://www.wwpdb.org/ |
| 221 | WORMS world register for marine species | http://www.marinespecies.org/subregisters.php |
| 222 | **Zenodo*** | https://zenodo.org |
| 223 | Zentrale Biomaterialbank der Charité | https://biobank.charite.de/projekte_partner/ |

*data platforms (*contacted in italic* and **interviewed in** **bold**)
