# Supplementary material for: Data platforms for open life sciences–A systematic analysis of management instruments
Source: PLoS One. 2022 Oct 25;17(10):e0276204. doi: 10.1371/journal.pone.0276204 (PMC9595524; doi:10.1371/journal.pone.0276204)
Supplement: S2 Table — (DOCX) [file pone.0276204.s002.docx]

# S2. Table. Overview of collected characteristics and values

| **Characteristic** | **Characteristic value 1** | **Characteristic value 2** | **Characteristic value 3** | **Characteristic value 4** |
| --- | --- | --- | --- | --- |
| **Platform type** | Making own work available as database | domain specific platform | non domain specific data platform | data aggreagator |
| **Access control** | yes | no |  |  |
| **History platform implementation** | single person | single research group | collaboration/research alliance/cohort | independent data platform |
|  | commercial | community action | institutional platform | unknown |
| **Submission through external parties** | no | yes | maybe | unknown |
| **Extension partner network** | yes | no | unknown |  |
| **Funding** | Project based | long term | unknown | diverse |
| **Sponsors** | Research projects | Industry | Public institutions | Foundations |
|  | State/ministry | unknown |  |  |
| **Data type** | Experimental | Simulation | Extraction from literature | patents |
|  | unknown |  |  |  |
| **Participating countries** | German wide | international | national (other) | unknown |
| **Headquarter** | Germany | other | unknown |  |
| **Website embedded in** | institute | independent | other |  |
| **Restrictions** | Privacy policy | commercial | licence (CC) | diverse |
|  | no |  |  |  |
| **Governance** | whole platform | individual data set | unknown |  |
| **Ownership** | Private sector | Public sector | Non-governmental Organisation | Platform cooperatism |
| **Curation** | manually | automatic | no |  |
| **Certifications** | e.g. Core Trust Seal |  |  |  |
