# Supplementary material for: Data platforms for open life sciences–A systematic analysis of management instruments
Source: PLoS One. 2022 Oct 25;17(10):e0276204. doi: 10.1371/journal.pone.0276204 (PMC9595524; doi:10.1371/journal.pone.0276204)
Supplement: S3 Table — (DOCX) [file pone.0276204.s003.docx]

# S3. Table. Additional information on interviews

| **Interview ID** | **Series** | **When?** | **Position** | **Institute / Company Type**  **or Platform Category** | **Duration [min]** |
| --- | --- | --- | --- | --- | --- |
| 1 | 1 | Q3/2019 | Senior Project Manager | Chemical Company | 60 |
| 2 | 1 | Q4/2019 | Lab Manager | Consumer Goods Company | 45 |
| 3 | 1 | Q4/2019 | Head of Life Science | Pharmaceutical Chemical Company | 46 |
| 4 | 1 | Q1/2020 | Politician | State Parliament | 35 |
| 5 | 1 | Q4/2019 | Scientist | Research Institute | 44 |
| 6 | 1 | Q4/2019 | Chief Technology Officer | University | 53 |
| 7 | 1 | Q1/2020 | Director of an institute | University | 15 |
| 8 | 1 | Q1/2020 | Director of an institute | University | 24 |
| 9 | 1 | Q4/2019 | Executive board member | University | 53 |
| 10 | 1 | Q4/2019 | Head of Research Data Management | University | 63 |
| 11 | 1 | Q4/2019 | Professor | University | 36 |
| 12 | 1 | Q4/2019 | Professor | University | 36 |
| 13 | 1 | Q4/2019 | Scientific Director | University | 37 |
| 14 | 2 | Q1/2020 | Coordinator | Backbone Platform (1) | 79 |
| 15 | 2 | Q1/2020 | Manager | Enabler Platform (1) | 52 |
| 16 | 2 | Q1/2020 | Responsible | Enabler Platform (2) | 78 |
| 17 | 2 | Q1/2020 | Head of Strategic Partnerships | Enabler Platform (3) | 65 |
| 18 | 2 | Q2/2020 | Chief Execution Officer | Enabler Platform (4) | 15 |
| 19 | 2 | Q1/2020 | Board member | Backbone Platform (2) | 53 |
| 20 | 2 | Q1/2020 | Board member | Backbone Platform (3) | 112 |
| 21 | 2 | Q1/2020 | Director Product Development | Generalist Platform (1) | 64 |
| 22 | 2 | Q1/2020 | Executive Director | Generalist Platform (2) | 58 |
| 23 | 2 | Q1/2020 | Marketing Director | Backbone Platform (4) | 65 |
| 24 | 2 | Q1/2020 | President | Backbone Platform (5) | 45 |
| 25 | 2 | Q1/2020 | Steering Committee member | Generalist Platform (3) | 42 |
| 26 | 2 | Q1/2020 | Head of Content and Service | Enabler Platform (5) | 56 |
| 27 | 2 | Q1/2020 | Responsible | Backbone Platform (6) | 77 |
| 28 | 2 | Q1/2020 | Manager | Enabler Platform (6) | 114 |
| 29 | 2 | Q1/2020 | Responsible | Backbone Platform (7) | 43 |
| 30 | 2 | Q1/2020 | Curator | Enabler Platform (7) | 90 |
| 31 | 2 | Q1/2020 | Manager | Generalist Platform (4) | 96 |
| 32 | 2 | Q1/2020 | Head of Platform | Generalist Platform (5) | 114 |
| 33 | 2 | Q1/2020 | Project Manager | Backbone Platform (8) | 60 |
| 34 | 2 | Q1/2020 | Project Manager | Backbone Platform (9) | 72 |
| 35 | 2 | Q1/2020 | Curator | Backbone Platform (10) | 98 |
| 36 | 2 | Q1/2020 | Responsible | Backbone Platform (11) | 62 |
| 37 | 2 | Q1/2020 | Scientific Coordinator | Enabler Platform (8) | 74 |
| 38 | 2 | Q1/2020 | Principal Investigator PI | Enabler Platform (9) | 71 |
| 39 | 2 | Q1/2020 | Service Manager | Generalist Platform (6) | 75 |
|  |  |  |  | **Average duration [Min]** | **61** |
|  |  |  |  | **Sum [Min]** | **2333** |
