# Supplementary material for: Data platforms for open life sciences–A systematic analysis of management instruments
Source: PLoS One. 2022 Oct 25;17(10):e0276204. doi: 10.1371/journal.pone.0276204 (PMC9595524; doi:10.1371/journal.pone.0276204)
Supplement: S4 Table — (DOCX) [file pone.0276204.s004.docx]

# S7. Table. Definition table of applied terms and concepts

| **#** | **Terms / Concepts** | **Definition** |
| --- | --- | --- |
| 1 | History | (Subjective and objective) events related to the establishment of the platform. An event is something that is characterized by change and dynamics. It is primarily about the history of how the platform was built and not how it has developed since its implementation. |
| 2 | Core Offering | Objective offers and functionalities, for which the platform was mainly designed. No offers in the future, intended or planned. |
| 3 | Success Factor | Success factors are concrete factors (fields of activity, tasks, actions, etc.) that are necessary for the platform to operate successfully in the life sciences. |
| 4 | Vision | The vision is about future goals, conditions and further indications, what the company wants to become by setting a defined direction for the company’s growth. These are neither short-term goals nor goals that have been achieved. |
| 5 | Organisational structure | An organisational structure is a system that outlines how certain activities are directed to achieve the goals of an organisation [...]. The structure indicates required work activities of the organisation, reporting relationships as well as departmental groupings (Daft et al., 2010). |
| 6 | Operational structure | The operational structure concerns the execution of the activities of the organisation, i.e. the work processes required to fulfil the tasks, thereby considering spatial and temporal aspects (Hub, 1994). It includes how employees carry out their tasks, what competencies they have and what the educational background is. |
| 7 | Responsibilities | Distribution of responsibility, which can be certain tasks, budgets. How the commitment behind it is accepted and how accountability is taken. |
| 8 | Decision | Factors and ways in which decisions are made and the corresponding communication channels/dependencies |
| 9 | Strategy | On the one hand, the presentation of strengths that clearly distinguish a product from other products, and on the other hand, the (usually long-term) planned behaviour of companies to achieve their goals. |
| 10 | Funding / Financing | Financing is all aspects that have an impact on the monetary situation and its persistence. |
| 11 | Goal and Goal Control | Objectives are statements about desired conditions in the future, which are to be achieved by appropriate behaviour, implementation and achievement. Goal control is all mechanisms that track the achievement of goals and make it controllable. |
| 12 | Difficulties Managing Platform | Difficulties in transforming an existing actual state into a desired target state. During the transfer, a barrier must be overcome. |
| 13 | Functionality | Feasible actions on the platforms are considered as a possibility. Functionalities are the set of functions with defined properties available on the platform |
| 14 | User group | The term user group refers to all different users, their educational and scientific background and other characteristic features such as country of origin and institute or company affiliation. |
| 15 | Difficulties Data Exchange | Data exchange refers to the entire process in which a user uploads data, searches for data on the platform and uses or recycles it, as well as all possible sub-steps in between. Difficulty has already been defined. |
| 16 | Reusability of Data | Reusability refers to the fact that a data set is not only used within an analysis but several times, also by different actors. |
| 17 | Rules and Governance | Rules are defined guidelines which users have to follow. This includes moving around on the platform as well as using the data and functionalities provided on the platform. |
| 18 | Requirments Data | A requirement is a statement of what prerequisites a user must meet in order for data to be uploaded and made available on the platform. Fulfilment is obligatory and not voluntary. |
| 19 | Trust | “The definition of trust [...] is the willingness of a party to be vulnerable to the actions of another party based on the expectation that the other will perform a particular action important to the trustor, irrespective of the ability to monitor or control that other party” Mayer et al. (1995). With regard to platforms, Botsman (2017) has developed a three-step process which he calls “Trust Stack”. First, a researcher trusts that the idea of sharing data is safe and worth trying. Then, trust must be established in the data platform. The final step is to trust the exchange partner that the data meets the appropriate criteria and appears useful. |
| 20 | Scientificly correct usage | Scientific correctness means the handling of scientific data, articles and other things according to good scientific practice. In particular, it deals with misrepresentation, the violation of intellectual property and problems of authorship and consent in scientific publications. |
| 21 | Data Quality | The quality of the data refers to the sum and/or quality of (all) characteristics of a data. Quality indicates the extent to which data comply with existing requirements, defined by users or the scientific community (Deutsches Institut für Normung, 2015). The quality of the data is often subjective and self-defined. Some prefer to call quality “fit for purpose“. |
| 22 | Incentives use | Incentives are behaviour-influencing stimuli that lie inside or outside a person. They can induce a person to behave in a certain way. |
| 23 | Scientific culture | Culture is an umbrella term which encompasses the social behavior and norms found in human societies, as well as the knowledge, beliefs, arts, laws, customs, capabilities and habits of the individuals in these groups (Tylor, 2016). |
| 24 | Development 10 years | Development is generally understood to be a process of emergence, change or decay (Stangl, 2020). |
| 25 | Success Dependency | Dependence is the relationship of reliance of one thing on another in any way. In this case, the reliance of success on another matter, circumstance or condition. |
