# Supplementary material for: Data platforms for open life sciences–A systematic analysis of management instruments
Source: PLoS One. 2022 Oct 25;17(10):e0276204. doi: 10.1371/journal.pone.0276204 (PMC9595524; doi:10.1371/journal.pone.0276204)
Supplement: S1 File — (DOCX) [file pone.0276204.s005.docx]

# S4. File. Interview transcription guideline

Content semantic transcript derived and adapted from Mayring^1^

- Transcribe completely and literally (keeping incompleteness and repetitions)
- Word blends are approximated to written German/English (example: “So’n Buch” becomes “So ein Buch”)
- The sentence form is retained, even if it contains syntactic errors
- Language and punctuation are slightly smoothed, i.e. approximated to written German/English
- The content is in the foreground, expressions like “uh” are ignored and dialectal is translated into German/English
- Agreeing or confirming utterances of the interviewer like “mhm” or “aha” are not transcribed as long as they do not interrupt the flow of speech of the interviewee
- Unintelligible words are marked with “unv.” and longer incomprehensible passages will be marked as “unv., microphone murmurs”. Assumptions by the interviewer are put in brackets with a question mark, e.g. “(axe?)” and unintelligible passages are marked with a time stamp.
- Dashes (-) are used for breaks and stagnation. For longer pauses, several dashes (–) are used accordingly.
- Indicated noticeable features as well as all non-verbal characteristics that are important for understanding the content are added in brackets like “(laughter)”
- Incomplete sentences are marked with the abort character “/”.

1. Mayring, P. *Qualitative Inhaltsanalyse* (Springer Fachmedien Wiesbaden, 2020).
