# Supplementary material for: Data platforms for open life sciences–A systematic analysis of management instruments
Source: PLoS One. 2022 Oct 25;17(10):e0276204. doi: 10.1371/journal.pone.0276204 (PMC9595524; doi:10.1371/journal.pone.0276204)
Supplement: S2 File — (DOCX) [file pone.0276204.s006.docx]

# S5. File. Interview guideline

The main part of the interview guideline has been modified within the course of the interviews. Series 1 and series 2 differ as the interviewees either answered main part I or main part II.

**Introduction**

- Introduction of the topic and the interviewer including confidential agreement of information and recording
- Description of the interview procedure and approximate duration
- Information on the interviewee: name, highest degree, professional title, age, team size, organization, organization affiliation since, research area, engagement in national or international research cooperation

**Main Part I**

1. Entry questions

- What are your daily tasks at _______?
- In which data sharing activities are you involved?

1. Key questions “Demand”

- To what extent do you use shared data from others in your research? If so, which and why?
- Which shared data would you like to access (additionally)?
- What requirements do you have for the shared data?
- How do you currently access the shared data?
- How do you imagine (in the future) the ideal access to required data?
- What problems and difficulties arise when using shared data?

1. Key questions “Offer”

- What information do you share with others and about what?
- What are your motives for sharing data with others?
- To what extent will or do you want to expand these data sharing activities?
- There may be many reasons why data are not exchanged. Under what circumstances or when do you not share data with others?
- What problems and barriers do they see in data sharing?

1. Supplementing questions / themes

- Financial aspects? To what extent do financial aspects influence data sharing?
- Legal aspects? To what extent do legal aspects influence data sharing?
- Corporate culture? Which culture is present in your company (process-, result-, or employee-oriented and to what extent does the culture in the company affect the activities?
- Management? What influence does management have on data sharing?
- Personal and team factors?
- Time exposure? How time consuming is it for you to use the system or learn how to use it?
- Technical aspects? To what extent do technical aspects influence data sharing?
- Recognition? To what extent is data sharing recognised? Whether in a team or in the scientific community?
- Which problems and barriers do you think have the greatest impact on data
- sharing?
- Do you have any other aspects that come to your mind on the subject of shared data or sharing data? If so, which ones?
- With increasing technological progress, more and more data volumes are needed for evaluation. How do you assess the influence of Big Data or artificial intelligence on your everyday research?

**Main Part II**

1. Entry questions

- Before we start the interview, I would like to know something about you. Could you please tell me briefly about your background and what you do?
- What is the history behind the platform? How was the platform actually created or implemented? Which parties were and are involved?
- What is the core offering of the platform?
- How do you general define success or a successful platform?
- What vision is the platform pursuing?

1. Organisational, Operational Structure and Governance

- What is the organizational structure behind the platform?
- How many employees with which activities and competences work for the platform?
- How are different responsibilities for the platform distributed?
- How are decisions made and what are the decision paths?
- How do you strategically position the platform in relation to competitors?
- How is the platform financed?
- What are specific goals of the platform and how do you control the goals you set?
- What are the difficulties/challenges in managing the platform?

1. Platform governance and use

- What functionalities and possibilities does the platform offer?
- How do you describe the user group and/or participating institutes as well as possible third-party funders of the platform?
- What kind of data is exchanged and what difficulties arise in exchanging data via the platform?
- If suitable: How do you estimate the reusability of the data?
- What requirements do you have for the submission of data?
- If general terms and conditions not found on website: What rules apply to the users of the platform?
- How do you ensure that people upload data to the platform in sufficient number and quality?
- How is trust in the data ensured?
- How do you ensure that the users of the platform deal scientifically/ethically with the use of data?
- How do you encourage researchers and/or organisations to actively use the platform?
- What other ways could be considered to generate users, attract organisations or increase activity on the platform?
- How does the scientific culture influence the use of the platform?

1. Final questions

- What development do you see for the platform over the next ten years?
- What does the success of the platform depend on?
- Is there anything else you want to tell me about factors of platforms?

1. Questions on platform statistics

- Is the software behind the platform a proprietary development or is it purchased?
- Is the infrastructure of the platform itself operated or not?
- How long has the platform been in existence (year of foundation?)
- How many current users does the platform have approximately?
- Number of visits: The number of visits to the website
- Number of page views: The number of page views of a user per visit

Number of employees for the platform

**Closing**
