# Supplementary material for: Data platforms for open life sciences–A systematic analysis of management instruments
Source: PLoS One. 2022 Oct 25;17(10):e0276204. doi: 10.1371/journal.pone.0276204 (PMC9595524; doi:10.1371/journal.pone.0276204)
Supplement: S3 File — (PDF) [file pone.0276204.s007.pdf]

Supporting Information S6. File. Interview transcripts (anonymized)

Interview	Time	Interviewee	Interviewer
Interview 1	Time	Interviewee 1	Interviewer
Interview 1	0:00		(...) So I just introduced myself. And before we start the interview, I would like to know something about you, your background and what you're doing.
Interview 1	0:11	(personal)	
Interview 1	0:56		And what is the history behind (platform)?
Interview 1		(anonymized)	
Interview 1	4:06		And what is the core offering of (platform)?
Interview 1	4:10	So the core offering is basically a place for researchers to upload their data and make it publicly available.	
Interview 1	4:19		My work now is about success and success factors. So how do you define success or a successful platform?
Interview 1		I think user adoption is very important. There's no point having a platform like (platform), if researches on on using it. That's both in a sense of creating an account and uploading their data.	
Interview 1		But then also people going to the platform and just browsing and then finding relevant information. If you need a, like a close parallel, if you think about like, more, like kinda like a YouTube or image, you're one of those sites but specifically for academic data.	
Interview 1	4:30	So there are obviously lots of content creators out there who are uploading content to YouTube, which is and that's an important success factor. But then also people coming along and viewing and downloading and interacting with the content is also extremely important.	
Interview 1	5:25		So what vision are you pursuing with the platform?
Interview 1		so we would like all academic outputs that are publicly funded to be openly available. So this comes with a few caveats that you know, there if you're thinking about medical data, patient data, that can be highly sensitive.	
Interview 1	5:29	So not all, not all data can be made openly available. But the bulk of research, you know, if you, you, being a chemist yourself, if the experiments that you run, you know, there's nothing highly sensitive in the results that you generate. So that should be shared with other chemists. So people can then take it and build upon it.	
Interview 1	6:11		So coming now to a more organizational focuses question part. what is the organizational structure behind (platform)?
Interview 1		So we have quite a unique structure in the sense that, so, (Founder) created the platform himself. And then after some time he was approached by a company (anonymized) . And (the company) is an umbrella company that invests in lots of different scientific startups and software companies with the view to accelerate research and get all these innovative companies together in the same space and integrating products and Building upon each other's work.	

Interview 1		So we have a core (platform) team. And that's mostly developers. And then we also have some business support some marketing people, some sales people within (platform). But then we have (mother company), which is an umbrella company, and an incubator, which we have shared resources with them.	
Interview 1		So for example, we all share an office together in (town) for the companies that have (town) based staff. We have a shared legal team, (unv) shared HR team, shared IT department.	
Interview 1	6:24	So there's a layer of (mother company), which I guess is like business support and Business Administration. And then there's the core product team and (platform), which are the developers, the marketers and the sales people who and the business support who only work on feature	
Interview 1	7:59		so how How many people together work for (platform)?
Interview 1	8:03	So (platform) in total, we're about 40. But then (mother company), they're probably about another 50 or so support staff. But then the entire if you were to take all the other feature type companies who have also been invested in this probably close to three or 400, worldwide worldwide now, but we only really interact with the (mother company) team. We don't interact so much with or share resources with the wider 3-, 400 people.	
Interview 1	8:40		And what kind of competences do those people have?
Interview 1		So there's software developers, and the people the core coders who build the products. There's the designers, the people who actually design the product. Do the user experience. Then there's marketers, salespeople, business admin, and customer support. Going around the company.	
Interview 1	8:46	And then yeah, some like product of product managers and Scrum masters and things like that. That's within the (platform) team. And then the higher up level, I'd say it's the more executive support and very specialized support, like HR or legal, things like that things that we don't necessarily need to use every day. But it's good to have that resource.	
Interview 1	9:49		You explain now that you have this core team, and how are the different responsibilities distributed for the platform within one team or dedicated to people? How are the responsibilities distributed?
Interview 1		Yeah, so there's the, I'd say the the most structured team is the development team because there's, there's the most of them. And then development teams need to work in quite an organized way. So there's a product director, who is in charge of the core delivery of the product.	
Interview 1		underneath him is like a scrum master type person who makes sure you know, all the all the tasks are actually divided out. And then underneath him is the different teams with all the different developers who would actually do the work and build the product and that is loosely developed divided off into design, front end development.	

Interview 1	10:05	middleware and back end. And then within those teams as well, they'll be like a team leader, and then people that work underneath them. The rest of the business is quite flat structure, where there's just the specialist and each area. So I'm the marketing person, I do all the marketing. There's a sales support person, they do all the sales support engagement person. They've, yeah, we're a little bit more flat on the the business support side of things.	
Interview 1	11:31		And when it comes to decisions, how are they made? And what are the decisions path?
Interview 1	11:38	Yeah, so it can be quite messy. We're because we're such a small company, and certain things. So there's a leadership team, which is a product director, marketing director, the CEO and the business developer Director. those, the four of us make, I guess the big strategic decisions around what areas of the product are we going to focus on if we're going to work in any new territories or build any new products? Any hiring, like big hiring strategic position decisions happen, I guess, between the four of us, and then that then cascades down to the rest of the team.	
Interview 1	12:31		You just mentioned the strategy. So how do you want to strategically position (platform)?
Interview 1		so I guess there's there's something to explain as well as that we do work across some quite different markets. So we have an end user product. I've used the YouTube example before where people can come along, sign up, upload, and it's completely free. There. We have a product for universities, so universities like our product, but they'd like to customize it and do certain bits and pieces with it.	
Interview 1	12:43	So we have a product that works with universities. We also work with publishers, we work with governments and funders. And we work with a few organizations as well. So from a strategic point of view, we work yeah, across quite a few different markets. And they they will vary quite a lot in the way that we approach them. Not sure if you have any more specific questions.	
Interview 1	13:43		So I'm wondering if you want to broaden the community size, or if you want to dive into a more intensive user within the existing community. So how would we go about that? I think details on that question.
Interview 1	14:12		I mean, this was more or less my question that if you want to broaden the community, or if you want to have a more intense usage,
Interview 1		right. And so there's, like I said, there's lots of different organizations that we work with. So by us signing up a university, they will then take the platform and then give it to all of their researchers, for them to use. So that's probably the main area that we broaden it as we partner with organizations on a b2b level, and then that then gets more usage because they give it to all of their users to use.	

Interview 1	14:22	Much like Like if you know you work for a company, and they have Microsoft Office and they they just they buy the license for everyone, then you as an employee or researcher, you just get access to that platform. And it just it's part of what the school provides too. So it's that's our main area of growth is by partnering at a b2b level, which then trickles down to the users.	
Interview 1	15:28		Okay, so for my understanding, so you provide the (platform) software to a company and they can set up their own (platform) layout within their clothes company.
Interview 1		No, not quite. It's more. So there's a growing need for particularly publishers and institutions to make their data available because the people who fought Research, starting to mandate this and say if you take money from us, then you have to make your data publicly available. So we are software as a service.	
Interview 1		So we we can, we're kind of the alternatives of what you provide what you just described, there are companies that do that and say, we will give you the software and then you can run it locally and do all the administration and do all the customization way or more the the other other side of it, where universities will come to us and say, we don't want to have to hire 20 developers and build and maintain this thing ourselves.	
Interview 1	15:46	So we will just buy a license of you. And then we do all the hosting and all the updates and all the support. And so that's it's one less thing that universities have to worry about.	
Interview 1	16:52		And the data within (platform) is public available?
Interview 1	16:57	Yes, by and large. There are few is where you can keep it private, if you want to, but all the data is publicly available.	
Interview 1	17:05		Okay, that is good to understand. And you mentioned that you got the (mother company) grant in the beginning, and how is the platform financed or funded now?
Interview 1	17:20	So all of the b2b customers that I told you about before, they all pay an annual license fee. So that's anywhere between (anonymized) pounds and some of the bigger ones pay paid more than that, cuz Yeah, they get more customized work. And then and we have over 100 customers now. So that's, that's enough to keep us sustainable.	
Interview 1	17:51		What specific goals you want to achieve?
Interview 1		So I think we We haven't really deviated from our original mission of why (platform) was set up. And that was to make all the publicly funded research publicly available.	
Interview 1	17:58	So that's the, by and large, one of the, the biggest driving forces, but then also, to help researchers get credit for all of the extra work that they do that falls outside of the, the academic credit system that people can be subscribed to. So it's twofold is to make the data openly available, but then also for the researchers to actually get credit for all of the work that they do.	

Interview 1	18:42		And how do you control the goals you set?
Interview 1	18:46	Luckily, we've we've had a very strong mission from the start, and we try to stick to it. So it's kind of the same mission from day one. To what to what we're doing now. It's obviously a lot more details. We, we deal with a lot more people. We have a lot more state stakeholders, but we try to keep the the main vision the same. And then the goals that that lead up to that. I guess again, we keep them there. They're quite easy to manage, I guess. Yeah.	
Interview 1	19:23		And what are the difficulties and challenges in managing (platform)?
Interview 1		I think, well, when we, when we first started, we were the first one will one of the first ones out there, certainly the most popular. And in recent years, we've had quite a lot of competition come along, which is ultimately a good thing. But we do now have to prioritize what work we do.	
Interview 1		And I think that's the hardest Is with a relatively small company, but we work with, you know, huge organizations all over the world. You have to balance what is a priority for your customer? And what is the priority against the (platform) mission, because you could end up doing all sorts of customized work, it only works in a very specific example, because you're one of your customers wants it.	
Interview 1	19:32	So I think building a platform that still works for the researchers and works across, you know, 100 organizations, and keeping keeping like, strict on that I think is the hottest thing.	
Interview 1	20:41		And do you have other or more difficulties?
Interview 1		I think the prioritization of the development workflow, I think that's probably the hardest one, I think going into new territories is always tricky as well, because every country has their own laws around day like data storage, and certain things like that. And then each fund in each area as well has different rules around when and how the data should be made an openly available.	
Interview 1	20:49	So because we work in (country), (country), (region), (region), right the way across (region), a bit in Asia as well. There are vastly differing requirements, I guess, at the governance level. And that is sometimes you get policy clash. So you need to try and work your way through that.	
Interview 1	21:50		Great. That means that we have finished the organizational block. coming to the platform governance And functionalities. You mentioned now you do a lot of customized development and so on. So what functionalities and possibilities and do you offer with (platform).
Interview 1	22:16	So there's, there's the core platform, which is (platform) calm and now has almost no customization. So if you take one of the free accounts, then that is you know, you get it out of the box and use it as it is, again, the YouTube example. It's just there to use and you you can't do much customization outside the fields that are provided to at an organizational level.	

Interview 1		We we can do some integration so if you have other systems within your so this is one in the paid for offering, if there's other systems that you want us to integrate with within your university and we can do That if you need us to integrate with the like local storage or something like that, we can do that. And then there's also some customization.	
Interview 1		Just there's still quite limited around the platform that you can, you know, manage your own users, you can manage storage quotas, you can customize some of the metadata. And so that that's a little bit more flexible. But then we also offer, we have an open API, which people have used to then build completely custom platforms.	
Interview 1		So (platform) then becomes kind of the nuts and bolts in the background. And then, but you then have your own developers who might want to like build something very specific. So we've seen some cool examples of that of people just using their the call. (platform) platforms, upload the data and manage them.	
Interview 1	22:44	We do lots of kind of the box ticking on that side of things. And then they build data visualization tools on top or a completely custom front end. And so we Yeah, we can be quite flexible, but there's only so much that we offer as a, as a company that gets to be there comes to a breaking point where we say, if you want to do that, then you need to hire another company or hire people to do that within within your organization.	
Interview 1	24:35		How do you describe the user groups and participating institutions?
Interview 1	24:41	The I don't quite understand the questions or?	
Interview 1	24:45		how do you describe the user group and participating institutions?
Interview 1		The user group so Okay, yeah, I think I understand. So I think you can break it up into probably three layers that The end users who are the researchers who upload their data and use (platform) on a daily basis. There's the curators, so they would be people who sit between the researcher and actually making things publicly available.	
Interview 1	24:50	So they kind of Yeah, they're like to someone that's a help that were the researcher and they would, they would be there. They wouldn't necessarily be the product owner at the institution, but they would be kind of like a layer up from the researchers, the end users, and then we also have the administrators, so they would have the power to to make much higher level changes	
Interview 1	25:49		I have one question to the creators. are those creators are working for (platform) or for an institute?
Interview 1	25:58	for the institute.	
Interview 1	26:01		Okay, good. That's good to know. So you don't have to pay them there. They do it by themselves.

Interview 1	26:09	Yeah, exactly. So generally, whenever say there'd be someone within the institution who manages the the product. And then there might be some subjects with specific expertise, who all they need to do is go in and check the files and make sure that, you know, all the fields are being input correctly. So and then there everyone is employed at the university or the organization.	
Interview 1	26:40		And is (platform) open for all kinds of research data? Or do you have some restrictions.
Interview 1		So the end user product, there's some restrictions just around file size. So there is I can't remember where they're currently file size limit is I think it's five gigabytes, and then so per file, and then you've got a quota of how much you can have in total in your profile. But at the institutional level, those those limits are a lot higher.	
Interview 1	26:50	So because the jet age generally integrates with our own storage, or they're paying us for storage, then they can have much higher limit. So it goes up to five terabytes per file. And but yeah, their their end user platform (platform) , we have some restrictions, just around size purely from a sustainability point of view, because we can't afford to host everyone's big data.	
Interview 1		And then the only other restrictions we have, I guess, is that it has to be academic content. So you can't just you know, upload a bunch of your holiday photos or something like that to figure out has to be related to some some kind of accurate, some kind of academic work. That's quite a broad, I guess criteria.	
Interview 1		So we try to be as flexible as possible. But it's just basically a way for us to to keep the spam off of the platform, I guess you could say. Yeah, I think that's all and then confidential data. So that's something that we don't have that much control over. We do check files before they will, all the files on (platform) get checked.	
Interview 1	27:45	I'd say it's mostly down to the research is to make sure that they're not uploading anything that's sensitive or could identify a patient or something like that. And generally, the research is good enough to not do that.	
Interview 1	28:54		Do you have further requirements for the submission of data?
Interview 1		so there's a minimum amounts of metadata. So we asked for a title, a category, a description, everything needs a license to the which shows how openly available everything is. And then there's a free text field where you can add context, just more contextual metadata. So and those are and we try to encourage people to be as descriptive as possible.	
Interview 1	28:59	So you know, don't just drag and drop a file from your desktop that's like my data 123 because that's not going to be descriptive and no one's going to be able to find that. So let me say that it's a spreadsheet that describes genes for this animal saying, so, which we're that's a little bit harder to legislate for, but we do encourage people to put as much descriptive metadata as possible.	
Interview 1	29:56		What difficulties arise when exchanging data via (platform)?

Interview 1		Um, I think it's pretty easy to be honest. I think, you know, internet speed could be an issue. If you're in a country or like a location where you didn't have fast internet, then if someone was sharing a big fall with you, then you're going to come into problems. But generally, we don't really run into that many problems.	
Interview 1	30:03	I think so (platform) is, I would say they've even (platform) is good for small data is good for medium sized data. But you know, for talking genomic data, which is petabytes, or astronomy data, which is even bigger, then it's it's it doesn't operate as well as that really high end scale. But we generally find that people who are sharing that kind of day said don't try to do it through (platform). They already have their own methods that are work better?	
Interview 1	31:02		How do you estimate the reusability of the data?
Interview 1		We don't. So that would be something around that the curator at a university would do that because we, we, as we, as I said before, like, we don't restrict. This is like disciplines or subjects. We So, you know, we can always say is that it needs to be academic content. There's no way that we could have the expertise within (platform), to know that if that data is reusable or not.	
Interview 1		But that's where I'm not sure if you're familiar with the fair principles, where there's like a wiving movement in the communities, academic communities to make old data fair, which stands for finable accessible, interoperable and reusable and the Fair principles have a number of requirements for repositories.	
Interview 1	31:07	And we make sure that we, we tick all the boxes there, but to to assess on an individual level, each file and (platform) to see if it's reusable. We don't have the capacity to do that there's over 5 million files on (platform) last time I checked so there's too much work for anyone.	
Interview 1	32:29		How do you ensure that people upload data to (platform) in sufficient number and quality?
Interview 1		Again, I would I would put me putting back to the previous answers that we can't we can do is we check we can add a glance you can usually see does this file look academic? Does it look useful in some way? And that's all we can really do. Like you know is this is this spam we get lots of people like Uploading, you know, sales presentations for products a, because yeah, they just want to put it somewhere.	
Interview 1		So we can say, well, that's not an academic piece of work. So we're not going to do that. But if it's just somebody spreadsheet or somebody code, there's no way that we can check each file. This is where having the curators of the universities, they would do that work. But that's only for people who are uploading from the university.	
Interview 1	32:38	The publishers who use us, they would check the files and make sure that the files are related to the paper that they're publishing. But (platform) doesn't do that. So I guess you could say we outsource it to an extent to the to our customers. But to figure it out on platform, we do a very basic check, but that's all	

Interview 1	33:51		I mean, related to the quantity is trust. So how do you ensure trust in the data?
Interview 1		I think it's more around. I guess its peer understanding peer to peer understanding. So there are general workflow for people uploading data to share would be a researcher would upload it, and then they would share it with their colleagues share across social media. I think I guess one of the things that you is a proxy for trust, is we have metrics on the platform.	
Interview 1		So you can see the amount of views and downloads a file have has had, we tracked all metrics. So if it's been talked about on social media, so you see how much attention that's had and click through and see who's who's been saying, What about it? If a news article has a news outlet has written about that data, or included the data you can also see that and then we also have citation counts as well.	
Interview 1		So you can see if it's actually being used within a published academic paper. So as a researcher, you could, if you saw some data on there, and it was uploaded a year ago, and it had only, you know, four views and six downloads or something like that, you could maybe treat it with a bit of skepticism, you can get in touch with the author speak to them about the data. And you would probably have some understanding of does this data look right?	
Interview 1	34:00	Does it look wrong? Is that something weird about it? Whereas on the other side, on the flip side, if you see that this data has been downloaded, you know, 50,000 times and commented, you know, 3000 times across social media, and the BBC and the Guardian has included it in some of the articles, then you can see that it's more trustworthy, perhaps, than the other one. That's not necessarily true. So it's a rough proxy.	
Interview 1	36:03		So I'm pretty lucky that I talked to you as a marketing director. So the next question is perfect for you. How do you encourage researchers and organizations to actively use the platform?
Interview 1		So that's a really good question. And we were quite naive at the start of (platform). So we had. So there's a general, open science open data movement who just wanted to use (platform). But then there's also a lot of people out there who don't want to share their data because they feel like they own the data.	
Interview 1		And it's it that's their IP almost, that's that's how they're going to get there. win the Nobel Prize by generating all this data and keeping it to themselves and writing lots of papers. So there's a lot of work to be done to encourage researchers to share their data. And there's actually quite a lot of studies out there to show that if researchers share their data, then they get more impact for their papers, get more citations.	

Interview 1		So it's kind of I guess, this myth busting for the people who don't want to share it. That that's one way and but then also, what we found at organizations, when we started working with universities, we thought that when a university buys (platform), they would have complete understanding of the space know exactly why they use it, and they have access to the research so they knew how to get it. So kind of a build it and they will come type mentality.	
Interview 1		But what we realized very quickly is that librarians who are the people who generally buy fake ship, they're very busy people. And research data isn't necessarily the highest thing on their priority because they have low things to do. So we actually have, we hired an engagement manager. And it's her job to as soon as so when they implement (platform), they get passed over to the implementation team to make sure they have a very smooth process.	
Interview 1		And it's not a big technical hassle. And then once they go live, then they get positive to the Engagement Manager. And she works with the librarians and the staff on a one on one basis to make sure that they have all the information that they need. So they have PowerPoint presentations, they have leaflets, we can come to their organization and do talks. We can do webinars for them. We run events, so they can meet other people who also engage with the platform. So the engagement strategy is, yeah, is that is something that we we recognized very early that we had to have Hope organizations with Because ultimately, you know, they pay an annual license.	
Interview 1		And if they after a year or two, they looked at it so that no researchers were using the platform, then quite simply they wouldn't renew. So it's in our interest. That's part of the mission. As I said, we want to get as many research outputs openly available. But it's also an interest to keep people engaged in the platform. So when it comes to renewal time, there's there's no difficult questions, difficult conversations.	
Interview 1		So yeah, the ensure a very extensive engagement platform, from Yeah, providing all the materials that they need, so it's less work for them, and they can just, they're the ones who have access to the researches. So, let them do what they're good at, and then we can provide them although everything else that we need.	
Interview 1	36:17	We don't want them to spend their time, designing flyers In building PowerPoint presentations, and then that also gives us an element of control over the way that the platform is being talked about, across a really vast around array of stakeholders	
Interview 1	40:17		Coming to control, I know you touched on a little bit, but I just want to ask it to you anyway. So how do you ensure that the users of the platform deal scientifically with the use of the data?

Interview 1		So this so I guess the, the most technical way of answering this would be the licensing. So everything you upload on (platform) can be given a different license, which dictates how open it is. So you can put it on you can say That you want this data, you can make it openly available. But no commercial companies can use it. And people can look at the data, or they can actually build on top of it.	
Interview 1		You could say, this data is openly available, but if anyone really uses it, they have to give me credit. So credit you somehow in the citations, and then or you could say, do what you want with this data. I didn't care. So and then within that is, there's I guess, like an academic code, which dictates that so if you take someone's work, and reuse it and don't cite them, then that's plagiarism.	
Interview 1		And that's obviously you can't do that in any in anywhere in academia. So taking it and dealing with it scientifically, I think it's our that. Yeah, I don't think there's a there's a real answer for that. But how can we make sure that it's not being misused or people not just taking it all and claiming it all for the for themselves?	
Interview 1	40:35	There's there's a licensing arrangement per data set, that they people who upload, they get to dictate how open or close that is. And then there's the general academic code, which stops people from plagiarizing or you know, if you cite someone's data set, then you would you would find out about if you uploaded some data I reused and cited you, then you would find out about that and say, Well, no, you didn't quite understand my data correctly. So I guess there's the scientific method, which is relatively self correcting as well.	
Interview 1	42:50		Coming back to the point with motivating users, do you see any other ways To generate user so to attract organizations, as you already mentioned,
Interview 1		User generation is the hardest thing when it comes to building a platform, especially if it's something new. And potentially something controversial, which, you know, years ago (platform) was, as I described before that, you know, people didn't necessarily want to share their data at first.	
Interview 1		So I think, I think there's the obvious like viral growth that like, if you search for picture on Twitter, you just see hundreds of thousands of resources, just sharing their data. And then their colleagues and peers coming in and commenting and saying all that's really great. You have people out there who are like, they'll see someone's published a paper, and then they'll get in touch with the author on Twitter and say, can you should put the data for this I'm (platform), and they'll be like, oh, what's (platform).	

Interview 1		So there's a real kind of organic viral growth between the end users. A lot of that is motivated by good scientific practice and wanting to share data and wanting to collaborate with your peers. And then I think the other thing, you know, just relying on end users isn't enough. You need to get those b2b partnerships. So for example, we now partnership, a partner with the NIH, which is the biggest funder of data is the (region)n funder.	
Interview 1		It's the biggest medical funder in the world. And they have they provide (platform) as a solution. So there's a lot of power in partnering with these big organizations because they already fund billions of dollars of research and have access to thousands of perceptions, which we wouldn't necessarily be out of reach or In the early days of feature, we did do a lot of things like you know, paid Google search and paid Facebook advertising and paid Twitter.	
Interview 1	43:06	But you then your cost per user acquisition goes up quite like too much for it to be sustainable. So a bit of a bit of paid advertising in the very early days just to get you out there is is good, but then viral growth between peers, and then partnering with organizations who have a much broader reach, I would say are the most important things.	
Interview 1	45:38		One special aspect about (platform) is that it lies within the scientific community. Yes. So how do you think that the scientific culture influence the use of the platform?
Interview 1	45:52	Yeah, like I was saying before, it's, it's just good scientific practice. So if you think about If there's you know, a few	
Interview 1	46:01		
Interview 1		reasons why science exists and it's, it's to generate new knowledge, but it's also to build on the work of people before you. So if you want to, if you've generated some new knowledge and you want to convince your peers that you've you've made a new discovery, then it should be expected that you share not only, you know, a few images and writes up a paper, you should be able to share your whole data set.	
Interview 1	46:06	So people can then understand it and validate your findings. And then they should then be able to take it and then build upon upon it to generate new knowledge. And then that then, that is the scientific method in a nutshell, because it's just it's more people. Taking the knowledge prior generated prior to them, building on top of it to make new knowledge and then pass it on to the next people to work on it. And that is the scientific method kind of takes care of that.	
Interview 1	47:17		And what development Do you see for (platform) in the next 10 years?

Interview 1		10 years? Good question. So I think, despite our growth, I think data sharing is still in its relative infancy. And I think it might take another 10 or 15 years for it to become really standard practice. And even then there might be pockets of of researchers who will never share their data. So I think there's still the base mission that will keep driving us but from for me from a where I personally will It's ago, I think there should be, the data should be more interactive and it should be	
Interview 1		It should be more data visualizations and make it a lot easier to understand not only for researchers but also for the general public. So if we think about all the problems, you know, with fake news or people misunderstanding climate data, the reason for that is because the climate data that is out there has been shared in quite an unintelligible way.	
Interview 1	47:22	And you need to be a scientist with some data analytic skills to really understand what the impact could be. So I think data being presented in a much more simple way, communicating really complex ideas, but in simple, simple methods. That's, I think, the most important space that we could be concentrating on.	
Interview 1	48:55		And what does the success of the platform depends on then?
Interview 1		I think reuse is is one of the things that we see a lot grows also, we measure the citation count. So how data is cited in, in other papers. So hopefully at the moment, it's, you know, the, the reuse in an academic, formal sizable way by that is still in its infancy. But we are seeing a year on year growth, it's growing exponentially.	
Interview 1	49:00	So from for me, success would be if we have 10s of thousands, hundreds of thousands, maybe millions of citations of the data and feature that shows that someone didn't just take it and upload it and make it available. It's actual, it's actually being reused by other people in the community, people are building upon it. And yeah, seeing the data actually being really used is, I think, probably the most important success criteria.	
Interview 1	50:07		Is there anything else you want to tell me about success or challenges or whatever comes to your mind?
Interview 1	50:14	Um, I think I think that's everything I think of right now.	
Interview 1	50:25	No, no, I think that's everything.	
Interview 2	Time	Interviewee 2	Interviewer
Interview 2	0:00		Short introduction
Interview 2	2:27		What about you? What is your background and what are you doing?
Interview 2		(anonymized)	
Interview 2	4:34		What is the history behind (platform)?
Interview 2	5:05	so origionally (platform) started with a different name it was called (anonymized). Research Data didn't have a very appealing name, right but but the idea was that	
Interview 2	5:22	institutions and research organizations that didn't have their own data repository to store for example, data sets or research outputs or any kinds of all sorts of	
Interview 2	5:34	artifacts that	

Interview 2	5:37	are produced from research, and they didn't have either the infrastructure or the the wasn't like a, like a national repository or something to to make the deposition subjects and then basically (company) started this small service at the time, which was exactly For this reason, so people go to (unv.) upload datasets, software slides, all sorts of other objects and kind of like how to place into to have them publicly available and share them and fight them. And	
Interview 2		eventually, this was kind of like work as a solution. It seems that it was a popular use case for for many institutions. And eventually (company) wanted to find a better home for managing the data and for for running the service and having like a nice, a better, sustainable, sustainable plan and how to do it right.	
Interview 2	6:14	Basically them (company) had already a data center and a lot of experience with managing big data and running services and providing infrastructure and hosting this kind of use cases. And that's how (platform) basically was one was born in (year).	
Interview 2	7:11	Another was launched as another way, running itself at this point. And it's been running since then for the last seven years, six, six years.	
Interview 2	7:26		And what is the core offering of the platform?
Interview 2	7:32	basically,	
Interview 2	7:35	to sum it up, right,	
Interview 2	7:37	they need to have a free available platform for any kind of user, not only in the EU, but basically on an international level where they can register the DOI for any type of artifact that's related to what we call the long tail of research. So it can be data sets can be software can be free. Yours. And an idea that also seems to be subject might get large over time, right? They would be. We provide by default 50 gigabytes	
Interview 2	8:13	in a storage space pair object	
Interview 2	8:17	that users can upload.	
Interview 2	8:20	That's the the core product of (platform) right? And then there's all these auxiliary services on top of that, which is like we the metadata. We store this data set base,where we provide this a community that a feature to organize these records and to manage them better than a github integration to make software preservation for example, or are you and then there's also all sorts of like programmatic API to access REST API, this IPM API's and	
Interview 2	8:58	other kinds of functionalities to build	
Interview 2	9:04		And what vision are you pursuing with (platform)?
Interview 2		basically, the vision is to, ideally (platform) would not exist as a Platform right, because each institution would have their own repository, its nation, for example, there would be a national level of prosperity that people would use. Or maybe this would be domain specific repositories where people could go and say, domain specific research that they're doing right.	

Interview 2		So then all these kind of like a is enabling all of these different enable all of these use cases that wouldn't normally exist. And all of these kind of data sets and all these research experts normally would be lost.	
Interview 2	9:11	There would be like, part of some hard drive or some server or some USB stick just imprisoned inside some of these, say private files and never be exposed as part of this this research chain right there to have a place where no matter what, you know, people will always be able to go the possibilities is able to deposit some of this research artifact and make them available and never have to worry about the servation aspect of it since So, and somebody's actually taking very good care of	
Interview 2	10:34	their their data and the files and the research.	
Interview 2	10:40		So, my work now is about success. And how do you define success or a successful platform?
Interview 2	10:51	So, I think successful couples have to have has to be easy to use. You have to have a very low entry barrier. People don't get scared away and, and think that it's very cumbersome to use it and begin to benefit others but I think it should be speaking to it should also encourage folks to start to try to speak to existing standards and best practices defined by all the different organizations like force 11, or IEEE and all of these things. So, for example, using commonly defined schemas, like data set, for example, or	
Interview 2	11:38	exposing, you know, ipmih endpoints, tracking user statistics that you should be using, for example, counter the counter called the practice. And I would say and the other part would be that successful panel should also have a sustainable time of keeping the whatever data and metadata know the subject and having a plan not only for startings in the current state, but also have a plan for what's going to happen after the platform perishes, right don't goes away or when there's no funding anymore.	
Interview 2	12:20		So, do you have more about success factors?
Interview 2	12:36	I think the other thing that can make (platform) kind of successfull is the partnerships and having finding nice synergies between either different platforms like Github, for example, or being able to serve a very specific very specific domains of science right? Because then that's actually enabling and, and making a lot of this research searchable and people can actually build platforms around and tooling around the platform, right.	
Interview 2	13:17		So I think and now I want to continue with a few questions about the organization behind (platform). So what is the organizational structure behind it?
Interview 2	13:34	So currently, it's not a legal entity in itself. It's part of self actually insane on itself is speaking, like a intergovernmental organization that	

Interview 2	13:51	is funded by has its own structure, internally like this, I think at the moment is five thousand people working on site, right. But in general, this is collaborating with other universities and institutions in this country send this. In at the end of the day this this almost I think 50,000 users, which are kind of like active	
Interview 2	14:20	and work either in collaboration with (company) on a visit from the Center for experimental, insights and again,	
Interview 2		I think the core part of (company) is experiments, the physics experiments have happened, but in support of these experiments is that there is a key department, which again, is split into further down into more groups like, which deal with the data centers, the storage, the services, that email services and all sorts of like the operational aspect of running an organisation.	
Interview 2	14:32	And again, on an even lower level. Part of the services is all is what we call the digital repositories section, which is also what portals and others. And this is where, for example, along with another some of our neighbor services is basically the internal repository that the person uses and where they stole all the videos and audio and	
Interview 2	15:31	the experiment, research artifacts and all of these kind of things.	
Interview 2	15:40	And then this (platform) repository which is causing in a sense / is set up and data, which is where all of the data that is produced from the experiment is kind of like a game made available	
Interview 2	15:57	and how many employees will. So at any time, we're aiming into having a group of around three to four people	
Interview 2		who are developing and, and running the infrastructure and infrastructure itself. But so it's a bit complex because (platform) team is for people who monitor the servicing and developing new features and running the help desk and all this kind of things, right.	
Interview 2	16:10	But (company) itself has a data center, which again employs almost like 500 people who are managing the infrastructure and the servers and networks and all the say, things that any other service would need to actually operate with it. So there's kind of like a hidden layer of of personnel but it's not very visible to the outside.	
Interview 2	16:56		And you are then all located in one team? Or are you distributed?
Interview 2	17:00	we are all in the same office actually in the same location	
Interview 2	17:08		and what competences do you and your colleagues have?
Interview 2	17:11	So we mainly looking for computer science and backgrounds. students write, web development and in general service management, product management personnel. So	
Interview 2	17:38	that's kind of like the core part for running (platform) specifically, right. But again, the rest of (company) This, again, a very diverse group of engineers and all sorts of other professions that are required.	
Interview 2	17:56		How are the responsibilities distributed?

Interview 2		So, basically, because of the nature of (company), and how contracts are being and how like personnel is being hired, and the contracts that take place, so there's a very high turnover factor. So people can attend and they will, they might stay on for a year, or they might stay for two to three years because of the contract tax that exists, and then this contract will span from five to eight years again.	
Interview 2		So there is that since the teams can be really big either. And the conflicts are very small. In the end, like everybody, does it be the very thing besides, again, the everybody's aware of technical infrastructure, everybody's aware, everybody knows how to develop features and market things this summer. Special technical specialties, right?	
Interview 2	18:01	So some people are experts on some things, but in general, everybody knows a bit of everything like everybody knows the social aspect doesn't know the community aspect development. And so it's kind of like the way of keeping it a theme that might be completely different thing for years. This means that everybody has to, like me be on top of everything and know how to run a service.	
Interview 2	19:27		I think the only the only differentiation, when it comes to decision, how are you deciding?
Interview 2	19:42	So, (platform) has a stearing boards as well, because most of us do, but they are not core members of the team but they are founding members of (platform) And let's say key let's say stakeholders, such as really kind of a bubble science and housing other reflect outside in the outside world, what kind of goals we have in what the vision was and and in general. So there's a steering board of three people and	
Interview 2	20:22	the idea is that, we we always consult also with the occasional funding partners. So depending on on the on the on the funding and the grants we have, at the moment we will, we will consult with services will be speaking but will consult with others to figure out what the what are the next features we're going to be developing, what kind of collaborations we can make and we can finally we can pursue these kinds of topics, Publix.	
Interview 2	20:55		So you are deciding On consensus?
Interview 2	21:06	Yeah, like because this, let's say, in terms of features it for example, like the certain features we have, we have gathered from users and partners and everything. So we have to prioritize all of these things. Right. And this always depends on the funding and the personnel that we have and what are the timelines that connected with the particle partners as well. So	
Interview 2	21:39	basically, planning responsibilities against the ground partners and that that's that's usually what's missing, but	
Interview 2	21:51		so in the beginning, you mentioned it's good or necessary to have a sustainable plan and how do you strategically positioned (platform)

Interview 2	22:05	So, we have some core partners like (company), for example, which is always looking into ways of, of either applying for funding or grants or so (platform) at the moment is mainly grant funded, funded by (region) projects and other private grants like from the (anonymized) Foundation,	
Interview 2	22:35	this kind of funding agencies.	
Interview 2	22:39	So, basically the responses we will always have on the loop for kind of like, proposals we can be part of and how we can host our different domains of scientific domains and what kind of features we can develop as part of these proposals.	
Interview 2	22:58	For example, now we also partner with With (platform), it is another repository in the US and	
Interview 2		So it is a big part of the financing, right this is basically the infrastructure is hospital itself is funded by all of the of its member states, nations. So which again is mostly (region) countries and some contributions as well from the from the rest of the world. But I think the biggest the biggest part is, is basically coming from the EU funding.	
Interview 2	23:09	And then we'll also looking into other ways of, of making possible again, the naldo and sons nonprofit rights and autism nonprofit as well. So we're also looking into, like, receiving funds from from individual projects. So making small collaborations with let's say, research groups that work on universities or	
Interview 2	24:01	private research institutions, they have workflows and you know they for all science, there's a lot of work that is being done with by diversity groups and because they need to start other figures and a lot of metadata faton because the graph and be part of (platform) is also funded by these kinds of initiatives.	
Interview 2	24:29		So for for my understanding those funding you receive - are they more project based and shorter or do you have also long term funding.
Interview 2	24:39	So, technically so is the local funding, part of infrastructure and hosting and being part of salary for individual	
Interview 2		but we also have two so this is let's say, like 10 or 20% of the operational costs, but for any kind of features and the running a help desk and basically the operations and the maintenance and all the things that this would be have to be extra funding	
Interview 2	24:55	So if we were to divide the operational costs that they are covered by sound, then the the maintenance and the health this can become like a technical support. This is handled by, again, funding, and then individual projects and individual grants, funding the advanced features and new things coming to (platform).	
Interview 2	25:41	at the moment things are grant funded	
Interview 2	25:48		And further question to the positioning part. So, when it comes to your community, how do you Describe the user group of the platform.
Interview 2	26:07	the general purpose supposedly, we don't host only one kind of the main, basically, we don't really classify content and users by any type of one of the common classifications that exists. So we get we get,	

Interview 2	26:25	we're getting, we have users from the (anonymized) university. We have users from astrophysics. We have users from the front of the human sciences right and social scientists	
Interview 2	26:40	of medical life sciences as well as our	
Interview 2	26:45	little datasets and software and you can department as well the condition all of them there. But in general, we kind of like we have a very diverse group of students from very different backgrounds.	
Interview 2	27:00		So in the future, do you want to have a more intense with a working with existing group? Or do you want to broaden the community size?
Interview 2	27:11	So we kind of want to do both. Because the interesting part there is that specific domains and specific use cases, they're trying to let the advanced, for example, how well metadata is captured and what kind of files or what kind of parts of the research process are being captured and preserved, right? So it's always interesting to look at specific examples, right, and how people want to, for example, annotate data.	
Interview 2	27:44	Like all these figures, and all of these	
Interview 2		come from biodiversity, for example, all these can, are usually part of the bigger graph that people want to connect between. And it's a very interesting exercise to develop that and chase after individual use cases and see what what each domain wants to get out of the other repository. At the same time, we want to also be able to, on the other hand, capture as much as possible, right?	
Interview 2	27:48	So this means that expanding these two different domains, and similar kinds of different research outputs can also be offered on (platform). But also of course, always it's a challenge because the idea is not always there. And not all domains of progress are progressing at the same rate, depending on how for example,	
Interview 2	28:40	how much technical they can get or how big is the it is usually technology that comes	
Interview 2	28:53	if they want to use a platform when it's a download, if you're using software to process some of the data or	
Interview 2	29:01		What specific goals that you want to achieve with (platform)?
Interview 2	29:09	So and even like in the short term, or like,	
Interview 2		more short term, think a lot of our efforts are in making the platform sustainable, So I'm trying to find the time to build the tools and processes that make For example, there's very big data sets, sometimes from (unv.)	
Interview 2		But the problem is that since our pattern by default offers 50 gigabytes a pair with a upload per object. For anything extra, we have to go into this This process of exploring funding options and how we can become have a collaboration	
Interview 2	29:15	And there's a lot of obstacles in between, usually legal obstacles into into sharing as an as an entity making making collaborations with individual groups all over the world, and the really heavy processor. But even though we are nonprofit, even though we don't	

Interview 2	30:25	like moving this direction, so all of our efforts now are into kind of like bringing, let's say, institutions and creating integrations between institutions and another. Because in this way, we can actually start mapping how all of these costs are kind of like distributed across domains and different institutions and how this you know, actually getting funding via how to make this this process of you know, The project funding which is usually some some things, actually projects include funding specifically for storage and the dissemination of for proper research. So we're looking into how we can actually make this even easier for for researchers and	
Interview 2	31:18	integrate this process inside.	
Interview 2	31:22		So when you set goals, how do you control them?
Interview 2	31:28	So, everything is kind of like focused around the funding aspect and this accountability aspect. And then it's also what what users are requesting and what kind of features users or users are required to do, cooperating with (platform). So so you asked how we define the goals for how we how we prioritize them, or	
Interview 2	32:01		Um, yeah, how do you control the goals when you set them?
Interview 2	32:05	So I think that I need to be able to always like ship things and you know, build things and be able to finish something with the resources we have and actually be able to deploy because otherwise it's because of also this very high smoke like high turnover like this and we have to be quick. In any kind of goal we have, like we have like very long term goals which spanned across like, years and years and smaller goals and smaller features that can fit in and satisfy more like smaller use cases, right? With a like a bigger goal of building a platform where everybody can, actually	
Interview 2	32:59	which is anybody worthless.	
Interview 2	33:03		Do you have KPI so something where you can compare, okay, we want to achieve this and this is the user number. And we are here or something like this.
Interview 2	33:14	So we have. So we track visitors and the number of records and number of the data volume for the knowledge	
Interview 2	33:24	is starting every year.	
Interview 2	33:28	We are mainly the things we're tracking. And then there's the aspect of communities and the number of users right.	
Interview 2	33:36	Let alone the server. In that case, again, what what we've seen in the past year is that there's always been a doubling every year, all of these all of these numbers, right. So that's kind of like the bar for us every year. So we're kind of like we're trying to see these trends and it's been Thankfully, things have been nice in line with his, with his projections. And basically, that's what we're looking at to see how successful the platform is. tracker.	
Interview 2	34:17		And what difficulties and challenges arise when managing (platform)?

Interview 2	34:27	since literally, the user base grows and the usage grows as time goes by, right, and as many types of different outputs and these outputs are shared, or journals and they're shared publicly someday there's gonna be news about some new discovery or some, some some research and there's going to be another link somewhere and everybody can click on it and try to download the data set or or see how the data looks.	
Interview 2	35:02	Or the site something, right. So	
Interview 2		that's usually a challenge because these are spikes of traffic and users that we see. And they've been growing as time goes by, you know, they've been growing more bigger and bigger. So one of it one of the challenges is dealing with this kind of individual use cases and, and hopefully, spectral popularity from time to time. The other the other challenges that a lot of people are building workflows on top of (platform).	
Interview 2		And these are automated workflows, which are not, let's say they're not driven by by by specific usages of progress, but but they are kind of like constantly running. Like somebody might be wrong might be validating some might be producing some research every month or something else	
Interview 2		Then having set up some some work for which, which includes another into this process, right? So the fact that these use cases are grown as time goes by this kind of like okay grows the, the standard load that another would get	
Interview 2		And of course, then we have also the use cases where people actually want to take what another really has, like harvest, the entire, say, metadata data set of logs and all the right all the right or the public records of the metadata records we have what's another and in these indicators, also this is this is it puts extra load on the system	
Interview 2	35:08	And we have to figure out ways to make things possible because we are actually valid use cases and, you know, we don't want to say no to be super possible, but at the same time we have to run the service and keep it stable for everybody to be able to use it, but	
Interview 2	36:56		you just mentioned the function of it. And just to make sure: Can you give me a brief overview of the functionality of (platform).
Interview 2		So basically, so the main is the object of what they call the records, right? All the records. These have device register for them. And they're kind of like two ways to go around creating these records. One is going by going through the, like, the manual process going via the UI, and creating, feeling in the metadata by hand and uploading files and, and, and then publishing the records and registering with the DOI actually	
Interview 2	37:09	And the other one is, it's about using the programmatic API's that we we provide, which I read REST API, rest again, basically. So this is where most of the entertain more complex workflows kind of like a are based on	
Interview 2	38:05	for example, that actually for putting content into (platform) like	

Interview 2		the inputs like aspect of this of this platform, right. So, in that sense the different workloads which are either automated via via services that are, some users are running, or, for example, the GitHub integration is working in this in this manner, right. This is like people published software on GitHub	
Interview 2		And then basically the software gets picked up by the noddle. And again, this is the cycle of, of files being created and records being created and DOIs registered and psycho chemicals alone. And then this research researchers that, for example, they they run regularly, they run some simulations, or they might run some machine learning and the content extraction classification algorithms and the results of these things.	
Interview 2		Again, they will anything want to do to preserve them inside another. So as part of their existing workflows, you know that the final step would be for them would be to whatever is produced by the REST API, there's another recipe would be a record would be created with this artifactory.	
Interview 2	38:18	on the, on the, let's say, on the output part of another way, this the OAI pmh endpoints API, which is for harvesting records. So this is this is where people for example can have like, Listen for For the changes in a specific domain, or in a specific community or an institution or a topic, or in general just leasing for changes in like new records are coming. But	
Interview 2	40:15		do you also offer some software packages on the platform?
Interview 2	40:25	I can think of their values like they are all implementations of tools tools around another. But we're not officially supporting any of these software. It's usually very specific to the to the to the use case of a specific domain. But there's nothing officially published buys an order that is used by doing to interact with one another.	
Interview 2	40:54		And what difficulties can arise when exchanging data via the Platform?
Interview 2		So I think the problem is is the level of of the metadata we capture, usually. So we're trying to do control the data set, for example, to the data schema like the (platform) metadata is based on the data site metadata schema. But of course, different platforms use different kinds of formats, right? This modeling for that was Mark XML, Mark 21, from older times, right?	
Interview 2	41:01	So converting between all of these different metadata format, that that's always a challenge. We think they are great with (platform), and being able to translate from one format to the other without loosing anything or keeping all of information in a machine readable way.	
Interview 2	42:00	Another general issue we dealings on in general is the fact that since the content is not curated by us, but on a very high level, right, we were just trying to curate the content. As part of the operation of some other we tend to do to filter out the spam records or misuse or abuse of the service, right. So there's a big process there and effort that's going into like tackling these kinds of kind of content that in a sense, is polluting the	
Interview 2	42:39	the output of other services. Now say that that's the main issue.	

Interview 2	42:50		Because you just mentioned the meta data and submission process. What requirements do you have for the submission of data?
Interview 2	43:00	So, basically, to begin with the top level, right?	
Interview 2	43:10	We have this 10s of users and other which are heavily based also on what (company) as an institution, like, supports on the values behind (company). So for example, we don't we don't start any, like military data or you know, like a data that can be used for military purposes or research that can be used for military purposes. Other than that, we we the metadata that user store, this is exposed as its cc zero license. So basically, it's a it's publicly available for anyone to download and to access. The files themselves are licensed by the users.	
Interview 2	43:53	The user is actually the copyright holder of the files themselves, right?	
Interview 2	44:00	And especially for to have a very low barrier, like entry level barrier. The only metadata where we require so much that the basically data site requires, which is the title list of offers under this description for the for the record. And the license for the for the files, which is, again, it depends on each use case, right. And it has to be at least also one file, like we don't register the eyes for, for just metadata. Like there has to be some some kind of research object that's, that's related to this video	
Interview 2	44:41		and how do you ensure the quality of the data set?
Interview 2	44:45	So, we try to automate the like the validation aspect of the data that is coming into the system, right? We tried to see that, for example, if people enter identifiers, let's, let's say all Kids or device or other kinds of types of identifiers, we try to validate all of these things to make sure that they they actually formatted correctly. They	
Interview 2	45:15	in some cases that they exist also right.	
Interview 2	45:19	But in general, in general, there is no human generated content of (platform). So, a lot of it has to have to rely on the automatic classification that we do from from like spam filters and other kind of like machine learning solutions we have developed over time. And then it's also community based so a lot of people will curate the content themselves like after, after uploading. But yeah, that's that's the biggest struggle for (platform). There's no easy way to control the quality of the data that is coming in. The only way is basically to form good collaborations with institutions to make sure that there's a steady income of, of, of quality data sets and quality research and quality as a object.	
Interview 2	46:18		And how do you estimate the reusability of the data?
Interview 2	46:27	we mainly focus on the beat level preservation of the data sets. We don't actually verify again, the fact that I gave an example that is the ZIP file that has been uploaded is actually the ZIP file order. The PDF is actually my PDF or any of these software checks, right.	

Interview 2	46:51	So as long as the data that hasn't been submitted is complete, and it's there's different kinds of checks to That we performed to see that, okay, the data that comes in is actually the data the user submitted and the one that we wanted to preserve, on and on that we were kind of agnostic to, whether like a black box at the end of the day, what kind of data comes in, like we try our best to, to, to showcase this data and preview it and kind of like, visualize it, but it	
Interview 2	47:24	ends up being like a black box	
Interview 2	47:28		related with the data quality is trust. So how do you ensure trust in the data?
Interview 2	47:39	That part is so since we cannot control what happens during during the submission, the data and what kind of objects the users submitted, the the best thing we can do is basically make sure that whatever is immediately preserved for the long term storage and and the The rest of the, for the for the lifetime of the novle (noble?) and beyond. So we basically run out of checks on the on the files after they have been submitted for there's always regular checks to make sure that the files are consistent, they haven't been changed as well, there is no what we call B-dropp (?) by data center and storage.	
Interview 2	48:26	We archive a lot of the data, we have processes for archiving the subjects along with their metadata and actually	
Interview 2	48:40	to preserve this whole workflow of who submitted what, when, how was the metadata changing over time and how the files were changed over time? If a change rates with the different versions of our objects	
Interview 2	48:56	but yeah, that's that's, that's . But what do we do? We we tried to keep a lot of copies of the data and archive them in common, commonly used for much.	
Interview 2	49:15		And because you you just mentioned you have no control of what people upload, and what is about ensuring how scientists use the data scientifically correct?
Interview 2	49:31	So, again, on that aspect again, we don't have something in places like we do, we try our best to create communities around the different data sets and the different kinds of objects have been uploaded. But at the end of the day, you just asked me to we end up with a lot of a lot of a lot of objects that are not like the best to be preserved. Eventually, like, we know the people will upload the upload the proprietary formats, like Word documents or into the PDFs, they will upload a custom format from from different kinds of software and there's no real way to actually force them to do some to do the correct thing.	
Interview 2	50:29	But yeah, this is the idea is that if the community itself, there are communities on (platform) that care about the data and care about this, about using the platform and preserving things correctly, setting up a reusability and these aspects of the Open Science right. They can set up workflows and they can they can achieve that by in on a community driven fact based.	
Interview 2	50:58		Coming to a question Regarding the community, how do you encourage researchers to use (platform)?

Interview 2	51:08	So besides our collaborations with the funding and the outreach of happens naturally by	
Interview 2		from our, from our partners, we also try to we tried to promote (platform) in institutions. And we will do webinars and talks and presentations. And we will have we always have materials are kind of like ready to send to people that want to start using (platform) or they want to set up a workflow.	
Interview 2	51:19	We tried to document some of the things that we were doing so all of the developments of (platform) happens in public. So everybody can like open and anybody is free to come and join. Let's say development process and comment on (platform), the source code of (platform) exists on GitHub. So, if you just want new features, for example, if they want to comment on how things are implemented, they can they can always do this. And,	
Interview 2	52:26	again, part of that is that writing proper documentation and, and, and conforming to standards and like trying to promote this this kind of way of working in the open. I think that that's also part of how people can get involved and how people see that these are not as a truly open platform and	
Interview 2	52:52	how they end up like putting the trust in the way topper in economic services.	
Interview 2	53:00		Do you see more ways of gaining new user?
Interview 2	53:07	So, again, new collaborations with institutions and creating these kinds of	
Interview 2	53:19	joining, for example, projects like the EOSC (region) Open Science cloud, right? So being part of this kind of catalogs is always beneficial because sometimes people indeed they have no idea where to go when they want to start to share the data and store them for the for long term preservation. We try to do as much efficient the outreaches as possible, but	
Interview 2	53:50	I think at the end of the day, it's really if we, if we manage to serve more and more use cases from the research community, and if we actually use into the problems that people have in the kinds of workflows they can do, they want to set up on top of (platform) and actually listening to the community and the users and satisfying these kinds of requests over time. I think that that's the way to do to move forward.	
Interview 2	54:21		So one special aspect about (platform) is that it's within the scientific community. So how do you think does the scientific culture influence the use of the platform?
Interview 2		You mean, like, like, how well for example, researchers are educated on it. For example, I think as long as a partner This big, big part of this actually is the publishing workflow, right? And the publishing process in general. So, I think, since it seems a lot is trying to capture basically objects that are not considered what we what we call the long tail of science, right?	

Interview 2	54:39	big part of it is the publishing workflow itself. And this is the it's the journals and editors of the journals and the data curators and the universities themselves and and the different projects and how they actually communicate that you know, doing scientist is one part but okay actually being aware of what kinds of aspects of the research can be preserved or should be preserved. And doing this in an open way and	
Interview 2		trying to find the, for example, ways to to to, to restore reproducible formats and captures the the research workflow and the reproducibility aspect of it right?	
Interview 2	55:50	So, I think as people know, all actors in this kind of process, get into this process and are educated about, about what is possible and how things should be done to avoid, for example, this reproducibility crisis that, like an experiencing now and i think i think it's it starts from, from, from the institutions, from the universities and the research organizations and all trickles down to individuals at the end of the day, right also the researchers themselves.	
Interview 2	56:45	And then again, journal editors. They should again know that, you know, if the see data set and software has been part of the let's say journal article, they should know that They should be stored, the DOI should be somewhere. And then again, the funding agencies themselves, they should also be aware that data sets and software and all of these, these are actually also part of the research. So for example, having a data management plan is a big part of making these things required for, for, for the deliverables, for example of a project. I think that's a good starting point.	
Interview 2	57:34		Coming to my last two questions, what development Do you see for the next 10 years and for (platform)?
Interview 2	57:45	So, we use you to plan but we expect them to be to exist for the next one years and this is the planets of the sun. Doing.	
Interview 2		So, basically we're looking into, into expanding into as many domains as possible, right. And eventually building enabling users to to be able to collaborate, to enter to enhance this collaborative aspect of, of having a platform working in the platform, and storing data on the repository and being able to curate it and, and have and build workflows around this. Because since since what what our main problem is also the quality of the data and the (duties?) to the curators.	
Interview 2	58:03	enabling users to do this would eventually create a platform that it's possible where it would be very easy to to do any kind of action and preserve any kind of object and work on it and collaborate and share it. Another second step also be able to do for workforce and all these things and be part of some bigger ecosystem with all of our efforts into into this into building communities basically going on. Because I think we there is a there is an obvious, it's obvious that people want people to put their content into (platform). So I think the next step is to be able to organize and create, actually with some community around	
Interview 2	59:31		and what does the success of (platform) depend on then?

Interview 2	59:38	in that case, it depends on being able to to connect these kinds of communities and whatever funding channels they would have, because I think all the different domains and different institutions, they work around the they have some funding models themselves, right. So it's being able to talk too (tap into?) and connect to these kinds of models and seeing how we can transfer either either offload For example, some of the operational costs to these communities, right? Or, or figure out ways for the community themselves to be easy to make it easy for them to offload	
Interview 2	1:00:23	this operational part by others. So, yep.	
Interview 2	1:00:34		Do you have anything else what you want to tell me about success factors, challenges or whatever comes to your mind?
Interview 2	1:00:42	So I think I didn't go very much into detail on one aspect of (platform) is that the the platform like the platform itself, but there's layers below (platform) just which is like, (platform) is a very thin layer on top of another basically piece of software which is calling (layer). And(layer) is this framework we have developed here at (company) which is for which is focused around building feature repositories. So, platforms like (platform) and other platforms like (other platform) and feature and obviously repositories that people want to	
Interview 2	1:01:25	be able to to start object and and expose them. So, the fact that we have a very solid core plat / like underlying platform	
Interview 2	1:01:39	which is a very active. And the thing even now, we basically what we have seen that happens is that people see (platform) as a platform and they want to set up something similar for example, for the institutions, right. So, for that reason specifically we said okay, let's let's take whatever is Know that the bottom layer of (platform) and create the product of itself and allow other communities to go and set up their own repositories, right.	
Interview 2		So in that sense having having a, you know, the thing has a platform, this (other platform), For example, this Fedora comments and all this other repository platforms that people can use to set up around the repositories	So basing / a repository based on these kinds of platforms is, is meant to, you know, it's meant to have a community on itself, like a meta community, on how to expand and how to have features and having new standards being integrated and coming back into the repository
Interview 2	1:02:14	So I think that's also one of the very important parts of it. Besides the social and community aspect Just being a repository of a specific domain or a general purpose domain, so the underlying infrastructure itself is pretty good though.	
Interview 2	1:03:09		Thank you.
Interview 2	1:03:11		So and one question just came up to my mind, you said that 50 GBs are free? Yes. What's where I have a data set that's bigger. That's okay for it.

Interview 2	1:03:26	Then basically, what we trying to do is, in that case, we try to measure the quality of the measurement quality but but see that, okay, it's a valid use cases, this is something that that it's actually required. And in that case, we have like a manual creation process where people can submit the request on our support line. And they can ask for if they have like 100 gigabytes or 260 gigabyte or	
Interview 2	1:03:58	something, something reasonable in terms of silence right volume.	
Interview 2	1:04:06	We can like go in on a per cases on a per case basis and try to evaluate the case is going to be cited by some, some published journal article is going to be, it is part of some funding is going to be presented on some, some workshop or some, some conference or something. So,	
Interview 2	1:04:27	it's kind of like a that's actually the only part where we we there is a manual operation done by us to see how we can accommodate these kinds of use cases.	
Interview 2	1:04:39	In which case we accept them and then it's okay. But again, we monitor in general the overall usage per user of automotive especially to provide the music and the services and operational Crazy.	
Interview 2	1:05:03		Great. So then I have a few points for my statistics in the end. So the software behind the platform is developed by yourself. And you also have an open source. Part of it. Right.
Interview 3	Time	Interviewee 3	Interviewer
Interview 3	00:00		Introduction
Interview 3	05:56		And what is the history of (platform)?
Interview 3		Yes, So this is where I look up my notes. And so, (platform) is (number) years old. It originated from an initiative among a group of leading journals and scientific societies in Ecology and Evolutionary Biology to adopt a joint data archiving policy. So because there was this idea that journals wanted to require data sharing, but if just one journal did it, then researchers could say, well, I don't want to share my data, I'm gonna go to this other journal.	
Interview 3		So a group of journals got together and decided to adopt a joint policy so that all these journals would do it at the same time. So it would really encourage the field around data sharing. And so out of that, they also wanted to think about, you know, where was it that they were going to be sharing their data? So the (platform) project emerged out of those discussions as a platform for the data sharing For this new policy, and it was really a recognition of wanting an open, easy to use, not for profit, community governed data infrastructure to support that policy.	
Interview 3	05:59	And so that's really still the guiding principles of the organization really connected to the research community. But now it works in all all domains of research. So it kind of started in that ecology, evolutionary biology space. And still, more of the publications are in the life sciences, but it supports all areas of research now.	
Interview 3	07:33		And what is the core offering of (platform)?

Interview 3	07:36	So we're a data. We're a platform, data sharing platform. And	
Interview 3		that's, so what we offer is that researchers through journals, so when people publish articles, so again, this is the origins of (platform) was that all data in (platform) was associated with a publication. So we wasn't just data that people had, it was always associated with a publication. Now, you don't have to have it associated with the publication. But there's still that idea that we want to have data sharing platform, but we also do curation.	
Interview 3	07:42	So we want the data to have good quality metadata for finding stability and accessibility. And we want the data to be of good quality, kind of conform to a few curation standards. So all the data that goes into jarhead, we check it before it goes in. So it's kind of when we say, a data sharing platform, it's that combination of the physical infrastructure of the platform itself, the sharing of data, the finding of data, and then that that curation aspect as well.	
Interview 3	08:48		Because you just mentioned the quality, I just want to jump to the question, how do you ensure the quality of the data?
Interview 3		Right, so for each data set, a curator looks at the dataset, they're checking the metadata for certain aspects. So we're a generalist repository. So it means that we don't have a lot of metadata fields, because we only have fields that are going to apply to every data set that we have in our repository. So those metadata fields and standards are selected around the fair principles, particularly the F and R, findable and accessible so we want good quality metadata that people can help find it for use and reuse.	
Interview 3		So we check that we make sure that things are correct. We might also recommend to the authors that they do some updates to make it a little easier for people. And then we download all the data files, and we make sure that we can open them that they're in a format that's usable. That doesn't just mean open formats. Some fields for instance, use MATLAB almost exclusively.	
Interview 3		So we do accept mat lab files, for instance. And then we're checking to make sure that they don't have personally identifiable information in them endangered species or sensitive sites, we require that all of the data that would be in English and we're also checking for licenses. So all of the data that we have in our repository is cc zero. So we need to make sure that people aren't submitting data that has a license that conflicts with the CC zero license.	
Interview 3		But then there are other things that the curators will recommend, particularly around like the readme files, to help explain their data and to work with the authors to recommend things that that help in that space as well.	
Interview 3	08:56	So it is a really challenging, I mean, maybe this is how you get to the challenges part. You know, it is challenging as generalist repository to be able to review all of those different types of datasets?	
Interview 3	11:05	That's our general approach.	

Interview 3	11:08		And going to the next question about success factors, because you know, my work is about success factors. And I'm quite curious, how do you define success or a successful platform?
Interview 3		Yeah, I think that's a really good question. I think thoughts, to be honest, more of what I'm thinking about as a new executive director. And what does success look for us? Like for us? I think that through the early stages of (platform) when data sharing was not very common, you researchers were still reluctant or concerneded about data sharing. It wasn't required many places.	
Interview 3		What success looked like was getting a lot of data deposited into (platform), right? So if we look at the number of data sets deposited over time, it keeps increasing and that is definitely a success. It's a success for us because it's That our platform is valuable. And, you know, we we have more data that we're making open.	
Interview 3		And it's also a successful from her perspective for the research community in that this idea of data sharing is becoming more normalized and accepted. Now that there is more more requirements, more acceptance of this as something that is done in research, I think one of the ways we're going to look at success is around the reuse side of things. So are people reusing it?	
Interview 3		How are they using it? What kind of impact are they having it with, with reuse, or reproducibility? So I think that might be a you know, don't mean you can quote me on this because I don't know that we necessarily, like decided that it that's a new success metric.	
Interview 3	11:21	But I think that that is something that's really important. The point of these platforms and repositories is not to just You know, store data for the sake of storing data, but to advance research. And so reuse is an important part of that.	
Interview 3	13:10		So are we now you're pretty new for (platform). And I'm curious, what vision Do you want to pursue?
Interview 3		Yeah, this is a pretty easy one, because we do have a vision statement. So (platform)s vision is to promote a world where research data is openly available, integrated with the scholarly literature and routinely reuse to create knowledge.	
Interview 3		And so I think that's, I mean, that's one of the things that really drew me to dry out as a role is because I think that that vision is so important.	
Interview 3	13:18	And, and so I think, you know, and you see there, the components we just talked about, where the data is openly available, and then also that reuse to create knowledge and I think both of those are really important and complimentary.	
Interview 3	13:58		So coming now to The first block about the organizational setup behind (platform). How is the organizational structure behind?

Interview 3		Sure. So we are a nonprofit organization. So we're our own organization. And we have a board. So nonprofits in the United States, most places has a board to 12 person elected board. And those people are elected by our members.	
Interview 3	14:10	And so we have member organizations, institutional publisher members, and they vote for those board members. The Executive Director, he reports to that board. And then and then we have a staff.	
Interview 3	14:45		How is this stuff structured?
Interview 3	14:47	Yep. So we have seven employees.	
Interview 3	14:52	And but we also have a partnership with the (company). And so our team, the team of People that work for (platform) has people employed by (platform), as well as people who are employed by (company).	
Interview 3	15:10		And what competencies do those people have?
Interview 3		So we have on the sort of across the team, but even on the staff, we have me as an executive director, and then we have an associate director. So we kind of have some programmatic work, but also kind of the business administration side of things.	
Interview 3	15:14	We have four curators. So those are the people that are looking at the data. On the (platform) staff. We have one developer and on the (company) side, they have the product manager and some developers as well.	
Interview 3	15:48		And how are the different responsibilities distributed?
Interview 3		So we have So essentially, we have like a curation team. So the set of people that do the curate And then we have the product development team. And so those teams, they work within in themselves to set the priority, you know, shorts, timescale priorities or make certain decisions.	
Interview 3	15:54	And then those decisions are in line with the ideas of where we want to go as an organization. So at sort of the higher level, that's where I'm setting goals of coordinating with the board, on setting more than the strategic goals. But day to day is left more in the hands of the individual teams and me and the associate director.	
Interview 3	16:38		So and then when you have to do decisions and you discuss it, or are you working with consensus, or how does it go?
Interview 3		Yeah, so definitely within the curation team, it's very consensus driven even when we have a question about a particular data set the characters we have a Slack channel carrier Slack channel. We'll post in there. What do you think about this? You know, we'll discuss it. And so it's very collaborative.	
Interview 3	16:47	On the product development side, it's the same, they work in two week sprints. So they decide their priorities, they back that out, they do their work for two weeks, they kind of do a, here's what we're doing. Here's what we did. Every two weeks I have stand up. So we are a very collaborative decision making organization.	

Interview 3	17:30		And when it comes to the strategically position of try it, and where do you see yourself?
Interview 3	17:39	Yeah, so I see that	
Interview 3		question is, you know, in relation to competitors, right. And so we don't focus too much really on that aspect of in relationship to our competitors. But what we do is really, we're really well integrated with the research community.	
Interview 3	17:41	So we work closely with that. In terms of what we see as priorities, or you know what works well for a design perspective. And so our board also has good representation from the research community. So when we're setting strategic priorities, we have our community while represented at that level of strategic planning.	
Interview 3	18:23		Because you just mentioned the community, what is your user group?
Interview 3		Yeah. So our user group is the researchers, essentially the ones that are depositing the data, the ones that are reusing the data. I think that that's something that will sort of continue to develop as an idea. I think we really talk with the community but in terms of like, who in our community, I think that's something that we're Sort of working on better defining in terms of just like who we sort of would interact more with on a day to day because a lot of our interaction with researchers is it's not a frequent touch, right?	
Interview 3		We were talking to them when they're depositing their data, or maybe they want to download the data. So that interaction, that what they're doing is something that we're talking to them about, about, you know, what makes sense for them.	
Interview 3	18:30	But outside of that interaction, what are the other opportunities to interact with the research community around data, sharing principles and in advocating for things like the fair principles and helping to support kind of the the ecosystem around data access and data sharing.	
Interview 3	19:52		So just to understand it a bit better. You don't have like a certain user community and specific fields, so you have like medicine, biology, and also social science and so on.
Interview 3	20:07	Right. All of those people deposit data with (platform).	
Interview 3		But in terms of how frequently do we interact with a (platform) community, it really right now is more at that point of when they're depositing or downloading data, not as much around sort of a continued ongoing conversation.	
Interview 3		But that's where we're we're looking to head more is to kind of create those opportunities to connect with people and talk more often and, you know, maybe even kind of sub communities within the community, right?	
Interview 3	20:13	What social scientists are thinking about is, you know, maybe different than what ecologists or physicists or people in medicine so I think that that's an opportunity for us to as we have more They open the repository to connect with individual sort of domains of research.	

Interview 3	21:07		And how do you encourage those researchers to use (platform)?
Interview 3		Yeah, we don't do much of that right now. The honest I mean, it's that people, a lot of word of mouth, you know, oh, you should use triad, you know. So within. That's why also within certain sub communities like ecology and evolution, were more standard they use because it's sort of what people use, and then they say, oh, why should I deposit my data?	
Interview 3		Oh, go put it in (platform), right? But the one thing that we do is we integrate with journals. So we have relationships with journals. So if someone is publishing a paper, and then they will the journal will say, and you should deposit your data at dry and then they'll send him a link and and how to deposit that and then we do that association.	
Interview 3	21:12	In between the journal in the data, so a lot of the that journal connection is a part of where researchers are contributing data is trying.	
Interview 3	22:11		Coming back to the organizational part. How is (platform) Finance?
Interview 3		So, the original financing mainly came from grants, the National Science Foundation, we still have some grants. So from National Science Foundation and the Sloan Foundation in particular, and but we also have a we have a data publishing church. So, for an individual dataset that you deposit, we have a charge we will waive that charge for people in countries classified. I can't remember the classification, but we will waive that charge for financial need, but also so things then.	
Interview 3		So those are the things but then we Also have memberships with journals. So it was just talking about that journal integration. So, for instance, and I was like, let me think of a new journal we have integrations with, well, to me, I want to say, I don't know, we'll just pick a life, we kind of have an integration with them. And if someone publishes with a life, they say, goes deposit your data in (platform), and then there's no charge because the journal is covering that fee for the authors.	
Interview 3		And so that's better for the author that's better for the journal. They don't have to figure out, you know, what do they need to do with the data, it's better for us just that we aren't. You know, we're kind of dealing with one organization rather than a bunch of individual authors.	
Interview 3	22:17	And, and then we also have more, this is something we're doing more of institutional memberships. So you're in Germany, Germany, in particular is very interested in German research. depositing their data within like the German library framework, or different German. There's there's national level things in Germany around data sharing. Sorry, one second my children.	
Interview 3	24:23:00	Sorry, I have to be right back just one second.	
Interview 3	24:26:00		Okay. Don't worry. Take your time over here. Okay.
Interview 3	27:40:00	Hello again	
Interview 3	27:44:00		so can you hear me? Yes, he just grabbed me some more water.
Interview 3	27:49:00	good plan. There we go.	

Interview 3	27:54:00	I think it was on institutional memberships?	
Interview 3	27:57:00		Yes and about the situation in Germany
Interview 3		Yes, Which there just are some really cool national level initiatives in Germany around data that is actually pretty unique and the library's engagement to there, but at an institutional level, even within the United States, and universities are deciding that they need to be responsible for the data that their researchers are producing. And so they're looking for solutions in terms of where they can have the researchers deposit data.	
Interview 3		So that's another thing that we're doing is we're having institutional memberships. So for instance, like with (university), they sign institutional membership and then they anyone at (university), now can put their data into (platform) to them for free, because (university) is covering the costs of the people at that institution. So that's kind of so that membership model is nice.	
Interview 3		It's a good model around finances. But it's also a really good sort of broader sustainability model because it is really that, again, connecting with the people that want to use the resource, and also helping the institution build the local capacity for data sharing and reuse.	
Interview 3	27:59:00	So a lot of the sort of institutional memberships are about not just about where do you put your data about a bit about helping them create those practices at their institution, so that they feel like, you know, this is something	
Interview 3	29:36:00	that we can do, we can support researchers into it. And so it was, I think, I don't know for sure. But I think almost all of those memberships are through the libraries. Because libraries are really being an important part of this ecosystem of data sharing and data management and provenance. Are there people that know about these things?	
Interview 3	30:00:00		Though is funding more long term or short term?
Interview 3	30:03:00	Yeah, so those are their year long memberships. And then they can renew those memberships once per year. Okay.	
Interview 3	30:13:00		And now coming off to two more short term topics, what specific goals do you want to achieve?
Interview 3	30:23:00	I think that's a good question. Um, I	
Interview 3		the specific goals, I would say is something having been here a month that I'm sort of trying to figure out, I think that they have been a lot around, you know, getting the data sets in making sure we have a good process for evaluating that data set.	
Interview 3	30:29:00	And I think, you know, looking ahead, we might set some other goals, I feel like they've sort of been we've sort of achieved some of the things that we wanted to achieve. So you know, sort of what's what's next.	
Interview 3	30:58:00		And when you set those goals, How do you control them?
Interview 3	31:02:00	I mean, I think it depends, right? What goals you set	
Interview 3	31:08:00	in terms of, is it something that you can do? Or is it something that you need to encourage your community to do? Right? So	

Interview 3	31:17:00	if we said, for instance, that we wanted a goal was that hundred data sets were used in articles after they were published, right? That's we can't directly control that. What we can do is do things to make the reuse easier. We could work with specific researchers maybe have fellows, you know, who could be exemplars for that.	
Interview 3		Or people who, you know, have prizes around data reuse, we could partner with them so that it's fairly community oriented. On that side of things, if our metric is more, you know, if a goal is more around, it takes 15 seconds to process a data set, you know, that's more directly in our control in terms of how we change our infrastructure.	
Interview 3		And so I think that that's just something to be when we're setting goals to be thoughtful about and what we can control versus what we can encourage. And I think there's this idea of like leading indicators versus lagging indicators. So you know, what is our long term goal?	
Interview 3	31:49:00	But what do we need to see now so that we can achieve that ultimate goal later? So setting setting those goals is it's really it's pretty important and so I don't want to say the specifics that's part of my three months is trying to figure out what those first revises Derby hang out what what are the what those specific goals are	
Interview 3	32:59:00		because reusability is so important topic for you. How do you estimate the reusability of the data?
Interview 3	33:08:00	Yeah, I mean, I think that the fair standards are one of the things that people know helps - I actually don't know the research around that be interesting - At least their suggestions that having fair data helps with reuse, because people can find it. It's accessible. They know how to use it.	
Interview 3	33:35:00	But how do you know if something's getting reused or what's important for you - switch?	
Interview 3	33:44:00		I mean, for me, your general opinion about it is important. Whatever comes to your mind.
Interview 3	33:52:00	Yeah, I think, I guess say I	
Interview 3		would say it's actually a lot like open source software in some sense and That things tend to be more used if there's more clarity around it, right? So if there's good documentation if there's a community around it, and so if there's other people who are using it, you know, a lot of it does actually sort of depend on the researcher.	
Interview 3		And, you know, Lenny, if they say, Hey, I have this data, and it's available, and I'm happy to help you reuse it. And here's the documentation. And I formatted it this way. And here's some scripts that do these data clean, like that data is going to have likely better reuse than an author that puts something out and says, Here you go, here's my data. Right?	
Interview 3		Because what is challenges to everyone's so busy? We have so many parties, if we have to really figure out what to do and do it. That's two separate things. If we can reduce the amount of figuring out what to do time to move it more towards being able to do something, then that's positive in terms of people's ability to use things.	

Interview 3		But it does require like, let's I think that's one of the things let's not pretend it doesn't require work. right in that first scenario with that author, they put a lot of work into that, you know, if they just published it for their use, they would have been done, you know, a long time ago, it's almost a little bit like the, you know, the 8020 rule.	
Interview 3		The last 20% takes up 80% of your time, right. And so in, I think that's similar in data sharing that last 20% that makes it easy for people to use actually takes a lot of time, a lot of time, a lot of knowledge.	
Interview 3	33:56:00	So I think that there's a lot of opportunity for education and valuing that work, respecting the time that it takes And that's a that's a cultural shift. So I think you know, a lot of even what we're talking about here is the cultural shifts within the research community.	
Interview 3	36:12:00		You're just touching another question of mine. I mean, one special aspect about (platform) Is that it's within the scientific community. So how do you think does the scientific culture influence the use of the platform?
Interview 3	36:28:00	Oh, it definitely does. I think especially because we have a cc zero license, which means you can reuse without attribution at different communities are more or less accepting of that as an idea. And so I think that does really influence people's choices around which platform to use	
Interview 3	37:01:00		Do you see other aspects of the scientific culture?
Interview 3	37:09:00	Yeah, I mean, certainly trying to think what what aspects are in it? I mean, a lot of it is tied to the open science principles, right, which is how do we how do we do science? Right. And I think that's a really a conversation we're still having, and I think the conversation is at different places in different fields of study.	
Interview 3		And I think that's why like I'm saying, you know, (number) years ago and (platform) started, it was really about get the datasets in because that was such a novelty, and I still think it is a novelty in some areas. And for good reasons, right. I think that there are different different domains also have different types of data that makes it harder or easier to share. communicate about, you know, I mean, I think actually, you know, qualitative data. Right?	
Interview 3		I mean, how would you share the these interview notes? Right, would you as the raw data to anonymize everything, you know, what are the practices? So I think that the culture is important and then, but then giving people guidance or education and building that in to sort of how do we how are we a researcher?	

Interview 3		How are we as scientists? Because I think that we don't really know necessarily, and then maybe when we do, we haven't, as researchers had the opportunity to really learn it yet. And this, you know, the education goes back to my carpentry work as well, which was, we were teaching people how to how to program and because so many researchers now need that in their work, but, you know, there's not yet that opportunity to learn it within most, you know, PhD Master's undergraduate programs.	
Interview 3	37:35:00	So people are needing to learn it sort of once they're on the job. I think that's that's similar around data sharing good data practices, and data reuse,	
Interview 3	39:13:00		and coming also to data practices. How do you ensure that the users of the platform use the data scientifically correct?
Interview 3		Yeah, we, we can't, right. Once it's there, and it's for anybody to use. I think, though, what we can do is the metadata helps, right. So one way that people can use data incorrectly is if there's not enough metadata to provide the context for a study and so that the data is used in a way that the study wasn't designed for.	
Interview 3		So I think, you know, in psychology, there's a lot more work around reproducibility And the Center for Open Science has done a lot. And it's, you know, it sounds like some of the challenges there is that, you know, maybe someone took a dataset and reused it, but the study design really wasn't appropriate, doesn't match the question they're answering, like the study was well designed, had the questions, but then reusing the data in a different context maybe isn't appropriate.	
Interview 3	39:27:00	So the metadata is really important. So both the sharing of the metadata, the evaluating of the metadata, maybe those are places that that can help.	
Interview 3	40:37:00		You just touched the challenges of this third time. So do you have any challenges in managing (platform) so far?
Interview 3	40:51:00	What aspects?	
Interview 3		I would say you know, I maybe the broader answer to that is That I think for any of the, you know, we're an organization. And we have a product, essentially right? And so there's always a couple of sides to all these. One is the product and the researchers and the curation and a lot of the things that we've talked about.	
Interview 3		But the other is you're running an organization, right? It's a small business. And so I think that it is, it's a challenge in this space where a lot of things start as projects in transition to organizations to how do you run an organization is different than how do you run a project?	
Interview 3	40:55:00	And, and so again, like in open source software, it's a similar thing where you have people that have started really amazing projects, and	
Interview 3	41:47:00	that got a lot of use, and then they're sort of	
Interview 3		big enough that essentially, they're an organization. You know, how do you make that transition? How does someone who is a software developer or scientist become immortal?	

Interview 3	41:50:00	Organizational leader What does organizational structure look like? So I think we're, you know, fortunate, (platform), it's been an on its own nonprofit for a while. So it has some experience in that space. But both of those things present different sets of challenges and sometimes different sets of expertise to	
Interview 3	42:22:00		because you just mentioned it is more or less a product. what functionalities and possibilities does (platform) offer.
Interview 3	42:30:00	Yes, so we are we're, we're platform. And so what we offer is that	
Interview 3	42:41:00	people can share their data, they put it their metadata, their share their files, and then it's archival. So we have three different places that we store the data to in the United States and one in (region), but was at the (company), and now it's going to be at (company).	
Interview 3	43:04:00	And	
Interview 3		so, and then you get a DOI associated with that data. So it's, it's archived, it's findable. So it creates it as a product for the researcher.	
Interview 3	43:05:00	And then the other thing we offer is right, the curation aspect so that we're going to look at your data, we're going to correspond with you as an author, to either say, you know, these are our requirements, we, you know, need to make updates or maybe offer some suggestions on how you could make it better.	
Interview 3	43:34:00		And do you have any applications on top or any other possibilities?
Interview 3	43:41:00	So we do have an API. And so we don't write like our packages, but there are packages for working with (platform). There's some updates now. We're working with (company). So what we have is the API and then we Work with researchers or researchers sort of do the work to do some of the integrations with with (platform) on.	
Interview 3	44:08:00		And when I would like to upload anything to dry it, what requirements Do I have to fulfill?
Interview 3		While you would sign in with your orchid, so we require orchid for sign in because we want to make that author data set Association. And then if you're looking to fill in our metadata, its title author, abstracts, and methods.	
Interview 3	44:21:00	So how you created the data are - I should look -Those are the main required fields, there might be a couple more, I can look.	
Interview 3	44:51:00	So there's a few required fields fields that you have to fill in. And then you have to upload your data set and it has to be typically in an open file. format, although, as I mentioned, you know, things like MATLAB, and, and it has to be not be corrupt. That's also good.	
Interview 3	45:13:00	And then kind of those other principles I mentioned around, you know what we're checking for in curation. So as a researcher, you really go, you input this information, you upload your data, and you press Submit. And that's sort of that's the process essentially,	
Interview 3	45:28:00		and what difficulties can arise when exchanging data via the platform?
Interview 3	45:34:00	So our data exchanges pretty robust. As far as I know, we do have a 300 gigabyte limit.	
Interview 3	45:44:00	And you can do	

Interview 3	45:46:00	data uploads via FTP rather than through the web interface. But the 300 gigabyte limit is also because it's not easy for people to use data that's more than 300 gigabytes, so we encourage people to split the data set up, if it's more than 300 gigabytes, I'm sure we could run into issues potentially. But I haven't seen too many since I've been here. So my sense is that that process is pretty robust. And	
Interview 3	46:20:00		when it comes to the trust in the data, I mean, it's quite different than the quality. How do you ensure that I can trust the sources?
Interview 3	46:32:00	And	
Interview 3	46:35:00	that's, you know, where does trust come from? Maybe trust comes from that authors Association, right, that you're a real person with a real orchid ID submitting the data, right? You can't anonymously share data. And then you really have like an institutional affiliation also with that author, so you have kind of	
Interview 3	46:58:00	the trust of that author. And that institution in the other trust is around me that information that they shared, and that someone's looked at it.	
Interview 3	47:18:00	You know, we can't caught fraud, we wouldn't detect. And because it would pass our checks, right? We don't rerun the analysis of a paper when we're looking at the data sets, and we don't. So if someone is trying to get away with fraud, that is something that would be difficult for us to detect and we leave that more to the research community.	
Interview 3	47:21:00		
Interview 3	47:52:00	So in that sense, I mean and that's an aspect of But that's that's a harder one.	
Interview 3	47:59:00		
Interview 3	48:02:00		Yeah, it is actually. So coming to the last two questions, and what development Do you see for (platform) in the next 10 years?
Interview 3	48:17:00	What a good question. I feel like if you asked me this in a couple months, I'd have a better answer.	
Interview 3		Um, I have a lot of thoughts about this. But I they're really by thoughts that are that are developing. Alright, so I don't know that I necessarily want you saying triad thanks. But I think you know, kind of that that piece we spoke about about the community, right and the sub communities and really.	
Interview 3	48:26:00	and I was, you know, also not normalizing that just data sharing, but the curation and Data Quality and having a better idea of what, you know, good data looks like	
Interview 3		and more robust, reuse or data sharing communities. So I think it could be really interesting to have kind of these these sub communities around like maybe (platform) as a platform, but maybe there's like a social science kind of front end to it, where social scientists can go and look for data and like have conversations maybe around the data, you know, within that system.	
Interview 3	49:07:00	So opportunities connect people around the data sharing aspect of things. I think the other thing that is literally like to see in the future is more of the sort of citizen scientist reuse of data. So	

Interview 3		kind of what I talk about is democratizing data. So we have data. And we have some people who have skills. But right now the people that have access to data and the people that have the skills to work at with it is a pretty limited set of people.	
Interview 3	50:01:00	And because data is so such a part of our future, and that we need to empower more people to be able to work with data, and that needs to extend beyond the scientific community. So if, you know someone is studying,	
Interview 3	50:33:00	you know, if someone lives in a city (city)	
Interview 3		somewhere, and they're interested in the water quality of their Lake, and they're not a scientist, well, if the scientist is setting that lake, there's information they should be able to have access to that data, to be able to answer a question about something literally in their backyard.	
Interview 3	50:40:00	Right, so connecting the data, you know, sort of about or with communities with those communities themselves, so that they can have that information available that to them to make the decisions that are important in their local context. So broadening out, who participates, I think in sort of access and use to scientific data.	
Interview 3	51:28:00	Since it's (number) years, I think that that's reasonable.	
Interview 3	51:33:00		Do you have anything else you want to tell me about success factors, challenges, whatever comes to your mind?
Interview 3		I think, you know, I mean, it's really a part of what I'm really interested to see, as you come up with, I think a conversation that's happening right now is where do researchers share their data? How do they make those decisions? So I think in general, the research community is still sort of wondering when they get to that point of published data, you know, what do they do?	
Interview 3		Where do they go? What kind of repository makes sense for them? Who do they want to have look at it. So I think that there needs to broadly across the repositories or agencies or institutions be more guidance for researchers on, on what they can do repositories can be a part of that, you know, we satisfy these requirements is Are those the requirements that you need, you know, but I think that there's just still a lot of confusion.	
Interview 3		And, and we see this we actually had an interview with a NIH, some NIH folks last week where they're asking questions about generalist repositories. And, you know, there's different guidance across different NIH, parts of the NIH for researchers. So that lack of clarity is a bit of a hindrance right now. And it'll never be perfect.	
Interview 3	51:41:00	And I think also the other thing is that there's not one answer. So you do need a robust ecosystem of data repositories, people They'll satisfy different needs. And so we don't want to get to a place where there's just one. But in that same sense, we need to help people then make a choice.	
Interview 3	53:15:00	So, we need a grid, a two by two grid, I would help.	
Interview 4	Time	Interviewee 4	Interviewer

Interview 4			Meine meine Frage am Anfang an Sie ist: in welche Plattform sind Sie jetzt eigentlich invobliert?
Interview 4	00:37	Plattform, was meinen Sie mit dem Begriff Plattform?	
Interview 4			Oder Datenbanken. Also (platform) ist für mich eine Datenbank-Plattform.
Interview 4		Also zur Begriffsklärung. (platform) ist nicht einfach nur eine Datenbank - Datenbanken haben viele. (platform) ist ein Informationssystem, was Daten hält, primär	
Interview 4		Wie sind ein Datenzentrum und ein Informationssystem. Also im generischen Sinne, weil Informationszentren eben wie gesagt beinhalten nicht nur die Technik sondernauch die Menschen, und die Menschen sind halt sehr wichtig (lachen). Bei Ihrer Frage nach nachhaltigkeit, sind eigentlich die Menschen wichtiger als die Technik.	
Interview 4	01:24	Ja, und. Formal, wie gesagt, sind wir halt ein Datenzentrum und das impliziert das dann.	
Interview 4			Ok.
Interview 4		Ja, das ist, ja(lachen). Plattform ist ein bisschen komisch, weil Plattform ist (-) ja, also, ist glaube ich der falsche Begriff.	
Interview 4			Weil Sie dann mehr sind?
Interview 4		Plattform ist einfach zu undefiniert.	
Interview 4			Ok.
Interview 4		Sie können auch meinen, ok, das sind dann die Hostinstitutionen oder was weiß ich, das ist der scope "Plattfrom (region)" was weiß ich. Das ist einfach zu undefiniert.	
Interview 4			Bei meiner Definiton handelt es sich genau um solche Informationssysteme oder auch Datenbanken, bei denen Forscherinnen und Forscher Ihre Daten hochladen und breit der Masse zur Verfügung stellen können.
Interview 4	02:20	Wenn Sie das so in ihrer Arbeit erklären, dann ist das sicher auch akzeptable (lachen)	
Interview 4			Ich habe auch schon gelesen, dass Sie bei (platform) aktiv sind?
Interview 4		Ja, ich koordiniere (platform) seit (nubmer) Jahren.	
Interview 4			Weil die / das ist auch eine sehr interessantes Interviewgespräch dann für mich. Aber heute soll es erstmal um (platform) gehen.
Interview 4		(platform) ist eine förderierte Infrastruktur. Eine fördertierte Infrastruktur, das heißt, da haben sich viele Einrichtungen zusammengeschlossen um eine Förderation zu bilden. Das heißt eine Konsoziale oder förderierte Infrastruktur mit entsprechenden Dienstleistungen zur Verfügung zu stellen. Die einzelnen Datenzentren, Repositiorien, Sammlungen, Museen, was hintersteht alles - die arbeiten da rein.	
Interview 4	03:30	NFDI - haben sie sicher auch schon gehört? Die Chemiker haben auch schon einen Antrag abgegeben.	
Interview 4			Ja

Interview 4		Sehr gut. Das ist übrigens ein gutes Arbeitsfeld für Sie, wenn sie mit ihrer Arbeit fertig sind. Also das wird auch eine förderierte Geschichte natürlich. Das sind dann auch Konsortien, die zusammenarbeiten und für die also auch ein gewissen legal frame geben muss, der sicherstellt, dass eben ein formaler Rahmen da ist und eine gewisse Arbeitsweise auch garantiert wird.	
Interview 4		Das sind auch so Sachen, (platform) hat halt auch einen formalen Rahmen und eine legal Base sozusagen, weil ohne dem geht es nicht. Irgendwas irgendwo im freien Raum machen wir nicht - das ist eine Grundvoraussetzung für nachhaltigkeit.	
Interview 4			Bei mir in der Arbeit möchte ich genau solche Hintergründe rausfinden, die dann clustern und weiter gucken.
Interview 4			Von vornherein möchte ich sie fragen: haben sie irgendwas, was sie mit mir klären wollen
Interview 4	04:50	Ne, sie sollten einfach ihre Fragen stellen.	
Interview 4			Wie kam es jetzt eigentlich dazu, dass (platform) entstanden ist?
Interview 4		ohje. Ja, wie kam es dazu? Ich war mal ein ganz normaler Wissenschaftler - habe also Informatik, Kunst und Geowissenschaften gemacht und habe damals Vbildverarbeitung von Partikeln gemacht , also Sedimentpartikeln und Geröllen und was weiß ich was. Und arbeite dann da beim Rechenzentrum beim (institut) ein Job bekommen.	
Interview 4		Das fand ich dann - genau wie sie damals - die Daten fand ich spannender als meine eigene Wissenschaft, weil die Wissenschaft selber eben dato ein bisschen was esoterisches, weil man doch mit seinem scope sehr limitiert ist.	
Interview 4		Also man muss sehr tief in die details reingehen und weitermachen um eben da ein Impact zu erzeugen. Und mit den Daten damals war das alles sehr neu. Und dann habe ich mit einem Kollegen mit der Geologie im AWI habe ich das dann angefangen.	
Interview 4	05:00	Ja, und dann haben wir angefangen Drittmittel einzuwerben und Projekte sozusagen dafür Dienstleistungen, Datenmanagementdiensleistungen zur Verfügung zu stellen, so hat es sich successive ergeben.	
Interview 4			War dann der Bedarf da sowas zu erstellen oder haben sie das dann daraus gemacht, weil das neu und spannend und eine möglichkeit war?
Interview 4		Es war neu, spannend, es war die Möglichkeit und es war die logische Konsequenz aus der vorgehenden Arbeit, wissenschaftliche Arbeit. Ich meine, heute hört sich das alles trivial an. Damals war das so, da waren große Zahlen, waren eher selten vor allem im Geo Bereich. Und ich hatte vorher im Death Valley und in (Kallavia?) gearbeitet und da haben wir auch schon, sagen wir mal, großskalig versucht Probleme anzugehen. Gerade auch mit den automatisierten Bildverarbeitungsmethoden war das damals erstmalig überhaupt möglich sowas zu machen.	
Interview 4	07:12	Und dann ergeben sich natürlich ganz andere Perspektiven, auch wenn man - ich weiß nicht ob sie stochastik im Stuidum gemacht habt.	

Interview 4			Ja
Interview 4		Wenn man initiativ in die Stochastik einsteigt, also für die ist es im Prinzip logisch, dass da erheblich viel mehr Potential drin liegt, wenn man Datenauswertungen komplexer Art oder auch großskaliger Art realisieren kann. Das heißt, Daten möglichst von vielen Providern, auch von unterschiedlichen Gruppen für kpomplexe Ansätze und so weiter.	
Interview 4		Also einfache Dinge sind natürlich so globale Datensätze, die bestimmte Merkmale abdecken als auch eben auch komplexe Applikationen. Da habe ich dann gedacht, als wir dann die Ebaloausbreitung zum Beispiel hatten. Das ist ja / werden, sage ich mal schon fast ein trivialer Forschungsansatz wäre gewesen das zu verbinden mit klimatischen, geowissenschaftlichen Daten um eben bessere Voraussagen zu machen für die Ausbreitung - und natürlich auch sozialwissenschaftliche Daten und wirtschaftlichen Daten um bessere Aussage machen zu können für die Ausbreitung voon Ebola.	
Interview 4		Sowas fällt einem dann natürlich ein. Und das lässt sich nur realisieren, wenn man halt konsequent aus allen Wissenschaftsdomänen Daten auch langzeitlich archiviert. Und auch da so rangeht, dass das eben nicht also / das ist immer noch das primäre jetzt nach einem viertel Jahrhundert. Das primäre Ziel, also die Daten werden erhoben, dann für so eine Art Erstauswertung, dann kommen irgendwie ein, zwei Paper irgendwie raus und das war es dann .	
Interview 4	08:40	Die Intention ist nicht, dass man jetzt globale Datensätze schafft oder eben mal systematisiert also auch dann solche Inventories - das kann man zum Teil kann man verstehen, weil Innovation ist ja immanent in der Wissenschaft. Also jeder Doktorand oder jeder Masterstudent, der findet natürlich seine Sachen, versucht daa irgendwie etwas neues zu machen. Und damit ist natürlich immer eine gewisse Konsistenz nicht geben. Irgendwelche neuen Messtypen, neue Methoden und so weiter. DAs ist immer so in der Wissenschaft drin.	
Interview 4	09:50	Aber es gibt noch sehr viele Mess- und Observationstypen, die stetig sind über die Jahrzehnte - manchmal noch viel länger. Temperatur zum Beispiel wird seit instrumeltenn seit dem 18.. Jahr gemessen. Und das sind heute wertvolle Daten, die man gut gebrauchen kann. Und selbst nominaldaten in der Richtung, ic h war mal in so einem Schwerpunkt mitbeteiligt. Da war eine Gruppe, die Papyrius aus Ägypten irgendwie ausgewertet hat, um Hinweise zu finden, auf das Klima zur Zeit der Pharaonen. Das (lachen) sowas gibt es auch. Und dann kann man diese Nominaldaten, die kann man dann auch auswerten. Also statistisch geht das alles. Das war die Motivation.	
Interview 4	10:50	Das zu tun.	
Interview 4	11:00		Und was ist dann das Kernangebot von (platform)?

Interview 4		Also wie gesagt, relativ eng haben wir angefange mit Paleoklimatischen Daten, aber ich möchte mal behaupten, wir sind einer der - also ich war schon - sind wir das einzige Datenzentrum, das einzige Informationssystem, was Cross diziplinäry ist und gleichzeitig die Daten hochstrukturiert und harmonisiert ablegt in einem relationalen System.	
Interview 4		Das ist / also die meisten von diesen, sage ich mal, sauber, gut arbeitenden Datenzentren, die arbeiten in einem relativ begrenzten Scope. Das sind zum Beispiel die nationalen Ozeanograpische Datenzentren, die machen also Temperatur, Salzgehalt und sowas ins WAsser, wenns hochkommt etwas Chemie, Nitrat und dit und dat. Das ist alles sehr überschaubar. Da ist man dann irgendie mit ein paar hundert Messgrößen ist man dann durch.	
Interview 4	12:00	Und wir haben rund 150.000. Und wir machen mittlerweise alles im Geo, Biobereich, wir machen jetzt mittlerweise auch sozialwissenschaftliche Daten und (-) ja. Das ist / Wir haben das dann ausgeweitet, weil wir die Expertise hatten. Vor allem am (institut), dann spöter an der Uni (town). Also ich bin ja jetzt seit vielen Jahren an der Uni (anonymisiert).	
Interview 4	12:40	In den Fachbereichen da ist die Expertise vorhanden und wir haben viel Unterstützung bekommen. Das ist soetwas, was wir als Peer Review bezeichnen. Und mittlerweile arbeiten auch viele Leute mit dieser entsprechenden Expertise bei (platform). Also wie gesagt, ich habe alleine angefangen, jetzt sind wir gefühlte dreißig Leute. Und werden vermutlich im Rahmen der NFDI nochmal so 15, 20 Leute zuwachs haben. Also das ist so die Erwartung. Im Moment wachsen wir rapide.	
Interview 4		Diese Expertise eben auch einer der Garanten, weil man muss die Sprache sprechen, die die Wissenschaftler auch sprechen, damit ein Verständnis da ist. Vor allem in der Anfangszeit war das sehr wichtig. Da haben wir halt - wir sind Schiff gefahren mit denen und dann sitzt man in den Instituten neben denen und sage mal, zeige mal her, was machst du da - OK, das sieht gut aus. Wurden die Daten dann einfach - damals noch Floppy Discs (lachen) - kopiert und archiviert.	
Interview 4	13:20	Also, viel Persönliches, viele persönliche Verbindungen, sehr großes Netzwerk, teilweise Leute, die - also die Fluktuation in der Wissenschaft ist ja SEHR groß. Und das sind teilweise Leute, die wir schon seit über 20 Jahre kennen, die dann irgendwo auf der Welt sitzen, die immer noch ihre Daten regelmäßig schicken und dann sozusagen Vervielfältiger sind.	
Interview 4		Die sagen dass dann ihren Kollegen und die machen das dann immer. Wir bekommen Daten aus aller Welt. Der Anteil der Nutzung aus Deutschland der ist unter 30 Prozent. EU ist irgendwie nochmal so 30 Prozent. Der Rest ist der Rest der Welt. USA ist viel, (country) ist viel, (country). (-) Also wir sind tatsächlich ein internationales Datenzentrum - haben eine große Vertrauensbasis da.	
Interview 4			Was ist für Sie denn ein erfolgreiches Datenzentrum?

Interview 4	15:10	Erfolgreich ist das, wenn man a es schafft, die Daten so abzulegen, dass sie effizient und also effizient nachnutzbar sind in einer eben für die jeweilige wissenschaftliche Applikation ausreichenden Qualität. Das muss man ein bisschen erklären.	
Interview 4		Wir haben bei uns so ein Dogma. Alles, was an Metainformationen vorhanden ist, wird erfasst und das wird gnadenlos harmonisert mit dem, was die Community Standards sind. Das heißt, da kann keiner irgendwie was eigenes, sondern das wird immer gempappt auf den Community Standard.	
Interview 4	16:00	Und wenn es eben Abweichungen gibt, dann muss man das aushandeln. Manchmal gibt es so Fälle, wo über mehrere Communities die auch schon solche Harmonisierungsaktivitäten angestoßen haben - wenn wir also feststellen, dass Units nicht kompatible sind, weil die einen machen gewichtsbasierte Messungen und die anderen machen volumenbasirte Geschichten oder die einen messen in Mol und die anderen machen was anderes. Und da kann man auch nicht mit trivialen Umrechnungen kann man keine Datenintegration hinbekommen.	
Interview 4	26:40:00	Da unterstützen wir halt über die Communities. Und das ist etwas, was die Effizienz letztendlich bei der Nachnutzung sehr erleichtert, weil also außer dass / innerhalb einer Community sehen die Leute: aha, das ist der Standard - kenne ich. Das sind Daten, so würde ich das auch erheben. Oder ich verstehe das zumindestens. Und dann haben wir die Daten natürlich auch immer im gleichen Format. Das heißt, man kann das auch programmatisch kann man das jkonsumieren.	
Interview 4	17:21	Wir haben vermehrt jetzt in den letzten Jahren, aRbeiten, Gruppen, die dann mit Statistiksscripten, R oder so, wir haben einen entsprechenden Webservices dafür - Python Schnittstelle und sowas da. Und dann werden Daten rausgesaugt eben für ganze Projekte. Hunderte, manhcmal auch tausende von Datensätze für bestimmte Apllikationen. Und das ist effizient und sowas ist gut. Das ist auch unterschiedlich zu dem - ich weiß nicht ob sie da (platform) oder Dryid solche Datenrepositorien, ob sie sowas kennen. Das sind so einfachere Repositorien, wo Daten Filebasiert abgelegt werden.	
Interview 4	18:10	In allen möglichen (main types?). Das ist dann häufig so, da ist dann vielleicht noch häufig so einfache bis manchmal sogar wirklich ausgedehnte Metadatenbeschreibung da, aber die Daten selber sind dann irgendwelche Excelfiles oder irgendwelche (-) was weiß ich, Textfiles oder so - das ist aber nicht harmonisiert. Da steht dann, irgendwas steht in den Headern drinnen, irgendwelche Werte häufig ohne Units und man muss sich das irgendwo rausholen dann - das kostet Zeit.	
Interview 4		Und wenn man jetzt über viele Providern hinweg dann versucht Daten zu integrieren, dann ist das auch eine Kostenfrage auch. Vor allem natürlich, das macht eine Gruppe dann immer wieder - was völliger schwachsinn ist. Und das ist mit einer der Erfolgsfaktoren. Wir stellen sogar ein Datenwarenhaus zur Verfügung. Da können sich die Leute innerhalb von Minuten riesige Matrizen herausholen.	

Interview 4		Das sind dann kompilate aus vielen Datensätze. Die dann natürlich nicht einfach so benutz werden dürfen - man muss schon in die einzeldatensätze reingehen und gucken, ob man das wirklich gebrauichen kann. Aber solche Ansätze unterstützen wir halt, das ist wichtig.	
Interview 4	19:40	Was würde ich sagen, was noch zum Erfolg beigetragen hat? Eben die Netzwerkerei, das habe ich ihnen schon gesagt, also die vielen persönlichen Kontakte auch, die wir haben. Das hat natürlich so einen Dominoeffekt über die Zeit auch. Am Anfang, ich sage es mal hanz salopp, hat es natürlich auch kritische Stimmen gegeben. Da haben dann Leute behauptet, ihr Auto sei nicht mehr angesprungen, weil sie (platform) benutzt haben. Also solche, also das ist jetzt überspitzt gesaft, aber das ist so	
Interview 4	20:00	Wir haben die abstrusesten Sachen auch gehört. Die negativpropaganda muss man auch sehr aktiv werden und versuchen auch immer - das ist auch ein Erfolgsfaktor - so schnell wie möglich sozusagen mögliche Fehler oder lapses irgendwas ausräumen. Und immer dran bleiben, solange bis (2) das beseitig ist - und das haben wir gemacht. Wir waren auch einer der ersten, die im Web, damals als es anfing, gab es ja noch kein Internet, und es gab ja nur dieses Unix-basierte Netzwerk. Also so eine Art Preinternet. Und dann gab es das APAnet, was die Militaries nutzen, das war schon ein bisschen schwierig. Da dann natürlich auch bei den Bandbreiten, die zur Verfügung standen, also wir haben verschiedenste Satelittensysteme in den verschiedenen Instituten. Die natürlich die Leute kannten wir alle, das war wieder dieses Netzwerk gewesen. Und heute haben wir einen zuwachs von rund registrierten Nutzern 1000 - 1500 pro Jahr. Wir haben zur Zeit etwa 15.00 registrierte Nutzer. Und wir haben angefange sie zu registrieren, das ist mal gerade (2) ja, wann haben wir damit angefangen?	
Interview 4	21:50	Ja. Und - was sollci hsagen? Was ist noch ein Erfolgsfaktor? Genau! Zu dieser Zeit haben wir angefangen ein Ticketsystem bei uns einzuführen. Weil die Daten, die Anfragen, auch Datensybmissions sind sehr, relativ gestiegen. Wir haben dann auch mehr Leute, die dann als Kuratoren oder Dateneditoren tätig waren, die dann mit den jeweiligen Datenprovidern kommuniziert haben. Da haben wir dann so ein Ticketsystem eingeführt. Das ist auch sicher ein Erfolgsfaktor, weil die gesamte Kommunikation, die natürlich auch als Dokumentation dient, also es ist im Ticketsystem abgelegt da und man kann die Vorgänge sharen untereinander, watch out draufsetzten, man kann die unterschiedliche sign dann an die jeweiligen Experten in den jeweiligen Bereichen und so - das hat die ganze Organisation vom Betrieb wesentlich verbessert. Ja und dann natürlich zum Schluss, naja und vorweg natürlich auch: wir haben ein paar gute Leute, die auch im technikbereich ziemlich gut sind. Wir haben so einen Überflieger auch dabei, der bei (anonymized) Foundation mitarbeitet, er ist corp commitor von Volltextsuchmaschinen - weiß nicht ob ihnen das was sagt, wahrscheinlich nicht so richtig (lachen=	
Interview 4			Volltextsuchmaschinen schon, ja.

Interview 4		Er macht da Geschichten mit Apache Lucine und Elastik. Das ist schon, also (-) sind so Leute, die sind bekannt, wie auch (platform) bekannt wie ein bunter Hund, weltweit - gutes Standing. Und, also, die daraus folgende Technik, die ist natürlich auch ein Erfolgsmoment, weil wir / wir haben einfach eine gute Technik, die jetzt wirklich up to date ist.	
Interview 4	23:40	Das, was wir da bereits in den (year)ern angefangen haben ist eine sogenannte dreibeinarchitektur mit Backend, Mittelware und Frontend. Und dadurch haben wir das geschafft, seiner Zeit habe ich das alles alleine aufgebaut erstmal, da haben wir das dann geschafft, das wir zwar mit einem hohen Aufwand an Mittdleware.	
Interview 4	24:40:00	Also das ist nicht ganz trivial gewesen, vor allem zur Zeiten, als es noch kein Java gab. (-) Diese Middleware komponenten waren alles C, C++. Das ist schon ziemlicher Aufwand. Aber daruch konnten wir im Backend konnten wir das Datenmodell konnten wir komplett normalisieren und dadurch haben wir die Flexibilität bekommen. Also wir können praktisch also an Datantypen einladen, was wir wollten, in das relationale System. Wenn jemand ankam, hat einen neuen Messtypen, dann wurde der einfach definiert und dann konnten die Daten reinlaufen. DAs war halt so ein gewisses Novum. Und im Front End bereich sind wir auch flexible. Als es das Internet noch nicht gab, gab es noch keine Web basierte Applikation im Front End Bereich - ist ja klar.	
Interview 4	25:36:00	Jetzt haben wir das, wir haben Webservices und alle möglichen Sachen und quasi Metadaten werden natürlich - was wahrscheinlich auch ein Erfolgsfaktor - die werden weltweit vertrieben. Manchmal harvesten uns die Leute anonym, wir wissen das manchmal nicht. Also erst, wenn sie Fehler machen, dann fällt uns das auf, dass sie da uns die Machine versuchen dicht zu machen. Dann werden sie abgedreht und / auch google zum Beipiel. Google über eine Sitemap, Google Data Search sind wir auch drin, wir sind coure resource von Geo, wir sind - also überall im Prinzip. Data One. Wir vertreiben die Metadaten eben. Das heißt, man kann uns auch überall finden. Das ist sicher auch eben auch vertrauensbildend.	
Interview 4	26:35:00		Verfolgen Sie denn eine Vision mit (platform)?
Interview 4		So ein bisschen, wir haben mit (platform) alleine kann man das nicht hinkriegen. Dafür ist das dann doch zu limitiert. Man sieht ja jetzt auch, innerhalb der NFDI, wir haben eine ganze Reiehe von Konsortien da. Und, naja, da gibt es halt - um jetzt mal so einen anderen, größeren Komplex zu nennen.	
Interview 4		Also die Mediziner, die auch in mehreren Konsortien jetzt darei ngegangen sind, die natürlich besondere Anforderungen haben. DAs ist für unsere Gesellschaften nochmal besonders interessant ist. Die Sozialwissenschaftler natürlich auch, also (institut), ist ja auch eine Einrichtung ,die ja auch schon relatvi lange besteht - mit dem wir übrigens auch einen guten Kontext haben. Auch ein etwas kleines, aber wir haben ein gemeinsames Projekt, wo wir dran arbeiten.	

Interview 4	27:35:00	Es ist so, wenn die Daten harmonisiert abgelegt werden in den jeweiligen Domänen, dann kann man mit überschaubarem Aufwand vielleicht auch KI gestütz, kann man eben eine Datenintegration erreichen auch über verschiedene Domänen hinweg. DA hatte ich jetzt für die NFDI ein Konzept entwickelt, das nennt sich Research Data Commons.	
Interview 4		Also der NFDI Research Commons. Also ein Commens. Da gibt es auch eine ganze Reihe von NFDIs commited. Es gibt ja die sogenannte (town) Declaration, wo der Commons sozusagen eines der Komponenten ist, die eben von einer ganen Reihe von Konsortien unterstütz wird.	
Interview 4	28:10:00	Und da ist schon die Idee, dass man die Daten, die in den jeweiligen Domänen gehostet, dass die dann cloudbasiert integriert werden. Also das ist sozusagen die Vision - die Daten aus (platform) gehen in die Cloud rein, werden dann nochmal weiter harmonisiert und kompatible gemacht mit dem was so andere Repositieren, Datenzentren lieferb. Und dann gibt es darauf gibt es verschiedene (-) so eine Art APllikationsschicht.	
Interview 4	29:15:00	Wir haben mit den Informatikern aus einer Reihe von Fachbereichen ein relatives detailiertes Konzept entwickelt. Das sieht dann vor, dass man einerseits einen Integrationslayer hat, der natürlich auch Termonologie gestützt ist. Das ist natürlich auch einer der Punkte, die ihnen villeicht über den Weg laufen wird - Vocabularien, (lachen) Terminologien, Onkologien, das wird immer wichtiger. Weil dadrin steckt dann auch die Semantik auch, also viel von der Semantik - was bedeutet das und so. Taxon also. wenn ich eine Spezies habe. Spezies bezeichne.Der eine macht es so, der andere macht es so.oder eine Lokation, oder - alleine bei Lokation	
Interview 4	30:00:00	also die Transkription zwischen den verscheidenen Sprachen erzeugt Probleme. Da brauchen wir halt entsprechende Lösungen, das können wir nicht alles selber pflegen, sonders dann kommt dann von ausßen, das können dann aber alle nutzen. Also die Datenzentren bereitstellen und auf diesem Wege kann man hoffen, dass es möglich ist, Daten über verschiedene Provider hinweg zu integrieren. Daraus entstehen auch größere, gepflegte Produkte zu machen. Mindestens aber, dass man so eine Art - wir haben das so genannt: semantische Container, die eben für bestimmte Applikationsspektren in der Wissenschaft hoch performant und (-) sehr effizienz Daten zur Verfügung stellen, wo die dann alles finden, was Sie brauchen.	
Interview 4	31:00:00	Jetzt und ihre eigene Daten noch dazu packen können und dann ihre Wissenschaft zu machen. Und das ist so meine Vision dabei. Wir arbeiten in Zukunft alle nicht mehr an Rechnern in den Instituten, zuhause oder sonst wo - natürlich schon, aber wir arbeiten cloudbasiert und kollaborativ. DAs ist die Erwartung.	
Interview 4	31:25:00		Um jetzt mal in einen anderen Bereich überzugehen: wie ist der organisatorische Aufbau denn hinter (platform)?

Interview 4		Wenn Sie noch, ja, schauen wir mal (guckt in seinem Computer) ganz kurz mal suchen. Dann kann ich Ihnen hier ein Dokument reinpasten.	
Interview 4		Falsches Teil (10) Bräuchte jetzt mal eine halbe Minute.	
Interview 4		Wir sind ja zertifiziert, wir sind ja zertifiziert nach einigen Geschichten. Also vor allem Contrastceal ist eine der Zertifikate, die wir haben, akkreditieres Data Center. Wir haben das international Councal for Science und wir haben - da gibt es (sucht weiterhin) kleinen Moment, ich finde das sicherlich gleich.	
Interview 4	33:05:00	(unv.) Weil das beantwortet da viel davon. Das haben Sie dann schriftlich und dann können Sie das lesen.	
Interview 4	34:04:00	Ok, das war der, das war das.	
Interview 4	34:45:00	Cotrast Seal - sagt ihnen das schon was?	
Interview 4			Als Zertifikat?
Interview 4		Ja (sucht weiter)	
Interview 4		Sind sie noch da.	
Interview 4			Ja, ich bin da
Interview 4		Ja, ich finde das jetzt im Moment nicht. Ich würde das ihnen noch schicken. Das ist im Prinzip ist das ein Prokoll dieses Antrages für die Zertifizierung. WO man halt sieht / diese ganzen Fragen werden von Contrast Seal auch gefragt. Ich bin ja mal als Teil von dem Scientific Board vom (organisation) gewesen. Seinerzeit habe ich die Zertifizierung auch mitentwickelt. Die folgt letzendlich dem, sagt ihnen (standard) was?	
Interview 4			(standard)? Nein, das sagt mir nichts
Interview 4		Das ist an der deutschen Bibliothek entwickelter - es gibt also ein internationales Bibliotheksbondont. Die haben dann letzendlich ein ISO Standard entwickelt und dieser ISO standard basiert wiederum auf einem anderen ISO Standard. Das ist die OES - also Open (Erciafs) Operations System.	
Interview 4	36:00:00	Wo letzendlich vom Injest, vom Daten zur Archivierung bis zur Disarminiation alles abgedeckt und darauf aufbauend wurden dann diese Zertifizierung dann auch entsprechend strukturiert. Und in Gänze auch die Organisationsgeschichten. Also wer / wie sind die Organisationen aufgestellt (-) im personellen, im organisatorischen und technischen Sinne.	
Interview 4			Und dann im organisatorischen ist dann auch, ob da irgenwelche / also welche Entscheidungswege es gibt?
Interview 4	37:00:00	Also, das ist alles damit drin. Das finden sie dann allles in diesem Dokument.	
Interview 4			Das wäre ja schön.
Interview 4		Also wenn ich es finde, dann schicke ich es ihnen.	
Interview 4			Das wäre dann wie Entscheidungen getroffen werden und wie die Entscheidungswege sind ist abgedeckt.
Interview 4		Ja, ist abgedeckt.	
Interview 4			Und wie der organisatorische Plattform dahinter ist, auch. Ok
Interview 4	37:38:00		Ich hake dann mal ein paar Fragen ab, die ich glaube, die damit dann fertig wären.
Interview 4			Sie schicken mir das dann spöter einfach, wenn sie das gefunden haben.

Interview 4		Ja, ich schicke ihnen das zu.	
Interview 4			Ok, das ist sehr gut.
Interview 4			Steht denn in dem Dokument auch, wie viele Mitarbeiter für die Plattform tätig sind?
Interview 4		Ich meine, das es da steht. Aber wir sind / aber das kann man bei uns auf der About sehen, wie viele da reinarbeitende. Wir haben - nicht, dass sie sich wundern, wir haben da auch Leute, die sind nicht in (university) oder beim (insitutut) ansäßig. Sondern wir haben auch Leute aus (town), aus (country), aus (town), (country), wir haben auch eine (country) dabei, also (platform) ist ein verteiltes system auch.	
Interview 4	38:45:00		Und wenn dann irgendwas entschieden werden muss, wird das dann in der Gemeinschaft entschieden oder wie funktioniert das.
Interview 4		Das steht auch in dem Dokument, wir haben im Prinzip ein Steering Comittee. Da sitzen auch die Direktoren sitzen dadrin. Da ist im Moment ein bisschen umbruch, weil ich bin sozusagen am rausgehen. Nachfolger ist schon gefunden, der wird das Ganze weiterbetreiben. Und da sind wir dann jetzt im moment dabei auch im Rahmen der kommenden NFDI, auch nochmal alles so ein bisschen umszustellen, aber vom Prinzip her bleibt es dasselbe.	
Interview 4			Und im Laufe der Zeit - haben Sie irgendwie eine Strategie entwickelt, wie sie sich etablieren wollen in der wissenschaftlichen Comunnity?
Interview 4	39:45:00	Ja, wir sind ja mehr oder weniger die Erfinder, wenn man so will (lachen). Es gibt / also das mit der Strategie ist ein guter Punkt, weil das haben wir (year/number) erstmals so eine Art Strategiepapier gemacht. Wir kommen allerdings nicht hinterher.	
Interview 4	40:00:00	Das braucht, also das muss, das muss renoviert werden. Also das ist, nicht mehr up to date. Aber es ist schon so, dass in den Köofen eine Strategie vorhanden ist. Es ist ungefähr so das, was ich ihnen geschildert habe, was so eine Zielvorstellung ist, eine Langzeitzielvorstellung ist, wie (platform) sich in die globale Landschaft also langrfristig einbettet und was es dafür dann übergeordnete Infrastruktur dann braucht.	
Interview 4	40:45:00	Das ist letzendlich die Strateige dahinter, die übergeordnete Strategie. Daneben haben wir auch kleinere (-) Ziele, sowas wie zum Beispiel mit den Terminologien. Wir haben so eine einzelne Sachen, das ist sehr wichtig, das müssen wir realisieren. Mit Terminologie ist sehr wichtig mit permanten Identifier, sagt ihnen sowas schon?	
Interview 4			Doi
Interview 4		Ja, wir sind ja auch die Erfinder von DOI. Das waren die deuschen Weltdatenzentren, die deutschen WDZ. Plus die TB in Hannover, die das (-) die das seinerzeit da in die Gänge gebracht haben. Und jetzt ist das ja eine international Association, ja, richtig groß und gut.	
Interview 4			DataCite

Interview 4		Ja, DataCite. Das sind halt so Komponenten, die wir auch in den 0er Jahren schon auf den Schirm hatten. Weil wir sagten, sowas muss es geben. Weil man muss ähnlich wie die Verlage, die ja damals schon DOIs benutzen, braucht man so eine Art Brand einerseits, aber auch andererseits auch (-) eben im Funktionalen Sinne. Das man dann garantieren kann, dass die Daten eben halt auf einer Domain liegen, auch wenn man so eine Domaine dann umstruktiert, persistend Referenzierbar bleiben in den Publikationen oder wo immer sie auch auftauchen.	
Interview 4	42:20:00	Bei uns die Daten die haben im Kern halten die Daten eine UOI, die sich aber nicht mit der DOI deckt, die werden darüber dann zusammen gebracht. Die DOI ist praktisch eine direktion, die garantiert, dass wenn wir im Kern umorganisieren, die DOI aber trotzdem noch valide ist, weil die dann einfach auf eine andere UOI zeigt. Das ist so der Mechanismus.	
Interview 4			Jetzt haben Sie ja von dem Strategiepaper erzählt aus (year/number). Kann man das auch öffentlich einsehen?
Interview 4	43:10:00	(guckt im Computer) Jetzt habe ich ja zuerst mal dieses Teil hier gefunden. Packe ich mal auf den Desktop.	
Interview 4		Und werfe Ihnen hier mal rein. Sie müssten das jetzt eigentlich bekommen.	
Interview 4	43:36:00		Ja, ich gucke gerade im Chat.
Interview 4		Das kann der dann nicht, wenn / naja. So, jetzt müsste das kommen.	
Interview 4			Beim Skype Chat selber komt noch nichts.
Interview 4		Ich sehe das aber schon.	
Interview 4			Ok, dann dauert das vielleicht ein bisschen, bis das übertragen ist.
Interview 4	44:10:00		Sonst alternativ - ah, da kommt es. Herunterladen.
Interview 4			Super,vielen Dank, dann gucke ich mir das danach einfach mal an.
Interview 4		Jetzt gucke ich mal kurz nach (guckt im Computer)	
Interview 4	45:45:00	Wir haben seinerzeit - alles in der cloud, alles Energie. Strategie und Implementation, genau. Was sagt er jetzt hier?	
Interview 4	45:30:00	Interessant, ok. (guckt weiter)	
Interview 4	46:15:00	Irgendwas stimmt nicht, das kann ich Ihnen dann danach auch zukommen lassen.	
Interview 4			Ja, gerne
Interview 4		Ein bisschen merkwürdig.	
Interview 4	46:50:00	Gut, muss ich mich später drüber machen. Ok,	
Interview 4			Gut, dann fahre ich mal mit der nächsten Frage fort.
Interview 4		Ja.	
Interview 4	47:15:00		Wie erfolgt die Finanzierung der Plattform?
Interview 4		Im Moment noch überwiegend über Drittmittel. Über viele Jahre hatten wir ein Drittmittelanteil gehabt, der über 80 Prozent lag. Das ist übrigens einer der Erfolgsfaktoren, das darf nicht zu viel sein. Das ist schon eine gewisse Gratwanderung da also dass man dann guckt, dass immer genügend Mittel da sind, damit man vor allem die Leute halten kann.	

Interview 4		Und jetzt liegen wir so bei 60 - 70, 60 Prozent eher, Drittmittel. Und der Rest ist fianziert über das Marum in (town) und das AWI im Bremerhaven. das sind dannn fixe Stellen, die dann da reingehen. Aber am Anfang waren wir praltisch 100 % Drittmittel
Interview 4	48:15:00	Das ist schon eine gewisse Gratwanderung, muss man eben sagen. Aber ist halt auch so, das ist auch einer der Punkte, die in die Nachhaltigkeit sehr stark reinspielen, es müssen um Dinge zu einem bestimmten Zweck machen zu können muss man eine gewisse Größe haben. Und wenn die Größe nicht da ist, dann (-) weiß nicht, sie kennen sicher ein paar von diesen Repositorien, die relativ speziell sind, die an einzelnen Personen irgendwie hängen, viele davon auch sehr historisch schon. Also die sind / also davon haben wir eingei davon schon über die Jahre geschlachtet auch ,weil die Leute dann in Rente gehen und dann weg sind und so.
Interview 4		Als auch eben Repositorien, die im institutionellen Rahmen auftauchen, das ist etwas, was vor allem die DFG seinerzeit induziert hat über die Regeln guter wissenscafhtlicher Praxis, dass man Daten mindestens 10 Jahre lang aufheben sollte. Und da ist dann im Rahmen von vielen Universitöten sind diese Repositieren aufgetreten, die aber im Prinzip keine Rolle spielen.
Interview 4	49:50:00	Weil die Daten / also entweder es ist marginal, was da überhaupt abgelegt wird oder es ist (-) nicht brauchbar, das Zeug. Das ist dann so wie (platform), das kann alles mögliche sein, man hat keine Sicherheit irgendwie, Kommunikation ist halt auch schwierig, wenn man wissen möchte, was mit diesen Daten los ist.
Interview 4	50	Und diese Sachen sind einfach alle zu klein. Das ist / das funktioniert nicht. (platform) hat insofern jetzt ein gutes Standing weil die auch Software machen, weil die auch, sage ich mal so, es auch erlauben Dokumente, Assets, was wir füher als graue Literatur bezeichnet haben, mal schnell eben persistent abzulegen, DOI und so. Bei Software sind sie relativ gut mitlerweile. Aber (platform), was auch ein relativ großer Player ist, von der (insitut), die sind, sage ich mal, die machen überhaupt gar keine Kuration und das ist eine reine Ablage. Das ist praktisch nicht brauchbar. Die sind auch letzendlich zu klein
Interview 4	51:14:00	Also die menpower dahinter und die, die sie haben, da stecken sie mal einen guten Teil in die Promotion rein, was zwar die Sache bekannt gemacht hat, aber die Qualität nicht besser. Also man muss im Vergleich, man muss das sehen. Es gibt also Datenzentren, die kennt kein Mensch, die arbeiten zum Beispiel - was heißt kein Mensch, die sind für bestimmte Communities halt da. Da arbeiten viele, viele Leute, teilweise hunderte.

Interview 4	51:50:00	Die halt nur einen bestimmten Zweck haben. Die Daten sind hoch konsistent, gut im Zugriff und so weiter, aber da wird nicht so ein Budenzauber drum gemacht. Sondern das sind wirklich etablierte Einrichtungen, die über viele Jahre schon arbeiten und auch garanten sind, dass bestimmte Daten, dass die nicht verloren gehen. Und im moment konzentriert sich viel auf eben (-) die gehypten Dinge - (platform), was da alles. Fighare ist auch so ein Ding, da kann man alles reinwerfen und ist im Prinzip wenig brauchbar.	
Interview 4			Eine Nachfrage zur Finanzierung. Nun haben Sie erzählt, dass man die Waage finden sollte zwischen Drittmittel und längerfristiger Finanzierung. Wie ist das denn bei Drittmitteln, sind die nur für zwei oder drei Jahre begrenzt oder wie läuft das?
Interview 4	52:50:00	Ja. Aber es ist halt so, wir machen viel so Infrastrukturprojekte, also im EU Rahmen sind sehr viel auch. Also diese H (year/number) - wir sind da im Prinzip schon seit. Das erste Ding, was wir hatten, war ein Mast-Programm der EU. Das ist noch Ende der (year) gewesen. DAs hat den großen Vorteil, dass wenn man drinnen sitzt, dann macht man Dinge, die auch relevant sind auch. Das ist nicht so, dass denken wir uns mal aus oder so. SOndern das sind Dinge, die werden auch / erfordern, dass man sich in größeren Konsortien abspricht und guckt, was ist überhaupt sinnvoll.	
Interview 4	53:50:00	Am besten sind die Projekte, wo man direkt in die Wissenschaft mit eingebettet wird als Datenmanagementpartner. Das ist auch so - da haben wir eine Seilship. Also kann man auch als Seilschaft bezeichnen, wo uns dann auch Communities immer wieder in solche Projekte mit reinnehmen als Datenmanagementpartner, weil sie gute Erfahrungen gemacht haben. Und das trägt erheblich bei auch dazu, dass man ein Standing in der Community entwickelt	
Interview 4		Das man bekannt, einfach bekannt ist. Aber daneben hat man natürlich ein Basisbetrieb, also von der Technik her, von (-9 grundsätzlich eben halt die Organisation und so weiter und so weiter. Und dieser Basisbetrieb der muss im Prinzip fix finanziert sein und das war am Anfang nicht und das ist es jetzt und von daher können wir uns schon so ein bisschen beruhigt zurlehnen und sagen: ok, wir sind hinter dem Berg. DAs hat so ein bisschen was von einer Firma.	
Interview 4			Und diese Langfristige Finanzierung ist dann geknüpft an irgendwelche Bedingungen?
Interview 4	55:15:00	Ne, also die Host-institutionen sind ja das (institut) und (insitut) von der Universität (univerity). Und die Uni hat sich kommitettet und das (insitut) hat sich auch komittet. Wir haben gesagt: ok das wollen wir langzeitig wollen wir das unterstüzen. Was dann langzeitig heißt, inwieweit das justizable ist kann natürlich keiner sagen. Wenn die Politik beschließt, dass die deutsche Politik beschließt, dass die deutsche Bibliothek zugemacht wird, dann wird sie zugemacht. So ist das (lachen).	
Interview 4			(lachen) gut, aber das Kommmitment ist dann ja immerhin schonmal da.

Interview 4		Ist da, ist wichtig.	
Interview 4	56:00:00		Welche Schweirigkeiten und Herausforderungen gibt es denn beim Management von (platform)
Interview 4	56:15:00	Ja, also sie kennen das sicher, weil sie auch Wirtschaftswissenschaften machen. Dass ist schon so, wenn man eben halt klein ist, dann halt man alles sozusagen in seiner Domäne, dann kann man, sage ich mal, die Schrauben so drehen, wie man will. Je größer man wird, desto mehr muss man also delegieren. Man hat dann mehr Worksharing. Und hat natürlich auch einen höheren Organisationsaufwand und dann natürlich auch für de Governance, also Abstimmungsprozesse und sowas -wird alles aufwendiger, muss man halt sagen.	
Interview 4		Letzendlich haben sir successive versucht, immer wieder dagegen zu arbeiten und zu sagen: OK, dann verbessern wir unseren Organisationsgrad an dieser Stelle, das machen wir neu, das müssen wir so und so weiter. Eben so wie mit diesem Ticketsystem, dass dann auch an einer bestimmten Stelle eingeführt wurde. Einfach um zu sehen: OK, damit bekommen wir die ganzen Kommnikationsprozesse rund um Technik, Datenabgaben und sonst was, das haben wir alles dann gut in Griff, wird transparent für alle und wir erkennen Probleme schneller und so weiter.	
Interview 4		Also. Das ist eine Herausforderung, würde ich mal sagen. Also, dann kommt jetzt eine Gewisse Inkurstation hinzu - das ist auch so, wenn die Personaldecke größer wird und Leute schon lange dabei sind, ist auch Motivation ist eine, ist eine große Herausforderung. Wir mischen halt junge und ältere Leute - so wie mich (lachen) und versuchen eben halt aus dieser gemeinsamen Gemengenlage auch für alle so die Motivation oben zu halten.	
Interview 4		Bisher kann ich sagen, dass es ganz gut funktioniert. Wir haben jetzt nicht (-) also das ist nicht so ein bürokratischer Apparat, der/ wo die Leute dann morgens kommen und irgendwas maschen da und man weiß eigentlich nicht mehr so genau, wohin sie sich dann bewegen. Also es ist schon so, dass wir (-) einen guten Zusammenhang haben. Also es finden alle sechs Wochen finden regelmäßige Treffen statt, wo auch überregional die Leute kommen.	
Interview 4	57:30:00	Höufig eben auch mal aus (country) sind die Leute dabei, wir fahren auch mal hin. Leute aus (country), dann, also. Da wird eine Dame wird da bezahlt von der Europäischen Atombehörde da, das ist /die hängen dann an irgendwelche H(year/number) Sachen hängen die drin. Das ist für uns dann auch profitabel, wenn man dann rumfährt und Face to Face meeeting dann mal macht und dann guckt: wo stehen die, was machen wir uns do weiter.	
Interview 4	59:10:00	Also es ist ein gewisser Communicationsoverhead, aber man kann das schaffen. Ich meine, das schaffen andere ja auch - ne gute Firma, wo man mehr als dreißig Leute, funktioniert das auch (lachen)	

Interview 4			Nun haben Sie schon häufiger die Datenqualität angesprochen, die es da auch gibt. Wie stellen sie denn sicher, dass eine ausreichende Anzahl an Personen entsprechende Daten mit Datenqualität hochladen.
Interview 4	59:54:00	Also dass Daten bei uns abgegeben werden, da brauchen wir uns nicht, NICHT mehr drum zu kümmern. Wir kriegen mehr Daten als - im Prinzip - als wir bewältigen können. Es ist, also wir, wir - weiß nicht wenn Sie da bei uns auf die Homepage da gehen und auf dieses Submission Button klicken, dann sehen sie schon, dass wir so ein Warning darein gebaut haben, dass es länger dauern kann bis wir die Daten drin haben. Wir werden wirklich zugeschüttet mit Daten.	
Interview 4			Und wie stellen Sie dann die Qualität sicher?
Interview 4		Ja, wir haben - wie gesagt, wir haben gute Technik, gute Systeme, wir haben ein, ein, eine editorial System, was sicherstellt, dass auch sicherstellt, dass alle Metadaten / das Editorialsystem ist mit dem Ticketsystem verbunden. Das heitß also die Datenabgabgen werden automatisiert rübergeholt in das Editorialsystem. Die Daten werden harmonisiert mit den Inhalten, die wir jetzt schon in der Datenbank drinn haben. Also Autoren werden abgeglichen, Referenzen, dann haben wir einen Websiten auch über Crossref, den wir da nutzen. Da reicht im Prinzip die DOI ob zu erkennen, ob es so ist wie es beschrieben ist, Institute - was weiß ich. Also eben alles, was man so braucht an Metainformationen. Da findet ein kompletter Abgleich statt. Was fehlt, wird neu eingefüttert.	
Interview 4	61:25:00	Und dann gehen die Daten, die eigentlichen Daten, gehen dann rein. Das ist, also vor allen Dingen in Mess- und Informationstypen und Methodik, das muss abgeglichen werden, wie ich das schon schilderte. Diese Typen, dass die - wir nennen das Parameter - dass die harmonisiert sind.	
Interview 4		Ja, und das ist eine richtige Redaktionsmaschine.	
Interview 4	62:00:00	Nur so, wir haben so im Schnitt so ein Dutzend Datenabgaben pro Tag und ja. (--)	
Interview 4			Das ist ja auch nicht wenig.
Interview 4		Nene Und dann kommen häufig eben noch so Sachen dazu, die auch automatisiert reingefüttert werden. Also Daten, die regelmäßig erhoben werden, die können automatisiert reingefüttert werden, also für Daten, die regelmäßig erhoben werden, die können wir dann vorkonfigureren und die müssen nur noch abgenickt werden. Also der Zuwachs ist in der Gegend ungefähr so bei 10.000 Datensätze pro Jahr.	
Interview 4	62:40:00		Wie ist das denn mit den Nutzern der Plattform? Welche Regeln unterliegen die?
Interview 4		Die Terms of Use. Da sitzen wir auch im Moment dran. Das auch / Wir haben im Prinzip nur rudimentäre Terms of Use jetzt. Wir müssen da jetzt auch mit dem neuen Datenschutz Verordnung müssen wir noch nachlegen da. Sind jetzt mit Juristen irgendwie da im Gespräch um das mal vernünftig aufzusetzen.	

Interview 4		Im Prinzip ist das so ganz grob über die Regeln zur guten wissenschaftlichen Praxis einerseits geregelt. Das heißt extern, (platform), sogenannt common consens (-) Und andererseits müssen wir die kreativ commons licencen, die ja Attribution Licence ist das, was wir primär empfehlen, die Leute können auch was anderes wählen, aber das ist eigentlich nicht sinnvoll.	
Interview 4		Und daraus resultieren dann halt die, zummindestens die Vorgaben für die Nachnutzung, für die Datenabgabe ist es klar dass also die Daten Nutzer bei uns, wenn sie also einmal den Datensatz publiziert haben, dann können Sie den nicht mehr so einfach rausholen.	
Interview 4		Zu sagen: hey, ich will da nicht mehr als Autor stehen oder zu sagen, ich möchte den Datensatz jetzt löschen oder so, das ist nicht so einfach möglich. Also wnen dieseer Datensatz wirklich korrupt ist, eben halt sehr fehlerhaft ist oder so, dann würde man eher eine neue Version da reinbringen mit dem Hinweis. Wenn es tatsächlich so ist, dass dann da Daten gefälscht wurden oder so, dann würden wir den schon also auch löschen mit einem entsprechenden Hinweis.	
Interview 4		Man findet das dann schon noch, aber eben nur Metainformationen mit dem Hinweis, dass das eben halt (-) gefälschter Datensatz war. Solche Sachen , sowas können wir machen und das wissen auch die Nutzer wenn sie bei uns die Daten abgegen, dass sie eben halt da (-) sage ich mal, dass das Copyright wie bei den Verlagen geht natürlich zu einem guten Teil auf uns über.	
Interview 4	64:10:00	Aber jetzt zum Beisiel was Datenschutz angeht, sind auch viele Daten unter Moratorium. Dass man einige, vor allem wenn Projekte noch nicht abgeschlossen sind das überlassen wir dann komplett den PIs, die können das dann entscheiden. Die sagen, die, der kann die Daten haben oder der kann sie nicht haben. Das ist bei uns auch alles so halb automatisert, das kann man dann anfragen. Und dann bekommen die einen Temp-Key und können dann darauf zugreifen.	
Interview 4	65:47:00	Oder sie kriegen Dauerrechte auf den Datensaz - wie man das auch will.	
Interview 4	66:00:00		Wie beeinflussen Sie denn, dass die Daten auf (platform) korrekt, und auch ethisch korrekt verwendet werden?
Interview 4		Ja, das ist so wie in der Literatur. Wenn der Datensatz runtergeladen ist, und weiter verwendet wird, dann haben Sie im Prinzip keine Handhabe mehr. Wir können das nicht kontrollieren. Wenn jetzt jemand ein Paper irgendwo runterlädt und schreibt gnadenlos, also verwendet irgendwelche Ideen, zititert das aber nicht, dann (-) wie gesagt, dann geht es einem vielleicht wie unsere ehemlige Forschungsministerin oder so oder anderen, die dann ihre Doktorarbeit irgendwie in die Tonne treten können.	
Interview 4	66:50:00	Das ist aber, ich möchte mal so sagen, selten - das solche Sachen wirklich transparent werden. In hoch umkäpmften gebieten oder wenn die Leute sehr prominent sind, dann mag vielleicht also angehen, dass es dann herauskommt, aber ich glaube Missbrauche, also sowohl bei Daten als auch bei Literatur - man muss immer mit einem gewissen Prozentsatz rechnen.	

Interview 4	67:20:00		Nun haben Sie ja schon angesprochen, dass Sie ja auch die Metadaten kontrollieren und eventuell auch Anmerkungen machen. Wie wird denn sonst noch das Vertrauen in die Daten sichergestellt?
Interview 4		Also die oberste Regel ist, es müssen alle Metainformationen ausreichend vorhanden um, die für eine Nachnutzung notwendige Information parat zu haben. Also es ist nicht wichtig, dass wir jetzt, sage ich mal, wenn ich - ich bringe dann immer das Beispiel von Temperaturmessungen.	
Interview 4	68:00:00	Ich kann Temperatur messen mit sage mit hochkompliziertes Termometer, super Apparatur misst irgendwie was weiß ich fünf Stellen auf den Komma gnwu. Ich kann das aber auch mit dem Finger machen. Wenn meine Applikation so aussieht, dass meine, sage ich mal jetzt, unterschiedliche Bearbeiter an unterschiedlichen Stellen irgemdwie mal einen Finger ins Wasser gehalten haben und sagen: oh das ist aber kalt oder sagen oh das ist aber warm. Dann habe ich vielleicht eine Applikation, wo diese Genauigkeit ausreicht. Haben wir natülich immer Fragen: Ok, aws bezeichnet der eine als warm, was bezeichnet der andere als kalt und so. Da ist ein riesen großen Fehler drauf.	
Interview 4	68:45:00	(lachen) Aber im statistischen Sinne kann man damit rechnen. Und wenn man das transparent macht, also wenn man nicht dem das so übersetzt, wenn jemand einen Finger reingehalten und hat dann hingeschrieben 15 Grad - und ich kann das nicht unterscheiden, ob es / mit welcher Methode das jetzt gemacht worden ist. Deswegen können das auch zehn Grad sein oder 20 Grad sein. Dann sind die Daten im Prinzipo nicht brauchbar.	
Interview 4	69:15:00	Wenn ich hinschreibe: ich habe es mit einem Finger gemacht, dann kann er es sich überlegen, ob er das für seine Applikation gebrauchen kann oder nicht.	
Interview 4			Nun haben Sie auch schon gesagt, dass Sie sich keine Sorgen darüber machen müssen (--) Nutzer von (platform) zu generieren. Wie ist es denn mit den Kanaälen auf denen Sie dann aktiv sind? Worüber kann man Sie dann antreffen?
Interview 4	69:50:00	Wir sind wie gesagt international in vielen, vielen Portalen sichtbar. Wir sind sichtbar in der Literatur. Wir haben also in den 0er Jahren angefangen mit sehr breitflächigen Kooperationen mit Wissenschaftsverlagen und so zu starten. Weiß nicht, kennen Sie Scholix?	
Interview 4	70:00:00		Scholix?
Interview 4	70:10:00	Ja.	
Interview 4			(überlegen)
Interview 4		Scholix. Scholix ist auch unsere Erfindung. (---) Schicke ich Ihnen den Link in den Chat rein.	
Interview 4			Ja, gerne.
Interview 4	70:35:00	Scholix.	

Interview 4	70:40:00	Das ist so, es werden ja die, es werden ja die wenigstens Datensätze werden in den Referenzlisten der Paper wirklich referenziert. Und wir halten aber so beim guten Teil der Daten, also ich glaube 90% oder so, bei den wir Literaturangaben auch mit in den Metadaten mit drin haben. Und diese sogenannte Linking Information, die geben wir auch weiter an DataCite. Und über Datacite - im Wesentlichen - wird Scholix dann befüllt.	
Interview 4	71:15:00	Scholix ist sozusagen eine Verbindung aus Crossref-Daten, die Lteraturhaben, DataCite, die Daten haben, pkus eben das, was die Verlage selber noch haben. Also es ist so ein Kongolummerat. Das kannst du mit mehr eigentlich. Das kannst du mit OpenAir - sagt Ihnen OpenAir was?	
Interview 4			Ja.
Interview 4	71:45:00	(unv.) es ist eine Gruppe in Italien, die wir auch seit vielen kollaborieren, die haben das dann übernommen und die pflegen dieses scholix dann.	
Interview 4		Das heißt, die Verlage bedinenen sich da und gucken dann nach: für einene bestimmten Artikel gibt es bestimmte Informationen, Referenzen auf Daten? (journal) macht das ziemlich konsequent. Also das wird alles abgebildet, was an Daten eben über Scholix verfügbar ist und wird dann in Splash Page von dem Artikel mit reingeblendet. Wir haben eine eigene Section bei Data References. Das ist sehr schön gemacht.	
Interview 4	72:24:00	Ja, und das ist auch etwas, was wir von der Strategie her gesehen haben, dass der Kontext mit der Literatur so wichtig ist. Da haben wir in den Mutterjahren sehr viel Energie reingesteckt. Wir haben erst so bilateralen Verbindungen mit einigen Verlagen - eben (journal) als Hauptpartner in der Richtung. Aber, das skaliert halt nicht, wenn man mehere Datenzentrenten hat und mehrere Verlage und die machen alle bilaterale Verbindungen auf - das ist nicht gut. Das kann man nicht unter / also Scholix ist sozusagen der One-for-all Verbinder. Alle bedienen sich da.	
Interview 4	73:10:00		Danke für den Hinweis. Ich werdemir das in Ruhe mal durchlesen, was da alles drauf stehst.
Interview 4	73:20:00		Kommen wir zu meinen letzten Fragen. Und zwar ist das Besondere bei meinem Thema ja auch, dass es sich um Informationssysteme oder Plattformen in der Wissenschaft handelt. Wie schätzen Sie denn diese Eingliederung in die Wissenschaft ein? Also wie beeinflusst die wissenschaftliche Kultur die Nutzung der Plattform?
Interview 4		Ja, ich möchte mal sagen, die Wissenschaft hinkt hinterher. Wir haben, wir haben / das war eigentlich auch so meine Erwwartungshaltung schon in den Neunziger Jahren, dass wir mehr und mehr so Metaanalysen sehen werden. Wo halt also ein übergeordneter Bedarf an Datan so da ist. Das ist auch schon da, aber auch was wir auch so sehen, dass also diese Gruppe, die dann solche Kompilate erstellen, in ihrem Kreis irgendiwe machen. Die haben dann Post-Docs, die machen dann dies und das und so.	

Interview 4	73:50:00	Und über Jahre werden dann Datensammlungen angelegt, die dann für den Scope dieser jeweiligen Arbeitsgruppe dann eine gewisse Grundlage darstellen. Und das ist etwas, das versuchen wir natürlich zu beeinflussen, aber es ist schwierig. Ehrlich gesagt ist es einfach schwieirig.	
Interview 4		Weil, das ist so wie -man muss sich das so vorstellen wie in der Gesamtbevölkerung auch. Ein großer Teil der Gesamtbevölkerung ist also was die Entwicklung mit dem Internet und überhaupt mit Daten, Informationen angeht, ist also mehr oder weniger abgehängt. Die durchblicken auch die vielen Mechanismen gar nicht. Also das, was einige vielleicht noch beherrschen ist dann Whatsapp, aber Gefahren sehen sie da auch nicht.	
Interview 4	74:45:00	Sage ich jetzt einfach mal so. Das ist so Standardwissen, was aber letzendlich auch nicht so richtig hilfreich ist. Und das Potential wird noch nicht vollkommen erkannt. Das ist so (-) ich sage immer: sollten wir das also schaffen, dass wir so (-) wir haben jetzt in der NFDI haben wir eine ganze Reihe von UseCases, die sich dann auch da commitet haben. Das ist sowohl in der NFDI for Earth ist das der Fall, also auch in NFDI for biodiversity. Das ist sozusagen der Follow up für (platform).	
Interview 4	76:06:00	Da sehen wir das schon, dass die auch ihre Arbeitsweise auf Cloudbasierte, collaboratives Arbeiten umstellen wollen, Weil die eben auch den Vorteil sehen. Wir sehen natürlich auch NFDI jetzt auf einer Zeitschiene von zehn Jahren. Das gibt uns Zeit das auch zu machen.	
Interview 4		Ich schätze mal so, wenn die ersten Gruppen anfangen über die effizientere Plattform bessere Wissenschaft zu machen oder wissenschaft die mehr Impact erzeugt, dann ist das ein selbstläufer. Dann wird sich das beschleunigen. Aber so lange wie gesagt überwiegend auch konventionell gearbeitet wird - also die Leute fahren raus, erheben dann Ihre Daten, dann schreiben sie ihre Paper, sitzen dann im Labor und schreiben sie ihre Paper oder so.	
Interview 4	76:27:00	Solange das überwiegend der Fall ist, wenn man damit eben halt eben auch den IMpact von den Instituten, Universitäten und sonst was belegt, solange eben halt wird, wird das noch so ein bisschen dümpeln. Sie kennen sich ja auch im Aktiengeschäft.	
Interview 4	77:25:00	Diese Start-Ups, die start-ups brauchen manchmal 5 Jahre, 10 Jahre bevor dann es richtig losgeht, weil genau diese Umstände sind dann plötzlihc so, dass man dann sagt: hey die lifern ja genau das, was wir jetzt brauchen.	
Interview 4			Und die haben dann schon vorgedacht.
Interview 4		Ja, so ist das.	
Interview 4	77:51:00		Gut. Dann noch zum allerschluss. Jetzt haben wir ja schon ganz ganz viele unterschiedliche Themen angesprochen. Und deswegen, wenn sie jetzt nochmal zurück denken, was wir alles angesprochen haben: so von dem Aufbau, von der Datennutzung, -aufbereitung, -qualitätssicherung, von den ganzen Netzwerken und so weiter. Wovon hängt auch in Zukunft noch der Erfolg von der, von (platform) ab?

Interview 4	78:23:00	Also ich könnte es nicht sagen. Also. Das ist sicher, das ist sicher auch eine, also, eben halt wenn man jejtzt groß und größer wird, also, das ist sicher auch eine gewisse Politikentscheidung da. Wir haben in dem Strategiepapier haben wir natürlich auch noch mal so überlegt, wo stehen wir denn eigentlich im Vergleich zu anderen irgendwie da. Und was sind so die Threats (unv.), maturity threats und daa und so. Wir haben da so eine Analyse da gemacht oder zumindest versucht.	
Interview 4	79:10:00	Und es ist natürlich ein Threat, wenn wir sagen wir jetzt mal google auf den Plan kommt und sagt: hey, wir machen das jetzt. Und die setzen die dann irgendwie zweihundert Entwickler daran und kaufen sich groß ein irgendwie in den wissenschaflichen Instituten mit tausenden Projekten.	
Interview 4		Dann können die sicher, also dann machen die sicher vieles platt, was da mühsam über Jahre gezüchtet wurden ist. Und das ist sicher eine Gefahr. Also die daraus entsteht. Also, so einfache Institutional Repositories, wenn ich jetzt google wäre oder so, wenn da / ich meine die sehen den Wert dann nicht, weil es diesen wert einfach nicht so richtig gibt. Aber ich meine, die können das alles platt machen da also - ganz leicht. Da gibt es dann nur politische Hemmnisse.	
Interview 4		Wo dann die Europäer vielleicht sagen: ne wir wollen das aber nicht alles bei google haben oder so. Ansonsten gibt es da keine Scharm mehr. Und mit sowas muss man immer rechnen, dass dann plötzlich irgendwelche big plaer aufkommen, die dann mit einer ganz tollen Geschichte da auffahren.	
Interview 4	80:00:00	Nur als kleines Beispiel. Google hat ja diese Data Search in (year/number) auf den Plan gebracht. Und wir waren da auch so dabei, wir haben also mit den Leuten hier in Deutschland haben wir also regelmäßig dann auch kommuniziert, weil wir auch lange schon eine google site map haben da und dass war für die dannauch relativ leicht, also (platform) da einzubingen.	
Interview 4	81:00:00	Die haben einfach gesagt: OK, also sie sind ja auch die Erfinder - so mehr oder weniger von diesem Metadatenstandard Schema.org - sagt Ihnen das was? Schema.org?	
Interview 4		Die haben enfach gesagt, wir machen schema.org und wir sind google. Und dann könnt ihr euch verbiegen. Und wenn ihr nicht schemaorg könnt, dann seid ihr nicht dabei. Und dann steht natürlich also ein ziemliches Momentum - wir konnten auch schon vorher Schema.org, also für uns ist das nie ein Problem gewesen.	
Interview 4		Wir haben da auch eine entsprechende Infrastruktur mit der wir alle Schemata irgendwie bedienen können. Nur Einrichtungen, die das nicht können oder so, die verbiegen sich schonmal eher als wenn man jetzt mit / selbst geo, geoss - sagt ihnen was? Also die Group of Earths Oberservation. Das ist auch eine große ministrielle auf dann Ministriellen Ebenen ist das dann ein zusammenschluss da.	

Interview 4	82:00:00	Selbst die, wie gesagt, haben diese Vorgabe mit diesem ISO, aber wenn die Leute nicht wollen, dann machen sie das nicht und dann geht ihnen das sonst wo vorbei da. Und kleinere Portalbetreiber bieten dann häufig dann die Möglichkeit: ja, ihr könnt dies machen und das machen und jenes machen und das könnt ihr auch noch machen - und das skaliert dann auch nicht. Das ist einfach viel zu aufwendig in der Unterhaltung. Und so arbeitet dann halt so ein großer Player, der dann einfach so attraktiv ist: ja, wir wollen dabei sein, deswegen machen wir das.	
Interview 4	82:42:00	Und so könnte das dann mit Daten auch funktionieren, wenn die dann eine Cloud Plattform zur Verfügung stellen und sagen: wir laden eure Daten so ein - die haben ja auch KI, gute KI Komponenten - wir laden eure Daten so ein, ihr könnt das also abnicken, das wird voll integriert und so. DAnn haben die sozusagen den (-) Durchbruch damit. Also die haben dann nicht, die haben dann noch nicht (platform) geschluckt, das ist aber / sie können tatsächlich Teile (-) können Sie, also, übernehmen, sage ich mal so.	
Interview 4	83:20:00	Und dann sind es eigentlich nur noch kleine Schritt, also dann zu sagen: ja, Daten könnt ihr auch bei uns direkt abgegebn. Ihr kriegt auch eine DOI, ist auch zitierfähig und so weiter. Wir haben auch Domänenexperten, wir haben ein Reviewsystem, wir haben Peer Reviewer Weltweit, die wollen alle bei uns mitmachen - die kriegen alle 1000 Euro mehr als bei (journal) oder irgendwie sowas (-)	
Interview 4	83:45:00		Und wovon hängt sonst der Erfolg noch ab? Fällt ihnen noch was ein?
Interview 4		Ja, dass wir vielleicht (lachen) jetzt nicht den Weltkrieg irgendwie anzetteln, uns die Klimaerwärmung nicht so um die Ohren schlägt, dass wir so nicht mehr (-) nicht mehr Handlungsfähig sind - was durchaus also sein kann. Das ist zwar sehr weit hergeholt, aber es ist / das hat man schon gesehen.	
Interview 4	84:00:00	Man hat schon Pferde kotzen sehen. Neulich noch einen beitrag irgendwie gelesen, dass unter Umstönden mit dem zwei Grad Ziel soweit drüber hinausschießen, dass die (-) die CO2 Akkumulation, dass die irreversible wird, dass es unter Umstönden wie auf dem MArs hier geben wird. Das sind dann so Zeitlinien - sind wir halt in 1000 Jahren sind wir halt weg vom Fenster.	
Interview 4	85:00:00	Also, der Erfolg von (platform) jetzt in den nächsten zehn Jahren sehe ich überhaupt keine Probleme. Aber langfristig, über zehn Jahre hinaus. (unv.) Müssen wir sehen.	
Interview 4			Ja, schön, Danke.
Interview 5	Time	Interviewee 5	Interviewer
Interview 5			Kurze Begrüßung
Interview 5			Datenschutz
Interview 5			Kurze Vorstellung
Interview 5	00:57		Meine Einstiegsfrage ist gleich, um Sie ein bisschen besser kennen zu lernen, wie ist so Ihr Hintergrund und was machen Sie jetzt. Also Ihre Tätigkeiten gerade.
Interview 5		(personalied)	

Interview 5	02:10		Wie ist denn die Geschichte hinter (platform)?
Interview 5		Oh, die ist lang. Also (platform) existiert seit 25 Jahren und wurde ursprünglich von (personen,insitut) und der Universität (town) etabliert. Die Idee war dass man Umweltsdaten publizieren kann also nachhaltig aufbewahren kann, publizieren kann und dann hat es ja früh angefangen mit den DOIs, Digital Object Identifiyer, sagt Ihnen wahrscheinlich was, oder?	
Interview 5		Also zitierbarkeit von Daten einzuführen udn es ist dann in den letzten Jahren relativ rapide gewachsen. Naja Sie wissen ja selber in den letzten Jahren hat sich sehr viel im Bereich Forschungsdatenmanagement getan. Die Bedeutung von Forschungsdatenmanagement und Data Science ist natürlich imens gewachsen.	
Interview 5		ich selber war vier Jahre im Rat für Informationsinfrastrukturen und dann haben wir dort die sogennante NFDI die Nationale Forschungsdateninfrastruktur mit geboren und jetzt bin ich, habe ich sozusagen Seiten gewechselt und habe selber ein Konsortium angemeldet für, ja (unv. Biodiversitäts? 3:16)daten inder eben (platform) und ja, vor allem (platform) auch eine große Rolle spielt.	
Interview 5		(platform) ist gewachsen von, ich sage jetzt mal einer Garagenfirma, also im Startup Bereich würde man sagen einer Garagenfirma, die Idee von zwei Leuten, die das dann mithilfe von einem Informatiker letztentlich angefangen haben, eine Datenbank und letztendlich ein Webinterface zu programmieren zu einem jetzt, wie viel sind wir jetzt ungefähr in (platform), ja ungefähr 35, also wenn man den AG-Teil dazu zählt sind es ungefähr so 30 bis 35 Leute, die im Moment im Bereich der Datenbank / oder im Bereich von (platform) arbeiten, teilweise im Backbone um eben die Daten / reine Datenaufbewahrung letztendlich zu sichern.	
Interview 5		Das wird am (unv. Abika 3:59) gemacht und eben mehr im Frontend, sage ich mal so, mehr im Web-Interface aber auch NGU-Projekten viele in verschiedensten Proojekten. National, International. Um natürlich auch die Finanzierung zu sichern, da ist natürlich immer ein großes Problem, wie man sowas über die Jahre hinweg sichern kann, im Universitärenumfeld.	
Interview 5	04:15		Und wie wird denn die Finanzierung gesichert?
Interview 5		Also, bisher war es so dass / die Forschungslandschaft in Deutschland sagt indes sowas, also was macht (institut), was macht (institut), was macht (institut), ja, also was macht (institut), also was machen die Unis. (town) (-)	
Interview 5	04:36		Die sind mir alle ein Begriff, ja.

Interview 5	Ja genau. Grob / Also grob kann man sagen Max-Panck ist für Grundlagenforschung zuständig (-) Leibnitz ist eine Bund-Länder Finanzierung (-) (institut) ist eine hauptsächlich Bundesfinanzierung, die Universitäten finanzieren sich im großen Teil aus landesmitteln, wie das Land (town) zum Beispiel und natürlich aus Drittmittelförderung. (platform) ist sozusagen inbetween, weil es im Moment zu Teilen aus der (institut) Grundfinanzierung finanziert wird, sozusagen allerdings erst mit einer Nachhaltigkeit seitdem ich jetztendlich da bin, das hat aber nichts mit mir zutun sondern, das hat damit zutun, dass Michael Diepenbroek in Rente geht, man hat einen Nachfolger gesucht und (--) man sich der Bedeutung von Datenmanagemnt bewusster geworden ist sund somit (institut) jetzt auch nochmal zusätzlich sozusagen Gelder in Form von festen Stellen auf die Seite von (institut) gepackt hat.	
Interview 5	Auf der anderen Seite hat auch die uni (town) sich bewegt und hat auch noch ein paar Stellen dazu gepackt und das ist eine Grundfianzierung von ich sag mal grob fünf, sechs Leuten da ist und der Rest wird nachwievor über Drittmittel finanziert, also (unv. 5:47)Projekte, nationale Projekte, BMWF, DFG-Projekte, wo eben sozusagen ,die (-)die Entwicklungsleistung , aber auch im Prinzip ein Betrieb, im Sinne von Forschungsdaten, Repositorium, Aufnahme von Daten, Kommulation von Daten Zurverfügungstellen von daten eben, gemacht wird.	
Interview 5	Also es ist eine Misch- / Also man muss sich sowas vorstellen(-) das nennt man Mischfinanzierungen, die wenn man Glück hat eine gwisse Grundfinanzierung über eine institutionelle Förderung bekommen, aber die Hauptlast, auch in den letzten Jahren muss man sagen, war eigentlich / bedingt durch die Drittmittelförderung gemacht. Deswegen auch die / (-) ja, die Nachhaltigkeit aller Systeme, auch meines eigenen (platform)-Systems zum Beispiel, was ja auch eine Datenbank eben ist, war lange Zeit oder ist im Moment noch nicht ganz gesichert im Moment ist deswegen wird die von Leibnitz übernommen oder soll von Leibnitz übernommen werden.	
Interview 5	Weil Leibnitz eben auch als Institution / also in Deutschland / Die Aussage ist in Deutschland können sie eigentlich kaum nachhaltige Infrastrukturen an Universitäten betreiben, dafür haben die Universitäten eigentlich von Haus aus kein Mandat (-) da gibt es eine spezielle Förderung Paragraph 91 b der ist jetzt vor ein paar Jahren erst aufgehoben worden, damit könnte man sowas machen, wird aber selten umgesetztdeswegen, wenn Sie nachhaltige Infrastruktur betreiben wollen müssen Sie an eine der Institute gehen: Leibnitz, Hemholtz, das sind jetzt so die zwei Hauptakteure in dem Bereich, weil sie beide Infrastrukturförderung machen, (institut) nur wenn Sie im Bereich von industrienaher Forschung sind. (-) Ja, (institut) gar nicht, weil (institut) macht Grundlagenforschung und generell macht keine Infrastrukturforschung, also ich war (years) Jahre (institut) (personalised)	

Interview 5	07:44		Was ist denn jetzt das Kernangebot von (platform)?
Interview 5		Also das Kernangebot ist eben die Ablage von / Ablage, das klingt ein bisschen spektierlich (-) Aufnahme von Forschungsdaten aus der biologischen Umwelt, den Umweltwissenschaften das geht sehr breit aber Geo- / also geologische Wissenschaften, biologische Wissenschaften, Atmosphärenforschung	
Interview 5		Was immer Hauptangebot and er Stelle ist, ist die sogenannte DatenKuration, d.h. es werden eben wenn einen Submitter / wenn jemand kommt mit seinen Daten, dann wird er nicht einfach nur die Daten abgelegt und eine DOI drangeklebt und dann nach dem Motto: "Hier geht es weiter", sondern man versucht / deswegen haben wir (-) eins, zwei, drei vier, im Moment (-) sechs Kuratoren , die praktisch die Daten annhemen und dann mit dem Submitter, mit jemjenigen der die Daten dann ablegen möchte zusammen die Metadatenstandards durch geht, die Daten in entsprechende Formate bringt, aufarbeitet, so dass sie praktisch die Nachnutzbarkeit der Daten gesichert wird.	
Interview 5		Also FAIR ist Ihnen vermutlich ja auch ein Begriff, ist ja auch so ein riesen Thema findable, accessible ist immer nicht so kompliziert, es reicht im prinzip, wenn man sie in einen (unv. Drive-Share? 9:00) oder (unv. 9:01) oder irgendeinem Standard Repositorium ablegt, aber interoperable, reusable ist dann das große Thema, da müssen dann eben die entsprechenden Standards eingehalten werden. Da müssen die Daten in die entsprechenden Standardformate überführt werden.	
Interview 5		Daten, Metadaten vorallen Dingen, damit die Leute nacher eben auch wieder was anfangen könne damit. Und das ist eben sozusagen das Angebot von (platform). Auch die Besonderheit ist ebene eine enge Verzahnung des sogenanten klassischen Daten / also klassischen Publikationsschiene, wie Ihnen sicher auch bewusst ist, das Wissenschaftssystem funktioniert ja zum Beispiel im Bereich der Lifesience im großen und Ganzen, aber auch Physik und Mathematik teilweise, über die Publikationen. Also das heißt, wenn Sie in der Wissenschaft was werden wollen dann müssen Sie irgendwann mal ein Paper schreiben, möglichst in hochrangigen Journalen, die irgendwo einen H-Index haben / (korrigiert sich) einen Impact-Factor haben, jenseits von 10 oder so und wenn Sie davon genügend geschrieben haben, dann haben Sie auch gute Chnacen Ihre nächste Doktor- / ihre nächste (Postdock? 10:00) oder letztendlich Ihre Professur zu bekommen und weil das sozusagen das Credit-System der Wissenschaft ist hat dagegen die Datenpublikation zumindest in den Köpfen vieler Leute / vieler Wissenschaften noch nicht den selben Stellenwert hat, hat (platform) sich sehr früh entschieden, dass man sie beide zusammenbringt.	

Interview 5		Dass man im prinzip die klassische Publikationsschiene einen Link auf die Datenpublikation setzt. Und dass die Leute im Prinzip ihre normale Paper schreiben können aber möglichst dazu auch eine DOI bekommen, wo sie ihre Daten publizieren können. Inzwischen sind wir ein bisschen von dem prinzip / versuchen wir gerade eine erzieherische Maßnahme durchzuführen, dass man sagt die Datenpublikation steht als sich selbst, sozusagen, ist jetzt nicht mehr ein Anhängsel an die klassische Publikation, denn die Datenpublikation ist es wert für sich selber zu stehen und das wird auch eben durch den ganzen Hype / oder den ganzen Bewusstsein um das Thema Daten, Forschungsdatenmanagement im Moment gefördert. Bei der DFG zum Beispiel schon verkündet nat, dass so eine Datenpublikation durchaus gleichwertig zu einer klassischen Publikation im Rahmen eines (CVs? 11:01) im Rahmen eines Antrags zulässt.	
Interview 5		Das heißt den Stellenwert der Datenpublikation versucht man gerade deutlich nach oben u treiben, sodass eben eine Datenpublikation auch eine eigene Wertschätzung hat. Es gibt ja inzwischen auch eine Menge Darajournals, die sich darauf spezialisiert haben einfach nur Daten zu publizieren. Naturedatascience ist eins zum Thema, da ist auch viel (-) ich sage mal Scharlatanerie im Umfeld weil natürlich auch die klassichen Publizierer erkannt haben, das ist ein neues Geschäftsmodell sein kann und desween (lacht) die natürlich aus diesen Zug aufspringen und im Prinzip jetzt sozusagen Geld verlangen, dass man seine Daten publiziert.	
Interview 5		(platform) macht das umsonst. Also kostenlos, nicht umsonst. Kostenlos, weil wir eben im Moment genügend Grundfinanzierung haben, weil das auch unseren / also vor allen Dingen Gründern, aucb meiner persönlichen Einstellung widerspricht Datensätze zu verkaufen oder überhaupt	
Interview 5	12:05	diesen Service zu verkaufen. Das kann ich jetzt so locker sagen, weil natürlich im Moment, wenn das so aussieht als wenn die Zukunft von (platform) gesichert ist (-) das muss man immer differenziert betrachten. Vor ein paar Jahren glaube ich , hat durchaus das Thema Geschäftsmodell und Verkauf auch mit im Raum gestanden. Ich selber habe das meinen Datensätzen, Datenbank auch schonaml angedacht. Wir haben sogar eine Firma ausgegründet die für den kommerzielen Bereich tatschlich Datensätze auch angeboten hat, weil wir irgendwann das überlebend er Datenbanken sonst nicht sichern könne, also wie gesagt, man muss es ei bisschen differenziert betrachten. (-) Mit vollen Hosen ist gut stinken, wenn man das Geld jetzt nicht hat dann, ja (-) "who keeps them running"? Wer bezahlt das Ganze am Schluss?	
Interview 5	12:42		Nun handelt meine (work) ja von Erfolgsfaktoren, was ist für Sie denn Erfolg oder eine erfolgreiche Platform?

Interview 5		Eine erfolgreiche Platform? Also für mich ist persönliche eine erfolgreiche Platform, wenn sie angenommen wird, wenn Nutzer sie benutzen. Wenn sie sozusagen in der Community angekommen ist, wenn man entsprechende Nutzerzahlen hat, respektive, wenn man auch tatsächlich (s) so wie (platform) oder auch (platform) die Nutzer aktiv nachfragen. Wenn sie Daten veröffentlichen wollen sozusagen in die / in den Dialog mit uns gehen und dann eben auch entsprechend ihre Daten bei uns ablegen und nicht dazu gezwungen werden sondenr letztentlich / gut Zwänge gibt es immer klar, die Journals verlangen das inzwischne auch die Förderer verlangen das, aber dass sie sozusagen hier von sich aus diese Datenbank auch nutzen und auch kommen und das machen.	
Interview 5		Das ist natürlich ein längerer Weg dahin, also das ist wie in jeder kommerzielen Bereich, wenn Sie ein neuens Produkt haben, dann haben sie nicht unbedingt gleich einen Pull-market, dass die Leute auch kommen und dann sozusagen aus der Hand reißen, außer sie verkaufen keine Ahnung, warme Semmel im Winter oder Temptaschentücher im Herbst oder die Arbeitszeit (lacht).	
Interview 5		aber es ist so Sie müssen das Produkt wirklich in den Markt bringen und das ist jetzt halt im Bereich von (platform) und ähnliches gilt für (platform) sehr aktiv durch die Gründer sozusagen gemacht worden, da hat man sich eben mit dne Nutzern sehr viel zusammengesetzt hat und überlegt hat wie man das so gestalten kann, dass die Leute auch mehr davon haben das ist (-) Also für mich der Erfolg ist / Zufriedene Nutzer um es mal auf den Punkt zu bringen.	
Interview 5	14:37		Was ist denn die Vision für (platform)?
Interview 5		Also, da wird im Moment sehr viel darüber diskutiert auch mit meiner Berufung letztendlich (-) neue Besen kehren ja gut muss man ja was neues machen das heißt (platform) ist sehr sehr stark im Bereich Datenablage im Moment das heißt / deshalb habe ich auch vorhin die beiden Worte Forschungsadatenmanagement und Datascience zusammen genannt also wir sind ein sehr gutes Repositorium, wir sind zertifiziert inzwischen wir haben Gold trust / (cort trust ziel Zertifikat? 15:07) also Nachhaltigkeit das ist alles wunderbar, ist auch weltweit anerkannt, aber die Leute wollen natürlich ganz gerne die Daten nachlesen und in den Bereich Datascience sozusagen gehen, das ist sicherlich etwas wo wir noch deutlich besser werden können, das heißt also, dass die Daten sozusagen noch besser aufbereitet werden, das auch zum Beispiel entsprechende Tools und Techniken auf der Website angeboten werden, sodass die Leute eben die Daten programmatisch einfacher aus der Datenbank holen könne um sie dann mit ihren Daten zu verschneiden, um ihre Fragestellungen zu beantworten.	

Interview 5	Das heißt also (Benachmark? 15:52) der letzten knapp 25 Jahre mehr auf dem Ingest, also dem was sozusagen in die Datenbank reinfließt, wie das aufbereitet werdne kann, wie das sozusagen entsprechend strukturiert werdne kann. Jetzt müssen wir mehr Schwerpunkte auf den Bereich der Datascience oder den Datasciencesupport, nennen wir es mal / wir machen ja selber keine Datasience, es geht nicht darum, dass wir die Daten auswerten, sondern es geht darum, dass andere / das wir die Möglichkeit schaffen, dass andere diese Daten nutzen können, um ihre Wissenschaft damit zu machen.	
Interview 5	Also enabeling science, also die sollen bessere Wissenschaft damit machen. Das ist eins der direkten Ziele oder Visionen wo es jetzt hingehen soll, also wir reden in diesem Moment gerade auch mit dne Nutzern,die die Daten nachnutzen wollen, auf der anderen Seite sind wir in zwei großen nationalen Forschungsdateninfrastrukturen aktiv mit (platform), das ist NFDI4Earth und NFDI4BioDiversity, NFDI sagt Ihnen etwas, oder? Nationale orschungsadateninfrastruktur. (Nehme ich an? 16:53) das ist ja ein großes Ding (-) wir haben zwei Anträge eingereicht, also was heißt zwei eingereicht.	
Interview 5	Eins kommt nicht von mir, Earth wurde von Lars Bernard an der TU Dresden eingereicht, ich habe eine für BioDiversity eingereicht, beides mit runf 50 Partnern Coapplicants, participants und in beiden spielt (platform) eine relativ große Rolle, wir stellen uns da vor eine sogenannte Research Data Commons zu bauen für die ganze NFDI, das heißt also ein zentraler Punkt wo die ganzen Daten zusammenfließen können und auch wieder zum Nutzer rausfließen / zu den Nutzern rausfließen können.	
Interview 5	Das ist eigentlich so die Idee, jetzt nicht nur (platform)s sondern eigentlich (platform) in die NFDI zu integrieren und das Ganze sozusagen dann nochmal ein Level größer zu machen, um eben verschiedene Communities mit diesen vielen Domains zu bedienen. Wir sind auch end verbandelt mit NFDI for Health, klingt jetzt erstmal etwas esotherisch, was macht denn jetzt hier die Healthforschung mit drin, aber ist klar, Umwelt und / also Ihr Lebensraum wird von der Umwelt bestimmt und natürlich wird vieles Ihrer Wohlfühlfaktoren auch damit bestimmt, wie sich sozusagen Ihre Umwelt entwickelt, somit sind Umweltdaten durchaus bedeutend, einige der Krankheiten sind auch / also nehmen wir mal Allergien und solche Sachen wird natürlich ganz stark auch im Klimawandel begünstigt im Moment, weil eben trockene Perioden da sind, weil eben Sporen zum Beispiel im Frühjahr da sind, in größeren Mengen da sind.	
Interview 5	Wenn Leute da allergisch reagieren / das hängt ja doch am Ende des Tages alles miteinander zusammen, da werden Sie Artensterben und Klimawandel haben, dann wird es auch eine Auswirkung auf die Umwelt haben das heißt / (-) also auf die Gesundheit haben, jedes Einzelnen, deswegen sehen wir da eine durchaus eine enge Zusammenspiel zwischen den verschiedenen NFDIs an der Stelle.	

Interview 5		Plus natürlich vielen anderen NFDIs, die im Moment noch sich aufgesetllt haben und die wir auch zumindest kennen, aber jetzt noch nicht so eng zusammenarbeiten. (Zeitmarke: 18:50) Also die Vision ist schon das Ganze mal noch einLevel nach Oben zu schieben, wobei wir beii unserem (Leisen? 18:56) bleiben, bei unseren Datenbank, aber das Ganze in einen größeren Kontext einzubetten. Ich hoffe wir werden gefördert, wir werden sehen. Ich weiß es nicht, ist noch nicht raus.	
Interview 5	18:50	Also die Vision ist schon das Ganze mal noch einLevel nach Oben zu schieben, wobei wir beii unserem (Leisen? 18:56) bleiben, bei unseren Datenbank, aber das Ganze in einen größeren Kontext einzubetten. Ich hoffe wir werden gefördert, wir werden sehen. Ich weiß es nicht, ist noch nicht raus.	
Interview 5	19:08		Wie ist denn der organisatorische Aufbau hinter der Plattform?
Interview 5		(Lacht) Ja, (-) das ist eine gute Frage. (--) Also es gibt (-) grob gesagt gibt es einen (-) technischen Bereich / also meinen Sie jetzt rein von der Hirarchie her oder von der / also von (---) also man kann jetzt in verschiedenen Bereichen / also man kann / es gibt eine reine Hirarchie, es gibt sozusagen mich als Gesamtleiter, darunter gibt es Gruppenleiter, jetzt habe ich gerade eine Gruppenleitering (platform) eingestellt hier, dann gibt es einen Gruppenleiter der jetzt auf der AG-Seite für die Technik zuständig / also für die Softwareentwicklung zuständig ist.	
Interview 5		Dann gibt es / da muss man leider differenzieren auf der einen (unv. 19:56)-Seite gibt es die Hirarchie in dem Sinne nicht da bin ich Gesamtleiter, da möchte ich noch einen Gruppenleiter einstellen (lacht) der ist noch nicht da (-) dort ahben sie eher in projekten gearbeitet das heißt da ist die Hirarchie auch, flach. Die einzelnen Leute haben einfach Projektarbeit gleistet und waren sozusagen in ihren Projekten dann zuständig für Arbeitspakete, was alles ist.	
Interview 5		Ja am AW ist es eher strikter organisiert da haben wir einen Bereich Systems, der jetzt mit einem Bereichsleiter zuständig ist für die ganze technische Infrastuktur, das heißt also Storage, Internetzugang, Website /also technische Backbone liefern. Dann eben / das fällt jetzt nicht in meinen Bereich, in meinen Bereich Data fällt die Softwareentwicklung, Softwareengeneering, Editorialsystem, alles was an Daten softwaretechnisch sozusagen verarbeitet werden muss und jetzt eben neu (platform)-Gruppenleitung, Kuration. Und wie gesagt auf der (Marun-Seite? 20:53) nicht so klar strukturiert, aber da gibt es auch Kurratoren und der Rest ist / aber wie gesagt bis vor (-) ich sage mal bis vor einem Jahr waren die meisten / hatten alle keine festen Stellen dort das heißt sie haben an den Projekten gerabeitet.	

Interview 5		Sogesehen hat sich die Frage der Strukturhirarchie gar nicht so ergeben, man hat einfach in Projekten geareitet und das Gesamtprojekt war (platform). Also so generell gesehen haben Sie einen technischen Bereich, der sich um Storage (-), IT, alles was damit zutun ist kümmert. Sie haben einen (-), ja wie soll ich sagen einen Software engeneeringbereich, alles was sich sizusagen um die Software kümmert und Sie haben (--) also PR, Marketing (-) Jaein, das wurde in erster Linie durch die Projekte gemacht oder im Rahmen von projekten gemacht.	
Interview 5		Und dann natürlich Frontend-Entwicklung, was eben im großen und Ganzen Softwareentwicklung ist aber doch mehr Schwerpunkt auf die Softwareentwicklung für den Nutzer und ein großes Thema ist eben das Korratorsystem, das eben als Softwareentwicklung auch dahinter ist, also Software. Dann hatten wir eben letztendlich, ja was Sie sonst brauchen, eben Marketing und natürlich Aquise , vorallendingen auf der Seite des (Marons? 22:13) Projektaquise. Sie müssen ja ständig Geld reinschaffen, nicht?	
Interview 5	22:20		Welche Kompetenzen besitzen denn dann die 30 bis 35 Mitarbeiter?
Interview 5		(-) Moment mal kurz hier hat sich eben ein komisches Fenster gemeldet, das muss ich kurz wegklicken. So. (--) Also wir sind (---) Also es gibt Hardcoreinformatiker es gibt (--) also die richtig Softwareentwicklung machen, also (unv. Ich kann es einfach nicht richtig verstehen 22:46-22:50) die Frontend und Backend können, wir haben aber inzwischen auch (unv. 22:54)-Designer drin, die ist jetzt nicht hundertprozenig (platform), aber macht auch da mit.	
Interview 5		Wir haben das geht dan jetzt Richtung Design und was sowas ist. Wir haben natürlich den ganzen großen Bereich der Biologen und Geologen, die da drin sind, dir vor allem im Korratorischenbereich arbeiten, die eben die Daten anngehmen / natürlich wissen müssen um was geht es bei den Daten, um dann letztendlich entsprechende Formate und eben alles amchne müssen, das sind dann eben Biologen und Geologen, die da drin sind.	
Interview 5		Ich glaube Michael selber hat mal Physik studiert oder Informatik oder sowas. Wie gesagt ich war mal Biologe bin dann in der Bioinformatik gelandet und jetzt wieder in dem Bereich zurückgekommen oder wieder zurück / irgendwie soetwas. Wir haben Leute drin, die von der Ausbildung her eher im Bereich Biologie oder (-) Geologie sind, aber eher im Management tätig sind, also Projektmanagement tätig sind, weil sie müssen ja auch sozusagen, gerade wenn es um die O und NFI-Projekte geht und BMWF-Projekte müssen gemanaget werden.	

Interview 5	Also es ist ein reicher Strauß oder ein bunter Strauß an Expertisen, die da drin sind. Im Prinzip kann man eigentlich / gibt es nichts was wir nicht brauchen können (lacht). Es geht / wir hatten eine Weile auch einen BWLer drin, der sich um Businessmodelle gekümmert hat oder da sich mal überlegt hat / das ist jetzt nicht mehr so prominent mehr / also wenn Sie mich fragen, was wir noch brauchen könnten, wir brauchen noch jemanden der Jura studiert hat, vielelicht noch jemand der BWL studiert hat, also könnte man jetzt wieder mal brauchen.	
Interview 5	Psychologie wäre noch sehr angemessen, weil sowohl für die Gruppnbetreuung, als auch die Nuterbetreuung braucht man glaube ich eher Psychologen (lacht) im Prinzip können Sie alles haben.	
Interview 5	Die rechtlichen Fragen kommen im Moment relativ massiv auf uns zu, Sie wissen ja Datenschutzgrundverordnung ist ja auch bei uns / obwohl wir jetzt nichts direkt / keine personenbezogenen Daten verarbeiten, eigentlich, aber Sie wissen ja jede Websiete muss im Prinzip mit diesem Ganzen terms of use und privacy policy und alles / das haben wir natürlich alles gemacht / machen wir mit einer Rechtsstelle zusammen, aber jetzt gibt es auch viele Themen die zum Beipsiel aktuell / also nochmal so / also (platform) ist im Kern sozusagen jetzt (town) und (town) (? 25:11), aber wir haben noch Außenstellen, wir haben noch Außenstellen in (town), wir haben eine Außenstelle in (country), wir haben eine Außenstelle in (town), (institute) und wir richten gerade eine Außenstelle noch in (town) ein am (Zarem ein? 25:24).	
Interview 5	Und das ist auch so ein Modell, also eine Vison jetzt nicht, aber weil es eher ein technische Sache ist, wir würden sozusagen gerne die Last der Kuration in so einem Front-Office-, Back-Offive- Modell nach Außen verlagern, das heißt / also wenn jetzt Datensätze in größerer Menge von bestimmten Instituten immerwieder gelifert werden, wie zum Beispiel (town) (institute) oder in (town) oder auch in (country) jemand sitzt/ oder wie ich sage jetzt in (Zarem? 25:53) in (town) das sie dort jetzt letzendlich jemanden anstellen / Korrator anstellen der die Daten schomal vor bearbeitet und dann bei uns die Datensätze anliefert, damit wir letztendlich dieses Domainwissen nicht alles hier aufbauen müssen sondern es letztendlich nach Außen verlagern und das heißt also auch von der Expertise wären dann nochmal Korratoren, die spezifisch für diese Domain dann letztendlich sind.	
Interview 5	Wir werden jetzt auch nicht komplett ewig breit werden, wir werden jetzt / ja wobei auch sozialwissenschaftliche Daten inzwischen haben (lacht), das ist so ein Nebenprodukt noch (-) (lacht) das schafft uns eine Menge Kopfzschmerzen, wir waren ja vorhin beim Thema Jura, weil das natürlich personenbezogene Daten sind, das ist (-) Sie wissen vielleichr (town) ist im Bereich Soziale- und Geisteswissenschaften aufgestellt, traditionell, das war szusagen noch so ein bisschen ein Wunsch vom Land (town), dass man sich auch darum kümmert.	

Interview 5		Das ist nicht unser Schwerpunkt, das muss ich sagen und ich bin da nicht so ganz glücklich damit, nicht weil ich sozusagen Geisteswissenschften nicht mag, sondern einfach weil sie eben von der Datenschutzrechtlichengrundlage (-) einen anderen Komplexitätsgrad haben. Also die Daten dürfen zum Beispiel nicht am (unv. 26:59) gelagert werden, weil sie diese Zertifizierung nicht haben, sondern sie liegen dann auf ganz speziellen Servern, die eben entsprechend gesichert sind. Eben weil es personenbezogene Daten sind. Ihre Interviews sind ja auch personenbezogene Daten, gut ist jetzt die Frage, wie weit / ja tatsächlich liegt das schon wieder in einer Grauzone, aber wenn Sie zum Beispiel, wenn es noch um medizinische Daten geht / also medizinische Interviwes / also wenn es um psychologische Daten geht, wo Sie sehr tief Einblick nehemn in die Persönlichkeit von Leuten, dann sind das schon hochgradig brisante personenbezogene Daten, die dürfen natürlich auf gar keinen Fall, irgendwo im internet auftauchen (lacht).	
Interview 5		Und gerade diese ganzen rechtlichen Fragen und Datenschutzrechtlichen Fragen, also, da würde ich mir manchmal Wünschen, dass wir eine Rechtsberatung haben. Was wir jetzt auch haben, wir sind gerade dabei mit einem Rechtsanwaltsbüro hier in (town) Kontakt aufzu- / also haben wir schon gemacht, um uns praktisch da bei diesen sogenanten terms of use und Sachen beraten zu lassen. Also eigentlich brauchen wir alles und wir haben auch ganz viel (lacht).	
Interview 5	28:08		gerade haben Sie schon erzählt, von sagen wir mal unterschiedlichen Bereichen. Haben diese dann auch die Verantrwortlichkeit für den jeweiligen Bereich? Oder wie sind die Verantwortlichkeiten verteilt?
Interview 5		(-) Ja / also ich habe den / als Gesamtleiter habe ich tatsächlich eigentlich die Verantwortung für alle Bereiche (--) also bis auf / technisch gesehen ist so Systems (-) Ja, jetzt ist die Frage der Verantwortlichkeit / letztendlich bin ich für den Betrieb und die Zukunft von (platform) zuständig. Es ist aber so, dass jetzt rein formal, am AWI bin ich weisungsbefugt gegenüber der (platform)gruppenleiterin, gegenüber dem Softwareengeneering-gruppenleiter und gegenüber meinen Leuten.	
Interview 5		Allerdings zum Beispiel gegenüber Systems, das heißt also was die technische Infrastruktur, also Storage, Accounting, alles im Sinne von Nutzerzugang (IDM? 29:10) also revenuemanagement und soweiter, da ist ein Bereichsmanager der ist am Ende wie ich, also ich kann ihm jetzt keine Anweisungen geben in dem Sinne. Ich kann ihn nur bitten (lacht). Das ist vielleicht auch die bessere Variante. Sogesehen wenn er sich jetzt aus irgendwelchen gründen komplett streuben würde und dann müsste ich sozusagen eien Stufe höher gehen damit dem Derektorium hier sagen: "Ja es tut mir leid, der Bereich Systems spurt nicht, wir kriegen unsere Daten nicht abgfelegt" dann würde sich das so auch regeln.	

Interview 5		Aber das kommt nicht vor (lacht). Da sind alle dran interessiert das wir das Ding gemeinsam am Laufen halten, aber rein formal-rechtlich sozusagen, targe ich die Verantwortung, habe aber nicht in dem Sinn die Weisungsbefugnis zum Beispiel jetzt zu sagen, wir kaufen noch ein Petabyte Speicher damit wir die Daten der Sozialwissenschaften / und wir sichern das ab, damit wir die Daten der Sozialwissenschaften bei uns targe könne, das ist / da könnte ich nur darum bitten, aber nichts machen.	
Interview 5	30:16:00		Und wenn jetzt Entscheidungen getroffen werden, wie findet die Entscheidungsfindung statt und wie sind die Entscheidungswege?
Interview 5		Genau, also das hat sich auch relativ dramatisch geändert mit meiner Anwesenheit vorher gab es einen relöativ komplexen Kreis von Leuten der am Schluss irgendwie dazu geführt hat, dass keine Entscheidungen getroffen wurden. Seidem ich da bin hat man das geändert, ich bin / es gibt regelmäßige Treffen mit den Direktoren, das heißt also der zwei Dirketoren des (institut)-Institutes, also die zwei Betreiber, die sozusagen hinter (platform) sstehen, das ist die Universität (town) Slash das Marum, das ist eine Einheit an der Universität (town) mit einem Direktor Miachael Schultz und das AW mit den beiden Direktoren Antje Boetius und Karsten Wurr	
Interview 5		und die Entscheidungen werden jetzt / tägliche Entscheidungen treffe ich selber, also das ist / da gibt es sozusagen Bagatellenentscheidungen sozusagen treffe ich selber, kann im Prinzip den täglichen Betrieb / da muss ich nicht ständig fragen. Aber wenn das strategische Entscheidungen sind zum beispiel ob man Mitglied wird in einem bestimmten (-) Verein um dort DOIs zu kaufen, oder was hatten wir jetzt alles so an Themen.	
Interview 5		Ob man eben zum Beispiel mit dem Zahm zusammen eine zusätzliche Außenstelle aufbaut, also größere Entscheidungen, die vor allem auch nachhaltig sind im Sinne die längerfristig sind, die bereite ich vor gebe sie letztendlich in ein sogenannte sLenkungsgremium, die dann aus den drei Direktoren und mir besteht und dann wird dann in einer gemeinsamen Sitzung, die so alle zwei, drei Monate stattfindet wird dann sozusagen die generellen staretgischen Entscheidungen getroffen.	
Interview 5		Das heißt dann zum Beispiel auch Teilnahme an NFDI, wobei das wurde / war schon vorher entscheiden worden, aber das wäre dann auch so ein Thema. Also wenn man an großen projekten teilnimmt, was viele Kräfte bindet.	
Interview 5		Es geht jetzt nicht darum mal an EU-Projekten teilzunehmen, die sich dann wieder finanzieren, sonder es geht darum, wo sozusagen (-) Grundsubstanz, also Leistung eher eingebracht werden muss um eine bestimmte Antrag zu befeuern oder mitzumachen, wo Eigenleistung vor allendingen drinsteckt, das muss natürlich durch die beiden Host-Institutionen abgedeckt werden, die ja letztendlich, sozusagen, die Leute bezahlen.	

Interview 5		Die erwarten natürlich auch, dass Ihre Daten, hier aus dem haus, aus dem Marum, aus der Uni (town) entsprechend bearbeitet werden; das heißt wenn ich jtzt hier hundert prozent der leistung oder auch nur 60, 70 Prozent plötzlich in ein projekt stecke, das mir gefällt, dann bleibt natürlich nichts mehr für die übrig, die das letztendlich bezahlen. Das geht nicht. Das ist wie bei einer Firma, sie haben immer sozusagen irgendwo einen Vorstand und einen CEO, CFO und bei uns der CEo wäre jetzt Antje und der CFO, financial Officer wäre Karsten Wurr und dazu kommt noch Michael Schultz, der ist dann beides in einem für das Marum und sie müssen Sie natürlich irgendwann fragen, wenn Sie eine gute Idee haben.	
Interview 5		Wenn Sie was machen wollen, dann müssen Sie im Prinzip so eine Art Antrag stellen: "Aus den Gründen würde ich es gerne machen, strategisches Investment, hier möchte ich gerne etwas reinstecken, um dann in zwei, drei Jahren zum Beispiel das wieder rauzukriegen"	
Interview 5	32:54:00	Wiegesagt ein Lenkungs gremium ist im Moment das hjöchste Entscheidungsboard von (platform), daneben gibt es noch ein Advisory-Board, also eine Scientifically-Advisory-Board,a ls teil vom Marum, das sozusagen das System mit evaluiert, das passiert aber nur alle / fragen Sie mich nicht/ fünf oder sechs Jahre oder sowas. Also das ist nichts für den täglichen Betrieb. (lacht) das ist halt so generell /	
Interview 5	34:08:00		Was macht es denn?
Interview 5		Na ja die schauen sich / also diese sogenannten Advisory-Boards SABs oder Sientifically-Advisory-Boards und wie sie alle heißen mögen die scheun sich normalerweise alle paar Jahre das gesamt ding an im Marum an und schaun sich alle Komponenten an und gucken / Externe, also wirklich Leute die jetzt nicht am Marum und nicht am AWi angestellt sind,. sondern die wirklich von Außen reinkommen honorige Wissenschaflter und Freaks , die eine Meinung haben sollten und die geben dann sozusagen Advice, die sagen dann "Okay also generel / Sie haben ja vorhin nach Visionen oder strategischem Investment gefragt, also (platform) sollte jetzt im Bereich dieses und jenes investieren, also sollte vielleicht hier und da noch reinschauen / also solltin diesen Nutzerkreis sich noch erschließen".	
Interview 5		Das wären so generelle Metaebenen (--). Ja nicht mal Entscheidungen sondern letzendlich Hilfestellungen. Man sichert sich eigentlich damit eiun bisschen ab, dass man jetzt nicht komplett an der Realität vorbeigeht, das tun wir eigentlich sowieso nicht, weil wir natürlich viel zu nah an den Nutzern dran sind, aber es ist sozusagen nochmal so ein Gremium von Lueten, die sich die Sachen sozusagen anschauen udn da ihre Meinung dazu geben	

Interview 5		DSas ist jetzt im Rahmen8unserer Begutachtung nicht so dramatisch, bei Leibnitz zum beispiel ist das relativ strikt, da wird das also sehr sher / also bei Leibnitz produziert man dann so einen Ordner hier (scheint etwas zu zeigen) (RWZs unv. Unterlagen? 35:46) ich weiß es deswegen weil ich gerade im Rahmen von Leibnitz, also nicht ich sodnern einer anderen Datenbank evaluiert wird (unv. 35:54, Krach) nachhaltigkeit kriegen und also so zweihundert Seiten Papier und wenn das schiefgeht dann wird so ein Leibnitz-Institut auch mal abgewickelt, das heißt dann gibt es das nicht mehr. Das passiert und jetzt nciht, aber im Großen udn Ganzen (-) ja (-) ja. (lacht)	
Interview 5	36:15:00		Noch eine Nachfrage zu den Entscheidungen treffen. Welche Rolle spielen dnan die Gruppenleiter?
Interview 5		Ja, das ist eine gute Frage. Es it so dass, die gruppenleiter, sozusagen ihren Bereich im täglichen / mit ihren leuten die Entscheidungen im täglichen Geschäft machen. Das heißt, wenn jetzt Datensätze reinkommen, wenn da Fragen auftreten, wenn jetzt Softwareentwicklungsfragen auftreten. da muss ja jemand da sein der jetzt entscheidet, das machen wir jetzt so, das machen wir jetzt so, das sind einfach verschiedene Flughöhen auf der also (---)	
Interview 5		ich sage mal das Dirketorium schwebt ganz oben drüber, das interessiert die details überhaupt nicht, das ist denen letztendöich / also wie jetzt die Datensätze, in welchem Format, an welcher Stelle geschrieben wir, das ist nichts für das Direktorium. Das ist auch nichts für mich, eigentlich, also solche sachen, da denke ich mir, das werden sie schon machen. Das Direktorium interessiert nur wo geht es generell hin, so.	
Interview 5		Mich interessiert untem Strich zwischen Direktorium un dem gruppenleiter so die gesamtidee zu behlaten. Und zwar über die beidne Institute hinweg. Gruppenleiter sehen ja doch letztendlich eher ihre eigenen Gruppen udn ihren Bereich, ich verushce sozusagen dasd Ganze drüber zu sehen. Und die Gruppenleiter wiederum schaun eigentlich den Leuten auf die finger und gucken, dass das sozusagen läuft und sind sozusagen der tägliche Ansprechpartner, wenn es irgendwoe Probleme gibt, ganz pragmatische Probleme gibt. Von / keine Ahnung / da Netzwerk funktioniet nicht, der Knopf funktioniert nciht / "da kommt ein Nutzer, der hat einen datensatz der sieht so und so aus, wir wissen nciht wie wir den jetzt aufbereiten sollen, welchen Standard " / gut das wissen die Kurratoren meistens, aber /	
Interview 5		"sollen wir es jetzt so machen oder so machen, welche Tags müssen da rein" also es sit eine große bandbreite an täglichen Fragen oder so Dingen, die nicht läuft am Ende des Tages. Und (-) wir haben jede Woche sozusagen mit den Gruppenleitern ein Treffen (unv. 38:34) wo wir dann letztendlich die Sachne eben besprechen, wenn dann auch so Sachen aufgekommen sind die ich dann wissen muss, dann / man darf sich das jetzt nicht so hirarchisch vortellen / du triffst dich jetzt einmal in der Woche und dann sagt Frank-Oliver: "Jetzt macht ihr das", das ist alles Quatsch, so funktioniert das nicht.	

Interview 5		Nein (lacht). Sondern letzendlich ist es (--) eine Diskussion / oder ein sie bringen ihr Wissen ein, ich bringe mein Wissen ein, bringe vielleicht noch ein bisschen die politische / oder was, wo ich sehe an der Stelle kann man besser oder schlechter agieren oder wo gibt es vielleicht Sachen, dass müssen wir so oder so aufbereiten, damir eben das Direktorium am Schluss sagt, ja machen wir so. Am Schluss geht es nämlich viel um Geld und Stellen (niest) von Unten kommt immer das Geschrei, wir brauchen mehr Stellen, wir brauchen mehr Geld.	
Interview 5	(Time: 38:!	Klar die Anforderungen steigen und irgendjemand muss es ja auch leisten, von Oben kommt natürlich Sparen, sparen, sparen und irgendwo dazwischen muss man dann eine lösung finden und das ist dann meine Aufgabe. Also der Vermittler zwischen den verschiedenen Welten und wie gesagt die Gruppenleiter sind in ihrem Bereich (hustet) / Sorry / in ihrem Bereich durchaus auch selbstständig und das wird von mir auch gefördert, dass sie letztendlich auch (räuspern) ihren eigenen Bereich / muss mal kurz etwas zu trinken besorgen, Sorry / darf ich mal kurz zwei Minuten unterbrechen?	
Interview 5	40:00:00		Ja, klar, gerne. Kein problem. (Es entsteht eine kuze Pause bis ca. 40:54)
Interview 5	40:54:00	Gut. Wie gesagt, man darf es sich jetzt nicht so vorstellen, dass das / also gerade im wissenschaftlichen Betrieb, ist das alles nicht so hirarchisch, wie in einer Firma oder soetwas. Das müssen wir davon ausgehen / gut die Gruppenleiter sind in der Regel jetzt festangestellt, hier (-) aber im normalen Betrieb sind diese Art von Datenbanken / jetzt zum Beispiel (platform), die habe ich jetzt 15 Jahre betrieben, letztendlich, ohne dass ich eine einzige feste Stelle darin hatte, das heißt am Ende des Tages ist es auch unheimlich viel Goodwill der Leute sich sozusagen mit dieser Sache zu identifizieren und immer wieder aufSoft-Money, auf Projektgeldern zu arbeiten, ohne feste Stelle, ohne Klarheit ob es weitergeht oder nicht. Das heißt, da kann man nicht so durchregieren und sagen, dann machst du mal das, dann machst du mal das und dann machen wir das zum Schluss. Dieses schon den Leuten auch selber überlassen, dass siedas letztendlich auch entsprechend betreiben und die Gruppenleiter hier arbeite ja auch zu neun- achzig prozent mit Leuten, die eben keine festen Stellen haben. (-) Das heißt (Räuspern) (Husten) Mein Husten immer noch nicht weg, ich merke schon. (Zeitmarke: 42:07) Das heißt sie müssen die Leute auch eher moti- (Räuspern) motivieren, als das sie (unv. aber zusammenhängend mit Husten 42:13) (---) Ich werde es überleben.	
Interview 5	42:07:00	Das heißt sie müssen die Leute auch eher moti- (Räuspern) motivieren, als das sie (unv. aber zusammenhängend mit Husten 42:13) (---) Ich werde es überleben.	
Interview 5	42:22:00		Ja, trinken Sie ruhig, das ist ja alles kein problem. Ich bin ja auch immer fleißig am Trinken. Und ich höre ja eher zu.
Interview 5		Und ich huste gerade mehr als ich rede. (----) Gut, was gibt es noch? Mir fällt nichts mehr ein. (Räuspern)	

Interview 5	42:46:00		Gut, dann mache ich mit der nächsten Frage weiter.
Interview 5		Machen wir so	
Interview 5			Wie ist denn die strategische Ausrichtung von (platform)?
Interview 5		(---)Strategisch gesehen wollen wir letzendlich in die NFDI, das ist das / wir sehen und eigentlich als (-) ich will nicht sagen perfekten, aber als durchaus wichtigen Baustein der NFDI, weil soviele zertifizierte Repositorien gibt es eigentlich nicht. Also im Umweltbereich eigentlich gar nichst in Deutschland soweit ich weiß und strategisch ist es für uns glaube ich / wäre es sehr wichtig auch eine wichtge Rolle in Bereich NFDI zu speilen (-).	
Interview 5		Natürlich ist die NFDI (-) also als nationale Infrastruktur natürlich entschiedend, aber für die europäische Schiene, also die (region) Open Science Cloud natürlich ist / und da sind wir auch schon mit mehreren Projekten sozusagen mit daran beteiligt / der nächste Sprung dann.	
Interview 5		Was heißt Sprung (-) Forschungsdaten sind nichts was nur einen Einzelnen betrifft oder nur ein institut betrifft sondern es betrifft Deutschland, es betrifft ganz (region), eigentlich die ganze Welt. Wollen wir mal die Welt kurz außen vor lassen, zumindest für Deutschland brauchen wir eine starke Infrastruktur und wir brauchen darin gute Spieler und ich denke (platform) ist ein guter Spieler darin und wir brauchen natürlich dann, um sozusagen handlungs- und sprechfähig zu sein, brauchen wir die NFDI, aber letztendlich um dann auch sinnvoll and er europäischen Entwicklung teilzunehmen.	
Interview 5		Also Deutschland macht es ja immer typisch deutsch, kann man jetzt drüber lästern oder nicht, aber ich habe es ja gesehen, wir sind ja auch im Rahmen des deutschen Bioinformatiknetzwerk-Infrastruktur , die (year/number), gestartet ist, sind wir jetzt der deutsche Knoten für ELIXIR geworden.	
Interview 5		ELIXIR ist die (region) life-science infrastructure und das hat nur funktioniert, weil sich Deutschland sozusagen erstmal intern organisiert hat, im Rahmen des sogenannten DNBi deutsches Bioinformatik-Netzwerk-Infrastruktur und dann letztendlich BMWF gesagt hat: "So jetzt sind wir reif" Und das BMWF war auch selber der Meinung, dass wir Teil am europäischen Infrastruktur werden können	
Interview 5		Und andere Länder sind zum Beispiel relativ früh auf diesen Zug aufgesprungen, mit relativ unkoordinierten Aktivitäten, das heißt dann war hier mal eine Arbeitsgruppe oder hier mal ein kleines Konsortium, das sich dann sozusagen mal gefundne hat und das Problem ist dann,wenn man dann koordiniert, organisierte Aktionen deutschlandweit machen will, dann ist es eigentlich doch wieder so ein Sack von flöhen. Und da schafft es Deutschland im Moment durchaus / und mit DNBI haben wir das vorgemacht, sage ich mal so /	

Interview 5		also cih bin da auch relativ im (-) in der Steuerungsdingsda mit drin und betreibe hier eben / also die datenbanken (platform) und (platform) ist auch wiederum Teil von DNBI und ich bin der Leiter von diesem Biodata-Leistungszentrum darin und ich denke was wir jetzt haben ist eien Struktur, die es dann auch erlaubt relativ gut und schnell auch eben an größeren Strukturen, wie eben zum Beispiel den (region)weiten Strukturen teilzunehmen. Und so sehe ich das im prinzip jetzt auch, (platform) hat sich in den letzten 25 Jahren sehr gut als Spieler etabliert, wir machen den nächsten Schritt, strategisch, in die NFDI und den nächsten Schritt in die europäische open Science und das ist eigentlich das Ziel (Räuspern),	
Interview 5	45:05:00	wie esagt, das haben wir auch schon unabhängig von NFDI verfolgt, oder würden wir auch unabhängig von der NFDI verfolgen, aber dann eher als Einzelspieler und jetzt machen wir es sozusagen als Teamplayer mit der NFDI zusammne. Und das ist eigentlich die strategische Ausrichtung an der Stelle.	
Interview 5	47:07:00		Das heißt wie positionieren Sie dann (platform) auf dem Datenmarkt?
Interview 5		Na ja, also (platform) hat sich glaube ich im Bereich umwelt und Environmentalscience, relativ gut schon selber positioniert, also s ist einer der Spieler, die glaube ich weltweit ziemlich anerkannt sind. Wir hatten einen, vielleicht als Nebenbemerkung, sozusagen ein laufendes DFi-gefördertes Projekt, das heißt, seit (year/number) läuft das (platform), das German Federation for Biological Data, das ist im Prinzip so eine Art vorläufer von NFDI gewesen und das ist nichtmal mehr auf unserem Mist wirklich gewachsen sondern das ist auf der DFG-Mist gewachsen.	
Interview 5		das heißt die DFG hat relativ früh erkannt, dass das Thema Forschungsdatenmanagement große / also Thema werden wird und hat dann sozusagen uns wirklich angerufen, ich glaube wirklich 30, 40 Spieler in Deutschland, und gefragt ob sie mitmachen wollen an einer deutschen initiative , für / nennt sich jetzt eben (platform) German Federation for Biological Data und (platform) ist dort die Koordinatorin von diesem Ding, inzwischen ist darus ein verein geworden, den ich jetzte las Schatzmeister betreue, um praktisch auch eien rechtliche Einheit zu schaffen (Räuspern9 und das heißt damit war ich / das war noch alles vor der NFDI, da wusste noch keiner was von der NFDI,	

Interview 5	das war eigentlich das strategische Ziel zu sagen wir bündeln jetzt 20 Partner in einem Verein, am Ende des tages und nehmen das dann als rechtliche Einheit, um praktisch damit in die größeren strategischen 7 also um uns, wie Sie sagen, am Markt zu positionieren und machen (platform) größer, dass wir sagen , die Gesellschaft für (domäne) daten e.V., eingetragener Verein gemeinnützig, das der sozusagen als Markenprodukt rausgeht und sagt: "So wir sammeln jetzt eben n8icht nur Umweltdaten ein sondern wir sammeln eben auch daten ein von Froschungs-/ Diversitätsdaten ein, wir sammeln Sequenzdaten, also Nukleotidendaten ein, kommt zu uns bringt uns eure daten , das was also bei euch Life Science ist und wir sorgen dafür, dass sie in die richtigen repositorien gehen.	
Interview 5	Das heißt in (platform) stecken auch acht, sieben oder acht Museen, Sammlungen drin, die bayrische Saatssammlung, die Museeum König und wie sie alle heißen, die praktisch auch klassische richtige Biodiversitätsdaten anmehmen können und letztendlich auch ablegen können. Repositorium ist es letztendlich. (Zeitmarke 50:00). Also sozusagen das Mini-NFDI und aus dem ist sozusagen eine BioDiversity jetzt entstanden das heißt also der / das product sozusagen, das wir sozusagen haben war (platform) plus was dazu kam, Sequenzdaten, Biodiversitätsdaten (platform) und jetzt machen wir es mit NFDI nochmal einen Schritt größer, für NFDI für Biodiversity erstmal um praktisch vor allem noch Citizens-Science-Daten dazu nehmen /	
Interview 5	also wenn Sie jetzt sagen, wo plazieren wir uns im Markt, dann ist das ja kommerziellSache natürlich jetzt, aber eigentlich würden wir jetzt / ich weiß ich habe auch / ich tendiere auch dazu es als Markt und als Produkt und so weiter zu bezeichnen, weil ich auch / komme aus der Wirtschaft sozusagen / elterlich geprägt bin, aber anyway. Ich habe ja gesagt, ich betreibe meine Sachen, wie eine Firma, die Währung ist in Publikationen und Zitaten und sonstiges Zeug, aber mann kann es ja so und so spielen.	
Interview 5	Das ist auch nicht so falsch, das heiß also so vom Produkt her ist die Idee, dass wir uns eben mit (platform) erweitern, in verschiedene Produktsparten, das ist die Diversitätsanalyse, Sequenzanalyse aus Sequenzdaten und mit der NFDI gehen wir jetzt gezielt nochmal in den Bereich der Citizen-Science, Vereine, Verbände, alles was so im Großbereich / also nochmal einen Schritt zurück.	
Interview 5	Es ist so, dass unheimlich viele sehr sehr wertvolle Daten werden durch Amateure gemacht. Der Schmetterlingsforscher, der Insektenforscher, der Vogelbeobachter, da gibt es den Spinnenverband, den Schmetterlingsverband, dann gibt es die Vögelbeobachter.	

Interview 5	Das sind leute, die machen nichts anderes, als in ihrer Freizeit rausgehen und sich damit zu beschäftigen, Hobby, aber sie produzieren unglaubliche Datenmengen. Diese zu mobilisieren und letztenndlich auch zugänglich zu machen für die Wirtschaft, aber auch natürlich für die politik und die Entscheidungsfindung, gerade im Bereich Artensterben, (unv.) 52:19)-forschung das war einer der Hauptziele der NFDI für Biodiversity, also wir haben (platform) genommen, gesagt wir haben die Archive und das und das und hier und hier und jetzt gehen wir einen Schritt weiter und beschäftigen uns ganz bewusst mit Bürgerwissenschaften, Verbänden (--) Vereinen, was auch immer draußen rumgurckt zusammenzubringen, damit wir sozusagen von denen /	
Interview 5	jetzt nicht die Daten abziehen sondern von denenzusammen die Daten nachbereiten, damit sie nachnutzbar werden. Und das ist sozusagen, wo wir uns sozusagen automatisch in dne Markt bringen, bis in die Infrastruktur, die letztendlich hoffentlich den Leuten hilft ihre wertvollen Daten nachnutzbar zu machen. Weil im Moment ist es oftmnals so sie haben weder die technischen Kompetenzen zum teil, umd die Sachen in entsprechende Datenbanken zu packen, sie haben manchmal auch nicht das Wissen welche Standards verwendet werden und und und, sondern sie sind halt in erster Linie dazu da rauszugehen und Datenaquise zu machen, also Vögel zu beobachten, Schmetterlinge zu sammeln, Insektenanzugucken und und und.	
Interview 5	Das ist toll, weil es gibt wenige Experten auf der Welt, die das noch überhaupt machen können, aber das Ganze in Struktur zu bringen, zu digitaliseren und letztendlich dann auch letzendlich entsprechend wieder verfügbar zu machen, in Datenbanken nachnutzbar zu machen, das ist dann die Aufgabe von NFDI. (--) Ganz so einfach ist es nicht, da hängen noch viel Emotionen an, aber im Großen und Ganzen.	
Interview 5		
Interview 5	Also sozusagen das Mini-NFDI und aus dem ist sozusagen eine BioDiversity jetzt entstanden das heißt also der / das product sozusagen, das wir sozusagen haben war (platform) plus was dazu kam, Sequenzdaten, Biodiversitätsdaten (platform) und jetzt machen wir es mit NFDI nochmal einen Schritt größer, für NFDI für Biodiversity erstmal um praktisch vor allem noch Citizens-Science-Daten dazu nehmen / also wenn Sie jetzt sagen, wo plazieren wir uns im Markt, dann ist das ja kommerziellSache natürlich jetzt, aber eigentlich würden wir jetzt / ich weiß ich habe auch / ich tendiere auch dazu es als Markt und als Produkt und so weiter zu bezeichnen, weil ich auch / komme aus der Wirtschaft sozusagen / elterlich geprägt bin, aber anyway.	

Interview 5		Ich habe ja gesagt, ich betreibe meine Sachen, wie eine Firma, die Währung ist in Publikationen und Zitaten und sonstiges Zeug, aber mann kann es ja so und so spielen. Das ist auch nicht so falsch, das heiß also so vom Produkt her ist die Idee, dass wir uns eben mit (platform) erweitern, in verschiedene Produktsparten, das ist die Diversitätsanalyse, Sequenzanalyse aus Sequenzdaten und mit der NFDI gehen wir jetzt gezielt nochmal in den Bereich der Citizen-Science, Vereine, Verbände, alles was so im Großbereich / also nochmal einen Schritt zurück.	
Interview 5		Es ist so, dass unheimlich viele sehr sehr wertvolle Daten werden durch Amateure gemacht. Der Schmetterlingsforscher, der Insektenforscher, der Vogelbeobachter, da gibt es den Spinnenverband, den Schmetterlingsverband, dann gibt es die Vögelbeobachter. Das sind leute, die machen nichts anderes, als in ihrer Freizeit rausgehen und sich damit zu beschäftigen, Hobby, aber sie produzieren unglaubliche Datenmengen.	
Interview 5		Diese zu mobilisieren und letztenndlich auch zugänglich zu machen für die Wirtschaft, aber auch natürlich für die politik und die Entscheidungsfindung, gerade im Bereich Artensterben, (unv. 52:19)-forschung das war einer der Hauptziele der NFDI für Biodiversity, also wir haben (platform) genommen, gesagt wir haben die Archive und das und das und hier und hier und jetzt gehen wir einen Schritt weiter und beschäftigen uns ganz bewusst mit Bürgerwissenschaften, Verbänden (--) Vereinen, was auch immer draußen rumgurckt zusammenzubringen, damit wir sozusagen von denen / jetzt nicht die Daten abziehen sondern von denenzusammen die Daten nachbereiten, damit sie nachnutzbar werden.	
Interview 5		Und das ist sozusagen, wo wir uns sozusagen automatisch in dne Markt bringen, bis in die Infrastruktur, die letztendlich hoffentlich den Leuten hilft ihre wertvollen Daten nachnutzbar zu machen. Weil im Moment ist es oftmnals so sie haben weder die technischen Kompetenzen zum teil, umd die Sachen in entsprechende Datenbanken zu packen, sie haben manchmal auch nicht das Wissen welche Standards verwendet werden und und und, sondern sie sind halt in erster Linie dazu da rauszugehen und Datenaquise zu machen, also Vögel zu beobachten, Schmetterlinge zu sammeln, Insektenanzugucken und und und.	
Interview 5	50:00:00	Das ist toll, weil es gibt wenige Experten auf der Welt, die das noch überhaupt machen können, aber das Ganze in Struktur zu bringen, zu digitaliseren und letztendlich dann auch letzendlich entsprechend wieder verfügbar zu machen, in Datenbanken nachnutzbar zu machen, das ist dann die Aufgabe von NFDI. (--) Ganz so einfach ist es nicht, da hängen noch viel Emotionen an, aber im Großen und Ganzen.	
Interview 5	53:48:00		Welche konkreten Ziele setzen Sie denn mit (platform)? Oder innerhalb (platform).

Interview 5	(--) Also (-) konkrete Ziele ist im Moment (-) wie gesagt dass Daten / also dieses Front-Office-, Back-Office-Modell aufzubauen, dass wir im Back-Office strukturierter sind (-) was uns fehlt ist Dokumentation, jetzt sind wir wieder beim Thema Firmen, wenn Sie im Bereich von Firmen sich bewegen, werden Sie feststellen, dass ganz viel arbeit in Dokumentation fließt, das ist natürlich auch notwendig, weil einfach / wenn eben Haftungsfragen da sind, man eben dokumentieren kann, warum man das so und so gemacht hat, dann ist aber zum Teil uach notwendig, gerade wenn so eine Gruppe wächst, dass eben andere modular in diese Teile mit einspringen können,	
Interview 5	das heißt wenn Sie so ein diffuses implizites Wissen haben , das funktioniert so in einer Garagenfirma, wo jeder sich Abends beim Bier trifft, man unterhält sich und dann weiß irgendwie jeder was jeder andere macht. Dann brauchen Sie nicht so viel fokumentieren, aber wenn Sie von einer Garagenfirma mit zwei, drei, vier Leuten rausgehen auf eine Größe von 20 oder 30 Leuten, dann müssen Sie dieses implizite Wissen irgendwann explizit machen, das heißt Sie müssen es dokumentieren. Das wurde nicht gemacht in (platform) (-) einfach der Historie geschuldet.	
Interview 5	Das ist eines der konkreten Aufgane, die wir im Moment haben, das wir niederschreiben, was wir eigentlich machen, damit aber auch klar definieren was wir nicht machen. Das heißt also eines der Themn, wenn Sie so nach konkreten Zielen fragen, ist auch Abgrenzkriterien zu finden, was wir nich tun, weil wir natürlich im Moment überlastet / also überladen werden mit Anfragen für Forschungsdatenmanagemnt und Datenaufbewahrung, wo wir sagen es ist nicht unser Ding (Zeitmarke: 55:46). Also, wir können nicht alles nehmen, wir sind nicht das Multirepositorium for Everyting, sondern wir haben schon einen gewissen Fokus, aber das muss mal definiert werden, das muss niedergeschrieben werden.	
Interview 5	Also terms of reference, terms of use, was genau ist eigentlich (platform), was nehmen wir, was nehmen wir nicht, wo grenzen wir ab. Soll ja nicht willkürlich sein "Ja, (...), die mag ich die nehme ich, aber die von, keine Ahnung, Hugo Sonstwas, die nehme ich nicht, weil der Name gefällt mir nicht oder so". So geht es ja nicht, also da muss / also Dokumentation um die, ja die Arbeitswege und letztendlich auch / was wir machen, das ist ein großes, ganz pragmatisches, aktuelles Ziel (-) auch notwendig um eben NFDI-Teil aufzunehmen, um eben auch darin zu sagen was ist (platform), was ist NFDI, was ist (platform), was tun wir, wie wirkt das zusammen und ja, strategisch gesehen natürlich ein thema Datascience, um zu versuchen uns letztendlich technisch besser aufzustellen, damit wir (-) damit wir sozusagen hier den / eine bessere Nachnutzbarkeit und auch technische Ziele / also sozusagen intern in der Lage sind besser mit den Daten umzugehen,	

Interview 5		da ist viel konkret auf die Datenbank, Technologie, wie es abgelegt wird, die Koratoren zu schulen, wir haben jetzt einiges an neuen Koratoren drin, eben auch weil im Moment muss man sagen relativ viel Geld fließt in diesen Bereich, wir kommen eigentlich kaum nach Leute einzustellen. Das ist übriegens auch nichmal ein thema, wenn Sie meinen (lacht) startegische Ziele , Ausbildung.	
Interview 5		Also, wir können nicht alles nehmen, wir sind nicht das Multirepositorium for Everyting, sondern wir haben schon einen gewissen Fokus, aber das muss mal definiert werden, das muss niedergeschrieben werden. Also terms of reference, terms of use, was genau ist eigentlich (platform), was nehmen wir, was nehmen wir nicht, wo grenzen wir ab. Soll ja nicht willkürlich sein "Ja, (...), die mag ich die nehme ich, aber die von, keine Ahnung, Hugo Sonstwas, die nehme ich nicht, weil der Name gefällt mir nicht oder so". So geht es ja nicht, also da muss / also Dokumentation um die, ja die Arbeitswege und letztendlich auch / was wir machen, das ist ein großes, ganz pragmatisches, aktuelles Ziel (-)	
Interview 5		auch notwendig um eben NFDI-Teil aufzunehmen, um eben auch darin zu sagen was ist (platform), was ist NFDI, was ist (platform), was tun wir, wie wirkt das zusammen und ja, strategisch gesehen natürlich ein thema Datascience, um zu versuchen uns letztendlich technisch besser aufzustellen, damit wir (-) damit wir sozusagen hier den / eine bessere Nachnutzbarkeit	
Interview 5	55:46:00	und auch technische Ziele / also sozusagen intern in der Lage sind besser mit den Daten umzugehen, da ist viel konkret auf die Datenbank, Technologie, wie es abgelegt wird, die Koratoren zu schulen, wir haben jetzt einiges an neuen Koratoren drin, eben auch weil im Moment muss man sagen relativ viel Geld fließt in diesen Bereich, wir kommen eigentlich kaum nach Leute einzustellen. Das ist übriegens auch nichmal ein thema, wenn Sie meinen (lacht) startegische Ziele , Ausbildung.	
Interview 5		Ausbildung ist tatsächlich auch ein strategisches Ziel, was wir in der NFDI sehr stark verfolgen, weil wir einfach einen immensen Mangel an Leuten haben, wir kriegen einfach niemanden. Es ist kein geldproblem am Ende, muss ich einfach so sagen. das ist wirklich ein problem die fachkräfte zu kriegen. Und deshalb, weil Sie vorhin auch gefragt haben, wer arbeitet bei uns, eigentlich jeder, der motiviert ist und gewisses Wissen zu einem Thema mitbringt. Also wir haben uns eben bemüht jemanden aus Kulumbien einzustellen, also ich hoffe das geht jetzt durch, ich warte darauf, dass sie sich meldet.	
Interview 5		Das Arbeitsamt hat sich dermaßen angesetllt, weil es kein EU-Land ist, risen Thema, weil wir einfach Fachkräfte brauchen. Jetzt nicht nur im IT-Bereich, da sowieso, da ist sowieso (Pft-Geräusch), riesen Thema, aber auch im Prinzip, das ganze Thema Forschungsdatenmanagement ist im Moment so ein heißes Thema, jede Uni stellt leute ein und / es ist/ ja.	

Interview 5		Giut also konkrete Ziele, einerseits natürlich klare Dokumentation nochmal klar auch, was Sie vorhin angesprochen haben, die internen Abläufe und internen Kommunikationen und Strukturen zu verbessern, wier gesagt wir sind an zwei orten lokalisiert, mit 60 Kilometern dazwischen (-), das ist eine Herausforderung, weil es sozusagen mal schnell in der Teeküche sich treffen ist nicht so einfach, wir brauchen deutlich bessere Austausch verfahren - medien, Treffen, ob immer die Treffen so gut ist wird man sehen, da muss mehr Transparenz und Durchlässigkeit sein, auch zwischen den Leuten.	
Interview 5	57:23:00	das sie mal hier und mal dort sitzen. Das war bisher nicht so sehr / das war schon, aber bisher nicht so deutlich, nicht so klar. Wir werden jetzt auch ein retreat veransalten, um das ganze / nochmal so ein bisschen,/ die Leute auch zusammenzubringen, damit auch im Kopf soetwas passiert, das sind ie konkreten pragmatischen Ziele. Die längerfristigen Ziele ist Datascience, im Bereich des (unv. 59:33), da müssen wir eine Teilautomatisierung versuchen letztendlich, von den Kurratoren last wegzunehmen, damit eben die daten vorbearbeitet werden. Front-Office, Back-Office, da kommen wir in das Gleiche, was ich vorhin schon erzählt habe, mehr / es reicht, wenn man es / it keeps us busy for the next two/ zwei bis drei Jahre, so.	
Interview 5	59:54:00		Haben Sie denn auch konkrete Ziele, was Anzahl an Nutzer oder Datenmengen oder so angeht?
Interview 5		(-) Key-Performance-Indicators. Jaein, also wir verfolgen natürlich unsere Nutzerzahlen, das ist klar, wir verfolgen die Anzahl an Tickets, die reinkommen, die abgearbeitet werden. Also wir monitoren schon, was passiert. Im Moment ist es auf einem guten Niveau, ich würde da jetzt / also ich bin kein Freund von (--) ihr müsst jetzt noch/ da gab es mal Anätze in der Vergangenheit / das möchte ich nicht / jetzt noch irgendwie tausend Tickets mehr hier oder noch zehntausend Datensätze mehr, weil Quantität hat für mich nicht so die Bedeutung , Qualität hat für mich die Bedeutung,	
Interview 5		das heißt also lieber habe ich, ich sage mal, hu8ndert Datensätzeweniger, aber dafür sin sie so aufgearbeitet, dass sie wirklich nachnutzbar sind, als dass ich noch die koratoren antreibe noch den letzten Datensatz da reinzuschieben nur damit die / damit sozusagen die Zahl nach oben geht und am Schluss haben wir nur noch Schrott dortdrin, das bringt nichts.	
Interview 5		Weil das passiert, wenn Sie eine gegeben Anzahl an Men- oder Womenpower haben, sozusagen und sie packen da immer mehr drauf, dann kann man natürlich sagen: "Okay ihr könnt effizienter werden", das geht eine Weile gut, irgendwann wir die Effizienz als Schlampigkeit umsiedeln, denn sie wollen auch irgendwann um / ich sage mal viele hier im Bereich der Kuration sind tatsächlich viele Frauen tätig sind, ich sehe das so, sie sind einfach gründlicher als Männer, ist einfach so. Männer sind eher so für das grobe.	

Interview 5		Nein, im Bereich IT ist es halt / ist es fast nur männlich geprägt. Im Bereich der Kuration, Datenaufbereitung und Managemnt ist fast nur Frauen tätig, also es ist kein gewolltes Ding so sondern das einfach so. Wenn Sie eine Stellenausschreibung machen, das ist immer so. Also wenn sie Kuratoren ausschreiben haben Sie zehn weibliche Bewerber und einen Mann und der taugt nichts und wenn Sie Informatik ausschreiben, dann haben Sie zehn Männer und ja taugt auch keiner was, aber auf jeden Fall finden Sie kaum einen, das ist so. Und unsere Genderbalance over all ist ziemlich gut, fünfzig-fünfzig kann man sagen (lacht)	
Interview 5	61:19:00	das ist kein Thema, aber das ist inhaltlich tatsächlich getrennt (lacht). So zurück zum Thema (-) ich bin kein großer Freund von irgendwelchen Zielvorgaben, die jetzt sagen, was weiß ich "Bis Ende des Jahres müsst ihr noch 10.000 mehr dadurch gejagt haben und noch 50 / noch 500 Tickets mehr bearbeitet haben", das führt aus meiner Sicht, wie gesagt nur dazu, dass irgendwann mal die Leute einfach schlampig werden, weil die Leute irgendwann um 4 oder 5 zu ihren Familien wollen und dann wir halt das zeug noch schnell reingedonnert wird (-) also nein.	
Interview 5	62:52:00		Und wie kontrollieren Sie dann die gesteckten Zeiel?
Interview 5		(-) Also ich sage mal so, wir gucken uns die Gesamtanzahl natürlich an, an archivierten Datensätzen an, wir gucken uns natürlich die Anzahl der Tickets an, wir gucken uns natürlich an wie viel Nutzerzahlen wir haben, wie viele reaccuring Users wir haben, also typischen Web-Statistiken, die wir haben. Wir gucken uns natürlich an; also noch nicht so, aber das wir verstärkt kommen; wie oft auch die Datensätze zitiert werden, sozusagen, wie sie benutzt werden, das ist noch im Werden im Moment (Altimetrics? 63:30) war da so ein Stichpunkt, das wollen wir auch einbauen bei uns, das war (unv, 63:33), wie zitiert und benutzt wird und so im Gesamtbild sollte sich halt /	
Interview 5		ich sage mal wenn es aufwärts geht ist es gut, wenn es gleich bleibt ist es auch noch nicht schlecht, wenn es runter geht, müssen wir uns überlegen wo das Problem ist. Also haben wir es, dass wir eben nicht mehr attraktiv sind, gehen die Nutzer wo anders hin? Was wir auch machen ist übrigens alles, also die ganzen Fragen da, die haben wir in Extenso im Rahmen des Deutschen Biomatik Netzwerkinfrastruktur durchgehechelt, wo eben das BMWF immer wieder nach Key-Performance-Indicators gefragt hat, jetzt ohne Zielvorstellung, aber zumindest wie ist denn der Satatus und das / daraus ist das fann auch entstanden, dass man eben jetzt mal systematisch das Ganze monitort plus User-Survays / also wir haben ein Instance- User-Survay am laufen, das heißt das ist ein ganz ganz kurze Fragen, das sind nur drei Fragen in und ein Freitext.	

Interview 5		Wo drinsteht, ich glaube Geschwindigkeit, also Speed, usability und also wie zufrieden war der Nutzer mit Responsivness. Ich glaube eins ist Responsivness, Quality, Speed oder soetwas in der Art, wo wir / und dann kann er noch einen Freitextfeld für Kommentare eingeben und damit letztendlich kriegen wir dann Nutzerfeedback. (--) Gibt es irgendwo ein problem, sage ich mal so. Wenn da X-Mal kommt "Ey, ich habe fünf E-/ Tickets geschrieben /" genau, das wollte ich noch sagen, bei uns läuft alles über ein Ticketsystem, das heißt also, alles was reinkommt wird im Ticketsystem vermerkt,	
Interview 5	64:30:00	das isnd nicht irgendwelche E-Mails, die irgendwo verloren gehen, sodnern ein Ticketsystem, wir können ganu sehen was reinkommt, was bearbeitet wird, theoretisch qauch wie lang für etwas gebraucht wird, aber das ist Datenschutzrechtlich wieder schwierig. Also da gibt es verschiedene Möglichkeiten sowas zu monitoren, aber eben auch den Feedback durch die Nutzer, wenn jemand immer wieder ähnliche Fragen kommen oder probleme kommen dann muss man halt schauen woran liegt das.	
Interview 5	65:34:00	Also, wie gesagt, jetzt nicht diese harten Zielvorgaben und so industriemäßig Akkordarbeit. " mach noch fünf Löcher mehr und dann ist gut", sondern / so funktioniert es halt nicht. Wie gesagt Qualität ist für mich wichtiger als Quantität, und die Leute müssen da irgendwie glücklich sein, dabei, sonst wir man irre (lacht)	
Interview 5	65:59:00		Welche Herausforderungen und Schwierigkeiten gibt es denn beim Management von (platform)?
Interview 5		Ja (-) also, (5 Sekunden) ganz klar natürlich die Leute bei der Stange zu halten, also sie arbeiten ja dadurch, dass sie nur einen relativ geringen Teil an festangestellten haben, arbeiten Sie eigentlich mit moving Targets, das heißt also, die Leute erstmal zu finden, dann die Ausbildung dauert (-) im Kurationsbereich zwischen einem halben Jahr bis einem Jahr. Sie haben dann aber Projektlaufzeiten die, zum Teil, so wie aktuell bis zum Teil nur bis zwei Jahre sind / anderthalb bis zwei Jahre.	
Interview 5		bei Eu-Projekten sind es auch mal drei oder vier Jahre. Beim DFG drei Jahre. Das heißt Sie bringen Leute rein, Sie bilden Sie aus und wenn sie gerade richtig produktiv werden läuft Ihnen das projekt aus oder der Mitarbeiter sagt, zurecht, was ist meine Zukunftsperspektive hier, ich schaue mich nach zwei Jahren nach einem anderen job um, weil gerade jetzt in der aktuellen Situation wird sowohl in der Industrie, als auch in vielen Universitäten, Instituten werden Forschungsdatenmanager oder Forschungsdatenscientists sozusagen gesucht, die dann zum Teil eben mit festen oder dauerhaften Stellen winken.	

Interview 5		Das heißt also da vom Managemnt ist es schon / die Fluktuation des Personals/ das es in der Forschung eher gewünscht ist, weil dann immer neues machen will, das ist eben auch der Unterschied in der Infrastruktur, es ist natürlich kompliziert. Weil Sie brauchen natürlich ein Set von Core-People, von wirklichen ausgebildeten leuten, die das Wissen auch erhalten und transferieren, damit sie das machen können deshalb ist auch die Ausbildung / oder wird einen großen Stellenwert bekommen.	
Interview 5	67:31:00	Aber / Also das zu erhalten in diesem ständigen Spannungsfeld zwischen "werden sie genehmigt, werden sie nicht genehmigt", wie jetzt ja auch, kommt die NFDI oder nicht. Wenn wir wüssten, dass die NFDI kommt ist es für uns nochmal ein ziemlicher Gamechanger, weil mit einem Schlag würden wir nach (town) dann 15 oder 20 zusätzliche Stellen bekommen, die wir erstmal besetzen müssen. Aber damit hätten wir nochmal ganz andere Möglichkeiten nochmal / jetzt nicht nur für (platform) sondern für das gesamte Forschungsdatenmanagement in (town) zu agieren und damit natürlich auch für (platform) zu agieren.	
Interview 5		Wenn es nicht kommt, (--) ja (lacht) müssen wir uns anderweitig umschauen, das heißt also, man ist ständig sozusagen dabei geld ranzuschaffen. Die durchscnittliche Bewilligungsrate von Anträgen liegt zwischen 20 und 30 prozent, wir kriegen wahrscheinlich so zwischen 50 und 60 Prozent unserer Anträge, weil wir sehr bekannt sind in der Community und weil es normalerweise jetzt gebraucht wird. Aber trotzdem haben Sie immer ein Risiko von sagen wir 50 Prozent, dass der Antrag nicht durchgeht,	
Interview 5		das heißt also wenn Sie / jetzt so auf einen einfachen Satz gebracht : Entweder ich habe Geld und keine Leute oder ich habe Leute und kein geld. Also eins von beidem tritt immer ein. Hat man Geld ist es so, dass man gerade Leutre nicht greifbar hat, weil der Markt schwierig ist und man vielleicht gerade jemanden hat gehen lassen müssen, weil da noch kein geld da war (--) hat man Leute / wie gesagt jetzt im anderen Fall.	
Interview 5	68:38:00	Man weiß genau denn bräcuhtest du, dann hast du kein Geld. Und der universitäre Bereich ist nicht gerade geprägt von Felexibilität und Dynamik in dem Bereich, also Administrationen sind (--) ja. Das dauert. Also im Moment stellen wir gerade wieder drei oder vier Leute ein (--). Da kann ich an die Decke gehen, wir lange soetwas dauert. Also in der Firma / ich habe ine kleine Firma nebenbei, wenn ich jemanden brauche und das Geld ist da, dann kann ich sagen: "Pass auf morgen fängst du an", und der Vertrag und so das dauert dann vielleicht noch zwei Wochen.	
Interview 5	70:00:00	Hier, da muss augeschrieben werden. Der Btriebsrat gefragt werdern, die Frauenbeauftragte, die Putzfrau und weiß der Geier wer noch alles. Und am Ende kommt auch nichts anderes raus, als das was man vorher wusste. Es gibt nur einen Kandidaten und (lacht) den stellt man dann ein, nur dass es dann leider nochmal sechs Monate dauert und wenn man Pech hat, der Kandidat sich inzwischen schon wo anders hin orientiert hat, was ich verstehen kann.	

Interview 5		Ja also von der Managementseite ist das ein Drama (lacht) also ja. Sie spielen ständig mit verschiedenen Faktoren. Das eine ist die Human Ressources, die Sie haben, die Sie so zu sagen glücklich halten müssen und die natürlich auch ihre eigenen Ideen haben, wie sie sich weiterentlickeln wollen oder wie jetzt grade (lacht) vorletzte Woche - ja vor zwei Wochen- kommt ein Mitarbeiter, der grade an einem EU Projekt angefangen hat.	
Interview 5		Denkt man, oh Gott sei dank endlich mal was erldeigt, dauert vier Jahre, alles gut. "Ja tut mir leid, ich habe grade ein Angebot bekommen und ich wollte schon immer mal in die Wissenschaft zurück. Ich bin zum 1.3. weg" -ähä was, ja stehst du dann da und denkst dir ok, jetzt hast du das EU Projekt, du hast das Geld aber leider keinen Mitarbeiter mehr. Kann ich ihm nicht verdenken, weil ich meine, ja ist halt so.	
Interview 5	70:38:00	(lacht) Ich bin dann auch nicht böse aber von der Managementseite her ist das natürlich... ständig ist man dabei irgrndwelche Lücken zu füllen. Entweder finanzielle Lücken oder menschliche Lücken, ist egal wie man es betrachtet. Das potenziert sich natürlich also ich habe mit dieser Aufgabe hier jetzt rund 70 Leute unter mir. Die, also die Wahrscheinlichkeit, dass das passiert -wundert mich, dass hier grade keiner rein kommt- also, dass irgendwas passiert hat sich also von -früher hatte ich so 20 bis 25, weiß nicht, hat sich jetzt mindestens verdoppelt ne	
Interview 5	72:07:00	Wenn nicht sogar verdreifacht, denn irgendwie, es geht nicht linear, sondern exponentiell habe ich das Gefühl. (lacht)	
Interview 5	72:16:00		Gut, haben Sie sonst noch weitere Herausforderungen und Schwierigkeiten?
Interview 5		Jetzt nicht mehr, ähm…	
Interview 5			Ok, dann mach ich weiter mit der nächsten Frage sonst, hm?
Interview 5	73:15:00		Gut, dann bin ich soweit mit dem organisatorischen Teil, mit der Plattform fertig und würde weiter machen mit dem Plattform Gouvernance Nutzungsaspekt.
Interview 5		Oha.	
Interview 5			Ja ähm, meine erste Frage ist, welchen Regeln unterliegen die Nutzer?
Interview 5		Wie gesagt, ähm, noch keinen. Im Prinzip kann jeder kommen und anfragen, ob er seine Daten bei uns ablegen kann. Und das wollen wir in Zukunft durch eine gewissen policy ein wenig strukturieren, dass wir sagen, bestimmte Anfragen braucht ihr gar nicht stellen, weil die werden wir nicht nehmen.	

Interview 5		Also wenn jetzt so eine typische Anfrage ist, "ich hab hier irgendwie fünf Terrabite an Daten aus der Physik, könnt ihr die nehmen?" - Nein, als ja technisch ja aber wollen nein, ist bei uns nicht richtig. Also im Moment gibt es keine festgeschriebenen Regeln, nur implizierte Regeln. Das heißt also wenn so eine ANfrage kommt, dann landet die beim Gruppenleiter und später dann bei mir und dann wird entschieden, verweisen wir auf andere Positorien. Und das muss, das wird in Zukunft denk ich mal, das muss verschriftlicht werden, das muss klarere und transparenter werden, was es im Moment noch nicht ist.	
Interview 5	74:33:00		Welche Anforderungen stellen Sie denn an die hochgeladenen Daten?
Interview 5		Also sie müssen natürlich erstmal strukturiert vorliegen, also sie müssen digital vorliegen, klar. Was auch nicht immer der Fall ist. Aber ja die müssen natürlich digital vorliegen und zwar nicht als gescanntes - ja könnten wir theoretisch auch, aber jetzt so eher nicht so, sondern schon eben strukturiert. Ob das jetzt Excel ist oder ne Datenbank ist, ist egal ist nur das Hauptformat aber netztendlich strukturiert. Entsprechende Metadaten müssen natürlich da sein.	
Interview 5		Also ohne Daten, ohne Metadaten sind die wertlos. Das heißt also Datenformate müssen natürlich nicht-poprietär sein. Also wenn das nur eine bestimmte Software einer bestimmten Firma lesen kann, ne danke. Kann ja keiner nachnutzen ne, typischer Fall: Firma geht pleite, Software gibt es nicht mehr, Daten sinnlos. Also und der Rest machen dann die Kuratoren. Die gehen dann in den Dialog mit den Submittern und dann bringene die denen dann letztendlich ihre Daten in Form.	
Interview 5		Das war aber bei (platform) also das ist bei (platform) aber sehr offen. Die können dann im Prinzip auch ein Word File schicken und die Kuratoren versuchen dann durch copy und paste die Sachen letztendlich in die Datenbank zu bringen. Das wird in Zukunft wohl, also da werden wir in Zukunft wohl ein bisschen strikter werden, weil das kostet einfach zu viel Zeit. Also das geht nicht. Aber so ein Excel file ist ok, so CSV was immer als sturkturierte Information kommt. Das ist immer relativ anspruchslos. Wie gesagt, das machen dann die Kuratoren.	
Interview 5	76:08:00		Wie stellen Sie denn sicher, dass eine ausreichende Anzahl an Forscherinnen und Forschern Daten in ausreichender Qualität und Menge hochladen?
Interview 5		Oooh, wie stellen wir das sicher.. Ähm..müssen wir das sicherstellen? (lacht) Ich sag mal so, wir haben ja eher das Problem, dass zu viele was hochladen wollen. Sogesehen stellt sich die Frage jetzt nicht so. Also wie stellen wir das sicher, dass nicht zu viele Daten bei uns landen, die dann wir nicht bearbeiten können. Also das ist jetzt... kann ich, kann ich Ihnen nicht sagen also, es kommt ja.	
Interview 5			Und wie bewegen Sie dann die Forscher, (platform) zu nutzen?

Interview 5		Einerseits sind wir in vielen Journals bereits als Repositorium genannt. Das heißt also da werden die Leute publizieren und sozusagen ihre Daten hinterlegen müssen. Dann werden wir als Referenz vom Positorium genannt. Darüber kommen eigentlich die meisten Sachen. Und der Rest ist einfach Bekanntheitsgrad. Das übliche halt. Konferenzen in Projekten, Teilnahme an Projekten, wo (platform) dann quasi das Positorium ist.	
Interview 5		Genau. Konferenzen, Projektteilnahme, Kooperation mit vielen Journals, die uns dann quasi als Referenz Repositorium nennen. Mund-zu-Mund Propaganda, wenn Sie mal ne gewisse user community haben und Multiplikatoren in einzelnen Instituten, dann sagt der eine dem nächste "wo hast denn du das Zeug abgelegt? Ah bei (platform). Dann schaff ichs da auch hin."	
Interview 5		Also das ist jetzt eher so, wenn Sie mal in den, das ist ja immer so so so ne lack Phase. Wenn Sie irgendwan, irgendwann kommen Sie mal in so eine Phase, da ist es dann ein Selbstläufer. Ich will jetzt nicht sagen, dass (platform) ein kompletter Selbstläufer ist, aber sie haben zumindest diesen Punkt überschritten, dass die Leute wissen eigentlich	
Interview 5		von (platform) und es tauch auch in fünftausend, also wenn Sie irgendwo hingehen dann taucht es immer irgendwo auf. Also irgendeiner nennt immer (platform).	
Interview 5	78:20:00	Und dann sind sie letztendlich im Markt drin, also wir haben jetzt eigentlich nicht, also wir haben jetzt vermutlich diese - jetzte sind wir wieder in Worten des kommerziellens Sektors- push market, pull market. Wir sind also fast schon im Bereich des pull markets. Das heißt also die Leute wollen jetzt zu uns kommen und wir sind nicht mehr so im push market, dass sie ständig sozusagen Werbung machen müssen dafür, dass jemand kommt, sonder eher in der Situation, dass die Leute von sich aus kommen, als dass, die kaufen die Tempotaschentücher oder die Softis natürlich, weil sie schnupfen haben und damit halt landen die bei uns letztendlich ne.	
Interview 5			Und wie stellen Sie dann das Vertrauen in die Daten her?
Interview 5		Also eins der Punkte war sicherlich Zertifizierung. Also (platform) ist schon seit vielen Jahren world data center und hat auch ein, zwei andere labels gehabt, die ich grad nicht mehr weiß und ist jetzt ganz neu, weil das wurde ja auch geändert jetzt in call trust Ziel Zertifikat.	
Interview 5		Und das heißt also, da werden so grundsätzliche Fragen, vieles von dem, was Sie mich grade gefargt haben, wird da auch abgefragt. Nachhaltigkeit und also wie funktioniert das , wie ist das strukturiert, kommentiert und das wird da abgefragt und danach wird das Label vergeben, so dass die Leute eben auch das Vertrauen haben, dass hier ein nachhaltiges Repositorium ist, wo die Daten mindestens nach DFG Richtlinien zehn Jahre vorgehalten werden und eben nicht verschwinden.	
Interview 5		Weil das ist natürlich ein großes Thema, wenn Sie ein neues Repositoium aufbauen, die Leute sollen da ihre Daten abliefern. Ja wer weiß denn, ob das Ding in fünf Jahren ncoh existent ist. Da hat man eienn riesen Terz und dann ist das Zeug natürlich weg.	

Interview 5		Haben wir alles schon gesehen, also ist alles nicht neu. Und wie kann man sicherstellen, dass grade im munestären Umfeld sowas eben Bestand hält und nachhaltig funktioniert.	
Interview 5		Da wird auch die NFDI ganz massiv drum kämpfen müssen, deshalb sind die auch durchaus interessiert, dadrin sozusagen Spieler zu haben, die eben Vertaruen in der community haben. Datenmanagement das ist Vertrauenssache. Das ist weniger technisches oder sonstiges. Also das ist Vertrauenssache.	
Interview 5	80:04:00	Wenn jetzt jemand seine Daten, also ok, das sind nicht seine Daten, aber es sind öffentlich geförderte Daten und damit öffentliche Daten aber gefühlt seine Daten, also Ihre Daten. Also Sie haben ja jetzt auch das Gefühl mit den ganzen Interviews, die Sie machen, das sind Ihre Daten. Ne ist es eigentlich nicht. Sie werden öffentlich gefördert, also gut jetzt (work) ist noch so ein DIng dazwischen. Aber weil ja eben die Förderung, ich vermute mal Sie kriegen kein Geld dafür (lacht)	
Interview 5			Richtig.
Interview 5		Genau. Ne genau. Da wäre es jetzt tatsächlich so, das wären jetzt wahrscheinlich sogar wirklich Ihre Daten. Könnte man jetzt mal so juristisch drüber philosophieren. Also weil natürlich in dem Sinne sind Sie ja nicht bezahlt, also nicht öffentlicher Hand, damit ist es jetzt wahrscheinlich sogar noch tatsächlich Ihre Daten	
Interview 5			Sie sind ja mein Interviewpartner.
Interview 5		Ja genau, also in dem Moment, wo es jetzt um eine Doktorarbeit gehen würde und Sie zum Beispiel durch die DFG gefördert würden, dann wären ja sozusagen das Geld, was Sie bekommen, um Ihre Leistung zu erbringen wären erstmal öffentliche Gelder und den Dienst, den Sie machen wären dann öffentlich und und und und dann wären es nicht mehr Ihre Daten, sondern es wären im Prinzip die Daten der Öffentlichekit ne.	
Interview 5		Und abgesehen von Master- und Bachelorarbeiten ist es - ich weiß gar nicht, wie es an der Stelle ist, müsste man eigentlich klären...naja Sie haben gar keinen Vertrag ne....- ach schwierig, ok gut ich sage ja Jura. Und... aber in dem Moment wo man wirklich im normalen Forschungsbetrieb Daten erzeugtwerden mit hier Schiffe, Ausfahrt, mit sonst was. In dem Moment sind es eigentlich nciht mehr persönliche Daten, sondern es sind öffentliche Daten aber es fühlt sich jeder wie seine eigenen Daten und um die sozusagen rauszugeben müssen sie auch Vertrauen haben, dass die Daten entsprechend an den Stellen landen und mit Muratorien versehen zum Beispiel, mit Zugänglichkeitsschutz und und und.	
Interview 5		Grade auch wenn es um persönliche Daten geht, Datenschutz und so weiter, dass alles eingehalten wird. Das geben sie nicht Hinz und Kunz. Da muss schon etablierte vertrauenswürdige Repositorien da sein. Deshalb hat man die NFDI ja auch so gebaut, obwohl es am Ende eine NFDI werden soll, dass es eigentlich domain spezifische Konsortien gibt, dass sie sozusagen die Verankerung in der Domaine haben und damit sie das Vertrauen in der Domaine haben.	

Interview 5	82:02:00	Sonst würden Sie keine Daten kriegen und deswegen braucht man eben auch so Sachen wie (platform) oder Repositorien, wie eben die Museen, die halt einfach eine Reputation haben, dass sie A) schon lange existent sind, vertrauenswürdig sind und das die Leutre ihre Daten dahin auch wirklich geben würden, also einen Mehrwert draus ziehen und nciht nur irgendein Grab wo irgendwann in fünf Jahren von irgendeinem Publisher gekauft wird und dann die Daten verkauft werden. Ist ja auch immer ein Thema.	
Interview 5	82:51:00		Welche Herausforderungen gibt es denn beim Austausch der Daten über die Plattform?
Interview 5		Oh hm, Formate, also die ja die Standards, also es gibt leider so viel Standards, dass es am Schluss ein Grauß ist. Und der typische Ansatz ist ja, wenn ich jetzt drei verschiedene oder fünf verschiedene Standards habe dann sagt einer so ich mach jetzt, ich, ich mach jetzt einen Standard und zum Schluss hast du dann einen sechsten Standard. Also es ist immer das selbe Thema, also der Austausch von Daten, Etrovalität, heißt halt man muss sie im Prinzip normalisieren, man muss sie auf einen gemeinsamen Standard bringen und dann letzendlich um Daten...	
Interview 5		also es fängt bei einfachen Dingen an, wie Temperaturen, wenn sie in Fahrenheit oder Grad Celsius abgelegt wird. Wenn Sie die Zahlen miteinander verschneiden am Schluss. Da kriegen Sie nur Schrott raus. Das muss natürlich normalisiert werden, eben auf oder es muss zumindest klar beschrieben werde, was drin ist, ne. Also der gesamte Datenaustausch, Metadaten Standards das ist ein riesen Thema, also da gibt es sicherlich viele Ansätze, auch semantische Sachen, Ontologien, Controll Vocabularies, auch damit wird sich die NFDI noch und nöcher beschäftigen.	
Interview 5		Grade wenn es über Domain Grenzen hinweg geht, wird das richtig aufwendig. Das ist.. Also das ganze ist eine riesen Herausforderung und müssen wir dran arbeiten, ist noch nichts wo ich sagen kann,das haben wir, also innerhalb der community geht das immernoch aber sobal es dann die community Grenzen verlässt, dann hat einer, also jeder hat seine 600 Felder, die alle anders benannt sind, obwohl die Inhalte ähnlich sind und da muss das erstmal abgeglichen werden. Also Mapping ist ein Thema aber man kann sich zu Tode mappen. Ja hm. Fragen Sie in zwei Jahren nochmal	
Interview 5	84:52:00		Wie stellen Sie denn sicher, dass die Nutzer von (platform) die Daten wissenschaftlich korrekt benutzen?
Interview 5		(lacht) Gar nicht. Das können wir nicht.	
Interview 5			Ok.

Interview 5		Das ist ne fiese Frage, weil das nämlcih eines der großen Kritikpunkte, eines der großen Themen open access und open data ist ne. Dass sozusagen die Wissenschaftler immer wieder mit dem Argument kommen " Ja wenn ich jetzt meine Daten einfach so zugänglich mache, könnten sie andere ja missbrauchen." Ja was heißt denn jetzt genau missbrauchen? Also heißt das jetzt, dass man daraus Schlüsse zieht, die man ursprünglich nicht hätte rausziehen wollen?	
Interview 5		Aber was ist Forschung? Forschung ist ja eigentlich das Spiel mit Daten, um daraus neue Erkenntnisse zu gewinnen. Und was ist jetzt richtig und was ist falsch? Was ist Missbrauch? Also Missbrauch... ja gut es gibt klaren Missbrauch.. personenbezogenen Daten bei Versicherungsfirmen, die hinterher deine jährlichen Beiträge erhöhen, ist vielleicht auch noch nicht Missbrauch, aber es ist zumindest ein Gebrauch, den ich vorher nicht authorisiert haben.	
Interview 5		Also Missbrauch bedeutet ja, dass ich vorher festgelegt habe, für was bestimmte Daten gebracuht werden können. Das sind antürlich personenbezogene Daten, das prime vorm consint, also das ganze Thema für was dürfen wir Daten, die aus der Tumorforschung von Probanden benutzt werden. Dürfen die auch der Pharma Firma zur Verfügung gestellt werden, also rein fürs wissenschaftliche. Das ist ein großes Thema im Datenschutz oder im ganzen personenbezogene Daten.	
Interview 5		Bei Umweltdaten, ich wüsste nicht, wie die missbraucht werden können. Ok, es gibt einen Fall, oder es gibt vielleicht wenige Fälle, wo sie missbrauchtwerden können. Wenn wir zum Beispiel Daten über seltene Pflanzen hätten, Rote List Arten. Aber dann ist natürlich klar, dass wir diese Daten, also die Situation hatten wir grade gehabt, die hatten wir sogar gestern, genau.	
Interview 5		Da ging es um geologische Daten. Wenn wir geologische Daten aufbewahren bei uns, dann sind die natürlich potentiell interessant für die Ölindustrie zur Suche als Battemetrie Daten, also Daten, die zum Beispiel Meeresgrund zum Beispiel mit Sonografie abfahren.	
Interview 5		Da kann man neue Lagestätten finden - Öl, Gas, Kohle. Da ist jetzt die Frage, also das ist jetzt kein Missbrauch in dem Sinne aber theoretisch, wenn man jetzt sagt wenn die Industrie diese Daten, die mit öffentlichen Geldern gemacht werden, missbraucht, um hinterher daraus ihren Profit zu generieren und vielleicht das letzte Ölfeld in der Antarktis anzubohren und da die Umwelt kaputt zu machen, dann kann man natürlich von Missbrauch reden.	
Interview 5	86:28:00	Sowas würden wir jetzt zum Beispiel nicht öffentlich stellen. Genau wie jetzt zum Beispiel eine Rote Liste mit, eine Liste mit seltenen Pflanzen oder... oder rote Liste Insekten. Sowas würde man jetzt zum Beispiel nicht öffentlich stellen. Aber es gibt da Mechanismen, gerade beim Thema Diversitätsdaten, dass man sie mit einem künstlichen Rauschen versieht. Das heißt dann sieht man, ok in dem Gebite..keine Ahnung..so und so...gibt es vielleicht diese Arten, aber man gibt nicht die GPS Position auf den Meter genau.	

Interview 5		Ähm...also, wenn Sie jetzt strikt fragen, wie stellen wir sicher, dass a) solche sensitiven Daten, jetzt nicht im Sinne von personenbezogenen Daten, aber sensitiv im Sinne von, könnte jemadn etwas daraus machen, um die letzte Rote Liste Art abzupflücken oder den letzten Tiger zu erschießen. Solche Daten würxden wir natürlich nicht öffentlich stellen, sondern gegebenenfalls halten aber dann mit einem Sperrvermerk versehen, so dass die Leute nachfragen müssen und dann wäre tatsächlioch die Frage und das haben wir gestern tatsächlich nur anüberlegt.	
Interview 5		Man müsste im Prinzzip ein internes, eine Art Gremium schaffen, was darüber entscheidet, wer die Daten sehen darf und wer nicht. Also wer frägt an? Das ist nciht unüblich. Das ist im medizinischen Bereich völlig normal, dass man Anträge stellt, um bestimmte Daten zu bekommen und dann entscheidet sozusagen ein Gremium darüber, ob man Zugriff auf diese Daten bekommt. Also ist der authorisiert das zu machen.	
Interview 5		Also ist das ein weltberühmter Tumorforscher, der genau diese kohorten Daten haben will. Ja. Ist es, ich sag mal hart gesagt, irgendein Spinner, der meint er muss jetgzt daraus irgendwie irgendwas ableiten.	
Interview 5	88:21:00	Da würde man überlegen..naja.hm..also... aber das ist dann nciht eine Entscheidung der Datenbank. Das ist dann im Prinzip die Entscheidung eines Expertengremiums, das das tut. Also wir würden es erstmal nicht öffentlich machen. Wir würden gegebenenfalls, dann jemanden befragen und sagen würden wir das tun oder nicht. Aber das sind tatsächlich alles sehr sehr gute Fragen, die wir tatsächlich, weil wir damit wenig Erfahrung haben bisher.	
Interview 5		Für uns, Umweltdaten waren alles öffentlich, weil unsere Prämisse ist öffentlich, weil hm.. aber sowas wird kommen und es kommt. Im Bereich sozialwissenschaftlicher Daten haben wir es bereits, im Bereich Diversitätsdaten ist es nicht unüblich. Jetzt wie gesagt, die Anfrage gestern, geologische Daten, Öl und Gasfundstellen, die man daraus ableiten kann. Das sind wirklich Themen, die wir in der NFDI besprechen müssen.	
Interview 5	90:00:00	Mit Leuten, die da wirklich Erfahrung haben also im Thema Datenschutz von Datenschutz im weiteren SInne halt eben Erafhrung haben und da können wir von den Mediziniern halt durchaus einiges lernen. Wie lösen die das? Welche Mechanismen haben die? Meinen die geht das bis zu safe rooms, wo Leute ohne alles am besten nackt rein gehen müssen, um praktisch nur Daten anzuschauen und aber nichts mitnehmen dürfen. Und solche Sachen. So weit wollen wir es jetzt nicht treiben aber ... ich sag mal... wir machen uns Gedanken drum, aber im Moment nicht so prikär, als dass wir da Handlungsnotwendigkeiten gesehen haben. Das wird sich jetzt mit NDCI ändern.	
Interview 5			Gut. Dann komme ich jetzt auch schon zu den letzten drei Fragen.
Interview 5		Oh mein Gott schon?	
Interview 5			Jaaa. Es sind ja nun auch schon anderthalb Stunden um, ne. (lacht)
Interview 5		Könnte man so sagen ja (lacht)	

Interview 5			Das besondere an (platform) ist ja , dass sie im wissenschaftlichen Feld eingegliedert ist. Wie glauben Sie beeinflusst die wissenschaftliche Kultur die Nutzung der Plattform?
Interview 5	91:28:00	Eine schwarze Spezies im Hintergrund.	
Interview 5			Ach mein Kater ist aufgewacht.
Interview 5		lacht	
Interview 5			Naja muss er jetzt warten.
Interview 5		Genau… jetzt habe ich auf den Kater geguckt muss ich gestehen.	
Interview 5			Ich wiederhole die Frage. Genau also wie beeinflusst die wissenschaftliche Kultur die Nutzung der Plattform?
Interview 5		Also es sind in der Wissenschaft denk ich mal, also dadurch, dass wir aus der Wissenschaft kommen relativ gut verankert. Das heißt also beeinflusst die Nutzung würde ich sagen. Also die Wissenschaft hat generell mehr Vertrauen zu einer Einheit, die auch im wissenschaftlichen Bereich angesiedelt ist als jetzt zum Beispiel im kommerziellen Bereich. Wobei das stimmt nicht ganz, weil 99,99999 Prozent aller Publikationen fließen über kommerzielle Anbieter.	
Interview 5		Ja... aber ich Bereich der Daten, ich denke mal, das spricht zum Beispiel auch gegen Kommerzialisierung, was auch mal im Raum stand. Ich glaube wir würden relativ viel Vertrauensverlust haben, wenn wir jetzt anfangen würden für Datensätze Geld zu nehmen oder überhaupt das ganze in den kommerziellen Bereich zu schicken, so dass man zum Beispiel also für den gesamten support, nicht für die Daten selber, aber für den support letztendlich Geld nimmt.	
Interview 5		Das ist im nicht-wissenschaftlichen Bereich durchaus nicht unüblich, wie gesagt mit Silva haben wir so ein dual Modell gefahren. Ich hab ne Firma ausgegründet (year/number), die sich um die ganzen kommerziellen Anfragen gekümmert hat und aber für den wissenschaftlichen Bereich haben wir immer das ganze Wissenschaftliche frei gehalten letztendlich ne. Also ich denke, in der Wissenschaft verankert zu sein auch mit dem Puls der wissenschaft zu leben und auch zu verstehen, was die wollen, hilft uns letztendlich die Datenbank weiterzuentwickeln auch in den Bereichen , wo es die Wissenschaft auch braucht.	
Interview 5	93:22:00	Auf der anderen Seite ist glaube ich das Vertrauensverhältnis zwischen.. Zwischen der Wissenschaft und uns größer zumal wir ja auch… also ich komme ja eigentlich auch aus der Wissenschaft und auch jetzt also meine Gruppenleiter die koommen auch alle aus der Wissenschaft. Also das ist... ich will jetzt nicht sagen das ist wie eine große Familie, weil das ist die Wissenschaft eigentlicha uch nicht aber es ist zumindest irgendwo im ähnlichen Umfeld. Das ist eben nicht der Bruch zu nem kommerziellen Anbieter, ne.	
Interview 5	93:50:00		Welche Entwicklung sehen Sie denn dann für (platform) konkret in den nächste Jahren?

Interview 5		Ja also so ganz konkret, wie gesagt da sind wir wie vorhin.. Dokumenationen, Ausbau in Richtung Data Science, bessere Zugänglichkeit der Datenprogramme, andere Zugänglichkeiten der Daten, insgesamt wie der Teil Dokumenationen, klare Abgrenzkriterien, was wir nehmen und was wir nicht nehmen, welche Workflows wir drin haben, klarere Strukturen auch im Bezug auf wo wir uns beteiligen. Seh ich jetzt auch wieder, wenn Anfragen auf uns zu uns kommen, müssen wir Abgrenzkriterien haben, was wor nehmen und was wir nicht nehmen.	
Interview 5		Wenn wir uns entscheiden, an was für Projekten wir teilnehmen, müssen wir im Prinzip wissen, wor wir teilnehmen und wo wir nicht teilnehmen. All das muss im Prinzip, ich sag mal, noch etwas oder besser strukturiet werden und klarer kommuniziert werden, so dass wir eigentlich, so dass für die Leute klar ist, was (platform) eigentlich wirklich ist am Ende des Tages. Also, was wir tun und was wir nicht tun.	
Interview 5		Und ich glaube das hilft dann auch beiden Seiten Missverständnisse zu vermeiden, dass nicht Erwartungshaltungen geweckt werden, die wir dann nicht erfüllen können. Und auf der anderen Seite, dass wir nicht mit Dingen überlastetwerden, die wir nicht leisten können an der Stelle. Ich will jetzt nicht ketzerisch sein, aber früher wurde häuffig je gesagt zu Dingen und dann passierte nichts, weil es einfach nicht leistbar war und es ist aus meiner Sicht besser nein zu sagen, als ja und sich hinterher sozusagen, dass nichts passiert.	
Interview 5		Das ist..ja (räuspert) ja. Aber das war auch.. das ist nicht negativ. Man muss ja auch, wenn man in den Markt rein will, muss man im Prinzip auch erstmal eine möglichst offene Strategie fahren und erstmal viel nehmen und irgendwann mal hat man halt die Chance wenn man, wenn sozusagen der Markt sich dreht und man eher mehr Anfragen hat, dann kann man selektiver werden. Und das sind eigentlich die konkreten Ziele an der Stelle.	
Interview 5	95:47:00	Aber das sind alles an der Stelle Management Ziele. Auf der Ebene Arbeitsebene ist es Austausch des Kurationssystems, bessere Metadatenstrukturen, Stationslisten einpflegen. Also ganz pragmatische Dinge, die so im täglichen Betrieb laufen ne.	
Interview 5			Und wovon hängt dann der Erfolg in zukunft ab?
Interview 5		Naja also ich denke schon, dass wir es schaffen klar einen Mehrwert für die Data Science zu schaffen. Also der Kritikpunkt, dass wir ein gutes Datenrepositorium sind, wo Daten sehr gut abgelegt werden können, und auch zitiert werden können und auch wieder gefunden werden können. Also findable und accessable glaube ich, das sind wir.	
Interview 5		Aber wenn ich die Nachnutzbarkeit und die generelle Frage, ob big data science oder data science den großen Mehrwert hat. Das ist nicht nur (platform). Das ist sowieso offen. Also jede Rede von big data und big data science. Aber was ist denn nun der tägliche Mehrwert? Und das muss auch für unseren Berecih irgendwann mal gezeigt werden. Da brauchen wir success stories, da wollen wir sehe "aha.	

Interview 5		Das sind Dinge die hätte man, sage ich jetzt mal, nicht ohn (platform) tun können. Ohne NFDI. Ohne, und das ist auch die Aufgabe übrigens in NFDI, die ich sehe. Die muss zeigen, dass am Ende das ganze Datensammelgut auch einen Mehrwert liefert und nicht nur die Festplatten voll macht und ne Haufen Geld kostet. Sondern wirklich einen Mehrwert für die WIssenschaft, dass daraus wirklich bessere Forschung wird ne.	
Interview 5		Und ich möchte im Moment behaupten, dass es in der Wissenschaft noch wenige echte success stories gibt, wo man sagt "ok aus dem gesamten Daten, riesigen Datenströmen wurde jetzt etwas erzeugt, was wir so in der Form nicht gesehen hätten, wenn wir diese riesigen Datenströme nicht hätten. Klar, da kommt das ganze Thema Machine Learning, Artificial Intelligence, was damit ja alles, was im Moment ja unglaublich gehyped wird, rein. Aber alle diese Methoden brauchen qualitativ hochwertige, saubere Datensätze und die müssen wir leifern.	
Interview 5		Und das ist widerum die Aufgabe von (platform) und NFDI. Das am Ende zu liefern. Also nicht shit in , shit out, sondern wirklich qualitative hochwertige Datensätze und deswegen, wie ich vorhin schon gesagt habe. Mir geht es nicht um den letzten Datensatz, sondern mir geht es darum, wirklich qualität zu liefern, damit damit dann halt eben diese modernen techniken befeuert werden können und somit dann auch tatsächlich (hoffentlich) einen Mehrwert für die Wissenschaft liefern.	
Interview 5	97:26:00	Wissenschaft ist natürlich auch wieder das Ding. Wissenschaft heißt für mich auch Innovation, heitß auch für die Wertschöpfungskette, heißt auch für die Industrie. Weil am Ende des Tages verbrate ich hier grade Steuergelder. Die müssen ja bezahlt werden von der Gewerbesteuer zum Beispiel. Also von Firmen. Und die tragen auch einen großen Teil dazu bei, dass wir im Prinzip in der Freiheit der Forschung überhaupt machen können. Das heißt wir müssen am Schluss natürlich auch der Forschung... ne der.. der Industrie einen Mehrwert bieten.	
Interview 5		Jetzt nicht direkt, indem wir denen irgendwelche Datensätze verkaufen, sondern, indem wir denen letztendlich Sachen bieten und Wissen schaffen, die dann daraus eben neue Innovation schafft. Deutschland ist nunmal kein Rohstoffland. Also, wir werden die letzten Kohle Dinger auch irgendwann sicht machen. Logischerweise.	
Interview 5	98:54:00	Und irgendwoher muss ja letztendlich der Mehrwert in der Wertschöpfungskette dann kommen. Und das geht eigentlich nur da irgendworum, ja.. (lacht) Hoffe ich mal. Vlt schaffen wir es ja auch mal irgendwann die Energiewende hinzukriegen. Also, ich bezweifle es ja mit unseren Politikern, aber das ist ein anderes Thema (räuspert)	
Interview 5			Sie haben ja nun schon häufig die Nachnutzbarkeit der Daten angesprochen. Da habe ich nochmal eine kleine Nachfrage. Wie schätzen Sie denn die Nachnutzbarkeit der Daten ein?

Interview 5		Das ist ne schwierige Frage (räuspert). Also ich würde sagen verbesserungsfähig. Es hängt ein bisschen davon ab, welcher Nutzer da ist. Der Standard Nutzer erwartet, dass die Daten so aufbereitet sind, dass er die nur noch in sein Tool einhacken muss und dann läuft das los. Der wird da kein Glück mit haben.	
Interview 5		Man muss schon -und das geht jetzt eben wieder in den Bereich Data Science oder auch Kenntnisse haben, der Nutzer muss sich Grundkenntnisse in der Programmierung aneignen. Also er muss zumindest Skriptsprache irgendwo können. Python oder irgendwie sowas, Java, um auch selber programmatisch an die Daten ranzukommen und auch programmatisch die aufzuarbeiten. Also das ist keine Mikrowelle, die man sich irgendwie in die Küche stellt, einschaltet und läuft los, sondern das ist schon, das ist ein komplexes Thema.	
Interview 5		Da muss man sich mit dem Thema Daten auseinandersetzen. Deswegen: Je nachdem , wen Sie jetzt befragen. Die einen würden sagen (platform) ist völlig nutzlos für mich, weil ich kann nicht auf der Website mir zehn Datensätze aus fünf verschiedenen Quellen zusammenklicken und daraus im Prinzip die Grafik erzeugen, die ich brauche. Der andere wird sagen, das ist für mich nicht relevant, weil ich möchte sowieso meine eigenen Grafiken erzeigen mit meinem eigenen R oder Toll und ich will mir nur die Datensätze runterholen und alles andere mache ich selber.	
Interview 5		Also Nachnutzbarkeit ist ein großes Thema. Also wir wollen die Daten schon und wir denken, ja also rein vom Kern her, sind die Daten schon so aufbereitet und mit Metadaten versehen, dass sie eiegntlich nachnutzbar sein sollten. Nur im Augen des Betrachters ist die Erwartungshaltung an der Stelle oftmals eine andere. Also die Nutzbarkeit ihres Telefons ist darin: ich kann jemanden anrufen. Ja, es ist ne valide Sache.	
Interview 5		Dahinter muss aber die ganze Netzinfrastruktur stehen, da muss im Prinzip all das alles stehen. Und theoretisch könnten Sie auch jemanden anrufen, indem Sie jetzt nicht ein Smartphone benutzen, sondern einfach nur den Klingeldraht und dann lautsprecher dran machen, wie beim Mikrofon.	
Interview 5	100:09:00	Ja also, ich sag mal so. Ich glaube schon, das ist verbesserungswürdig. Dabei bleibe ich. Das ist klar. Und die Frage ist jetzt, wie viel auf der Seite der Nutzer verbessert werden muss, dass der einfach die Kompetenzen erhält, sich selber auch, also die Ausbildung erhält, dass er diese Daten dann auch entsprechend nutzen muss und wie viel wir jetzt letztendlich auf unserer Seite verbessern müssen, damit die Daten leichter nachnutzbar werden.	
Interview 5		Das ist noch ein Prozess, der muss, ja, der muss noch durchlaufen werden. Dazu brauchen wir aber use cases. Das heißt dafür brauchen wir im Prinzip eigentlich so eine Art Testnutzer, die uns letztendlich helfen zu sagen, an der Stelle müsst ihr das jetzt noch so und so verbessern, damit wir damit besser umgehen können. Und das ist eben eins der Ziele im gesamten Thema Data Science und Aufbereitung und Zur Verfügung Stellen von Schnittstellen, wie zum Beispiel Jupiter Notebook und solche Sachen.	

Interview 5		Damit man dann einfacher an die Daten, schon programmatisch, aber einfacher rankommt mit Framework, dass die halt nicht so komplex sind, wie... - Also erst vor kurzem kam einer zu mir und meinte er braucht Zugang auf MioSKL, also auf die Datenbankstruktur, also MioSKL läuft dahinter. Da sag ich, ne das brauchst du nicht. Das willst du auch gar nicht. Du brauchst ne Restschnittstelle, ne API, wo du drauf zugreifen kannst und die haben wir. Achsoo aaah jap.	
Interview 5	102:28:00	Also manchmal ist es halt so, dass es da ist, aber die Nutzer nicht wissen, dass es da ist und entweder völlig zu kompliziert denken oder zu simpel denken.Da ist wieder Thema, Kommunikation, Dokumentation, und und und.Das... Da ist es eher verbesserungswürdig, ja. Also Nachnutzbarkeit hat ganz viele Aspekte, wie kann man Daten besseraufbereiten, Dokumentation besser aufbereiten, wie kann man Kommunikation besser verbessern. Das sind alles Punkte für Nachnutzbarkeit glaube ich. Und auch die Kompetenz der Nutzer verbessern.	
Interview 5	103:51:00		Haben Sie sonst noch was zum Thema Erfolgsfaktoren?
Interview 5		Och	
Interview 5			(lacht)
Interview 5		Erfolgsfaktoren… Naja Vertrauen, mit den Leuten reden. Das merkt man immer wieder jetzt. Offen sein für Neues, Innovation. Aber auch nicht zu schnell. Die Leute wollen nicht alle fünf Minuten ne neue Datenbank haben und neue Struktur, sondern sie wollen sich darauf verlassen, dass etwas funktioniert und wie hoch die Funktion ist. 24 hours/7 ist auch ein großes Thema. Stabilität. Also dass man einfach sagt, die Webpage läuft und sie können zu jeder Zeit drauf zugreifen.	
Interview 5		Wenig Ausfälle. Einfach so Basisfaktoren. Im Prinzip das gleiche, was Sie eigentlich erwarten von einem Service. Sie ertwarten, dass Skype funktioniert. Sie schalten ein und das Ding läuft. Sie wollen weder jetzt im Backbone mit dem Rechenzentrum kommunizieren oder mit Ihrem Internetanbieter wegen Ihrer Internetleitung, noch mit Logitech über Ihre Webcam diskutieren oder Ihr Headset. Sonder das muss einfach laufen.	
Interview 5		Das ist eben ne große Ingenieursleistung, die von nem Prototypen bis hin zu einem echt funktionierendem System gemacht werden muss und da steckt wahnsinnig viel arbeit drin. von der Idee eines Autos bis zu einem fahrenden Objekt, das jeden Tag bei jeder Temperatur von früh bis spät durch die Lande zieht.	
Interview 5		Das ist... und zwar von jedem eingeschaltet werden kann. Das ist eine ganz große Sache. Da muss man als Service Provider hin. Und das ist nicht einfach. Grade im akademischen Umfeld nicht, wo Sie Leute mit ganz vielen Ideen haben. Nicht jede davon (räuspert) muss immer umgesetzt werden. (lacht)	

Interview 5	105:26:00	Also Agilität und Stabilität sind die zwei Punkte, die müssen immer ausgewogen sein. Also man muss innovativ sein, aber andererseits auch die Stabilität haben, dass die Leute nicht alle fünf Minuten: Das Ding funnktioniert nicht, weil wieder grade einer irgendein Softwareentwickler beschlossen hat e, er muss die neuste Framework einbauen, was zwar toll ist von der akademischen Seite, aber leider dem Nutzer keinen Mehrwert bringt, außer dass das Ding down ist. Das macht keinen Spaß	
Interview 5			Ja super. Das wars jetzt auch schon von meiner Seite mit den 26 Fragen. Also, ja waren echt sehr schöne Einblicke.
Interview 5		Haben Sie denn jetzt Zeit das alles auszuwerten? (lacht)	
Interview 5	106:08:00		Jaaa…(lacht) Also zuerst werde ich es ja transkripieren. Ich habe hier mal noch ein paar Pubkte für die Statistik, die ich hier mal mit aufnehmen.
Interview 6	Time	Interviewee 6	Interviewer
Interview 6	05:26		So I would say for my interview now, we should focus on the (platform). Okay, so not to make it too complicated and also with the time constraints, it's better just to focus there.
Interview 6	05:38	Yeah, just just one thing that it's very important though, because when you say that, because the (platform) one of the attractive attractions, attractions and different about the (platform) is that it provides this platform for other for any university I mean, there are many (platform)s in in (region), for example, that are used for supporting research. choosing their own university, right, and several countries. So	
Interview 6	06:06	I think that's a very important part of.	
Interview 6	06:09	I mean, I don't know how you want to compare it. But I think that that's a very part of the fact that it's built the harbor, the rivers is built on top of this open source (platform) software. So it's, it's what makes it different than other repositories.	
Interview 6	06:29		So what is the history behind it?
Interview 6		So we started here at the Institute for quantitative social science here, (town), build it about (year) years ago, almost no. And with the one it was, initially, the main focus was for social science, quantitative data. So a lot of data sets that were tabular files that might have Well, a bunch of columns So the rows are observations about anything something related to election something related to, to social behavior economics, so many, many different well, psychology or so, the Woodrow would include well that would be modularly like these even though it accepted any type of data file and data format that there was a lot of emphasis in these social science quantitative data sets right.	
Interview 6		But But then later well any any was initiated because there was the demand between researchers to be able to share the those data sets or to make that data said that they were working on accessible to others and to either believe well, validator is a result of reuse it and initially here that was done Sort of men one right that people would come to harbor data center to, to get, for example, a tape.	

Interview 6		Oh, these guys so too, with the data set, because they needed that data. So we started creating it as a repository to make that it easy and enable others to, to deposit but one very important part of it was that to build incentives so that people that were deposed people would want to deposit so that if they deposit they get credit for it is their data set, they still own it, they still can manage it and him and decide who can access it.	
Interview 6	06:32	And so they so they still have control of the data set and they still and they have a citation in the data said that the where they are the data authors, the authors of that,	
Interview 6	08:54		and what is the core offering of (platform) so it's
Interview 6		And it's the software I mean, that does the (platform) as as the product is a software to build a data repository that kinda that allows to deposit data with a lot of extensive metadata. So that a generate automatically a citation, right? For the data said that eight supports versioning of the data set it, it supports not only the core metadata, but also allow custom metadata on top that additional metadata that you might have for the for the year.	
Interview 6		They simply know for this type of data to parse any data type, any data type, you know, it has a deer access well at different levels of accessing data so it could be completely open, but you could also allow for people to To have to register you could restrict it. So the data files have to be well addressed. The data user will need permissions to access the data of house right?	
Interview 6		You there is a lot of different levels of setting this up so that that you can request access or not. The metadata once the data set is published, the metadata has to be open. So there is discoverable it supports very well the fair principles, so for fine findability, accessibility interoperability under usability, and am well enough to present a whole set of API's.	
Interview 6	09:01	So the application programming interfaces so that other tools, Guru, Guru search for the data,	
Interview 6	10:54	get the metadata access the data sets, and so	
Interview 6	10:58	so is really the built all the features that are necessary to have a repository for sharing research data, and in support with with fair principles and so	
Interview 6	11:11		and what a vision I are pursuing.
Interview 6		Oh, the vision. So that was the from the initial vision. First of all, let me clarify that even though we start them with social science, now it's used across all disciplines, so biomedicine, astronomy, astronomy and astrophysics and physics and chemistry, law business, you know, across for everything of your culture and so, so the, but the vision is that I'm facilitating making data research data accessible for two main things.	

Interview 6		One is verifying results that are published in a in a in an article right, and if there is a claim that you use that data code and some code to to get the results that you can point and you can say it right or have a reference to the data set in the repository and make sure that that will always proceed that the reference will be will work no matter no matter what and that so that you provide long term access to that data.	
Interview 6	11:15	The other The second is that not only for is for verifying and reproducibility right of previous results, but also for reuse so that if you for your research project, you put a lot of effort to generate a new data set. And it's a data set that can help understanding some some aspect of science or improve some other research that somebody else can use it and continue their research using your data set.	
Interview 6	13:01	Those are those are the main things.	
Interview 6	13:04		So my work is about success and success factors. How do you define success or a successful platform?
Interview 6		Yeah, that's a that's a good question, I think in in the case of an open source platform, so again, when when we talk about the (platform) the open source software, or that our policy story, right, so for the open source software, success is is is that you have a community that are very active, engaged, that they're driving the new features and contributing to make the project better.	
Interview 6		So when, when you there are actually have one project that is defining some metrics of success of open source software, but a lot of it is about having making that product Very, very easy to use for others and may have a very engaged community.	
Interview 6		So that's, again, for open source software for repository itself like hardware, the rubbers, right? I think the success is the fact that there is the number of data sets that are, are shared, right that the quality of the data and the how much that data is reuse. So, for example, download citations to the data.	
Interview 6	13:13	And so, so all these, I mean, the that that crowd was the success rate in this case for the repository, because it's that it is used not only the quantity but also quality in terms of metadata that it makes it that that it's easy to reuse by others.	
Interview 6	14:55		Because you mentioned the quality. How do you ensure The quality of the data sets.
Interview 6		Yeah. So So there are different options in it and different data repositories might have from policies for that. Part of it is that it was the features itself of data (platform) making sure that there is already sufficient metadata. The depositor can add, in some cases they have to add, but better than describe the data set.	

Interview 6		The other thing is that, for example, adding some features that allow already to extract metadata automatically from the data set, so that it makes it easy. Well possible for for others to identify that well, not only accepting the meta data, but also changing the format to other formats that other people can use. So, so that if, if you upload a statistical data And that could be in SPSS.	
Interview 6		So, an Excel file that it takes to get all the information about the data set and the variables of every table and calculate the summary statistics descriptive statistics about those variables and so, and this adds to the quality. So, it informs other users more about what that what is the content of that data set right.	
Interview 6		So, so part of it is with technology right for with the right features to to improve the quality and providing sufficient information or creating that information automatically. Some other part of it, you cannot do only with technology. So so that's why there are some facilitating curation services and making sure that for The collections that are managed that way that they they have their the data sets are clean. They they're well documented.	
Interview 6	15:02	They are, yeah. Then described well and in formats that can be reused and so related, know that. So we work and the (town) the (platform) was a lot of journals that required the authors to deposit the data in a positive way like (platform). And then in those cases, the, the quality is part of the data, the review of the paper and the verification that the data said and the gold provides the results of the paper	
Interview 6	17:49		related with data quality is trust. So how do you ensure trust in the data
Interview 6		I'm trusting the data or in the room auditory, I think they're connected. So, for the delay is a critical mass of water what it goes into a repository is in general good quality, there is more trash already in the you what you can find there right on that. One.	
Interview 6		So, scarper the river, there is a lot of critical mass of very well data sets that are associated with papers that are good quality, so that that helps them to, to have tasks in the repository itself. In terms of the data, the individual data sets, though. I this is something that the community at the end decides because of how good that data set is how, how impactful how useful it is for further research.	
Interview 6		I don't think that It can be decided ahead of time. Yeah, time also besides that, but, uh, I think that for a lot of the thing about trust is the the transparency and the describing all the context about the data set.	
Interview 6	17:57	So the more you have the data set with the code and and well described well documented and so, there is more ties because there is more information that you provide that supports the results of	
Interview 6	19:33	scientific study right. So	
Interview 6	19:37		coming to a complete different question, what is the organizational setup behind the how what data was all the data was in general?

Interview 6		Yeah. So, for the database software in general, there is a core team here, a hardware there that provides the development One of the main, most Well, the main features, and then there is a global (platform) community Consortium, it's an organization that I'm one of the CO chairs, and there are two other co chairs.	
Interview 6		There is an international organization that represents the community and and the organization helps prioritize what the community who are, are using the data of our software, right, what what they need from this from the feet, what features they need, what are the what are the best practices on how to use those features and, and how to improve data sharing and data access.	
Interview 6	19:48	And so, so that's one part for the (town) University repository, the a lot of the government so is the we have a collaboration between our institute Institute for quantitative social science here. library, the hardware library and the hardware it information technology so that we it hosts the hardware the (platform) in the cloud and the library provides also support and and together we collaborate to provide a curation services and the support for using the database.	
Interview 6	21:31		How many people are involved?
Interview 6	21:36	So about so about 15 people in the core team here (town), and same broadly, sometimes is more or less depending on we have some research projects that we get grants for and may increase the number of people for those better and In 100, or more that are contributing from the community	
Interview 6	22:07		and what competences to those 15 people have.
Interview 6	22:11	So it's a mix of software engineers, you user interface and user experience.	
Interview 6	22:20	Experts, right.	
Interview 6	22:23	Quality Assurance and then data curation and so data, data science skills that I get information cyan, meta data specialists then and data curation and data management skills. So that's the mix.	
Interview 6	22:45	Quite a bit of software development, obviously.	
Interview 6	22:50		And when it comes to decisions, how do you decide how was the decision process?
Interview 6		Yeah, so again, Have to separate from the software. So the core development team here, when there is contributions from the community, it review by the core here, who is the developers here, (town). And any bits of the code is good quality and it works well and tested well. And it's accepted. Right.	
Interview 6		So in terms of prioritizing what we're working on the features and so we have a community meetings, we have an annual community meetings here hardware, some just an all once a year, right. But there are now also regional community meetings and there was one in Norway for the (region) community that uses a (platform) so that and together with the global (platform) community Consortium, right.	
Interview 6		These are Organization helps them prioritize. We provide a roadmap right from here from (town) about the things that are important to work on based on the feedback from others stakeholders.	

Interview 6		And then in this community meetings and also, we have a weekly call, so every two weeks, we have calls with, from people all around the world to, to contribute into what is important for the (platform) and, and that helps make the decisions.	
Interview 6	22:58	So it comes a lot from the community. repository itself is more of the the collaboration with the library and ID and our group here and the curation team within my group	
Interview 6	24:50:00	in this case,	
Interview 6	24:51:00		so when it comes to responsibilities, and you mentioned that you have quite divided tasks, Is all of them are autonomous
Interview 6	25:02:00		or do you have like a head off? Who
Interview 6	25:04:00		decides? How are the responsibilities distributed?
Interview 6	25:09:00	You mean in the in the the team here (town)	
Interview 6	25:14:00	know there is a manager so I'm one of the CO lead with the Faculty Director here, there is two or two above that there are the KPIs that principal investigators right. So, we lead the vision	
Interview 6	25:29:00	and then there is a program	
Interview 6	25:34:00	project manager, program manager that leads the day to day development. And then there is a data curation manager that leads the curation services and then for the community, we are three co chairs the of these organization that we overview, we overview what	
Interview 6	25:59:00	what is it important to the community.	
Interview 6	26:03:00		And I have one question back. So when,
Interview 6	26:06:00		let's say some people (country) on (region) decide what they want they forwarded to your team. And then you implemented changes
Interview 6	26:15:00		or how
Interview 6		it was no. So no, it's a lot more community oriented. So it's really an open source. Open Source contributions from the community. So so they could be could be two ways. So we track everything in GitHub. So for example, our group might create an issue you have saying this is an important feature that you may be before these guys in one of those calls is every two weeks we have a call and maybe they bring that feature.	
Interview 6		If it's not something that is already entering the GitHub issues is enter, create a new feature a new issue, and that goes into a black backlog that is review and prioritize right together with the group here in the community. What if What, based on how many other people demands it, one wants that. and other stakeholders, some some of the features are funded by grants.	
Interview 6		And we need to do the hard way. But the most important thing and what makes the (platform) so successful now is that not only the core group here, (town) are implementing these features. The groups, for example, in Netherlands are implementing a whole set of features. And they sent also a pull request, you know, right up from GitHub, you can do a port requires meaning.	

Interview 6	26:17:00	You have a copy of the code, you make some changes. And then you ask the people that core team to review those changes to make sure they are good quality and they well tested. And so there are 100 other developers around the world could contribute	
Interview 6	27:58:00		as you just mentioned, The funding and also the product based funding. How is the how our data was funded?
Interview 6	28:07:00	Yeah. So the core team here in hardware is is funded by (town). So the provost,	
Interview 6	28:17:00	the you know, like the better and	
Interview 6		Mindy pundit, then there is about the contributions from all these other groups is that all these institutions of these other universities are contributing some part of it with resources into data (platform) they're paid by their institutions.	
Interview 6	28:21:00	Then there is like about an additional, maybe 30%. Sometimes it's even as much as 50% of additional work we're doing that is funded by research grants from federal agencies are from foundations that are supporting the project is finally additional like for example, now we're working on on Super Embedded reproducibility workflows and containers that have the wrong code. And that's supported, funded by as long foundation.	
Interview 6	29:11:00		So the funding from (university) is a more long term funding.
Interview 6	29:15:00	Exactly.	
Interview 6	29:16:00		Okay. Good. And do you have a strategic position of (platform) /
Interview 6		I want to add one more thing about the funding the the consortium, the community Consortium, every member of that consortium of the disease institutions, the universities there that they also have a data (platform) installation under repository.	
Interview 6	29:20:00	There they are paying to the consortium a fee every year for assistance on some of them. Well, some of the services that facilitate the development of what the community wants and raise money So there is a body this organization has made is funded by the members.	
Interview 6	30:06:00		And you also mentioned before that you also offer some additional services where you also get some fees. Right.
Interview 6	30:13:00	Exactly. Yeah, what additional curation services here?	
Interview 6	30:19:00		So coming back to my question, and
Interview 6	30:24:00		how do you want to strategically position data worse, let's say on the data market.
Interview 6	30:33:00	I'll move on. So it's, it's a critical soda.	
Interview 6	30:39:00	It's one of the very few truly open source community driven platforms to build data repositories. So that that means that it's a free platform that that any university can use. To build a data repository and be able to support fair research data management in the institution	
Interview 6	31:10:00		and when it comes to difficulties and challenges and managing, and that I was,
Interview 6	31:18:00		what do you experience? Well,

Interview 6		right, there has been a lot of challenges in in the last years, but actually, we're in a very good moment now. So it's very fortunate in in Paris because the community has grown so much and it's so engaged and active and participating. Right.	
Interview 6		So now it's not only depends on the group here at (town), but it's also the many many universities are contributing to the success but uh, but in it, I mean it usually the the challenge always continues being that for a lot of them Well, for software to continue being attracted and adapting to new technologies, you need to continue creating new new features and new development.	
Interview 6	31:25:00	And, and sometimes, well, that is a constant require for new research grants to do that. So you, you need to continue thinking about that even if we have very strong support for for a lot of the core development right.	
Interview 6	32:30:00	So that's	
Interview 6		that that's always one part of that the dependence on on some of these research grants for some new features. But um, I would say that it's, the thing is that now it's in a very steady growing place. So there are a lot fewer challenges.	
Interview 6		The challenges are more external that there are for example, we are We're working working on the net, one of the next things we want to support very well is sensitive data, very, very large data sets and have remote storage.	
Interview 6	32:33:00	So there you have the metadata and the repository, but the data set is, is in a, another storage and you can provide access to the data. So we're already working on on those	
Interview 6	33:21:00	new capabilities. And, and that's,	
Interview 6	33:26:00	that's a very important area that how then	
Interview 6		and researchers use the the data there, the sensitive data sets, right. And we are working on other projects that are not (platform), but complement other regional projects that could help to do analysis on the data without accessing the data inside.	
Interview 6		So it's, that's, I mean, the this science of the data and the sanctity of Data is one of the things that are more challenging, and we're working on it. But we have several solutions already in place. The other thing is that integrating with entire research data management within an institution so that to be able to integrate with other tools, and with cloud computing so that you go from the data that is active that are you still doing research to, to publishing it for others to use, but in a way that is more seamless and easier to easier to do that you don't need to go from one platform to another, you're in the same platform.	
Interview 6	33:32:00	And that's what we're proposing this data comments that would integrate (platform) with other pieces of the research, workflow lifecycle, and other tools and cloud computing.	
Interview 6	34:58:00		I'm just having an Eye on the clock. I know I know.
Interview 6	35:05:00	A lot of information so you have enough information.	

Interview 7	Time	Interviewee 7	Interviewer
Interview 7	02:23		Can I get to know something about you? What you have done.
Interview 7		(personalised)	
Interview 7			Coming to the platform or the (platform). What is the history behind it?
Interview 7	04:20	right. Well. The (-) I cannot give you dates of hands but you can look them up ont he web. It was created by (person). Do you have that name?	
Interview 7			Jap
Interview 7		(person) set this up because he felt that the current access of cristallographic data was not suffeciently open - and I agree with him. He startet to collect open Data from the literature and make it available as this database. After some years - and I am not exactly sure when - he transfered this willingly to a group in (country) in (town).	
Interview 7		So that is (-) the group there - sometimes I forget names actually. (name) has run that for many years. That is a management group, advisory board, which I am on. which includes (name) and (name) and various people that I have not met. That is active and deals with a number of recieves that come up.	
Interview 7	05:49	Should I go on? Or do you want me to ask more questions?	
Interview 7			What is the core offering of the platform?
Interview 7	05:55	The core offering is to extract public knowledge from cristallograpic peer reviewed papers - there are maybe one or two which are not peer reviewed. To extract at the moment the cristallograpic coordinates and make them avail/ to validate them as much as possible and to make them available freely to the whole world.	
Interview 7	06:26		My work now is about success and success factors. How do you define success or a successful platform?
Interview 7	06:38	Right. Well. This is rather an unusual platform. Because it has both a technical goal and a political goal, right. The technical goal is to collect all this data in semantic form and they/ we have collected half a million data sets. So O think this is a good technical success. Politicly the goal is to make this openly available. And the problem is, although there are other data base who are do the same, they are not open. They are partially open. Do you what a walled garden is?	
Interview 7	07:21		No, can you explain it to?
Interview 7	07:25	W-A-L-L-E-D walled Garden. You can find this - let me see if I can find it (searching on the computer)	
Interview 7	07:40	I look everything up on wikipedia. So.	
Interview 7	08:00	Basecly it is something that people have access to but they do not have controll over and where the conditions of use may vary at any time. Typically walled gardens are facebook and (platform) and things of this sort. They offer this service but often it is said that the user is the product.	
Interview 7	08:30	You heard this phrase?	
Interview 7			Yes, I have heard this phrase.

Interview 7	08:31	That is true of these. I have got actually - this is wrong walled garden here (reffering to the link he send via skype chat)	
Interview 7	08:48	It is also called here closed platform in wikipedia.	
Interview 7	09:00	So. A walled garden is in a useful horticultural. Why cannot I find (talking to himself)	
Interview 7	09:17	Yes, so that is really what it is. So. The databases that - if you like - our competitors are the (platform). I have nothing to with that altough I am in (town). And the (platform). And they maintain that there are free in the term, you can get a small amount of content for free but there are basically subscription services	
Interview 7	09:55		What is the vision behind the (platform)?
Interview 7	10:00	Right. Well. I will give you what I think the founders would say and the current. It is to create a database of all the published cristollagrophy which has been validated and enhanced and is freely available to everybody under an open licence, such as CC0 or CCBi. And which hopefully would aggreagate a community of use.	
Interview 7	10:35		What is the organization structure behind the platform?
Interview 7		Good question. When (name) set it up it was really very much a personal effort. Now th´hat (name) is taken it over it run from the University of (town) but it is very much his activity. But the (-) governance as I sad this advisory board. There is an occusaniol funding.	
Interview 7	11:15		How are the responsibiliteis for the platform distributed?
Interview 7	11:22	The main responsibility is (name) and his group. He runs by technology cristollography as a research scientist but he has marginal resources and he usually has two or three phd and post-docs who spend a proportion of that time working on that database.	
Interview 7	11:53		So how many people are working for the data base in general?
Interview 7		Nobody is working full time I would say that (name) and two (-) volunt/ well phds are working part time within their academica responsibilites. That is something that fits into gaps in programs. I am not sure what (name)s position is. I think he is propably retired but there are two or three others who take you know they mail, they find resources and they will add data in as well.	
Interview 7	12:35		And when it comes to desicions regarding the platform. Who takes the decisions and how are the decisionspaths.
Interview 7	12:40	As far as I know there arent any votes. It is done on a mailing by general consenses.	
Interview 7			OK.
Interview 7	13:00		You already mentioned the funding. How is the current funding of the platform?
Interview 7	13:05	I believe that (-) I am not absolutely sure and I think you should check with (name). I would sayy that university provides some of the infrastructural resources and it is done on marginal resources that (name) has as part of his research.	

Interview 7	13:30		You mentioned already that (platform) and (platform) are competitors from your side. When you are set up and manage the data base now. Do you think about a strategic position of the (platform)
Interview 7		Well. I would say / OK (-) What I would say is that the value of the database is that because it is open. It can be used in ways that you cannot do it with the other platforms. So. Being completly open allows you to do what you like with it, so long as you actual tribute it. So in other word you can put it in a variety of machines, you can tranform it into other products, you can take parts of it, you can combine it with other information and you dont have to get persmission for it.	
Interview 7	13:50	If you take the other platforms. You cannot do anything with the data without their explicit permission. And very often they will put sever restriction on redistribition of many of these unless they are actively involved. They might for example forbid you to do it unless you have a research collaboration with them where they would gain academic papers and so on.	
Interview 7	15:03		What are specific goals are now for this platform?
Interview 7		The goals - I would say - (unv.) they are a to get as much data in as possible, b to develope software which will be valuable in maintaining and enhancing the data base. And we are looking all the time for new applications and new data that we might put into it.	
Interview 7	15:05	One of the things we are looking it is incomplete in the literature which wouldnt get into the other data bases and can we find a way of putting them in and making them valuable even if they are incomplete. The fact that there is some information very valuable for people. That is typpicaly of that sort of thing that people want to do.	
Interview 7			And how do you control the goals you set?
Interview 7	16:05	I would say most of it is controlled trough the actual implemention in (town). In other words: (town) is a sort of mastersite for this. And (-) there are / there are not people who clone it and do it and things in general that we know of.	
Interview 7	16:20		What are difficulties and challenging / challenges in manging the platform?
Interview 7	16:25	Ok, well. Obviously there are resources because of (-) it is very difficult to get grands for these sorts things. Although we are always looking for things like (region) funding because it (-) you know there is an increasing interest in open science, in (region) funding.	
Interview 7	17:30		Now we have finished the first whole blog about the organisation behind (platform). I want to continue now with the data governance and functionalities of the platform. My first questions to you: what functionalities and possibilites does (platform) offer?

Interview 7	17:53	Well. The main functions are to injest new data sets. I would say to cruel new literature for new data sets, to take those data sets and finally date them against a large set of criteria and either to say that they are compliant or to (-) come up with enhanced (unv) to that data to make them compliant. That is the primary activity.	
Interview 7	18:25		And what are further activities?
Interview 7		Further activites are to offer the world search facilities on the (town) site. And of cource anybody who (crunsh) it but primarly on the (town) site so they / you can search it for cristallographic information.	
Interview 7	18:40		How do you describes the participating user group the user group and participating institutions or organisations.
Interview 7	18:54	Right. I dont have a complete overview of this. There are a number of people and institutions who work quite closely with (town). So I think the(society) Society has an (-) agreement where they make data immediatly available to the data base. I think one or two other examples like that.	
Interview 7	19:22	Other than that there is an open community but they often dont tell us - this is true of many open data resources that people will take it and use it but you dont know who they are. And this is accepted as part of the philosophy but it is al ittle bit frustrating occasionaly.	
Interview 7	19:50		What difficulties arise when exchanging the data?
Interview 7	19:05	Exchanging it where?	
Interview 7	19:57		Via the platform. For example when someone uploads something or download somethings and has to or wants to reuse the data.
Interview 7	20:05	Well, there should not be any proc / any legal (-difficulties. And if people upload data which are not in the correct format, it will fail to upload and they will be given a (-) email note message. As it and why it doesnt upload and if they have got seriuos problems they will contact (name) and he will help them sort it out.	
Interview 7	20:35		What requirements do you have for submitting the data?
Interview 7		Well. I am not sure that this is an official answer but it is: the data has been published publicly that people can read them somewhere. And we transfind the diffinity dataset which represents that publication. I think there are pwople who are also submitt their own personal data set as well and that will be on personal agreement with (town).	
Interview 7	21:10		How do you ensure that people upload an efficient number of data sets?
Interview 7	21:27	Well. The minimum is one. People have uploaded one data set. And people have uploaded collection of data files and so on. And sometimes it can be in thousands.	
Interview 7	21:49		And how do you ensure the quality?
Interview 7	21:51	Maily algorithmicly. So. There are many many checks. The Internatonal Union of cristallography has a batterie of 500 checks which can be applied. And (platform) applies all these checks. Is there something internally inconsitence with the data it will be picked up.	

Interview 7	22:17		Related to data quality is also trust. How do you ensure trust into the data?
Interview 7		Well. (-) The primary trust problem is small. It would be that somebody creates an artificall data set and uploads it. I think this is very unlikely because almost all these data sets are associated with a publication. And it would be for the journal or other publications take on the responsibility of vetting it.	
Interview 7	22:25	I am not aware that this is a significant number of papers which would be uploaded by individuals which might not be trust worthy. It is actually difiicitult to see the motivation for this. Because You dont get much in the world public credit for it. There had been some cristallografic studies in the literature which have been fraudulent. but these might have got uploaded into (platform) but there are / if anybody detects the fraud than they would be removed from (platform).	
Interview 7	23:42		How do you encourage researchers to actively use (platform)?
Interview 7	23:50	Well. Right. Mainly by email lists, tweeting, social media in general.	
Interview 7			And what other possibilities do you see?
Interview 7	24:10:00	For advertising it?	
Interview 7			Yes, I have heard this phrase.
Interview 7		Well, if you public your work - openly. Which I hope you will do. Then anybody reading this work will see - that is another advertisment for (platform).	
Interview 7	24:24:00		How do you ensure that users are using the data scientificly correct?
Interview 7	24:30:00	This is, this is difficult for us to do, formally. Because the CCB oder CC0 licence says you can do whatever you like with this data. And it is absolutly not mechanism of contraining how people use the data. If people want to take the data and say: I have created another data set where I shifted all the atoms around because I wanted to see what happened with rendom structures - they are welcome to do it.	
Interview 7	25:09:00	It is concivebale you might get some confusion here but we can always determ that it was not the data set in (platform). So. if people want to use it for (-) for purposes which will be against the general value of the human race, if they want to search for compounds which can be used to make illegal substances or to make nuclear products or something like that, we can stop them.	
Interview 7	25:59:00		How do you estimate the reusability of the data?
Interview 7	26:00:00	The reusability is high because it uses a standard for cristallography called CIF - C I F. Which have been worked out for 40 year. So it is probably the best scientific data interchange format in the world. Certainly for individual data sets. And by publishing with CIF you garantee that it is reusable and that there will be software which allows you to be transformed and used in a variety of ways.	
Interview 7	26:40:00		One special asect about (platform) is that it is within the scientific community. How do you think does the scientific culture unfluence the use of the plattform?

Interview 7	26:50:00	Well. I would say the cristallography has a very long tradition of open knowledge - even though it is not always been descriped with the term "open". So cristollographies had many studies where people collect data in different labs an compare it ot see wether the methods are consistence - much more so than many many other subjects. It is a tradition of validation what you do. There is also a tradition in open publicatiosn. It it always been ahead of all other subjects in depositing data in public view when they are created. And that is a continue process.	
Interview 7	27:50:00		What development do you see for the plattform in the next ten years.
Interview 7	28:00:00	That is a good question. It depends on how many people we can convince that it is truely valuable in terms of ist comparison with the other data bases. I would say that the things that we got - first of all - that it maybe that there are projects which require the data use to be open and that is not true for the other platforms. It maybe that we could create downstream products by aggregation or by (-) compartment lisiation which are more valuable. My particular ingest it linking it to the open literature that we include text of the data as well. (-) but wether it takes off or not will depend on the world in general.	
Interview 7	29:00:00		This is exactly my next question: What does the success of the platform depend on then in the future?
Interview 7		It depends on the world appreciating the value of opennes. Now - I have been in open for 25 years. It has been very difficult in chemistry to create open chemistry. I am probably you know the one or two leading people in open chemistry. But still most chemists will not publish their data openly, many will hide it and so on.	
Interview 7	29:04:00	In bioscience it depends on the actual bioscience as to how open it is. So. In fiels like (-) new genome research there are a lot of facilities for open genome research and so on. But there are other fields where everything is very closed. So it depends very much on the field.	
Interview 7		I had hopes 15 years ago that the world would rapidly become open but this is not happend. The major problem with scientific publishers. The major publishers are generally aggressivly anti-open despite what they say: Springer Nature, Wiley, Tele in Frances (unv.) The (region)n chemical society are all determenly that their primarly products are not open and that they keep their information that is given to them and that they control it to use it in their own way.	
Interview 7	30:00:00	While that happens I think it is going to be difficult to get an open e-source. The open-access movement has stalled (still stehen) and it is, it is hang up on (-) it is hang up delocracy about how much you pay for open rather than acuatally declaring it: all papers should be put into open repositories and similiars created. This will come but it will take 15 years. By that time (journal) will probably run the whole of science.	
Interview 7	31:20:00		You just mentioned that it is hard to establish open chemistry. Why is it the case?

Interview 7		Well. I think it, has for several reasons. First of all, Chemistry is a subject where you can do a lot of chemistry in your own laboraty with a group of twenty people and you dont have to collaborate with other people. In Bioscience it is increasingly impossible to deal bioscience without interacting between genes, organims, enviroment, methodology etc.	
Interview 7		You find many bioscientists have to work in large collaborative groups. This is not generally true of chemistry. Secondly, there is a history of chemistry creating information products which are sold. So typical examples are chemical abstracts which generats many millions a year - we dont know how many, but it is probably in the hundreds of millions. With that e-scource there is no incentive to make it open. So the (region)n chemical society runs chemistry essentially as a top-down empire.	
Interview 7	33:15:00		Do you have any thing else you want to tell me about platforms, about success factor of plattforms?
Interview 7		Well, there is a whole spectrum. The thing one has to be very careful of is platforms which start out looking open and then become closed. So typical examples of this are (platform) where people would collaborate in this and then (platform) decided to going, sorry (platform) sold to (journal) and it become closed.	
Interview 7	33:25:00	It was not a surprise to me but it was a surprise to various people. The same was true of (platform). Which were run by two people who run it - apparently for the validity of the community but now sold it to (journal) for a large amount of money - much larger than yoou might think. And the product has become a very clear walled garden.	
Interview 7	34:20:00	Same is true with (platform). Which is now beeing agressivle developed for floatation on the market and where the volunteers feels extremely ard done. So anywhere were you have volunteers you have to be very clear that these voluntees - unless they are protected - will end up in somebodies else walled garden.	
Interview 7	34:50:00	We need regulators. We dont have regulators in this space. I am (-) (unv) appoled by the lack of regulations of scientific publications and I continue to say so. he publishers can do whatever they like. And (journal) do. I believe that - what will happen - (journal) will take over research support frameworks, the infrastructure. So you end up not beeing able to do knowledge research except on (journal) platform. Because the university will sell to (journal). That is what I predict will happen.	
Interview 7	35:35:00		Anything else from your site?
Interview 7		Well. I would say it is important to keep the idea of open clear. So unfortunaltely "open" as a term is almost useless.-It is been what is called open wahsed. And therefore you have to talk about licences. And than I will come back to you. Do you plan to make your work open?	
Interview 7	36:00:00		Yes.
Interview 7	36:04:00	Tight. Well make sure that you have a fully open licence. Such as CC-B.	

Interview 7	38:50:00	What I would ask you to (...) - if you have time, it is a big job- is to analyse all of the platforms you have talked about and see if you can say wether there is a licence for upload of data that protects the uploader and wether there is I think on the platform wich prevents it becoming closed in the future.	
Interview 7			And I can find in the licence agreement.
Interview 7	39:24:00	If there is alicence agreement it will tell you about reuse of the data. But you should also try and find out wether the platform has any garanteee against becoming closed.	
Interview 7			And what else is important looking for?
Interview 7		Well, those are the most important things. Can anyone get the data back out of the platform and use it? Right. What I would say also - what data does the platform collect about users. So in other words is it GDPR compliant, are the platforms using user data for any purposes that the users may not be deliberatly aware of.	
Interview 7	40:00:00	Do they publish that? Is the (-) review body for each platform which looks at the ethics of the platform? And (-) are there any guarantee about the future. It is difficult to get garantues about the future but the best ones are if the platform is run by charityable foundation. So because charitys will not allow change of use in this way, generally. Are all if it is run by a trusted organisation such as a, you know, (region) organisation, a orga/ a learning society or whatever. The learnint can not always be trusted. Does it all makes sense?	
Interview 7			Yes, it does and I am very grateful that you tell me all those aspects
Interview 7	41:15:00	(someone interrupted in the room)	
Interview 7	41:45:00	Hello.	
Interview 7			Yes, I am here.
Interview 7			Do you have anything else what I sould consider?
Interview 7		I think those are the main things. What I told you just now will require a lot of (-) will require a lot of loking in detail at this. But it is important. Because otherwise people say they are open and you can find in a year there are by (journal).	
Interview 8	Time	Interviewee 8	Interviewer
Interview 8	01:16:00	Lets go ahead	
Interview 8			Now I have introduced myself. What about you. What is your background and what are you doing?
Interview 8	02:50:00	personalised	
Interview 8	03:45:00		
Interview 8	04:15:00		And you also gave me an insight into the history of the data base as well.
Interview 8	04:27:00	It is very important to mention people. I could not mention in the brief story all people who are involved, there are many more of them. You can read them / read them in publications and so on. Find the list of the advisory board or in the history of the (platform) but briefly it is like.	
Interview 8	04:50:00		And what is the core offering of (platform)?

Interview 8	04:54:00	So the core offering is that all published and known small molecules cristalls structures. With saying all this of course (-) it is to be qulitified. All that we manage to collect so far by various means. Either by scan literature, getting supplementary data or by direct depositions of cristallographer, chemists in our data base.	
Interview 8	05:10:00	And this is by no means at the moment, all published structures but we are getting to half a million records wich is already interesting I would say. For example for machine learning if you want a training set you can grab it from there. If you want to make statistics to see what molecules are out there in the wild you can look at them there. And our main goal so to say, we should say mission and vision that is fashionable -our goal while we are doing this, we want to make all published data openly avaible on the web.	
Interview 8	06:17:00	And that is the direction which we are going htis and of yourse we want / we dont do just for the sake of having it, well, on the hard disc which is of course fun but is not so useful. The useful thing that I am sure we, you, all scientist will be able to make useful inferences, scientific inferences from that data set. And also from other data that are related to it which we then having an open data set we can interconnect with chemical data, with pharmacological data, with the rpb and (-) biologoceal cristallographic data.	
Interview 8	07:04:00		So my work now is about success and also success factors. How do you define success or a successful platform?
Interview 8	07:17:00	Well. Of course I will be biased. First of all, the platform needs to have data of reasanable qualitiy. It is not in a just dump all file into one place. We need to clean this up. You need to make sure that is uniform, you need to index it, you make it findable. And there is now a lot of movement to make data findable, accessible, interopareble, reusable - FAIR, you know - buzzwords out there. But they have a real background because scientist need that, industrie needs that. You need high quality data set.	
Interview 8	08:07:00	It needs to be reasonable comprehensive. Of course when the data base starts, we started wih 200 records or (year/number). Well, that is very little. But now we have a substanial sample of what have been determenand by means of cristallography. I think you can already find a lot of representatives with us.	
Interview 8		And then of course a resource needs to have policies what you put into there, what are the criteria accept or reject strucutres, it needs to be curated. For example there were known (-) cases of scientific fraud in published cristallographic structures. So, you cannot leave fraudulance in your data base, you need to handle this on the policy level on the technical level.	
Interview 8	08:30:00	So that is what the (platform) does for the data we have. And then, I said I will be biased, you need to make sure that your resource is available. Because, it is not my metaphor, I think it comes from Blake Astin, Search data base maybe from some other. The mad is full of data doompstens, data graves. What does it mean?	

Interview 8	09:27:00	Somebody gets a projects, gets financed saying set up a data base. Transfor three years, four years, the projects ends, the money is out, the postdoc leaves, the students go away, nobody knows how maintain the (platform)e, how do grade it and eventually the server vanishes, nobody updates that database. That is definetly not a success, ok. And the policiy explicitly want to publish about a data base in good journals, I assure it will be sustainable.	
Interview 8	10:00:00	So the (platform) is now here for (number) years. So, I think we have proven in / with all our actions we can sustain for quite a while. It survived several institutional, merges of university, transfer from (country) to (country). But across all this already several generation of computer software and hardware, ok. So this longlevilety means you can rely on it. Apart from that (-) what else is a success or not.	
Interview 8		It needs to be used. It needs to be useful. You can have a long lived resource of your cats photographs, it is nice but it is not - maybe it is also a success - but the (platform) is used as I can judge citations and scientific literature. It is from the team which we are working, for my western colleaques it is one of the most cited papers which have written several papers.	
Interview 8	10:50:00	And it is for me a demosite paper. People are using it for different research purposes. Companies are using it to supplement their equipment so that they can search match cristallographic sample. This means that it is useful. And I think this is a criterion of usccess. Well.	
Interview 8	11:55:00	That is basically it.	
Interview 8	12:00:00		Thank you to the insides. Coming to a different topic: what is the organisation structure behind (platform)?
Interview 8		okay? That's probably not very usual. So the (platform) is governed by the (platform) advisory board. That advisor what's not institutionalized in any way. It is a bunch of enthusiast of scientists. Some of them founded (platform). Some of them like me are like, my students now like beta mirus, dry and later as experts or as you know, supporters, and it functions on a mailing list.	
Interview 8		It functions quite efficiently, I would say. So we have informal voting procedures. So if we need to make the decision, how we store data, what will be our next policy, what we will write in our next paper wall that we just declare Change emails discuss. Usually all technical organizational matters come to a unanimous or near unanimous agreement, which makes it somewhat easier to organize and run.	
Interview 8		Of course, I must admit since we don't have institutionalized it. So there are no big legal issues with that. We don't need, you know, coming to understand some contracts or some things. And there is no much funding involved. Each group seeks their own funding. We seek funding indigenous people in (country) on in (country) on (country) they have their own scientific funding.	

Interview 8	12:04	This means that we don't have much money to share or to this and maybe if this (platform)becomes say project that has its Dedicated finances when the government's will have to be upgraded. But so far it's been broke.	
Interview 8	14:14		So just for an understanding, so when you make decisions, you send emails, and then everyone can vote it.
Interview 8	14:25	Okay? But there's no formal, you know, procedure that well, you need to answer it within one week and then your vote is counted at that and you need to, you know, wait for three months before you know that as the drastic change mails, those people who think it's relevant for them, they do answer those who don't they don't answer. And those who answer basically take a decision and those who don't, you know, so it's very unfortunate.	
Interview 8	14:55		And how are the responsibilities distributed?
Interview 8		So, the (country) team, we now run the servers. And we run, we construct software and we run the web crawlers that collect published structures and deposit them to the car. There are three groups four groups so Granada university where the maguet of Eros represents the court Danielle certain year and Ramallah by in France in different universities but shut in yet in MC con French name a French city egg problems pronouncing it properly with it and then there is bit MC and be made states they all are on mirrors.	
Interview 8		So they have preserved copies. If something happens bad for the lunar server, the data is not lost. That's what and otherwise responsibilities and armel he organizes volunteers who deposit structures he himself deposit structures does data curation, and so does Miguel Miguel managers derived chemical data.	
Interview 8	15:00	He has also publication on that. So that you can interrogate chemical properties of the crystals description we have. So basically, the responsibilities are accepted by a voluntary basis on based on what research interests of every member are.	
Interview 8	16:52	For example, (name) and some member of the advisory board, they made a resource that right from (platform) so that you can grab 3d printable molecule models. So if you have a 3d printer and need to use the model for your class, you can just grab any molecule you want, print it out. And so let's say they do that. And then taking decisions is if you want to respond, a self accepted responsibility, to participate in the mail, endless discussions, it's not a very busy mailing list.	
Interview 8	17:38		So you mentioned there are many different peoples and many different institutions involved. How many employees with what competences work for (platform)?
Interview 8	17:51	Well, I wouldn't call employees we don't have employees because a we don't have formal legal structure. So we come to play employed by the university not and second. The we don't have funds and and flow of cash to imply somebody. So we don't imply people's not institutionalized. Maybe we should change this we are thinking with this change. But okay, that's brainstorming that goes on the background.	

Interview 8	18:25		Now the people who are involved there are, how many?
Interview 8		Well, in the (platform) advisory board, you can actually grab the list of names and count them. Basically some I'm not very active, some are more active. So it's it's about 12, 15 people not counting their students and collaborators and (name), we have three people who want more, more or less permanently on the (platform) of course, as a as a part of their research, so we use the kafala research for which we get paid.	
Interview 8		And since we need it we for research, we keep maintaining and updating and supporting it. This this is part of all working. And then of course, we're happy to share this result with all about us that that's the best. So and then you see since it's an open database, so anybody could can grab the data and do what are the What was that?	
Interview 8	18:29	So this means that companies are recently political update of their different topics. So they have some people working on there. I know there are people, students, software designers who use the (platform)for to display molecules and their soft I don't Know how many people work there? Maybe, again, a couple of dozen times times 10. Maybe it's but this this spread out. The answer your question?	
Interview 8	20:12		Yes. Coming to the funding part, you mentioned that every participants are involved, people are looking for their own funding. That's right. So how do you get the funding in general?
Interview 8		Well, actually, we, I recently listed something like I think six possible models to fund the (platform). And, and the rejected the five of them. And the last is, but the current models like this, and scientists and research so apply for a grant to the research, and my research is connected with data and the cloud.	
Interview 8		So say look for this interview. allegation which I will do, I will need the (platform) database. So we put some of the resources into it. And then this way, we had two grants of (insitutes) in (year), I think and I can look up the years, quite a while ago. And currently we have a (institute) grant, where we use the (platform) for mining industry to identify minerals there, and we have a tiny part from the whole project is open 5%, but it's a substantial part, for for for our group and our team.	
Interview 8		And in this way, caught keeps going. And the same is for other research groups that are involved and this (region) project be participating But several colleagues from the crowd advisor reports from not just one group that participates in this project, but now this funding model.	
Interview 8		If I'm chatting too long, please feel free to interrupt. Because so everything is fine. I think this funding model is fair because you compete, unlike other databases that and I'll say, if you want to have access to data, they asked us some period. Okay, you either pay or you pay.	

Interview 8		Now we compete, honestly, for funding. And I say that the database needs to be funded by public money, only if it is useful for public. So if I can prove that it's useful, then we keep on doing that. If I can't, well, we do something else. But then of course, we need to take into account that the resource may vanish.	
Interview 8		But the problem is of course that it's patch. Okay, there's a huge petition there. So now you have the funding then you don't have then you have it again then don't have again. Now what keeps the (platform)going online? And is that we use very so it's very it's not resource intensive. Let's put it this way. So basically the web server University pays the currently for our electricity internet connection, because of the bringing projects for that this arrangement.	
Interview 8		But in principle, if they don't, I can put it in my you know, garage, drums from there. That's not a joke. The first server that was put online was bought by (name) by from his private Me and put under his desk in his universe that this first server was bought by my private money, we just went to shop bought a cheap PC.	
Interview 8	20:29	And it's good enough for serving open date. And then of course, it's open source software that also allows us to work on very low costs. We don't have any license costs. That's, of course, we need to have qualified people to do that. But since we are scientists, and we're using that software anyway, this is not an issue. So I would say even if there are interruptions in the (platform) financing, we have a fair chance that the cloud will survive.	
Interview 8	24:50	But of course, if we have, if we had more constant financing, we could do more. We would have not maybe half a million structure. So we would have linear structures, which would be nearly everything that was published in the scientific literature. So you see, it's it's a trade off.	
Interview 8	25:12		Do you have like long lasting financing from the Institute to run the server is everything via project money?
Interview 8		So currently, we have agreement with the university that they host our servers. And that's it. So this is of course, a contribution and kind from from us and the same agreement was from the Institute with the Institute of biotechnology, that hospitals before before emotional theaters.	
Interview 8		Now of course, this is this conditional on as getting projects stopped getting project for some time, maybe some different arrangement will have to be done. But as I said, I'm not very much afraid that you know, Okay, now you get out your service, we always find the place where to put this into some data.	
Interview 8	25:22	Keep on going, please. Yeah, since since the data is open and licenses public domain, so nobody can say we own the data. Okay, the University can't say they own the data. I hope in any case we receive from our mail the original data set on the understanding that it's open database and it will be developed as such. So this means that we can put it anywhere on the web and people can	

Interview 8	26:40		you just mentioned that you want to prove that (platform) is always useful and so on. Do you have in mind where or how do you want to strategically position caught?
Interview 8	26:57	What do you mean strategically position,I can conjecture my own interpretation.	
Interview 8	27:04		So I mean, when I speak from an economic perspective, there is like a whole data market on the web. And there The question is, where do you want to see cut in the future? Or where do you want to position it for users or institutes?
Interview 8		Well, I must admit, I'm not very much aware about the data markets. Maybe that's our weakness. Maybe we should have participated in several initiatives where the web talks about the marketplace digital marketplace, but I didn't see how this would could take off and work reasonably now, as I said, discussed and dismissed funding models for (platform). Father (platform) One is licensing data access, that's definitely a no go that the reason why the (platform) was created is to make no licensing restrictions on access to date.	
Interview 8		And this is very fundamental this has to it. So either this goes like this or there is no sense of because you have commercial database where you can buy it, everything is in the structural database, you pay the money, you agree with their license, you don't publish your results, and then you have that data. So if that's okay for you, you are you are fine. But for many purposes, this is not okay. There are publications that you know, explicitly statement so that's not okay.	
Interview 8	27:27	So, what we definitely don't want, we don't want to lock up the data and say now for access to that a two players, that would be nice away. But of course the competition is there. Everything already is already partitioned in these commercial databases. They are available. But this is a subset classical marketplace model right. Now, this is not good for many reasons, which I can elaborate, if you want.	
Interview 8	29:18		So, yeah, if you want, please.
Interview 8		Well, there are two issues with this. One is, I would say the rights issue. I think that if research has been done for public money, every citizen has a right to access data and has a right to use it and to republish their results and to discuss them. So these results must be online openly without licenses and district restrictions. Nobody should have a right to say, Look, I know grab the publicly funded research data, not lock it up.	
Interview 8		And then now I forbid you to republish rate your results. That's essentially what happens with commercial. So I think I have a right to access this data, you have right experts in the system. This is fundamental human right. And it's increasingly important because you know, if you studied machine learning you know, training data sets is all what you need to put them it's it's fundamental for don't have good training data says can you do machine learning, you probably know better than me.	

Interview 8		So, so it's very important you you have to track to access this data somehow. And if you are cut off from data source, then you are disadvantage. And the second this technical and and efficiency reason, we live in a connected world 21st century now all data flows freely. interactive see, we are talking your Germany and the Sienna, no problem, data flowing freely, and social scientific data, social crystal structures do if you want to know how, for example, Sakura structure looks, you should not go to somewhere and understand licenses, you know, get your university manager to buy a database, maybe next month now you just click and you get into one second.	
Interview 8	29:22	And if you can do this and then you can immediately do computations, scientific inferences, planning anything instantly. It reduces time span. And I think this is a huge advantage for now and beta. Okay, how you get it financed and sustainable and who pays for the additive it's not free like and free beer. It's pretty like in free speech. You know, this metaphor from free software movement. That's another question. But I think so far we are up and running and going Live and kicking (lachen).	
Interview 8	32:04	Okay, so so that back to your question on how position the (platform): as an open resource, it should eventually end up with all or nearly all crystal structures that were published, available public. And it should maintain accepted scientific quality criteria set by the International Union of the crystallography. So if you draw your own molecule and say, Oh, that's very nice. I want to deposit now you have to make an experiment to have solid data to support your model, then it will go into the cloud. This means that you can the users can rely on the scientific standards.	
Interview 8	32:50		In the beginning, you explain me your vision of (platform), do you have any specific goals you want to achieve?
Interview 8	33:03	Well, we are currently working (promised not to publish)	
Interview 8	38:24		So, how do you control the various goals you set?
Interview 8	38:34	What do I mean by control?	
Interview 8	38:37		Well, I mean, people sometimes use KPIs or have a review process or I don't know. So how do you control the goals?
Interview 8	38:49	It's very informal. We don't what is KPI? what's what's the KPI?	
Interview 8	38:53		KPI is a key performance indicator.
Interview 8		Oh well, I see. we are not into that. Again. We are much less formal. We are we are much more grassroot. And basically you have an idea and you try it sit at the computer (platforme is it works then you get a student who does it. And the key performance indicator for us is if we can publish a paper on that.	

Interview 8	38:59	Okay, if we saw that the ultimate goal is to have our findings published in a period of scientific press, because we academic workers, and if we can do this, we are happy and then we said we have this agreed quality indicators. So we look through our data and say, okay, is it a good trade? Can we draw inferences on it can use us access it and if there are some flaws and that for example, some data records, we know that our flaws, so we say, Okay, this is an issue for data curation. And we file an issue and project management system and then eventually, it's shouldn't be resolved when the resources are there	
Interview 8	40:25		digging a little bit deeper into the quality indicators. So how do you ensure the quality of the data sets?
Interview 8		That's, that's, that's tough question. So first of all, so first of all, the ground level thing is syntactic correctness of the records that come into the (platform), the crystal structures, as you probably know, distributors, so files and serve has a formula specified computer readable syntax. So it's not that you can just write anything you want. must conform to rules. And these are mathematically defined and formula checked. And if they don't, just don't talk.	
Interview 8		Now, you may think it's what's the issue here is the action, but the truth is that even some published structures you know, the older ones do not conform to these rules. So, we have all of the even specific software that tries different heuristics and tries to to restore the centers. Then there is irrational you know, crystallography sets certain quality indicators. For example, what should be the agreement between your modal and observed the so called are factors goodness of it's difficult to gauge how much data has to be measured before you can claim the structures and all these are checked if you would pose it in Your structures of personal if the structure has been published, then it's not much we can do that, because it went through peer review process of the journal.	
Interview 8		And it's published and we can only register that fact. But the structure was it was published. So sometimes, but we can then flag them as having, you know, high discrepancies in again, you need to think how to do this. You know, you can't do we don't want to put blame on anybody. That's not constructive. But we can say you can say, we can say as a matter of fact that look, you're the structure has an R factor of, you know, 15%, which is three times larger than the Euro structure of this symbol that compounds in our database.	
Interview 8		So you think as a user, why is it so and what can you draw your conclusions from that? Not this weekend? Then there are formal validations using dictionaries or ontologies maybe now, so, we can check if all required date items out there if the connections between the data items are right and so on so forth.	

Interview 8		And and then comes the matter of the physical and make sense right. So, the, this is again then probably we get into research area which was not yet finished, you know, how do you determine how to discover wrong data items, sometimes you see that the molecule is distorted in the model and you know from experience it cannot be like you know benzene ring must be flat.	
Interview 8		If it's not then something is wrong. Now, you can spot this immediately if you have one structure, you can Go through Well, in the day I can go probably through a couple of hundred structures quickly, maybe 1000. But if you have hundred thousand no track, so right a software business automatically collect statistic and not on the pens and brings other parameters and directions and so forth. And then finally, you come to philosophical question.	
Interview 8	40:32	So what is the scientific truth? So, always provisional measurements are always approximate. So, Germans have the saying. How unsecure is secure enough. So, I can say how inaccurate is accurate enough room purposes it may be good and their fondest. It's not that different levels of accuracy. We need to find ways to record them to represent them faithfully. And this comes then into inferences which you do from the day	
Interview 8	45:03		related with quality is also trust. So how do you ensure trust in the data? Oh?
Interview 8	45:16	Okay, in which sense Do you do use the trust? Is it can computer term or is it a human? So social term? The social human term social health doesn't computers ensure trust by exchanging cryptographic certificates	
Interview 8		Yes, key key management problems. So we also have this we have certificates on now so you can, you can trust that your computer can trust that they connect us. So if you want so now human Well, I would say it's like, like general in science how how to trust what the scientists said.	
Interview 8		Well, usually our trade is based on trust, you know, you get funding, because you're a scientist, and it's a matter of honor, it's a code of conduct is that you report all the things which you find that things are true, and you check them accurately. So the same goes to database. I mean, we look through the new structures as much as we can.	
Interview 8		Well, not all structures, but pretty much daily, have a look at the database, check whether it's not has not been hacked, that the processes are running recently that new structures come in. Sometimes we need to notice that something was wrong. And we do that. And we promised that we write in our papers what we promised for other people.	
Interview 8	45:36	So if people believe without promises, but probably earned some trust, and again that we cited us, this probably is a proxy indicator to that trust. But it's like any scientist, right? Like why do you trust your doctor? Okay, Doctor said that you should take this pill and you'll get better. Okay. You know depending on the circumstances you may or may not do that but but usually you know,	

Interview 8	47:22		coming back to the management of (platform), what challenges and difficulties do you have in managing the platform?
Interview 8		Oh probably the challenge the biggest challenge is as you can guess this ensuring the stable, the uninterrupted finance now is boosting that we are not afraid of. Of course we want to do the best in the station we are in. But I admit that for me the getting caught Finance this one of the headaches that I don't have 100% solution for now, nobody does, actually, even the commercial database, you know, they don't have 100% guarantee that things are there.	
Interview 8		But so we'll have to set that there are several, several set, we don't want a closed access funding model. We are working with ground based funding model, which there's a sort of okay for now. But it's so far, it's the probably the only funding model we have. So if something goes wrong there, then we might be in trouble. Then you might think that you have some services offered on top of the cloud.	
Interview 8		We tried it but since we're not Very much commercial people we are scientists within proceeded foreign nothing, no companies don't want to depart with their money there is soon. So maybe you need a market more marketing for this and also there is a danger that if you do some accelerator service that brings in money, then this is becomes your main object. And free open database is something that you do as a hobby.	
Interview 8		Even if that there's an media which allows you to do business you need to be really very, you know, enlightened to say okay, we need to put this much funds from our profits to maintain the open resource. So this This is dangerous path and we agreed that we discussed this and advisory board and also stated or simply agreed that this is not fast rate for government funding like (platform) I think we need to get larger and more used to approach governments.	
Interview 8	47:35	And and again, do you need to do some lobbying policy This is this takes time. And again, scientists are not very good at lobbying business because no affair but good at doing business I would not be sitting here and the nurse	
Interview 8		is probably I don't know. Or so this is another, okay, challenges. Another big challenge is political one. Because many structures are not that were made for public money that were for which the papers were published. This means that the data should be public. And even sometimes papers open access, we can get the paper but the data not data ends up	
Interview 8		For example, in the (platform) that explicitly for better to take the data and put it into an open database, and we had serious ropes was that because, of course, let's say undermines their funding model, they, which I would say is morally wrong, because what they do they create artificial scarcity deprive people of access to data, and then they, you know, start extracting money from them.	

Interview 8		So that's, you know, understand that they need to finance themselves somehow, but I don't subscribe to such such funding well. Okay. But of course, there is a lot of policy going a lot of underwater currents, probably, many of which I don't know. But the result we have now is some publishers are not putting data on their websites.	
Interview 8	50:26	And they're saying, Oh, look, we can fetch it from Free from the (platform) and the centrally such data are locked away from the (platform) or from any other open database that might exist. This is a problem which will somehow need to approach. We do this by by raising the question scientific conferences in press and in our papers.	
Interview 8	52:28	You know, approaching scientists, this is an evolution that goes on. publishers. so probably these are the two most most problematic aspects of what we do.	
Interview 8	52:48		Do you have other challenges? Or otherwise I can continue with the next question.
Interview 8	52:55	I just go with the next question. I think these are all others are technical things, technical things. All of the computer breaks down to go buy another one or replace a desk. Go ahead.	
Interview 8	53:09		Coming also to technical things now, and what functionalities and possibilities does (platform) offer.
Interview 8		Okay, so it start with the least, user friendly probably. But the one that said is the best search method of (platform). So you can download the whole data set. And it's in the standard past computer readable form and you can do whatever computations you want to this and people do that. You can access using different protocols. So the ocean protocol, arcing protocol, FTP HTTP protocol, standard web protocol, you can download the archive.	
Interview 8		We also offer access to the whole creation and creation of history. Good, because I think we're pretty much one of the first scientific databases that introduced us throughout history in versioning. The method comes from software development. And basically, you know, what the software version control systems are. So we all our data is and the version control system and some version for the last since (year). Missing that need to look up.	
Interview 8		Yeah, I think (year) we receive data from our man, and we needed to put it under version control. Since that time, it's an interrupt history is correct. So we can look at all history. You can see when the (platform) was created, what was strange, you can estimate where the errors were corrected, or maybe unfortunately, you were introduced, then these need to be corrected. Everything is correct. And you just can check out your work and then you can update to the daily To get new structures that come, then we have very small simple website where you can search for the structures you are interested.	

Interview 8		Then you can also search by structural formula. We have done this recently. It's maybe not very flashy that search mechanism, but it works and it's supposed to give you a first croute selection, which you then can download and process on your site if you need a lot of data. And if you want just to look at one or two or five structures, then each structure has landing page where you can see in a dream a lap lead, how the molecule or the crystal looks like what are the main parameters there is a reference to the paper.	
Interview 8	53:18	So basically, you can use network navigation, investigating that and we also offer restful interface, of course. So can Clara same website. computerized means and we offer access, read only access to the SQL tables. So if you know SQL, you can do select directly from the database. And we try to keep the tables compatible and don't change them too quickly too often, too, drastically. So basically your search and the know people, some people do that, as well as, of course, immensely powerful comes to data. Data.	
Interview 8	56:37		You just said people use that. How do you describe the user group of the platform?
Interview 8		It's very diverse. Now we don't since you know, we don't use it for marketing purposes, these numbers so don't deliberately invest in you know, counting how many We have how many, you know, unique users, all that stuff. And that statistic would also be misleading because unlike many other resources that offer access only through the work, we say, and encourage people to download the whole (platform), and then we don't know maybe the only university in somewhere in (country) downloaded it.	
Interview 8		And, you know, just one download gave us 10,000 users. Thanks, Caleb pretty quickly in (country), that yes, there are access from look at so relaxed, maybe we should have a NAPLAN actually, that we're dating with these pins from request, but very useful from trying other users from (country), some (country)ese corporation that dresses you know, make connections, United States, Germany and so basically the whole world access to (country)	
Interview 8		So, and then you talk in conferences, and for me the most valuable feedback agitations of scientific press, because that's what we do the cop, you know, we might have may have, you know, 10,000 unique clicks and maybe 5000 downloads and who cares if people just downloaded looked at this file?	
Interview 8	56:45	So I don't understand what throat way or just looked at the structure said, Oh, nice, okay, let's go drink coffee. That's not very high impact. But if they take data and say, Look, we can now see what is in there bring new knowledge. That I think is an impact. Even if it's not that larger numbers, think it's larger in significance,	
Interview 8	58:55		and what difficulties can arise when exchanging data via (platform)?
Interview 8	59:02	What you mean by exchanging via (platform)?	
Interview 8	59:06		I mean, it's the platform of the depositing data. And both sides.

Interview 8	Okay. So when you deposit data you need to. So for downloading data, you don't need to register we don't collect any data about you is basically free. Now, if you want to deposit data, then we asked you to create an account. We think we are GDPR compliant and must state this. So we really take care about personal data. We don't sell it without the password protected passwords are encrypted.	
Interview 8	So we have technical standards. And if something bad happens, then we have procedures that allow you to rescue a beta. And we again there was discussion advisory board. But if the person says, Well, I want to let my account forget me. There is a problem if you think of this in scientific publishing, because if you publish a paper, and you underwriters, then you can't come back and say, Oh, no, no, no, I know, you can retract your paper, but it will stay there for posterity.	
Interview 8	So there is a certain commitment from your side when you publish a scientific paper. And the same goes for the (platform). But still, we decided that we will honor the users requests to delay the data. Because basically our accounts up so, we don't practice it. We don't care who is the user who deposit the structure.	
Interview 8	We care that it's the same user, we can contact him or her, do some that occasionally and that the data they provide Legally clean and reasonable quality if that is specified. This means that we essentially, if there's us, please let my account it let that there will be anonymous user ID that will say the user number 25 two possibilities data and we will think about it So, but you need to make an account and then you need to upload the structure it needs to be in some format that's crystallographic Interchange for accepted its responsibility of the users to produce this.	
Interview 8	Mostly it's not a problem because all software right off the correct files nowadays, but still you need you need to supply metadata, who published the structure, when what are the quality indicators and so forth. Then read upload, go through these internet interactive web tracks and the brand you can if everything is okay, ultracapacitor can click on the possible this is sometimes a problem, you need to invest some energy to do some small barrier of entry for that now, but it thinks some part of this bar is fundamental because it's not just a wiki.	
Interview 8	Really, you can come in anybody can edit anything, and then maybe somebody will correct it. But it's a center of a database. So you need to make sure that you understand what you're doing. Then when the people get data, then you get to file it standard, we ensure that the syntax is correct. We try to ensure as much as we can that semantics, correct that is complete and so on, so forth.	
Interview 8	But how you professors, it's again on your site, you need to know how to extract data from. Now, for those who need some computer science background, I mean, it's not rocket science. But, you know, if you think that you can just, I don't know, copy paste things with a mouse, then you're probably not yet the right trip to use it.	

Interview 8		And I should emphasize that it's a responsibility for the end user to make a proper selection, proper filtering of the data that they use for their work and inferences. That would probably go without saying with any database, okay, any scientific resource with any scientific paper you read that was written and you check whether it's okay for you or not. But this is this, of course, assumes that the user put in some work filtering and processing for coffee.	
Interview 8		Why this is necessary because the applications are very different for data and each application has different sensitivity to quality issues. For one, application coordinate error is not so important for the it's more important for one application bibliographic data is relevant essentially for other application may be that thing you are looking at and so forth.	
Interview 8	59:13	So, we cannot filter or classify and put labels for every possible use our policies that instead we put numbers which we can compare, and then you can select by these numbers, what are what is okay for your application or what is	
Interview 8	1:04:54		coming back to the requirements for the submission. Do you have more other requirements? for submission? I just want to make sure that you told me everything.
Interview 8	1:05:10	Well, we do not require but we highly encourage that along with coordinate data and scattering factor data. So ops data, or powdered fracture increases supply. For protein Crystal macromolecule crystallographer. This is a strict requirement since probably (year/number) since that we can't insist yet that this is always the case. Because no journal, not all journals require that. But this is increasingly becoming a standard. And we highly encourage people to provide the state. Let's let's put it this way.	
Interview 8	1:05:53		And how do you estimate the reusability of the data?
Interview 8	1:06:06	What What do you mean by estimate? Do you want me to call a number?	
Interview 8	1:06:12		No, I'm, well, I don't want to answer this question in advance.
Interview 8	1:06:19		So because crystallography has its own specific ways of data reporting, it may differ from other scientific domains in regards of the reusability of the data.
Interview 8		Well, okay may boast a little bit I think crystallography is parodic example of how reusable data can be exposed and collected for access and crystallography, the International Union of crystallography has devised it was successful at that, but the venture this was a very successful attempt to set the framework for exchanging did they maintain and develop dictionaries for four decades 20 years now they're devising a second version of these that has think.	

Interview 8		So, thousands of parameters, these parameters very precisely describe what is the scientific meaning, what is physical meaning, what are the units, what are the measurement conditions, how that should be recorded, everything is specified there. And this means that if you get the file with several hundred of these parameters, you can precisely now if it says so mermen temperature let it needs to be this way, it must be in kelvins.	
Interview 8		If it's not, it's wrong, and so on, so forth, if it says, and it's quite funny thing. For example, for atomic coordinates, you can record them as a fraction of unit cell address or in a Cartesian frame and they're not canalization conventionally To be supplied in the special This is all described.	
Interview 8		Now, I take some old publication to put the data into the (platform)and the says that the the data are in the quarter sign next month. So I put in Cartesian frame x times x y Zed put in look at the molecule in the graphical display and no human eye human neural network is trained to recognize good things and bad things you need to say something is fishy, that it doesn't look like all the look at more carefully see that all bends and rings are compressed.	
Interview 8		I think what the hell is well the first year your first instinct is the unit cell is wrong or something like that. Or something is wrong and it go through all and then after half an hour of you know investigation and and data forensics, I realized that this was These were angstroms along unit cell address, so it was not an unprofessional frame and the angle was not nine degrees, it was slightly off. So there was a slide don't immediately see that it's wrong you need to meet to look at this graphical display the number of look.	
Interview 8		Okay, so Okay, we had to write a script to convert that proper frame and so forth. Now, now it's not thinking that I UCR standardizes two ways of conveying to the coroner's and human fantasies and limited This is a third way. What else can you mean you can use inches instead of angstroms or whatever. By the way, sometimes unit cells given a pic on meters, okay? Sometimes the old literature gives angles not an degrees and decimal part of this but they give degrees, minutes and seconds and need to convert please So in that sense, so you see, if you have these diverse sources and diverse conventions, how many problems we have?	
Interview 8		It's one half, half an hour per structure to make it sure everything is okay. That's not a computer speed. Now, in that sense that I UCR, and the (platform)makes two orders of magnitude faster. It's all uniform. It's all in the same reference frame. It's all in the same units. You just grab it and and process it, and we do it.	
Interview 8	1:06:39	My favorite example is I grab a record from the (platform). It has a symmetric unit in it. Then we apply submitter operators. We wrote a software for that called tools. It's open source software available on GitHub. So we reconstruct the symmetry then we find the connected networks of flatirons called molecules. Okay, bots. And then We, my PhD student who is now doing his doctoral thesis on this he wrote	

Interview 8		so say, improved existing libraries and wrote a tool to make chemical comprehension of this. Then from that you can say this molecule here we have an aromatic ring, here's a have a double bond here have a charge on nitrogen and so forth. And from this pipeline, there's just one pipeline and in the Union Unix in the hand, you have information that has chemical knowledge attached.	
Interview 8	1:11:08	Okay. So I think in this sense, it's immensely reusable and could even show how and then you have you see that in this crystal we have three different molecules and these molecules can now be looked at in the book him and and Felicia PK, you know, and there I don't know	
Interview 8	1:12:00	Heat, delta G water whatever you want. Of course, you need to have proper tools for that. Of course, in this pipeline, we convert from formats from surf, that descriptive of graphical lingua franca to let's say SDF or CML. Beta (name) that constructed the inventor CML, the Convert to this which contains chemical description of the mark. No longer crystal a breath. This neat this way needs to be gone. But the good thing I think is that can be done automatically.	
Interview 8	1:12:46		Well, I just processes during the same time you're talking and how do you encourage people to actively use (platform).
Interview 8		I think we just tried to make it good and open. And people use it. And we publish papers and people. You don't we don't go out to people and say, Hello, you, Scott, actually, we sometimes go out and say, Look, please deposit your data into the (platform). Because if it's where you will find it, they're always and everybody else will find it, and they will be able to use it. And you know, it will be in good shape. The the people need to be persuaded to invest some energy into building together this this open data resource.	
Interview 8		And for us as well, people need data, they come to us, they grab data and computers, we never try to persuade anybody to use it. And actually, again, I'll post a little bit when we neglect all sale search engine optimizations. We didn't do anything. Don't use Google and analytics or less. And at some stage we I think we are now if you enter (platform) beyond the first hit on Google and DuckDuckGo so we can say we have that crystal and and in if it's a useful resource people find it and use it.	
Interview 8	1:12:58	You could tweak it at some stage if you would enter open database will be on the third hit, now we have our other open resources become popular. So I think apart from publishing papers between my property should should publish a bit more on our recent things, we have recently made road to book chapters describing how we can use this material modeling handbook crystallographic modeling Books. So you just provides information of how the database can be people.	
Interview 8	1:15:11		One special aspect about (platform) is that it's within the scientific community. So how do you think that's the scientific culture influence the use of the platform?

Interview 8	1:15:27	(...) It's tough question. I think I said, well, scientists, as a rule can be trusted, which probably saves us in many respects that we know if you would want very much among us if you would want to subvert (platform), put on fraudulent data. There are ways to do that. But scientists as a rule, don't do Such things, because that's against their carrier goals against their worldview against anything that you're working on. So AdRoll can say, of course, we can hack Web CT website, but you need to note set for that. And the intersection between people who would like to hack it and most surface zero, let's say it was this was.	
Interview 8	1:16:24	So I think in this sense that scientific integrity culture test helps us being English. One one thing would be to have some trading platform that does things for money and when people want to share that money with you over them sadly you have to share it with itself. And another thing is to make scientific resource which is done for the sake of knowing things	
Interview 8	1:16:59		coming to the last questions, what development Do you see for the next 10 years of (platform)?
Interview 8	1:17:09	For the next I would like to see it becoming more comprehensive, closer to everything that is public. I want to see it integrated into the linked Open Data Cloud, maybe as a metaphor, not exactly as it and I want to see machines doing automatic inference of on the (platform) and using it for to answer useful questions for for anybody, for industry, for pharma for health scientists, for material scientists. If this happens, then I would say the ground goals of the (platform) would be fulfilled	
Interview 8	1:18:00		What does the success on the depend on then?
Interview 8	1:18:05	Sorry.	
Interview 8	1:18:10		And what does the success depend on then?
Interview 8	1:18:11	on dependent on?	
Interview 8	1:18:14		the success of (platform), what does it depend on in this scenario?
Interview 8		Well it depends on the (platform) being online. It depends on (platform) the growing reasonably and and collecting things. It depends on (platform)maintaining quality and structure as we talked it, and that depends on us doing interconnect interconnections useful data presentations, we need to make may be derived as a set across scientific disciplines. There are different languages let people talk so crystallographer describe structures in one way chemists.	
Interview 8	1:18:18	As I mentioned, we have tools to come from one direction to another. Now we want to publish tools and maybe to publish deprived databases that allow chemists to get this information immediately without getting these tools running them for their own redoing calculations. So this this, I think might contribute to the success of the (platform).	
Interview 8	1:19:25	What else well, doing more on the web tools might or might not help. I'm not sure up. You signal is getting weaker.	
Interview 9	Time	Interviewee 9	Interviewer

Interview 9	01:27		Before we start I would like to know about you and your background - what are you involved in right now?
Interview 9	01:43	(personal)	
Interview 9	02:25		And what is the history behind (platform)?
Interview 9	04:44		And what is the core offering of (platform)?
Interview 9		The core offering is (-) you can go there to (platform) and you can download, you can find where species have been observed for example you can go to the data base and lok up, search species (unv) and you can get points on the map of africa where (unv) have been observed.	
Interview 9	04:50	You can look up species of plants and you can see where it have been species of plant have been collected. You can get a time period where it was first collected and information where the different observations on specimients where they are stored in the data about that speciems. Our overall goal is to kind of tight together all the different worlds of biodiversity knowledge into one serious infrastrucutre that people can access and use for researcher decision making.	
Interview 9	05:51		And what is the vision behind it?
Interview 9	06:00	The vision is to provide an open source data for. An open source for people to upload data. So it is free to input data and free to download data, information (unv.) So it is a very broad vision on depth in biodiversity. Very focused on where biodiversity is. We provide the information to people use the information for every purpose they need. Our goal is to provide the best and detailed information on where biodiversity is.	
Interview 9	06:35		So my work now is about success and success factors. So how do you generally define success or a successful platform?
Interview 9		That is a tough one. I guess the primarily manufactureers of (platform) in the past have been researchers. And within the scientific literature there is a (-) history of use of aggregated data. And (platform) about five years ago started a very detailed process in which is can track how people use their data.	
Interview 9	06:45	So we have probably the best mechanism for seeing how people use our data so we can measure our success in the number of scientific publications that are ppublished each year. And we can actually track those and see how it grows over time. And we see what different types of research using our data. So that is one way we measure successes by the scientific literature. And we have a point of firewall tracking system for that.	
Interview 9	07:52	Another way to measure success is trough our network. We are secretary of people here working in (town). But we are a diverse network of people in almost every country who are actually mobilizing the data - so digitazing data in their museum. (unv) And then using our standardisation to allow the data to flow up to us, or let us access that and then we can take that data from tens of different places or actually thousands places and put it allin our data set.	

Interview 9	08:30	In one way we measure success by the growth of number of people who are putting their data in our open repositories. We have literally twenty, ten to twenty new data sets every day that comes in. So we measure success by data growth. We also try to measure by data quality but that is a bit more diffcult. And we also measure by participation organisation and participating countries. So we measure success. And we have more countries and more museems participating - so a good measure of success for us as well.	
Interview 9	09:12		Do you have more to this topic?
Interview 9		We have - success. What are the measures of success? I guess we can also point to the quantifiable size of the secretatiat, our budget and things like that which I guess are not good measure of success because we measure our success by the data that comes in how the data is used. So data uses is really the best success metric.	
Interview 9		The citations in the scientific literature is the best way we have in doing that. As well as measuring the number of times that the data is downloaded by someone around the world. We have a metric of every time someone downloads a data points and measure that counted.	
Interview 9	09:20	And some we measure the amount of data coming in and the number of data coming out. And then futher of data coming out we can measure how it is used during the citation tracking system trough the literaute. That is pretty easy for us to measure the data coming in, data coming out and the different categories within the data for example it it data just on vertebral data, is it data on plants or is it data on bacterial.	
Interview 9	10:40	We can measure success of different types of biodiversity, different geographic areas, so we can get ninety of where the success is. For us it is more a question where the data gaps are. So if there is an area in biodiversity around the world that is for example insects in subsaharian africa. We can measure how much data is coming in from there. And if it is not sufficient on making decisions on biodiversity and we can try to get people to mobilize data from that area on insects. That we have more data in our data base that can be used to / there is more data for better decisions.	
Interview 9	11:25		Coming to a complete different question. What is the organsiational structure behind (platform)?
Interview 9		Ok, so we are a little bit different. We are an inter-governental organisation. So we all of our, we are founded by national governents, by participants who signed that non binding memorium of understanding. And currently we have 59 countries that have signes onto that and about 30 of rganisations.	

Interview 9	11:35	About 40 of those countries are actually do paying numbers. so countries, their national governents, usually their science ministry, enviromently ministry or research ministry, take on a commitment to fund based on their gross GDP to fund the secretariat. They also fund the national network as well. So there is not only the (platform) secretariat but each country has its own node how we call it which is various grately from a part of a persons time to have in several staffment in various (unv) what hey are able to do based on their funding and their comittment to the national government.	
Interview 9		The national governments are funded as secretariat where stuffed with 27 people. But then we have these nodes of all over the world - 59 different countries. That have stuff members from one to three to five. Actually, going out and trying to work with the universities and museums in those countries to get their data mobilized and into the network. So we go from a very small secreteriat in (town) to these regional areas where we have countries.	
Interview 9		And in each countrie there is a network of people who are suppling, that are data basing and mobilizing data that flows up in the secretariat. We do then data mangement. And wen people come to the secreatiat web page in order ti use the data. The structure is organised in a centralize strucutre and secreteriat.	
Interview 9	12:56	But then very diffused on a local level. There are actually thousand of institutions around the world a part of the (platform) network because their data is flowing up to be mobilized by us. We have a governent structure in which each of these participating countries is a member of a governing board.	
Interview 9		And the governing board elects an executive comittee - about people. The executive comittee is the oversight of the secretariat. The executive comitte meets several times per year. The governing board meets once per year. In the annual meeting where we disuss and approve the board programm and the budget for the upcoming year.	
Interview 9	14:20	A large part of the secretariat is to come up with a world programm to mobilze and use biodiversity data within secretariat but also promote the network to do their work. And then the governing body gives us some budget and then we allocate that to different work programm to make this happen. The governing board oversees that process to this annual meeting.	
Interview 9	15:15	And the elect that executive comittee to be the more operational oversight on a, meeting couple of month to discuss stuff.	
Interview 9	15:30		For my understanding: how are the decisions made?
Interview 9		A lot of it falls to me as (personal). So i have the decisions over the budget and the world programm. I finalise that and then my decisons at a secretary level needs to be approved by the governing board. So I have to take my ideas to the governing board. The implementation of the world programm falls here under the secretariat.	

Interview 9	15:37	And then each individual country has room making this, saying what they gonna do with their budget in their country. And that is decided independently in the secretariat because their funds are coming directly to them. Some country have many funds, some countries have more. So it is very variable what countries can do.	
Interview 9	16:34	But the secretariat has a lot of autonomy to involvement the vision of the governing board but we do have the approval of the yearly work programm and budget.	
Interview 9	16:48		How are the different responsibilites ditributed? The nodes are independend more or less?
Interview 9		The nodes have the kind of room to structure as well. The nodes selforganise into a structure what the regions - for example here in (region) are 20 countries that have participants nodes - and they have organised themselves into the (region) regional group. Where they have the regional, two regional representative that kind of coordinates the work here in (region).	
Interview 9		That is a very minor part of the role, trying to look for new iniatives and trying to help countrioes join the (platform) network. That is kind of the work this regional representative. And the regional representative come to (town) to the secretariat once a year plus the governing board so years so twice. as well as phone calls to provide advice to the secretariat on how people with the world programm and the nodes.	
Interview 9	17:00	so the secretariat provides information to the nodes to this nodes steering is called and then they provide that information to the countries and then give that information up to the node steering group as well.	
Interview 9	18:24	Those are all elected from the countries that is not partipated from the secretariat. So we get feedback through all the countries particpiates (unv.).	
Interview 9	18:34		And how many employees work all together for the platform. You said 27 in the secretary.
Interview 9		Yes, that is the only number I can give you. Because up to said it is basicly impossible. We havent tried to quantifiy that. Because every single represenatative out of the secreatiat is probably more than one role. There are - I should say that- there are a few nodes, countrie nodes, in which they have fulltime person just to work in (platform).	
Interview 9		For example here in(town) there is one full time person who is employee of the (institute) standardisation fazility. So for (country) there is one person has a official representative of (country). Other countries might not official but it is somebody who does the (platform) work and has three other jobs as well.	
Interview 9	18:45	Other countries might have two to three people just doing (platform) work. That really depends on buying in and support they have from the country. That is kind of official (platform) network but it is really important to count as well all the museums around the world. Because there are people in this museums who are really are important members of our (platform) network.	

Interview 9	20:00	But there are not official (platform) people.	
Interview 9	20:10	So they will be data basers, IT specialist, every museum in the world who are formatting data and working as part of their job is to get the data from their local instituions to the (platform) network. The amount of time they spent in the (platform) network is very hard to quantifiy. But it is, that network on the local level is very broad but very dificult to quantify. So the executive it is 27 and after that it gets very difficult to quantify. We have been asked to try that but we havent figured it out how to do that.	
Interview 9	20:56		Coming to a different question: how do you strategically position (platform)?
Interview 9		We have been around now for (number) years and we have captured very the well defined niche in biodiversity niche in biodiversity informatics. So that we really dont have much of any competition in that field. We work very (unv) with other initatives which are complementiary to (platform). We have this position because the countries decided that this is how they want to use their money for this global infrastructure.	
Interview 9	21:09	And they think we are doing well and they dont think there needs to be competitions directly for our niche. So we, it is important for us that we stay within that niche. Otherwise it could be position, position could be difficult because then we might be seen as competitors or something else what we dont really intend to be. But there is certain enough to be done what is in this niche with different data types we would stay busy with this particular niche. Does this make sense?	
Interview 9	22:33		Yes it does. Thank you. You mentioned that the platforms is funded by governments and so on? Are there any different ways of funding for the platform?
Interview 9		We have this called core funding. Is just from the national governments and that is in euro per year/ (unv.) it depends because some countries can pay it per year, addition they have to do. We have over the last few years we had some small supplementary funds. For example we had a major iniative from the EU.	
Interview 9	22:47	It is a project in (region) over five years or so to mobilze data in mainly in sub saharian africa, a little in carabiean and (unv) it is basically we manage a grand programm to people in individual institutions in these parts of the world to data base digitazed biodiversity data and to bring that into the (platform) network.	
Interview 9	24:00:00	That is something that has been a part of a supplementary fund. We have some other small, very very small supplementary funds from indivudal projects. But the major funds come from the core funds from the countries and from so far from the EU DEVCO Grand. But we are trying to find more funds like the (fund) Fund to expand that data mobilization fund in the global south. So that is something we are looking at expanding but it is difficult to find money to do so.	
Interview 9	24:45:00		And what are specific goals of the platform you want to achieve?

Interview 9	24:52:00	So, we want to (-) the goal is to be have the best data for data use for research and decision making. And make the very high quality as well so. The goal is kind of ambigues one. In that regards we want to be able to also expand our data types with this new technologies and new initatives that allow us to make (unv) in order species have been observed such as DNA sequencing. So we are looking for bringin in new data types that we think can be relevant for research and policing. That is one of the initiatives to be more relevant. What was the questions agains?	
Interview 9	25:50:00		What are specific goals?
Interview 9		Specific goals yes. We would like to expand the data mobilization from areas of data gaps. So we know what our data, where we have data in our data base. But it is not there we know biological speaching biodiverse reach areas in the world. Because the areas we have the most are the richest countries because there are more able to digitaze biodiversity data.	
Interview 9	25:55:00	So a major goal is to fill those data gaps especailly in sub sahraian areas, tropical (region)s, tropical asia which we know are highly biodiverse and highly (unv) but we have less data from those area than we do from northern (region) simply because there is less work done in those areas. So a major goal of ours is to get more data from those areas not only for the global network but also for - more importantly - for the local countries that they have data through base enviromental decisions its on.	
Interview 9		That is the major objective of the programm funded by DEVCO is to mobilize the data gab periods. That is a major goal. And then we have other majors where we just maintain an infrastructure that grows with the data growth and is robust to more data and new types of data. As soon as we get (unv.) we have to be nibled in our infrastructure to handle that and be able to display and (unv) that data out to other people.	
Interview 9		That is a constant goal sort of be the strong infrastructure in that regard. And then there is the kind of issue with data quality making sure the data is of highest quality. Basicaly we recieve data from anyone and we get variable quality of data. I say quality that will be mean that some data some are people missing or filled in incorrectly.	
Interview 9	27:10:00	Some of them can fixed as the data comes in but some of them isnt. So it is constant work of ours to include the quality of the data. And to do that we have to work with all of these ten of thousand of data providers around the world. That is a goal of ours.	
Interview 9	28:40:00		And how do you controll the goals you set?
Interview 9		I am working on the developing the next strategic plan. So, we have five year strategic plans. The current one runs to (year). And we are trying to think about the next strategic plan will run (year) to (year). This means to be governing (unv) in this topic of conversation now that in order to kind of see where we are in the field and help with the strategic plan - above 1,5 year ago, the commission a review (platform), that basically 20th anniversy.	

Interview 9	28:50:00	In this we view we put several recommendations, it is very fair and i am quite happy with the report - recommendations where we are and what the (platform) user community in large thinks we should go. So really good recommendations to where we go.	
Interview 9		We are now discing a starting of processes, looking at those recommendations, deciding which ones are priority for the secretariat and then have a consultation process with all these nodes around the world. But I think the priority should be. And we have a meeting in late march in which we all start bringing these ideas together.	
Interview 9	30:00:00	And then after that we will form a lately strategic plan to meet these recommendations and put forward a new set of goals and then - the consultation process and then hopefully the (unv) the goals that we come up with will be crueded by the governing board which will be in september. So we have a very robust process to come up with goals based on outside recommendations and based on interations and discussions with our global network.	
Interview 9	31:10:00	And that recommendations come from the executive commetiee and myself and then we will take that the governing board and there discussion.	
Interview 9	31:21:00		And what are difficulties and challenges in managing (platform)?
Interview 9		So. Imagine from the discussion there is a lot of different, very variability in the national nodes. Some nations that are supported and strongand others dont get much support from the government and arent able to provide the support at the local level that they would like or we would like.	
Interview 9	31:30:00	So it is a constant struggle to give as much support on the local level that we can offer but we really dont have the resources to fund the national networks themselves. As the secretary we just have money to support national infrastructure. We are too strict in the national nodes (unv) the data flows robust it shows the (unv) so in order to help the national nodes we have a process last at our last governing board meeting in which we discussed the value propsitions.	
Interview 9		How do the nodes in our network consider the relationship to the secretariat and (platform) in general and how do they communicate about it. So we are probably together those ideas that we learn from that process and we work on a communcation strategy towards the nodes in order to help them self promote (platform) around countries and to get better (unv) from the national governements.	
Interview 9	32:40:00	So a value proposition strategy for the work and the researches in those nodes. So we try to train and boost that. Sorry what was the question again?	
Interview 9	33:33:00		What difficulties and challenges in managing the platform?
Interview 9		Another one is that we have poor representation in part of the world particular asia. We do our best with mind reaches. We are a small secreatariat, we dont have any ties or (unv) (country) speakers. We use the DON languages and we have the most of our web page in the un languages.	

Interview 9		Our footprint asia is weak. Partially because of language and partially (unv) as well. And we have difficulties for us and that can be a focus for us in the future. We are getting data from flow, dara coming from asia but we dont have that national participation really brings our strength.	
Interview 9	33:34:00	And then we have the what I have metnioned before - the neccessity maintaining the actual computational infrastructure as the data standards change and there is types of data coming in and amount of data. So there is a continue process of making sure our infrastructure is robust to various elements.	
Interview 9		There is always the funding marks as well. Right now the country participation is reasonable. But our funding levels from national governents has not really changed in ten years. And we had some ountries drop of and come back on. The stability in the funding is always an issue.	
Interview 9	34:52:00	And we give more a consistent funding from countries would be very useful and helpful for us to strategic planning. But we do have a kind of a good problem in that. This was part of the twenty year review that emphasize that there is really high expectation for what (platform) does. That we are able to produce a lot and product. There is high expectation that we can add more, but we just dont have the resources to do. One of the risks we have to manage is the expecation that we can do more than we actually can.	
Interview 9	36:05:00		How do you encourage researchers, institutes, museums and so on to actively use the platform?
Interview 9		We do have / we have a good of force staff who does particiaption and engagement. There will it is more to get more people involved in data mobilization. So we do this all over the world bring people bring (platform) knowledge to people so that they know what we are and (unv)involved .	
Interview 9	36:20:00	(platform) is quite well known within the research comunities. There is a lot of work to try to transplay the researcher interested and the government interest so that the nations will join the network because the national governments, people who are not scientist might not know about (platform).	
Interview 9	37:08:00	We do have a small group - and I gues I am a part of this - in which we reach out to and attend scientific society meetings, researchers to tell them about what we do is (platform) and to get feedback on the systems so that they can, hopefully use (platform) data. So we do this outreach. of curse we have lots of different of platform outreaches trhough our webpages and industed through our network.	
Interview 9	37:48:00		And how do you ensure that people uplaod data in sufficient number and quality?
Interview 9		So we have what we called an IPD migrated - what is it called? Anyway, it is a piece of software which informs international recognized data standards and the data providers need to put their data to the software and puts it into a specific format we need. And if it doesnt conform the data is not uploaded.	

Interview 9		That is a part of the role of the nodes, the main actions what a node manages does in a countrie on the national level is to approve data sets. So talk to people that whant to provide data to (platform) and show somehow how to do it. The data helps the data publishers with that process.	
Interview 9	37:57:00	So that is a major role of the national node. When data publishers want to publish but there is no national node then they have to use publishers / they have to use designed PTs from different places and it gets a little bit more difficult but we need to have data publishers in countries that are partiipants.	
Interview 9		So that is kind of minimal standard of data coming in but there are also (unv) but it could be data errors such as the amount of longitude and magnitude could be swapped so the number are in the wrong order so we do have anywhere from letting new ones where we go with the data set and try to find this simple errors and have them connected.	
Interview 9	39:10:00	But we dont hold the data ourselves. The data is held by the local research insitutions. So we dont get in and change the data. Our model is that the data is / the principal data is at the location. So we ask the local data provider to check data and to make the change there and then they send the data back to us so that there is only one version	
Interview 9	40:00:00	So that is part of our proces of data quality. It is very difficult and time consuming with the publishers.	
Interview 9	40:23:00		Do you have further requirements for the submission of data?
Interview 9		There is within this standards some metadata that comes more with. So when every new data publishers publishes a data set you can go to (platform) and every data sets probably ten of thousands and every has ist own data page which tells you (-) these comes in from the submission, what kind of data it is, who submitted it, what instituteion.	
Interview 9	40:30:00	The basic data about it. And then give this all of the currencies the data that comes with it. And with this we monitor how much it is used. We have a very robust system of showing what is in each data set. That is up to the data provider to put the data into we get ten to twenty data set every day.	
Interview 9	41:28:00	(unv)	And in this context: how do you ensure trust in the data?
Interview 9		That is a constant issue. Because some people dont trust (platform) data because of these issues. We work very hard to make sure that the data is make people note that a (-) percent is spent for certain uses. We have a very robust (-) system of (--) you can download the data and use to tell you what different tyoes of data to use different types of data. So it is possible to filter the data to the uses aspects.	
Interview 9	41:40:00	And it is difficult / we spent a lot of time people find errors in the data. And trying to get those corrected is a major part of one of our teams. But in general people do know that the data in (platform) is heterogenious and they need to be careful and using in the best scientific practises when using it.	

Interview 9	42:47:00	Which means to editing it. We are working towards having some more reference data sets that are expert curated ready by the community. Actually we are not having those we are working towards that. So expert curated data within (platform) in which would need to be creditet / editet per use. That is one way we are trying to get more trust in the system. But that is work in progress.	
Interview 9	43:24:00		You already mentioned the scientific work. So my question: how do you ensure that the user of (platform) deal scientificly correct with the use of data?
Interview 9	43:40:00	We dont. We /It is a completly open source, open use. We dont / they / we oblige them to cite they data that they donwloaded. But how they interpret the data and alyse the data is completly to them. You cannot probably not at all. To track /	
Interview 9		But we try to track the use of the data but we dont / that quality in the data of ours is something that we cannot. The community is pretty god. There are a lot of papers, there are papers every few month in which are people actually looking at the quality of our data which helps us so they (unv) this is good this bad, this is / the data could be imporved if (platform) could do this. So this is a good way of the community to help us to improve the data. But we dont get in the critique of data analyses of the data.	
Interview 9	44:45:00		What difficulties arise when exchanging data via (platform)?
Interview 9		I guess would be the making sure that the user understand the data that they have. Cause right now we have a mixture of data from museums, data from citizen science projects and data from literarute and data from enviromental studies. And each of those data types and subsets what they need the different.	
Interview 9		And the user really need to understand which component of the (platform) data they have when they do the analysis. That (-) is a constant work is to get that message out that give that information is much more possebile to use so they can interpret their data as best as possible.	
Interview 9	44:53:00	And can (-) you know data that might not be as (-) accurate as they need to their type of analysis. So we call that fittnes for use and we have had several working groups in the past and we kind of we started one this your, two this year in which we have users from the community make recommendations for us and then we try to met thos erecommendations and get to work back up to the community.	
Interview 9		So there is a lot of community input and it is sort of the end of the year time frame. It is not quit but it is pretty robust. The machanic goes back to the expecation management. We have a lot of these ideas coming from the community we have not the ressources to implment them all.	
Interview 9	46:20:00	We need to be very careful about what we ask to the community because we cannot develop that expecations. Because we cannot that we dont have the funds, hardly infrastructure to do. It is about us.	
Interview 9	47:02:00		How do you describe the community and the user of (platform)?

Interview 9		The community is really the basic community is that started (platform) was of the national history collection around of the world. So any hstory museum was the orignial user group for (platform) both for data entry and data use. But now that has expanded. It is now have data coming in from other sources like citizen science projects, genomics projects.	
Interview 9		And then the user base has expanded as well. Is still mainly researchers but also more applied research. That gives a copy on our website. We do a science review every year which own what different types of data. Or what kind of (-) how the data is been used for entr, what types of questions is now being used for factor tricks of the human user.	
Interview 9	47:08:00	Some for they will call (unv) of course (unv). So the type user (platform) user data has expanded as well. It expanded the data types. So we need to be aware of daily user that could be novel from the data and bei agil to bringing new data that could meet a need that we not previously know about.	
Interview 9	48:45:00		And what functionality does the platform offer?
Interview 9		So for each data set / so each data point of (platform) is a webpage which tells you if there is a point on the map and it gives you the metadata that cames from the either the institution or science projects, that tracks where that is. And then it gives you on a species there is an aggregation of the point, 1,5 millionen species pages.	
Interview 9	48:50:00	Wo how good you see a map with where that all those occurences where found. And then with links everyone of those occurences which is the layer below. Pus there is information on all the all the fotographes that came with those occurencies. Sometimes there will be hundreds of photographs that are taken. And there are also be the taxonomy which is kind of the (-) the infrastructure background, information backbone that ties everything together is the taxonomie. The taxonomie needs to be slethes with page as well.	
Interview 9	49:55:00	So there is this deep network of information from individual occurences aggreated up to the species and of course it can be aggregated to higherlevel. So that is that information that people really want. That indivudal information.	
Interview 9		Also we are starting to bring in checks as well. So for each species you can get indications wether it is considered an invasive species in a certain country. So you can see where it is originally ranges plus where it is expanded because of human intervention.	
Interview 9	50:30:00	And then there is also, that is a kind of the output they generate all the inputs the (unv) for each data provider each museum each data set that condense inforkation about the data come in at different levels. And then information how it goes out as well, and if it has aggreaget with other data sets during the standardisation process.	
Interview 9			Sorry. I just had an eye at the timing and I have four questions left.
Interview 9	56:42:00	Next Meeting	

Interview 9	1:40		So the one special aspects about (platform) is that it is located within the scientific community. How do you think that's the scientific culture influence the use of the platform?
Interview 9	1:50	Yes, so that my mind is kind of shift incentive community from closed data to open data. And (platform) is kind of been working to make that transition and making people realize the benefits of open data. So I think we've played a role in facilitating the knowledge that open data is important by how we operate and making this a focal point that all data is open. So I think that that has been a major part of our success, especially over the last few years has been getting the community to buy into the open data. Part of our	
Interview 9	2:44		and what development Do you see for the platform in the next 10 years?
Interview 9	2:52	Okay. There will be a need to Shift to new types of data coming out of new technologies. So we're going to be getting data from DNA sequencing technologies and also data from artificial intelligence. So we're going to have to adapt our data model to bring in new types of data and types of information in being able to organize handle that disseminated going to be a major part of our work for the next 10 years as the types of data that are needed and useful, and by virtue day that are going to change due to technologies.	
Interview 9	3:40		And what does the success of the platform depend on them?
Interview 9	3:48	A lot of its gonna be depend on being able to communicate the value of the new data and how to use it appropriately. Because a lot of our users aren't going to be Now each building a new types of data. And we're going to have to make sure that we can communicate the value without the use of the data to to our community, if we aren't able to show that value that will be a major issue for us.	
Interview 9	4:23		And do you see what aspects you rely on in the future?
Interview 9	4:34	Right now we're funded by national governments. So there is always the potential of national governments deciding they don't want to fund this type of research. Right now, there's no indication of that. But we have to be cognizant of that and look broadly for future funds to support our work and broaden our Work. We're doing a bit of that. So we have to solely rely on how we're doing things, to try to be nimble to adapt to changing conditions that might come our way.	
Interview 9	5:14		So do you have anything else you want to tell me about challenges about success? Or whatever comes to your mind you want to tell me?

Interview 9		We are a global network of people in infrastructure, but whatever major challenges, I think we actually do pretty well as balancing the demands of, you know, richer and poorer nations in the biodiversity data. So we have advanced nations that have a lot of data and infrastructure. And not a lot of biodiversity that we have poor nations that have a lot of biodiversity, but not the infrastructure and that the ability to mobilize That data.	
Interview 9	5:26	So they are, each of these groups has different demands on us. And we have to balance it was competing today and try to get a deep get ability for the developing nations to mobilize data can use the data, while still being unable to effectively bring in new types of data from developed nations. So that's always a balancing act for us to make sure to satisfy both these two communities that we serve.	
Interview 9	6:38		Great. So this were the remaining questions from last time.
Interview 9	6:45	That was fast now. And just a few points for my statistics because I tried to quantify the platforms I'm evaluating so the software behind the platform is developed by yourself, right?	
Interview 10	Time	Interviewee 10	Interviewer
Interview 10	04:43		Und wie ist dann die Plattform entstanden?
Interview 10	06:18	(anonymized)	
Interview 10	06:32		Was ist denn dann das Kernangebot von (platform)?
Interview 10		Bei (platform) ist das Kernangebot, dass denke ich, die Funde anderer zu sehen, also wirklich die gesamte Flora zu sehen, wo sind die Arten und selber die Sachen melden zu können. Das gibt es doch mittlerweile für relativ viele Arten Gruppen, also für die Vögel gibt es ja die (unv) COmmunity, da laufen die Leute letztendlich auch mit ihren Handys rum und tragen Vogelbeobachtungen ein.	
Interview 10	06:35	Und die haben viele Millionen Beobachtungen pro Jahr. Bei den Pflanzen ist das ein bisschen weniger. Aber auch da gibt es großes Potential. Da gibt es jetzt gerade eine Handyapp, die das mit Foto erkennung macht. Also da ist die Einstiegsbarrieren och geringer, du musst die gar nicht kennen. Und da ist im letzten Jahr eine million mal die Schaafsgabe fotografiert worden. Also da ist noch viel Luft nach oben.	
Interview 10	07:35		Ist das denn - eine Nachfrage zu (platform) - ist das denn Community Science?
Interview 10		Ja, ist es. Was wir gerade haben - oder noch anders. Es gibt ein paar Gesellschaften, die sind dann fünfzig oder hundert Jahre alt. Wo sich Leute treffen und draußen zusammen rum laufen und sich gegenseitig beibringen, wie die Bestimmung von Arten funktioniert. Wo dann die Arten vorkommen, die also da an der (platform) interessiert sind.	

Interview 10		Und die haben diese Datensammlung auch primär gemacht. Und staatliche Aufgabe, die werden dann ein bisschen unterstützt vom Landesamt für Naturschutz und geben dann unter bestimmten Bedingungen dann auch ihre Daten weiter. Das ist aber auch immer in Diskussion darüber, wie sich die Community sich wertgeschätzt fühlt. Also in Mecklenburg-Vorpommern ist das eine relativ aktive Gruppe, die treffen sich einmal im Jahr (unv) in Brandenburg - das sind dann 150 Leute oder so.	
Interview 10	07:42	Also insgesamt sind das so 250 oder 350 Leute, die sich da ab und zu - regelmäßig treffen sich so 50 Leute. Das sind die besten Artenkenner, die wir haben. Das gibt es in jedem Bundesland oder auch zu jedem Bundesland und da gibt es dann noch eine weitere Organisation, die sich um (platform) kümmert auf Bundesebene. Das reale Vorkommen und über die Arten zu Informieren ist dennoch ein relativ großer Kreis.	
Interview 10	09:25		Nun handelt meine Arbeit ja von dem Erfolg. Was ist für Sie persönlich denn Erfolg oder eine erfolgreiche Plattform?
Interview 10		Das kommt drauf an, wen sie fragen. Wenn sie mich als Wissenschaftler fragen, dann sage ich, dass ich die Datensammlung erfolgreich auswerten kann. Das hat im Prinzip 20 Jahre gedauert, also das war eine mühsame Arbeit, das soweit zu kriegen, dass man da auch wirklich eine Auswertung machen kann. Wir haben ein wissenschaftliches Projekt beim IDIF in Leipzig, das ist das (unv) Zentrum, wo wir versuchen aus all diesen Daten Trendanalysen zu machen.	
Interview 10		Wie ist es denn bei der Flora, wie ist es den Libellen, wie ist es den Laufkäfern ergangen in den letzten Jahrzehnten in Deutschland. Und dafür versuchen wir alle Daten zu mobilisieren und verfügbar zu machen für diese Auswertung. Und da gibt es die ersten Auswertungen, in diesem Jahr wird auch so einiges rauskommen; da brauchen wir eine Menge statistische Verfahren, die eigentlich nicht dafür geeignet sind, diesen Trend abzuleiten.	
Interview 10		Sie können sich vorstellen, das ist ganz unterschiedlich gewesen in verschiedenen Jahren, wie viele Beobachtungen die Leute gemacht haben, tendenziell nimmt es zu. Die zahl der Beobachtungen, die wir publiziert bekommen zumindestens.	
Interview 10	09:30	Und daraus kann man natürlich nicht ableiten, ob es den Arten gut geht, nur weil man sie öfters beobachten kann. Das heißt, man braucht eigentlich eine Abschätzung der Beobachtungsintensität oder irgendwelche anderen (unv) um die Trends der einzelarten Ableiten zu können.	
Interview 10	11:06		Nun haben Sie ja gesagt, Erfolg hängt davon ab, wen man fragt. Welche anderen Erfolgsmaße sehen sie denn noch?
Interview 10		Achso, die Teilnehmer, die jetzt ihre Daten melden, die sind ja eigentlich das wichtige. Also damit die Plattform funktioniert. Und in der Folge, dass sie das Gefühl haben, dass ihre Arbeit was wert ist - weil sie sichtbar ist. (-) am nächsten Tag ist dann ein gelber Punkt auf der Verbreitungskarte zu sehen.	

Interview 10	11:10	Sie können damit arbeiten, sie können auch andere Daten damit einbeziehen und das vergleichen. Sie kriegen auch eine Rückmeldung, also die Beobachtungen können kommentiert werden "tolle Sache" oder "oh ne, das glaube ich dir aber nicht" oder "hast du mal einen Beleg? oderFoto?". Also es gibt eine Kommunikation darüber. Und das ist eine Wertschätzung. Das ist Motivation um da mitzumachen.	
Interview 10	12:14		Welche Visionen werden denn verfolgt? Sei es bei (platform) oder auch (platform) beispielsweise.
Interview 10	12:20	Tatsächlich aus den einzelnen Sammlungen größere Sammlungen zu machen um übergreifende Analysen zu machen.	
Interview 10		Das heißt bei (platform) war jetzt wirklich die Intention zu schauen global, was gibt es denn da für Daten und daraus ist viel gewachsen. Es gibt jetzt ein europäisches Vegetationsdatenarchiv, was sich daraus gegründet hat und wo schon viele Publikationen drüber gelaufen sind, weil man die realen Daten zusammen gesammelt hat.	
Interview 10	12:30	Und daraus eine wissenschaftliche Publikation daraus gemacht hat. Und bei (platform), das wird jetzt auch genutzt als Metadatensammlung für eine globale Vegetationsdatenbank. Wie eben auch auf weltweiter Ebene die Vegetation zu analysieren.	
Interview 10	13:06		Wie ist denn der organisatorische Aufbau hinter den Plattformen?
Interview 10	13:19	Schmal. Also bei (platform) gibt es ein Steering Comittee. Also eine Gruppe derer, die sich beteiligt haben, gewählt haben. Und tatsächlich treffen wir uns vielleicht einmal im Jahr per Skype. Um die Sachen zu besprechen. Also die Plattform läuft, da gibt es gerade nicht so viel zu entscheiden. Und dementsprechend schlank ist das.	
Interview 10		Bei den Deutschland-(platform)-geschichten, das war etwas aufwendiger weil als Teil eines Forschungsdatenprojektes diese Organisation zu entwickeln, weil eben diese regionalen Gruppen, diese Fachgesellschaften beteiligt sind, weil der Bundesverband beteiligt ist, der Interesse daran hat, die Daten zu sammeln, aber die Regionalverbände nicht unbedingt wollen.	
Interview 10		Und es gibt staatliche Organisationen, die etwa für Naturschutz zuständig sind, die haben eine Verpflichtung bestimmte Reports zu machen an die EU und sich entsprechend darum kümmern. Das heißt, die haben auch Interesse an den Daten. Die individuelle Vereinbarung der Fachgesellschaften auf regionaler Ebene und gleichzeitig auf landesebene um sie damit auswertbar zu machen.	
Interview 10	13:50	Und das alles ist dann zu verhandeln, das möglichst schriftlich hat, damit das auch stattfindet und dann technisch umzusetzen in so einer Plattform wo die Fachgesellschaft sagt: "ja, du hast Nutzungsrecht, aber die Daten gehören mir weiterhin und darfst die auch nicht per (unv)Informationsgesetz weitergeben, wie das normalerweise der Fall wäre, wenn es im Landesamt ist (unv) Forschungsprojekt wäre.	

Interview 10	15:15		Wie viele Mitarbeiter arbeiten denn mit welchem Kompetenzen für die Plattformen?
Interview 10	15:20	Also bei (platform) arbeitet niemand dafür, das ist wirklich komplett / wenn der Server nicht funktioniert dann muss ich irgendjemanden fragen, wen ich in anderen Projekten engagiert habe oder irgendjemanden an der Uni.	
Interview 10	15:44	Und bei den anderen (Plattformen Anmerkung) haben wir Projekte laufen, da sind drei ITler dran, also Programmierer, die (unv) programmieren, ein Kordinator plus nochmal eine Stelle für die Heuschrecken (sagt er so wirklich) (unv) weil wir was neues entwickeln.	
Interview 10	16:10		Und wie sind die unterschiedlichen Verantwortlichkeiten für die Plattformen verteilt?
Interview 10	16:15	Wir / Also bei (platform) bin ich der Vorsitzende des Steering Comittees und so - da bin ich verantwortlich. Es sind ja aber auch nur Metadaten. Das heißt, da kann man jetzt nicht so viel anfangen. Es geht um Kommunikation, um Adressenaustausch, das ist ok.	
Interview 10	16:35	Bei den anderen, wo es um Beobachtungsdaten geht, jetzt muss die Politik sagen, wir machen nur die Technik, wir stellen das zur Verfügung. Die Besitzer der Daten bleiben weiterhin die Besitzer der Daten. Das ist auch bei Veget.web, der Vegetationsdatenbank so, dass die in der Regel die Daten primär akkumuliert haben. Nicht primär die Daten erhoben haben, das sind primär Datenakkumulatoren. Die können weiterhin darüber entscheiden, was mit ihren Daten passiert.	
Interview 10	17:14	Also bei (platform) ist das die aggregierte Botanik von Mecklenburg Vorpommern, die die Fachgesellschaft jetzt - dieh aben auch einen Vorstand. Wenn der vorstand dann sagt: "ne, wollen wir nicht mehr, dann würde ich die Daten wieder rausnehmen.	
Interview 10	17:25		Wenn jetzt irgendwelche Entscheidungen getroffen werden müssen - sei es über die (platform) beispielsweise oder (platform) - wie sind dann die Entscheidungswege?
Interview 10		Also ich überlege gerade. Es werden ja Anfragen an uns gestellt, eine Funktion zu erweitern oder wenn irgendetwas nicht funktioniert oder so. Das ist der eine Weg. Dann müssen die Leute -das kann ich nur von meiner Seite aus (unv) / oft erreichen mich Leute oder meine Projektmitarbeiter und sagen dann "hey, das funktioniert nicht, ändert das doch mal."	
Interview 10	17:40	das ist meistens ziemlich eindeutig, was man da machen muss oder was es zu entscheiden gibt. Umgekehrt wenn ich das (unv) ändern, dafür ist die Entscheidung der Beteiligten notwendig. Dann muss ich irgendwie eine Email schreiben, ein Newsletter, und versuche dann möglichst viele zu acquerieren unabhängig davon, wie es formal geregelt ist.	

Interview 10	18:40	Wie gerade gesagt, bei den (unv) Platformen entwickeln wir das gerade erst, bei (platform) gibt es nicht viel zu entscheiden. (unv) Also bei dem, bei dem es viel zu entscheiden gibt, das ist das europäische Archiv und der weltweit vegetationsdatenpool und da gibt es das sogenannte Steering Comittee. Also gewählte Leute, von den Beteiligten gewählte Leute, fünf, sechs Leute mit einem Vorsitzenden, und die skypen dann so oft wie nötig um Entscheidungen zu treffen.	
Interview 10	19:18		Wie erfolgt denn dann die Finanzierung der Plattformen?
Interview 10	19:22	Über Forschungsprojekte, über Forschungsgelder, Haushaltsgelder der Beteiligten Professoren.	
Interview 10	19:30		Ist das dann eher eine Kurzfristige oder langfristige Finanzierung?
Interview 10	19:34	Die Entwicklung ist meistens Kurzfristig finanziert und das langfristige muss über Haushaltsmittel finanziert werden. Dass man immer wieder bereit ist Zeit darein zu stecken.	
Interview 10	19:55		Als Sie die unterschiedlichen Plattformen aufgesetzt und entwickelt haben, haben sie sich dann auch überlegt, wie sie die Plattform strategisch auf dem Datenmarkt, sage ich mal, platzieren wollen?
Interview 10		Ja, was heißt strategisch und was heißt Markt in dem Falle? Also es gibt zum Beispiel gerade im Rahmen der DFG stärkere Zusammenarbeit und Standardisierung für Biodiversitätsdaten (unv) in Deutschland. Man denkt, das ist jetzt schon alles, aber das ist gar nicht so. Es gibt ein paar Standards, aber biologische Daten sind so verschieden.	
Interview 10		Die Genbank für genetische Daten, da gibt es eigentlich schon sehr starke Standardisierungen - weltweite Datenbank. Das gibt es für viele biologische Daten nicht. Da geht es dann natürlich schon adrum - irgendwie ist man dann dabei - und dann wird man gefragt wenn es darum geht Standards weiterzu entwickeln oder eben auch Netzwerke zu bauen.	
Interview 10	20:07	Das ist der Wissenschaftsmarkt. Und der andere ist, das denke ich bei der Frage, wie stark engagiert sich der Staat sich um Biodiversitätsdaten verfügbar zu machen oder eben biologische Diversität hochhalten zu können. Es ist auch nicht ein Markt in dem Sinne sondern eher ein Interesse das zu benutzen.	
Interview 10	21:30		Nun haben Sie ja erzählt, was sie für Visionen für die Plattformen haben. Haben Sie auch konkrete Ziele gesteckt, die sie erreichen möchten?
Interview 10	21:40	Ja, natürlich. (unv) heißt das Projekt, wo wir mit Bundesländern zusammen arbeiten um diese (theoretisch?) Beobachtungedaten zusammen zu führen. Da gibt es natülich viele für das Projekt. Die Idee, dass die fünf Bundesländer eben dauerhaft zusammen arbeiten. Die investieren in die Infrastruktur und regeln, wie sie kooperieren wollen. Dass man eben eine Arbeitsplattform für die jeweilige Landeseben hat.	
Interview 10		Sei es jetzt eben was gemeinsames für die beteiligten Bundesländer, aber im Grunde genommen auch ganz Deutschland eine Blaupause hat, wie zusammen gearbeitet werden kann.	

Interview 10	22:28		Und wie kontrollieren Sie dann die gesteckten Ziele?
Interview 10	22:30	Da gibt es ein Evaluationsprogramm. Da gibt es dann eben Meetings, wo die Beteiligten zusammen kommen, wo man den Stand erläutert und sagt, so sieht es aus. Und tatsächlich steht dann für dieses Jahr an zu überlegen, wie geht es dann nach Ende nächsten Jahres, wenn das Projekt zuende ist, dauerhaft weiter. Also finanzieren die Landesbehörden zum Beispiel dauerhaft die Infrastruktur.	
Interview 10	23:00		Welche weiteren Schwierigkeiten und Herausforderungen gibt es denn für die Plattformen?
Interview 10		Wir haben gerade einen schönen Artikel geschrieben, dass die Analyse Integration erfordert, die Beteiligung von ganz vielen Leuten, und was man ins Auge fassen muss ist, dass man nicht ausgehen muss, dass das einfach so funktioniert.	
Interview 10		Also wenn ich einen neuen Doktoranden habe, der ist daran interessiert, (region)weit oder weltweit Daten zu analysieren, freut er sich natrürlich, wenn alles frei im Internet verfügbar ist. Und da gibt es mittlerweile ja auch schon eine Menge Sammlungen - das schürt irgendwie die Erwartungshaltung, dass das immer so sein sollte.	
Interview 10	23:05	DAss dann da im Zweifelsfall Jahrzehntelange Arbeit hinter steckt und auch das Bedürfnis gesehen zu werden, in der Regel unentgeltlichen Arbeit, die man gemacht hat, das wird dann nicht gesehen. Der Ruf nach Open Data ist durchaus berechtigt.	
Interview 10		Er ermöglicht uns ganz vieles. Ist auch in der Regel für die, die sich beteiligen, sehr vorteilhaft, aber zurückhaltung ist auch verständniss. Weil die Leute nicht Beteiligt sein wollen, weil es darum geht, dass sie spezialisten sind für Ihre Daten. Deshalb ist dieser Identifikationsprozess, diese Leute, die tatsächlich Ihre Daten zusammen zu bringen, und zu (hinter?)fragen, wer hat welches Interesse, wie können wir das berücksichtigen.	
Interview 10	24:05:00	Das ist ein ganz wichtiger Prozess, der auch eigentlich dauerhauft unterstützt gehört. Also in Großbritannien, wo die Survey viel verbreiteter sind für alle Artengruppen solche Daten zu sammeln. Da gibt es auch ein Biological Record Center. Die sind dauerhaft daran gewöhnt, ihr ehrenamt zu pflegen, zusammen zu bringen, weiter zu pflegen. Das haben wir in Deutschland in der Form nicht, sondern höchstens auf Fachgruppenebene. Also für die einzelnen Arten.	
Interview 10	25:26:00		Nun haben Sie ja erzählt, dass (platform) eine Metadaten-Datenbank ist. Welche Funktionalitäten und Möglichkeiten bietet denn dann die (platform) beispielsweise.
Interview 10	25:35:00	Da gibt es die realen Beobachtungen.	
Interview 10	25:38:00	Also bei (platform) ist es so, dass nur die Informationen über die Datenbanken in den eigentlich Vegetationsaufnahmen da abgespeichert werden. Die eigentliche Untersuchung dann nicht.	

Interview 10	25:45:00	(platform) wird (unv)Nutzeraufnahme auch aus Deutschland abgespeichert und ich kann sie mir auch runterladen. Genauso ist bei (platform) auch die reale Beobachtung in der Datenbank und ich kann mir die angucken, runterladen und gucken, wo ist die gemacht worden, wann ist die gemacht worden, wo ist genau der Platz gewesen. Das heißt, ich kann damit arbeiten, wie hat sich was verändert. Oder wie sind bestimmte Muster, warum kommen die da vor und woanders nicht. Das kann ich bei den Metadaten so nicht tun. Das ist eine Adresssammlung.	
Interview 10	26:33:00		Welche Schwierigkeiten treten denn bei diesem Datenaustausch auf?
Interview 10		Die Schwierigkeit, dass unklar ist, wie viel diese Daten wert sind. Denen (Die?) Muss mal also einen Preis geben. Es gibt etnweder generelle Regeln, also bei der europäischen Vegetationsdatenbank und bei der internationalen Vegetationsdatenbank gibt es zum Beispiel die Regel, dass wenn du deine Daten dazu tust, und es gibt eine Analyse, dann wird erstmal geguckt, welche Daten werden gebraucht für die Analyse und wenn man einen relevanten Anteil dran hat, dann darfst du Coautor sein.	
Interview 10	26:35:00	Du kannst dich also bei der Publikation beteiligen. Oder jetzt eben in der (Beo?), die Zusammenarbeit der Deutschen - oder ne- die Beobachtungsdaten geht, bei den Heuschrecken ist es nicht anders, da geht es eben darum, ja noch eine bestimmte Entscheidungsgewalt zu haben über die Daten. Und gefragt zu werden, wenn es eine größere Auswertung gibt. Es geht weniger um eine Publikation sondern eher darüber genannt zu werden.	
Interview 10	27:45:00		Welche Anforderungen stellen sie denn an die hochgeladenen Daten?
Interview 10		Das ist unterschiedlich natürlich, weil es verschiedene Plattformen sind. Also es gibt immer irgendwelche obligatorischen Felder, die ausgefüllt sein müssen. Da geht man dann in der Regel auch irgendwelche Kompromisse ein. (unv) Aber die Plattformen haben dezediert bestimmte Mindeststandards, damit man die Daten nachher brauchen kann.	
Interview 10	7:50:00	Es ist wichtig, sich auf Sets von obligatorischen Daten zu einigen damit man da eben auf der einen Seite mit arbeiten kann und auf der anderen Seite nicht verhindert, dass die Leute ihre Daten nicht hochstellen weil sie sagen: habe ich ja gar nicht, diese Information.	
Interview 10	8:38:00		Wie wird das Vertrauen in die Daten sicher gestellt?
Interview 10	28:42:00	Indem man von Anfang an möglichst viele einbezieht, indem man prktisch Player zusammen holt, indem die Leute wissen, dass sie bedeutsam sind, große Datensammlungen haben, in dem man transparent ist, indem wie man das ganze verwaltet, indem man regelmäßig Rückmeldungen gibt, und indem man tatsächlich nachfragt sind sie zufrieden.	
Interview 10	29:07:00		Wie bewegen Sie denn Forscher und auch Organisationen dazu, die Plattformen aktiv zu nutzen?

Interview 10		Ja, das geht genauso, dass ich irgendwie Leute frage, sogenannte Incentives mir überlege - das heißt, was könnte verlockend sein. Heißt im Falle der Vegetationsdaten geht es darum, wir können gemeinsame (Forschung?) machen, ihr seid dabei.	
Interview 10	29:11:00	Und weil wir die größten Datenbanken zuerst gefragt haben, sowohl auf deutscher Ebene als auch auf internationaler Ebene und zuerst mit den großen geeinigt haben, wollten dann auch alle anderen mitmachen.	
Interview 10	29:47:00		Sehen Sie denn noch weitere Möglichkeiten?
Interview 10	29:50:00	Ja, das lässt sich für (unv) Grupen genauso machen, damit wir mit in die Diskussion - Bundesregierung, beschließt hoffentlich in wenigen Tagen, die Gründung eines Biodiversitätsmonitoringzentrums, die braucht dann eben auch solche Daten. Und da werden wir zumindest beraten zur Verfügung stehen zu sagen, wie machen wir das für die jeweiligen Artengruppen. Wie können wir die Fachgesellschaft stärken, die ja eigentlich diese Datensammlung als einzige gut betreuen können. Wie kann man die zusammen bringen, damit man tatsächlich auch auswertungen machen kann.	
Interview 10	30:10		Wie stellen Sie denn sicher, dass die Nutzer der Plattforsch die Daten wissenschaftlich korrekt verwerten?
Interview 10		Gar nicht, das macht die Wissenschaft. Das / wir können nur sozusagen versuchen zu gewährleisten, dass die, die die Daten bekommen, zurecht bekommen. Dass sie die Adresse haben, von den Leuten, die sie fragen können. Halt wenn ich selber daran beteiligt bin, jetzt zum Beispiel die Trendanalyse der Libellen in Deutschland haben wir eben rumgefragt, ob wir die Daten bekommen, haben die ersten Modelle laufen lassen, haben dann relativ aufwendig wiederholt die Fachleute gefragt: ist das realistisch?	
Interview 10		Sind das plausible Trends? Lassen die sich erklären? Glaubt ihr den Daten oder könnte es an irgendeiner Stelle irgendwie Schwierigkeiten geben? Was sind die Pitchfalse der Daten? Sodass wir da jetzt einen sehr großen Konsens haben. Die Fachleute, die wir einbezogen haben, sagen: da glauben wir dran, dass die Modelle gut sind und wir sehen auch, was die Modelle gemacht haben mit unseren Daten.	
Interview 10	30:45	Und das empfehle ich natürlich allen anderen auch, das so zu tun. Also rückzufragen. Mitzuarbeiten mit den Leuten, die überhaupt keine Ahnung haben von der Statistik, die jetzt nicht selber Wissenschaftler sind - oder ja genau. Und die aber wirklich in der Fachgesellschaft die größte Ahnung haben von der jeweiligen Artengruppe.	
Interview 10	32:27:00		Nun ist das Besondere ja der Plattformen, dass sie in die Wissenschaft eingegliedert sind. Wie glauben Sie beeinflusst die wisesnschaftliche Kultur die Nutzung der Plattformen?

Interview 10		Also. Tatsächlich muss man viel Überzeugungsarbeit leisten, damit die Citizen Scientist, so nenne ich die Fachgesellschaft, die da hobbymäsig mit beschäftigt sind - unentgeltlich, ohne dass sie da ihren Lebensunterhalt verdienen. Sie haben durchaus Skepsis gegenüber den Wissenschaftlern. Weil die erstmal das Gefühl haben: ah, die wollen unsere Daten absaugen. Die machen damit irgendeinen Unsinn.	
Interview 10		Also da kann ich auch durchaus Beispiele zeigen, dass eben Quatsch bei rauskam, wenn die Leute diese Daten analysiert haben, wissenscahftlich veröffentlicht haben, aber diese Ergebnisse sind einfach Unsinn, weil sie die Schwierigkeiten der Daten nicht ordentlich berücksichtigt haben. Und sind erstmal zögerlich, sage ich mal. Das habe ich schon mehrfach erlebt - und die muss man erstmal überzeugen, dass das eine gute Sache ist.	
Interview 10	32:43	In diesem Workshop, den wir da haben, das ist ein Best Practise Beispiel, der ist auch ausgezeichnet worden mit (unv) biologische Vielfalt, ESPLON heißt das, das ist ein Auswertungsprojekt, wo wir es geschafft haben, die Fachgesellschaften, die Landesbehörden, die ja von staatlicher Sicht auch zustöndig sind, und Wissenshaftler zusammen zu bringen. In quasi gleichen Teilen. Und in vertrauensvoller Atnosphäre. Das haben wir jetzt im vierten Jahr, die Leute zusammen gebracht, (rund/vor) zwei Wochen.	
Interview 10		Und genau da kann die Identifikation passieren. Wenn das klappt, dann ist glaube ich die Wissenschaft ein Motor oder ein Katalysator dafür, dass nicht nur die vorhandenen Daten zusammen getragen werden, sondern dass es hoffentlich nochmal einen Schub gibt für das Sammeln neuer Daten.	
Interview 10	34:21	Das Interese an der Natur, das aus meiner Sicht weit verbreitet ist, ist weitestgehend nicht abgeschöpft, die Bereitschaft sozusagen die Natur kennen zu lernen um (unv) sammeln, da könnte man noch viel mehr tun. Wenn man eben staatliches interesse, wissenschaftliches Interesse und Ehrenamt zusammen bringt, stärker zusammen bringt so.	
Interview 10	35:09:00		Welche Entwicklungen sehen Sie denn für die Plattformen in den nächsten zehn Jahren?
Interview 10	35:11:00	Eine weitere Vernetzung, eine Strukturierung der Geschichte, also sind wir gerade in Verhandlung, die Beobachtungen von der Handyapp kommen, die 500.000 Schaafsgaben, die fotografiert werden, dann in den Plattformen entsprechend auch anzuzeigen. Man kennt, wo die herkommen, aber ich sage mal die gemeinsame Auswertung ist schier unmöglich.	
Interview 10	35:48:00		Und die Entwicklung für (platform)?
Interview 10		Das ist eine so erfolgreiche Plattform, dass die eigentlich zuende entwickelt ist. Die Idee, da die realen Datenbanken weiter zusammenwachsen zu lassen, die gibt es natürlich. Also da wären wir froh, wenn auf nationaler Ebene da mehr Länder sowas hätten. Da gibt es sehr viele Daten, die noch zu strukturieren sind.	

Interview 10	35:50:00	Wir haben in Deutschland höchstens zehn Prozent der analogen Daten - wahrscheinlich noch viel weniger - mobilisiert, also digitalisiert, in irgendwelchen Datenbanken zur Verfügung. Da würde ich die Aufgabe eher darin sehen auf der Organisierungs/Mobilisierungsseite(?) noch mehr (unv). Die (platform) Datenbank ist da sozusagen das die obere Spitze vom Eisberg, die man von oben etwas sehen kann, die das ein bisschen sichtbar machen kann, dass das eine sinnvolle Sache ist.	
Interview 10	36:50:00		Und wovon hängt dann der Erfolg der Plattformen in Zukunft ab?
Interview 10		Dass man die Netzwerke aufrecht erhält. Es geht weniger um die Technik, da gibt es auch immer sicherlich etwas zu tun, um das nicht veralten zu lassen, sodass wir das regelmäßig funktionaler machen, einfacher machen, einfacher bedienbar machen, und so weiter. Aber der eigentliche Auftrag ist die jeweiligen Communities zusammen zu halten.	
Interview 10	36:55:00	Die sagen, ja das macht Sinn, das ist nicht nur eine anonyme Plattform, sondern eine Gradwurzelorganisation (?), wo du auch beteiligt sein kannst, dich einbringen kannst, dich persönlich engagierst, eben auch Entscheidungsbefugnisse hast oder Mitarbeitungsmöglichkeiten.	
Interview 10	37:50:00		Gibt es sonst noch etwas, was Sie mir zum Thema mitgeben möchten auf den Weg?
Interview 10	37:55:00	Wenn man soetwas aufbaut würde ich immer zuerst sagen, Marktanalyse machen in dem Sinne, wer hat denn das was ich brauche. Und zu überlegen, was ist da der Merhwert von dem, was ich da entwickle. Dass dann zu verkaufen und zu sagen, ja, seht ihr das auch so oder bin ich im falschen Dampfer. Und wenn ihr das auch so seht, wie können wir da zusammen arbeiten. Das sind glaube ich die wesentlichen Faktoren, warum das funktioniert.	
Interview 10	38:35:00		Ja super, vielen lieben Dank
Interview 11	Time	Interviewee 11	Interviewer
Interview 11			
Interview 11		Das Kernangebot der Plattform ist… (name) willst du was sagen?	
Interview 11			Hallo?
Interview 11		Ja	
Interview 11			Ja bei mir reißt es andauernd ab. Ich krieg nur die Hälfte mit.
Interview 11		Ah schade. Gut…Dann sag ich da was zu. Das Kernangbeot ist dir Möglichkeit für Wissenschaftler aus der Morphologie, Forschungsdaten aufzuladen. Speziell im Moment in Informationen zu Organismen plus angehängte Multimedia Daten. Das ganze unterstützt mit Literaturdaten. Also Referenzen. Und die Möglichkeit eine sogenannte morphologische Matrix zu erstellen. Also eine Vergelichsmatrix über verschiedene Arten oder Organismen und deren Merkmale.	
Interview 11	04:20:00		

Interview 11		Die erfolgreiche Plattform im Bezug auf (platform) in dem Fall ist eine breit.. ne eine User Community, die sich aktiv beteiligt, Daten hochlädt und diese auch nutzt.	
Interview 11			
Interview 11		Ja ähm…bis dahin… da sind wir aber noch nicht.. bis dahin, dass die Daten, die hochgeladen werden auch zitiert werden. Also das wäre so der Gold Level sozusagen, dass die Daten, die hochgeladen werden immer eine DOI zitiert werden in Publikationen.	
Interview 11			
Interview 11		Da ist (name) jetzt dran.	
Interview 11			
Interview 11		Ok… ganz schelcht den Ball darüber zu geben..	
Interview 11			Hallo jetzt höre ich was
Interview 11		Ja super	
Interview 11			Ich soll was über Vision sagen nehme ich mal an
Interview 11		Genau	
Interview 11	05:51:00		
Interview 11			Also so viel habe ich gehört und dann war die Verbindung weg
Interview 11		Also du bist sehr gut zu hören, Lars. Wenn du redest wird es vermutlich gehen	
Interview 11			ok. Gut dann gucken wir mal. Also die Vision ist, eine Plattform zu erstellen, die ein Knowledge Graph für… ja langfristig sogar Biodiversitätsinformationen liefert. Aber kurzfristig für Phenomtypbeschreibung. Und ein knowledge graph bedeutet, dass die Daten so abgelegt werden, dass sie.. man sagt das so im englischen..machine actionable sind. Also, dass Maschinen die lesen können, dass sie zugänglich sind für Maschinen, dass Maschinen die auch auswerten können, sodass wenn man mit großen Datenmengen arbeitet, die Maschienn einem zur Hand gehen kann, um bestimmte Fragestellungen zu beantworten.
Interview 11			
Interview 11	07:05:00	Wenn ich mir die restl.. wenn ich mir die Fragen in dem Block angucke, (name), was ist konkret mit Organisation gemeint? Welche Leute dahinter sitzen, wie die aufgehängt ist oder was ist damit gemein?	
Interview 11			

Interview 11		Ok also. Die Plattform (platform) wird hier vom(museum) in (town) betrieben und das bedeutet.. also. Betrieben bedeutet wir erstellen die Server und den Plattenplatz und die Internetanbindung. Gleichzeitig sind damit zwei feste Stellen verbunden, deren Hauptaufgabe nicht das betreiben der Plattform ist, aber hier wird vom Help Desk Registrierungsabwicklung und Bugfixing betrieben. Die Weiterentwicklung der Plattform geschieht im Wesentlichen über Drittmittel-Projekte. Also wo konkrete Themen angegangen werden, wie was (name) sagte, die Implementierung der Knowledge Graph Application.	
Interview 11			
Interview 11	08:19:00	Über die Hauhaltsmittel des Museums in (town) plus Drittmittel.	
Interview 11			
Interview 11		Die sind langfristig gesichert, ja.	
Interview 11			
Interview 11		Nein.	
Interview 11			
Interview 11		Genau	
Interview 11		Zur Zeit ist das noch eine Programmiererin, die auf Restdrittmitteln angestellt ist und wir arbeiten an einem weiteren Drittmittelantrag, wo dann wieder Programmierer kommen. Es ist jetzt.. Ende des letzten Jahres ist ein größeres Drittmittelprojekt zu ende gegangen. Da hatten wir insgesamt drei Programmierer eingestellt. Also das sind immer so Schübe.	
Interview 11			
Interview 11		Die jetzt zugenommen wurden über die Drittmittel? Das sind Programmierer.	
Interview 11			
Interview 11		Kernprogrammierer. Wir hatten zwei Jahre lang, ne drei Jahre sogar, hatten wir einen Mediengestalter mit dabei. Der hat die Oberfläche, also die Kommunikation letztendlich mit den Benutzern entwickelt.	
Interview 11			Auf ner halben Stelle aber.
Interview 11		Auf ner halben Stelle genau. Aber weil ein Programmierer nich unbedingt derjeniege ist, der in der Lage ist, die Interfaces so herzustellen, dass ein normaler Benutzer sie auch versteht. Deshalb war und das wichtig.	
Interview 11	10:08:00		
Interview 11		Das ist in eine Abteilung also in meine Abteilung im Prinzip eingegliedert.	
Interview 11			
Interview 11		Die Haupt… jetzt rechtlich die Verantwortung? Entwicklungstechnisch? Welche?	
Interview 11			
Interview 11		Alle? (lacht)	
Interview 11			

Interview 11		Also rein rechtlich was zum Beispiel Datenschutzkonformität angeht, trägt der Direktor des Institutes hier. Der Leiter, der jeweilige. Die Entwicklungsverantwortlichkeit liegt im Wesentlichen bei (name). Ich unterstütze ihn dabei und bei mir liegt die Verantwortung das ganze eigentlich am Leben zu halten. Also die Sustainability, wie man so schön sagt.	
Interview 11	11:12:00		
Interview 11		Über eine Konsensbildung würde ich jetzt mal sagen. Wir stimmen nicht ab, sondern wir diskutieren das aus und versuchen den besten Weg, der auch, das hängt immer ein bisschen davon ab, einerseits wo liegen die Finanzquellen für weitere Drittmittelprojekte, was könnte finanziert werden und wo ist die Lücke, in die wir rein können.	
Interview 11			
Interview 11		Wir haben keine Marktanalyse gemacht, wenn du das meinst. (lacht) Das ganze ist in dem Maße, wie es jetzt entstanden ist, im Rahmen eines größeren Forschungsverbundes entstanden, wo das die Plattform war, um die Datenkommuniktaion und Archivierung durchzuführen.	
Interview 11		Also wir hatten von vornherein, wenn auch kleinen, aber interessierten Markt, der uns auch geholfen hat, die Anforderungen zu spezifizieren und weiterzuentwickeln. Das ist immer so.... in dem Bereich, wo wir da unterwegs sind, ist das so ein sich gegenseitig erhellender Prozess.	
Interview 11		Wir entwickeln ein Stück, stellen das den Benutzern vor und die Benutzer sagen " ja das geht in die richtige Richtung". Dann machen wir da weiter. Oder "nein so gehts nicht oder das ist zu kompliziert". Dann versuchen wir das anzupassen. Also User driven development.	
Interview 11	12:57:00		
Interview 11		Forscher. Also Wissenschaftler.	
Interview 11			
Interview 11		Bioilogie, Morphologie. Und jetzt dann, das ist die zukünftige Ausrichtung, naturhistorische Sammlungen und Biodiversitätsentdeckungen, wie man so schön sagt. Oder Biodiversität allgemein.	
Interview 11			
Interview 11	13:32	Was meinst du mit Austausch?	
Interview 11			
Interview 11		(--)	Also es gibt viele Herausforderungen, weil wir es gerade im Biodiveristätsbereich mit sehr unterschiedlichen Datenquellen und Datenformaten zu tun haben. Was auch ein Grund dafür ist, warum solche Datenplattformen wichtig sind, damit die Daten in einer vereinheitlichten und vergleichbaren Form gesammelt werden können. Also ich weiß nicht, ob Sie das meinen mit Problem oder in der Interaktion mit den Usern, wenn sie Sachen runterladen wollen, da gibt es bisher keine Probleme. Zumindest nicht, von denen ich wüsste.
Interview 11			

Interview 11			Ja.
Interview 11			
Interview 11			Ne, also das Problem ist letztendlich wirklich / wir haben es / es sind mehrere Sachen, ein mal, dass es aus verschiedenen Communities und damit auch verschiedenen Historien verbunden ist - Historien der Art und Weise, wie Daten dokummentiert werden und welche Formate benutzt werden und so weiter und das andere ist, dass wir es teilweise auch mit sehr alten Daten zu tun haben. Also wenn es darum geht, zum Beispiel jetzt im Biodiversitätsbereich sogenannte Occurence-Daten, also Sichstungsdaten, wann ist welche Art wo gesichtet worden, die sind teilweise noch in Feldheften vorhanden. Sind noch nicht mal digitalisiert. Und dann hat man das Problem mit Veränderung von Ortbezeichnung und so weiter und so fort. Also da sind viele Probleme, die den Vergleich der Daten untereinander dann erschweren und wenn man sie einmal in eine digitale Form gebracht hat, die so ist, dass dan Maschinen auch damit umgehen können, ist diese Hürde der Vergleichbarkeit dann ausgeräumt - der Nichtvergleichbarkeit ausgeräumt.
Interview 11			
Interview 11			Bitte?
Interview 11			
Interview 11	16:17		Also, da müssen wir unterscheiden zwischen der Plattform, so wie sie jetzt online ist und unserer Vision. Das, was online ist, ist noch nicht maschinenlesbar, wir haben im letzten Projekt einen Prototypen entwickelt, der maschinenlesbare Daten liefert und für den schätze ich das so ein, dass, dadurch dass man dann Algorithmen entwickeln kann, Programme entwickeln kann, die genau die diese Daten nutzen werden, sehr gut ein.
Interview 11			
Interview 11	17:11	Nein. In Bezug auf Anzahl der Nutzer, Anzahl der zu erreichenden hochgeladen Gigabyte oder was immer man da für Metriken nehmen will, nicht. Wir versuchen möglichst gut in der Community anzukommen, möglichst viele Nutzer zu haben, die Daten nutzen, die Daten hochladen, aber konkrete Ziele haben wir nicht.	
Interview 11			
Interview 11		Aufgrund der Nutzer, die wir haben, wie gut die das nutzen. Wir kennen die Community, die ist relativ klein in der Morphologie. Und von daher ist das ein relativ guter Überblick. Wer das nutzt, wer das nicht nutzt, wir sehen das an den Publikationen, wer das genutzt hat und wer es nicht genutzt hat, so in der Form können wir das evaluieren.	

Interview 11		Also wenn Sachen publiziert wurden in irgendwelchen Zeitschriften, die nicht Daten von (platform) beinhalten, sehen wir, ok, da ist eine Lücke, wobei ich zugeben muss, das ist der überwiegende Teil (-), der es nicht nutzt.	
Interview 11	18:33		
Interview 11		Über direktes Ansprechen und über / ja, über direktes Ansprechen, entweder per Email oder per Kontakt auf Konferenzen.	
Interview 11			
Interview 11		Das große Ziel ist /	
Interview 11			Also eine Möglichkeit ist es / Daten dann reinzubekommen ist die Verwendung von artifizieller Intelligenz. Indem Bilder über Deeplearning-Algorithem automatisch annotiert werden und aus diesen Annotationen dann Phänotypbeschreibungen extrahiert werden. Das ist etwas, was wir in der Zukunft angehen möchten. Weil das große Bottleneck insgesamt in der Morphologie ist das Erstellen von diesen Beschreibungen. Das ist wahnsinnig zeitaufwenig.
Interview 11	19:33		
Interview 11			Genau, da könnte man die ganzen Bildersätze, die im Zuge der Digiatlisierung in den naturkundlichen Museen entstanden sind, auswerten und entsprechende Beschreibungen erstellen und da hat man den Vorteil, dass das Material auch einigermaßen sicher bestimmt ist, sodass die Metadaten auch stimmen und man müsste eine hohe Qualität an Daten dadurch erzielen können.
Interview 11			
Interview 11			Das ist ja immer Angebot und Nachfrage. Also, wenn man viel Content hat und der maschinenlesbar ist, dann wird das auch interessant für Nicht-Morphologen. Und es gibt sehr viele Biologen, die warten darauf, phänotypische Daten in einer Form zu bekommen, dass sie damit arbeiten können. Und im Moment sind diese Daten in PDFs verteckt und unglaublich schwierig für den Nicht-Experten auszulesen und zu interpretieren.
Interview 11		Und die andere Möglichkeit ist die Vernetzung mit anderen Angeboten, mit anderen Portalen, also die Daten dort verfügbar machen. Das machen wir auch schon innerhalb der Biologie. Ein weiterer Weg ist die Anbindung an Verlage, an Publisher direkt. Dass jemand, der publiziert, seine Daten bei uns hochlädt, um dort die Metadaten und die erweiterten Daten zur Verfügung zu stellen.	
Interview 11			

Interview 11		Nein, noch nicht verpflichtend. Also es ist nicht so, wie in der Genetik zum Beispiel, wo die Leute verpflichtet sind, die Gensequenzen, die sie generiert haben, bei (platform) hochzuladen. So weit sind wir noch nicht.	
Interview 11	21:32		
Interview 11			Beides. (lacht)
Interview 11		Ja, ganz klar. (lacht)	
Interview 11			
Interview 11		Einmal, dass diejenigen, die Informationen / Forschungsinformationen haben, die verstärkt hochladen und, dass das Angebot, was wir haben, so nutzbar ist, dass es nicht nur innerhalb der Community nutzbar ist, sondern eben auch, wie ich sage und auch Lars, verschneidbar ist mit anderen Plattformen und dort als Angebot unter anderem zur Verfügung steht. Das heißt, die Angebote dort ergänzt, die Informationen. Und dadurch mehr gestreut wird und ja, genau...	
Interview 11			
Interview 11	22:45	Kann ich aus der letzten Zeit erzählen, zum Beispiel so etwas, wie die Implementierung der DSGVO. Aber ansonsten (-).	
Interview 11			Ja, ein Problem ist sicherlich, dadurch, dass wir in diesen Semantikbereich vordringen / es ist schwierig, Programmierer zu finden, die diesen Hintergrund haben.
Interview 11			
Interview 11		Vom Datenmanagement her ist es kein Problem ist es / auch eine weitere Schwierigkeit ist, wenn sich Anforderderungen am Inhalt ändern. Das dann einzupflegen. Das war auch der Grund / mit einer der Gründe, warum wir das mehr in diese Knowledge-Graph Applikation entwickelt haben, weil dort wird es einfacher. Also, wenn zum Beispiel ganz einfach ein neues Formularfeld hinzukommen soll, dann ist das ein Programmieraufwand.	
Interview 11	23:47		
Interview 11		Das sind unsere User.	
Interview 11			
Interview 11		Wie sie zur Zeit online ist, gibt es die Möglichkeit für den anonymen Nutzer, also den nicht registrierten Nutzer, veröffentlichte Daten zu suchen und herunterzuladen. Das sind zum einen Informationen über Organismen, also wo kommt ein Organismus vor, wie heißt der Organismus, wer hat ihn hochgeladen, was für ein Fundort war das und so weiter und so fort.	
Interview 11		Zum anderen sind das Bilder, allgemein Multimediadaten. Das können Bilder sein, das können Stapel von Bildern sein, PDFs und so weiter und so fort, Literaturdaten plus diese morpholigische Matrix, die ich eingangs schon beschrieben habe. Also eine Vergleichstabelle, wenn man so will, über die Organismen, über die Arten und deren Merkmale.	

Interview 11		Alle diese vier Sachen, die ich nannte, können miteinander verlinkt werden, also Literaturdaten in die Artinformationen, in die Bildinformationen, die Bilder können in die einzelnen Sachen drangelinkt werden und so weiter. Für den registrierten Benutzer gibt es die Möglichkeit, die Daten zu editieren, zu löschen, in Gruppen verfügbar zu machen, also zu teilen, kollaborativ an den Daten zu arbeiten.	
Interview 11			
Interview 11	25:56:00	Der guten wissenschaftlichen Praxis zu folgen. Das bedeutet, Fakten hochzuladen und diese auch zu dokumentieren, dafür eben die Literaturdaten.	
Interview 11			
Interview 11		Wir haben eine kleine Registrierungshürde, nämlich die Leute müssen eine gewisse - diejenigen Forscher oder Wissenschaftler, die sich registrieren oder die Menschen, die sich registrieren, müssen in irgendeiner Form eine Referenz vorweisen, eine Angehörigkeit zu einem Institut oder ähnlichem.	
Interview 11		Von daher haben wir da schon mal eine Hürde darauf, dass das nicht jeder macht und ein Wissenschaftler im Allgemeinen, der diese Plattform nutzen will, arbeitet meistens in einem Forschungsprojekt oder in seiner eigenen Forschung und ist daran interessiert, gute Daten zur Verfügung zu stellen. Wir hatten bisher, in der ganzen Zeit wie die Plattform läuft, noch keinen Spam, wenn man es so sehen will.	
Interview 11		Das schränkt natürlich die Anzahl der registrierten Nutzer unglaublich ein, aber erhöht die Qualität der Daten.	
Interview 11			
Interview 11		Keine.	
Interview 11			
Interview 11		Also, wir haben eine Reihe von Formularfeldern zu den jeweiligen Eintragstypen, diese Formularfelder unterliegen Community-Standards. Also, die Informationen, die ich zu einer Art, zu einem Organismus, den ich hochlade eingebe, sind anerkannte Inhalte, die auch in anderen, ähnlichen Plattformen gefordert werden.	
Interview 11	27:27:00	Zu jedem Eingabefeld gibt es einen Hilfetext mit eventuell sogar einem Link, wo weitere Informationen zu finden sind. Das sind so die Hilfestellungen, weniger die Anforderungen, mehr die Hilfestellungen an die Benutzer. Bei den Multimediainformationen kann alles hochgeladen werden. Das können auch Excel Tabellen sein. Alles, was der Forscher in seiner Forschertätigkeit generiert und dokumentieren will und nachhaltig zur Verfügung stellen will.	
Interview 11			
Interview 11		Über die Standards.	
Interview 11			
Interview 11		Dass die Standards, die wir zur Verfügung stellen in den einzelnen Formularfeldern auch genutzt werden. Wir haben jetzt keine redaktionelle Arbeit im Hintergrund, die die Inhalte auch kontrolliert und dann frei gbit. Das sind im Wesentlichen die Gruppen, die kollaborativ an den Daten arbeiten.	

Interview 11		Ein wirkliches, hunderprozentiges Sicherstellen, dass die Daten auch korrekt sind, können wir natürlich nicht, weil wir die Versuche, die den Daten zugrunde liegen nicht nachvollziehen. Wir haben auch kein Peer-Review-Verfahren, was / wo Dritte erst aufgerufen sind, diese Daten zu / mal einen Blick drauf zu werfen und zu sagen, ok, die sind gut oder die sind nicht gut. Das würde den Rahmen sprengen einfach.	
Interview 11			Den Aufwand können wir nicht betreiben. Man muss aber auch dazu sagen, also in dem semantischen Bereich ist es so, dass wir eine ganz klare Inputkontrolle haben und da ein Datenmodell dem zugrunde liegt, sodass es gar nicht möglich ist, zumindest inkonsistente Daten hochzuladen / logisch inkonsistente Daten. Und dann muss man auch dazu sagen, in der Morphologie ist es so, dass die Bilder eine ganz wichtige Rollte spielen und, dass Morphologen immer auf die Bilder gucken und die mit den Beschreibungen vergleichen und das ist die Qualitätskontrolle, die immer gemacht wird.
Interview 11	30:16:00		
Interview 11		Was heißt ausreichend?	
Interview 11			
Interview 11		Wir verstehen uns als Dienst an der Wissenschaft im Prinzip, als Unterstützung der Wissenschaft. Von daher ist schon ein Hochladen / ist schon ausreichend. Ein Upload. Ein Upload, der auch genutzt werden kann, also publiziert wird auf der Plattform, also für den anonymen Nutzer zur Verfügung steht.	
Interview 11			Kann ich vielleicht nochmal was dazu sagen, was unsere Erfahrungen sind. Wir machen das jetzt ja schon eine ganze Zeit lang und am Anfang / das is auch so, dass das ein Prozess ist, der in der Community stattfindet und am Anfang waren die Bedenken unglaublich groß überhaupt irgendwelche Daten hochzuladen. Und da findet ein Lernprozess statt, dass die, die hochladen auch davon einen Vorteil haben und dass das nicht nur die anderen sind, die dann die Daten nutzen, sondern, wenn man selber hochlädt, dann ist die Chance, dass jemand anderes kommt und sagt, ich möchte mit dir zusammenarbeiten, mich interessieren diese Daten, die du da hochgeladen hast, die ist sehr groß. Und das lernt die Community auch.
Interview 11	31:49:00		

Interview 11			Das ist nicht nur die wissenschaftliche Kultur, ich glaube, das ist insgesamt ein Kulturwandel. Also wenn wir es jetzt mit jungen Forschern und Forscherinnnen zu tun haben, die haben gar keine Hemmschwelle, Dinge digital ins Internet hochzuladen. Wohingegen ältere Kollegen und Kolleginnen da eine größere Hemmschwelle haben. Das ist einfach weil die junge Generation wächst mit dem Internet auf und für die ist das auch normal und die sehen auch den Vorteil sofort, da muss man gar nicht argumentieren.
Interview 11			
Interview 11		Ne, das deckt es im Wesentlichen ab. Auch die Genese des Ganzen, was (name) sagte, mit diesen anfänglichen Schwierigkeiten. Wenn man sich die älteren Uploads anguckt auf der Plattform, dann sind das kleingeränderte Bilder, weil die Kollegen Angst hatten, dass die Daten ungefragt genutzt werden, dass die Lizenz nicht beachtet wird und so weiter und so fort.	
Interview 11		Aber das hat sich sehr stark geändert, also da werden jetzt richtig große Multimedia-Uploads gemacht, wo wir kaum mit der Hardware hinterherkommen. Das ist / das hat sich sehr stark geändert. Diese Sicht auf Daten und Datennutzbarkeit im Rahmen von solchen Plattformen, internetbasierten Plattformen.	
Interview 11	33:33:00		
Interview 11			(name), willst du oder soll ich?
Interview 11		Ne, mach du, das sind genau ja deine Sachen.	
Interview 11			Ein bisschen angerissen haben wir die Frage ja schon. Also wir möchten in diesen semantischen Bereich rein, wir wollen die Datenbank in einen sogegannten Knowledge-Graph überführen, sodass die Daten maschinennutzbar sind und wir wollen die Verknüpfung verstärken zu anderen Plattformen, wir wollen das Datenspektrum erweitern hin zu Biodiversitätsdaten und möchten auch exploren. Wie sagt man das denn im Deutschen? Erkunden oder ausloten, wie weit wir kommen mit einer maschinenunterstützten, halbautomatsischen Generierung von Phänotyp-Beschreibungen. Eben das, was ich schon erwähnt habe, das Auswerten von Bildern, das Anwenden von Deep Learning Algorithmen zum automatischen Annotieren von Bildern und da Beschreibungen raus zu extrahieren. In diese Richtung möchten wir gerne, dass wir uns weiterentwickeln.
Interview 11			

Interview 11	35:05:00		Ja, also da sind offene Fragen, wie weit man mit diesen Deep-Learning Algorithmen da wirklich kommt. Ob man auch / ob sich das auf bestimmte Taxa beschränkt, also bestimmte Gruppen von Organismen und bei anderen ist es einfach zu schwierig, dass das nicht funktioniert. Das sind so die Fragen, die da offen sind. Das ist eine Frage, nicht des Erfolgs der Plattform, sondern des Erfolges dieser Vision, ob man maschinengestützt Daten hochladen kann.
Interview 11			
Interview 11			Also es ist, wenn man darauf seine wissenschaftliche Karriere aufbaut, ist es ein harter Weg.
Interview 11			
Interview 11			Also wenn man viel seiner Arbeitszeit in das Entwickeln von solchen Plattformen steckt, dann ist der Output an Publikationen relativ überschaubar. Das ist inzwischen besser geworden, weil es mehr Journals gibt, wo man solche Sachen publizieren kann. Als wir angefangen haben war das ziemlich dünn. Die Währung im wissenschaftlichen Betrieb sind nunmal die Publikationen.
Interview 11			
Interview 11			Genau. Und der Erfolg / der Erfolgsfaktor sind neben den Benutzerzahlen ganz platt, einfach die Einbindung / oder die Vernetzung mit verschiedenen anderen Plattformen. Und solchen Sachen, wie ich zwischendurch sagte mit Publishern und so weiter und so fort.
Interview 11			
Interview 12	Time	Interviewee 12	Interviewer
Interview 12	04:38		Was ist denn dann das Kernangebot von (platform)?
Interview 12		Das Kernangebot besteht im Wesentlichen darin, dass wir definiert haben, welche Parameter und Eigenschaften werden gebraucht um Enzyme umfassend zu characterisieren. Das ist einmal die Funktion der Enzyme, welche Substrate können umgesetzt werden, welche metabolischen Pfade sind / in welchen metabolischen Pfaden sind die Enzyme aktiv.	
Interview 12	04:42	Das sind aber auch (-) Parameter oder Eigenschaften von Enzymen, für die Anwender wichtig sind wie zum Beispiel die Hitzestabilität oder die pH Stabilität, Stabilität und was hat das Protein für eine Sequenz, was hat das Protein für eine 3D Struktur, wie unterscheidet sich das Protein von anderen Proteinen. Also im Prinzip ungefähr 50 Parameter, die wir aus wissenschaftlichen Publikationen herausziehen.	

Interview 12		Das heißt, eine Miterarbeiterin aus der Gruppe in der Literatur nach, wo gibt es neue Publikationen über Enzyme. Ie werden dann bei uns intern als PDF runtergeladen, werden an die externe Mitarbeiter und Mitarbeiterinnen versand. Das sind Fachleute entweder Biologen oder auch welche, die so als Naturwisse / biologische Journalisten aktiv sind.	
Interview 12	05:49	Die lesen die Literatur durch, extrahieren die wichtigen Daten aus der Literaturstelle, aus dem Paper, schicken uns das ganze als in tabellarischer Form und wir bauen das dann in unserer Datenbank ein nachdem es verschiedene umfangreiche Qualitätskontrollen darüber haben laufen lassen. Und so haben wir im LAufen der letzten dreißig Jahre, eigentlich seit 87 so hunderfünzig, hundertsehcigtausend Literaturstellen ausgewertet. Und das hat dann zu ungefähr 4 Millionen Daten in der (platform) Datenbank geführt.	
Interview 12		Das ist so der Kern. Und daneben machen wir eben noch Data Mining. Das heißt wir gucken international, wo gibt es Informationen über Enzyme, die jetzt nicht direkt in Papern stehen, wie zum Beispiel die (unv), Proteinsequenzen oder auch die 3D, die direkten Koordinaten. Und das wird dann zusätzlich bei uns noch in die Datenbank mit eingezogen zum Beispiel vom (region) Bioninformatics Institut. UniPot.	
Interview 12		Die (platform) Daten. Manche haben. Zusätzlich machen wir noch Textmining.. Das hei´ßt wir wissen natürlich, dass wir nicht alle Publikationen auswerten können. Das heißt weir checke noch wo wir aus Abstrakten oder auch Volltexten aus Papern automatisch über Computerprogramme Informationen arausziehen können und haben da jetzt noch ungefähr zusätzlich zu unseren 150000 3.000.000 Papern ausgewertet, was beispielsweise das Vorkommen von Enzymen angeht, in welchen Organismen kommen die vor oder auch in welchen Organen - wenn das in den Menschen oder Pflanzen sind, kommt das Enzym vor wo es expreniert.	
Interview 12	07:12	Wo sitzt in der Zelle, als o ist das in der Membrana oder ähnliches. Das sind so die drei Komponenten. Also unsere eigenene manuelle Auswertung, dann die Textmining automatische Auswertung und die, der Eibau, die Integration von weiteren Enzymdaten wir wir also aus dem Internet bekommen.	
Interview 12	08:58		Und man kann doch auch Daten selber hochladen oder?
Interview 12	09:02	Kann man auch, das freut uns auch immer sehr, passiert aber auch extrem selten.	
Interview 12	09:08		Nun handelt meine Masterabreit davon, was eine Erfolgreiche Plattform ist. Wie definieren Sie denn Erfolg oder eine erfolgreiche Plattform?
Interview 12		Also einerseits hängt davon ab, einmal Umfangreich und umfassend Daten haben und muss diese Daten dem Nutzer in einer Form zur Verfügung stellen, dass das eben jeder Nutzer die Informationen auch mit sehr wenigen Klick also in kurzer Zeit bekommt. Und das ist bei uns bei den Enzymen natürlich sehr kompliziert, weil die Nutzer von (platform) stammen aus wissenschaftlich gesehen sehr weiten Feld.	

Interview 12	09:19	Das sind einerseits die Biochemikern, die direkt mit Enzymen arbeiten, das sind die Mikrobiologen, die wissen wollen, welche Enzyme kommen denn in welchen Organismen vor, das sind die Mediziner, die wissen wollen, wie hängen Krankheiten mit Fehlfunktionen von Enzymen zusammen. Das sind die Biotechnologen, die aus Enzymen eben Produkte produzieren wollen entweder daraus dass sie Mikroorganismen dazu benutzen oder Enzyme direkt selbst produzieren und also ohne Mikroorganismen in einem Reaktor umsetzten.	
Interview 12		Jeder davon hat natürlich einen anderen Background und hat verschiedene Ansprüche an die Datenbank, sodass wir auf der einen Seite dafür sorgen müssen, dass jemand sehr schnell Informationen über die Enzyme bekommt. Andererseits aber auch die vielen Detailinformationen relatvi kurzfristig bekommt.	
Interview 12	10:36	Das heißt Datenbestand, sehr effiziente Query Engine, die auf verschiedene Nutzeransprüche Rücksicht nimmt und eine Visualisierung der Daten, die teilweise eben als Zahlen oder Buchsstaben darsgestellt werden kann - in vielen Fällen aber auch als Grafiken um den Benutzer eben seine Ansprüche zu erfüllen. Das heißt aus meiner Sicht eben die drei Komponenten: Datenbestand, Query Enginge und Visualisierung der Daten und shcneler Datenzugriff.	
Interview 12	11:36		Welche Vision wird denn mit der Plattform verfolgt?
Interview 12		Möglichst alle diese Ansprüche der Benutzer zu erfüllen und dafpr zu sorgen, dass der wissenschaftliche Fortschritt - Enzyme steht ja da im Zentrum - praktisch alles, was Oeganismen machen, passiert durch Enzyme. Den wissenschaftlichen Fortschritt zu beschleunigen auch. Wir definieren unseren Erfolg eigentlich aus den Nutzerzahlen.	
Interview 12		Das heißt, wenn da eben so 50.000-10.000 Benutzer extern pro Monat auf unsere Datenbank zugreifen, dann ist das natürlich ein vielfaches von jeder Bibliothek Und wir gucken genau hin, welche wissenschaftlichen Weiterentwicklungen, und definieren dann auch mehr Ansprüchevon unseren Nutzern, die wir dann eben auch in unseren, unserer Interface und in unserem Datenbestand aufsplitten.	
Interview 12	11:41	Das heißt, Mitarbeiterin oder ich eben als Leiter gehen zu Konferenzen, die wissenscahftlichen Fortschritt bei auf den Enzymen eben beschreiben udn gucken dann nach, was müssen wir usätzlich in (platform) aufnehmen. Also die Vision ist immer Up to date und gut zu sein oder hot zu sein.	
Interview 12	13:11		Wie ist denn der orgnaisatorische Aufbau hinter der Plattform?
Interview 12	13:17	Meinen Sie den technischen oder Mitarneiter?	
Interview 12			Mitarbeiter reher?
Interview 12		JA, die Gruppe ist relativ klein. Es sind vier Mitarbeiterinnen, die intern arbeiten. Zwei davon kümern sich um den manuellen Core, das heißt die Datenextraktion und der Identifizierung von spannenden neuen Publikationen. Eine dritte Mitarbeiterin entwickelt neue Software, die praktisch permanent notwendig ist.	

Interview 12		Entwicklungen müssen immer eben wie gesagt an / wenn es fortschritte gibt muss das eingebaut werden. Und die vierte mitarbeiterin ist dafür zuständig, dass die Hardware hocheffizient läuft, Wir haben ja normalerweise, jeder Nutzer von den 50.000 die Pro Monat da sind, sind es bei uns normalerweise 5-10 Minuten bei uns auf der Datenbank, sodass wir teilweise 100Nutzern gleichzeitig haben, die dann auch effizient mit kurzen Antwortzeiten ihre Ergebnisse erwarten.	
Interview 12	13:25	Das heißt, zwei aus dem (platform) Core, eine Mitarbeiterin für Software Entwicklung und eine Mitarbeiterin, die sich um Hardware und auch das Gesamtsystem, die Query Enginge kümmern und den halbjähren Update eben auch realisiert.	
Interview 12	15:00		Welche Kompetenzen besitzen denn die Mitarbeiter?
Interview 12		Jeder ist auf seinem Gebiet weitgehend unabhängig. Das heißt, die Mitarbeiterinnen sind bei uns auch relativ lange da. Also. Die letzte, die wir von den - achso, was ich jetzt noch vergessen habe. Dann haben wir natürlich noch die externen Mitarbeiter, die die Datenauswertung geschickt werden. Die anderen sind längerfristig da. Also die, meine Frau hat eigentlich schon von Anfang mitgearbeitet, die scheidet jetzt auch dieses Jahr aus, Alterbedingt.	
Interview 12		Eine Mitarbeiterin ist seit (year), eine seit (year) und eine seit (year). Und jetzt kommt eben eine neue da, die dann meine Frau ersetzen wird, sodass jeder auf seinem Gebiet Experte ist und eigentlich völlig selbstständig arbeitet. Wir treffen uns dann in regelmäßigen Abständen und diskutieren was, wo sind irgendwie Probleme aufgetaucht, wo haben wir Fehler gefangen, wo sind Neuentwicklungen, die wir in den nächsten Monaten realisieren müssen.	
Interview 12	15:03	Und zu dem Zeitpunkt werden dann die Aufgaben verteilt und danach arbeitet dann jeder von den Vieren völlig selbststädig an jedem Teil. Auf der anderen Seite müssen wir natürlich auch sorgen, dass das so ein bisschen redundanz da ist. Das heißt, wenn dann eben jemand im urlaub ist oder auch mal krank wird, dann muss jemand Zweites da sein, der sich zumindest soweit auf dem Gebiet auskennt, dass das halt die notwendigsten Dinge funktionieren. Was natprlich bei so einer kleinen Gurppe relativ viel schwieriger ist, wenn man, so iwe dass (insitut) so 120 Leute hat.	
Interview 12	17:00		Sie haben gerade erzählt, dass Sie zusammen sitzen, Sachen diskutieren und auch besprechen. Wie werden dann die Entscheidungen getroffen?
Interview 12		Eigentlich, ja. Jemand von uns fünfen macht einen Vorschlag, das sollten wir neu machen. Dann wird darüber diskutiert, wie viel Aufwand steckt hinter dem neuen Projekt hinter, wie wichtig ist das. Und normalerweise entscheiden wir dann nicht mit einer Abstimmung aber mit, ja, in Übereinstimmung.	

Interview 12	17:13	Also wenn jemand sagt: OK, wenn ich jetzt eine neue Idee habe, da habe ich was gesehen, das sollten wir machen und die zuständige Mitarbeiterin sagt dann, dass würde aber drei Monate Implementierungszeit brauchen und ich habe im Moment noch andere Prioritäten, dann verstehen wir das als Projekt, das - das läuft bei uns dann, es gibt Projekt, die wichtig sind und aktuell sind und es gibt Projekte, die Nice to have sind, wo es schön wäre wenn wir die hätten, die wir dann aberangehen, wenn wir Zeit haben.	
Interview 12	18:20		Als Sie die Datenbank aufgebaut haben oder auch jetzt gerade, haben Sie sie sich überlegt, wie Sie die Plattform strategisch Positionieren wollen?
Interview 12		Es gab, die strategische Positionierung ergab sich automatisch aus dem Inhalt. Enzyme haben eine bestimmte Funktion in der Biochemie oder in den Biowissenschaften, in den Lebenswissenschaften insgesamt. Und der Ansatz war vom Vornherein das so zu machen, dass man möglichst alle Nutzergruppen befriedigen können.	
Interview 12		Das wir irgendwann mal Core Data Resource werden würden, das heißt eine der unverzichtbaren Datenbanken in (region), war natürlich von vornherein nicht absehbar. Es war ja auch immer sehr schwierig die Datenbank zu finanzierne. Wir haben uns so von zwei bis drei Jahresdrittmittelprojekten zum nächsten gehangelt.	
Interview 12	18:37	Und zwischendurch gab es Zeiten, da gab es keine, überhaupt gar keine Finanzierung. Da habe ich das aus Autorenhonoraren, die ich aus einem Buch bekommen habe, eigenfinanziert - das war aber nur eine kurze Zeit. Ja, die strategische Ausrichtung sollte immer so sein, möglichst hohe Nutzerzahlen, möglichst viele Leute zu erreichen und vielen Leuten, die /	
Interview 12	20:00	Primärliteratur zu ersparen.	
Interview 12	20:08		Wie kontrollieren Sie denn die gesteckten Ziele für die Plattform
Interview 12	20:13	Ja, über Nutzerzahlen.	
Interview 12			Wie erheben Sie /
Interview 12		Also gut, natürlich haben wir einerseits: wie viele Publikationen wollen wir pro Jahr auswerten. Das wird dann also zweimal im Jahr angeguckt. Und wenn ich dann sehe, da gibt es irgendwo weniger als wir wollten, dann setze ich mich mit den beiden zuständigen Mitarbeitern zusammen und dann besprechen woran das liegt.	
Interview 12		Und ansonsten gucken wir den Zuwachs an, gucken die Nutzerzahlen an, gucken an wie viel Feedback bekommen wir über Fehler in der Datenbank und haben da eben bestimmte Standards, die wir halten oder verbessern. Man kann ja / jeder (unv.) Nutzer wenn ihm irgendetwas auffällt, was ein Problem oder falsch sein sollte, dann schickt er uns ein Ticket - wir haben ein Ticketsystem - und das muss dann eben möglichst schnell bearbeitet und beseitigt werden, wenn es ein Problem gibt.	

Interview 12	21:26		Sie haben gerade auch schon von der Finanzierung gesprochen und dass es lange über Drittmittelprojekten beziehungsweise Eigenfinanzierung ging. Wie erfolgt denn momentan die Finanzierung
Interview 12		Also im Moment finanziert / Das Ende (year/number) funktioniert die Finanzierung über das / Seit (year/number) über das deutsche Netzwerk Bioinformatik Infrastruktur, wo eben (--) ein Strategiekonzept des BMBF - also das Bundesminististerium für Bildung und Forschung - entwickelt worden ist, die in Deutschland den Forschern und auch den Firmen schnelleren Zugriff sowohl auf Daten als auch auf eigene Softwareentwicklungen geben wollten.	
Interview 12		Und dadurch, dass wir gerade (unv.) geworden sind und das heißt als unverzichtbar deklariert wurden, soll ab (year/number) die Datenbank dann tatsächlich perpetuiert werden, das heißt (unv.) werden, sodass nicht alle paar Jahre eine neue Finanzierungsquelle gefunden werden muss.	
Interview 12	21:39	Wir sind ein paar Jahre zwischen (year/number) und (year/number) durch die EU finanziert worden, mal durch das Land, mal / häufig durch vom BMBF, also unterschiedliche Finanzierungsquellen. Die, die gerade auf dem Gebiet Gelder zur Verfügung gestellt	
Interview 12	23:05		Welche Schwierigkeiten und Herausforderungen gibt es denn beim Management der Plattform?
Interview 12		[SEUFZ] Naja, es sind alles Profis. Klar. Also die Schwierigkeiten und Herausforderungen besteht darin, dass das ganze System äußerst komplex ist und in dem Moment, wo irgendeine Mitarbeiterin kündigt und weggeht, da erfordert das eine längere Einarbeitungszeit, bevor es dann so wie normal weitergehen kann.	
Interview 12	23:10	Und es werden natürlich auch / jeder, der aus dem Internet bekannter ist, ist einer Menge von Hackerangriffen ausgesetzt und wir müssen uns dagegen wehren und versuchen, da immer einen Schritt besser als der Hacker zu sein. Also toitoitoi, bisher ist in den dreißig Jahren also / Tatsächlich sind wir nie wirklich gehackt worden.	
Interview 12	24:10		Somit haben wir den ganzen Block mit dem Organisatorischen hinter der Plattform schon einmal abgehakt - das ist gut. Komme ich zum Thema Plattformgovernance und -nutzung. Sie haben das natürlich schon ein bisschen angesprochen, aber vielleicht kommen gleich noch einmal andere Aspekte, deswegen frage ich das einfach noch einmal: Welche Funktionalitäten und Möglichkeiten bietet die Plattform?

Interview 12		Naja, die Enzymdaten sind nicht wie zum Beispiel Sequenz- oder Textdaten, einfach bestehen nicht nur aus Zahlen und Buchstaben, sondern bestehen auch aus Strukturen. Also zum Beispiel so eine Molekülstruktur von einer chemischen Verbindung, die hat eben eine gewisse Struktur. Und in Texten zu suchen, da möglichst effizient Dinge zu finden wie zum Beispiel in einer Sequenzdatenbank, wo man nach dem Organismus fragt und vielleicht nach einer bestimmten Sequenz, das ist (unv.) trivial, aber eben hocheffizient im (-) in Strukturformeln / Strukturen sind einerseits die Protein-3D-Strukturen, sind die chemischen Strukturformeln und sind die metabolischen Pfade.	
Interview 12		Das ist etwas, wo wir eigentlich ziemlich stolz drauf sind, dass der Nutzer hier bei uns nicht nur nach Substanznamen suchen kann, sondern auch nach Strukturen und eben auch viele Dinge verbinden kann. Er kann eben auf der einen / Er kann sagen: "okay, mich interessiert ein Enzym, was zum Beispiel in einer Membran gebunden ist, was hitzestabil bei / bis 60 Grad ist, was ein ganz bestimmtes Substrat umsetzt, und was nicht durch xyz imidiert (unv?) oder zerstört werden kann".	
Interview 12	24:35	Er kann also diese / Diese Komplexität der Abfragen ist eben sehr wichtig und auf der anderen Seite die Darstellung der Ergebnisse, die dem Benutzer mit einem Blick eigentlich zeigt: wo geht es bei diesem Enzym über so eine Wordmap, so eine Oracle von Begriffen, die mit diesem Enzym verbunden sind, und auf der anderen Seite ihm eben die 5000 Einzeldaten sehr effizient zur Verfügung stellt und ihm auch die erlaubt diese Datenbanken herunterzuladen.	
Interview 12	26:42		Wie beschreiben Sie die Nutzergruppe und / oder beteiligte Institute der Plattform?
Interview 12		Ja, die Nutzergruppen habe ich schon intensiv im vorherigen Block beschrieben, irgendwie die Mediziner, (unv.), Mikrobiologen, Zoologen, Botaniker, also die gesamten Lebenswissenschaftler und kooperierende Partner, dabei sind natürlich die zum Beispiel das (region) Bioinformatics Institute, mit dem wir sehr lange gemeinsame Grants(?) und Drittmittel hatten.	
Interview 12	26:50	Und natürlich letzten Endes jeder Nutzer, dem irgendetwas auffällt und der uns Vorschläge macht. Jetzt arbeiten wir zur Zeit im (unv.) den anderen Gruppen dieses deutschen für Bioinformatik-Infrastruktur zusammen, ansonsten wie gesagt (platform), BI, hier in (town) die deutschen (unv.) für Mikroorganismen, weil sehr viele der Enzyme auch in Mikroorganismen vorkommen und, was die Protein-3D-Strukturen, Raumstrukturen, angeht, mit einer Gruppe Bioinformatik in Hamburg.	
Interview 12	27:57		Welchen Regeln unterliegen denn die Nutzer?
Interview 12	28:01	Keiner. Können machen, was sie wollen.	
Interview 12	28:05		Vorhin haben Sie gesagt, dass Daten hochgeladen werden können, aber das nicht so häufig genutzt wird.
Interview 12		Richtig	

Interview 12			Wenn Daten hochgeladen werden: Welche Anforderungen stellen Sie denn an diese?
Interview 12		Naja klar, wir geben eine bestimmte / Also einerseits müssen die Daten natürlich richtig sein und sie müssen auch formal richtig sein, das heißt wir geben eine bestimmte Datenstruktur vor, in der der Nutzer die Daten hochladen kann, und das wird sofort beim Hochladen geprüft, und wenn die Struktur nicht stimmt, dann kann er das gar nicht hochladen. Und danach läuft dann eben ein Check über die wissenschaftliche Plausibilität der Daten.	
Interview 12		Auf jeden Fall müssen die Daten publiziert sein, also niemand kann irgendwie aus seinem privaten Labor Daten hochladen, die nicht publiziert und über die Publikation schon einmal geprüft worden sind. Was unsere eigenen Mitarbeiter angeht / externen Mitarbeiter angeht, die von uns beauftragt werden, eben bestimmte Publikationen auszuwerten:	
Interview 12	28:19	auch bei denen ist es so - die werden ja pro ausgewerteter Literaturstelle bezahlt - und wenn sie mit der Auswertung fertig sind, können sie die Daten hochladen, können sie aber nur dann hochladen, wenn sie formal und nach einem ersten Check ok sind. Und das wird schon beim Hochladen getestet: ist das, was sie gemacht haben, vernünftig? Sonst können sie das gar nicht hochladen.	
Interview 12	29:45		Und wie erfolgt dann die Qualitätskontrolle?
Interview 12		Ja - Formal direkt also (-) vorwiegend erst einmal über Computerprogramme, die dann checken, zum Beispiel ist hier ein pH-Wert zwischen 0 und 14 und ist ein KM-Wert in dem Bereich, wo er normalerweise sein sollte, und danach wird jeder Datensatz, der ausgewertet wurde von der Publikation, von zwei internen Mitarbeiterinnen gelesen; einer, die biologische Vorkenntnisse hat, einer anderen die chemische Vorkenntnisse hat, um da auch Fehler zu finden.	
Interview 12	29:47	Und danach wird / nachdem sie in unseren Server eingelesen worden sind, gibt es Computerprogramme, die jede einzelne Webseite / die Fragen stellen / also, die Fragen an die Website stellen, genau wie es Nutzer stellen würde, und die die Antwort kontrollieren - Also sehr umfangreiche Qualitätskontrolle.	
Interview 12	30:48:00		Und wie wird dann das Vertrauen in die Daten sichergestellt?
Interview 12		Naja, durch unsere Kontrolle - Also die Nutzer müssen uns vertrauen, dass wir eben vernünftig das Ganze kontrolliert haben. (--stottert--) Sagen wir so, das geht ja immer so durch die Medien, dass es pro Jahr vielleicht einige Publikationen gibt, die gefaked wurden und wenn das passiert, dann werden diese Publikationen normalerweise von den wissenschaftlichen Journalen aus ihrem Bestand genommen und genauso wenn bei uns mal eine Publikation ausgewertet wurde, wo der Nutzer die Daten gefaked hat, dann werden auch diese Daten bei uns rausgenommen.	

Interview 12	30:52:00	Das heißt, auch das kontrollieren wir: gibt's da irgendwelche gefakten Publikationen bei uns, die bei den Journalen durchgerutscht sind und dann auch bei uns mit aufgenommen wurden? Und die werden dann auch bei uns sofort gelöscht.	
Interview 12	31:55:00		Wie stellen Sie denn sicher, dass die Nutzer der Plattform wissenschaftlich korrekt mit der Datenverwertung umgehen?
Interview 12		Stellen wir nicht fest - wir haben ja keine persönlichen Daten. Das heißt, letzten Endes ist so eine öffentliche Datenbank - und das betrifft die großen Genomdatenbanken, das betrifft die Proteindatenbanken, das betrifft auch uns - letzten Endes kann der Nutzer natürlich im Prinzip mit den Daten alles machen, was er will.	
Interview 12	32:03:00	Nun sind Enzyme normalerweise bis heute nicht irgendwo geeignet, um - im Gegensatz zu Mikroorganismen - um daraus (unv.) zu machen, aber ansonsten hat der Nutzer die Eigenverantwortung, mit den Daten so umzugehen, dass er keine Gesetze verletzt, aber (-) dass (-) jeder (-), der seine Daten so öffentlich zur Verfügung stellt, hat keine Kontrolle mehr.	
Interview 12	33:07:00		Welche Schwierigkeiten treten denn beim Datenaustausch über die Plattform auf?
Interview 12	33:12:00	Keine, die ich kenne.	
Interview 12	33:18:00		Gut. Wie bewegen Sie denn Forscher und Organisationen, die Plattform aktiv zu nutzen?
Interview 12		Ja, in / natürlich haben wir / Inzwischen sind wir so bekannt, dass jeder, der etwas über Enzyme wissen will, erst einmal zu uns kommt, aber es gibt natürlich auch viele Leute - gerade wenn jemand neu auf dem Enzymgebiet ist, der das vielleicht noch nicht kennt, das auch nicht über Hörensagen kennt - da gehen wir auf Tagungen und nehmen dann häufig auch einen Laptop mit und zeigen dort, was die Datenbank kann, da wo sich Wissenschaftler treffen.	
Interview 12		Wir wissen auch / Also bei uns zum Beispiel hier / In den Vorlesungen wird (platform) vorgestellt, und wir sehen auch immer dann, wenn das Semester beginnt - sowohl hier in Deutschland, als auch wenn das akademische Jahr dann in den USA zum Beispiel beginnt - , dass plötzlich die Benutzerzahlen schnell ansteigen, wenn (platform) in den Vorlesungen vorkommt.	
Interview 12	33:25:00	Wir machen auch Tutorials, das heißt wir lancieren Kurse, die in die Nutzung von (platform) einführen und bieten die dann eben mehrmals im Jahr an. Eine Zeit lang sind wir herumgereist bis ins Baltikum und haben eben dann zum Beispiel gemeinsam mit (platform) eben die Datenbanken vorgestellt.	
Interview 12	34:59:00		Welche weiteren Möglichkeiten können noch in Betracht gezogen werden, um Nutzer zu generieren?
Interview 12	35:08:00	Wenn ich das wüsste, hätten wir das schon gemacht.	

Interview 12	35:13:00		Ok, gut. Das Besondere an der Plattform ist ja jetzt auch, dass sie in die wissenschaftliche Kultur eingegliedert ist. Wie, denken Sie, beeinflusst die wissenschaftliche Kultur die Nutzung der Plattform?
Interview 12		Ja klar, die Datenbank und auch die wissenschaftliche Kultur hat sich ja in den letzten Jahrzehnten sehr stark in Richtung Digitalisierung entwickelt. Wo der Wissenschaftler noch vor / Also ich habe noch Ende der 90er Jahre, Anfang (year/number) mit Wissenschaftlern gesprochen, die gesagt haben: "Also ich gehe lieber in die Bibliothek und lese Paper anstatt diese komischen Datenbanken zu benutzen", aber der Trend ist klar: Jeder versucht heute, seine Informationen, die er für seine Forschung braucht, so schnell wie möglich zu bekommen und das geht eben nicht ohne die Datenbanken.	
Interview 12	35:28:00	Was die nächste Herausforderungen seit Jahren ist, ist natürlich, die Daten, die an den verschiedenen Stellen auf dem Internet liegen, möglichst so zu vernetzen, dass der Benutzer nicht nur das bekommt, was jetzt in (platform) oder in (platform) so steckt, sondern vielleicht den Hinweis bekommt: "Hier, da gibt es weitere Daten, die vielleicht auch interessant sein könnten", aber das ist so diese Hypervernetzung, da sind wir noch nicht sehr / ist insgesamt die Szene noch nicht so weit, unabhängig vom Fach.	
Interview 12	37:05:00		Welche Entwicklung sehen Sie denn für die Plattform in den nächsten zehn Jahren?
Interview 12		Ja, das ist eine Frage, die wird sich immer stellen. Also tatsächlich denke ich, geht die Entwicklung in den Biowissenschaften sehr stark in Richtung Modellierung. Das heißt, in bestimmten Teilbereichen haben wir schon die virtuelle Zelle, wo man dann die Frage stellen kann: "Wie kann ich zum Beispiel einen Mikroorganismus pathogenes (unv.) umbringen(?)? oder wenn er biotechnologisch interessant ist, am Leben erhalten?	
Interview 12		Das heißt es / Heute ist es so, der Nutzer guckt sich die Daten eines oder mehrerer Enzyme an, schreibt sich auf und versucht sich zu überlegen, welche Auswirkungen auf den Organismus hätte das zum Beispiel, wenn ein Antibiotikum ihm gebe. Das ist heute weitgehend noch auf einen Organismus beschränkt und man weiß nicht: wie lange braucht der Organismus dazu, um resistent zu werden?	
Interview 12		Wie funktioniert das wirklich in den großen Verbänden? Wenn der Organismus meinetwegen Alkohol(unv., ?) produzieren soll oder Medikamente produzieren soll. Und das ganze wird in Zukunft mit Hilfe der Daten, die wir und andere zur Verfügung stellen, in Modelle einfließen, sodass man wahrscheinlich in (unv., wsl 10) Jahren sagen kann: "ok, das sind die Bedingungen, die ich dem Organismus geben könnte, wie viel Alkohol kann er produzieren?	

Interview 12		Wie lange überlebt der Organismus in der Umgebung? Wann gibt es zu wenig Sauerstoff? Wann muss die Temperatur vielleicht um fünf Grad erhöht werden?" Also, dass er nicht mehr so viele Daten abfragt, sondern dann ganze Zeitabläufe abfragt und seine / das was er sich versucht im Kopf vorzustellen, in Modelle steckt.	
Interview 12	37:11:00	So die virtuelle Zelle, der virtuelle Mensch, der virtuelle Darm, wie / welches Antibiotikum muss ich geben, um nur einen Organismus abzutöten und welchen Einfluss hat das auf die anderen? Also die starke Entwicklung in Richtung Simulation, virtuelle Zelle, Modelle.	
Interview 12	40:05:00		Und wovon hängt dann der Erfolg der Plattform ab?
Interview 12	40:09:00	Ja, ob sie immer wissenschaftlich am Puls der Zeit bleibt. Das ist natürlich auch etwas, was mich auch bewegt, denn wenn ich jetzt die Leitung abgebe, wird mein Nachfolger da genauso eben sein Ohr überall haben und sehen: "Ok, hier müssen wir jetzt eine Software einbauen, die jetzt nicht nur Daten aufbereitet und toll zur Verfügung stellt, sondern auch gleich ein Modell daraus kreiert und dem Nutzer sagt: 'Ok, so sieht es heute aus, aber drücke mal auf den Knopf, dann erfährst du, wie es bei dem Organismus in zwei Wochen aussieht'"	
Interview 12	40:52:00		Gibt es sonst noch etwas, was Sie mir zum Thema Erfolgsfaktoren von (platform) erzählen möchten?
Interview 12	40:59:00	Ich glaube nicht. Also ich glaube, wir haben über das meiste gesprochen.	
Interview 12	41:02:00		Sehr gut. Dann habe ich noch ein paar /
Interview 12		Doch, entschuldigung. Eines muss ich noch erwähnen. Man braucht natürlich sehr gute und hoch engagierte MitarbeiterInnen. Ich hatte ja zu Anfang gesagt, jeder arbeitet sehr weit selbstständig. Wenn das nicht klappt und auch das Teamwork in der Gruppe nicht klappt, dann ist bei einer so kleinen Gruppe so ein Projekt sehr schnell tot.	
Interview 12	41:07:00	Und da ist natürlich auf der einen Seite die Mitarbeiter / denen muss es Spaß machen, es muss aber auch jemand da sein, der das Projekt leitet und die Mitarbeiterinnen und Mitarbeiter dann auch motivieren kann. Gut, dass war das, was mir noch auf der Seele lag.	
Interview 13	Time	Interviewee 13	Interviewer
Interview 13	5:38		And what is about the future funding of the platform?
Interview 13		So we didn't. Okay, so. (organisation) is a (region) infrastructure project. What does that mean is you have a number of countries that commit to contribte to that. So one part is with money. So each country provides money directly to the project. And in the case of (organisation), it means that we have what we call there's a service provider in (country) who scored at the opera project and in Spain, you have the IT infrastructure.	

Interview 13	5:44	And that's what that money that Belgium is paid for you life which is going to and next to that you have incurred contributions, and that's actually funding that Belgium is providing or another country for for their country, IT projects within that country in doing that, so in Belgium, you have that it is Belgium is a complicated country. So you have that at the regional level. So in Flanders you have Flis (?) which is the Flemish marine Institute	
Interview 13		So they work a lot. Also, what we did in the past with (unv)is linked a lot with activities with List. So we've been collaborating with them. So part of that is working on taxonomic backbones, tracking data. In the region, you have people that work more with satellite data, and trying to, for instance, detect forest fires in (region) using satellite data, or vegetation types and those kind of things. And so at the federal level, so the national level, we have two projects.	
Interview 13		So one is our Antarctic projects. And the other one is Baku, which is trying to do molecular identification of any kind of specimen. So that might be samples that they find in the airport if they want to identify animals and the goal there is to mostly monitor site the species society species are species that are protected and are not allowed to be traded. it. And so they provide a service that people can check if they have a DNA sample that matches with a protected species.	
Interview 13		So that's (organisation). So we've now had a funding round for four years. And the idea is now that there will be sort of new call that we have to subscribe to, and that will be funding for another four years. So but we have to submit to that. And then that continues. There is a bit of a caveat in that is that Belgium is still waiting to have a government.	
Interview 13		So what we know as we're in an intermediary phase, so we extended our project from the last four years with one year because there's no government, they can't put out the call for for new funding, but we get continuation of the funding at this stage. So it's, so it's, but all of the money comes from project money.	
Interview 13	7:01	So in in the staffing, it's all project, there's not a fixed institutional funding for that. But most of the funding is sort of semi committed for for a longer term. So it's not really short short term. It's sort of mid mid to long term that we are funding, and usually in the past with yearly evaluations to see if we're meeting objectives and if the funding would continue.	
Interview 13	9:27		And what is the core offering of the platform now?
Interview 13		So what we're working with now is, and it's a bit it's a bit of a hiatus if what we have with life, which is so we don't have full access to the central IT services yet. So we still focus a lot on the access to data, educating people about data management, how they should do that. And like I said, developing tools to make data more discoverable, but also making it more interlinked. So one of the updates we're working on now is for the genomic data.	

Interview 13	So you have genomic sequences, and you have existing platforms where people can publish that data. So you have (platform) is one of the most well known one. And if you can put sequences, but for instance, the sequences, often when you submit them, you have to do that for a paper. But often the environmental information is long not linked to the sequences. And so the context of that sample is often lost.	
Interview 13	And so what we try to do is, in one hand, trying to ingest the information that exists around the world, but also provide a platform where people can publish the data from the genetic information and the environmental information together. So the idea there is that that every mental data sort of getting lost otherwise. But also with the biodiversity portal, it's about trying to link different types of information.	
Interview 13	So another example and that links with other things in (organisation). So what I said earlier with the register 130 arctic marine species. That's part of its complicated. That's part of words, which is a world register of marine species. And within (organisation), they're sort of doing a similar exercise by expanding the scope.	
Interview 13	And we're working within 30 community too. So the Taxonomic Information is basically what's the name of a species we described it. And what we're trying now is to add more ecological information there. What's the size of an animal? How does it reproduce? And the idea is that if we can link those different data portals is That queries that we can do become more interesting.	
Interview 13	A simple example for instance, is that, in the Merida environment, an important distinction is between the logic and benteke simple logic is anything that lives in the water column. And then take is what lifts on the sea floor.	
Interview 13	Fill some groups you can just based on the taxonomy of a species you can say, Okay, this is gonna be it's gonna be that, but in a lot of cases, these can be either political benteke and you don't know that until somebody says you that it's illogical, benteke app. So for real, you know, a will, will be the logic because it lives in the water column.	
Interview 13	For a crap the most craps, you know that it's bentik (anm: bentisch auf deutsch) but the crustacean you have krill which is pelagic, or you're having the same crap which is bentik. So the idea That's if you can lick that information. What you can do in terms of queries is much more interesting. Because if you have that information of the ecology of the species, you can make a query that says, Give me the distribution of all the pelagic species.	
Interview 13	If this is changing in the pelagic environment, which we can get from from different types of model, this is what we expect to be changing for that group. So it's really I think the key thing, what we try to do is make data. But it's going back to the fair principle. So we've always worked on on making data findable and accessible.	

Interview 13		So that's what we've done since since we started 15 years ago. But I think what we're now trying to focus on is the interoperable and reusable and that's following standards, but also leave it to other data context, so that people can can use it in a better way. And for now, we've been focusing a bit Things like our tools.	
Interview 13	9:36	So R is a programming language. And we try to use that quite quite extensively because that's what biologists or ecologists tend to use the most often. And it's a very transportable way of working, you can set up a server that built during that will that will run that software or people can run it on their computer. So it creates a lot of flexibility.	
Interview 13	14:33		So my work now is about success and success factors. How do you define success or a successful platform?
Interview 13	14:48	I think that's a difficult one. I think I think the easy one to do to define a successful platform is just the number of hits and the number of downloads but actually With the way that we are set up,	
Interview 13		like I said, we do educational data publication and we help people publish data. But we're an Antarctic node, so we're domestic. So countries are researchers, the countries that we work with my publish data to their own national portal. So we provide data to (platform) and cheapish mentioned, you know what you do this.	
Interview 13		So that that's destinations publishing the data. So actually, a lot of the work that we do in training researchers to use the right formatting for that data might not be visible because they're not publishing data through our portal, but through another resource. So I think it's a bit tricky to to quantify success in that way. I think for me, it's in a scientific level. It's having researchers use the tools and You provide and cited tools that you provide.	
Interview 13		But we also want to be relevant at a policy level, or that's what we're asked it to be in the context of our project. And I think again, that's that's that's sometimes hard to quantify. Now, I know within the EU (organisation) scheme. The goal there is that at some point, they want to centralize most of the services.	
Interview 13		And the idea there is that the way that no city that you would log into a platform that uses services and that based on the usage to that platform that you can't quantify that, but like I said, I think it's what's happening is going to be more let's do more dispersed than that.	
Interview 13	15:03	So I think it's always a bit tricky to to quantify the success for us we had so many from (year/number) We published a very big emphasis on the biogeography of Antarctic species. And I think in a way with the commitment from this community that we got for that. And having that established, I think that was a key measure for for the success that we had at the time.	
Interview 13	17:19		And what vision Are you pursuing with the platform?

Interview 13	17:30	Well, the the the catchphrase that we always use is free and open access to Antarctic biodiversity data. So for us also. So we work in an Antarctic complex, and there's actually an Antarctic Treaty there. And that actually states that research from the Antarctic should be the free available and so it's really trying to push that. So that's a phrase that we've already had before. The fair principles. And I think for us, it's really trying to have and antarctic biological and ecological data available according to do the fair principles.	
Interview 13	18:13		Good. So now I want to continue with a more organizational view to the platform. So my first question is, what is the organizational structure behind the platform?
Interview 13		So that's what I said in my email. I actually want to change it because I'm not happy with it. So currently, organizationally is so we get our funding from Belgium science policy. And that's most of the funding that we get. And so that's the funding then goes to the Natural History Museum in Brussels, Belgium, natural sciences.	
Interview 13	18:30	So I managed project. And then I have two staff additionally, who work on development and one more. So one is a database developer and another one is more than our programming. And then we currently have a technical advisory committee which is a requirement for having the funding from from the Belgian science policy and we have four people on that one.	
Interview 13		And then Currently, the way that we operate is that we're active in a number of scar activities. So we did scar. You have a number of working groups Yes, you have the the life science in the working life sciences group. And then you have different working groups below that our active in in the set up these working groups, and basically by directions with the researchers. We sort of get an idea of what they want.	
Interview 13		And then we propose activities which go to our, our technical advisory board, and then we move in in that direction, and we do the planning at an annual basis. And what I would like to see rather is that we would set up something like a scientific steering committee that would be broaden so that we have eight or 10 people that are all researchers involved in the skaar community.	
Interview 13		And that would they would actually give us a more formal advice on what they think is useful? Because I think the risk of it what we are working with now is that we might focus too much on the IDs of these are these people, because we hear them more often or because more hearable. and I think by setting up a steering committee, the idea with that is that we get more engagement from the community and the people, the researchers can put more of their IDs for it.	

Interview 13		So it's, yeah. And so then with the registrant Arctic marine species, there is a bit the same, we manage the community. And it's Yes, basically the taxonomies that add the data. And occasionally we organize a workshop to advance on on certain topics, but I think in the structure. So now, also due to the financing, it's a bit focused on on Belgium, but I think we need to bring it down a bit more in order to allow more formal engagement because I think we'll end up with half of these people will be people that we're already engaging with, quite regularly, but the idea is that if we said have a scientific steering committee, they can put that on the sci fi.	
Interview 13		And they can say this is something that before many contribute to so that they can recognize the effort. And ideally, then we also have, and I think that's important is that, for instance, after four years, we can say, okay, you served your term. And now it's time for a younger scientists to take over.	
Interview 13	19:32	And I think that rollover is something that we are a bit limited to. So we have people that have been with us for the last 15 years that are still involved. But if you look at what data management and data scientists, it's not necessarily what it was 15 years ago. So the idea is there that we can get some some some new ideas in as well.	
Interview 13	22:42		So, what competences do that people have now working for the platform?
Interview 13	22:49	the people that work for the platform, so they all have a background in biology, somehow, so you Like I said, I did a PhD on biology. I sort of learned data management by by working on the project. You mean, who's our database developer? She has worked. She did it.	
Interview 13		He did the mostest Yes, it's um, it's a secondary master that she did on bioinformatics. So she worked from from / with larger data and in our studies, and Maxine was doing a lot of the art programming. He did a PhD on Antarctic microbes. So looking also at the genetics and and these things.	
Interview 13		So I think everybody that's working for the platform is a biologist, but with a strong a component of working with larger data sets and again, trying to integrate those statistics. different sources. I think that the data management itself, so we don't have a formal it profile.	
Interview 13	23:18	But that's sort of, again, if I look at all the data divisions in our institute, you quite often see that actually biologists get turned into data scientists. And that can mean imagine that a lot of people with a scientific degree go through a similar process. Because as researcher, you need to work with data and then it sort of follows from that.	
Interview 13	24:37		Um, when you have to decide something regarding the platform, how was the decision process behind it?
Interview 13		So the idea there is that, like I said, so on an annual basis will be their technical steering committee. And so during the We get certain ideas that we develop either by communicating or with with smaller workshops. And then for the next year, we sort of present the outlines of what we want to do.	

Interview 13		And we get that with our technical steering committee, and then they give sort of the go ahead for the year what we plan to do. So last year, as an example, for that we had a workshop the on the taxonomy, and there it is discussed these traits. And that's where we set up an outline of these are the traits for this year that we want to focus on.	
Interview 13	24:51	And this is the information that we want to try to work actively on. To include with the data publication, it's it's a bit, it's an ongoing effort. And it might be that we get a certain request to help publishing data sets, or that we need to provide advice but that's sort of a non gap (?) basis. So we keep it in the best Remind that we might need to put time and effort in those things. But normally it's sort of trying to do what's planned in usually in December and then we try to follow that truth for the rest of the year.	
Interview 13	26:13		Are you working with consensus regarding the decisions?
Interview 13	26:24	Not really.	
Interview 13	26:28		So how does it work?
Interview 13		Those are so so so now, what we do what we think this is the right way to go. So I think it's so it's I think with a scientific steering committee, you would go more towards having something where you work with a consensus that's, that's what I said. So if people come to us and say we have these issues, and this needs to be resolved, and if they convince this, it's an issue, then we'll do it.	
Interview 13	26:30	And then it's a matter about the time and the funding that we can afford or if there's a case that's another thing. If there is a context where we can get additional funding to do additional work, that sort of also will will will get more traction. So an example actually for the last couple of years is that we've been working.	
Interview 13		Try not to do to go too much into different acronyms. So we had a group who works with tracking data. So they put a small logger on the pinguin or receive or a bird. And they wanted to do an analysis at the Sacramento Arctic scale, combining data from different institutes from different researchers.	
Interview 13		And so they said, We need help in managing this and we said that's fine. But so what they actually did is they because I just like to question this through the proposal of That they submitted in France on having a postdoc that would work on that as well adding money to organize a number of workshops.	
Interview 13		And so that funding but true, and because we there was funding for us to participate in the workshops, and there was a postdoc with that we could interact with, to do decides part of the work. And that meant that we could advance a lot more in that context. And by the fact that they had funding to do that they sort of moved up in the priority list.	

Interview 13		If you look at the work that we did for the the microbial genomes, sort of still where we had the community asking to work on that, but we've never had a whole lot of dedicate we had some dedicated funds funding for that couple of years ago, but that was only I think, three months over a four year period that we're going in funding so that's kept going slow.	
Interview 13		And then last year, we decided that we need to finish That project. So we decided to add to put more money of our budget to that, which actually will result in an update of the portal that will do normally by the end of March, in order to accommodate that, but it's sort of, yeah. But I can imagine so one of the discussions that's going on now is pictures of animals and specimens that are in collections.	
Interview 13		And we get requests from different people, this is an issue, and then we identify that as an issue, and we try to find additional funds to accommodate that. And that's how it goes. But like I said, if it's somebody else that comes up with that, or it's more other people that come up with that than that, we might go into another direction.	
Interview 13		And the idea is that if we set up that steering committee, is that we ensure better that we don't, by accident, follow just the opinion of a select group of people but that we provide something that's abuse to our array.	
Interview 13	27:22	And of course, what we also get is because of the context we do in life, which were funded in, will also check what we're doing with the goals of (organisation) to see if that corresponds to what we do within the (organisation) project, because if that will match, then it wouldn't make sense to do that. But with most things that we do, we've actually managed quite well to link that to other activities within the (organisation) project.	
Interview 13	30:29		So don't you have a strategic positioning of the platform in mind?
Interview 13	30:37	What do you mean with strategic position?	
Interview 13	30:41		Well, I mean, let's describe it this way. I mean, there's a whole data market available for research and also scientists. So if you thought about how you want to position, the platform, with all the different data available.
Interview 13	31:07		So are do you want to broaden the community size? Or do you want to have like a more intense engagement of the actual community?
Interview 13		Okay, I mean, you mean that in it? I think for us, it's one of the key things that so what we see is that we've got very good relationships with specific countries. So if there's researchers in specific countries that we've been working on for for a while, that's quite good. But we also see that in certain countries that it's not very well developed.	

Interview 13		So for instance, (region), there's lots of relevant and 30 data that's coming from (region). But still, we don't have to the foothold there. In what we're doing. So I think getting more (region)n data out is one of the key aspects. And I think and that's part I'm always reminded I we did we did some data cleaning last weekend.	
Interview 13		And I'm always reminded that actually proper data management for for researchers is one of the key things. So you can look at it at an institutional level. And you can say, well, these institutes needs to have data management plans to curate the data and to advance that.	
Interview 13		But it's my belief, from personal experiences that it actually always starts with the research and if you have a researcher which is very meticulous about entering their data, then you will get good data at the end. But if the data from the start is quite sloppy, then it will stay sloppy and you still see a big variety between countries but also within country.	
Interview 13		Got a hot countries Actually, it's just between researchers. So in any country, you might have a researcher, which does it very good and one doesn't very poorly. And especially with biological data, I think it's important because with something like temperature or salinity, it just device it doesn't measurement, you can do a purely analytical Well, in silico, QC saying, Okay, this value looks great, and we're going to filter it out.	
Interview 13	31:19	But with biological data, because we need to identify a sample there is a face where you need human interaction. And if that is not working, as it should be, you lose a lot of quality, the data and their inequality of the data, I think one of the key item is also	
Interview 13		so when we started back in (year/number), (year/number). We when we had presents data, knowing where species was seen, you're very happy. And now it's more clearer and clearer with what we need in terms of policy, its abundance data. So I think that's going to be the next phase of when we get the next phase of funding, I think that's going to be one of the key aspects is that we don't just need the presence data, but actually have the abundance data.	
Interview 13		And this is actually linked in a bit to the technological evolution. So we try to work with a standard for biological data. And data basically, it's only in the last three to four years that they've developed a way to handle abundance data in a good way.	
Interview 13		So so it's a Yeah, get more of the the non traditional and 30 countries involved in overall get the researchers to do their management of the data at a personal level that don't so we're not we're not looking at Improving how an institute as an institute manager that data, I think that's usually a discussion they have need to have on the road, we can say this is what we recommend for the biological data, but as a whole data management, or how they should work with databases.	

Interview 13	33:54	I think that's that's not something that we should be avoided. And so the thing is that with the technological advances to make sure that these can be integrated in in what we do, and then again, make the data that we can get out of these, these types of Portal more usable and more meaningful in a global context. I think in terms of strategic perspective to who our clients are, it's we try to have everything openly available and that people use the data.	
Interview 13		I'm also not necessarily a fan of having which is sort of in control. addiction, maybe with what we do with with what the goals of (organisation) is that the approach that we've been using is really to have a distributed network. And that we have systems that will find data in different places.	
Interview 13		Because I'm not entirely convinced that that central repository of all data that will work and I think, if you look at there was a big meeting. What's it called ocean data meeting that was that happened last year in October in in Hawaii.	
Interview 13	35:54	And I think it's generally the oceanography community that it's this idea of putting everything in one central place that that's not really working, you really need a network and then interact. But I think by using standards that allow systems to speak with each other that you can advance that, but again, for the biological data, if you put crap data in it's not going to do much at the higher level	
Interview 13	37:00		Because you were talking about the quality of the data, how do you ensure that people upload data with a sufficient quality?
Interview 13		Normally, so we, if we get data will do a number of QC steps, looking at the data, so the simplest thing is, so for the logical data, you do a match with a taxonomy. So check if that taxonomy corresponds to the list that we have then displayed on a map to see if there's anything strange.	
Interview 13		And then you go through the fields or also try to ensure that as many of the fields are filled out as possible, and that's actually one of the trickiest things. Very often, if you've asked data from biologist you'll get the latitude and the longitude and the species but and that's in essence that's correct information.	
Interview 13		But the detail that you have might be limited. And then over time, you might want to know, which was the season, which was the year. And these things, I think a large, large proponent of it is reaching data and working. So what we have is a set a setup where we work with experts, and sort of, we will engage with expert to validate some of the data as well.	
Interview 13	37:13	So when we did the by traffic Atlas, there, we are the experts doing literature reviews, or reviewing data that's available, no BS and gpfs saying, Okay, this is data that we trust, and that data was flagged as being good data. And so yeah, we do not use the process that we have and then interacting with the experts to where we don't have sufficient knowledge because we got everything of all the species in the Antarctic	
Interview 13	39:00		Because you just mentioned trust, how do you ensure trust in the data?

Interview 13	39:08	I think with with the setup that we have with both worlds and working to (platform) there, the key is really that we have researchers that evaluate the data. I think that's the key component there. If you look at (platform), (platform) is about flex more flexible in terms of the data that you accept. And to me it is a bit/.	
Interview 13		Trust is a different concept that with these things, the difficult concept, it's this distrust in in in many directions, this. So trusting the data, I think if you download data from a repository, you should always have the scientific scrutiny to check the data because even in You have all the expert providing the data, I think that can still be mistakes. So there is a responsibility for people that use the data, to look at the data and to analyze it properly and to see if it's fit for their purpose.	
Interview 13		And that's feeding back, then to address the issue that we have with data publishers, the one that we hear a lot is that they don't trust other people to use their data in the appropriate way. So if something is measured in one way, or in another way, if you start mixing that together, you might not be able to compare that in a meaningful way.	
Interview 13	39:40	But it's Yeah, and so that's often a barrier in people publishing their data. And at the same time in people using the data and to solution there is actually to provide more data because if you say this was caught with this device in this one And you provide more details. It allows people to assess better if it's fit for their purpose. But yeah, I think it's your reliability. And a lot of the scientific trust is of being a researcher that publishes a data that has good publications and that has a scientific merit and that will save will trust it. But I think at the same time, there might be citizen science data or from tourists, that's equally valuable.	
Interview 13	41:37	But that's harder to do to bring to the surface.	
Interview 13	41:42		Coming back to the organizational part behind the platform, what are difficulties in managing the platform?
Interview 13		The difficulties, So in our case, I think, so with the funding, it's sort of now it's, sort of long term, but then we still like this year, we have a hiatus. So I think with a lot of these platforms, even if you're talking about five years stability or 10 years stability, and I think there is an issue that remains that if you're too much reliant on money, that is difficult to manage that. In our context, specifically, because we're an international project.	
Interview 13		I think the difficulty there is a bit getting funding from more resources from more sources, because now it's the main funding is from Belgium, so it's also recognized as a Belgian project. So it's not always as easy for others to provide funding to us because But it might be easier for them to pay somebody in their own country to do QC on a data set and provided to us rather than providing the funding to us.	

Interview 13	41:57	So that's a bit the continuity and the stability of what we're doing. And I think in our context is because we're International, it means that you're need to talk with people across the globe. And that's often still difficult even though we have nice technology like Skype, and you can communicate with people but there's still time differences and there's also cultural differences.	
Interview 13	43:39	The argument is that you use, to discuss something with somebody from (country) or somebody from (region) can be quite different. So I think the cultural differences are quite important to consider as well.	
Interview 13	43:58		And what specific goals now now you're aiming for achieving?
Interview 13		But like I said, I think we can mean for for the project for the platform so well so we're now working on an update of our portal. And I hope with doing the update of the portal that we can communicate a bit more directly with people. So now we've had often is true that we didn't communicate as as quickly as good or not in a online visible way we communicate a lot with people that we know and but I think by making more avenues of communication through the website and updates and what we do and providing guidelines, and people get access to information about more easy that we can get more engagement of people or engagement is so that people can already discover a lot of things without having to contact somebody in person so that the barrier for getting involved becomes lower.	
Interview 13	44:08	And I think in general is to continue to develop these aspects to provide access to data, but also to to provide the tools for for for analyzing the data, and to try to continue that. On the long term. I think those are the most important goals at this stage.	
Interview 13	45:36		And how do you control the goals you set?
Interview 13	45:46	You mean the short term goals, long term goals?	
Interview 13	45:49		Is there a difference between them?
Interview 13		Well, if if my goal is to have more stable funding, that's just that's that's that Said necessity, I don't have any control over that. And so I think for the shorter term goals is, that's to our interaction with our now technical steering committee that we sort of said, these are the options that we could do. And this is what's advisable. So one is getting input from Polo researchers in what they consider the priorities.	
Interview 13		Otherwise, there's the funding agency, which is basketball or life which and looking at what are their requests are, and then we try to make the link between those two things in order to prove that so a very concrete example is with so you have worse which we've been working on in the past and are the (organisation) taxonomic backbone, so they want to progress that back backbone, we see that we need to have more enrichment in our data.	

Interview 13		So the goal is to enrich what we have in terms of anti information within the taxonomic backbone, because that's allows us to be more interactive, which is a request that we get from our funding agency to interact more with other projects within nightwatch. So that's our goal that we achieve. And we achieve the goal of the researchers that they want to have worked on that. And I think it's always a combination of that looking at different requests that we get, and then again, annually trying to set up a list of goals.	
Interview 13		But it's mainly what we think is relevant. And so I think that's, again, why I think we should have a scientific steering committee that we have a more objective layer where we can test those ideas. And so, I guess one of the goals by that is also which I forgot is, from an organizational point of view, I think one of the main goals is that we set that in place in During this year, and that we can have a follow up for that.	
Interview 13	45:53	So each year,no, every two years, there's a big Antarctic Science Conference. And so the idea is actually the goal is that when we have that meeting in August, that will be the inaugural meeting for the Scientific Committee that we have made at that point, and then we can move on and try to direct that a bit more in in which way that we go.	
Interview 13	48:29	And yeah, the goals again, it's, I think I mentioned that before, it depends also on where the funding opportunities come from. So if it's so in Belgium, it's it's often you have domestic calls. And so based on what's available within the call, and what we can fit in into the call, that's something that we will work on. And so we'll always have a number of projects that we sort of Trying to launch. And what we actually work on will depend on what's get funding.	
Interview 13	49:25		Good, thank you. So, we finished now the organizational part behind the platform. Now I want to continue more with the data governance and functionality. So in short, you already mentioned you have you offer different analytical tools. So in general, what different possibilities and functionalities does the platform offer.
Interview 13		So what we currently offer is done, yeah. publishing and downloading of occurrence data. So that's according to the Darby-Core (?) Standard, which is used by (platform) and (platform). So the idea is that if you publish data that we allow the data to be published in a big network, and at the same time, the data that we have in our portal comes from both (platform) and (platform). So that you get a lot of information.	
Interview 13		So then we have the taxonomic backbone, which is providing the Taxonomic Information. And so the idea is that we have the interplay between two and that we can enrich that type of information. Then in terms of the art packages, so that's more work that we do with the international community.	

Interview 13	49:34	So we don't necessarily develop all of the packages ourselves. But the idea is that we provide courses on different packages that people can use on how to use those and that we also provide that's when we do the update of our portal will have an update list with what we would call recommended tools for people to use for their analysis. And yeah, I think it's a it's a lot of it is education about best practices.	
Interview 13	50:54		How do you describe the user group?
Interview 13		We assume that they will use it Group is researchers, mostly. So it's researchers. And again, it goes back to what I mentioned early, what we are trying to aim for, I think with our user group is. And that's a sort of a conscious decision is it needs to be people that can do their own analysis that are willing to work with the data and go through the data, data science steps of going through that data.	
Interview 13		And we've had discussions in the past about developing things which are easy to use, and have a nice online interface. But we sort of stepped away from that because the chance of people doing something that might seem sensible but isn't, was too too big. And they're the case the typical example could be like a policy. maker if you say you can create a map with the distribution of the species, but if this model is not working very well you get a something that doesn't mean anything.	
Interview 13	50:58	But if a policymaker then uses that, that would be very bad. And sort of by focusing on these art tools, it sets up a barrier in the way that you analyze the data. But we think it's useful because it requires more knowledge of the people working with the data and in the thoughts that you get oversimplifications. But otherwise, the idea is that the data is out there and people can get do with it what they want.	
Interview 13	52:35		And continuing with this question, do you have other ways of how to ensure that the users use the data scientifically correct?
Interview 13	52:51	I think in reality, very little, I mean, yes, no, it's something Nobody wants to intentionally or not intentionally, if people are going to use it in a wrong way, they're gonna use it in the wrong way. And I think there's there's very little at the time where they're analyzing something that you can do with it, it's I think, then you should have a peer review process where people look at a publication and then judge if something is done, good or bad. I'm sometimes a bit skeptical about that as well.	
Interview 13	53:29		Another question, what difficulties can arise when exchanging data via the platform?
Interview 13	53:44	I think the major issue that we have with a change in data to the platform is is duplication. I think with (platform) and (platform) and an Auckland from below left. The biggest issue that we have is duplication and Then having not enough information about how the data was collected to to make an assessment of I never used the quality of the data, I use the term fit for purpose of the data. So	

Interview 13		but yeah, duplication is a big issue. So there is you might have the researcher that collected the data that publishes the information, or he might not have done it, but he might have published the paper. So somebody else read that paper, and the the literature review of the information on the species or species group, and they might publish that data as well. And that research is and also working at a museum that has a collection, that museum I decide to publish that information as well.	
Interview 13		And so then what I said before is that if there's not enough metadata, additional information around that data, that it might seem that is three observations, while it's actually just one observation of the same animal. And I think that issue boat, if you look at at what we have with our current is database is actually the same with meta data.	
Interview 13		So if you look at identifying data sets, and how that's dispersed in in different platforms, that's one of the key difficulties that we have. Because in the end, you can't it becomes also difficult to find the original source. And I guess that also feeds back into what you said about trust is, I think the key there is that you can find that you should be able to find the original source of information.	
Interview 13	54:14	And that you then can contact that person if you have any questions about how to use the data or about mistakes in the data. But I think with having a dispersed system, it's sort of one of the drawbacks, that there's duplication and we're trying to solve it for a while, but I think the key there is that if people provide enough information. The more information that you have, the more that you can match and clean out.	
Interview 13	56:09		What requirements do you have for the submission of data? Unless to fill out an Mater Mater data?
Interview 13	56:19		Do you have any other requirements?
Interview 13	56:22	They have to submit the metadata and then we do a quality control ourselves. And then if the quality control is acceptable, then that data can be published. But I think, like I said before, as well, I think we're sort of in a turnover. We've been in a in a phase where we've been very happy for any information that we could find, especially in targets often data limited. But I think we're increasing more going just from presence data to abundance data. So I mentioned that in the future that we might really sink it. We need your governance information for As well,	
Interview 13	57:01		having an eye on the clock, I know one hours already over. Do you have time for another five questions?
Interview 13	57:12	No. Okay, that's good. Good point.	
Interview 13	57:15		So, um, how do you encourage researchers to actively use the platform?

Interview 13		for using the platform? I think it's by it's, again, what I said before, I think it's, we are some of the features that we offer are the same as (platform) and (platform). So if you just about downloading the data, they can do that in those places as well. I think for us, the key thing is that we try to enrich data so that you day what they can find in our platform is richer than what they find in another platform, or that they can combine more types of information.	
Interview 13	57:26	I think what we're now most advanced that with that ID is in our microbial portal where we really combining this genomic data with enviromental data, because that's data that you otherwise don't find together in a very usable way. And now we create an interface where people can do an initial search online, they can get some idea of the data, and then they can download the data for more in depth analysis of that information.	
Interview 13	58:27		Do you see more ways to encourage users?
Interview 13		Well, the other thing it's already mentioned that I think it's if we can link the So if you just make a portal to make the data open, then anybody can use it. And and it's it's just that. And I think what is also key is that if we can link us to certain research questions or research groups that have a certain interest, that by listening to them and to try integrate their needs into what the portal is offering, that we can provide a better experience.	
Interview 13	58:51	And I think there is key that we create things that answer specific questions. So like I said, the tracking data, people have specific questions. Now, there's a number of movie ecosystem assessments where they need the abundance data. And so it's basically providing data or data formats that they can use. And I think that's key. But also so that's having the right type of data, but also interacting with the researchers that you can say, Okay, this is your issue. This is how we can can solve this issue.	
Interview 13	1:00:02		One special aspect about the platform that it's located within the scientific community. How do you think does the scientific culture influence the use of the platform?
Interview 13	1:00:32	That's a difficult one. How does the?	
Interview 13	1:00:46		How does the scientific culture influence the use of the platform?
Interview 13	1:00:54	So how I / I think that the type of platform that we have works fairly well, because we're within the interdependency I can imagine if you, we always make the comparison that we use the entire community as sort of a test case. So it's a fairly, it's a relatively small research community.	

Interview 13	1:01:19	So and there is this this mandate that people have, due to the Antarctic system that they should be sharing their data. So I think within that context, we have sort of an advantage that there's more of an inclination from researchers or from institutions to say, okay, we want to share the data. So you get an attempt percentage with with that, that people are more willing to share that data in some context. And so I think that influences in what we're doing in that sense	
Interview 13	1:01:55	if you talk about, the scientific community, with You do still see is that people sometimes the idea that you have to publish papers and that you need to link things to to a paper	
Interview 13		that that stays at an important item. So in terms of data publications, we've noticed that if you tell people, you should, you can also do a data paper, which is just a meta data record, in essence, which is a bit longer, but that's something that people can provide in the tools that we have as well is, but still, you see that researchers will want to create a data paper as well, which is basically the same information that you just pay a publisher 600 euros to get it published.	
Interview 13		And so I think that's sort of, in a way the scientific culture and the citation culture that in a way is holding that that competes because that information could be shared openly in public. From the start. And if they've done their research and they've written their papers, I don't think it has a negative effect of what they could publish, after all.	
Interview 13	1:02:12	So I think that sort of, so you have the openness from the attic on one side, but then you still have this publishing culture that sort of pushes back on that. But it depends in in what level you look at. If you look at the policy level, that then those considerations sort of disappear if you have a researcher. So we have some researchers that work in the fisheries management. You see that they they don't care about publications. So there is it so their restrictions.	
Interview 13	1:03:47		What development Do you see for the platform for the next 10 years?
Interview 13		The next 10 years? Oh, I think there's going to be interoperability with more different data formats and data standards. I think that's going to be the key aspects. So now we're mostly looking at integrating more biological data. And I think one of the key developments is going to be once we can integrate that, that that works better.	
Interview 13	1:03:54	If I look at it from a (organisation) perspective, it's going to be increasing the traceability that we really can say, this data originates from here, and that there's a clear traceability of where the data is coming from. And what a process that has been going, gone through. One of the things but I have no idea what which way that's going to evolve is	
Interview 13	1:04:55	so what's it called?	

Interview 13		So it's they call it the bio chain. So you have blockchain, which is used for Bitcoin. And so that's doing calculations, but it means that what you do is is fixed you can perfectly trace that and have within the life which community that's often mentioned as one of the key evolutions so that you have this chain of whatever happened with with the political data that you had and that this Yeah, permanent records of bourbon and virtual records of data.	
Interview 13	1:05:02	I think that's going to be an important issue, but I don't know how it's going to be resolved. Because if you keep this kind of information in an online environment, you have to maintain that online environment. And I think there I think we're gonna hit sort of a trade off between that we have to decide what we want to do with that because	
Interview 13		It's sort of ambiguous that you're going to create an infrastructure which consumes a lot lots of energy in order to conserve the political world. And which for which resets as researchers quite often that we should consume less energy and and these things. So I think that's going to be one of these things maybe in the next 10 years where we have to decide what we want to do.	
Interview 13	1:06:01	But the way I see I think it's going to evolve into real a network of information, where you can say, I have this question and that goes to the cloud. And you can ask a query and more and more these queries will find the data that you want, and the producer results, which also means that this traceability steps become more and more important, even though they might not be always used.	
Interview 13	1:07:01		What does the success of the platform depends on then?
Interview 13		In that context, I think it's going to stay the same. It's having access to well curated data that has good reliability where you can say, these are sources that we trust, being able to better distinguish between good. And that data, which is a discussion that we've already had for the last 10 years as well. It's, it's again, why I always say fitness for us because it depending on what study you're doing presence data might be good, but you might actually so.	
Interview 13		So I think it's going to be flagging of information and ensuring that you can show the reliability of the data that it's well curated and linking up to to other platforms, but at the same time, Hi, this is also it's now I'm getting quite philosophical at some point is, if all these things are in the cloud, the platform as a portal might become less, less important, actually.	
Interview 13		Because if we have all the scripts and all of the things in at our hands that we can from our own laptop, or some online presence can do these queries and assemble that data. I think a platform as a platform identity sort of becomes useless. We already have it a bit with keep it going.	

Interview 13		Okay, so a lot of the data is in digital systems, and it's very fluent, and it's hard to trace. So the platform on its own, I think, it's only a tool for a community to come together and to work on things. And it should be an enabler. And in theory, that platform as a platform could disappear, but it's the enablement of the community and allowing the communication between researchers and sharing of information.	
Interview 13	1:07:07	That's important. And that's talking a bit against my own shop. But I think in that there could be a concept where you say, well, we need to focus on this community.	
Interview 13	1:09:20	And yeah, the portal can is a tool for that.	
Interview 13	1:09:25		Great. Do you have anything else you want to tell me about success factors or challenges?
Interview 13		Yeah, I think it's the typical thing of financing is important, but I think you need to listen to the community. And I think at some point, it's also useful. But I think part of our success is that we never intend we never set ourselves out to compete with something like (platform) and (platform).	
Interview 13		We've always said we are Part of a big network, we want to contribute to that network. And I think if you want too much to compete, compete and do your own unique individual thing that limits that, and I think it's sort of a natural cycle. You've seen, we've seen it with Bill how worms evolved and obese and other things as well as that.	
Interview 13	1:09:33	You get a number of grassroots IDs. And those might take off but then they might be overtaken by another one. And there will be something new and you see the in everything yet, what was it decades ago yet things like MySpace and now via Facebook, and in five or 10 years, there will be something else then that is Facebook. So it's sort of a natural progression and but that's the surface at the foundation of that, again, it's working at together with the community and whether that's in form A or B that I think is a bit more fluent.	
Interview 13	1:10:59		Great. Okay, thank you. This was very nice. And
Interview 14	Time	Interviewee 14	Interviewer
Interview 14	4:54		Can you briefly explain the history behind it?
Interview 14	5:03	the long history. it gives you (points to a paper on the table) the long history. So, way back in the olden days when I was still postdoc, there was a paper that was published	
Interview 14	5:26	It was published in Nature.	
Interview 14	5:29	And it said that losses on phones, clusters and solution on the high concentration conditions. And this paper was published in Nature.	
Interview 14	5:40	And they presented the data in such a way that it was unintelligent.	
Interview 14	5:47	You couldn't understand the data, you couldn't understand what was going on. There was no access to the data, and it got a lot of people	

Interview 14	5:55	flustered, because you saw downstairs when I showed you that the structure of our data is just this 1D plot of intensity versus angle. The data structure itself is not that complicated. And it's very easy to change, manipulate, reformat, presented in a different way is to lead people down paths of conclusions that don't exist. losses on does not form clusters, concentrations, which was subsequently shown in light publications.	
Interview 14		And there's still a debate in the literature. What that paper did is it really said, okay, there's a lot of data that has been published that hasn't undergone any qualify insurance. It's not accessible. So there's no way to evaluate what the author is saying and of the structural biology techniques small angle scattering did not have a way of validating data, its data structure.	
Interview 14	6:00	And at the time was right stop the process of looking at how do we publish the data in a systematic fashion that is standardized across the field but not so restrictive is to limit the creativity in the (unv.). And this has been a very important balance between demanding standards and starting creativity.	
Interview 14	7:37	touch on this bit later with regards to (platform), because this is was the forefront of that mindset.	
Interview 14	7:47	We could in principle adhere to strict rules of physics, which small angle scattering is embedded in strict rules.	
Interview 14	7:56	But the strict rules of physics as I've mentioned previously, You don't necessarily reflect the reality of biology. And that can be very different. So there has to be a level of creativity that can go in. But subsequent to that mindfulness advisor (name), along with a postdoc in our lab, and (name), my current boss, published a small paper about what you should consider when you're publishing a newsletter coming from this crazy Paer in Nature.	
Interview 14		and it was just a systematic outline of, you know, if a then be a b&b kind of steps that you need to do. And then there was kind of this impetus from the International Union of crystallography, which is sort of a massive voluntary organization that really does set the standards for the structural biology community in terms of x-ray science, mainly X-ray crystallography.	
Interview 14		And obviously now is becoming more and more fashionable. So that's creeping into the icea and NMR spectroscopy. So the International Union of crystallography set up the small angle scattering commission, which my firmer boss and my current boss have been and all are on. And it was decided that there was not only a pressing need to have publication guidelines, but also a means to access the data.	
Interview 14		And my boss being my boss went, Okay, let's do it. Literally, he just said, okay, people want to have a database, we make one. So he did. So when I arrived here, in (year/number), there was no (platform). There was a call for people to start developing these things. And so (name), who's on the first paper, first of all, that was Part of his PhD project, she started set up (platform). So this was literally a major part of a PhD project between (name) and (name).	

Interview 14		And it kind of started from there. So in (year/number), the data bank had been established for the sole reason to provide open access data for people that they could download, they can look at, they could also grab the biologically relevant information. So I suppose the difference between (platform) and just being a database, and I suppose you could call the database or something that has his data, you saw that this data is very, very simple and out of context, you wouldn't know what you're looking at.
Interview 14		If I just showed you the scattering pattern, you go well so we decided that not only We deposit the data. But we turn it more into a data bank where we have all the metadata associated with that experiment. So most of the samples of proteins, we're going to be linking this to (platform) data back.
Interview 14		If there are previously described crystal structures or crystal structure fragments that have been incorporated into the model, and we're going to be linking to the Protein Data Bank. deposit is going to have to define sequence information about their protein, they're going to have to define experimental conditions, what was the sample condition, what was the sample concentration.
Interview 14		So then not only with the people going over (platform) have access to the data that would have access to the experimental conditions as well as the instruments where it was measured, how it was measured, how long it was measured, temperature was measured. So at least there could be a context to this.
Interview 14	8:33	So if someone was to repeat the experiment They are they use this protein from this organism with this modification in this condition on this instrument, we can repeat. And so that was the main impetus, as opposed to just being a data base where you just deposit data under some random number and no noise, what it is. So the impetus came from (name), he thought it was a good idea. And
Interview 14	12:28	(name) and I are very, very different people, but I think we both share the philosophy then why not? Why not just do it.
Interview 14		People wanted then why not? And I think this is a good approach that he has, and it's just developed from there. So that's kind of the background history of (platform) and where it came from, and subsequent to its release. There is this paper by a trowel it out. My boss is on here.
Interview 14	12:37	(name) is not here as a representative of the (institute). This goes into great detail about what needs to be positive (year/number) publication guidelines by Adele and they then also recommend in this paper to deposit the data into (platform) in the tables and quote, the codes in (platform) when you're publishing your work.

Interview 14	13:32	it's now developed into something that the ICR has recognized as something that is of interest and importance to them. And I think going forward will eventually become it will eventually become a what's called a federated data bank with big people on the block, the (platform), (platform), etc. But that still requires a lot of work to become officially federated. Because at the moment, I think one of your questions relates to who funds this.	
Interview 14	14:05	The (instiute) funds this. We've recently had a small grant awarded from I think it's the (insitute) have courteously and very heavily provided us funding for a postdoc.	
Interview 14	14:26	And this is been great because we've only had this for the past, maybe 12 months, then over in (country).	
Interview 14	14:39		It's a long term funding?
Interview 14	14:42	It is long funding. Of course, long term funding is something that is always interesting in the world science, long term and sciences three years I mean, (name) is a senior scientist and staff member. So it's good that it goes on, because he has the resources to to support that funding. And with the support of now the DFG, I think it will start to get more and more backing as a result with very small so in relationship to the number of people.	
Interview 14	15:27	Right now there's myself on the head curator of the database, so I'm the person who literally looks at every single entry and goes through each one, manually checking all the data, checking all the models, checking his information, putting in missing information were deposited this habit	
Interview 14	15:47	and then on the person who approves the entries, and does all the correspondence with respect to revisions, correspondence with restricted deposit is around happy about certain things on the one who fields all of the complaints.	
Interview 14		And I'm also the one that advises us is that you know if they really sure they want to deposit this parameter, because it's quite right. So that's my role. But that's not my why I'm employed here employed as a staff scientist. So I only spend 20% 15% of my my role full time is described as asking to be and the other people who are currently involved now Alex (unv. vgl Paper) he's been with it from the start, along with myself and (name), but he's only 15 20% of time devoted to accessibility as well.	
Interview 14		And he's the lead design developer and validation of Data metrics. So he spearheads what the website looks like what it reports how it reports it, what type of new data validation metrics should we be developing to provide a user an instant quality assessment of the data, etc.	

Interview 14	16:08	So he does lots of work on that. And then there's Clementin Borders (Namen vgl. Paper). He is the person again, he's not a 100%. He's only about 15% and (platform) as well. He is the architecture person, he's the person who does all of the architecture implementations of the data bank to ensure that a good runs and B if there are any changes, modifications or updates that need doing his role is to do those implements the implementation of that at the at the architecture level, and (name)	
Interview 14	17:51	(unv.) Dima, as we call him, because now we have two Demetrius in the group.	
Interview 14	17:59	Dana is the postdoc Who's now just been recently funded through this, this through this DFG grant. So and he's really involved in looking at historical data. So if you want to look at the numbers of personnel, were really, up until the beginning of this year, less than one person.	
Interview 14	18:30		Full time?
Interview 14	18:35	Full time, was working on this. Now with deema, we now have like one and a half, or one in three quarters of a person working on this. So it's already a part time, part time roles, and certainly not the main focus of of, of our standards defined roles at the (unv) station	
Interview 14	18:43		What background Do they have?
Interview 14		So, Clementin is a brilliant database person. I mean, he his expertise is in database architecture, management, design and implementation. So it's great having one These cookies from the computer group is not punk. He's partially funded by our group. But he's, he's, he's, he said in the computer group and it infrastructures here.	
Interview 14		So but he does this. And he also works with the crystallographers. So he's absolutely fantastic to have on board because it makes your life very easy if you need to have this change in front of you, does it? I don't know how he does it, but he's very good at it. And Alexei, as I said, is mainly involved in the design development, spearheading what is reported on the side of what's available to the public, and also we are all sort of involved in catering to the needs of the community.	
Interview 14		So if the community sort of wants this shown, based on not only the (platform) commission, and what it suggests needs to be included, but also just from general people who deposit engines database and market: Why don't you show this? do you think it is A good idea to do? We're always extremely open to feedback suggestions from the community as well as from the official conditions.	
Interview 14	18:45	And we tried to integrate that as best as best we can. We may have differences of opinions about what shouldn't should not be shown. But we try and cater to as much whether we think showing this plot is a waste of time or not. If people wanted, and there's a call for it, then we will go (for it?).	
Interview 14	20:36		And how do you decide?

Interview 14		It's through consensus, Actually. a lot of it is consensus. We will come up with our own ideas about what could be an exciting thing to have. So just a couple of weeks ago, there's now an increase in coal across other data banks. So the (platform) (region) wants to start crawling over (platform) and grabbing the data and creating a validation report and they want certain specific things from us through our API access to the data bank of which there are many combinations.	
Interview 14		And we've sat down and gone, okay, we have to start thinking about our own Validation Reports, how the data is then handled by a third party and to make sure that a third party doesn't change the data in such a way that if they provide a report to person x, and we provide report person x and person x goes why does (platform) say this and why does (platform) say that. you can see the complications of curation, if there's inconsistency in these reports.	
Interview 14		So now I think we're probably going to be working in our own type of validation report, for example, because that's what the community wants. So we do it. We just sit down we go, can we do it? The answer is probably Yes. How do we do it? We've sort of workshop that on a case by case basis. (name) is always coming up with suggestions as well, about happening should be plotted, how things should be presented and what should be there.	
Interview 14	20:38	I mean, the other the other month that three months ago, we had a depositor who wanted to deposit their entire MD simulations, which was over a gigabyte of data. That was interesting, trying to get that in, because we weren't expecting such a huge amount of information to be put in. So we had to sort of juggle things around and change space allocations for them. So we try and just cater to the,	
Interview 14		as I say, the request from the community, the requests from submissions, and he was trying to workshop it As we go with the resources that we had people go Why didn't have Validation Reports already as well as because it requires time, resources and money. And when you've had point seven 5.8 of a person working on this full time for the past six years, it's really hard to, to implement this advanced stuff.	
Interview 14		If you don't have the, if you don't have the funding, the personnel to do. And it's not easy to attract people to do that type of work, either. I mean, we're the (institute), we're here to do structural biology. We're here to do biological experimentation. We're here to, you know, to do that type of work.	
Interview 14	22:52	I mean, most of the people who work here are over in the laboratories doing biological research, she suddenly put your hand up and go, we want someone to do look at the database. It's very hard to get people to do that, especially when the world is moving towards artificial intelligence which is far more tracks. Young people then sitting down going through data.	
Interview 14	24:07		So what is the core offering now?

Interview 14	So the offering now for the the community is is access to small angle scattering data, the focus on structural biology and not only for standard types of experiments, but also for non standard types. So, we're now going into the world of soft matter. And this is what Deem is going to be looking at as part of his postdoc is, it's more than justice proteins and solution.	
Interview 14	Someone might have manufactured a new nano composite material that incorporates biological macromolecules into it. And it's interesting to note what the substructure of that nano composite is. How do we deal with that type of data? We have people who attach proteins to nanoparticles I want to know the effect of the nanoparticle and the protein. That's not just protein solution. That's a soft metal biology by material interface.	
Interview 14	How do we deal with that? So the next step is that what we offer at the moment for those candidates is that I try and modify the presentation, not the data, but the presentation of that type of information as best I can within the standards format that we have at the moment.	
Interview 14	So for example, we had some squid islands. scattering says someone put some squid Orleans's into being scattering patterns of the squid on the whole squid islands. And it took a couple of weeks to get it into the data bank but we got it in and it was there to be reported for people and the deposited in (region) with you're very happy that we're able to do it. Look, it doesn't look right. Doesn't look perfect because, you know, we can't cater to all of these crazy things at the moment anyway. But it's there and it's on offer.	
Interview 14	And it's available. We also offer depositors a whole range of additional validation tools. So for example, they might have done their MD simulations. If they want to deposit them, they can, they might have done alternative biophysical method. So if they've got ultra analytical centrifugation data, they've got multi angle laser light scattering data, and they've got mass spectrometry data they've got on any type of additional biophysics data that supports the claims that they're making in the paper, then we can package that information up and and make it available.	
Interview 14	And as it archives, it's not immediately presented on the website, but it's made available. If they think that that backup biophysical information is important to prove their point or to support claims. The other thing we offer is of course, our links from (platform) to multiple data banks. So we always want the manuscript has been published. We update project titles to the published manuscript title, we provide links from (platform) to the publication, we provide links from (platform) unit problems has been up to the (platform).	
Interview 14	What we're really aiming for is, as as we grow, is for the reverse to start happening as well, to try and get reciprocal links back from the start events into us. So, but at the moment, we're still small. And getting into these larger, more internationally recognized federated data banks is not straightforward. There's certain criteria that we have to fulfill. And I think we're well on the way in getting there, which is good news.	

Interview 14	24:10	And also we offer the facility that now that there are a few journals that require open access and open access data. I think (platform) (platform) does provide that facility for them for those journals and those authors to publish in those journals, that if the General Growth you have to make your data open access, then they can come to us. They can deposit the data, open access requirement is fulfilled. And I think that's a contributing factor to especially when you know, most of this data is paid for by the taxpayer. Having it open access, for me anyway is something that is an important feature of (platform)	
Interview 14	28:40		and what vision I are pursuing?
Interview 14		So, the vision at the moment is is twofold at a practical level. As I mentioned before, there are lots of non standard sort of scatterings burning experiments that go into soft metal that go that sort of combine materials Science with biological science and there is a need to cater for those people in the community who work on their systems. Because at the moment, there is no soft meta scattering data bank.	
Interview 14		And it would be nice to have that and as I say, (name) is called a postdoc working on this integration with with the folks in lubic with manford rossler. And this is sort of the the next sort of practical development that I see going forward. Now how those data are standardized, is going to be up to the soft medic community. One of the advantages of the biological small angle scattering data is that it can be fairly well standardized, there are certain parameters that are fairly well known.	
Interview 14		They're based in physics. And you'd expect extract those parameters from the data with soft matter because the sample is so non uniform from one experiment to another it poses a whole new set of challenges with respect to the type of data that's deposited. Here, I mentioned me to positive one day data that's been processed from the image as well as soft matter, you're going to have to store the images.	
Interview 14		Because the data I'm going to be nice, nicely uniform, as a function of angle, they're going to be all over the split spots and rings and crazy diffuse patterns. And so you're going to have to have a facility to store those images, which then opens up the technical logistics of all, how do we store those images? Where do we store them? Who's paying for the storage, because we're going from a 14 kilobytes dat file to a three and a half megabyte zipped image file, and you can collect nowadays 1000 images in two seconds.	
Interview 14		So who's storing this and we have facilitate yet, because one we don't have the storage space to, we don't know what the standards are or what the soft community want in terms of reporting for their data. So that has to that link has to be established to the soft metal community what they would like to see in such a data bank, and what's important to them.	

Interview 14	28:45	For them, it might not be the finally produce data, it might be that the images are what they want, and what they mean. So it becomes much more of a multivariate problem with regards to the soft material aspect of things. But I think it's kind of important and exciting as well, because then they will have at least an X an open access option	
Interview 14		in terms of becoming more recognizable in the data bank community, not necessarily in the structural biology community, but the Data Bank community is that We do have standard formats of data exchange. With the (platform). We do now have links directly with the (platform). So if crystallographer has (platform) data, they can deposit their crystal structure in the (platform).	
Interview 14		And in parallel through the (platform)s interface deposit data into (platform). Or they can come to (platform) and deposited through our interface which 90% of people do. So that link has been established for some time. And I think within the next year or so I think the (platform) will grab who asks us (platform) maybe if all goes well to become a federated data bank and then wants to become a federated doc data bank, it becomes a lot easier to access co funding.	
Interview 14		Especially if you are applying to large Institutes for that funding. So government body Or, or large governmental organizations, it's very difficult if you are not federated to get that funding, because one of the key features of a data bank is that it needs to be longevity. And that's not easy.	
Interview 14	31:45	When you have such a tiny stuff. It's really it's and I'm extremely happy that it's such (platform) has been able to maintain consistency over the years because there really aren't a lot of us doing it.	
Interview 14	33:38		
Interview 14		And, but once you get that sort of federated title, it adds a level of credibility to it. And then that really starts in opening up future possibilities not only in terms of funding but also attracting people to come work for you. It's pretty hard to say to post Stop for a Senior Technical Officer come work for (platform).	
Interview 14	33:45	And again, what what the hell is it? Yeah, if you can say our federated data bank with (platform) or (platform), then this becomes interesting for them. So I think it also opens those avenues of opportunities going forward as well. But that's in the future. And	
Interview 14	34:25	you know, there are still some things that we need to to go through and discuss. I mean, the success conditions and things have to be happy with that performance. We probably well I would personally think we should ask for an external review from from these types of organizations for them to give us feedback on what they think should or should not do. But this is was this is always something that I'm open and receptive to but it's kind of a like jelly moving forward.	
Interview 14	34:53		Do you have a specific goals you want to achieve?
Interview 14	35:01	My primary goal is to ensure that (platform) is maintained at a level where it remains a credible repository for small angle scattering data.	

Interview 14	35:16	So my time here is limited everyone's time getting NBL is limited. We don't have permanent contracts unless your senior scientist, Demetrius senior scientist, which is okay. But we're all on time	
Interview 14	35:35	and we will leave one thing when you join the NBA, you know that you will be leaving	
Interview 14	35:40	there's a very, very remote chance that you'll be staying on for more than nine years and onwards the eighth.	
Interview 14	35:50	Alexei is even more senior than me in terms of the number of years deema is a postdoctoral fellow.	
Interview 14		You can see How can quickly even the removal of one of these people that's on this current (year/number) paper could result in quite a destabilization of the consistent flow that's currently in the data bank. So what I want to do, personally, is to ensure that as BDB goes on, after one of us, more than one of us have lift the veil.	
Interview 14		And I think by trying and and aiming high with regards to becoming a federated data bank with a larger data bank, and opening up those opportunities, I think that's the first biggest step in ensuring guarantee that longevity, so that's my primary goal. The secondary goal is, of course, to ensure that society be stays up to date and is always responsive to the needs of the community. Because those needs will change over time.	
Interview 14		What was asked for in (year/number) is different to (year/number). People don't think it is people think that physics is set in stone, and it never changes. Or if you become a curator of a data bank, you quickly see that the needs and wants of a community change, and they change in response to fashion, what's new, etc.	
Interview 14	35:59	And it's just human enterprises, any other human enterprise. Someone comes up with a new technique and everyone thinks it's the best thing and it's a quick mad rush to make it an official standard and then someone finds that they've screwed up. You get waves of fashion going through and it's important that I think that (platform) maintains a stable but still fluidic direction going for that it doesn't stifle creativity, especially for the biologists and doesn't overly restrict the field through the imposition of a set of obtainable standards	
Interview 14	38:18	because they normally do some scanning for us to do the perfect scattering experiments just cannot be done experimentally. This is something that is not perfect. So, those are my two personal goals is longevity and to ensure that we remain receptive to this. That means that (platform) needs to have a curator even after on gone, there needs to be living human beings behind it unless you of course come up with a fabulous artificial intelligence way of looking at data and metadata and combining it.	
Interview 14	39:04	If you do that, then you'd quickly probably shift from what you're doing into smelling the scattering and become a superstar in the field.	
Interview 14	39:12	So I know people have been working on this for some time. But you do need a human being to be behind the data bank.	
Interview 14	39:22		And how do you control the goals you set?

Interview 14	39:28	So the control of course, (name) is a great advisor in the directions.	
Interview 14		I also am very lucky that I have quite a lot of leeway in my job. So I try and see if there are opportunities to capitalize on them if I can. So last year, the intrinsically disordered protein community approached me because they, they want to start up a Data Bank of intrinsically disordered by writings which are very, very important in biology. Right and getting the structure of an IDP is not easy because you can personalize them in a marsabit.	
Interview 14		Honey. But with (platform) is fantastic. So they invited me to go to their workshops where they brought the mass spectrum, this and Mr. People and CDs proposals, and there was me assessment of me. And we kind of workshop, the idea of an IDP. What it means at the different techniques, and each of the techniques has a very, very different definition of what an IDP is.	
Interview 14		They're all called intrinsically disordered proteins, but an NMR spectroscopy season completely different nature was x person. But then, over the course of the last year that that workshop turned into a white paper which is turning into an intrinsically disordered community, which is then going to hopefully link in with Alexia codata resources in (region).	
Interview 14	39:38	And so from something small, I can see that we could be an integrated component of, of that community. And it's getting the sport of these types of communities and just putting our face out there that I think is going to be really important going forward. The other one is, of course, this Federation with a PDP and we've we have a very close relationship with the (platform). And I think I'm cautiously optimistic that the Federation within (unv.) who directs that?	
Interview 14		Well, some would say, Oh, the IDP thing that was all me No, that's rubbish. It's it's not rubbish. That was just a small thing that happened. My boss is in support of seeing where it's going to go. Well, it could have as easily blown up and it probably still could Because, you know, the IDP community is funded by these people. And if these people decide to stop funding them, then the whole thing falls apart.	
Interview 14		But the thing is, is that the starting point was there, and it just grew. And if, you know, we all sit down and say, do we think this is a good idea for accessibility pain? Everyone says, Yeah, it's probably a good idea. Let's see where it goes, then. That's how it goes. So that is actually one of the advantages of not having funding from external sources.	
Interview 14	41:41	Because once you apply for external source funding, then you have to say you're going to do these milestones and goals and things. You'd better bloody do them because you've signed a funding agreement. One of the advantages of being internally funded is that if we see opportunity over there, we can go do it. And that's, that's a fantastic freedom to have.	

Interview 14	42:55	So it's, it's it's. Yeah, this is how How it is at the moment it sounds really big. I'm sure that once we become more formalized and the funding arrangements start coming in from other sources, then directions will be much more rigid in their, in their name and their overall objectives.	
Interview 14	43:19		Number work is about success and success factors. So yes, how do you define success or a successful platform?
Interview 14	43:28	Well, that's very good question.	
Interview 14	43:31	For me, success is providing service in context of society be. It's there, the community can elect to use it or not to use it. Not to sound arrogant, but when we came on to the market, they were three dot events. It was by ISIS.	
Interview 14	43:51	Perhaps not the best name.	
Interview 14	43:54	There was also the (platform) bank as well.	
Interview 14		And there was us, and we had grand plans. The grand plan was is that we've already got our standard exchange format that (name) helped develop back in (year/number), which is called this SUSIF (?) format. And the idea was, well, we've already got the standard means of data exchange. You do SASIF, we use SASIF, everyone that SASIF (platform) does SASIF, (platform) is happy because as as it's compatible with this, it's already there.	
Interview 14		It's set up. So let's let's have a federated set of data banks from the beginning - (platform) in (region). and inspire losses (unv?) in (region) and the Department of some database in (country), we all get together and we make it happen. But BIOISIS is dead. It's no longer existing. The present ensemble database is one of these databases that was there then died, it's got brought back to life.	
Interview 14	44:02	Now there's only very, very the slightest form of vital signs in it. So success, yes, we successful. were the only ones around, we are the only available thing.	
Interview 14	45:11	Do we have our critics? Absolutely. Some people in the open access. So some people who, in principle agree with open access, for example, don't want to have the curated data bank, they want a database, they just simply want to satisfy their open access requirements by putting in some data that has no meaning when out of context of their experiment, so they can just say, Oh, we deposited the data into this data base. This is the number which to anyone means nothing, by itself. So one of the criticisms we have is that we asked too much information. We asked about the experiment, the sample, the instrument that you measured.	
Interview 14	45:53	Some people don't like it. They just want to go here's the data.	
Interview 14	46:00	With no validation or anything, but it ticks the open access box technically. I don't agree with this	
Interview 14		position because I think you need to have more information than that. Do I say if someone decided to set up an open access data repository for small angle scattering that people would use it? Yes. Because people are lazy, fundamentally, we all are at the end of the day, if we don't have to do a job, you won't do it rather, either drink, smoke, watch TV.	

Interview 14		And if people can just simply upload garbage data to a data bank that doesn't is just there to store stuff to take an open access qualification, then people will of course, and I don't think that's really been stopped that but I think if we can demonstrate as success in reaching out to other communities of IDP community like making sure that we have, we can demonstrate the we had, you know, active exchange with the depositors as well as these commissions.	
Interview 14	46:07	I think that will, and I hope, ensure that has really been not only goes on, but also hopefully we'll start up other notes around the world using a similar format to what we use. So that would be nice. The (platform) for example, is centered with (institute) and things then it's an (region), it's in (country). And to see (country) (platform), (country) will happen it would be nice that if these other ones - if we become federated - that other nodes of (platform) start forming in the same direction.	
Interview 14	48:00		Other success aspects?
Interview 14	48:04	Not going mad. I think that's a success factor, isnt it? And one thing they never tell you about is that look, most (platform) depositors are nice.	
Interview 14		But there are five or 10% who are not nice. The one aspect that they never tell you, or you don't realize before you get into this job is that you have to have a thick skin. You have to put up with what could be considered considered by some depending on which culture you're from, as abuse.	
Interview 14	48:19	You have to be prepared to stand up for yourself and you have to be psychologically strong. As I say, That's for the 5% of the cases that 95% are okay, but it's the 5% that take up 95% of your time. These these are Things that are not considered when you have a human being curating the data bank.	
Interview 14	49:11	And what's critically important for speed up is that we have to ensure that our perspectives and our opinions about experiments and data quality don't influence the deposition process. Because even though the intent of society be is for open access data, doesn't mean that the experiments and the way they've been interpreted correct, that's still open. an open question and we asked, I absolutely refuse to put my own personal opinion on the quality of data that's coming in. So the metrics that we provide are very much numbers.	
Interview 14		And that is it. I certainly don't write towards repositors: your data is garbage, please go away. It's unfair on them. And it's the responsibility fundamentally, of peer reviews of journals. 93% of suspended entries are in the scientific literature. It's not my job to debate the journals.	
Interview 14		It's not my job to debate reviewers of the articles. If the editor of science says it is okay, then it's okay. Even though it's clearly complete garbage to anyone who's an expert in the field, but it's not for me to judge this in the small percentage of cases. So this is the one one aspect of a living data bank that is often overseen and the biggest criticism we have is why do you accept garbage into society.	

Interview 14	49:58	We do not want We accept everything. It's up to the community to evaluate that. And if it gets published, if you have a problem with this data, then you should be contacting the editor of those journals and asking for traction, etc, or repeat the experiment and show that what's been shown is, it's but you get a lot of criticism. And you have to get up and explain to people in a conference in front of hundreds of people to position themselves speak up and you have to make sure that you remain open to concerns but also defend a position. And this requires a balancing act. And it requires a lot of emotional investment. That's pretty bad.	
Interview 14	51:48		Because you were just mentioned also the quality checks. I mean, how do you ensure the quality?
Interview 14	51:55	so it's very luckily because small angle scattering is based in the world of Physics. The for example, the radius of generation of a protein is a well defined metric.	
Interview 14	52:09	So the depositor says it's got an RG of 2.5. Well, it either has or has not, and that you can't, you just can't get around that. So if it comes back that you put the data into the processing software, and it comes back with a different RG, then I'm sorry, if it's different, then what the depositor says it is is not correct.	
Interview 14		In that case, I would probably then enter an email correspondence exchange saying, you cannot get this number from this data. Because your calculated Yeah, yeah, because you can count I mean, and it's not like you don't need software to use an Excel spreadsheet because it's just a basic transformation plot.	
Interview 14	52:38	But you can literally do it on a piece of paper. You don't even need Excel. You can literally use a piece of paper to work out this number, it doesn't take so long. It only takes like a 10th of a second to do it in that data processing software, I mean, basically instantaneous. And either this talking numbers right versus wrong. And then there are other things like How noisy the data is? Well, if it's very noisy, and someone's drawn a line, like a flood of a bumblebee through it, you can save look,	
Interview 14		come on your fitting noise. And but there are ways of evaluating this impressive program that you can be using. Just put it in and go here. You've got the data measured from point A to B, but really only one 10th of the data is usable. And it's just a number that gets spit out. So the type of metrics that we report it (platform)P are well, RG Is that right? Yes or no? Yes tick. The maximum particle dimension is it Yes or no? Yes. Is the data too noisy? Yes. No. Does the model you develop for the data? Yes, no. So we just report back those metrics.	
Interview 14		And we don't sort of assign any sort of judgment today. And I think it's important because sometimes people want to show that the model but for it was doesn't reflect the solution state. And this is a question I keep posing. But what if I want to show that what everyone thinks is the structure based on say the crystal is not what's going on the solution. They want to show something that doesn't fit.	

Interview 14		Then we report the fit as being complete crap, but that's the point. So we can't say this is a good fit, or a bad fit, we can just say it doesn't fit. And that might be actually good for the author because they want to point that the crystal structure is garbage? And what's going on and solution is correct.	
Interview 14	53:32	So we don't ascribe these these judgment qualifies today to the metrics at all. It's all very numbers. And that's it. And then it's up to people to decide what the numbers mean, and how to interpret them in context of what's being presented.	
Interview 14	55:26		And can you ensure that the people are using the data scientifically correct?
Interview 14	55:33	No, I mean that we have no oversight on how the data are used after they deposited. People wish to download the data, they can have a use, how they use the data, we have no control. So we really don't have any detailed information on how the data is processed in the use.	
Interview 14		you know, our only already caveats, of course with this is if commercial clients or commercial company or someone that has a product may have proprietary rights over their data, we make it very clear that yes, you can deposit the data. But please ensure that when it's released, that it's okay to release it.	
Interview 14	55:56	Because if it's proprietary and then gets used by third party and they want something else, you can see how a chain of events, especially in political science can lead to a complete disaster. So, with those more commercial sides of things, then yes, we do, but that happened very rarely is anything happening to companies, the positive spin up, but we're very, very careful that that information	
Interview 14	56:55	is released at an appropriate time on On the request of the of the company,	
Interview 14	57:01		so related to data quality is also trust in the data. How do you ensure trust?
Interview 14		So the data quality is based in common sense, in a big way, when you look at garbage (platform) data is really quite obvious. The quality of the data is the primary responsibility of the depositor not access (platform). So, for example, the other week, I had great data that was deposited by someone but they didn't include the metadata. So they didn't include any sample ID or anything about writing it was, etc, etc. But the data was fantastic.	
Interview 14		So I had to ask them for revision, I said we need to know is further information you can send it to us, we can It will dry. Then basically on the same day I received another deposition from a completely different set of people. And the data were completly misinterpreted, that were complete garbage. But they had satisfied all of the requirements for deposition and (platform). So I instantly assign them the code.	

Interview 14		So I know the data or crap, but it's not up to me to make a decision. As a curator, it's up to the community to make that decision whether or not that data is reflecting what the authors and making friends and that becomes really, really difficult. When that crap data that's it hits all the publication guidelines, (unv.) are made. It's garbage. It gets published. And then (platform) is left with garbage data that satisfies everything in standards. But still garbage as published and accepted. Then it just says like this forever.	
Interview 14		And if people have a problem with it, then I need to address the author or the journal. So yeah, sometimes it's a bit depressing. We have people who have great data, and then they don't provide the additional stuff and you have to send it back. So they typically come back very quickly with all the relevant information. And we can put it in. So, so yes, how the data is actually handeld post deposition, I know that. In terms of API's, the IDP community, the Elexir IDP community that I mentioned before, have requested certain things that they can access.	
Interview 14		So they can use our API's to crawl over our data bank and grab the necessary information that they need for their projects. So for their project, they need a listing of (platform)s codes. So we insist that depositors put a (platform) code in all projects, unless of course, it's purely synthetic protein, or it's an organism that hasn't been annotated. In any progress. There aren't many. And at the moment, we kind of have our architecture over here. We have society be organized in terms of projects, but they want to know, we want to we want to organize in terms of unipro codes.	
Interview 14		So I, for example, wrote the API to ensure that, yes, IDP community, if you access this API from our website, you can grab it and then you can organize (platform) by (platform) code. And by by tag, what tags Do you want, we can put IDP on the IDP tags, or will flexible protein on the flexible protein.	
Interview 14	57:11	And so we're currently in the process of adding tags to our entries going through and so then if they want to organize by (platform) code and tag, they can. So I know that and so I know that that's going on. the (platform) in (region) wanted something where they can grab the data and do transformation plots. For the validation reports, we're in the process now of	
Interview 14		writing or writing the API's for limbs unable to do that what they particularly want. But we're also going to be putting on a GitHub this, how we do the plotting in (platform). So if you want to replicate the blocks, exactly, you can, etc, etc. So I know that there are those, those people, the (platform) in (region) and RDP community here in (region), using iprs to progress mentioned and the (platform) in (country) have already got their own little sub website where they use an API to gather information and they represented in exactly that in a different format to us, but they report the same information.	
Interview 14	1:01:15	So I know that that goes on but at an individual depositor level or someone who's individually looking at the website, we we don't have information on, on how the data is being used by by those people.	

Interview 14	1:02:17		Because we were just talking about you were talking about the user. Yeah. How do you describe the user group?
Interview 14		So the user group is a wide ranging cacophony of people. So the user group can be defined by as little as I'm a (persoonalised). And I did one small angle scattering X ray experiment. And these are the results and we're writing a paper and this is going to be three sentences in our overall paper because I spent the past five years looking at shift essays.	
Interview 14	1:02:23	And is this that type of user all the way through to the full on my God, I've been here This for 20 years. And will I do a small angle scattering	
Interview 14	1:03:05	but x rays and neutrons (unv.), the neutron scattering community and, you know, hardcore entire paper is just small angles, x rays or neutron scattering. And it's a heavy heavy reading. So the the user community spans the completely pragmatic my god I have to get it in because my supervisor told me because I'm better on my thesis and I need to submit this now or otherwise papers and kind of get accepted all the way through to the with taking this very seriously. Please make sure that everything is reported correctly. Cross checks, double validators	
Interview 14	1:03:48		Those people all have more or less biological background?
Interview 14	1:03:51	Yes, Yes, I do. Everyone has the biological background. We haven't had any requests from pure hardcore software. People yet? Yes.	
Interview 14	1:04:05	Not not specifically not the most weirdest. The weirdest samples have been used by material crossovers, but they're almost always biologists. In these biomaterial areas, we haven't had someone who's interested in looking at the phone on propagation of certain gels under pressure and high temperature would she have, for example, we've had none of none of this type of stuff being requested to be deposited.	
Interview 14	1:04:37		
Interview 14		so the biggest is, of course, these guidelines from the ICR. Almost all structural biologists either do crystallography or NMR on and these days, em, so they have the well aware of ICR and how it's going so I mean, our biggest proponent is the ICR. Yeah, they're the ones who have this small angle scattering task force. They're the ones who have these micro symposiums every, every every four years or so or how many years they have ICR conferences.	
Interview 14		This is a major mechanism of communication. And then I mean, some journals now of course demanded open access is fulfilled. This year, I think we will, we will approach some journals maybe. So, (journal) is one that we will probably approach their big publishing house, but they they have very strict requirements about what they accept. So, you have to prove that you've been around for this long you need to prove that you've had this many of the positives, you have to prove that you're still growing, etc, etc. (platform) satisfied all of that now, because we're sort of getting into the six year period.	

Interview 14		And I think we can demonstrate that with longevity and you can understand why journals wants to make sure All that the data bank that they demanded that forces deposit into is going to be around because it's a bit silly if it gets closed down. So, but there are some journals that are absolutely, I mean, I think there are a couple of journals in (region), they always say you have to deposit this in (platform), and that's voluntary on their behalf.	
Interview 14	1:04:42	We have not officially approached journals at all ICR journals have taken up the cause and said yes, we should people who publish ICR journals should be doing this. So there's that kind of thing. And of course, we're always going to many conferences and venues for for this. So, people do not do know about this and what you have to remember is that compared to crystallography and and in a mass spectroscopy, small angle scattering is a sliver of the size of community. So it doesn't take much to get the word around that this data banks around in our community because the community is very small start with, you know, it's it's tiny. So in relation to.	
Interview 14	1:07:15		You already explained me some functionalities and possibilities of the data bank. And just to make sure that I got everything.
Interview 14	1:07:31	So you can look up so you can upload that you can download that additional data as well as the additional.	
Interview 14	1:07:33		data as well. What other functionalities?
Interview 14		So we have these little validation slider bars on there. So if people who are unfamiliar, we'll see whether this entry fits in the sort of the Blue Zone which is good or the red zone which is not so good relative to the other depositions in the data bank. So they can sort of immediately assess where this deposition fits in terms of overall quality. But aside from that, and aside from storing we, you know, we have had pages on our website, we explained parameters.	
Interview 14		We have links to these papers, we explain what's going to scattering is a help page. There's a somewhat long deposition guide was I wrote it, I have urgency to override things. But there's a deposition God and things like this available on our website. And of course, then it's the standard search functions. This browse is keyword search, this author search, there's publication search, there's instrument search, there's country search, it goes on and on.	
Interview 14		But you can search by specific beamline. So it's, it's quite, that's what we present to the to the world, fundamentally, and that's what's made available to the people. So the second paper by Alexis Alexei really goes through all of this. Yeah, the first page is a bit more technical.	

Interview 14	1:07:38	This one's a bit more user friendly in terms of understanding what the hell's going on in terms of our Yes, this is good with your best. Not so good. And you don't have to be an expert to know that it's in the red. So maybe there's something a bit funny going on. And it talks about, what, what's what's made available and what's required in terms of the metadata in this paper, and then how the, how the data are validated in terms of quality, and some of the challenges facing the field.	
Interview 14	1:09:40		And what problems can arise when exchanging data via the data bank.
Interview 14	1:09:50	I think it's a fairly straightforward process.	
Interview 14		In terms of data exchange, I think our API's are pretty good and they are the big one. Use them they can. And it's all very consistent. The biggest, the biggest complexity of this data bank is actually just dealing with other human beings. It really is. I mean, because you have very stressed people saying, I mean, I receive the requests to the deposition, and I have to go through and I have to curate it. And you know, this is not something, as I said, is not my main job. Typically, I do this first thing when I get up in the mornings, before I come to work.	
Interview 14		And, you know, some people are so stressed that they, they will literally deposit and then five minutes later send an email going, where's my coach? Because I need it. Because the editor of a journal is about to reject us. We've got this and four hours to get back into it. Then why did you leave it to four hours before rejection, to deposit into (platform)? And so dealing with that pressure is tricky, especially when they're not first in the list. You have a list of priorities, you know, you have to prioritize these I prioritize by when I receive them.	
Interview 14		Yeah. So if I receive you before that person, then I serve that person first. So basic kind of, I'm at the supermarket, I have to wait in line. The supermarket market person doesn't go No, you back. I like you better. You're more stressed than the person that I'm serving, you get to go first.	
Interview 14	1:09:54	But somehow those rules of etiquette go out the window. When you're dealing with the data bank, because they just think there's an order box behind it. They don't think that there's a human actually. And so having to deal with that, that that really is the biggest challenge. It's not actually the data, dissemination of data or the reporting of data. It's actually dealing with people's shit.	
Interview 14	1:11:56		Coming to a different question: how do you estimate the usability of the data?
Interview 14		Well, I think the usability is from just a pure, purely pragmatic side, the usability of the data is that it's there. And that's important to demonstrate, for good reason. The biggest utility of the data is just shown that if a group in bomb has taken half a million euros of taxpayers money, and on the project I'm sorry, there is a responsibility that don't get reported somebody, you know, the taxpayers have paid for this data. Yeah.	
Interview 14		So it should be available, but the taxpayer might not know what the hell it is. But the fact that it's there and can be evaluated and criticized and critiqued.	

Interview 14	1:12:05	agreed to debated. I think that is a role that cannot be underestimated in terms of taxpayers paying money for this project. And I think there is a responsibility on on on groups to report that and I'm glad (platform) up is there to facilitate them in being able to do it. And I really think this is the plan in terms of its scientific validity.	
Interview 14	1:13:35	We don't know yet. I think it's still too early.	
Interview 14	1:13:40	There are one or two data sets in there that have created the base.	
Interview 14		Interestingly, the most debatable data sets and the most controversial data sets the authors don't want to deposit into (platform) (platform). So there's a data set right now that's floating around in the literature that says certain methods can could be wrong. And this method has been around for the last 12 years.	
Interview 14		And they often say it's wrong. But they will not deposit the data into (platform) (platform). So on one side I have both is claiming method is wrong. They have not deposited the data. Inside be, I've got the person who developed the method saying where's the data and (platform). So I can't, I can't contact the authors for them to deposit.	
Interview 14	1:13:45	So here I'm, again in the sandwich of personal crisis, that I have to try and mediate as best I can. Because he is ideally they will provide the data. So the person who developed the metric would at least grab it and show that Actually, no, there's nothing wrong with my method. You just don't know how to use it properly. You know, but so often the most controversial things I'll lift out besides people	
Interview 14	1:14:59		anyway, A while ago, you told me the story with the other two data banks,
Interview 14	1:15:04	which was unfortunate that they went they went and but that was because of funding.	
Interview 14	1:15:10		So my question in this regard is how do you strategically position the data bank on the data market? Let's say it this way?
Interview 14	1:15:19	Well, it's pretty hard to say when you're the only one, right.	
Interview 14	1:15:25		My strategic position is that, for me, personally, we're always open to other people and their initiatives.
Interview 14		If they wish to set up an alternative, then I think it's critically important that society be engaged with the alternative. Because I'm always more authors, Marissa kind of a person. I think the more people involved in one or multiple projects, that funding We have the same overall goals of the community at heart. It's important to work together. I think for this and not against each other, working against each other.	

Interview 14	1:15:35	I think it's a waste of time. And if it's in its most stressful I think active engagements would be there and I think (platform) has, as best as can try to strategically place itself in the position that if other people wish to jump on board or they wish to develop their own platforms, then we should be able to respectfully integrate with him. without someone being the best or the worst, or it's not so good. You don't do this. You don't know. I'm not interested in that kind of discourse at all. It should be. Well look, if	
Interview 14	1:16:55	(platform) (country) wants to start doing this morning good scattering and depositing the lead Absolutely. Yep. Can we help on this? Are you interested in it? And hopefully we try to do.	
Interview 14	1:17:11		I have one more question about the submission process. You said that, you know, the researcher, I have to give us certain information about metadata. Yes. Do we have other requirements for submission?
Interview 14	1:17:25	Not really we have, we have quite a few options for the submission. So there are us, you know, people have different packages analyze different data, we've got options for people to upload data that have processed from different packages. We're trying to automate this as best we can. But this is very, very difficult because software developers do not maintain consistency over time and they suddenly switch to the format of the way that they do data.	
Interview 14	1:17:55	This fundamentally, I think we've pretty flexible at the point In a deposition,	
Interview 14	1:18:02	and aside from what's outlined in this (year/number) paper, in terms of the requirements that are needed for your standard deposition, we don't really ask for anything more. But we're open to requests. I mean, if people want to deposit ultra analytical centrifugation data, they can simply do this. They don't do it at the point of deposition. The special requests are always done by me through email correspondence. So I'll get a question. Question, but I've got last week, for example, was we've done the study we have these models.	
Interview 14		There are, as I say, maybe 20 or 30 models, we don't want to deposit all of them one by one. What do we do? Well, maybe deposit one or two representatives. Just do your deposition as if then package up any other additional information you want you email me or set up a cloud link or something like that.	
Interview 14	1:18:40	Grab it, and then I can add it to you. What we won't do is I don't think for the moment anyway, we're going to add the facility to add massive folders at through the points of deposition, for two reasons security, because you don't know what's in this folder and to wait we don't have unlimited storage resources. And we don't want people swapping the data bank with just useless never to be used information.	

Interview 14	1:19:39	However, if there's a requirement by for example, besides commission to say you have to start depositing your 2d images, then we're going to need we need to get funding for this because we can't fit it. So special requests can be catered to, not at the point of deposition, but through email correspondence afterwards.	
Interview 14	1:19:59		What kind of challenges do you have in managing the data?
Interview 14	1:20:09	I think the the challenges at a technical level are just ensuring that if we want to implement something new, like a validation report, or trying to ensure that the data reported in (platform), it's the consistent hub of the data. So, as opposed to people coming in grabbing our data and then re processing it and coming up with different conclusions, we provide those people with an already set standard of Yes, this parameter is this we don't care what you say, what how you process the data, this is what the deposit said it was.	
Interview 14	1:20:45	There is there is this and one of the aspects are there which which relations , do you? Sorry.	
Interview 14	1:20:58		What challenges do you as a manager?
Interview 14	1:21:00	Yep. Well, there's also the technical challenges, but (name) is very, very on top of that. Some of the technical challenges are, of course, that a, the, the underlying architecture of the data bank, which is by gender platform is always being updated. This has to be implemented is always technical bugs. We're always running into certain things create errors on our servers that we quickly know about, because the deposited will tell us.	
Interview 14		But there's also making sure that sort of this technical implementation through this, this web framework is kept up to date, which is not trivial. And (name) does a great job at making sure that when there are updates, or when certain software's go out of software support this. It's done in a seamless manner.	
Interview 14	1:21:31	So for example, we have a production version of (platform). And we have the public version, and they're basically nervous. each other, that (name) will always implement updates and infrastructural changes, tactics, changes on the production server first and do thorough testing before we put it into production. So there's always this model. But that's actually a very technical challenge going forward.	
Interview 14	1:22:20	The other technical challenges, I suppose, the increasing need for more space. It's a technical challenge.	
Interview 14		In terms of users, the technical challenge from this is education. And this (year/number) paper by Adele has been fantastic in lifting the overall education community, because although the requirements in this paper I believe, personally are overly onerous, what they have done is they've lifted the level to such a high esteem that most people although they can't reach them At least try to get there. Which means that I have seen an improvement in the quality of data and experiments over the past five years compared to what was being deposited in (year/number).	

Interview 14		What's been positive now in (year/number), on average is better. And it comes down to having someone or group of people at the top saying that shall adhere to the Ark of the Covenant. It's more negative, scattered, and people are doing it. So education is a critical component from the user side. From the curation side, as I mentioned, the challenges to people, that's always a challenge.	
Interview 14		I think from the software side, the challenge is software developers just need to start capturing more information in their in their processes. So if you go to a beam line, and the beam line records all of the metadata information Already, I would like to see software developers from stop stripping this and the processing software. So the users then have to go back to find out where it was, it would be nice to have this sort of metadata flowing through the whole process.	
Interview 14	1:22:31	So the depositor just has to upload the dat file or the data on (platform). And everything gets passed and automatically fields. And they don't have to look back to (year/number) from this beam line, in this phase of the moon when they collected this data. That would be nice. It would be nice if software developers were consistent over time. So for example, we have just simple three column Format Data, right? angle, functional angle, record s, intensity and error.	
Interview 14		Kind of straightforward that you want to plop first two columns x versus y with an error. Don't swap the error and Why column makes it really difficult and not only that don't have a regular for years and then do a software update and then suddenly swap the column. Because that small change in software means that we can no longer read that file automatically. And we have to then update our architecture to read the reformatted file from (year).	
Interview 14		And before versus (year). And after, which means then we have to ask the user for the version number of the software if they want to automatically pass it because otherwise then they have to type in the parameters manually which uses don't like doing the go why kind of really needy can't explain to them, the software developer change the format something ridiculous.	
Interview 14	1:24:50	So they're also something that and that, again, I think comes back to education, but education for software developers who do some crazy stuff over time and just just be consistent. So that I suppose there that's sort of a three Things that software development side, the user side and, and the architecture.	
Interview 14	1:26:05		But one special aspect about (platform) is that it's within the scientific community. Yes,
Interview 14	1:26:11	very much.	
Interview 14	1:26:13		how do you think does the scientific culture influence the use of the platform?

Interview 14		Oh, God, fashion dictates everything in science. Right now every structural biologist on the planet thinks electron microscopy is the best thing since gold pressed (unv.). Everyone is running towards the date, because everyone's getting nature papers. I mean, I'm sorry, the only way to get ahead in science is if you published in high impact journals.	
Interview 14	1:26:16	It's the fact of life if you have candidate a has an impact factor journals 10 and above, and candidate B who does not. trust me candidate a is going to get employed. It's just a fact of life. It's the cold harsh reality of science. And now, you literally do the EM microscopy structure of a coffee bean, you're going to get into Nature. Everyone's capitalizing on this and they're running towards it. It's complete fashion.	
Interview 14	1:27:07	It's fashion.	
Interview 14		They talk about the resolution revolution and electron microscopy, true. What's happened? Ironically, no one talks about the resolution. They go, we solved the structure of the (unv.) 3.5 angstroms, using cryo electron microscopy, and you go, yes, it's still a blob. You haven't told me what's happening at three and a half angstrom resolution. crystallographers, they will tell you what's going on that for angstrom resolution, they will tell you what's going on two angstrom resolution, I'll tell you what's going on.	
Interview 14		That will literally say that amino acid flip from that position to that position and move by, you know, they will tell crystallographers obsessed with resolution. Electron Microscopy is suddenly had this resolution revolution that I talked about resolution, they still published blobss but they're important you blobs they could publish in nature.	
Interview 14	1:27:09	it's all fashion. And the winds of fashion will come and go. 10 years ago it was all free electron lasers and how you're going to be delivering single molecules of losses on into a free electron laser. Any any first year PhD student working in a wet lab would have told the physicists you can't do it.	
Interview 14	1:28:25	It's not possible. because you just can't get a singular molecule of losses on to be happy in a vacuum being flooded through a free electron laser. But every single paper and except for (unv) was (journal) will science back than. I hadn't seen the next (unv.) science paper in the long time. now all and I see our journal, (unv.) but every other paper in (journal) at the moment is EM, well in four years time. This will be ICR journal, PCP and structure. it'll, it'll, it'll died. So fashion is the biggest challenge.	
Interview 14	1:29:11	Because scientists want bang for their buck. They want a career, they want to feed their families. And funding agencies want to see the glitz and the glam.	
Interview 14	1:29:23	They want to see two (journal) papers as a post doc, physical chemistry. That's just the way it is.	
Interview 14	1:29:32		And what development Do you see for (platform)?
Interview 14	1:29:38	in terms of that negotiating that environment, I think, in general is escaping the visible visibility of the technique.	

Interview 14	1:29:45	And I think this goes a long way. Because (platform) is a pretty small section in structural biology, and it could easily be crushed by fashion. But small angle scattering has been around longer than crystallography. I've been around of all of the techniques in x-ray science, or electron science. It's been around the longest in around since early last century, people seem to fail to recognize it's gone through over the fashions. It still produces a blob. But it can be interpreters and still going because the technique is robust.	
Interview 14	1:30:27	And oh, yeah, I think (platform) plays a role in making sure that it remains a visible technique and of relevance to a community that's so fickle (fragil?). Structural biologists, methodological consumers.	
Interview 14		If you went to an ice conference in the late 20th century, it was all small molecules. You'd see about two protein structures. Now you go to an ICR conference, well, maybe not the last Been before 90% of it is protein crystals, the postman molecule people have stuck out the shed with the back.	
Interview 14		Talking about the electron orbitals, no one cares. No last time ICR was 5%, small molecule 80% protein macromolecule and 20% am next ICR will be 80% in your proposal and you're going Why is this a crystallography conference? To be talking about crystallography anymore? Because that's what structural biology, let's say a technique that gets them to a place they use it, they take it, they can see them and then move on.	
Interview 14	1:30:47	And it's important that (platform) is there to just be the little annoying nerd in the background of the class gain wisdom here. After almost 400 years. We're still here.	
Interview 14	1:31:51		And what does the success depend on them?
Interview 14		The success I think Depends on collaboration. And I think also having an open attitude towards techniques.	
Interview 14		I think the journey of the field is far more interesting than the results from the journey is fantastic in terms of technological advancements. But that can be incorporated. And I think as long as that's kind of the basis philosophy of moving forward, then I think it's gonna get better and will survive and it will benefit the community.	
Interview 14	1:31:56	Not only because it does reveal valuable information is one of the fastest ways of getting information without too much hustle. And that's his real strength. And as long as small angle scattering doesn't box itself off, which I don't think you can do, I think	
Interview 14	1:33:14	this is something that the future needs to be consciously aware of.	
Interview 15	Time	Interviewee 15	Interviewer
Interview 15			Das heißt Sie sind dann ja auch bei der (platform) involviert, ne?

Interview 15		Genau, das Projekt, sag mal, ist aus einer Diplomarbeit heraus entstanden. Die da, ich sage mal, (name) mit betreut habe. Und Mitarbeiter das letztendlich jetzt fester bestnadteil der Insituts-quasi-Publikationsdatenindrastruktur. Ja,a lso, die direkte Programmierung, das macht ein Mitarbeiter, der (name). So ist letztendnedes die Einordung von dem (platform)-System.	
Interview 15	04:17		Das heißt dann, welche weitere Personen oder Organisation waren dann involviert bei der Implementierung?
Interview 15		Also, ich sage mal, insgesamt - ich weiß es jetzt nicht mehr aus dem Kopf - waren es jetzt glaube ich drei Abschlussabreiten, die sich mit der - und auch Praktikum gehörz dazu - mit der Implementierung quasi beschäftigt haben. Innerhalb / Institutionsell sind es jetzt schwerpunktsmäßig zwei.	
Interview 15		Das heißt hauptsächlich die, ich sage mal, Umsetzung, Konzeption passierte schon am (institute), aber letzendlich mit Verbundpartner haben wir letztendlich Softwaretest gemacht, ich sage mal, Konfigurationstest und letztendlich in verschiedenen IT Umgebungen, das letztendlich Installieren zu können. Und hauptsächlich ist war jetzt das Forschungszentrum (town), was letztendlich eben viel Input quasi gegeben hat.	
Interview 15	05:25		Wie würden Sie denn jetzt das Kernangebot von der Plattform beschreiben?
Interview 15		Das Kernangebot der Plattform ist letztendlich eine, ich sage mal, eine Infrastrukturm, die auf der Idee quasi von Edge Computing bzw. und Premis Infrastruktur letzendlich aufbaut um Forschungsdaten, die im großen Umfang in Forschungsinstituten letzendlich anfallen aber auch lokal gehalten werden müssen wegen Ihres Charakters, dass diese Forschungsdaten eben publiziert werden können. Publiziert im Sinne von Verfolgung öffentlicher Standards im Bereich Zitation.	
Interview 15		Das heißt, eine Vergabe von DOIs, Document Object Identifiers, und letztendlich ein minimalen Satz von technischen-administrativen Metadaten um letzendlich, ich sage mal, eine Referenzqualität für die Forschungsdaten zu erreichen, die dadurch eben auch ja wissenschaftlichen Aufsätzen, Papieren, letzendlich eben Referenzierbar sind und dadurch eben dauerhaft ja zitierbar gehalten werden. Und die Besonderen Charakteri, Charakteristika der Forschungsdaten, die letztendlich im e!DAL-(platform) System veröffentlicht werden, sind letztendlich der (-) ich sage mal oftmals sehr (-) domänenübergreifende Charakter und letztendlich auch so einen Charakter von Forschungsdaten, die letztenendes nicht in öffentliche Repositorien eben passen von, ich sage mal, von der Datendomäne letztenendes dann halt her, z.B. Phänotypisierungsdaten, Pflanzenphänotypisierungsdaten, für die es letztenedes keine öffentliche Repositorien gibt.	

Interview 15		Grundsätzlich eben aufgrund des Datenvolums, der letztendlich eben ja (-) höheren dreistelligen Gigabytebereich letzendlich dann halt eben liegen kann, was eben öffentliche Repositeren eben auch nicht anbieten bzw. was dann einfach letztenendes monetär zu teuer wäre und auch von einer Datentransfer zu externen Einrichtung letzendlich eben da, ich sage mal, die Netzwerkbandbreite eben nicht ausreicht oder eben auch, ich sage mal, Eigentümerschaftsbedenken letztendenes eben dann exisiteren	
Interview 15	03:55		Nun handelt meine (work) ja von den Erfolgsfaktoren. Was ist denn für sie persönlich Erfolg bzw. eine erfolgreiche Plattform?
Interview 15	04:08	(--) Sie meinen letztenende im globalen Sinne sondern nicht nur für das spezielle Repositorie, oder was meinen Sie?	
Interview 15			Beide Möglichkeiten gehen.
Interview 15		Also Erfolg ist letztenendes die Akzeptanz. Von zum Einen den Forschenden, die aktiv Daten bereit sind über die Infrastrukturen in einem (-) also nach FAIR Kriterien zur Verfügung zu stellen, als letzendlich auch die Nachnutzung der Daten aktiv durch die Forschungscommunity. Das wären letzendlich die beiden zwei hauptsächlichen, ich sage mal, wissenschafltichen Erfolgsfaktoren	
Interview 15	05:03		Sehen Sie da noch weitere Aspekte, die da mit reinfließen?
Interview 15		Nein	
Interview 15			Ok, gut.
Interview 15			Verfolgen Sie denn eine Vision mit der (platform)?
Interview 15		Na, die zentrale, ich sage mal, Fragestellung ist letzendlich die, ich sage mal, die Forschungsdaten als Asset zu begreifen. Letzendlich dass nicht nur die geschriebenen Papiere einen Mehrwert in der wissenschaftlandschaft darstellen sondern letztendes die Forschungsdaten an sich halt auch.	
Interview 15	05:22	Und diese werden häufig zu geringem Maße publiziert und auch in zu geringem Maße mit Metadaten versehen, das letzendes ein Generationsübergreifender Bewahrung des Wissens eben stattfindet als auch letzendlich die, die Wertschätzung - so würde ich es mal formulieren - für die Arbeit im Bereich Datenerhebung, Datenkuration, Datenanalyse. Letzendlich im Bereich (-) wissenschaftliche Gütekriterien mit zu etablieren. Gütekriterien in dem Sinne, dass letzendlich der Impact der, der wissenschaftlichen Arbeit eben auch mit genutzt, abgeleitet wird aus den veröffentlichten Daten und der Qualität der veröffentlichten Daten.	
Interview 15		also Sie kennen ja die Impact Factoren wie den Hirsch Index als Zitationsmetriken zum Beispiel und das wäre eine Vision, da eine Datenzitationsmetrik aber auch basierend auf der Datenqualität letzendlich eben mit so einem wissenschaftliches Portfolio von einem, von einem, von einem, von einem Wissenschaftler mit aufzunehmen.	

Interview 15	07:20	Das wäre der eine Aspekte. Und der andere Aspekt wäre die Wissensbewahrung. Das letzendlich die Vielzahl der Daten, die erhoben werden, ich sage mal, in einer entsprechenden Qualität für die Zukunft verfügbar gehalten werden.	
Interview 15	07:40		Gut, das waren ja schonmal viele Aufgaben mit der Plattform. Kommen wir jetzt zu einem Bereich, der sich eher hinter dem organisatiorischen Bereich der Plattform befindet
Interview 15			Wie ist denn der organisatorische Aufbau hinter Plattform?
Interview 15		Was meinen Sie mit organisatorischen Aufbau?	
Interview 15			In dem Sinne, ob / Naja, Sie haben ja jetzt gesagt, es arbeiten eher zwei Menschen dran, richtig?
Interview 15		Mehr oder weniger, ja. Ich sage mal, die Weiterentwicklung wird konkret von zwei Leuten vorgenommen. Die Begutachtung der eingereichten Datensätze das machen eben am (institute) drei Mitarbeiter, in (town), wo es noch eine Installation gibt, ist das momentan noch nicht so ganz klar. Aber da ist eine ähnliche Revieworganisationsstruktur vorgesehen wie am (institute). Das heißt, es gibt drei, die auf die Qualität der submitteten Daten gucken.	
Interview 15		Das wäre der betriebsaspekt, wenn du das unter Organisation verstehen. Weiterentwicklung zwei, Beguachtung und den operativen Betrieb vom System darf man natürlich auch nicht vergessen, das ist letzendlich die IT entsprechend im Hause. Dann ist jeweiligen Server halt wartet.	
Interview 15		Das ist aber von Personalstellen relativ schwierig zu bennen, weil das eben nicht nur dediziert eine Plattform ist die rein für das (platform) betrieben wird sondern das läuft auf einer ganzen Infrastruktur-Service-Ebene, was letzendlich über Speicherdatenbanken, Netzwerk und so weiter in ein zentral (-) Services-Level von der (institute)-IT zur Verfügung gestellt wird.	
Interview 15	10:00		Und wie sind dann die unterschiedlichen Verantwortlichkeiten für die Plattform verteilt?
Interview 15		Also Verantwortlich für den reinen IT-Betrieb ist die (institute)-Bioinformatik und IT-Gruppe und hier letztendlich die Teilbereich-Serveradministration. Die Weiterentwicklung ist im Wissenschaftsbetrieb angesiedelt. Das heißt, die Anforderungen aufzunehmen, die Kommunikation auch das Training.	
Interview 15		Wie gesagt, ein bis zwei Mitarbeiter kümmern sich letzendlich darum. Und die Qualitätscheck und Kommunikation mit den Nutzern das passiert mit drei Leuten. Aber natürlich nicht hauptberuflich, sondern es ist eben wie im wissenschaftsbetrieb üblich mit allen anderen Arbeiten verzahnt.	
Interview 15	11:06		Wie werden denn Entscheidungen bezüglich der Plattform getroffen und wie sind dann die Entscheidungswege?
Interview 15		Entscheidungen in welcher Hinsicht? Bezüglich Erweiterungen, Nutzeranforderungen?	

Interview 15			Ja, genau - wer kann was bestimmten oder wie ist da so die Absprache untereinander?
Interview 15		Die ist eher förderal. Letztendlich ist es so, dass hier eine gewisse Gleichberechtigung bezüglich Ideengebeung quasi ist, welche Features vielleicht noch interessant wäre. Final gibt es / eigentlich ist es in der Verantwortung derjenigen, die das System weiterentwickeln, die beiden Personen.	
Interview 15		Es betrifft letzendlich einen Mitarbeiter,(name), und ich mache da in der Diskussion auch mit, aber letztendlich so eine Idee-Feedback-Runde, die da exisitert. Aber es ist jetzt keine Hierachie, die irgewndwo jetzt festlegt, wie was entwickelt wird. Weil die Nutzeranforderungen sind manigfaltig und dann muss man letztendlich schauen bezüglich Zeitbudget von der Priorisierung.	
Interview 15		Was halt am entscheidensten ist natürlich erstmal Fehlerbereinigung, das ist quasi ganz klar, den operativen Betrieb aufrecht zu erhalten und was jetzt eben neue Feature auf technischer Ebene anbelangt, das entscheidet der (name) alleine.	
Interview 15		Also das ist letzendlich, weil das muss ja wuasi implementiert werden. Das sind eher so flache Entscheidungsebenen, wenn ich das mal so zusammenfassen darf.	
Interview 15	13:12		Als Sie dann die Idee mit der Plattform hatten und das dann auch umgesetzt haben, haben Sie irgendwie eine strategie entwickelt wie sie sich dann auf dem Markt oder auf dem Datenmarkt, nne ich es mal- positionieren wollen?
Interview 15		Also wir arbeiten ja im wissenschaftlichen Umfeld und von daher ist natürlich der Markt schon da bezüglich Aufbau der eigenen Reputation. Die eigene Strategie basiert quasi auf dem klassischen Verfahren. Das heißt, man fährt zu Konferenz, man macht eben, man arbeitet an Veröffentlichungen und versucht eben einen großen Impact zu erreichen indem man in entsprechendne Konsortionen dieses Thema einbringt, Trainingskurse veranstaltet.	
Interview 15		Das ist jetzt mehr oder weniger diese übliche Wissenschaftsstrategie um die eigenen Forschungsergebnisse letztendlich in der Öffentlichkeit zu präsentieren um eine gewisse Attraktivität für die wissenschaftliche Community hat zu bilden. Von daher kann man nicht von einem speziellen Strategiekonzept für eine ich sage mal Softwareplattform wie dass das e!DAL (platform) jetzt ist, sondern es trifft ja im Allgemeinen auf die Verbreitung von wissenschaftlichen Ergebnissen letztendluch halt zu, ja.	

Interview 15		Also es ist jetzt weniger als Produkt zu begreifen sondern als Serviceangebot, das im wissenschaftlichen Kontext einfach entstanden ist, weil es eine Notwendigkeit gab, die eben von dem Fehlen von Repositorien für Forschungsdaten (-) mit dem Charakteristika, die ich am Anfang erwähnt haben, eben resultiert hat. Und dadurch haben wir jetzt nicht eine Marktstrategie in dem Sinne entwickelt um ein Produkt zu platzieren und damit irgendiwe Geld zu verdienen. Sondern wir haben einen Bedarf gesehen und dazu eine Lösung angeboten. Also, ich will mal so drauf antworten auf Ihre Frage.	
Interview 15			Jetzt haben Sie ja schon das Geld angesprochen. Wie erfolgt denn dann die Finanzierung der Plattform?
Interview 15		Kernfinanzierung der (institute)	
Interview 15			Das heißt dann genauer was?
Interview 15		Es gibt letzendlich, ich sage mal, eine Zuweisung von Bund und Ländern, das ist eine Leibnizeinrichtung das (institute). Und darüber werden, ja, wird der Wissenschaftsbetrieb, der Servicebetrieb und alles anderes finanziert. Anschubsfinanzierung für das Projekt kam von einem Drittmittelprojekt, das ist nun mittlerweile ausgelaufen.	
Interview 15	15:28	Und jetzt wurde das System in diese Kernfinanzierung vom (institute) mitgenommen. Das heißt jetzt konkret, was ich eben erzählt habe, dass die IT vom (institute), dieses System auf dem, der (institute) gewarteten Infrastruktur laufen lässt. Und (-) die angestellten Personen sind aus (institute) quasi Kernfinanzierungshaushaltsmittel. Das heißt wir nehmen für diese Plattform einfach kein Geld, das ist ein freier Service.	
Interview 15			Erfolgt die Finanzierung denn auch langfristig oder muss die immer wieder erneuert werden?
Interview 15	16:35	In dem Fall ist sie langfristig finanziert. Da gibt es eine konkrete Zugabe, Zusage von der (institute) Geschäftsführung das dieses System dauerhauft finanziert ist.	
Interview 15			Das ist ja auch schön.
Interview 15		Mal noch ein Kommentarzu Ihrer Feststellung. Im Bereich wissenschaftlichen Datenrepositorien haben wir eine gewisse Funding Crisis, das können Sie auch in wissenschaftlichen Papieren nachlesen, das da das Problem ist, dass Datenrepositorien häufig eben entwickelt werden, auch bereitgestellt werden, Daten eingespeist werden aber aufgrund von fehlendem Dauerfunding häufig nicht mehr weiter betrieben werden können.	
Interview 15		Und da manchmal auch keine Exitstrategien gibt um Datenbestände zum Beispiel zu überführen oder die Systeme woanders hosten zu lassen, sieht man beio öffentlichen Datenbanken häufig das Problem, dass die nach fünf bis zehn Jahren häufig nicht mehr da sind. Also da gibt es dann auch, wenn Sie das für Ihre (work) vielleicht dann auch näher recherchieren wollen: In (-) einer Zeitrschrift (journal)	

Interview 15		Am Anfang des Jahres gibt es immer eine Publikation von wichtigen Datenbanken in den Lebenswissenschaften. Und wenn man die letztendlich über die Jahre verfolgt, dann ist dann eben nur noch ein Bruchteil der dort veröffentlichten Datenbanken tatsächlich noch operativ.	
Interview 15		Also insofern ist Ihre Feststellung wirklich entscheidend, dass es sehr schön ist, wenn solche Systeme in das Kernfunding von Foreschungseinrichtungen überführt werden können.	
Interview 15	18:30		Dann, bei welcher Frage war ich jetzt - ich musste nochmal kurz darüber nachdenken. Meine nächste Frage lautet dann: welche Schwierigkeiten und Heuasforderungen sehen sie - mal abgesehen von dem finanziellen, was sie gerade angesprochen haben - für Plattformen oder auch die Organisation hinter der Plattform
Interview 15		Qualifiziertes Personal zu bekommen um das weiterzuführen und die (-) ich sage mal, dass Dissimination. Also die Verteilung der Information, dass es diese Plattform gibt. Und letztendlich die Nutzerschulung, also Training. Das sind extreme Ressourcen, die man da halt reinstecken muss um nicht nur das System zu betreiben sondern auch weiter zu entwickeln und die Nutzercommunity wirklich eben zu unterstützen.	
Interview 15		Weil das Feedback aßer der Nutzercommunity ist extreme wichtig um letzendlich aktuelle Bedürfnisse auch abbilden zu können. Und das ist eine wirkliche Herausforderung in dem Gebiet, weil es eben durch, das muss durch Personal abgedeckt sein. Und da wir keine Firma sind, die damit Geld verdient, ist es eben so, dass es immer nur nebenher gemacht wird. Und das ist manchmal eben nicht ausreichend.	
Interview 15	20:04		Am Anfang vom Interview haben Sie mir ja einen Überblick gegeben von der Plattform und welche Vision Sie haben und auch welche Ziele sie stecken. Wie kontrollieren Sie denn die Ziele, die sie sich selber geben?
Interview 15	20:23	Das ist eine gute Frage. Die Ziele, die wir uns selber geben, ist, wird über Metriken halt versucht zu Quantifizieren. Metriken in dem Sinne wie die Plattform genutzt wird, die (-) Papiere, die zur Plattform geschrieben wurden und auch das Trainingsmaterial wie das referenziert und genutzt wird. Also das würde ich jetzt kurz und knapp antworten.	
Interview 15		Also da sind mal eben auf die eher quantitativen Metriken angewiesen wie Downloadzahlen, Zitierungen von den wissenschaftlichen Aufsätzen zum (platform)-System oder auch die Zitierung der im (platform) hochgeladenen und veröffentlichten Datenbestände. Das ist letzendlich Feedback.	

Interview 15		Und direktes Feedback zu den Zielen ist auch die Akzeptanz in der User Community, das ist ein weicher Faktor, den kann man durch Feedback Gespräche rauskriegen, durch Einladungen an Projektanträgen mnitzumachen, wo das System mitbenutz werden soll und solche Sachen. Das sind letzendlich diese weiche Faktoren, die nicht quantifizierbar sind.	
Interview 15	21:50		Damit haben wir jetzt den großen Blog um die Organisation dahinter. Jetzt möchte ich in den nächsten Blog, das geht mehr um die Nutzung oder auch der Governance von der Plattform.
Interview 15			Zuerst einmal, sie haben es am Anfang schon erwähnt, das doppelt sich jetzt vielleicht etwas, aber für mich jetzt nochmal so zur darstellung:
Interview 15			Welche Funktionalitäten und Möglichkeiten bietet die Plattform?
Interview 15		Daten zu veröffentlichen, um das quasi mal so zusammenzufassen. Also ich habe es tatsächlich am Anfang schon so ein bisschen erwähnt, diese eben spezialitiertes Edge beziehungsweise onPremise Computing, die Daten letztendlich vor Ort zu behalten und auch vor Ort, da wo sie eben anfallen, Institutionen, zu veröffentlichen.	
Interview 15		Daneben bietet es weite Recherchemöglichkeiten. Das mit international etablierten Standards angereicherte Metainformationen werden bereitgestellt, es wird für Suchmaschinen aufbereitet, entsprechend Landingpages zur Verfügung gestellt, es bietet auch Programmierschnittstellen umd das vielleicht zu erwähnen - das habe ich glaube ich am Anfang nicht erwähnt. Es kann in bestehende Infrastrukturen eingebettet werden.	
Interview 15			Wie beschreiben Sie dann die Nutzer der Plattform? Es sind natürlich Wissenschaftler, aber in welchen Feldern dann?
Interview 15		Das ist schwierig zu sagen, weil die Nutzer sind, arbeiten anonym. Wir haben kein Authentizierungsmechanismen, jeder kann sich die Daten frei runterladen. Wenn man sich die Statistiken anguckt, geht das weltweit. Also hauptsächliche Nutzer kommen aus dem Europäischen Raum.	
Interview 15		ich würde vermuten, dass es im europäischen Forschungskontext Nutzer sind, aber da haben wir keine spezielle Statisitk. Also Nordamerikanische Zugriffe sind auch stark verzeichnet, aber auch aus dem asiatischen, insbesondere (country), Indien, sind da stark vertreten. Aber welche konkreten Nutzerprofile das sind, kann ich ehrlich gesagt nicht sagen. Also ob das Firmen, Privatanwender oder hauptsächlich eben Forschungstreibende, wo ich jetzt von ausgehen, da haben wir keine Statiskten drüber.	
Interview 15	24:32		Welche Regeln unterliegen denn die Nutzer?
Interview 15		Regel im Sinne von?	
Interview 15			Oftmals ist die Bedingung ist das zu zitieren oder ob sie sich dann anmelden - dann ja nicht.

Interview 15		Also die Regel ist so, wie es im wissenschaftliche Umfeld üblich ist, wenn die Daten genutzt werden, müssen die Daten zitiert werden. Dazu geben wir Zitierhinweise, wie so ein Datensatz zu zitieren ist. Dann gelten letztendlich die Lizenzbedinung, die der jeweilige Autor spezifiziert für den jeweiligen Datensatz, das wäre vielleicht noch zu erwähnen.	
Interview 15			Das heißt, es gibt dann keine grundlegende Bestimmung für die gesamte Plattform sondern das ist dann Datensatzabhängig.
Interview 15		Für die Nutzung gibt es keine grundsätzliche Bestimmung. Für das Data Deposition, also die Ablage, gibt es schon eine Bestimmung. Da gibt es ein (-) ein Lizenz Agreement, das ist dann von jedem, der Daten submitten will, zu akzeptieren. Da geht es darum, dass gewisser Ausschuss von Garantien exisitert zur Dauerverfügbarkeit. Also solche Dinge, wenn eben das Repository eben mal eine Stunde ausfällt, sind wir da nicht haftbar.	
Interview 15	25:25	Dann muss letztendlich das Data Ownership ganz klar an dem Lizenzagreement geklärt. Das letzendlich wenn die Daten zu uns hochgeladen werden, dass die im Eigentum des Nutzers bleiben. Und werden Hinweise geben zu diesem Lizenztypen für die eingestellten Daten, dass der Autor möglich Open Access Lizenzen nutzen sollen. Also wenn Sie da konkrete Interesse haben an allen Details für die Daten Submitter, dann können sie das Lizenz Agreement von der (institute) Webiste für das e!DAL System herunterladen, da ist es verlinkt.	
Interview 15			Gibt es sonst noch weitere rechlichte Bestimmungen, die die Plattform betreffen?
Interview 15		Nein, die sind alle in dem Lizenz Agreement aufgeschrieben.	
Interview 15			Wie schätzen Sie denn die Nachnutzbarkeit der Daten auf der Plattform ein?
Interview 15		Gut bis sehr gut. Das hängt von der Metadatenannotation konkret halt ab. Weil wir (-) also es ist ein System, was ein relativ freien Upload von Daten zur Verfügung stellt mit nur einen minimalen Satz von technisch-administrativen Metadaten - ich habe es am Anfang erwähnt. Konkret handelt es sich um Dublin-Core-Standartd, der da implementiert ist. (-) Und die Daten, die an sich publiziert sind, die haben verschiedene Nachnutzbarkeitgüten.	
Interview 15		Das hängt von dem jeweiligen Autor. Beim Review Prozess der submitteten Daten achten wir auf mindestens die Verwendung von offenen Dateiformaten, dass keine propäritären Daten abgelegt werden. Beispielsweise offene Bildformate oder offene Datenstrukturen, das eben vorzugsweise CSV, Jason als Format abgelegt wird und letztendlich keine Binärdaten, wo es jetzt eben keine Software für gibt, um die der Allgemeinheit zur Verfügung zu stellen umd die auslesen zu können.	

Interview 15		Das ist ein Bereich. Nachnutzung wäre wichtig zu erwähnen. Was aber die konkreten semantischen Metadaten anbelangt, also die Beschreibung, also inhaltliche Beschreibung der Daten, da ist noch relativ viel Luft nach oben, weil das aber auch die Eigenschaft von dem System, weil wir ein breites Spektrum von (--) Daten aus den Pflanzenwissenschaften akzeptieren, da gibt es eben noch nicht den einen Mindeststandard um die Semantik von den Daten zu beschreiben.	
Interview 15		Weil Dublin-Core als Mindeste eine inhaltiche Beschreibung erfodert, textuell zwar, können wir eigentlich davon ausgehen, dass der Kontext der Daten sehr gut nachvollziehbar ist und dementsprechend an sich gut nachbenutzt werden können. Insbesondere auch weil die Erzeuger der, der Datensätze eindeutig identifiziert hinterlegt werden, sodass man immer noch einen Kontaktpunkt hat, falls es irgendiwe Fragen zur Datennachnutzung gibt.	
Interview 15	29:50		Wie stellen Sie denn dann sicher / Wie stellen Sie denn dann die Qualität der Daten sicher abgesehen jetzt von den Metadaten?
Interview 15		Gar nicht. Also die Qualität der Daten das unterliegt nicht dem Reviewprozess. Das obliegt dem Autor. Wir stellen eigentlich nur die Qualität der beschreibenden Information, wie gesagt die Dublin Core-Metadaten sicher. Die gucken wir dann an und gucken dann auch ob die Daten auf technischer Ebene die qualität haben, im Sinne von Zugriff verwenden kann, aber inhaltlich können wir das nicht beurteilen.	
Interview 15		Das ist ieben Aufgabe vom Wissenschaftler. Da gibt es häufig eine Paperpublikation dazu, die natürlich auch reviewed wird. Im Wissenschaftsbetrieb ist das ja nun üblich, wenn man ein Papier veröffentlichen will - wie bei Ihrer (work) gibt es ja auch Gutachter.	
Interview 15		Und das ist letzendlich die inhaltliche Überprüfung. Das passiert aber nicht für alle Datensätze, das muss man eben auch ganz ehrlich sagen, aber das ist auch nicht machbar. Da sprechen sie einen wichtigen Punkt an: Datenqualität. Aber das ist im wissenschaftsbetrieb immer noch eine offene Frage, wie denn die Qualität von Daten überprüft werden kann. Ich sage mal mit den jetzigen Resourcen nicht möglich. Insbesondere auch die Überüprüfung von veröffentlichten Artikeln ist so, dass einfach nun keine angestellte Personen machen sondern das machen ja Wissenschaftler, die in Institutionen weltweit arbeiten und das nebenher, neben ihrer eigenen Arbeit leisten, die Publikation zu begutachten - so wie es ihr Betreuer in (town) eben auch macht.	
Interview 15	30:47	Aber um die Antwort nochmal zusammen zu fassen: wir sind uns das Problems bwwusst,aber wir können es uns nicht leisten. Also Datenqualität ist wirklich ein Problem.	
Interview 15	32:20		Eng verzahnt mit der Datenqualität ist ja auch das Vertrauen in die Daten. Welche Möglichkeiten sehen Sie denn, da Vertrauen aufzubauen?

Interview 15		Vertrauen basiert ja letztendlich als förderierter Prozess für, im Rahmen der Nachnutzung. Das heißt, wenn es Zweifel an der Qualität der Daten gibt, dann wird es ja meistens dann offensichtlicht, wenn konkurrierende Wissenschaftler sich die Daten angucken und mit Ihren eigenen Ergebnissen vergleichen.	
Interview 15		Wenn da gewisse Zweifel exisiteren, dann wird das natürlich in der Wissenschaftscommunity kommuniziert. Aber es ist natürlich ein Vorschussvertrauen. Andererseits ist, wenn jemand erstmal nachgewiesen wurden, dass seine Daten nicht korrekt erhoben wurden oder auch falsch sind, dann ist die Vertrauensbasis nicht mehr da.	
Interview 15		Wie sie schon festgestellt haben, Qualität ist aktuell eine Vertrauenssache, weil es einfach nicht kontrolliert werden kann.	
Interview 15			Nun haben Sie ja auch schon erzählt, dass Sie keine Nutzerprofile habe, da ist bei mir die Frage aufgekommen, wie sie denn versuchen sicherzustellen, dass mit den Daten ethisch korrekt umgegangen wird.
Interview 15		Wir lassen nicht zu, ethisch fragwürdig Daten zu publizieren. Also im Pflanzenumfeld ist es weniger problematisch als im medizinsichen Umfeld, oder generell mit Daten im Bereich von Menschen. Also bei uns spielt eher das Nugero Protokoll eine Rolle, dass wir die Nachnutzung der Ergebnisse sicherstellen müssen bezüglich der Herkunftsländer des Pflanzenmaterials als auch Spezialfall.	
Interview 15	34:00	Wenn es sich um bedrohte Arten handelt, dass wir die Fundorte nicht mit veröfentlichen dürfen um Touristen oder auch kommerziell interessierte Leute davon abzuhalten die schon bedrohte Art komplett auszurotten, weil wir den Fundort zur Verfügung stellen. Das wäre ein Aspekt im Bereich Ethik, den wir uns im Kontekt mit der (platform) verpflichtet fühlen.	
Interview 15	35:22		Das ist dann die Ethik im Sinne von welche Daten werden dann hochgeladen oder zur Verfügung gestellt.
Interview 15		Genau	
Interview 15			Sehen Sie auch einen Einfluss auf die Nutzer, dass die mit der Datenverwertung umgehen können?
Interview 15		Beschreiben Sie mal die Ethik der Datenverwendung.	
Interview 15			Im Sinne, dass (-) dass man den Datensatz auch so aufbaut, dass man Ihn nicht weiter verfälschen kann oder dass man weiß, dass der und der die Daten erstellt hat. Gibt es da irgendwelche Möglichkeiten?
Interview 15		Das sind die Lizenzen einfach. Jeder Datensatz liegt ja unter einer gewissen Lizenz. Wir haben usn wie gesagt dafür entschieden die Open Access Lizenzen zu empfehlen. Das ist die Creative Commons Lizenzfamilie. Da ist außer der CC0 Lizenz ist verpflichtend die Autoren oder den Ursprung der verwendeten Daten, wenn ich die für meine Analyse weiter verwenden möchte, anzugehen. Also das ist im Bereich Zitationsethik, wenn ich Sie richtig verstanden haben.	

Interview 15			Am Anfang haben Sie erzählt, dass Sie für die, auf die Plattform aufmerksam machen im Sinne von Kongresen usw. Welche weiteren Möglichkeiten sehen Sie denn aktiv Nutzer zu generieren, die außerhalb von dem wissenschaftlichen Kontext sind oder ist das nicht so relevant?
Interview 15	37:06	Das ist durchaus schon relevant. Also Soziale Medien spielen da natürlich eine Rolle. Twitter hauptsächlich ist im Wissenschaftsumfeld immer beliebter. Das ist ein Medium, aber auch Plattformen wie Wikipedia, was natürlich einen großen Impact hat, Youtube Trainingsvideos zu Plattform, da arbeiten wir tatsächlich konkret dran um die Wissenschaftskommunikation zu / noch ein bisschen weiter zu hinterlegen, aber das ist natürlich auch ein Resourcen Problem.	
Interview 15			Was meinen Sie mit Problem?
Interview 15		Es muss ja jemand machen (lachen)	
Interview 15			(lachen) Ja, das stimmt wohl.
Interview 15			Generell ist die Besonderheit ider Plattform ja auch, dass Sie in die Wissenschaft integriert ist. Inwieweit schätzen Sie dann ein, dass die wissenschaftliche Kultur die Plattform beeinflusst?
Interview 15		Die beeinflusst sie sehr stark. Es gibt letztendlich wissenschaftsdomänenspezifisch oftmals eione andere Datennutzungskultur. Also bei den Astronomiewissenschaften ist es so, dass es ohne Datenpublikation und Nachnutzung gar nicht geht, weil einfach die Instrumente zu teuer sind. Weil nicht jeder kann sich als NAtion ein Hubble Teleskop im Weltraum leisten. Von daher ist man da bezüglich Datenutzung drauf angewiesen.	
Interview 15		Und das funktioniert eben auch ganz gut. In den Lebenswissenschaften funktioniert es in allen Bereichen noch nicht so wirklich gut. Das hängt aber auch damit zusammen, dass die Wertschätzung der Datenerhebung, und Datenqualitätsicherung und Datenkuration eben nicht so hoch ist wie ein wissenschaftlicher Aufsatz. In anderen Bereichen wie Sozialwissenschaften spielt das (-) eine große Rolle um auch im Bereich Belegwesen, das heißt, stimmt die Umfrageauswertung eigentlich, kann ich auf die einzelnen Umfragen, Interviews zugreifen.	
Interview 15	38:11	Da spielt das auch noch eine große Rolle. Also ich würde jetzt einfach das differenziert beantworten. Also es ist nicht im Wissenschaftsumfeld allgemein gut oder schlecht sondern es hängt wirklich mit den einzelnen Wieenschaftsdomänen zusammen oder wissenschaftlichen Fachrichtung.	
Interview 15		Also es reicht von sehr gut bis schlecht.	
Interview 15			Ok, Um nochmal zurück auf (platform) zu kommen. Welche Entwicklung sehen Sie denn für die Plattform in den nächsten Jahren?

Interview 15		(5) Also in den nächsten Jahren sehe ich, dass der Bedarf an Forschungsdatenpublikationen steigen wird - auch im Bereich Datenvolumen. Die Anbindung an Cloud-Computing-Infrastrukturen wird entscheidend sein für die Plattform als auch den Machinenlesbaren Zugriff auf die Datenbeschreibung als uach die Dateninhalte zu gewähren. Das wären die drei großen Aufgaben, die jetzt zukünftig zu sehen sind.	
Interview 15			Und wovon hängt dann der Erfolg der Plattform ab?
Interview 15		Von der Plattform. Also die Nutzerbedarfe zu identifizieren und in technische Lösungen zu überführen. Also das ist der Entscheidende Erfolgsfaktoren. Und die Nutzerbedarfe zu bekommen, das ist eben die schwierige Aufgaben	
Interview 15			Und wo sehen Sie sich dabei?
Interview 15	42:01	In der Kommunikation - Wissenschaftskommunikation.	
Interview 15			
Interview 16	Time	Interviewee 16	Interviewer
Interview 16	00:07:08		Was ist dann die Geschichte hinter der (platform)?
Interview 16		Die Geschichte hinter der (platform) ist, die wurde (year/number) von der (country)ischen Gesellschaft für Massenspektrometrie entwickelt. Und die haben damals einfach gesagt, jetzt kommen eben die ersten Instrumente, die hochaufläsenden Massenspektrometer auf den Markt und die kommen aus dem Metabolomics-Bereich ursprünglich - hauptsächlich in Meetabolomics. Es gibt aber noch keine kommerziell verfügbaren MAssenspektren-Bibliotheken für hochauflösende Daten.	
Interview 16	00:07:09	Also High equity Daten / das sind im wesentlichen vorhandene Datenbanken - und die Wileys Libaries - aber die sind damals hauptsächlich entstanden, zu madamliger Zeit waren die hauptsächlich für GCMS und da gab es halt so ein bisschen LCMS Daten. Und die haben halt gesagt, da ist einfach eine Lücke da, das gibt es kommerziell nicht.	

Interview 16	00:08:04	Und dabei haben sie eben angefangen und sagen Ok, wir machen dann eine (platform)- Library, wo die Leute ihre Sachen dann heutzutage OpenAccess zur Verfügung stellen können und hochladen können. Das heißt die Leute messen dann ihre Sstandards oder identifizieren, was sie dort eben Umweltproben (unv) / GEnau, und die haben dann angefangen. Und das ist jetzt eine sehr typoffene Library. Das ist nicht an ein Instrument gebunden oder an eine Messtechnologie. Sondern sie sgt einfach, ok, wir haben das Format so offen gestaltet, dass man eben alle Daten an Massenspektrometriedaten da hochladen kann. Und der Hintergrund der (platform) (region) ist, dass wir - wir haben eine Organisation, die nennt sich (organisation). Ich kann dir da vielleicht auch noch einen Link schicken oder so. Und das ist ein Zusammenschluss, also eine non-for Profit Organisation, von verschiedenen europäischen Institutionen und Behörden, die die Identifikiation und Bestimmung von sogenannten emerence pollutants verbessern sollen. Und in dem Rahmen haben wir eben (year/number)/(year/number) gesagt, wir wollen eben ein (platform) server hier in (region) aufbauen. Seit dem betreibe ich das auch.	
Interview 16	00:09:35	Und das ist im Prinzip, weil die Idee dahinter, dass es eben nicht nur für Metabolomics oder Life Science Massenspektren sein soll, sondern auch für Umwelt proben. Das heißt, Umweltchemikalien. Das heißt, wir haben ganz viele / früher war das eben Naturstoffe und so - oder Biomarker oder so- und heute haben wir ganz viele Arzneimittel oder Pestizide oder andere Stoffe in der (platform)	
Interview 16	00:10:10		Was ist denn jetzt das Kernangebot der (platform)?
Interview 16	00:10:15	Das Kernangebot der (platform) ist im Prinzip die Bereitstellung von Massenspektren - Open Access Massenspektren-Library oder Bibliothek. Das heißt wir stellen / die Leute können eben direkt die Daten runterladen oder sie können auch den Server suchen. Wenn man eine Messung macht, dann hat man am Ende hat man eine Peakliste also im Prinzip deine Masse, dein Massenfragment und die Intensität. Und damit kann man eben in der (platform) suchen und schauen, ob es da vergleichsbare Spektren gibt. Das heißt, man kann darüber auch eine Identifikation deiner (Probe/Spezies?) machen. Kernaufgabe einer Massenspektro-Library.	
Interview 16	00:11:02	Und unsere Idee ist eben, dass es nicht im Computer ist - natprlich ist es im Computer - aber es ist eben ein Online-Angebot und eben nicht eine fest verlinkte Bank, wie es sage ich mal bei Vendor Software ist.	
Interview 16	00:11:21		Welche Vision wird denn mit der (platform) verfolgt?

Interview 16	00:11:25	Die Vision der (platform) ist, dass wir die Identifikation - also ich sage mal, das ist jetzt nicht das von (organisation) - die Vision ist ttscählich, dass wir die Identifikation von Stoffen in Umweltproben oder Proben, die wir bearbeiten, verbessern können. Das heißt, dass es einfacher wird in Zukunft darin zu suchen, und zu sagen ich habe hier eine gute Datengrundlage und ich kann mit einem sehr guten Match Value- das heißt, einem sehr guten Prozentsatz an Singularität, Ähnlichkeit sagen, dass ist der Stoff, den ich in meiner Probe gemssen habe. Und mit dem Match in der (platform) kann ich sagen, dass / kann ich diesen Stoff einfach annotieren dieses Massenspektrum mit meiner Chemikalie.	
Interview 16	00:12:16		Nun handelt meine Arbeit ja von Erfolgsfaktoren. Wie definierst du denn Erfolg bzw. eine erfolgreiche Plattform?
Interview 16	00:12:28	Ich (-) man kann das vielleicht (-) kategorisieren. Das erste wäre natürlich erstmal die Zugriffszahlen, das heißt wie viele Leute greifen überhaupt so zu - das sind so 300 Leute pro Tag.	
Interview 16		Dann ist natürlich eine Awareness, also werden eben Massenspeltren zitiert? Benutzen die Leute das wenn sie eben ihre Publikation schreiben: OK, ich konnte aufgrund von (platform) kann ich was identifizieren und die Leute das auch ordnungsgemäß reinschreiben. Manchmal machen sie das vielleicht nicht, vergessen es. Und das wir dann letztendlich auch sehen in den Publikationszahlen oder Zitaten, ob das jetzt auf Google ist oder ob das irgendwelche Zitationsservices sind. Ich mache da ja meine Auswertung und das ist auch wirklich ansteigend. Das würde ich als Erfolgsfaktor sehen, die Zitationen.	
Interview 16		Und der dritte Bereich ist auch, wie aktiv die Community ist. Wie viele Leute / Es darf im Prinzip auch jeder Spektren hochladen, wir haben da eine gewisse Qualitätskontrolle natürlich. Aber im Prinzip kann da jeder Beitragen und es soll auch jeder beitragen. Das ist so ein Community System.	
Interview 16	00:13:41	Es steht und fällt mit den Submissions, von den Leuten, die sich daran beiteiligen. (unv) sofern man dafür auch Geld acquirieren kann für eine Plattform. Der feste (?) Faktor wäre, dass ich sagen kann, kann ich die Plattform auch irgendwie finanzieren. Ob das hier ein hosting ist bei mir am Institut oder ob ich mal Geld kriege mal jemanden zu bezahlen, der Programmierung macht oder MAssenspektren hochlädt oder soetwas.	
Interview 16	00:14:34	Diese vier Punkte würde ich als Erfolgsfaktoren sehen.Also, was dafür wichtig ist.	
Interview 16	00:14:47		Nun hast du ja schon angesprochen, dass jeder Spektren hochladen kann. Welche Anforderungen stellt ihr denn an die Submission?
Interview 16	00:14:55	Du meint an die Qualität?	
Interview 16	00:15:00		Ja, theoretisch beides: also einmal was man als User für Requirements erfüllen solllte.

Interview 16	00:15:09	Also wir haben halt ein bestimmtes Format. Das heißt, das ist das Record-Format. Da kann ich dir auch nochmal einen Link zu schicken, wenn dich das interessiert. Da haben wir sozusagen / Das ist halt relativ gut definiert. Und wir haben zum Beispiel auch verschiedem Tools geschrieben - es gibt Java basierte Tools, es gibt auch ein R basiertes Tool, mit dem man den Massenspektrum (unv) kann. Und die Voraussetzung ist, dass wir die (unv) bekommen, das heißt, in der Regel ist es nicht so, du hast hier meine Verbidnungsliste und meine Retentionszeiten und meine Massenspektren - prozessiere das mal bitte für mich.	
Interview 16		Das machen wir in der Regel nicht. Sondern wir erwarten schon von den Leuten, dass sie das selber produzieren - prozessieren. Weil wir können eben nicht sagen - unmöglich eben auch mit unseren Tools, weils wir Qualitätssicherungsmaßnahmen auch schon eingebaut haben. Wir haben mit R-Mass, dass ist das (unv) Tool, das macht zum beispiel eine rekallibration der Daten, dass heißt wir rekalibrieren am functional compound werden die Daten dann rekallibiriert.	
Interview 16	00:15:53	DAs ist sozusagen die Akurazität der Daten. Manchmal sind eben die / jenach dem was das für ein Messinstrument ist oder doch die Massen abweichen (unv) manchmal ist das auch ein unterschied, ob ich direkt nach der Kallibrierung messe oder 3,4, 5 Stunden später. Da gibt es dann schon Geräte, die nicht mehr so akkurat sind. Das sind so die Anforderungen: dass es vernünftig prozessiert ist und das eben die Records vorliegen.	
Interview 16	00:17:09	Was natprlich dann die Qualität der Spektren selber anbetrifft, da haben wir selber keine Möglichkeiten. Natürlich kann man schon schauen, welche Massen sind da, welche Fragmente. Auf der anderen Seite ist natprlich auch jede Messung ein bisschen anders und wir haben jetzt aber noch keine Möglichkeiten zu sagen, was weiß ich, über künstliche - artifizielle Fragmentierung bislang zu sagen: ok wir können jedes Massenspektrum simulieren. Wir können sagen, wir können so ein Gerät simulieren. Das können wir eben noch nicht. Aber deswegen müssen wir darauf vertrauen, dass die Leute möglichst beste Qualität da liefern.	
Interview 16	00:18:00		Und welche Regel unterliege ich auf der Plattform? Also wenn ich jetzt Daten hochladen würde, dann muss ich ja die bestimmten Kriterien erfüllen. Dann sage ich mal, Qualitätskontrolle ist dann ja schwierig. Gibt es sonst noch Regeln auf der Plattform für mich als Nutzer?
Interview 16		Wir haben eigentlich keine Regeln. Das hochladen funktioniert so, dass das CIF-Plot, das ist eben letztendlich ein Textformat, das ist eine Textdatein, wo eben über / dieses Schema drauf abgebildet ist. Und wir regeln das alles über GitHub. Das heißt, wir haben den kompletten Datenbestand bei GitHub, da ist das auch öffentlich einsehbar und runterladbar. Der Vorteil ist, dass wir eine Versionskontrolle haben.	

Interview 16	00:18:17	Das heißt, es ist immer nachvollziehbar, was wurde am Record geändert. Zum Beispiel - was weiß ich - wenn jemand sagt die Datenqualität ist schlecht und wir haben das verbessert oder ich habe das doch rekalibriert, dann ist das eben dokumentiert. Und von dort aus wird das dann automatisch hoch geladen. Die Eingangshürrden versuchen wir eigentlich relativ gering zu halten, weil die Leute sonst nicht bereit sind, Daten bereit zu stellen. Wenn wir jetzt sagen würden: OK, ihr müsst noch dieses und jedes tun, dann würden wir nur wenige Leute abholen.	
Interview 16			
Interview 16		Ich meine, es ist ein Kosten-Nutzen Faktor. Nutzen wir die (platform) wirklich so viel, dass ich da was hochladen will? Wenn ich diese Hürden relativ niederig habe, dann wird mehr hochgeladen. Wie ich schon mich schon gesagt habe. Wenn wir unsere Tools nutzen, ist eine gewisse Qualitätskontrolle da, Sofern es möglich ist.	
Interview 16	00:19:29	Aber wir sind natürlich eine Community. Wir sind kein kommerzielles Unternehmen wie (institute), - wie heißt er -. Wir können natürlich niemand bezahlen dafür. Ich habe jetzt keine (Kuratoren?), die tagtäglich ihre Sachen hochladen und sich jedes Spektrum einzeln anschauen. Das können wir natürlich nicht leisten. Weil da wird einfach, da wird ganz viel freiwillige Arbeit drin stecken.	
Interview 16	00:19:48		Gerade hast du ja schon die ganzen Tools angesprochen. Auch die zum Beispiel Suche nach den Spektren. welche Funktionalitäten und Möglichkeiten gibt es denn so gesamt auf der Plattform?
Interview 16		Also, jetzt muss ich die Seite doch mal aufmachen. Also wenn man das eben aufmacht, dann hat man ja vor allem zwei Felder im Prinzip, zwei Tableaus. Das eine ist Suchen. Da kann ich zum Beispiel nach einem Compound suchen oder wenn ich eine Bestimmte Bandbreite habe / in meinem Massenspektrum kann ich nach dieser bestimmten Masse suchen. Und dann gibt es eine Toleranz. Ich kann eine Formel angeben oder eine Formel eintragen.	
Interview 16		Ich kann verschiedene Instrumente angeben. Das ist sozusagen wenn ich schnell suchen will nach einem Compound. Dann kann ich meine Peakliste hochladen. Damit kann ich dann direkt suchen. Ich kann mit einzelnen Peaks suchen. Ich kann aber auch nach anderen chemischen Identifiern suchen. Der Inchi Code ist zum Beispiel der Hashwert, der sozusagen die Struktur beschreibt. Dann kann ich direkt in der Datenbank suchen. Und wenn man den hat, dann kann auch mit dem sogenannten Quell (?) suchen. Der Quell, das ist ein Spektren hash, den haben wir	

Interview 16		Vor zwei Jahren entwickelt. Um einfach jedes Massenspektrum einen Fingerprint zu geben. Das heißt das Ganze Massenspektrum oder diese Peakliste wird dann (unv) verhasht um einfach zu sehen, weil wir unsere Massenspektren ja nicht nur aus der (platform) zu sehen sind sondern es ist irgendwo bei (Unv) es gibt eben andere Massenspektrendatenbaken wie (platform) of north amerika oder spezifische Datenbanken wie Metabolom Database, die speichern unsere Spektren auch ab um einen Vergleich zu hben.	
Interview 16	00:21:15	Und über den Hashwert, wenn man jetzt eine google suche macht, dann würde ich sehen, ich sehe dann ok, ich sehe 50 spektren, aber wenn ich dann denn Vergleich sehe, dann sind das 50 Dublikate von der Plattform. Das ist dann relevanter als wenn ich sagen kann, ok ich habe nur 4 oder 4 hits. Der zweite Teil der suche ist der Record index, da kann man eben direkt suchen nach den Instituten, die was begeitreagen, haben, nach Instrumenten oder auch einzelnen Substanzen. Aber wenn ich nach Substanz suche, ist das eher historisch gewachsen.	
Interview 16	00:23:09		Welche Schwierigkeiten können denn auftreten beim Datenaustausch Über (platform)?
Interview 16		Was meinst du mit Schwierigkeit?	
Interview 16	00:23:24		Beispielsweise dass vielleicht User nicht genau wissen wie sie mit den Daten umgehen sollen, oder Formate, oder alle möglichen Schwierigkeiten?
Interview 16	00:23:43	Es ist natürlich shcon ein Expertensystem. Wir erwarten natürlich schon vom Nutzer, dass er Ahnung und praktische Erfahrung in der Massenspektrometrie hat.	
Interview 16		Ansonsten Ja natürlich wenn ich jetzt eine Suche mache, wenn ich meine Peakliste hochlade und irgendwelche Matches kriege – unser MAtching erfahren ist jetzt noch nicht so ausgereift- da ist noch ziemlich viel in Gange, was da entwickelt wird. Wenn ich jetzt einen Score habe von 80, heißt das noch lange nicht, dass das auch der Wert ist.	
Interview 16	00:23:51	NAtprlich erwarten wir von unseren Nutzern, dass sie es selber bewerten ob das ein richtiger Match ist. Ansonsnte, also ich würde eher sagen, das ist jetzt eher ein Fehlender – von der (platform) würde ich da jetzt keine Schwierigkeiten sagen. Ich würde sagen, dass eher fehlende Ausbildung oder fehlende Erfahrung, da wird jetzt vielleicht mal falsch bewertet. Aber eltztendlich vonn der (platform)seite her sehe ich da jetzt keine.	
Interview 16	00:25:14		Wie schätzt du dann die nach Nutzbarkeit der Daten ein?
Interview 16		Die sehe ich als sehr hoch. Wir betreiben natürlich irgendwie. Wir betreiben natürlich die Plattform so, also alle Spektren sind frei nutzbar. Teilweise sind es sogar schon veröffentlicht und viele, quasi, mit DOI veröffentlicht.	

Interview 16	00:25:44	Das heißt Die Daten können im Prinzip auch in andere Plattformen hochgeladen werden, genutzt werden die. Die Leute können auch theoretisch, wenn es möglich wäre, in anderen Plattformen verwenden. Im Endeffekt können die Hersteller auhch sagen, wir geben das als Give Away oder zur Software dazu, das werden sie nicht machen. Theoretisch können sie das machen, weil das einfach kommerziell nutzbar ist, dadurch dass das auf github ist, ist alles frei verfügbar. Daadurch haben wir eine hohe nachnutzbarkeitsgarantie, und wir machen auch dafür, dass die plattform irgendwann 100% FAIR ist.	
Interview 16			Fair nach den Fair Prinzien?
Interview 16		Ja, Es ist noch nicht 100% implementeriert, aber wir sind da dran. Und wie gesagt die Spektren sind frei verfügbar und werden auch immer mehr als Public Domain angesehen. Ist in Deutschland etwas schweirig, weil es das in Deutschland nicht gibt. Und wir versuchen in zukunft auch mehr das ist jetzt auch eine der nächsten schritte, die geplaant sind, dass die Massendaten auch in einem so genannten GIF Format, das ist ein Format für MAssensektren oder auch in anderen Formaten, also das (unv) Format ist auch geplant.	
Interview 16		Das einfach die verschiedene Leute, die das nutzen wollen, die in ihrer Vendor software oder auch in Richtung (unv) Tools in Life Sciences schreiben, oder machine learning leute und so, dass die das eben besser nutzen können und auch Format wie Jason haben, was die auch eicht verarbeiten können mit den Tools.	
Interview 16	00:28:07		Wie stellt ihr denn sicher, dass die Nutzer der Plattform wissenschaftlich korrekt mit der Datennutzung umgehen.
Interview 16		Nun hab ich ja schon gesagt ich gehe natürlich davon aus dass die Leute Fachanwender sind. Das sie eben wissen, ok, das ist jetzt auch nicht eine Informative Plattform. Und sagen ok, so sieht dichlorfenac aus, das ist ja nicht zugangsbeschränkt. Andererseits kann ich nicht kontrollieren, ob die Leute die richtigen Schlüsse ziehen und tatsächlich auch sagen und auch das richtige Massenspektrum auswählen um die Identifikation richtig zu gestalten.	
Interview 16		Das ist egal ob wir das jetzt sind oder jemand in den Umweltwissenschaften oder Life Sciences, wenn einer einen Stoff annotiert oder Massenspektrum annotiert anhand der (platform), das reicht eben nicht aus um zu sagen, das ist eine volle Identifikation. Und so gibt es eben ein paar Schemen und Ideen. Und da ist ein Library Match ein Teil davon. Man muss eben auch verschiedene Identifikationspunkte haben um ein Stoff letztendlich zu annotieren.	
Interview 16		Da ist letztendlich der Goldstandard, wenn man eben einen Standard kauft, wenn er zur Verfügung steht um ihn zu bestätigen. Insofern sehe ich da jetzt nicht, dass da irgendwie Missbrauch möglich ist. Weil eigentlich das wissenschaftliche System, Kontroll- und Bewertungssystem das nur als einen Punkt ansieht.	

Interview 16	00:28:19	Und man eben auch, Von Peer Revisiern eben erwartet wird, dass ein Stoff richtig identifiziert wird. Von leuten ausserhalb der Wissenschaft, Umweltlabore oder so, klar kann ich nicht bewerten, muss ich auf den Experten vertrauen. Die haben letztendlich auch ihre Qualitätssicherungsmaßanahmen, Sie müssen ja auch garantieren, dass ihre Messungen richtig sind und Identifikationen richtig sind. Und sie werden dann auch ähnliche Instrumente anwenden um zu bestätigen, Wenn ich jetzt / Wenn sie in Schwierigkeiten kommen, wenn ich im Labor schlechte Annotationen machen, werden sie sie nicht mehr beauftragen, weil sie sagen, die Annotation ist schlecht.	
Interview 16	00:31:08		Sind denn dann überwiegend Daten in der (platform) von veröffentlichten Strukturen oder kann man auch die Strukturen hochladen wenn sie noch nicht an eine Pubikation gebunden sind?
Interview 16		Man kann. Also was wir machen / der Großteil sind Standardmesungen. Da werden eben Stoffe aus Standards gemessen. Wir haben als Anzahl - wie viele sind denn das? Moment. (-) Wir haben ungefähr jetzt 76.000 Spektren drin. Und ich sage mal 1000 Spektren sind Spektren, die tentative sind. Die wurden identifiziert in Umweltproben, abe rda gab es zum beisiel noch kein Standard dafür. (unv) WO wir die Stellung einer CH Gruppe noch nicht / das ist eine bestimmte Struktur, wir können aber nicht sagen, dass es das oder das ist.	
Interview 16	00:31:55	Und wir haben auch keine Standards dafür. Das ist ein kleiner Prozentsatz, aber das ist möglich. Und in der Regel wird das auch im Rahmen einer Publikation veröffentlicht und dann sagt man: Ok wir stellen das auch für die (platform) bereit. Aber wie gesagt, dass kann jeder im PRinzip.	
Interview 16	00:33:24	Also dadurch, dass wir das Submission System haben und wir auch durch das Version Controll sehen wir natürlich auch shcon so ein bisschen, was die Leute hochladen. Wir gucken uns das natürlich auch durch. Und wenn da natürlich die ganze Zeit die Sachen drin sind, dann werden wir das auch registrieren und sagen OK, was ist denn das eigenlich oder so. Und mit den Leuten diskutieren, ob das wirklich / Aber wie gesagt, viel geht ja auch so, die Community ist jetzt nicht so groß - die meisten sprechen uns ja auch darauf an und sagen: ich möchte das und das hochladen - kann ich das machen?	
Interview 16	00:34:00	Und dann sagen wir: ja schicke mal rüber. Und dann schauen wir uns das mal an. Also das ist daruch / das ist relativ familiär.	
Interview 16	00:34:09		Nun haben wir ja schon viel über die Nutzer gesprochen. Wie beschreiben Sie denn die Nutzer von (platform)?

Interview 16		Schwer zu sagen, ich kenne sie nicht. Also ich würde mal sagen, es sind im wesentlichen Wissenschaftler im Bereich Life Science, Metabolomics und Umweltwissenschaftler im Bereich der Umweltchemie. Ansonsten weiß ich auch von ein paar Wasserwerken, die (platform) auch relativ viel nutzen. Also die Bundesanstlat für Wetterkunde, die müssen auch Messdaten erstellen - was ist der Trend von Umweltchemikalien in Gewässern?	
Interview 16	00:34:19	Und die Nutzen die (platform) beispielsweise auch um (-) Identifikation zu machen. Also ich würde sagen, der Hauptteil sind Wissenschaftler - und dann würde ich sagen Behörden und dann gibt es sicher auch noch einige kommerzielle Labore, die da auch mal reinschauen. Aber dadurch dass ich / ich mache ja keine / ich kann ja die Leute nicht alle tracken und so. Und manchmal sieht man ja auch das Feedback nicht. Sobald es in den kommerziellen Bereich geht, dann sehe ich das Feedback auch gar nicht mehr über die Publikationen, wie die (platform) genutzt wird.	
Interview 16	00:35:40		Wie bewegt ihr denn Forscher und auch Organisationen die (platform) zu nutzen?
Interview 16	00:35:45	Wir sind eben in dieser (unv (organisation)?) Association, das heißt, wir betreiben die natürlich, diese Community, diese Umweltforschung von (region) wird die ja benutzt. Und das gleiche ist im Prinzip im Metabolmics-Bereich. Also wir versuchen in unseren Communities die anzupreisen und zu nutzen und auch immer wieder in Vorträgen mal vorzustellen. Und das steigt natprlich auch mit den Anzahl der Publikationen oder Vorträgen, die man darüber hält. Im Wesentlichen darüber.	
Interview 16	00:36:28	Dann haben wir / wir twittern ab und zu mal darüber, wenn es mal was neues gibt. Channel ja, aber wir haben jetzt keinen eigenen Account. Das ist aber auch mehr so für Announcements, weil wir haben ganz viele von den Leuten, die das auch nutzen und beitragen, die derzeit eben auch Twitter benutzen als Austauschaplattform. Wäre das eine andere Plattform wie Research Gate, dann wrde man das eher da tun. Aber wie gesagt, die Leute denke ich erreicht man. Man kann auch nicht alle Channel gleichzeitig / also wir sind ja auch keine große, kommerzielle Plattform, genau.	
Interview 16	00:37:18	Dann reden wir mit den Leuten - willst du nicht auch was beitragen oder so. Oder was weiß ich. Teilweise habe ich das auch schon gemacht, ich habe auch Reviews schon geschrieben und dann habe ich Leute gefragt, ob sie (Feedback?) abgeben. Also Sachen hochladen usw. Und wenn man ein Paper liest, dann kann man die Leute schon ansprechen. (unv) Aber wie gesagt, dass ist alles sehr Community getrieben, vom persönlichen Umfeld oder wenn man weiß von einem Umfeld, versuchen wir das zu propagieren.	

Interview 16		Wir sind auch immer wieder im Gespräch mit den Herstellern, dass man die (platform) da besser implimentiert in der Herstellersoftware, was sowohl Nutzbarkeit der Daten anbetriefft und aber auch auf der anderen Seite, dass das auch für die Nutzer einfacher werden soll in der Zukunft. Dass Sie in ihrer Hersteller Software eine Massenspektren Library anlegen, eine eigene. und dass sie diese Sachen auch einfach weiter nach (platform) exportieren können und dadurch auch besser beitragen können.	
Interview 16		Also da arbeiten wir auch, aber die sind immer so ein bisschen (-) naja, die müssen ja auch ihre eigenen Sachen verkaufen. Die wollen / dann haben sie eben Qualitätsbedenken, wo wir aber sagen, naja (unv) ihr preist hier immer eure eigenen Libray systeme an, die haben ja auch eine gute Qualität, aber warum baut ihr nicht einfach für die Nutzer die Möglichkeit ein, diese Massenspektren zu exportieren, dann sind wir aus dem Problem raus und so.	
Interview 16	00:37:56	Thermo (?) zum Beispiel hat ihre eigene Cloud Plattform, die sie betreiben. Wir fangen jetzt schon an mit Edgeländer was zu machen. Da wird in Zukunft etwas passieren, das ist so ein Vendor oder / irgendwann in einer Herstellersoftware auftaucht, das ist dann eine Frage der Zeit, bis die anderen das auch implementeren, weil klar, Konkurrenz schläft nicht.	
Interview 16	00:39:35		Siehst du denn noch weitere Möglichkeiten User zu generieren?
Interview 16	00:39:42	Also jetzt extra / nö, weiß ich nicht, nö. Nee da habe ich jetzt keine - das ist alles, was wir machen können und was in den Zeiten drin ist. Kommt so ein bisschen auch auf die Mund zu Mund Propaganda an. Und gute Publikationen, die damit geschrieben werden. Dass Leute dann sehen: Aha, das hilft bei der Identifikation. (unv)	
Interview 16	00:40:44	Wir sind auch im Gespräch mit großen Chemieplattformen zum Beispiel mit (organisaition) zum Beispiel. Da sind eben auch Massenspektren mit verbandet (?). Es gibt eine Plattform von der USPEA, also der Umweltbehörde, das heißt chemical dashboard. Dort sind unsere Substanzen auch registriert und umgekehrt und solche Sachen. Wir versuchen dann auch diese chemische Domäne eben zu vereinfachen und das eben zu verlinken mit anderen Datenbanken.	
Interview 16	00:41:26	Das heißt die Leute können dann eben, wenn sie in (organisation) sind sagen OK, ich möchte mir ein Massenspektrum anschauen. Dann wird da eben ein Massenspektrum aus der (platform) angezeigt.	
Interview 16	00:41:38		Verwandt mit der Qualitätskontrolle von Daten ist ja auch das Vertrauen. Wie stellt ihr denn das Vertrauen in die Daten sicher?

Interview 16	00:41:48	(lachen) Wie gesagt, wir versuchen - sofern das unsere Möglichkeit ist - diese Daten zu kurieren. Also das wir tatsächlich sagen / oder das auch zu verbessern. Das war früher jetzt sehr stark, bis vor drei, vier oder fünf Jahren, war die Entwicklung von den Leuten in (country) sehr stark getrieben unddann haben wir das quasi übernommen in (region). Die hatten eher so das Paradigma, ok die Leute sind selbst dafür verantworlicht, die Leute die das submitten, dass sie die Qualitätskontrolle erfüllen.Wir sind eher der Meinung, wir müssen eine gewisse Grundkontrolle machen sofern das eben möglich ist.	
Interview 16		Ds heißt zum Beispiel das eben die Metadaten stimmen, dass eben ein Massenspektrum stimmt. Wir gucken zum Beispiel nach den Precursormassen der Formel, die da submitted wird, ob die mit der Formel übereinstimmt. Also Sachen schauen wir schon, lass es einfach Daten sein und das wir die Vergleiche eben raussuchen können.	
Interview 16	00:42:44	Die Fragmentierung können wir eben noch nicht so schnell und so gut hervorsagen, dass wir sagen können, dieses Massenspektrum hat irgendwo ein Problem oder da lief etwas schief oder irgendwas. Dann bräuchte man auch diese Libraries auch nicht mehr, wenn wir das mal können. Wenn wir das In-Silico machen können.	
Interview 16		Was die / von daher habe ich, glaube ich shcon, dass die Leute Vertrauen haben können. Das Vertrauen in die Qualität. Natürlich wird es auch Leute geben, die nicht so gut kallibrierte Daten Submission machen - da habe ich natürlich mehr falsch positives, falsch negative Matches, weil eben die Massenabweichungen irgendwann nicht mehr stimmen. Das kann schon sein, aber das ist schon etwas, was wir zur Zeit eben nicht händeln können oder nur in einem gewissen Umfang.	
Interview 16		Weil wir sozusagen sagen zu den Leuten: nimt bitte unsere Software um das zu generieren. Das ist ein Bereich, wo wir uns verbessern können. Also ich würde nicht sagen, dass man dem nicht vertrauen kann, aber ich sage auch Ok ich würde dem nicht blind vertrauen. Aber das würde ich auch bei einer Vendors Library nicht machen, auch einer hochqualitativen, die von drei Chemikern oder Massenspektrometrikern irgendwie kuriert wird.	
Interview 16	00:43:36	Der würde ich auch nicht blind vertrauen. Das kommt immer auf dein eigenes Expertenwissen an und ich meine, die Leute sind Chemiker, die davor sitzen und die müssen das selbst bewerten. Das ist letzlich nur eine Entscheidungshilfe - ein tool.	
Interview 16	00:45:00		Um jetzt mal auf ein ganz anderes Thema zu kommen: Wie ist der organisatorische Aufbau hinter (platform)?
Interview 16		Wir sind im Prinzip eine Community, die eigentlich frei organisiert ist. Wir haben zur Zeit zwei größere Sponsoren. Das ist einmal eben (organisation). Das ist das UFZ, das eben das ganze Hosting anbetrifft. Und das ist das (institute) in (town) - das ist das (insitute), ja genau.	

Interview 16	00:45:15	Wir tragen eben noch ein Teil davon. Und es gibt eine - wie heißt das - (name), das ist so ein BMBF Forschungsverbund, die finanzieren eben auch einen Teil davon. Ansonsten sage ich mal, wir sind eigentlich eine kooperative Community. Das heißt, wir haben im Prinzip keinen Chef. Naja, sagen wir mal, dass ist XY, das ist (name), das bin ich, die das so ein bisschen leiten.	
Interview 16	00:46:10	Und schauen, dass das alles so / das die Issues abgearbeitet werden, dass wir die Kontakte aufrecht erhalten. Aber das ist alles, das ist eine echte Community, da gibt es keine wirkliche hierachische Struktur oder so sondern die Themen werden eben diskutiert und wir können Issues schreiben auf GibtHub und so weiter. Das ist eben eine sehr (-) wirkliche Community.	
Interview 16	00:46:45		Das heißt, wie viele Arbeiten dann für die (platform)?
Interview 16	00:46:51	naja sagen wir mal es arbeiten / wir haben einen halben programmierer, der vom (instiute) bezahlt wird, wir haben noch einen Hiwi und - die stelle ist noch unbesetzt, aber wir hätten Geld dafür - und das sind drei, vier Leute, die das im Kern betreuen und eben auch Software dafür schreiben.Aber das ist echt eine Community-Leistung. Wir haben andere Jobs. Das ist auch nicht unsere Hauptaufgabe, nicht unsere Jobs, sondern das ist irgendwo - nicht unser Hobby, aber wir werden ja nicht dafür bezahlt, sondern das sind Stunden, wie wir irgendwo anders abknapsen um uns darum zu kümmern. Also das ist wirklich sehr sehr non-for Profit (unv)	
Interview 16	00:47:45		Und die, die jetzt involviert sind in die (platform) - was für Kompetenzen haben die?
Interview 16	00:47:50	Also wir haben eben zwei sehr gute Programmierer, das sind (name) und (name), das ist der vierte im Bunde. Dann eben Leute, die mehr im Bereich Massenspektrometrie unterwegs sind, das sind eben (name), (name), (name) und ich. Und der Hauptpart von (name) ist eben die Chemoinformatics bei den ganzen Sachen. Also die Kontakte (unv) und alle einzubeziehen. Und (name) eben auch computational Massspektrometrie. Ich betreibe hier den Server und kann von allem ein bisschen. Und genau, das sind so die Kompetenzen.	
Interview 16	00:48:40		Wenn jetzt irgendwelche Entscheidungen in Bezug auf die (platform) getroffen werden, wie findet das statt?
Interview 16	00:48:50	Naja, wir diskutieren das eben, das wird dann - was weiß ich - eben ein Issue aufgemacht auf Github oder irgendjemand bringt dann ein Thema auf und das wird dann per Email entschieden und diskutiert was wir da jetzt machen. Und dann sehen wir halt zu, dass dann ein Issue geschrieben wird und das dann implementiert wird. Es gibt natprlich auch Dinge - wir haben ja auch alle Projekte und so weiter - da kommen auch Themen auf, wo wir sagen, OK das wird dann halt implementiert und gemacht, da wird dann auch nicht jedes mal heder und alle gefragt oder deren Meinung. Sagen wir mal, das ist ein sehr offener Prozess.	

Interview 16	00:49:35		Gerade hast du ja schon von der Finanzierung mit (organisation), (institute)und so weiter gesprochen. Ist das dann eher projektbasierte Finanzierung oder langfristige Zusicherung?
Interview 16		Also derzeit ist das eher eine projektbasierte Finanzierung. Alsowir haben jetzt dieses (institute). Da gibt es ab und zu ein bisschen Geld von (platform), von (organisation). Wie gesagt, das (institute)stellt kostenlos die Plattform zur Verfügung. Das ist schon entschiede worauf der SErver läuft, die Anforderungen sind da nicht so hoch an das System und auch die Betreuung. Ich betreue den Server hauptsächlich, aber ich habe natprlich einen IT menschen im Hintergrund, der mir die virtuellen Machinen verwaltet, damit ich weiter komme. Das ist dann projektbasiert bzw. dann Grundfinanziert.	
Interview 16		Wir sind jetzt dabei, das i´n eine längerfristige Finanzierung über zu führen, also die (platform) wird - ich weiß nicht ob du davon gehört hast, es gibt diese NFDI, also die nationalen Forschungsdateninrastrukturen. Das ist eben im Grunde ein großes Projekt, Konsortium, oder projektbereich, der von der DFG finanziert wird. Und wir haben jetzt eben ein Projekt, wo ich auch beteiligt bin, das heißt NFDI4Chem.	
Interview 16	00:49:50	Dort wird eben die ganze Forschungsdateninfrastruktur für Chemie allgemein verbessert werden. Und da wird eben (platform) auch ganz stark implementiert werden als Datenbank für Massenspektrometrie. Und das ist dann auch etwas, wo wir hoffen, dass das dann in Dauerförderung oder irgendwie dann übergeführt wird. Oder das da dann irgendwie ein Topf dafür ist, dass das dann auch dauerhaft - jetzt vielleicht nicht jemanden dafür anzustellen - oder vielleicht auch dauerhaft jemanden zu haben, der das auf Dauer betreut, der eben das auf Dauer auch programmiert, weiter entwickelt.	
Interview 16		Aber das ist zur Zeit eher noch projektbezogen. Aauch für NFDI ist das jetzt für fünf Jahre. Ende diesen Jahres fängt das dann an. Da weiß ich dann in vier oder fünf Jahren mehr, ob sich das als Daueraufgabe implementiert hat oder nicht. Aber wie gesagt, die Nutzung wird ansteigen.	
Interview 16	00:51:51	Das heißt auch, es gibt mehr Bereitschaft so etwas zu fördern. Auf der anderen Seite sehe ich die Bereitschaft in die Datenbanken, die gefördert werden, ist meistens nicht so hoch. Zur Zeit macht das halt jemand, weil er es in seiner Doktorarbeit irgendwie macht oder in Projekten. Und da weiß man, dass derjenige das irgendwann nicht mehr betreut. Ich hoffe, dass das mit (platform) nicht passiert. Ich bin auch ziemlich überzeugt, dass das mit (platform) nicht passieren wird.	
Interview 16	00:52:45		Werfolgt ihr denn irgendwelche konkreten Ziele, die ihr mit (platform) erreichen wollt?

Interview 16		Naja wir wollen / unsere derzeitigen Ziele sind soweit, das API verbessern - wir können das noch besser. Das nächste Ziel ist es FAIR zu sein - erreichbarkeit und diese Dinge. Da scheitert es bei uns gerade noch beim API, das wird jetzt gerade verbessert. Wir sind auch im Kontakt mit anderen Plattformen um einfach zu sagen - wir wollen ja uch nicht ins blaue reinprogrammieren und sagen, wir machen nur das was, da ist.	
Interview 16	00:52:55	Dafür ist unsere Zeit auch zu kostbar. Wir sind derzeitig auch sehr stark mit den Schwesternplattformen in Kontakt um zu schauen, was brauchen die eigentlich um unsere Daten suchen zu können, um unsere Plattform leichter einbinden zu können oder suchen, wenn sie (hintergrund?) bei uns machen. Und die ganzen Dinge. Das ist eigentlich das nächste Ziel. Und das mittlere Ziel ist, dass wir wirkich FAIR 100% umsetzten. Aber ich denke, da sind wir auf einem guten Weg. Und dann mittelfristig die Implementierung in NFDI, in die NFDI (-) Plattform.	
Interview 16	00:54:08		Und wenn ihr euch die Ziele steckt, wie kontrolliert ihr dann die Erreichung der Ziele?
Interview 16	00:54:23	(lachen) du meinst im Sinne von Projektmanagement? Naja das wird eben / ich meine die Fair Prinzips implementierung wird eben (-) ist halt da, das wollen wir halt erreichen. Wir haben jetzt nicht irgendwelche Milestones: bis dann und dann das machen. Das haben wir tatsächlich nicht. Und es wird teilweise eben auch durch die beiden Projekte, (institute) und dann auch NFDI, wird das dann da auch mit implementiert. Das heißt, da wird es dann natürlich irgendwelche Milestones oder irgendwas geben. Oder Ziele, die innerhalb des Projekts erreicht werden sollen. Und das werden wir dann natürlich auch in (platform) implementerien.	
Interview 16	00:55:15		Und in Zukunft wie sieht das dann aus - wollt ihr die Comunityarbeit intensivieren oder eher vergrößern mit der (platform)?
Interview 16		Wir wollen die Community natürlich auch vergrößern. Iund deswegen arbeiten wir auch daran, dass es für die Leute auch einfacher wird ihre Daten an die (platform) zu übermitteln - wenn es in meiner Herstellersoftware drin ist oder im Bereich der NFDI ist geplant, da soll es ein elektronschies Labbook geben, also speziell für die Chemie und die Anwendungen.	
Interview 16	00:05:29	Und dort soll es eben dann auch möglich sein, dass man direkt aus seinem Labbook die Massenspektren letzlich hoch lädt in (platform). Und das vereinfacht das natürlich für die LEute. Wenn ich eh schon alles zusammen habe, dann sind das auch nur noch drei, vier Mausklickst, direkt (unv) hochzuladen.	

Interview 16	00:56:17	Das heißt, wir wollen natürlich die Community vergrößern. Und die Intensivierung innerhalb der Community (-) denke, natprlich will man immer mehr Leute dabei haben auf der einen Seite, die das können, auf der anderen Seite snd die Leute, die da auch wirklich Fähigkeiten haben was dazu bei zu tragen, wirklich sehr rar. Und sind oft auch mit ihrere eigenen Forschungsarbeit, beispielsweise im Metabolomicsbereich beschäftigt oder sie arbeiten selber auch and anderen Tools und haben auch nicht so die Zeit so die Serverplattform mitzubetreiben.	
Interview 16		Es ist natprlich wünschenswert, wenn man aber auf Dauer eine Finanzierung findet, wo man sagt, man kann auch Leute dafür bezahlen, dann sehe ich das eher als Schwierig an. Sondern es wird eher an 5,6 Leuten hängen bleiben. Bislang, die eh schon involviert sind.	
Interview 16		Die Leute aus (country) wollen wieder einsteigen, aber das werden wir sehen, wie das funktioniert. Da ist die Kommunikation ein bisschen schwierig. Einfach wegen der Sprachbarriere. DA wird der alte Entwickler wieder eingestellt, aber der spricht halt kein englisch. Er versteht sehr gut, er kann alles lesen, aber er kommuniziert quasi nicht auf englisch und das ist dann immer etwas schwierig, wenn da niemand (unv) zu ihm sprechen kann.	
Interview 16	00:57:27	Werden wir sehen. Ich denke, bei der Community, die Größe wird bleiben. Bei der Submission Community, Nutzer oder auch andere hoffe ich, dass das noch viel mehr wird.	
Interview 16	00:58:17		Apropos Schwierigkeiten. Welche Schwierigketein und Herausforderungen gibt es denn noch beim Management der Plattform?
Interview 16	00:58:28	Wir haben keine Probleme, was das supporten anbetrifft. Ich sehe da jetzt keine Schwierigkeiten. Von der technischen Seite (…) von der Communication (-) naja es ist so ein bisschen die Communication mit den Herstellern. Das sind sehr lanwierige Prozesse, aber das hängt auch damit zusammen, dass in Firmen die Entscheidungsprozesse sehr langwierig sind. Ansonsten sehe ich da aber keine weiteren Schwierigkeiten.	
Interview 16	00:59:20		Gut, dann kann ich jetzt zu meinen letzten Fragen kommen. (unwichtiger Part dass eine Stunde um ist und ob er lönger Zeit hat.
Interview 16			Eine Besonderheit von (platform) ist ja auch, dass es innerhalb der wissenschaftlichen Community ist. Wie schätzt du den Einfluss der wissenschafltichen Kultur auf die Nutzung von (platform) ein?

Interview 16	00:59:51	(---) Das ist natürlich, bei solchen Plattformen, die steigen und fallen natürlich a) mit der Nutzung der Leute und b) eben auch durch die Bereitstellung eigener Daten. Eine Plattform, die nicht durch die Community / also so eine freie Plattform oder Community Plattform, die von der Community, den Wissenschaftlern befüllt werden, oder die eben dann nicht zitiert wird, obwohl sie benutzt wird usw, die wird auf Dauer marginalisiert werden.und natürlich / von daher ist die Community schon wichtig. Beantwortet das deine Frage oder meinst du noch was anderres?	
Interview 16	01:00:47		Also die Frage ist tatsächlich komplett offen gestellt- was auch immer dir dazu einfällt.
Interview 16		(---)	
Interview 16	01:01:02		Ansonsten mache ich auch einfach mit der nächsten Frage weiter.
Interview 16	01:01:05	Ja, mach mal.	
Interview 16	01:01:06		Welche Entwicklung siehst du denn für (platform) für die nächsten zehn Jahre?
Interview 16		Wie gesagt, in den nächsten zehn Jahren hängt es davon ab, ob wir eine Finanzierung finden, das in NFDI4Chem implementieren als Basisplattform, dann das wir eine bessere Vernetzung haben mit allen anderen Plattformen, dass wir wirklich aufFAIR Prinzipien umstellen, umsetzten komplett, und das wir auch - ich hoffe zumindest, dass wir in einigen Vendors Plattformen auch anerkannt sind.	
Interview 16	01:01:10	Dass es dann möglich ist, in der (platform) direkt zu suchen oder die Records zu durchsuchen, die man dann vielleicht lokal hält und dass es auch einfacher wird für die Nutzung kommerzieller Software Massenspektren beizutragen.	
Interview 16	01:02:22		Und wovon hängt der Erfolg der Plattform dann ab?
Interview 16	01:02:25	Ich sehe da immer noch die Nutzung. Ich meine die soll natürlich zitiert werden, die soll genutzt werden. Wenn es keiner mehr nutzt, dann kann ich sie auch irgendwann abschalten weil ich - was weiß ich - wenn die Nutzerzahlen auf zehn gehen über ein Jahr weg, dann kann ich auch sagen, nagut das hat sich wohl irgendwie tot gelaufen, das braucht auch keiner mehr, das kann ich dann auch abschalten. Es bleibt natprlich weiterhin dieses: haben wir erfolg bei den Zitationen, haben wir erfolg bei der Vernetzung mit anderen Plattformen, haben wir Erfolg, werden wir zitiert, tragen die Leute was bei usw. Das ändert sich glaube ich nie.	
Interview 16	01:03:18		Sehr schön. Hast du denn sonst noch etwas, was dir zu Erfolgsfaktoren einfällt, zu Herausforderungen oder was auch immer dir noch auf der Zunge liegt?
Interview 16		Es muss eben / Plattformen dürfen eben kein Selbstzweck sein. Ich kann nicht nur eine Plattform machen, weil ich irgendwie Lust habe. Jetzt sozusagen, also wir hatten schon mal den Fall in einem WO(?) Projekt, wo es nur darum ging - also das tool hieß Rifgepedia. Und das sollte sozusagen eine Plattform werden, so Wikipediamäßig, was die Leute dann eben befüllen können mit Risikofaktoren zum Beispiel für die aquatische Umwelt.	

Interview 16		Was weiß ich, irgendwelche Dinge. Das Ding wurde dann irgendwann abgestellt, weil auch die Hersteller, Betreiber - die das eben erfunden haben - nicht bereit waren, da selbst beizutragen. Da ging es wirklich nur um den Selbstzweck, ich erstelle diese Plattform und ich muss schon auch den Leuten zeigen als Betreiber einer Plattform, dass ich die auch selbst nutze. Weil ich dadurch auch am Glaubwürdigsten es wird, dass es eben nicht um einen selbstzeck geht.	
Interview 16	01:03:34	Oder die Erfüllung irgendwelcher Projekte, Projektwünsche um irgendwelche Mittel für irgendetwas anderes zu acquieren, sondern dass ich da auch wirklich eigeninteresse drin habe. Das zeige ich auch darüber, dass ich es selber benutze, ein offenes Gehör habe für meine Community und dass ich selber damit auch coole Science damit mache. und meine eigene Arbeitsgruppe oder so dann auch Publikationen hat, wo man sagt: ok, da hat das (platform) geholfen. Dass ich also sage, ok wir konnten den Stoff identifizieren, weil ich eben in der (platform) zu der Struktur gefunden habe zum Beispiel. Und das gilt jetzt für andere Plattformen auch.	
Interview 16	01:05:21	Wenn ich es nur technologisch betreibe, habe ich glaube ich keinen Erfolg, sondern ich muss sozusagen auch wirklich auch zeigen, OK, das ist wirklich was, was mich selbst interessiert, was ich selbst benutze und was ich auch gerne nicht nur technisch präsentiere, sondern damit letzendlich auch gute Stories generieren kann aus meiner Forschung heraus.	
Interview 16	01:05:52		Ja gut, vielen Dank
Interview 16		Ja, bitte.	
Interview 17	Time	Interviewee 17	Interviewer
Interview 17	3:57		what is the core offering (platform)?
Interview 17	4:02	so (platform) is a collection of protein sequences and the annotation upon those sequences. And the database is really divided into two sections. One section is the part that's curated by experts from the literature. And there's about half a million sequences. And then there are another hundred and 90 million sequences in the other section which we call (platform). And those have only automatic annotation upon them. So but over time, as as proteins are experiment to characterize this slowly things move from the (platform) section into the (platform) section.	
Interview 17	4:50		When I look at the website, there's also (name), (name) and (name). So these are also part of the (platform)?
Interview 17		Yeah, so that they're all part of it. So (name) gives a sort of a slice of view of the data, which is useful for sequence similarity searching gives you a kind of a non redundant view, (name) is it's kind of like a back end thing, people, people, only the experts kind of use it really.	
Interview 17	5:00	And it just has every sequence that we've ever seen or know within it. That has very little metadata attached to those sequences. And you mentioned (name). So we also have Yeah, as sort of a proteome or genome slice of the data as well, to just to organize it and some people have sort of been vested in viewing the data in that kind of, but	

Interview 17	5:56		my work is now about success factors. My question to you is, what is success or a successful platform?
Interview 17		Okay, let's have a think. That's good. Good question. So, for me, I think a good success measure is twofold. One, that you're doing something important and useful. And the second part is that it's widely used by people. And so, you know, there are many data resources and platforms and things out there, which are just not do it.	
Interview 17	6:09	They're not doing something that's important or useful. Oddly enough, that you know, the people that they can be quite specific and niche sort of things, whereas something like (platform), it's very general. It attempts to look at all natural proteins. So as long as the data and The content and the way it's presented is good then it should be very, very widely useful to people.	
Interview 17	06:35	So yeah, success factors well, there are so many of them, so of course it's important to have good people, good funding. You need stability and support and (platform) like it has institutional support. Which is, you know, really critical as well.	
Interview 17	7:39	I need to write start writing this down so I can organize my thoughts. There's so much to it. Okay, is there anything that you particularly want to focus on from from those while I'm gathering my thoughts a bit more.	
Interview 17	7:55		For me, everything is important, especially when it comes to management and organizational part behind it also.
Interview 17	8:05	Yeah, absolutely.	
Interview 17	8:16	Yeah, and a unique problem the management is important because it's a an international consortium. So, you know, it's very important that we coordinate our work as well and so we have regular meetings and so on to do that and project plan for my grandson.	
Interview 17	8:44	But I'm just trying to think if my order, how would I do that?	
Interview 17	8:56	Okay, there's a complete a very long essay to be written here. who's writing it of course.	
Interview 17		And, and I guess data quality is, is really critical here as well. So one can be working on the most important problem in the world. But if if the data is not organized well and put into, you know, ontologies and made machine readable, then then it's not going to be useful for people. And I guess so, as well as quality, there's kind of coverage as well, which is important with these things. So, people if, if you if you do 10% of the job really well.	
Interview 17		Well, actually, it's better to do 90% of the job less well than that. So this coverage idea of coverage is quite important because You know, if you've got the audience coming sort of randomly to look about their work, if they can't find anything relevant, then they'll leave and they'll never come back again. So something maybe not for unique profit, something I learned from another database that I've worked on p fan.	

Interview 17	9:06	And the reason for its its success was much down to coverage over quality, surprisingly. And we had many long discussions about whether we should focus on on very high quality versus not so high quality but much more of it. With (platform). We, we firmly hold on to quality and and I think that is at the cost of some coverage. And there are certain areas of biology that we cover less well	
Interview 17	10:57	to the detriment of those audiences.	
Interview 17	11:02	And that coverage is kind of governed by the importance of the area and the funding available. So for example, human proteins we hot on human proteins, we try and cover everything. If, if it's dictyostelium discord and you know, slime molds, wonderful, but it's, you know, economically much less important and a relatively minor model organism, so it gets much less attention.	
Interview 17	11:41	Okay, so then funding, I think funding has been a kind of a big issue. So (platform) been largely successful with keeping and maintaining funding. Over the years,	
Interview 17	12:02	but there have been problems. So sort of (year) years ago, the (country) funding was cut. And this caused enormous problems for the database.	
Interview 17	12:21		So how is it How is (platform) funded now?
Interview 17	12:26	So (platform) is funded by three major funders, which is the National Institutes of Health in the (region), in the (region). The (region) molecular biology laboratory, which is funded by the 26 embel member states, and in(country) by the (insitiute). And very in broad brushstrokes, you can say that a third of the funding roughly comes from each of those sources. And, yeah, that that funds the work of about 80 staff around the world, and based in(country), (country) and (country).	
Interview 17	13:15		Great. So then we can keep on with your other points of your list.
Interview 17	13:22	Yes. So, um, I mean that I think there's there is more to say about funding and just how important that is for the success of a platform. You know, as soon as the funding goes away, then it's very difficult to maintain and run the platform. Having for (platform) having the funding split across three sites and three funders, I think gives some resilience. I mean, it would still be a disaster if one funder Institute or something cut funding stopped operating. But it means that that, that that would continue the database platform would continue.	
Interview 17	14:11	And having that institutional support, you know, through the (region) bioinformatics Institute and the (Insitute) and so on. That means that, you know, the key, the really key staff tend to be funded off of this core institutional funding. So, you know, even if there were problems short term with funding, then the really key staff generally would continue.	
Interview 17	14:43	And, and these things, I think, yeah, over the long over the long term, are important for success. So that the stuff that we have Everything relies on having good, well trained staff.	

Interview 17		And that can be an issue. So recruitment at various times through the years has been difficult, particularly for software developers. And we're in a phase now where data science and you know, artificial intelligence and these sorts of skills that actually we want and value in unipro are actually wanted by all sorts of industries. And so we are trying to compete with them to get good software developers. And so that's one of the challenges that we have at the moment.	
Interview 17		Let's see. So we've talked about Management Science, funding people and communication is also important. Talk to your audience making the mobile era of your work. And we have a variety of channels that we do that from going to conferences and talking through social media channels, and we write regular articles that go into the nucleic acid research database issue.	
Interview 17	15:05	And and I think when people are looking at, you know, finding papers describing data platforms, biological databases, then that's probably the first place that they will go to find out information and the paper to site when they using the word. So yeah, we write one of those papers every two years, and that gives us an opportunity to explain the new features and things that we've developed in one place.	
Interview 17	16:56		Great. This was very insightful. Do you have more aspect about this?
Interview 17	17:03	Why should I about success factors? That was the original question?	
Interview 17		Um, okay, so there are some other success factors. And I'm drawing on all my different experiences of biological databases and so on. So actually being early to the field, I think is quite an important success factor. You know, there's often a bit of a competitive advantage if you're the first to the market.	
Interview 17	17:12	And I think you definitely see that in biological databases, that the early ones, they get them the market share and brand awareness and people will sort of tend to stick with the thing that they know even if It's actually maybe not the best one out there. I'm not saying that for unit at all, actually, I think (platform) is the major resource and there isn't very much sort of competition actually in the field. Over the years that the sort of things have merged, so there was a database called (platform).	
Interview 17	18:21	And that merged with (platform), and (platform) back in (year/number), to form (platform). So that's sort of, you're thinking about economic models, and, and so on. So, you know, you get this innovation phase where you get multiple things databases being generated, which do more or less the same thing. And then, you know, there's consolidation. We see this in pharmaceutical companies and so on. Fewer and fewer and, and so it can be with biological databases to actually in biological databases, you don't see takeovers so often or merges mostly One of them wins out continues to get funded and the others sort of wither away and don't grow.	
Interview 17	19:11		Now, you mentioned a part of the history of (platform). So can you tell me a little bit more about the history?

Interview 17	19:22	Yeah, sure. So the the original database, I guess that we consider the seed of this was (platform), and that was founded about 30 years ago. I am also a rock.	
Interview 17	19:36	And then after about a year, he	
Interview 17		started collaborating with the(platform). And so they started to curate entries and in fact, the first person to work on that was (name), who then became sort of poi for the unit. Along with animals and epi, they develop this sort of the (platform) resources is sort of automated supplement to the hand curated entries and (platform).	
Interview 17	19:40	Then I should know this, but I can't remember the year but around the sort of turn of the millennium, then (platform) suffered a funding crisis. And in fact it commercialized for a couple of years, and so was licensed industry and that was how it was paid for. And then NIH stepped in and started to fund it. And that was the beginning of the creation of the (platform) database, which incorporated PR which was run from University of (town) in (country). (name) joins us the third sort of poi for the for the consortium.	
Interview 17	21:00	Then since that time it's it's continued to develop and so the leadership has changed. So now if the original three only Kathy woo is still a p i so I'm the poi here it (instiute) an (name) is the API in (town). The (insitutite) instituted by informatics so that's the plotted kind of history. I think there's a lot more detail that but i i'm not sure you need eat all of that.	
Interview 17	21:38	But yeah, not a straight history, I would say.	
Interview 17	21:42		No, what, what a vision Are you pursuing with (platform)?
Interview 17	21:50	So vision, so I mean, the vision	
Interview 17		state, quite constant, I think is to, you know, provide it high quality, comprehensive and freely available resource for the scientific community for them to understand the function of proteins, you know that that the efficient the focus has changed over time and that the community has sort of changed how it does its science and so on and we've had to adapt to that. with NIH funding, I think our focuses in creased, we have more of a focus on human and disease aspects and so on. Let's call it a biomedical focus. I think that's that's increased significantly in the last few years.	
Interview 17		Within delivering that, I think there are lots of exciting things that we sort of keep a watch on. So one thing is just the sheer amount of secret And how do we scale with that sequence. And so that's been a big focus for us to make the data manageable. You know, if if we, if we gave people every possible sequence that we know about that the data would just be unmanageable. We would probably have 500 million proteins in the database rather than 200 million.	
Interview 17		And in fact, we're, we're kind of excited about the new set of sequences that will be coming from the tree of life projects. So these are projects that will be sequencing essentially every animal and plant species in various countries. And also, there's environmental sequencing called meta genomics.	

Interview 17	21:54	And high throughput sequencing has got so deep now, that just if you assemble meta genomics DNA data Complete genomes Fallout, mainly bacterial. And so this gives us another source of data. And if we look at that data, then there are literally billions of novel sequences that we don't have a unique problem. So we have a major data challenge in the coming five or so years to make this available in a way that's useful to our users. And doesn't break everyone's pipelines because too much data.	
Interview 17	24:33		You mentioned that there are three institutions involved and they're around 80 people working for (platform). How is the organizational structure behind it?
Interview 17	24:48	So actually, it's three countries and four institutions. So in the US, or Kathy who has joint appointments at the unit, (town) University in Washington, And also at the University of Delaware. And so, in terms of the sort of management organizational structure, so we have the the PIs so myself, Kathy and Alan.	
Interview 17		And we then have a next layer of staff that we call the key staff, which is 11 sort of senior group project leaders who who really manage the day to day delivery of the projects. We have regular teleconferences. So we have a kind of a, I'd call it a strategy teleconference which is the P is in the key staff.	
Interview 17		We also have a monthly poi teleconference as well. And then there's also When operations teleconference and that the PIs actually don't attend that one. That's the key staff that people who get on and do the sort of the operational stuff. And then we have two consortium meetings each year, where we rotate around the different countries and we have a face to face meeting. And at those, we go through all the project plans and milestones and discuss future future plans and so on.	
Interview 17	25:16	So we, in terms of the management, we work by consensus, I mean, we do have a mechanism for voting between with three votes for the three p eyes if something becomes contentious, but some that's very rarely ever used. What else can I tell you? So we have a dedicated project manager, staff person who helps organize things and keep us in line and delivering on the various projects.	
Interview 17	27:16		You mentioned the milestones now, so I wondering, what specific goals are you setting?
Interview 17	27:24	Hmm? Oh, I haven't got that.	
Interview 17		So, we have a variety of things on there. So there are some, which are, you know, just ongoing milestones, which is deliver monthly releases for the database, and then some more specific development plans. So we have, we're developing a new website. And so there are lots of sort of phased milestones there about delivering parts of the front end, back end and so on. We also have Have more biologically focused milestones which might be to no focus annotation in a specific area of biology.	

Interview 17	27:28	We, we have ones to integrate certain types of data into the resource. So so the granularity of the milestones is relatively large. You know, these are things that happen on a sort of three to six month timeframe rather than a sort of a week or two weeks or something. Those those big milestones around the slides get split up and into their sub parts that get worked on by the software developers and curators themselves.	
Interview 17	28:50		And how do you control your goal achievement?
Interview 17	28:57	I'm so Well, I mean, at the very top level, then is is going through the milestones. And but yeah, the each of those, as I've said that quite the granularity is quite large. So then it, it goes down to the key staff who, who are really the operators and their teams who are delivering on those. And then within those teams, generally, they're using a kind of an agile process. So they have sprints and Scrum masters, and all of those sorts of things. I don't get into that level of management. So that's delegated to them to to deal with the Manage.	
Interview 17	29:44		Which brings me to the next question, and how are the different responsibilities distributed?
Interview 17	29:56	So we have Yeah, across across three sites, so I mean, it's a variety. It's, it's really a variety. So we have some, some of the milestones are very clearly something that one side or the other is working on. And so then the ultimate responsibility would be with the sipi. But in actual fact it always, it's delegated down to the key staff members. Sometimes they're a joint sort of activities that run across across the sites and generally one of the key staff will step up to take responsibility and reports on all that activity.	
Interview 17	30:47		I have one question regarding the employees and the key stuff. What kind of competences do they have?
Interview 17	30:58	The stuff or the key stuff	
Interview 17	31:01	All of them. Okay.	
Interview 17	31:05	So it's,	
Interview 17		I guess that there were probably two major classes of people that we have. Now, let's say three. So there's the straight software developers. And these are people who are who are really developing code or maintaining databases. And they often, but not always come from, you know, an industry background. You know, they may have relatively little domain knowledge in the area of biology, sometimes none at all.	
Interview 17		And then we have the curators who are PhD level biologists, who are the people that read the literature and extract information and organize that Generally they are not they don't have coding experience. Some of them do. But not mainly, I think they do not. But they're using software tools developed by other people all the time so that they're very computer savvy, but they're not coding. And then we have this sort of third class, which sit in the middle, which are called bio informaticians.	

Interview 17		And they are people that have domain knowledge. So they're generally biologists, but they also have more coding skills. And so they, for example, they might do prototyping work to understand if, you know, a particular approach is going to work, how it might deliver. And then once they've done that prototyping, then the actual heavy lifting and coding would get handed off to a software developer, for example, and the bioinformaticians would be doing you know phrase quality control.	
Interview 17	31:07	Helping the curators by automating processes that they that would be helpful to them to be more productive. And of course, we have sort of other people who are managers of one sort or another, but generally those managers have been promoted up through the ranks and would fall into one of those categories.	
Interview 17	33:25		You mentioned those regulary strategic meetings, have you set up and strategy, how you want to position (platform) on the market, a data market.
Interview 17	33:43	So we,	
Interview 17		when we write our we have a major grant from NIH, we sort of we're always thinking about the positioning there and also to the SIP and epi, we have to present Every four or five years to our respective in Institute reviews. And so it's in those times that we're really thinking about the strategic positioning and how we fit in the international context and so on.	
Interview 17		And we're really helped in that by our scientific advisory board that meets once a year. And so we have a dozen senior scientists from various fields who, who come and advise us and this is one of the areas that, that Yeah, we get good advice from them about, you know, with data science or artificial intelligence, how we positioning ourselves and are we doing, you know, doing doing the right things there or no, in structural biology, there's a lot going on and changing that.	
Interview 17	33:47	So, you know, all these different fields and new areas that may be opening up that Yeah, we were watching and migrating all sort of strategic vision?	
Interview 17	35:09		So do you want to broaden your scope? Or do you want to dive deeper into the existing community?
Interview 17		So I think that one of the major areas that we foresee that we need to broaden into is the medical field. And so there are very many different clinical communities that that will be using this because, you know, genomics is moving into the clinic and becoming more and more popular and so we need to work out how, how we make information about mutations and disease more available and available in a kind of an easy to understand manner.	
Interview 17		uni pots never going to be the place where you go to sort of do a diagnosis of those sorts of things. But, you know, there are various clinical academics who definitely could use (platform). But the problem is that the clinicians are not one community. It's an enormous, enormous set of different complex overlapping communities.	

Interview 17	35:18	And it's, yeah, it's it's one of the challenges that we're working on. And we have various kinds of user testing and user centric meetings where we gather together people we're working with the outsiders disease community currently developing a what we're calling a disease portal. And this will be a way that, you know, if, if you're interested in a specific disease, and you can come into (platform) from that perspective, and then find all the relevant proteins and mission?	
Interview 17	37:04		What are the challenges in managing (platform)?
Interview 17	37:09	Well, for me personally, it's, it's the money, that's always my biggest sort of thing that I'm worrying about, and to ensure the continued funding in (platform)s, and	
Interview 17	37:28	that's the one that keeps me awake at night.	
Interview 17		So we, for example, will be reapplying for our NIH funding and we've already started planning out the goals and milestones of vision for the next for that next grant. So, I mean, I hate to say it, but, you know, Brexit is another challenge and it makes it more difficult to recruit the best people from around the world, in the (country) , particularly continental (region)s, unsurprisingly, less willing to come to the (country) at the moment because of the uncertainty caused by Brexit.	
Interview 17	37:31	And also, you know, with that recruitment, then the fact that many companies are trying to recruit data scientists and the sorts of people we're interested to, it's difficult to compete with those cell phones. So yeah, keeping getting and keeping good people is Yeah, mate remains a challenge in this area and won't get any easier, I think for the next few years.	
Interview 17	38:57	So I think that the fact our funding is Best constant. And yet we're living in a, an exponentially growing world of data. And so the this kind of the third challenge is keeping up with the scale of the data and the complexity of the the information that people so I think the papers describing biology these days, more complex, have more data types, bigger stories in than they did perhaps even a decade ago. And so, that presents challenges as well. How do you how do you represent all this data and keep the efficiency of the curators improving? So plenty of challenges, but that's what we're here for and makes the job interesting. So	
Interview 17	39:50	and we think we can we can, you know, manage, we'd love more funding. We've made estimates that actually Actually, if we had double the funding, we could probably curates basically all of the world's protein literature. With our current level of funding, we do have to make strategic decisions about what to focus on.	
Interview 17	40:18		Great. So, we are now finished with our organizational part. I want to focus now on the data governance and functionality on the platzer platform itself. So, first of all, to give me an overview, I know you mentioned it a little bit in the beginning, what functionalities and possibilities does (platform) offer.

Interview 17		So, the main one is a few, an entry few of a protein I suppose. So A user can look up a protein with a search and then get an entry. This will tell them more or less everything that they might want to know about that protein. And the information comes from a combination of this manual curation from the literature, but also some automation information integrated from other biological data resources.	
Interview 17	40:51	And that's really the heart I think, of (platform) that are these sort of genome for us. And we'll be developing a disease view of the data as well. And people will want, you know, download this data and use it locally. That's another important use case. And we also provide API's so that other computational biologists can access the data and repurpose it and reuse it within their own work. I could probably talk for the next 20 minutes or so in more detail, but probably that that's a sort of a broad brushstroke.	
Interview 17	42:14		good. And how do you describe the user off the platform?
Interview 17	42:22	So we get asked a lot, who is I use the community, and particularly by funders to tell them about that.	
Interview 17	42:32	And it's actually really difficult to do.	
Interview 17		It's even more difficult to do in this world of GDPR. Because, you know, we can't keep and track weblog and so on and work out who people are, that's just not allowed anymore. So we, uh, we meet people at conferences. We have lots of requests to our help. desk and we get to know people audiences that way. Social media, although the audience you meet by social media is a bit biased, certain kind of person. We and also through surveys so we take we get data from the FBI does a user survey.	
Interview 17		And so we can look at that data for the people who use (platform) and understand sort of a bit more about them through that because they asked to say, Well, what, how do they identify themselves? So we we have a lot of computational biologists, geneticists, genomics, cysts, structural biologists using a cell biologists, all kinds of experimental biologists of every every time.	
Interview 17	42:37	We know the audience is large. So according to our web logs, Something like 700,000 unique users per month are accessing the websites, you have to take those numbers with a pinch of salt.	
Interview 17		It's it's difficult because some some people might be using multiple IP addresses, and other IP addresses might count for 1000 different people behind the firewall. So it's these numbers. But they give you an indication of the order of magnitude of users. It's hundreds of thousands of people per month. We have we do have clinicians using (platform), but there's probably a much larger number of people that could be using the Prots.	

Interview 17	44:14	So we can also look at the citations of people who are citing (platform) publications in their papers and in fact, we've Got some sort of nice images from doing that there's something called I think it's called last viewer, if you know that, but it's a neat tool that you can use for finding out who's, you know, from a group of publications and make networks and so on. So that's quite useful to me as well.	
Interview 17	45:24		Are there any difficulties during the exchange process of data?
Interview 17		You mean with the users or? So yeah, for sure that I think that one of one of the biggest, biggest issues is is around the structuring of the data. And so for certain parts of our work, it's very well organized, they're a good ontologies or controlled vocabulary. That one can utilize the standards in the field. And, you know, we have adopted or been involved in creating those standards. But other parts of the data, they've been in the database and growing for 20 years, and we started putting them in before there was good structure, you know, descriptions around them.	
Interview 17		And so for some data types, then it's difficult for users to extract and computationally summarize and so on, because, you know, either they, the ontologies don't exist, or we haven't adopted them yet, because it would be such a ton of work to go back and, and relook at all the data and puts it into the new structure. And so, you know, with with this, you get this, this balance of what, with limited manpower, what do you do with your time do you just Keep extracting all the latest information from the rich? Or do you go and backfill and tidy up existing data to make it more usable?	
Interview 17	45:34	And we have that conversation and discussion regularly, when when we're looking at different data types, we have a kind of a long wish list of what we would love to do with various data types and how we could organize and structure it. But we don't have the resources to do all of that those things. And so we just pick off the ones that we think are most important. So I think that's it. Yeah. One of the one of the important data challenges.	
Interview 17	47:44		How do you estimate the reusability of the data?
Interview 17		Oh, yeah, we reuse is, I think, something that that really needs a lot more attention. It's very difficult to measure reuse. So you can you can measure use relatively easily. I think I'll get a proxy through citations and so on. But once someone takes (platform) data and puts it in their resource, or almost always the provenance gets lost in doing that. I mean, I'm kind of okay with that. I think we want to make (platform) use widely useful as possible and for it to be with us.	
Interview 17		So you don't want to put barriers up saying you must have this provenance trail in this particular way. So just on that, that front, we did actually change our licensing. I think it was (year/number) from a creative commons, non commercial license to just Straight Creative Commons CC by license, which made the data freely usable by everyone, as long as you attribute it. In fact, not everyone attributes it and everyone just lives with them.	

Interview 17	47:50	So I mean, I've been thinking about ways to try and calculate the use of (platform) data and it's really very, very hard. We know about, you know, NCBI ref seek takes the (platform) Records and shows those so that's relatively easy. We know that thousands of people download all the files by FTP. And probably we know from our surveys that the people who do that they tend to make it available to other people within their institutions. So we know there is reuse there, but we don't know this scale of it.	
Interview 17	50:00	I mean, I've thought about doing, you know, research by using Google searches. And just, you know, looking for specific phrases from (platform) and seeing how often they reappear in on the web. And those sorts of things, but they haven't really got beyond thought experiments yet. But yeah, it's a great it definitely, people need to do some work in this area that we used to try and understand how much this data is we used and it's probably a sign of a successful resource is that we use? Yeah, it's very difficult to track.	
Interview 17	50:39		(platform) has opportunity to upload data, and what are the requirements for the submission of those data?
Interview 17		So there's two sorts of upload. One is where a user is bringing their own data, and they just want to do some kind of analysis like blasts similarity search will make multiple sequence alignment. And then there are essentially no requirements. The other part is if they're uploading proteomics data, or proteomic sequencing of proteins, and there it has to be uploaded through this tool called spin. So built into that tool or a variety of sort of checks that happen.	
Interview 17		And there are certain rules and requirements about the source of data. Actually, I'm, I'm not the right person to ask about that. There are people in the organization who know exactly the rules and regulations. But because it's quite a small aspect of what we do, it's not something I spend much time thinking about. And then once the submission come in den a curator an expert sits down and work through them and and checks goes back to the authors if need be to ask more questions.	
Interview 17	50:53	so on. So we, unlike places like the sort of nucleotide archives, then we do not include every submission that we get. So whereas some things, archives, I think they're kind of obligated to take the data, whereas we have a sort of a certain bar that that has to be gotten over. So certainly not. I'm not sure what the proportion is, but it's not 100% of the data gets incorporated.	
Interview 17	52:42		So this is similar to the question, how do you ensure that the upload of people is insufficient quality?
Interview 17	52:55	Yeah, so this this is really the the curators job. So jet, there's generally a paper associated. So the work has gone through peer review. And also this double checking by the curator, I think the major Oh, and, of course, the the tool, the upload tool itself has some basic quality control in check the data. So I think that the sort of three, three ways that we ensure the quality	
Interview 17	53:26		related to quality is also trust. So how do you ensure trust in the data?

Interview 17	53:41	That's a good question. I would have to go and ask someone about that this sort of specifics of how what would be the warning signs for something was not trustworthy.	
Interview 17	53:59	Yes. I'm not sure about that.	
Interview 17	54:02		Okay, then I can continue with the next question. How do you ensure that users of the platform deal scientifically correct with the data?
Interview 17	54:17	The users? Oh, no, we we can't really control that. No users can can go in abuse and mess. mess things up.	
Interview 17	54:29	Very easily.	
Interview 17	54:32	So we, I think, yes, that's an interesting question. So i i i think that Yeah, we're not obligated or have having responsibility for for downstream users mess ups of the data. We certainly take responsibility if there are errors in the data. And we absolutely want to fit those. But downstream of that, I think it's probably better than look out. I expect there's something in the licensing terms about,	
Interview 17	55:16	about these sorts of things, but I don't know them off the top my head. Okay.	
Interview 17	55:26		Coming back to uploading the data on the platform, how do you encourage users or organizations to use (platform)? And also to upload data?
Interview 17		Yeah, so we are the kind of standard place for uploading the peptide data. And so this you'll find this if you go browsing through sort of various journals, instructions to authors and other websites, have recommendations on yet where to upload various data types. So, and and some journals enforce those things other journals don't enforce them. We, we don't spend a lot of efforts trying to make authors submit this kind of data to be honest.	
Interview 17		Honestly, it's not the most useful data in the world anymore. It was, you know, quite useful, I think, some while ago, but we maintain it as a service to our users and user community so that they still have somewhere to submit this kind of data. But this could change actually in the future.	
Interview 17	55:42	So there are technologies being developed for protein sequencing using nanopores or surface based sort of fluorescent techniques like a highly parallel admin degradation. So it could be in the coming five to 10 years that actually protein sequencing becomes a more realistic possibility and and will definitely be there with open arms and be encouraging that community to submit data and I think then that could be really, really valuable.	
Interview 17	57:24	But we'll see. I mean, we just have a watching brief on	
Interview 17	57:30		one special aspect about (platform) is that it lies in the scientific community. So what do you think? How does the scientific culture influence the use of the platform scientific culture?
Interview 17	57:56	interesting.	

Interview 17		That's a good question. I'm not really sure. I haven't thought about that. I mean, certainly that there's a kind of a culture, I think a certain groups of scientists just probably won't use (platform). And there's some sets who just know, they only work on one protein and they know it. They wrote most of the literature on it, and they know it very well. And they really have no need of using it. I think they're not going to find out anything new.	
Interview 17		But increasingly, biology is sort of more of a high throughput data science, people are working on 10s of proteins and finding new things related to whatever process they're working on. And that sort of more genomics high throughput culture. I think those sorts of people find (platform) totally essential, because I just want to quickly dip in find out what this protein does. And (platform) really helps them with that. So that's one cultural aspect.	
Interview 17	58:05	I guess there's another cultural aspect, which is a geographical one. So people who are in the US are more likely to use ref seek from NCBI. And (region)s and other parts of the world I think are more likely to use (platform). Although the offerings I think, are quite different, (platform) is better.	
Interview 17	59:32	So that, you know that that's another sort of cultural difference. You know, you might if you use NCBI resources, generally day to day, then you're just more likely to use resi because you're set teams, but you know, we work with the ref seek folks and try and coordinate and make sure that we're not duplicating efforts.	
Interview 17	59:56		What development Do you see for (platform) in the next 10 years?
Interview 17		Hmm. So I think going back to the growth of sequences is a big one, and increasing the amount of data from environmental sequencing and these Tree of Life projects. I think in terms of interpreting human mutations, I'm hopeful that we'll see a lot more data on deep mutational scanning. So rather than collecting information about individual mutations from the literature, that people will just in the lab make every possible mutation for all the human proteins and do high throughput experiments to understand the functional consequences of those.	
Interview 17	1:00:01	I think that will really transform our ability to understand personal genomics data and the human genome. And I think (platform) would be a great place to have that kind of data and make it useful to the world.	
Interview 17	1:01:03		And what does the success of the platform depends on then?
Interview 17	1:01:11	Oh, money, people management, the same things, the same, the same things.	
Interview 17	1:01:20	But engaging with the right communities also is important. to make sure that we, pick the right things to do, pick the right problems and take the right approaches to solve them.	
Interview 17	1:01:37		Great. Is there anything else you want to tell me about success factors, or what comes up to your mind?

Interview 17	1:01:44	I think we've covered a lot of ground here and I can't think of anything immediately that we haven't talked about. It we should have done so but if I think of anything, I'll drop you an email.	
Interview 17	1:01:58		That will be great. So now I have some points for the statistics. So the software behind unit prod is developed by yourself, correct?
Interview 18	Time	Interviewee 18	Interviewer
Interview 18	0:00	Which you can look up, see approval as a good resource. So some of the things you ask actually have written the answers here for them or at least bits of information.	
Interview 18	0:16		So, they are asking the same question as me.
Interview 18	0:23		Good. So let's start with you in the beginning. So before we start, I would like to know something about you. Your background. What are you doing?
Interview 18	0:34	Well, I'm a professor and Department of biochemistry or biochemistry, cellular and molecular biology at (town) University Medical School Center in Washington, DC and a PhD. Mainly in molecular biology. I used to work in a lab doing genetic engineering of plants, viruses and things. But then I moved into bioinformatics many years ago and now I do bioinformatics. I work for (platform) the resource that you found. I'm also involved and have been involved in several other databases and data resources for proteomics and	
Interview 18	1:22	other types of data.	
Interview 18	1:24		Okay. And what's the history behind (platform)?
Interview 18	1:30	Well, it's a long history there. It's the universal protein resource and it was formed. Want to say (year/number)? Is that what we say in our website (year/number). From the merging of three other databases that kind of covered the same territory. The oldest was here was at (town) was the protein information. resource PR. They ran a database they originally in the 1960s released a book. We outgrew the book made a computer database, I was not there then but	
Interview 18	2:14	distributed in other ways.	
Interview 18		And another database in the 1980s called (platform) was started in (town). And they started doing the several things you read in the literature and curating protein sequences and protein function. Something we did at (platform) were kind of they were kind of competitors for a while. A spin off from (platform) was the (platform) database at (insitute) and (instiute) and (country). Where (platform), strictly curated from the literature which time somewhat time Consuming task.	
Interview 18	2:17	And they made an automatic dump of all the other sequences that were pouring in for (platform). And that gets annotated now by various sorts of rules automatically stuff because there's so much sequencing and data comes in so fast. And a lot of it will never, never be studied in the lab directly. There's only so many model organisms of scientific labs and study all these proteins and their actual functions.	

Interview 18	3:32	So at some point, these were all merged the funder saying this is silly. We have three competing things or semi competing.	
Interview 18	3:44	So they formed a unit funded in part by a grant from the National Institutes of Health in the United States.	
Interview 18	3:59		And what is Core offering of (platform)?
Interview 18	4:04	Well, the core offering is one of our databases, we have several databases called the (name)	
Interview 18	4:16	which is consists and we have all this document in various forms our website of the (platform) section, which is a little more over half a million entries. It's expertly annotated and reviewed.	
Interview 18	4:34	We still sometimes use the term manual. It's it's hardly just manual labor and stuff. I mean, we have expert curators that have a lot of sophisticated tools and things, but they do read the papers and curate from them and do other sorts of analysis and stuff to spread the annotation on the function.	
Interview 18	4:55	They focus on certain model organisms there.	
Interview 18	5:00	The (platform) section is automatically annotated but not individually reviewed. Something we do have rules that do it and we have ways to check that the rules are accurate but	
Interview 18	5:13	and that's by far the largest section	
Interview 18	5:17	closing in on 200 million records just for that section alone. And those are after we weed out things that we know are not good. And we emerge identical sequences and things.	
Interview 18	5:33	So that is our core offering.	
Interview 18	5:36	Us biomolecular molecular biologists, biologists, bioinformatics folks around the world. It's a global resource produced by the (platform) Consortium, which consists of people in (country) on the(platform) section and (institute) in (country), (country) that's an international organization. They're not all British people. And PR here in the US.	
Interview 18	6:05		My work now is about success and success factors. So how do you generally define success or a successful platform?
Interview 18	6:26	Well, I mean success. We measure it by how many users we have and try to measure the impact we have on science stuff. And that's true. I've worked in some bioinformatics software companies into and their success for companies is usually sales, but they're your users and things like that. And it's the ones that are free. It's mainly by how many people adoption, adopt them are free. software tools, resources like that. So we measure our web traffic. That's a big thing. Now people using interact through the web.	
Interview 18		We have large data sets that they download. And try to measure that we try to measure. So citations of our database in the literature, which is difficult because we're such an established resource, and there are other databases like that (platform), the structural databases and some of the (platform) and bull nucleic acid resources and stuff that are so common, everybody knows about them. They don't necessarily get cited.	

Interview 18	7:10	At least, you know, maybe if we're lucky 70% of the people who use us in their research will actually put a citation in their papers. We've checked on that we've we've searched through their papers, and look at citations because no, that's true and and other resources say the same thing or position.	
Interview 18	8:08		What vision Do you pursui you with (platform)?
Interview 18	8:16	Well, our official mission statement is to provide the scientific community with a comprehensive, high quality and freely accessible resource of protein sequence and functional information. We worked on that for a while and it's held up we have not changed that mission statement.	
Interview 18	8:38	I don't know how long we've had it now 15 years.	
Interview 18	8:41	And what is the vision behind it? vision while we are on the vision part. I mean, usually for each Grant, we come up with a slightly older vision and stuff. But we do that by providing these databases and resources. Try to work closely with the scientific community do outreach and things I don't don't really have a good vision statement for that. Or I'm forgetting. Let's see if I wrote something down on that. Description depository.	
Interview 18	9:43	Vision is we're going to keep doing this and improving it. We think it's quite valuable. I think it's proven to be valuable that we've been around so long. I mean, literally, some of this activity started in the 19 60s before it was a computer database that people needed to know protein function and sequence there. We just had individual sequences of proteins. Then we started doing some nucleic acids. Of course, all these people have retired and some of these people have died since then. And then when sequencing took off, then it became a whole new world and there's a lot of other resources came into play. There more competition and things, but	
Interview 18	10:29	we have survived.	
Interview 18	10:32	So we think we're doing a pretty good job and people tell us that and stuff. It's just so calm, but he wants to know what have you done now? What have you done this year? That's new and better.	
Interview 18	10:46		So coming to the organizational part behind you and (platform) - what is the organizational structure?
Interview 18		well, collaboration between three institutions and groups. The resume put produce the (platform) the (platform) and the (platform). So, and organizational instruction is we have three co principal investigators. Okay. Currently it's(name) that (institute) we took over a several years ago from rough we're all who helped start the inner road and got the original grant organized and stuff there's (name) is fairly new and this was broad section and exactly the third p p i there and currently it's (name)	
Interview 18		And (town), actually she's at (town), and the University of (town). She has groups in two places. She acts, she took a job. She was at (town) and took a job at the University of (town). But she's still the (position) of that section. So we have over the years divided up the responsibilities obviously.	

Interview 18	10:57	Some always were I mean, the, the group producing (platform) is always produced it and (platform) and we produce a couple other databases called the (platform)s and we're responsible for some of the other API's, the FTP site and some other things so we've worked out there's areas we have to collaborate and areas where we kind of leave each other alone. They do that part and unless we find a problem	
Interview 18	12:55	we don't mess with it. This is took some time to work out We're sometimes early on disputes about how we should do things and who should handle the website and things. But	
Interview 18	13:08	we fought those out years ago. So we tried to work by consensus, formally. And this is accidentally fairly recent if the they've agreed that if two guys want to go one way, and the other doesn't, they will still go that way. But we try to work it out by consensus first.	
Interview 18	13:29		And how does the structure look below the PI level?
Interview 18	13:36	Well, we have a number of key staff.	
Interview 18		I'm one of them. These are the people who actually do the work and and manage the other people are producing, you know, run the production, do the curation, develop the tools and things. So there's a few key staff at (platform) were by far the smallest of the groups there. There's some others at the other institutions and they organize internally.	
Interview 18	13:39	They have their own way. Some are little more hierarchical than others, and how they manage their personnel, the people working on it. We meet, we meet. We used to meet in person three times a year that's been going down. We are having a face to face meeting in March, but we're having some more. We have phone calls, at least once a month, sometimes more and there's sometimes individual groups that meet online Skype or now we use zoom.	
Interview 18	14:42	Meeting software a lot. A lot of email.	
Interview 18	14:48		And how many employees work all together for (platform)?
Interview 18	14:54	Well, that varies, but When it's gone down over the years funding is always tight. So currently, we say we have all three groups a total of around 80 full time equivalents. Working. So that's about 80 people. We often list more than 100 people because not everyone works full time. I mean, I work I get paid, sometimes work more for 24 720 5% of my time on going abroad. Now in some of the other groups, there's there's curators that 100% paid just to do curation and some developers hundred percent paid to do development and things.	

Interview 18	15:46	It kind of depends locally and stuff. Our particular grant doesn't fund everybody. So most of us are other institutions have other sources of funding. For similar other research and things, so some of them work on other projects too. But most of the things we are similar in some way or I mean a lot of the other grants or contracts that I work on, I'm probably there partly because of my connection with the interpro. I have the knowledge and I can use the data, promote the data through it. And some of the people that I work with there will be using the data, so it's useful.	
Interview 18	16:35		I have another question to the employees, which different what kind of competences do they have?
Interview 18		Well, it depends on their job. I mean, most of the ones that do curation are masters or PhD. A lot of them are a PhD level scientists Developers they come from right i think that they're good at developing the code. Some of the web masters or PhD, some don't. Pretty much everyone has some advanced degrees though.	
Interview 18	16:48	What's important for we have a few outreach people that are specialists in you know, organizing work groups and getting information people that way. We've recently hired some more user experience people that work on the website, and just how the database is structured and things so majority of people I interact with are PhD scientists of some sort some people	
Interview 18	17:48		and you describe that your three different PIs and - how are the different responsibilities distributed?
Interview 18	18:08	Part of it like I said is historical. Okay. (platform), folk group hand handles that. So they're ultimately in charge of their output and the production of that part of the database. And similarly with the (platform) section from (institute(, we've, we produce University these are sequence clusters. It's part of a sort of specialized set where you want to look at the sequences and in groups so we cluster them at different similarities. We're also several us are involved (name) and become more important because most of the sequences now is complete genomes which produce complete proteins. 99% of what comes in now our whole genome sequencing projects and stuff.	
Interview 18	19:08	We're involved in clustering those. So they've kind of evolved. And so what we're doing we have had some disagreements in the past about who was going to do what, and they usually been settled by one group doing a better job at it.	
Interview 18	19:31	Or that happened on some of the clustering things from the past. But now everything actually most of those things have run smoothly. We've kind of divided up the work and the fact is, we can't compete. There's so much work to do. The database has grown so much that	
Interview 18	19:53	it's not worth it. I think there was some internal competition People know each other. I wasn't working for (institute) at the time and actually formed I came in a few years later. I did work at PR once before it was part of the interpro. But then I left work for a software company for a while.	

Interview 18	20:18		Coming back to your position and also the other key staff and employees. How are decisions made? And what are the decision path behind it?
Interview 18	20:39	Well, we have meetings I mean, and there are some of the key staff will meet separately there. There's a meeting about curation when they want to change creation standards or how that works. Because there's curators at all three sites, you know, and they use some common software and stuff	
Interview 18		So mainly done by consensus, or one group makes a more powerful argument or something that they're pretty collegial. Now a lot of us have worked together for many years. So we know what topics will be contentious and what wall and sometimes some of us who've been here work work together to make sure it goes smoothly.	
Interview 18	21:00	I don't we don't have a formal system on that. I mean, we can if it gets contentious on something, then we'll have the P ice decide but it usually doesn't do that they prefer that the people doing the work decide. We do get advice from outside we have a scientific advisory group and stuff and they sometimes tell us we should be working in other areas. Want us to explain how we do stuff and we do and they sometimes go quite useful feedback. Because our advisors often work at other resources and have some experience in this	
Interview 18	22:06		how do you strategically position (platform)?
Interview 18		Well, we're kind of lucky because we don't have any more a lot of direct competition. So we are a knowledge base. Okay, most of our our sequences don't aren't submitted to us directly. There's only a small subset and stuff. And so we accumulate linked to a lot of other resources. So we tried to be the hub for genomics. People work on interactions, people working on structures. We provide resources to to map to these different representations, molecular data, and things so we kind of feel we're a key link between them.	
Interview 18		So that's how we try to position ourselves for a knowledge base on protein function stuff. The three dimensional structures, they have another database, we work closely with them. There are other databases that work closely on interacting proteins. We actually helped contribute to one of those.	
Interview 18	22:13	Similarly, there's other there's the gene ontology group for coming in with standardize functions for genes. And we participate in that or one of the big contributors to that so we try to position ourselves in between are these groups and collaborating there used to be competition and some of these fields but again, it is so difficult there is so much data that everybody's kind of said, Okay, you guys work on that. We're going to standardize these names. You will accept those names and put them into your database and things like that.	
Interview 18	24:04		Coming to a different topic, how is the platform finance or funded?
Interview 18	24:12	Well, I can't give you a complete breakdown.	

Interview 18	24:17		Though I have, I don't have it with me I have some number subway somewhere and stuff. Our key unifying funding is a grant from the National Institutes of Health in the United States, that pays for 50% of the money actually spent on it. Sometimes we say it's a little lower and stuff. And then because of its importance, most sites have some other funds that they leverage for this. Like I said, there are some people you know, the work part time on this and then they work on other related things that are funded separately. There are Some grants
Interview 18	25:03	I mean, not strictly the (region) Union but there are see let me see it may have some of that written down here. So that is a key thing and we have to renew that every several years and we're going to renew it again this year.	
Interview 18	25:34	There's money somehow through the (region) molecular biology laboratory (institute).	
Interview 18	25:48	The (country) government I don't know the proper name for the group, help support.	
Interview 18	25:56	(platform) section and (town), I had that listed somewhere.	
Interview 18	26:07	If you really want to know I could send it to you later because I'm not coming up with it now.	
Interview 18	26:20		Yeah, you can send it later to me if you want. That's not the problem.
Interview 18	26:25	But essentially, (region) groups and not think it's not formally the (region) Union and stuff. But ambolyn stuff, there are grants. (institute) seems to have be fairly well funded and stuff and I think they supplemented through some of their funds. And the (country) government helps support the (platform) and we have some other grants and resources here that support some of the people here there's a protein ontology grant is support some people at PR and stuff and the are obviously related. So some people work on both. And that's partly true. I know there are some people work on (platform). But they work on some other databases they have also that are related.	
Interview 18	27:17		So could I will continue with the next question.
Interview 18	27:21	We actually had a website, we have a funding if you go about (platform). They mentioned various Institute's that give little bits of money and stuff. We sometimes get supplemental grants to do some things, particularly within (platform) cities to get some, some grants due to specifically spend a little more effort curating certain pathogenic organisms or something.	
Interview 18	27:46	Go on, that's fine. Okay, good.	
Interview 18	27:49		What are specific goals you are pursuing and how do you control the goals you've said?

Interview 18		Um, well, there are certain goals. Since we do have some grants that, you know, we have to set goals and things. So we definitely propose that we're going to curate automatic rules produce new rules produce so many numbers of annotate so many particular entries or review so many entries and things. There's also various improvements we promise to make on the websites or some related certain analysis tools.	
Interview 18	28:01	Maybe add some additional annotation or improve certain annotation and things. So we have a very sometimes they're enumerated with with actual numbers on them and most of our grant proposals and we try to meet those internally, there We have other things where we have to improve our database or our pipelines because as the data grows, some things that took a little little time before take a lot of time now. And they're some of our data comes from external sources are related sources. And if they change their processes, we have to change our processes to do to cope, to keep the pipelines running.	
Interview 18	29:29	It's fairly focused, we produce these resources and we have to keep producing them and improving them and we try to add new beneficial things where we can	
Interview 18	29:40		And how do you control the goals you set?
Interview 18	29:46	Well, we monitor them I mean, it we have and our multiple yearly meetings, work the staff get together and then other meetings we sometimes review Our milestones and how we're proceeding on those goals. So, in the in the grants, we do submit, we say in the next five years, we're going to do this, this and this we do review those routinely. Are we going to make them? Are we not we need to modify it. Sometimes things come up that were we didn't anticipate that we have to undertake and let me decide what will change this or change the delivery dates on that.	
Interview 18	30:29		What are difficulties and challenges in managing unit?
Interview 18	30:40	It's large. Being an international consortium has its benefits and problems. I mean, there would be efficiencies if everything was done at one side, I'm sure. Would it be better though, having the different groups coming with different ideas definitely helps. I mean, there's many ideas that I've had or others have had that sound great until you talk to the other group and they point out problems that you hadn't anticipated and stuff. And sometimes this results in something better. Sometimes it results in, you know, that wasn't as good idea as we thought. Because a lot of people use their data in different ways. It's hard for us to know everything.	
Interview 18	31:30	I monitor the health mail and some others do, because that tells us a little bit more about how people are using it or trying to use it in ways that won't work. So that's quite a useful thing. And we often get new ideas that way, or somebody trying to use it for something and we say, Oh, well, we can improve. That wouldn't be hard if we do this than that. And we outreach to new communities. It's been extensive outreach genomic communities in recent years with all the interest on genetic variation, variants and genes.	

Interview 18	32:11	We're trying to interact with those groups more directly.	
Interview 18	32:16		Coming to more "useful things" - functionalities and possibilities does (platform) offer?
Interview 18	32:27	Well, the best way to describe that been we we offer a very high quality database depending on which organism you're looking at.	
Interview 18	32:50	We have very good data. Is it perfect? No, no, nothing could be the challenge is huge. We offer very Good search services better than most people know. Because it does take a little effort to learn it nowadays everybody expects Google style search to solve everything. And we, we do that as best we can. But it's this data is fairly complex that it has a certain structure, it helps if you know it. And its strengths and weaknesses. It's not really answering your question, but you repeat the question actually	
Interview 18	33:35		shows you what functionalities and possibilities does (platform) offer?
Interview 18		Well, we offer some great core key search and analysis possibilities. On functionality. It's basically the on search, and we offer actually offer quite a different ways. I have some slides and I kind of people I mean, we offer you many ways to access this data and data many ways for you to slice and dice it. If you just want a table and an Excel spreadsheet of our data, you can make it on the website. If you want to do very specific searches, search your data in certain ways.	
Interview 18	33:45	You can do it on the website and get it in variety of formats, if you're a computational person, even have what's called a sparkle endpoint, which is designed to integrate with other sparkle endpoints to do cross database searches and things. That's a fairly advanced thing for mainly for programmers in certain groups, but it's quite powerful. We offer a certain set of unique tools and tables. Like I said, Our identifier mapping allows you to map gene identifiers or protein or structure identifiers various different identifiers in one resource to our to the proteins in our resource and vice versa. That's a very popular tool and database, we do have some other basic search and alignment tools peptide search tool that's fairly popular in certain corners peptide match tool.	
Interview 18	35:26	We have one problem. It's unrelated, but I'll tell you about it. Our funding, we're funded as a resource, so they don't really want to fund us to do analysis or develop new and novel tools. Though some of us would like to kind of feel the funding agencies at least in the US want to fund other people for that, and resources like us just to maintain the data and make it available useful ways so come up with a new, brilliant bioinformatics way to use the data and stuff. It's not necessarily they want us to spend our money on that.	
Interview 18	36:13		How do you describe the user group?

Interview 18		Well, it's fairly new servers, I'd say a largest group are still bench scientists and stuff looking to find out things about the genes and proteins are doing. I mean, I actually have a table from this report or a community of who they are. I mean, obviously, bioinformatics and computational biology people are large genomics and genetics is a large group that uses our thing. Structural biologists, people studying human diseases. There are proteomics Protein science cell biology. This is all going down. I'm looking at a graph, molecular biologist, biochemistry.	
Interview 18	36:17	Those are some of the largest groups. But we have neuroscience, drug discovery, evolutionary biologists. As you see it's informatics and some aspect of biology. biomedicine and stuff are most of them because that's basically uses our data. In commercial commercial groups use it, companies use it, research institutions use it. It's not for the general public unless they have an interest in biology and protein function. They're happy to use it. And we do get queries from some or students that want help on their papers. We get queries for that. But we can't help them in that. We're not we're not We have disclaimers I mean, no we we will tell you a proteins are associated with disease. We don't provide any medically medically relevant information and stuff don't take what you read on us for treatment,	
Interview 18	38:20		what difficulties arise when exchanging data via the platform?
Interview 18		no better difficulties. I mean, internally, we have difficulties because some of the data is gone so large. So people want to download all our data, they better have a lot of disk space and a good internet connection because it's going to take a lot. But we don't recommend that you should download the parts you need.	
Interview 18	38:28	You don't need to get everything. Dealing with the size of the data and its exponential growth is a problem. Otherwise, it's just now, there's a lot of resources out there. Sometimes. We've been around long enough that most people have heard of us, but they don't always know about or use us as much as they could I think it would be beneficial. So	
Interview 18	39:21		how do you estimate the reusability of the data?
Interview 18	39:37	Sure, we, not sure we've come up with a good metric or that we do know that people use it. And once I've told you, there's metrics defined it reusability we try very hard. We offer multiple formats. We offer extensive documentation.	
Interview 18	40:03	They can access this in various ways, but not, I don't have a better answer for that. Because I how how, what is reusability of the people use it? I mean, they can reuse it. They can they do. We do get requests for archived versions, sometimes people want to go back and for some reason, check what they did before or what somebody else did from earlier release, which we can usually accommodate it depends on the specificity of their query.	
Interview 18	40:45		So I can continue with the next question. Okay. What requirements do you have for submitting data?

Interview 18	40:56	Well, we don't take Many direct submissions. We do most of most of protein sequence data comes from translations from gene sequencing from DNA sequence. So we take those are submitted to (platform) or the (platform), they actually are a consortium if you submit any of those, they exchange the data overnight and stuff, translations of those we import into (region).	
Interview 18	41:36	So 99.99% of our actual sequence data starting material comes that way. We still take some that are done by direct protein sequencing. There are chemical and and now there's some mass spec methods where you can directly sequence the amino acids of a protein. We do have a portal where that submit We'll go and that directly gets seen by a curator. We do have a list of requirements of metadata, we want to know about where the protein came from the species	
Interview 18	42:11	if you have a publication with it and stuff, but I don't know a few years ago, that was maybe 300 a year, which is pretty small. I'm not sure. I think it's actually declined since but it still happens. We still get people wanting to submit their sequence. But for most of them the route to submit is to submit to (platform) or (platform) and and we will pick it up in a few months. When whatever the databases sync up,	
Interview 18	42:42		how do you ensure the quality of the data?
Interview 18	42:47	Um, well, when anything that's due comes in gets there's some automatic analysis That we do I mean, if it's 100% identical, and to end with sequences we already have which is quite common and it's from the same organism.	
Interview 18	43:13	They get merged together automatically in the (platform) section. Since the (platform) sections needs manually review that takes a little longer and stuff. So we try our goal is actually to represent the one canonical sequence for one gene. Now, genes have multiple versions of a protein sometimes, so we try to put that all in one entry. That's a goal. It's not necessarily always met because merging some of these isoforms can be difficult for the quality so we have some quality checks there. We haven't already Live where we keep all sequences	
Interview 18	44:08	from all organisms merged, if they're 100%, identical end to end, that's called the unit part archive. Occasionally, in the course of annotation or building rules, we find things that just are completely wrong there Miss annotated their contamination from a bacteria and some other genome or something. And if that gets identified by users or us, we will remove it. But it stays in the archive. This gives us one way, if we see it again. We can identify and remove it again.	
Interview 18	44:45	It comes back in from some other source.	

Interview 18	44:49	Then, like I said, if it's a manually reviewed entry, they will find if they find mistakes, they'll get rid of it. We have some automated rule system. And things and they start picking up them. We have some automated procedures because there are new new hits to the rule that don't look right. We have ways to automatically flag them and have people sometimes look at them take out the rule change the role. They find the sequence is really a mistake, because they look at it, they'll remove it. They'll stay in the archive.	
Interview 18	45:26		And how do you ensure that the users of the platform and use the data scientifically correct.
Interview 18	45:38	We can't really ensure it what our data is completely free. You can redistribute it, you can do pretty much what you want. We'd like you to attribute it to us.	
Interview 18	45:53	We used to worry about that more, but it's just we can't control what people use. If they asked us, we will help them I will tell them, we provide lots of documentation but don't really have any enforcement mechanism.	
Interview 18	46:12	Some of our data gets taken and redistributed by others, I mean, several other institutions download our whole data set and put it with our own tools and reuse it. We did have some worry that some people would take out little bits of the data and or the annotation and not use it properly. But you know, we've done our best to document these things.	
Interview 18	46:39		And how do you ensure trust in the data?
Interview 18	46:45	Well, like I said, I have a document here committing to a core trust seal group and stuff which is trying to kind of put us there, several	
Interview 18	47:02	attempts and groups out there that try to do something like this do to try to make a trusted repositories that will preserve the data and people can trust this as a useful resource. So we're applying to one of these now and I think this is the last go round. They review our comments and things.	
Interview 18	47:26	Partly we publish our own papers and things and we use our own data. And we think that and give some trust to the scientific community. We've been a while. The (region) has designated us a core resources this elixir project. Go on our homepage, you'll see a little seal at the bottom about elixir. elixir Core Data resource. This is something from the (region) and we'll Have a court trust seal emblem down there hopefully in this year.	
Interview 18	48:07	These are just little things that help people know they can trust us if they don't.	
Interview 18	48:14	I mean, when I trust the resource, I go look at other people have used them, I look at their data and make my own evaluation. I also look at how often they update their data on evidence of funding, you know, other groups are funding their data and the resource has been around a while, which we have probably more than anybody, I think that's a good good sign that they're probably trustworthy.	
Interview 18	48:41		And how do you encourage researchers and organizations to actively use the platform?

Interview 18	48:49	Um, we talked to them we go to meetings, sometimes have workshops, showing them things that we have, they may not know it They didn't look too deeply and what can be done with it?	
Interview 18	49:06	That's our main thing. It's what we call outreach.	
Interview 18	49:11	Try to make a better website. We do have occasional workshops with other groups. If we get funding for something like that we'll invite some other resources and scientists to attend partly to get feedback from them about what they like dislike. What they're feeling is our about the resource. And we get good feedback, how we can improve it that we have problems, certain things aren't working for them. And they often find out that Oh, the stuff they wanted is there, it's just wasn't obvious. It took some, some digging, and how to use the resource.	
Interview 18	49:47	And knowing what we do with the data, and do you see more ways and channels to reach to them. There are many ways it's just	
Interview 18	50:02	Like many things, at some point it becomes how much funding do you have? And what can you do with it? Can you hire full time person just to do really good outreach? I seen some other larger. You know, institutionally are government funded resources that have done this. And, yes, they got some very good people who can really give presentations and talk about it. But that's a full time job for them. We have not been able to fund such a position so far.	
Interview 18	50:40		Okay. One special aspect about (platform) is that it is integrated in the scientific community. How do you think does the scientific culture influence the use of the platform?
Interview 18		Well, quite a bit. I mean most of us are scientists, many of us have spent some time has been scientists, somewhere in our career and stuff. So we come out with that perspective. So I think that helps us. So you can lose touch. So I say outreach is important to find out what they're doing now. new techniques, new types of data come up, we try to incorporate those are sometimes a little conservative, because history has shown that some of these new techniques and stuff aren't as great when they first come out as people thought.	
Interview 18	51:01	There's various instances in the past where we've been told they want us to integrate some high throughput data we have and then it's come out that the data wasn't that reliable. And the worst thing for something less is have to go back and fix something like that. So we're a little conservative on some of those things. Let's wait another year or so see how this plays out this new technology before we we adopt it and incorporate that data. Because people have been burned in the past and it's sometimes very difficult to go back and retrofit something you've added.	
Interview 18	52:22		What development Do you see for (platform) in the next 10 years?

Interview 18		Well, we're trying to work that out. We have a vision for a new grand stuff, but it's not a 10 year grant. I want to grow, we want to have a new, better website. We're going to have to adopt some new technologies to cope with the data. better integration with genomics and stuff. That's one with thing. It was simple, but it's not. A lot of people think these things are simple, but they're simple if you stick to one organism and one data set, but if you aggregate data from multiple sources and research groups who find out its little more complex, slight differences in how you do things make make certain mappings a little more difficult.	
Interview 18	52:36	Partly because all the different groups are working in the past somewhat worked in isolation. So we're trying to get tighter integrated with the genomics groups and we're succeeding. We can now go map amino acids and stuff back to the codes, DNA codes and stuff but each group tends to	
Interview 18	54:00	Do things their way. And then there's there's global attempts to standardized processes and things like this and data processes too. So I think it's going to be a lot better. But there's still a challenge and we're working with those groups.	
Interview 18	54:14		And what and what does the success of the platform depends on then?
Interview 18	54:22	Well, better penetrate we get with the other resources and the easier it is for the research community to get what they want is basically will predict our it's our success.	
Interview 18		We I think we've an our group, I mean, we've been around (year/number) as (platform) but really some of us some of the group, the group's not necessarily individuals have been around for decades before that. So crossing our fingers, we hope we will survive more decades. There's a lot of institutional knowledge and ability that's built up. So, but we'll see, it does take a fair number of people and some steady funding for these things. And not everyone appreciates that.	
Interview 18	54:42	So, you know, the fact that we actually have people curating Some people think this can all be done automatically now, but it can't. We do a lot of things automatically. We're using machine learning techniques and stuff too. But there still is no magic. There still needs to be laboratory research and other sorts of research. And unfortunately, now most of that gets captured in the literature which is difficult to extract.	
Interview 18	55:58	We're trying to get more community involvement. Various groups and included have tried this over the years. But so we're taking we actually have an online. It's online now where people can submit papers related to a certain protein and what they're doing. We don't let the community annotate directly. There have been attempts, wikis and protein wikis and things for that, that some of us tried this. Back in the 1990s, before there were wikis and stuff, but that's difficult.	

Interview 18	56:44	People have different opinions about things that people do. Tell us and tell us about updates and stuff. We add those to the things, new functional information comes out. People pointed out to us and stuff. We're looking at ways they can contribute more directly and Automatically, but still keep the quality up. Because some people will just say, some papers interpretation is wright or wrong. That's the way research is sometimes we try to resolve that or don't put down stuff that the community is not comfortable with.	
Interview 18	57:20		Do you have anything else what you want to tell me about success factors, (platform) challenge challenges or so?
Interview 18	57:35	Not that I can think of, because there afterwards I will.	
Interview 18	57:42	I might answer thing fairly clear to you. I'm not sure. Yes, sure. I mean, I will.	
Interview 18	57:50		I will take my time to transcribe everything. But yeah, I'm sure I can work pretty well with this interview.
Interview 18	58:00	Okay, um, so I'll see if you have any particular questions you can email me.	
Interview 19	Time	Interviewee 19	Interviewer
Interview 19	4:05		Now I heard something about your history. And what is about the history of metabolites. So where does it started? What was the reason?
Interview 19		it was started in (year/number) by my predecessor (name) who is now at the University of (town). And it was realized at that point that metabolomics was really going to evolve from quite a niche area into a more widely accepted omics. And so he, he came from the research side of things, and he was Muslim expressing but with the interest in the computer science, so he got funding from the (state) government effectively, to establish metabolites. You got two grounds basically to set it up. But that one And the flow of data. So as there is now, so what he established was a place where hardcore metabolomics people could come and put quality data sets and build tools around them as training sets effectively.	
Interview 19		And it wasn't so much what we would call a repository, and which is what a lot of what (institute) does, we have large scale genomics places, proteomics places, and, and protein stuff. But metabolomics is always very small. And when he came to the end of his nine years, which is the usual career evolution at our institute, and the directors asked me to take over the database, that was the (year/number) January and am and they said, Okay, now take this and make it into a Service repository that can take large amounts of data and can be reused by everybody and can do this that in that and help people who don't know anything about metabolomics. And, and that I embrace that challenge.	

Interview 19	4:15	I've done it before I spent many years building resources. And this was slightly different because I usually started from first principles. And this case, I had to come in and take over something that was existing, understand what it was trying to achieve, reach out to the community, them, is it delivering or what else do you want? And that's what we've been doing for the last three years. And why it's ongoing, because the field is changing every moment.	
Interview 19	6:46		And what is the core offering?
Interview 19		The core offering is the data set. And basically, it's the information about the studies. So you have and this is a bit unique to that. Two other resources, we actually request the raw data as well, so that people, so you get the raw data. I don't know how much metabolomics you know, but it's a mass spec output. And then there's a whole processing steps and steps and steps. And then at the very long level, you then are told what you have actually seen by analyzing the spectrum. What a lot of people do is they just put the end result summer.	
Interview 19		So it's basically telling you, this is what we saw, but it in other resources, they don't tell you how they got from A to be and they don't make a available. We've always insisted that he should be available so that other people can come back and look at that very expensively generated spectra, use more up to date tools, more up to date libraries, and reanalyze it on an ongoing basis in order to do that, and to come parrot, which is critical in science, because this is very expensive science, you can't just do it on your desktop like you can do genomics today.	
Interview 19		And, and I do mean. And the key thing is that you document every step of the process. So what temperature? What kind of volume? What kind of machine? And how long did you use blanks? Did you use blanks again, halfway through, you have to document absolutely the majority of your experiment in order for someone else to either replicate it or compare it to what they're doing. And that's our strength right now. And, and a lot of funders, even when they're based in other countries were required their researchers to submit to us because they know that we will not accept the data unless it has all those components.	
Interview 19	6:49	means it's quite hard work for submitters. So that's basically the big change that I had to make when I came here. It was set up for, as I said, people who did Metabolomics every single day, and completely understood everything. So what we have really focused on in the last few years is completely changing the submission model. So it was a downloadable tool that you did locally, then you upload that and it failed. Then you downloaded it, you made a few changes you uploaded it failed. It got very traumatized by that. Now it's all online, you go step by step suggestions are made to you. There's little video tutorial in the corner for you to click, because people don't ever even in my time, but definitely not the younger generation. like reading documentation. We are little videos,	

Interview 19	9:58	press this button. Okay, go for that. Um, that has worked really, really well. So we've gone from getting a couple of hundred studies a year to 100 studies month, because people can do it so much more easily. They understand what they're doing and they don't get frustrated. And also we use quite a lot the drop down menus, so they don't have to sit there trying to guess what you're asking. It's one of these options, choose one of them. So that's really helped them. And now the next thing that I really want to focus on, because I come from	
Interview 19		even though I was originally trained in chemistry, biochemistry, I went into very much the protein side of things. So I was the content lead for (platform) for a long time. So now my mission is to connect metabolomics into the other omics because metabolomic is actually the I always used to argue with the genomics people that yes, you may have a variant, but it doesn't mean it has any consequence on the protein, you can make lots of changes to a protein.	
Interview 19		But if it's not an important part of the protein, the amino acid change won't make any difference. If it happens at a certain place, then yes, it will have a consequence. So the two communities have to work together. And in between them is the proteomics one. Because a lot of genes are never expressed. We don't really understand why. Or they're expressing really, really low abundance, and you need a certain critical mass for it to be actually useful. So for instance, if you have a blood test, and they're actually testing that you have a certain protein in your blood, they really should be quantifying it as well as detecting it because if if you don't have enough then you're going to You and I, but they'll say, Oh, no, no, we saw it.	
Interview 19		So you're fine. And you know, but the final thing is then the metabolomic, because you might have the proteins. But if the proteins are not in the right place, not in the right cell, or in this sub cellular structure of the cell, then it won't do its work and the metabolite, which is the outcome of what the enzyme is doing, at will tech will be missing. And that's the final conclusive. This is what you've got wrong with you. So, I have been doing some prototypes with the English National Health Service. And then he just where they have seen certain conditions and they have all the blood tests of these people and they have their exome sequencing.	
Interview 19		They have these variants from what is the so called norm that is in itself a bit wishy washy at the moment, because we don't know everything. Obviously, in the building right next to me, they're sequencing 100,000 British people at the moment in order to be a bit more precise on what is norm. But considering there's, I think, 65 million English people 100,000 is a drop in the ocean. But that's the concept that they have.	

Interview 19	10:43	So, with the work we were able to do, taking the metabolites that were identified in the blood, I could narrow it back through the pathways, biological pathways to say it was one particular variation, that was the one that was causing this particular outcome. And so then they can screen for it better. And if someone didn't have that variation, and something else was going on with them, and you have to work with them slightly differently. Power. That's the power.	
Interview 19	14:00		Very, very interesting combination of all those different fields and how they are connected. So I'm wondering, because my work is about success factors, how do you define success or a successful platform
Interview 19		that people find it easy to use? That is the most critical thing. And this is what I've had many years of designing databases and designing websites way back. You're much much younger than me. But you know, we didn't have Windows machines. We did not have Netscape or Google or any of you we had nothing like that. Now we do, but there are still a lot of very clunky interfaces, and I inherited one, which was fine for a very niche community, but took a lot of time.	
Interview 19		But if you have to do this as a critical part of your daily work, which is now what funders are requiring, it can't be that clunky, you can't be sitting around for three days submitting something, you just get frustrated. And when people get frustrated, especially scientists, they do the minimum that they can get away with. If you can make it easy for them, then they'll give you the richness of the data, they'll go above and beyond.	
Interview 19		And that benefits both sides. It benefits other researchers who come to use their work, and it benefits the my repository. And another way that I'm planning to evolve the database similar to how we did it in (platform) is the more information you give me. I'll give you more of a goldstar. So there's a minimum requirement, but if you give above and beyond, then I'll give you a gold star in your fingers can see that You did a great job.	
Interview 19	14:22	But the ease of use both of submission and by other people were using the data is success. Because if you can design the most fabulous thing, but if your community doesn't like it, they won't use it. Or if they have to, if they're made to that, like I said, they will do the bare minimum and that helps nobody.	
Interview 19	16:23		And what vision Are you pursuing with the metabolites?
Interview 19	16:29	vision.	
Interview 19	16:31		So do you have a vision for the future? What do you want to reach?

Interview 19	16:36	What we wanted to develop because there is so there one other resource and metabolomics workbench in San Diego and hopefully meeting I have met them before and I'm hoping to excitement New York, and that we will exchange data. I don't know if you know the STC model, which is that the (region)ns, the (country) and (insitute), take all the global genomics data and exchange it. So the (country) have already decided that they want to have a centralized resource. So they took our GitHub, basically. And because everything we produce a DB is freely available. And they're installing it locally, and they will set up an Asian point.	
Interview 19		We will be the (region) side, and the Mac in San Diego will be the (region)n side. But to be honest, a lot of (region)ns submit to us anyway. So you know, that's fine. As long as we don't exchange the data and have all of it pool together, it will make a very powerful but the key vision is to make sure that the data evolves, because the way that metabolomics- and you will probably notice this business chemist was that it was very much associated with With pharma, and, and ecosystems, but now it's firmly being used in the clinical setting.	
Interview 19		And we are control, developing right now, a controlled access version of metabolomics. There is no such thing so metabolomics data is very, very hidden, because no one has a forum to put it somewhere where people can get it. And obviously, through ethics committees, but this is the so we're developing that right now. And what we already have such a platform here at (institute) for genomics data, which is globally accepted.	
Interview 19	17:30	So we're working together so that in the future, people can come and submit the genomics data to EGA, the metabolomics data to us and that they will cross link so people will understand that they are able to download both sets of data and understand how they relate together. And that's going to be incredible powerful for disease. But it's not just disease having diseases what everybody, to certain extent gets obsessed by. But there's also a very I had a call this morning very early and about nutrition. So I just had an apple for lunch. And if you eat the same Apple, exactly the same Apple, the metabolites might be different. Because you have a different microbiome to me, you have maybe a different enzyme diversity to me. And	
Interview 19	19:40	one of the biggest challenges to human health is is not and a lot of it is actually preventative. This is a key point and we don't fully understand. We think Eating very healthy because we're eating a sports bar. And they said this is healthy. And you're like, Nope, it's full of sugar. It might be full of fruit sugar, but it's still full sugar. You need to understand this combination. So everyone is into this right now. But a lot of it is.	

Interview 19	20:21	It's quackery (crackerly?), basically. And the science has to be done. But what people have noticed when they're dealing with people with health problems, and they tried to change their diet, there is this always humans. are little bit cheating in their self reporting about what they eat and drink and smoke. This is a human nature. Apparently my Danish colleagues don't lie at all. They just don't care. And because I've been involved with some studies there so they tell the truth, but they have no intention of changing lives to good	
Interview 19		But, um, there is also a factor that now studies have shown that even when they control the environment, when they put people in a room for a week or so and they could have access to no other food, they still weren't producing the same metabolites as the person next. So some people are following the diets they're given and for some reason, it's not giving them the same benefit as it is to someone else. And that's critical to understand both for to help those people in particular and to make them feel better about the fact that they're not responding the way everyone's expecting them to.	
Interview 19		And also to put a stop to the crackery. To say, this is what for you, personally, is the best thing. And if you want to live to build in good health, we're all living to build but the number of people who are living to build, not good health is bad for that. person, but it's also bad for the health system. And it's bad for the government and the general financial situation. So this is where the science has a huge ability to help people. Then there's the other thing of as climate change happens, and more and more plants are not growing where they usually grow. So for instance, in France, they're very, very worried about their, my French is appalling terror.	
Interview 19		So you know that certain wines, for instance, are, they can only be called something because they're in a certain region. They're struggling now in certain regions to grow those particular vines, because climate change has changed. The environment. It's too wet, too. It's too hot, and the vines not growing. So how, how do they respond to them? There's so I mean, I understand that they have this concept of a geographical designation. And you're like, Well, yeah, we can grow it in (country). That's not champagne. That's just fizzy water, or whatever they say.	
Interview 19	21:00	But the point is, sometimes these vines are now growing better somewhere else. And the taste of the ones that are still in their traditional areas are not tasting the way that they're supposed to. And while I don't particularly care about these things, obviously, certain countries do, and they want to know how to adapt the plans so that they can adapt to the climate. But there The thing is to learn about what the plant actually truly needs, so that you can move those food groups to a more important place and find other food groups that would grow more effectively with the climate that you have. And this is what I,	

Interview 19	24:05	I'm not so interested in making an artificial environment, what you need, if we have climate change, we need to adapt to it. And putting whack load of fertilizer on something is really not going to help in the long term. That's just setting up yet another problem. So you,	
Interview 19	24:26		You just mentioned quite a few seconds ago, that there is an ethics committee that decides about data release, can you say something more about this?
Interview 19		So this is about human data, in particular, and so people when people sign up to your particle cohort study, depending on the person involved or Hi faces, there is a blanket approach that people don't want their data shared. Because in the paperwork, it will, for instance, if I was part of a cohort study, it will say my name, it will say where I was born, it would say, my age, it would say my BMI, it would say what medication I was taking, it would give my address, and then it would have my genome or my metabolome. And that's very, very private information.	
Interview 19		And in certain countries, like in the states that can be used against you. And insurance companies won't give you medical insurance if they know you already have either a medical condition or you have a medical condition in your family. And you're you're just blocked. And in countries like the states where there is no state help, really, that's a big problem. In my home country, and in this country. There is a state system. And in (country), you cannot it's written in law, that if you take private insurance, the private insurance company cat penalize you for inherited conditions, they just have to put up with it, basically.	
Interview 19		Because that's it. We don't have many private companies who want to take that chance. But we have a state system. So it's okay. And countries are different. Some countries are very open to sharing some parts of the data you I guess, I don't know if you know, the whole of Iceland was not that many people. And they all got sequenced. And that is used for by companies to do medical science. They're doing the same in my home country because until very recently, we've We're also a very small population. We're still very small, but we were even smaller.	
Interview 19		(country) 3.2 million. So you know, not a lot. Were anywhere else except home, actually. So again, we're very homogeneous people. And we have very specific and (country) is similar. They are very specific diseases like celiac disease, lactose intolerance, which is not normally associated with white people, for instance, it's more associated with Asia, but for various reasons, we have evolved that way as well.	

Interview 19	24:40	So, this date, the ID, the decision was taken to make this global and it will be as protected as possible. So all that personal data is hidden from the researchers who come in to look at it, for some of it might be released, such as say She's white, Caucasian, and her BMI is this and her medication is this but they would hide everything else. But even to get access to that information, they have to go pass an ethics committee to say that this research is going to good do good stuff with this. They're not just going to go and	
Interview 19		make a Fauxpair. Some bad things happened in the early years with this thing, and there's very, I don't know, if you're aware, there's a very rare disease where people they can't be exposed to sunlight because their skin peels off. I don't know if you've ever seen people with this, but it's incredibly painful.	
Interview 19	28:22	And it's very unpleasant and and people who have rare diseases like that often are very happy to get involved with companies that will help them find a cure but In this case, what the company actually came up with was a cream for very which white old ladies to help them not get wrinkles. It did not help the children in any way, shape or form. So now people are very protective. And I understand this completely. They're very protective about their data. So there's a platform called cipher.	
Interview 19		So, this is where our families give their permission to their conditions, to share details about their children, and everything is protected, except the phenotypes are compared. So for instance, there was a clinician in (country), I think, realize that condition in the States was the same one and by the combining them, they were able to understand what was happening to the children. They can't cure it. But they now know what is the faulty gene that's creating it.	
Interview 19	29:29	So, and this is very practical. Unfortunately, that family lost two children. Because of this. They both born apparently looking completely normal, very, very well. And then when they were about one, they started, they died very quickly. And it happened twice. They didn't want to have any more children until they understood what was actually happening. Now that they knew that it was this gene that was causing it, they could do selective IBM and choose the embryo that didn't have the default. And then they were able to have a healthy baby. So they can't fix theories from a problem, but they now know what's causing it. And they can have if the family wants to try again. They can help them.	
Interview 19	31:02		And who is sitting in those committees?
Interview 19	31:06	So that's appointed by it's a mixture. So it depends here in the (country), I was only looking this up yesterday for another reason. And they appoint conditions, they appoint patient representatives. they appoint local legal people, and who also understand, you know, GDPR and everything. So these committees are multidisciplinary. And people, for instance, from here are on these committees as well. We advise but how to protect the data.	

Interview 19	31:50		You have very interesting so, this question were not on my guideline, but I really want to understand what's happening there.
Interview 19		A lot. So for instance, also, there is a control access part. So the (region) chemical agency is now doing requesting metabolomics as part of approving chemicals. And again, I don't know if you know them I didn't, that the data is submitted to by the company to ACA, and then ACA s experts look at it, the ACA staff don't actually have access, because it's all IP of the company, they just calling the experts to say, is this safe?	
Interview 19		The experts say yes, it's safe, then. This is just wrapped and encrypted. Now they want us to, to help with that. So it's not just medical data. It's also IP for pharmaceuticals. Because the day to day is submit Then their patent will be invalid if someone could see this data basically. And so it's in all aspects and you have to manage all of it because you need to enable people to progress. Like I said, the verities disease community because of them getting hurt came up with this concept of decipher where it was clinicians talking to clinicians and parents being involved, and then it progresses on and, and that's very, very powerful.	
Interview 19		That's just you know, mix at the moment. It's, it's based here actually the institute next door to me, and, and it's particularly helpful. That was a very acute case because the children died, and it was originally more what was tissues for the most is developmental disorders. The so you can see the child is slower to walk slower to talk slower to do everything. So there's something going on but you don't know.	
Interview 19	32:01	And so many of these children just get a developmental disorder as a label that does not help the parents know what to expect of them. What help to give them should I focus on their mobility or on their nutrition, it's very, very hard for parents to know what to do.	
Interview 19	34:37		Okay, very, very interesting insights. And continuing now with a more organizational view behind metabolites. I'm interested in the first in the organizational structure behind it.
Interview 19		So it's a split according to the data that we collect. So you have the we use various ontologies, where we've built them ourselves in collaboration with others to capture so the instrumentation data, the protocols data, they asked a data. So that said data will be, for instance, human information, or plant information, the organism part and the taxonomy. And then it will be the protocols will be capturing exactly what you did.	

Interview 19	34:55	And that's all split into different tables and then connected across by identifiers, various, it's not simple at all because we capture so many different kinds of information, but the overall concept of it is straightforward. And then the idea is by using the ontologies throughout it, you could for instance, pull out any study that has been connected with a disease or any study that was done using a certain machine or study that was with a certain plant and our certain protocol. And that's the concept that our users really want.	
Interview 19		So it's very much structured we're going through / a bit of analyst is a key thing for every platform that you ever advise on, is that you need to build in that it constantly evolves. You cannot write things in stone. They have to be modular. They have to interact across don't over engineer for the sake of it, but don't minimize as much as possible what is free text or what can be what we call in the business a dump. So it but it's a very, very tricky concept to balance between a dump and overengineering, where you break it down to the tiny tiniest component and then adding in a new component takes her investment. That balance is absolutely critical. What we have to do right now is I want to because the files are so huge.	
Interview 19		We have a certain amount in, for instance, the raw files are not in the database. They're separate and just linked by identifiers. But we're now capturing new data reflecting the way the field is developing. So for instance, they weren't that interested in quantification before. We now have to add in a column what quantity Did you see? And another thing is foxo. Mix. I didn't have your of this, but I metabolites is the only metabolites at a certain moment. So you take a blood test right now, you take a blood test and a half an hour, the results can be different.	
Interview 19		And that's focus on banks and what people are really interested in Again, it's very important for human data. A lot of people take medication, which doesn't actually have any impact on them at all. But again, this coming back to personalized medication and personalized medicine, you can take the same blood pressure pill as the person next to you. But because of your own personal physiology, it could go straight through you and has no effect, or it has only a minimum effect.	
Interview 19	36:12	So being able to track someone through the day, I didn't. In the past, people would say put a blood pressure monitor on people and they have to wear it all day and this is big, clunky thing and they're just testing your heart on you. Every now and then going me. Yeah, are you still there. Yes, I'm still here.	
Interview 19	39:02	It's the same metabolomics. But the thing is we needed right now we're developing our, our templates, again, to actually document what probes you're putting in for the flux omics. Because the probes themselves, depending on the sensitivity will do different things just like the machines. So we have to document this added additional information. So that's basically it, trying to think of everything you possibly might want to know. But plan for the fact that they'll always be new things.	

Interview 19	39:40		So now you were focusing on the technical structure behind the platform. And what is about the personal structure behind it is everything in your team or do you have different different app store?
Interview 19	39:57	So, Most things are within our team in the sense	
Interview 19		that we have our own environment. But excuse me, it's within the (institute) framework. So they maintain the clusters. So they give us access to the clusters of machines. We have to manage how much we're going to use. And when we're going to use it to make sure we don't compete with other resources here at the Institute. That's an important thing to understand. They have to we also have to track how our database is growing for storage.	
Interview 19		Because again, that centralized there isn't a problem. When I asked for more storage, they give it to me, but I have to plan every year next year. I need this much storage based on my growth in the past year. I expect to need you to buy this extra storage So that's one component. The other components. And that was another meeting I had this morning is because we have a central API website where you can search all the data across the institute and try to relate it together.	
Interview 19		So because I'm changing my data model, I have to talk with the search team and explain to them, our file dumps are going to be different, you're going to have to index them differently. And you need to understand how they relate to fields and other resources. So we're very much an ecosystem of resources here at (institute). That's not necessarily how people and other Institute's do it. But in my opinion, and it was saying this because I've been involved in the creation of Muslim and it's the most powerful way to do it because I met blow makes unit practice proteins pride is proteomic eta, eta, if the only all that exciting science I told you about is only going to work, if we can all talk to each other, our databases can talk to each other.	
Interview 19	40:07	So that's an important thing. Also, if you're setting up the platform to understand Are you ambitious enough that you should connect into other platforms that already exist or being planned? Because you can end up and this is the biggest, biggest crime in science, you can end up being completely siloed. So you can be doing Beautiful, beautiful science in your little field. But no one else will get to exploit it. And that's not how we do science here.	
Interview 19	42:48		So how many employees work for metabolites and with what competences?
Interview 19	42:54	So right now, we have	
Interview 19	43:00	I count them, there are enough here, one, two, three, four, 5, 6, 7. 3 curators, and 4 programmers but one programmers more biomedicion, and he has ontology skills as well. So the curators are obviously chemists. And I hope to recruit next year a metabolomics passion to keep it moving.	

Interview 19	43:37	The software, the programmers are software engineers, with various they evolve. Right now they're all into Jason and Mongo DB. And but at the (institute) we provide constant training and new technologies. So while people are here, they they don't just come with what They were trained on before they evolve with the Institute, they learn the new upcoming things. And like I said, one person is more of a bioinformatician. And he he does ontologies as well.	
Interview 19	44:16		And how are the responsibilities distributed?
Interview 19		So because it's such a small team, I have a project leader. That's Ken and he's been here the longest, to be honest. And but he he's also very good with people. And so he plans out with me every week. Not every two weeks. I'm not here every week, and I'm too busy off getting money. And he plans out to work with me every two weeks and then we set a month plan and then we discuss it with the team. We all agree with We're going to do this called Scrum palling.	
Interview 19		You've probably heard of it. So we do a scrum and retrospective. And I introduced that when I came to this team. They didn't have that concept, but I think it, it helps people be realistic about what they can do because I'm an eight manages expectations. So people will say, Oh, I can do this. I can do this. I can do this. They're like all of it at the same time. I don't think I've been doing this a long time when they're young and enthusiastic.	
Interview 19		But with the scrummy, especially with the retrospective, you can see actually we only managed to do 60% of what we said we were going to do. So then over time, they have learned that let's be more realistic and say this is what this is going to take and this an hour we're at about 90% we achieve what we hope to achieve And I think that's realistic really. And so he leads that. And then I have a senior curator Kiva and, and she also does outreach and training.	
Interview 19	44:21	Again, this is something that we have in house training here. So we as called train the trainer. So basically, the core training team here that will teach more traditional scientists how to evolve into training other people. And because that's a skill set in its own right, just because you know how to do it doesn't mean that you are the right person to teach other people.	
Interview 19	46:45	As I've learned, that might be a particularly scientific problem. But not all people have social skills in quite the right way for teaching other people. So we run courses here as well. So that is part of what we do.	
Interview 19	47:05		and other competences or tasks?
Interview 19	47:10	Well, we have a big user help component that again comes back to the social skills part of things. And but I have insisted that all team members share responsibility for the help desk and that they learn how to understand we as I said, we have a global reach. So not everybody who writes to what my team is not.	

Interview 19		I think we have one British person and yes, so. So English is not our first language and neither is it for many of our submitters. So that is another competency that you have to respect When people write in and say they're having difficulty with a particular area, being able to understand what they're saying, being able to interact with them, and ask them, what's going on being respectful about this being timely about this, this is a big thing that I care about.	
Interview 19		We are front facing. And we are a service. And I think that's another area where some resources fall down on. Some resources will say, Well, if I answer it within a two week window, that's okay. But I don't personally agree with that, because there's someone waiting on the other side who's really stuck. So I insist that you, if it's something quick, you answer it, if it's something wrong, you let them know that this is actually going to take a little bit of work.	
Interview 19	47:38	And then I'll get back to and try and give them some kind of timeframe. So That they don't get frustrated with you. This is really, you can develop a fabulous platform. But if you don't help your users, it won't succeed. It comes back again to what are you building the platform for, to use, hopefully.	
Interview 19	49:19		And when it comes to decision, whatever kinds for the platform, how are those decisions made?
Interview 19	49:30	I am a very this, this comes with. It depends on the manager, personally.	
Interview 19		And, again, it comes back to the mantra that I've tried to say to you, we have to deal with our users. So I took over this resource. I listened to what the staff told me why they did certain things certain ways. They showed me what They were doing and they told me why. I said, Great, thank you. And then I went out to our users and said, What would you like? Are you happy with this? And then I got a long list of requirements. And then I came back, and we put the team information and communities information together. And I said, How can we work this together?	
Interview 19		What is realistic to manage for the community? What is realistic? and be honest with me, what parts of what you're doing right now, do you think is silly anyway? And we were able to build a story together. In the end, I make the decision, but I want to, because I'm the one who knows how much resources we have, how much money we have. But then with that information, you can go back out to the community and say, Thanks for the feedback.	
Interview 19		This is what we're going to do. And then you're managing their expectations. And if they want more from you, then you say helped me write a grant together helped me connect into funding sources and then it will move faster. But unless you have a clear what people want, you have a clear what resources that will take. And that's where your team will help you understand how much work that is. put those two things together and you can make good decisions.	

Interview 19		Sometimes you have to make hard decisions because you don't have the money but you also have to be I'm sure you've heard the expression, low hanging fruit. And let's start with what we can start with the will already make people's lives easier. And let's break down the harder problems into smaller components.	
Interview 19	49:44	And maybe it will take us like it's taken us three years to completely revamp the submission system. It is much better people are saying lovely things about us now. But it did take three years, not one year as they told me at the beginning, but I didn't believe it. sounds funny.	
Interview 19	52:07		So you just mentioned the funding part. Yeah. And how is the metabolites funded?
Interview 19	52:15	So we have a mixture of funding. So I don't know if you understand the way the (institute) is set up. It's an internal governmental organization. So a bit like the EU, but separately, so countries join our member. mothership are our overall organization which is (region) molecular biology laboratory, which the headquarters is in Heidelberg, Germany. Then, there is in Hamburg, and in (how many blank) the south of France and Rome, in Spain, Spain is the newest one in Barcelona. Okay, so as of today, I'd be quite happier being in Barcelona than here, but no man.	
Interview 19		So countries join and they give a percentage of their GDP just like they do to the (region) Union. So that gives core funds to the entire Institute and then within the entire Institute, an organization it's decided, which goes to which parts and depending on what is the priority, that priority is set with the Council, which has representatives in the member countries. So the member countries say yes, we really want you to concentrate in this area.	
Interview 19		This is really important for us, we work on a five year plan. So that's one thing. Then we are the data science place of the whole organization, we add the compute power for the rest of the organization. Where you get tough were the most outward facing part of the organization in one way. So we have about half of our funding actually comes from grants. So that can be local British organizations, it can be horizon (year/number) it can be the NIH.	
Interview 19	53:12	It can be the (country) government or the (country) government. It can be anything in anything, anything that's open for application. And we apply, usually because we're, we work on global resources. Many of our I mentioned, I'm building this collaboration with (country) and the United States. Many of the other resources here, already have that in place. So the funding is, is global comes here.	
Interview 19	55:00		So you said like 50%, funded via project. Yeah. And the other 50% is more long term?
Interview 19		Yeah, its core funding from the Institute, if the institute has decided that they want this resource, overtime resources might not be useful anymore. Like we had a resource for microarrays, but very few people use microarrays anymore. So it was cutting edge back then. But now the technology has moved on.	

Interview 19	55:08	And so resources are closed down. But as long as the institute thinks that a certain resource is valuable, they, we get core funding. So the idea of that is you you always have a couple of people to do the work. And then you build on it. You can go out and saying, Listen, I have this core funding. I will leverage this. So if you give me this much money, you're actually getting this much money, because I have so many people who are paid for by the Institute and you will benefit from that anyway.	
Interview 19	56:09		And do you have a strategic position of metabolites, what you're aiming for?
Interview 19		So that global coalition that I talked about is our next big push. And like I said, the (country)ese want to do it, and they're in the process of setting it up with the (region)ns. And they also want to do it, but it's, it's a bit more tricky because we have quite a lot to discuss about. They, they capture different data to us. And there's no point in US exchanging data if we don't follow the same standards, or the same data model. So we agree in principle, but we have a piece of work to do to sit together and agree. This is Actually what we need to capture.	
Interview 19		And I don't think there's going to be problems, but that it's just to sit down and actually documented and say, This is what we all need to achieve. Because we also have different folk I, I worked for a long time with genomics data, so NCBI in the states dbj in in (country), and the API here. And if you go to the (region)n website, you wouldn't ever know that the data is coming from the API because it's been packaged in a different way. They have a different wrapper around it, because I know the identifier numbers, I will say on us and epi. I know that kind of stuff.	
Interview 19	56:17	But most users wouldn't have a clue. And that's fine. That's the whole concept of it being global. So the court what they call a future table, that core data is exactly the same whichever resource you go, but the packaging around it, the connecting into other databases around at the searching of it is different on each site. So because they have their own user community, and they want to see it in a different way, and they want to connected into different things. And that's the ideal way that we share what we can share. And we also serve our own communities and their needs.	
Interview 19	58:31		And what are other specific goals do you want to achieve?
Interview 19	58:38	to continue to evolve with the data as it evolves. So, this, one of the reasons why I was given this responsibility when Chris left was because they wanted to connect it into the mix because they began to see the power of what metabolomics could do to have bar genomic And proteomics colleagues and and that is a powerful challenge to, to do. But it's also enabling people who don't understand anything about metabolomics to be able to come to our site and understand the power of it for themselves. Another key aspect, and I really hope I'm applied for	

Interview 19		sure I'm just looking at my board my to do list is truly scary. And I'm waiting for a grant for setting up a metabolite knowledge base. Because partly because this has come out of industry chemistry is one of the oldest industries on the planet. What people call standards for identifying with respect to our costs guys lots of money, so a lot of people are doing multiple ohmic Study. And because they don't have the standard to compare it with, they don't actually know what they have.	
Interview 19	59:30	So they've spent the money doing the mass spectrometer to experiment. But they don't actually know what the spectrum means. They only have a limited set to compare with. And that's what I call it library. So what a lot of people are now saying is that we need to have the central library, what I'm calling a metabolite knowledge base.	
Interview 19		And basically, then you can still buy it as founders. The companies are not going to go out of business. But for the people who do an experiment and want to push it on next, next, they need to know which of those specter actually informative which are the spectra, tell them something about their original study design. I am Sure, they will still go and buy that standard when they want to push the experiment on to the next level.	
Interview 19		But in the meantime, for them to effectively narrow down what they want to do next, they need to have a public resource available of those standards to compare against, and the tools to take their spectra and compare it against it with all the protocol information, all the processing information. It's usually challenging. But I think we, we have to start basically, I'm not saying that we will solve it within one year anything.	
Interview 19	1:00:34	But once we start, then everyone has said, this needs to be done. The community has said you do it will help you you do it. So now we just have to find the money to actually do it. But the goodwill is huge. The community for this because and in fact, companies who make the standards are happy to be involved. Because if we say this is the standard from here, then to be honest people can go and buy it from there. So again, it's all an ecosystem you have to enable everybody to get what they need.	
Interview 19	1:02:23		And how do you control the goals you set?
Interview 19		So I have a scientific advisory board and all the resources that (institute) do and we meet every 18 months, I present to them. My Okay, so there's two things don't have the (institute) has a scientific advisory board. We are reviewed every four years. So we just had ours in just was March last year was the last one I presented my four year plan. I told them what we had achieved, had promised and achieved in the previous four years, even though I wasn't responsible for an event, but I did. I told them what I want to do for the next four years. And they tell me whether they think that's reasonable or not.	

Interview 19		They guide the directors and understanding whether they should give me extra funding or not. And whether they think the science is appropriate. So that's one big thing. So the directors take that into account. Then I have my own Scientific Advisory Board, which meets every 16 months, and they are very specific metabolomics people.	
Interview 19	1:02:28	And so they keep us up to date on what are the areas that are evolving, what data we should be capturing so I can reach out to them for advice, for instance, on flux on banks. And because we haven't had to deal with that, but For it has been purely purely a niche research area but it's now moving into more widespread use. So that all guys me on what I set as goals. And then as I said earlier within those goals, I have to break down prioritization and resources	
Interview 19	1:04:25		and do what kind of challenges and difficulties do you have in managing the platform?
Interview 19		I think I have, a luxury in some level in computer, compute. It's not a problem for me, because CBI is such a big Institute. Other resources really struggle with that another Institute's because metagenomic state is absolutely huge. You can have one studies one Three terabytes. So, but it does pose a challenge in manipulating that data. So if you have one study, that's three terabytes, how do you actually physically compute on it, not just store it, how do you query it? How do you break it into components? And that's a constant challenge.	
Interview 19		And we're growing exponentially. I need with my team to constantly evolve the technology to understand what parts of the data can I compress? What parts of the data do I need for day to day processing and querying by users? What data for instance like the raw data, they will only ever download and put into another computer so they don't actually need to see it. So that can be compressed and put somewhere else until they requested this is all technology.	
Interview 19	1:04:35	Then the other challenge is to keep on talking Top of the user community. So what do they actually want to do with the data? What are the kinds of queries they want to do? And that's where I came back to earlier. We have to keep evolving what we're actually capturing, such as quantification data, the probe data. So that's, that is a big challenge to make sure that you keep up to date with your community, especially one that's moving as fast as this. And then the, the thing is, I have three curators. We've had an exponential growth. They didn't have to deal with that before. And now they're saying to me, can you stop being so successful at telling people to submit to us? Slow down?	

Interview 19		We're getting too much data now. And I'm like, okay, okay. So then it's my job to say, right? Are we getting the right data? Am we are, so that's not the problem. So then where do I need to find the resources to get more curators. But then there's the other point. It's not just growing the curators. And this is another thing that because of my background, was how to automate the processes so that we make the most of the curators, because as I said, I've been in this field a long time before the word bioinformatics was even created.	
Interview 19		And curators with very many skills, were basically doing work that should have been automated, very repetitive work, such as the ontology searching, that cannot be automated, and, and they can help build anthologies, but they shouldn't be typing it in, you know, our remembering it in their heads their tools for them. I have developed many of those. So I can come to this database and say, Okay this bit.	
Interview 19	1:06:55	So then I had a couple of sessions to the curators, I watched what they were doing. And then I asked them to tell me what were the parts that you found the most boring and was doing. And we, we documented that. And then we work through the programs to automate those things. So now the curators are far happier that they're using actually their knowledge and skills, rather than just typing and remembering. And that has speeded us up as well. But we still need more curators	
Interview 19	1:08:39		just mentioned that you are so successful and getting new users. So how do you encourage people to use metabolites?
Interview 19		Well, this is again, this. I was very, very lucky at the point when I took over this resort. And you can never underestimate the power of luck. I am a very outgoing person. I do come from an institute like the FBI, which is very well known. And, and so if you come with API behind you, people are going to take you seriously anyway, because we have a track record. In other resources. I have a track record in other resources. So you already start with the positivity.	
Interview 19		The next thing is to ask them, Do you know what metabolizes? And if they say yes, so why aren't you using it in a nice way to say this? Okay. That's what directly but in a nice way, and then they complained about various things such as the clunky submission process such as this. So you capture all of that, and you you're intensely respectful. You don't say, That's not good enough. I say, Oh, thank you very much for that feedback.	
Interview 19		And then I go back to the team, and I don't always go These things by myself from these guys and the team as well. Otherwise, I would have no life at all. And, and we go back and we say, right, they said they can't use it because of this, this and this. Is that genuine? Is this real? If it is, what can we do to fix it? And then at this, we got to the meeting, the next time we present the changes that we've made, we encourage them to try again.	

Interview 19		And that has been very successful. That every resource you do, that's nothing to do with luck. That's just good resource development. And you don't stop either. Just because you solved one problem. You have to keep asking that question. Don't ever get complacent that, oh, I have the perfect platform. I have the perfect service. Because as I said before, and I'll say it many, many times on this subject, the data keeps changing, but also the scientists that are interacting.
Interview 19		So the data the matter but omics people are generating is changing, but the people who are coming into Metabolomics don't have chemistry background. They don't know what a Spectre is. You have to educate them. And, and they want to be educated. But you have to bear that in mind. You can't assume that they know what these words mean. You have to help them with what they're doing. Where I got very lucky, was at the same time, as I was doing on this outreach work was that certain funders began for the first time to say you have to submit.
Interview 19		It's not enough to put it on your FTP site. It's not enough to put it in your supplemental data on your paper. And, and that was really powerful. And I worked with the NIH to help them communicate to their people, their researchers that they were funding, how they should submit to us. I set up templates where Tonight, the team set up templates to enable people to submit quickly and effectively, efficiently. And that was all a part of the grant. And that was very successful. The other thing was, the journals, quite a few of them decided that they also wanted to make this mandatory.
Interview 19		They didn't before. They were fine with supplementals. But this is something that's happening in a lot of journals. supplementary data, maintaining all of that for journals is very, very expensive. And they don't journal publishing itself is in an evolving, I'm sure you're aware of it, between public access and private and charges for publication. All of that's changing a lot. So lots of the journals have reached out to the BI amongst others. (region) PMC is based here as well. So we can give do eyes, two datasets. So then the journals don't have to maintain them anymore.
Interview 19	1:08:50	They asked their submitters their authors to submit the data to us, then they get a DUI, they can publish that in the paper and they're no longer responsible. But when they reach out to us about that, our answer was, we're not going to store it unless it's good data. Help us. Let's work together to ensure the data is in the right format. We're not just going to be an FTP site where you can dump
Interview 19	1:13:43	it. It has to be useful data in the right format, and high quality so that's what metabolite does. So we now have a process where you submit to us and it's private It gets, once we approve, the curators approve the quality of the set, then they get a link, and it goes to the journal reviewers. And the genuine reviewers can see the data while it's still private. And then when the papers published, the study goes public. So the publication of the data is linked to the publication itself. Yeah. So you so you cannot, as a researcher, upload your scientific data without a publication.

Interview 19		You can upload it, but we haven't traditionally, because no one asked us to made it public until the paper comes out because they want to protect their data until the papers published in the future. like to know max evolved, because way back in the day, there was a paper associated with every sequence.	
Interview 19	1:14:35	Now there isn't. So lots of The data in the genomic sequence have not papers associated with it. metagenomics community is not quite there yet. It is still in the mindset that they need to keep this information private until they get their paper. I foresee in the future that it will evolve like genomics. And that data will go in there and will be made publicly available as soon as we validate it. And but we're not there yet the community is not there yet.	
Interview 19	1:15:30		So coming back to the quality check, how do you ensure the quality of the data?
Interview 19		so we have worked with the community over many years to define standards. So there is a minimum set of information that you need and minimum processes and part of our submission process redevelopment was a lot of that was always Animated. So we made effectively what the curators had. Traditionally as a checklist, we have now automated within the system. So this column needs to be completed it needs to have this values in it.	
Interview 19		And it needs to have no values and measurement types. So as much as we could automate validation, and that's really helped the submitters because as I explained earlier, helping them through each step. They can now see, okay, I finished this page, there's a green tag, I'm not waiting to the end to know that what I filled in here is correct. And also because they filled in here, for instance, a certain kind they've done mass spectrometry not NMR.	
Interview 19		They see an a different next page. So if they see mass spectrometry, they see this page if they do NMR they do see this page so they no longer have this big confusing page with all the options they just see. what's relevant what they put in before. So then that's all automated. So then it's just making sure what the curators that all of that flows appropriately as well as they can. They're still an expert knowledge. And then you can see certain consistencies. So for instance, in the paper, it says and would have done this, but there isn't this.	
Interview 19		So how did you come to that conclusion? And you can. Sometimes something is optional, because it is optional, but there's something that gives you a clue somewhere else that says actually, they didn't do this. So it's not optional. If you have that you should be giving it to us. Okay. So, go back there and put that back in.	

Interview 19	1:15:38	So going forward, we would like to evolve that even further because I'd say mentioned in the previously, the raw data is underlying. So as we build our metabolite knowledge base and we build, another aspect of our work is building tools for people to use. We want to have the ability for the curators to go back to the raw data. We won all the processes, and come up with a new conclusions and label that appropriately saying, This is what we know. Now, we have this better tool, and we have this special library. So we can now say what was not labeled as an unknown spectra. We now know this is this. And the curators are very excited about that, because then they feel like they're doing really good science and helping the field progress.	
Interview 19	1:18:49		Because you just mentioned the different tools for the user what different possibilities and functionalities Do you offer
Interview 19	1:19:00	So right now we only have very limited tools we were involved in a very big brand called phenomenal and if you search with phenomenal just add n metabolomics. And then you find it. And	
Interview 19	1:19:15	this was a / I've been talking too long now so my beginning to bubble.	
Interview 19	1:19:21		I mean, you also get something to drink if you like.
Interview 19	1:19:28	I can talk this much normally. And I did talk and as you've gathered, if you start me off from something I'm passing this about I don't stop and	
Interview 19		the phenomenal platform was a cloud platform for clinical metabolomics. And what's gonna happen happened there was we brought together a whole bunch it started before I took over But about together a whole bunch of tool developers from across (region) was a horizon (year/number) thing. And they had a great deal of fun of tuning their tools, deciding which ones were the best ones and putting them in workflows, workflows are critical.	
Interview 19		Because one tool is not going to do everything for you. There is no black box, you have to know what tool goes up to what tool which is the best for NMR. What is the best for mass spectrometry and they did a huge amount of work on this. And that's available on the cloud. And but then the funding came to an end and the PIs didn't really have a plan. So what I've applied for funding to do is actually to migrate those tools and workflows into our environment here.	
Interview 19	1:19:40	So then I will be able, the team will be able to offer that going forward and we will maintain them which is also the main problem in bioinformatics people spend a lot of money, lots of time and a lot of intelligence, developing tools, and then don't have the resources or sometimes the interest, because they're scientists and researchers, they've solved the problem. Look for the next problem by	

Interview 19		I'm done. Give me something new to do. Constant. Now, of course, people are like that. We don't like to be bored. It's a common thing. And so this is the other challenge that I have to address in the sense of the platform. So our concept is not only that you submit to your data here, that you can come here and upload your raw data, do the analysis here, and then submit it to us. And again, that will help us because the protocols and our user community, because the protocols will then all be audited, we can see exactly what they did. And so it will be easy.	
Interview 19	1:21:17	For someone else to replicate the exact same steps and not just depend on words in a text, they can, they can say, just like has happened in genomics. I use this technology, I use this version, they can come in, do exactly the same thing. And if they get different results, then they can understand that their samples slightly different whatever, it removes some of the uncertainty of the processes.	
Interview 19	1:22:28		And you just mentioned the user community. How do you describe the user of the platform?
Interview 19		but traditionally, as I said, they were very hardcore metabolomics people, and, and there were two types, the people who actually were doing the metagenomics, and the people who were building tools for them. So they were building the tools using the set as gold standard. So The heart the magical omics, people were following those sets as guides of best practice for themselves to do their own experiments. But then it has changed because in the past, they didn't necessarily then submit what they did. Now they have to, so they're doing that.	
Interview 19		So that means our user community is much wider. It's not just the expert in the team who came and had a look and then told everyone else in the lab what to do. Now it can be a PhD student has been told by his professor, that you have to submit the study. So they they show up and go happen to I do. And that's what I mean, again, about helping our users use the platform. We are having people coming in, and we're running a course next month, who are completely new temescal omics are completely new to chemistry. They do not have this language in any way, shape or form that their clinicians that Analysis their labs, by chemists the labs are expanding.	
Interview 19	1:22:38	For instance, quite a few people commission metabolomic studies, so they might be doing genomic studies in their lab, or protein studies. And they said, Well, we have this sample, we'll send it away to a company, they'll give us back the magical omics data. There are quite a few companies, we work with a couple of them that they submit to us as part of their process. But then these people need to understand what does that outcome actually mean?	

Interview 19		And how did and this is a very first step. How did they design their study in the first place, to make sure they can do metabolomics at the end, because even the most basic things, such as how you store it, you can store genomics data genomic. You can store samples, blood samples, to do genomics and a certain way. And that's family. But that won't work for metabolomics. So if you the conditions around the storage of the sample will not protect the metabolites, they will degrade.	
Interview 19		So you need to change right at the very beginning of your study that you storage in a way that is compatible for both genomic testing, and metabolomics. Testing. So, again, we're dealing with people like that. So one of the things is development in this course. So they come in and they learn all of these things that wouldn't encourage them because they're trained in genomics. So we need to help them. And then our latest community is the omics community.	
Interview 19		So they're now coming to the studies saying, okay, I've seen this paper. They've done meta genomics, or to know mix Metabolomics. I understand what genomics is about, because that's my background. I know how to understand this paper, except for this whacking big component, which I don't understand the top. And what does that actually mean? And how does that connect in and that's my next big challenge, because I don't know how much in your chemistry you did in biochemistry, and many universities, that's quite siloed.	
Interview 19		So you need to understand your metabolite might be a chemical compound, but it's usually produced by a protein working on another chemical or another protein. And you need to understand if you have this metabolite, and this is where I see metabolomics people complete and chemists completely and utterly struggling to understand where this fits because pathways in human or implants is intensely difficult to understand.	
Interview 19		tracing your way. So the work I referred to earlier about helping the NHS study, understand which of these three genes was the one that was having this effect on the metabolite, it was not straightforward because all three genes had this metabolite somewhere in their pathway. It's only by understanding the data around it. What is likely to be in that one gene, and that takes quite a lot of protein knowledge, which is what I have.	
Interview 19	1:24:32	But this comes back once again to a mission of mine, that scientific education has become very siloed in many countries, and so you go straight to university to study genetics. And you may never have learned any chemistry or any physics. Any Max, em. And you're like, you really limit your your students in that way. I think it's very important as part of education that they get about broad ground grounding before they specialize. And it's not just because how do you know when you're 18 years old that you want to do genetics.	

Interview 19		But science, yes, but it also helps you evolve with the field, like you have done you, you have business chemistry, now you moving to computational biology and AI, that's the way to go. But you have to, one have the confidence to do that. And to be enabled to learn the skills along the way and that I don't think we're that great at just yet.	
Interview 19		But unless that's why I love working Here, my background is a bit unique, not unique, completely but special. When you have like you have a my team, you have pure software engineers have never studied biology since they were 14 or something. And you have pure chemists or pure by chemists or pure geneticists, getting them all to speak to each other in the same language is a challenge.	
Interview 19	1:28:27	But it's ratably useful for a successful product. Sorry, that's another thing about your platform that you really need to stress is that the people who are developing the platform are multidisciplinary	
Interview 19	1:29:51		Also coming to speaking the same language. What difficulties can arise when exchanging data I have a platform.
Interview 19		So they're speaking the same language. So for instance, very simple thing at Do you know what FTP is? Yes. Yes. Lots of people who try to give us data don't. So uploading data to an FTP site, and they wrote back saying, What is FTP? And then you say, if you read the Golden finiman, here that you have to set this up on your, your computer, you download this, you pull your data and you upload to us. And then they say, Well, I don't have administration rights on my computer.	
Interview 19		I can't do this. And I know that's not language, but in a way it is language it's managing. So right now what we're doing is developing with another team here, something more like the windows model where you just drag and drop They don't have to think, what's, what's behind it. And they don't have to. And you do that by the website.	
Interview 19	1:30:03	So again, they don't have to install something locally on the computer, which they don't have permission to do because they don't have the administration rights, all of these things that horrify our software engineers, but which is completely normal for many users, then managing by language because you can get quite detailed. We would say machine vendors.	
Interview 19	1:31:39	Vendors is an English word. to them. It could be machine sellers or can be machine companies. You need to be constantly aware of how you're phrasing it. Like I said, we have documentation but it is a human fault that we never read it.	
Interview 19	1:32:00		Okay, I get it.
Interview 19		So you have to I refer to that earlier about when when I started here, and they gave me all their documentation, it was something like 60 pages worth. And I just spent, right. Okay. We're gonna make movies. And I read for the thing, this is my job. For our users, they're not gonna wait through 60 pages of documentation, looking for one thing, which they're not even sure what they're looking for.	

Interview 19	1:32:04	Put that little movie in the corner of where what you're expecting them to do on that particular page. And then they'll get it. And we've really seen because we have a Chinese member of staff, and sometimes before we have to ask him to help with something because there just was a communication problem. And sometimes he couldn't help because Person (country) needs don't speak one language.	
Interview 19	1:33:05	And they couldn't really help. But now at the movies, we don't need to help so much, because they can actually see it happening. And they can work it out for themselves.	
Interview 19	1:33:17		And also coming to a data exchange, how do you estimate the reusability of the data?
Interview 19		Well, this comes back to the standards thing again. So we have worked very much with the community I we have basically said, here's what a study looks like what's missing for you to actually sit down and do this experiment again. And that was built the international standards, the MSI, the metabolomic standards initiative. And so for every experiment that they thought of at that time, they said, This is what we need to know in order to reuse it.	
Interview 19	1:33:25	And we just follow that and enforce that. That is the message phenomics society, they have working groups that keep the evolving that and we keep in touch with that. And when they release a new version, then we come back and make changes in ours that we can't do by ourselves because we're not in the lab doing the experiment. We have to work with the community for that.	
Interview 19	1:34:21	And when users can upload their data, do you have any other requirements for the submissions, then filling out all the mandatory fields.	
Interview 19	1:34:35	And they have to give us who they are, their affiliation. It's all protected by GDPR. We don't share that with anyone. And they have to agree that the data is public at a certain time. And they have to agree that if they don't finish your submission in in a bunch of years, then we're just going to delete it. We're not going to keep storing forever and ever. So there is an agreement between us.	
Interview 19		But once the study is and if they never seem to, we have a mechanism. Sometimes it can take a long time for a paper to be published, but sometimes they just forget to tell us. And so we have processes to check because we're conveniently here with your (region) see, if we see our accession number published in the paper in the journals, then we can update the journal reference ourselves.	
Interview 19	1:35:05	And but if they don't come back to us within a time period, then we go back to them and say, Are you still trying to publish this? And so we make it clear that there has been ongoing conversation where a community and ourselves we're not just a place for you to dump so And forget about it.	
Interview 19	1:36:02		And how do you ensure the trust and the data?

Interview 19		This is where the curators come in. And this is what they want to spend more time on. Obviously, if someone completely falsifies the data by copying someone else's work, there's no complete way for anyone to tell that. But that in science as a whole trust is a really big issue. We see more and more that papers are being retracted. Because people falsified data, we have no way of knowing that. All we can say is it looks like a very complete study.	
Interview 19	1:36:07	It has all the components, someone's put a lot of thought into this. They've put a lot of effort into this. So we just have to trust that they did that in the right way and they just didn't make it up on the spot. But to be honest, what we want to push on with, We haven't ever needed to withdraw data so far. I think our community is too small for that. And	
Interview 19		to be honest, I think it's more common in other scientific fields, and particularly genomics and stem cell stuff. There's a bit of abuse in that field. There isn't a reputation for that and metagenomics just yet. But what I would like to put in place is, once we have enough data, you can expect a normal range, for instance, of metabolites who should be seen and quantifications of what you should be seeing. And then if something is too high or too low, we would just flag it up as something and they haven't mentioned this in the paper. Something that is because he sometimes that is the whole point of the paper that this person it is too low or too high and that's why they have this disease. But if they're not even mentioning it, then it should be a red flag that this is something we need to check.	
Interview 19		So the curators are very aware of this. And they're. So one of my curators is I've got her effectively an internship and admits blowing slam in (town), so she's going to spend a few weeks because it's been a while since she's been in the lab, to go there and work with the people who are running the machines who are running and chat with them and see where the field is going. And so refresh her whole mind about how this is doing and this is something that I I mean, I think started as a curator, way back. You are so scientists, you are still curious.	
Interview 19		You're not just inputting data, you want to be thinking about the data and what it what does this actually mean? And when she said, Oh, I want a refresher, I want to understand what they're dying in the labs these days, because everything is moving. Then I arranged that. And that's another thing that's very, very important.	
Interview 19	1:37:14	I mentioned about the cross disciplinary of the platform developers, but also to make sure that if you intend to have people working on a certain platform for a significant amount of time, I mean, she's worked for us for four years. But the field is so quick. Four years is a long time in another field for years is nothing like protein structure. They're still doing what I did in 1990. Hasn't progressed. So, my knowledge, they're still fine. But other fields evolve. And you have to be aware.	

Interview 19	1:40:12		You used focused as far as I understood on the trust related when it comes to uploading the data to the data and base. And what is about ensuring the correct scientific use of the data?
Interview 19		Well, this is the thing about the (institute). And it's a huge question. You're completely correct, but we have and it is the mission of the VI, that all our data is free to use. And whatever you want to do with it, go and make a company out of it. Use it for driving your research. That's fine. We are not.	
Interview 19		It's there in our terms of use. And that's part of when you submit data to us in that form that You register, you sign up to that, that your data can be used by anyone. We are we are not police officers, we will not go around the world and say they've used this metabolites study to develop nerve agent. Hopefully, people will do bad things. Hopefully not, but some people do. But our mission is to make it fully available for everyone to use.	
Interview 19	1:40:30	And we're completely agnostic about who that is there, except for that clinical data that I referred to earlier. So that's when you come back to the ethics community. We have those committees, I don't make the decision who has access to the data, the access committee decides, and then they're responsible for what that researcher does with that data.	
Interview 19	1:41:53	That's separate, but everything that has been agreed to go public. Everyone gets Us.	
Interview 19	1:42:02		One special aspect about metabolites is also that it's within the scientific community. So how do you think that's the scientific culture influence the use of the platform?
Interview 19		So that's a very important question. And that is actually changing quite a lot. So because again, metabolomics is quite odd from us the resources here, because it has traditionally come out of chemistry, which as I said, is one of the oldest industries. So there was not a concept of sharing. So actually, sharing now is a big challenge for a lot of the people who have traditionally done this because they didn't share the data.	
Interview 19		So they're going through cultural mindset, and they're going in the right way. But then there is still a challenge that many of them don't one, particularly the companies don't want to share the full details of the protocols, because that's their IP. And that's something I have to manage. And I'm in discussions about how to do that. Some are fine. Others they don't want, we have to find a way to say this is patentable. This is trade markable.	
Interview 19		And, but I don't fully have a solution for that right now. What I'm seeing, surprisingly, in genomics, which has been around for such a long time, is that people are very happy. Now, once we got over the initial blip, where company patented human gene, and that was never allowed to happen again. People are now a few. I'm not encouraging this. But if you Go on Twitter, look up research parasite, as a term.	

Interview 19		And there's a lot of people out there in the world who do really good science, who publish it, but want to control how other people use it. They want to keep it for themselves, even though they're funded, not by companies, but by governments, and funding agencies. They still want to say, this is my data. I want to make the next breakthrough. I want to build my career with the next paper. I don't want other researchers benefiting from my experiments.	
Interview 19	1:42:16	And I think that's really, really sad. Myself, but I've spent my whole life in open data. So and I'm probably biased, but I think it's a real problem when they have been funded publicly to take this attitude. I can understand for company does it because they invested a lot of money that no one gave them. They had to raise that blah, blah, blah. I still think they should share more than they do. But I understand. But when you are publicly funded, you should not be putting these barriers around the data is people's tax money that paid for this?	
Interview 19	1:45:14		So coming to the two last questions, I was just having a look at the clock, but it is so Amazing with you. It's just great. What development Do you see for metabolites in the next 10 years?
Interview 19	1:45:31	their connection with the omics.	
Interview 19	1:45:35	And that has to be across, and it won't just be in human health, but it will also be in agriculture, it will be in climate change. It will be across the board, and any marine biology, checking our oceans, all of this as Metabolomics it's just people don't actually understand how powerful it is. It's Not a buzzword like genomics, the world is going to learn what this really means every now is getting really excited about your microbiome, all those little bugs in your tummy. And that's a sexy term right now. But metabolomics is completely behind that.	
Interview 19	1:46:19	But actually, we have to collect enough data, we have to evolve it so that it's cheaper to do. We have to get this concept of the personal metabolome because that will drive all the other developments.	
Interview 19	1:46:33		And what does the success of metabolites depends on then?
Interview 19		Today tough. We have to keep evolving with the data and with the community and making sure that everyone has access to it in the most easy, accessible way you'll have heard of fair. That's it, and I'm completely committed to that. I'm quite passionate about it again. This has been my entire career, I've been very, very lucky to work in a place where our mission is always to be freely available. And you Scott, not just freely available, useful is Mikey, but that I don't get to decide what's useful.	
Interview 19		And that's where I'm always always always working with the community, but also not just your community. And that's where I think is so powerful to work in a place like the epi, because I hear from my colleagues what they're doing in their fields. And I can see how this can connect in. And that's the other thing about science and you've already shown yourself to be very open minded by your career choices.	

Interview 19	1:46:39	You have to keep an eye on what's going on everywhere else, because it's only then you will see how your field fits in. And we are all interconnected. Every human every organism on this planet is interconnected and on Last week connect up the science. The danger of unforeseen consequences is what climate change is right now. We thought we were making the planet a better place. by exploiting all this coal and stuff. Actually, we've we've helped destroy parts of the planet. Understanding how it all fits together in an ecosystem is the way that we can avoid unintended consequences.	
Interview 19	1:48:32		Nice. I am so impressed with this interview. It's, it's amazing. I really, really, really enjoyed it seriously. So do you have anything else you want to tell me about success factors of a platform or
Interview 20	Time	Interviewee 20	Interviewer
Interview 20	5:53		And what is that so what is the history behind (platform) ? You already mentioned the project.
Interview 20	6:00	Yes, so the	
Interview 20		the, there's two researchers who kind of came up with this idea in the first place. One is (name) was the director of. So the institute changed from(institute), this past year to maximum Institute for animal behavior. And so he's the director of a department of migration there. And so he has been working for many decades on on animal movements and migrations, and then (name), who's the director of the biodiversity group at the Museum (name)s, in (town), in (country).	
Interview 20		And and so they have known each other for a very long time and they had this idea of we need, people aren't collecting all these great data and going back many decades, with some of the earlier ones radio telemetry, but there is not a good place to manage it, lots of people are still just using their Excel files. It's not well managed and there's definitely will all be lost on everybody's hard drives. And so they wanted to make a very open database that could be used that was kind of above the not just for a certain country, not just for a certain region, not just for a certain species or a certain method that anybody could use as much as possible.	
Interview 20	6:03	And to enable sharing collaboration and and help people manage their data. So that's how it started. That was kind of before I was part of it. And then it's really I think, hit very, there's a big need for it. So so it's really been growing very, very fast. Also, as the technology advances, the resolution becomes higher, the data volumes become higher. So it's a it's a challenge to keep up with From an infrastructure side, yeah.	
Interview 20	8:04		And what is the core offering of (platform) ?

Interview 20	Okay, so for a user, we and so it's an open data online data platform. We, that kind of it supports the use of data from the original data collection. So we have offered data feeds. So you can begin to feed the data to (platform) , even before you deploy it on an animal wherever you are still testing the tag. You can be sending it to study and moving and making sure it's working. You can deploy it on an animal and then come back and add all the information manage what they would call it capture or deployment information, what species it's on, when did you put it on what was the field site measurements you took of the animal as you're putting the tag on.	
Interview 20	So you can manage it from the from the very beginning and then as the data are coming in there issues that come up so, so once you find a date that an animal has died, okay, you can say you can give the endpoint. So the tag might keep collecting data even once the animals died, but you don't want to analyze it. So you can create the end point there. If there we help manage outliers. So you get even with the GPS, you can get locations that are not correct. And you want to mark those. So helping deal with data quality issues, filtering and things. And then once so then you've got it in there.	
Interview 20	We offer a, as I said, a data repository. So the actual publication of data so there you get a persistent link, a DUI you get a citation so people can formally cite you in their papers. You can put it on your CV, which helps people get credit for for their work. Also. The journals and funders are more often requiring this now.	
Interview 20	So let's them need to give them a useful way to meet this requirement. And if they're already using (platform) anyways, it's quite a seamless next step to just go ahead and have it published, then you also get their state licenses with those, so it helps for so then for the very end stage would be reuse data reuse.	
Interview 20	And so facilitating data reuse by helping people discover the data, allowing data to be public. And then also allowing someone to contact it owner of a data so find the description of this study, say, Oh, I would like to use this. And then if they have not made it public, they can you can contact them and ask.	
Interview 20	And so as a combination, this lets people be interacting and sharing and collaborating with data before they're at the stage to make it public, which I think is really important. As we have changes happening, your studies going on for 20 years, and changes that are happening within those 20 years where we need to be finding about them and sharing them before they may be fully public, as soon as possible. So then as far as analysis tools, so we have an API that allows people also to build automated tools to log in, look for data, pull the data and do things with it.	

Interview 20		So there are analysis tools that are developed around being able to query data for (platform) ing access data. So in this field, the most commonly used analysis software would be R and so there is an R package that can facilitate this and then other our packages building on this. So there's like a, an animation, some animation tools that have been built and so people can build tools freely that that will Work with its data format.	
Interview 20		We have published the vocabulary. So now this is a recent thing. So people so it creates a persistent link to an attribute defining that attribute that could can also be used by other people. So we've also had people publish data in the (platform) format in other repositories, but they can refer back to these persistent links to define what their data are. And then another I guess, a key analysis tool we have is a product a system called the end data.	
Interview 20	8:08	And what what this does is we have built links to many different remote sensing data products. So for example from weather models from a satellite imagery products, such as the green greenness index so measure of vegetation productivity,	
Interview 20		Rainfall models. And so often what happened to somebody wants to ask a question they want to know, does the weather affect a birds decision to start on migration? Or does the availability of food via the greenness, the vegetation productivity, will that allow them to initiate a migration or define to help them decide whether to stop along the way for a while, and how long to stop and where to find the breathing site in the end or?	
Interview 20		And so the questions are straightforward, it makes sense. But it can be very difficult to actually get the data to answer the question. And so we pull in these global data sets, and you can actually submit have them annotated so you can have the rainfall estimate or the temperature estimate or the greenness estimate added to every row of your data file and just delivered to you with the original Me file because the other trick with these these products is they can be very difficult to find. What are the units of what you just gave me? What does this mean? Because they're more they're not.	
Interview 20	13:01	They're more designed really for remote sensing experts or weather modelers or climate modelers. And so sometimes movement ecologists or ecologist don't know all the different terms around the weather model. And so we help provide this kind of information so they can understand what it is they've been given. So that would be one of the other kind of larger features that we offer.	
Interview 20	14:34	And those features are there	
Interview 20	14:36	on top of the platform.	
Interview 20	14:39	Those are also everything is within the platform with exception of these external program apps and tools that are pulling from (platform) so we have like a there's a mobile app, and those I would say are separate from the core platform. But the platform is enabling them.	

Interview 20	15:04		And do you have other or more functionalities I should know about?
Interview 20	15:10	I would say those are the main	
Interview 20	15:15	those are really the main ones. Okay. Yeah.	
Interview 20	15:19		So my work now is about success and our success factors. So what is successful you are a successful platform.
Interview 20	15:33	So I'm in every day continue to use and continue to grow and maintain maintaining relevance in fields that are really changing quite quickly.	
Interview 20		I guess one we've been working on lately is is also there's the, okay so people are using it so people are talking about it, people aren't going to give presentations talking about it. The usage is going up, the data volume is going up even faster. And we also have a set of goals that have been basically the same goal since the very beginning. And one of those is how making sure these data stay available in the future. And so that's the one we've been working on the last past year because we noticed most of the data are staying private, which is fine when we researchers active	
Interview 20		However, once they stop being active, they may not remember to go back to (platform) and make the data public or or put give it to pass it on to somebody else. And so we need some more ways to facilitate and encourage that because from the very beginning, we also allow allowed private data, which gives us a lot of benefits. And I think a lot of people would never I don't know moving that ever would have grown if we hadn't offered this very flexible way for users to be to be using moving from the very beginning when they would not have made it public.	
Interview 20		When the public would not have been able to understand it, and so, so it's kind of some kind of a competing thing of, we can't require the data to be public, because we want this flexibility. But we wanted to go public at the end. And then also, how do we curate in the long term access to data that should not be made public for perhaps sensitive species or their data owned by organizations that may have legal or other administrative prohibitions on on making data public? And so how can we help maintain access so that somebody can address it?	
Interview 20	15:48	If someone wants to use the data? Who do they go to make sure they will get a response, make sure they can use the data to, you know, reasonable request. And so that's, I would say another feature of successes that we we can get to where we are growing the num proportion of data that are accessible and that they remain accessible over time.	
Interview 20	18:01	And how do you solve this question you just said with those special species,	
Interview 20	18:10	and for example.	

Interview 20		I mean, the main thing is that we allow people to share the data with individuals. And so that core feature is there that ability to not share it publicly, but share it with this person who contacted me or a collaborator and share the discovery of the data without having to share the complete data. So that's been there from the beginning, we are actually working in sometime very soon in the next weeks, we will be releasing some updates that will, that are to address this so so why is we make it more difficult because we also allow studies to be completely hidden.	
Interview 20		And what we are going to do is have people define their study as a test study, which is a private, or a research study, and the research study the public can see. They can still restrict the data access, but they can at least see it exists and contact the user. And so this is a, I think will change the default behavior of the user to if they're really creating a real study, they want to keep their people will be able to find it unless they asked us for an exemption for some reason if they don't even think that's safe. And then they have the test to study which lets them do their kind of scratchpad type work.	
Interview 20		And those are eventually deleted. So that also accommodates like I just want to see if I can use it I don't know if I want to do this. I just trying it see what happens. And then we also are adding some embargo options. So you can do be able to choose a date later to make the data public up to 10 years later. And so for a long term, this is maybe I think a lot of people are comfortable saying, Oh, I will make the data public in 10 years, when they are not comfortable saying I'll do it next week. So and it will, they'll have to think about this at the very beginning.	
Interview 20		So So we think that will get more people encouraged encouraged to to move in that direction. That also we will have an option to share the data but hide more recent data. So in the case of an endangered species, in some cases, we have had people tell us, I would be happy to share the data publicly after if it is 30 days old. I don't want them to see where the animals are right now. And so we allow them to kind of mask just the more recent data and as time goes forward, this, this window will move so and in addition will allow for data that are pop can be downloaded publicly, they will have Creative Commons licenses.	
Interview 20	18:16	So this helps the user so we have some larger conservation groups that are trying to acquire data through (platform) for large conservation projects. And so we find that those licenses help clarify how the data may be reused, and would be really helpful. And so, we've also been working with some kind of conservation applications and seeing how their experience is with dealing with the users to get access and what questions they had or requests they had to make it work more smoothly. I	
Interview 20	21:54		just interrupted you with the success factor part. Do you have other more Success Factors.

Interview 20	22:02	And I would say so one thing is that it can to some degree run on its own. I think for most databases, there is not expect it. At least if your user group is large. We cannot help every person individually with every question. It must be something where they can do much of the work themselves. And we have a contact point where the questions happen.	
Interview 20	22:33	And I find it so the US space has grown a lot those entire time but we still manage with about the same amount of support and effort. So this is good when I hear from people that they created all this all these studies, they organize them, they share them and I never heard of them. This is very good means they're working on their own. So allowing the independence of the users allows us to scale the database without having to scale our staff at the same rate, because this becomes difficult for most platforms like this.	
Interview 20	23:15		So, you just mentioned various goals you have. So when it comes to the bigger picture, what vision Are you pursuing?
Interview 20	23:25	Um, I would say, let me, so the	
Interview 20		the leaders of move back so so (name), I would say that cut so in addition to what I said is really making the data accessible discoverable. Getting people talking with each other working with The data to do different kinds of things so that the data can have, you know, multiple lives. And allowing, supporting like citizen science classroom groups being able to do project with data from (platform) . There are also a kind of a larger vision is this idea of allowing animals to speak for themselves. And, and creating a place to kind of take a pulse of life on the planet.	
Interview 20		From the perspective that is somewhat more separated from the human perspective. Of course, we're influencing we're putting the tags on animals are choosing where to go, we're getting the funding to do that, or not getting the funding to do that. So, so the human is not taken out of it.	
Interview 20	23:42	But these animals can tell us where they're going. They can tell us how they're changing, if we can follow them. So So this idea of, of this kind of visibility of life and movement in ways that that we can't currently Do and so this is what the technology developments making the tech smaller, cheaper and finding other great new methods for how we can track them globally	
Interview 20	25:14	is all kind of a piece of that.	
Interview 20	25:17		So my work is more or less focusing on also the organizational perspective behind (platform) . So how is the organizational structure behind it?

Interview 20	25:31	So that's a good question I would say and this might differ. So I could also kind of compare moving to some other appear databases where I work I talk with people kind of in my role of other databases differently. We do not have in the same way a government mandate either for the users are typically not mandated and and we You don't have a map like a funding mandates that enables, you know, ongoing funding. So, so there's other databases where, you know, they're supporting data archiving for (region), for the (region) for (country).	
Interview 20	26:18	And so they have a bunch of kind of	
Interview 20		rules that are defining how they grow based on, you know, a legal you know, some kind of law that's regulating why they're there. And we also then don't have, in some cases, you know, government agency, everybody collecting data in that country may be required to archive their data there. So they, this is an easy way to have a reason to be there. So what we have to this is our job is to do the data, whereas ours is more volunteer.	
Interview 20		(name), the people who are contributing are more or less volunteers, and we're Offering all these services I mentioned as as enticements and encouragement. But typically, in most cases it someone's not their boss or their their employer or their organization is not requiring them to use it. So that changes the the infrastructure bit. It's less hierarchical. So we have kind of, there's the researchers who are getting grants, getting funding. And so there's kind of like, there's (name) and roll into I've mentioned and then there's (name), who's a meteorologist at The (country) University and he is the one who has brought in some grants, building the funding for this the pulling and the environmental data and data system and, and so they're bringing in money, and they're also the ones who are kind of they travel a lot.	
Interview 20		They talk with us You know, they're kind of at the top of their field, they're talking with other researchers a lot. And so they're doing a lot of kind of Outreach at the, at the academic level or at the government levels, where they're presenting about what they're doing, showing that that (platform) is relevant at meetings with the space agencies with the public conservation groups.	
Interview 20		So So that's kind of a one level and then there's, there's the developers, and people like me one thing we are very, like geographically distributed, which I keep seeing actually, at first, I felt like I was very alone and being in this project where I'm in (town). There you have the PIs in (country)	
Interview 20		The developers also in (country), but kind of spread out and as I as time goes forward, I find more people who are doing something similar where Yes, our group is Spread out because you want to find the right people, and they might not be in the same place. And for a global project like this, it's I'm mostly working with people that are far away from me anyways.	

Interview 20		So it's going to, I need an internet connection, I need to email and otherwise it doesn't so much matter where I am, especially we have some overlap of time between (region) and the (country) works quite nicely. So. So geographically, it's a bit fuzzy, and then there's been Yes, so the host really the core host really is the (Institute) for animal behavior.	
Interview 20	26:22	So that kind of creates a core kind of that sort of the heart of it lies and most of the funding and, and the so that kind of grounds it and then there's a user base since this organization is also where some of the top like state of the art technology is coming out. being used. They also are a good core base of users. So often the developments are driven by what's happening at that at the (Institute). But yet, that's what the other users are going to be needing in a year or two anyway, so so it's a good kind of development hub.	
Interview 20	30:23		So how many people work for (platform) altogether?
Interview 20	30:28	So. so you have the PIs who aren't currently working for (platform) , but it's kind of a big piece of their research. And so it's coming up in their throughout their careers and their interactions. Then there's myself full time as a data curator, we have another part time. And then it's probably around two full time developers, so it's quite small, and as far as people who are devoted directly on (platform) . For most of their efforts, and then there's other groups. So for example we have there's groups that will work like in and out. So so we get a grant for	
Interview 20	31:18	to cover staff at you at the University of constants for two or three years. And so that so there's kind of like the contemporary people who are working on specific projects. So that was how I started. And so there are sushi right now we have several other people working on specific projects related to (platform) related to the repository, sensor development, and students that are working on projects with that so	
Interview 20	31:51	yes, I don't know what competencies do those people have working for (platform) so I mean, there's the developers who have the, you know, database development, web development. They also most of them actually have a biology background. Several of them have PhDs in biology from (country). And let's see, yeah, so the other character I'm working with her backgrounds in in zoology and biology. So most of it would be environmental sciences and Life Sciences.	
Interview 20	32:32		And how do you distribute the responsibility between?
Interview 20	32:39	Yeah, again, it's not very hierarchical. So there would be that. So (name)i would, I guess, be probably the main person who we're going to go to he's providing most of the funding he's he's the main person to go to as far as making financing related decisions or	

Interview 20		Um, to the degree there's like a top-down, but then there's a lot of like lateral management which can be a challenge but I also think that for a group this small, it's, it's effective and we get things done quickly enough. And and sometimes I imagine it would be really great to have like a project leader and in this room and a project leader in this group and have like, a more formalized meeting structure, but then if you like, but then everything would take longer.	
Interview 20		So, so it's Yeah, it's it's there's a lot of like, lateral kind of, of communications and planning and working together. With with and then I'm you I'm often some I might be making the call at like these new policy decisions about creating these new embargoes and allies. Soon, I mean that we're going to have it more more clear data policy and, and privacy policy in these things. So that was mostly led by me, I, you know, and I reach out to everybody to get feedback and input both, both from our core like our staff, but then also from I know who the users are.	
Interview 20	33:02	So I can select, like some key users who I know will provide really valuable feedback about these plans. And so so I'm often doing that kind of liaison ruled with the kind of policy and and details of the user experience kind of thing. And then, and then the developers, there's also like a small hierarchy there. With the there's a company that's doing most of the development and so they have the owners of the company, and then the people that they will assign tasks to and sometimes they bring in some of their other employees to work on (platform) and they're also working on other projects. So that would be Thursday, that pocket over there as well.	
Interview 20	35:04		You just mentioned the decision process. So just to be clear, so when it comes to decision and you getting several feedback, and then are you working with consensus or how are you proceeding?
Interview 20	35:20	Yeah, that's a good question. I don't think we've we don't have written down anywhere how our decision making works, I would say largely with the PIs and myself as far as kind of the the managerial and, and I can say, I would say it's, it's nearly a consensus type, but we all we are all quite on the same page for most things. So. So works really I don't know what happens if we really had a disagreement. I doubt but Because we are all kind of have a similar vision, I think it is sometimes, you know, there are some different ideas, but always with the discussion, we end up essentially with a consensus, I would say.	
Interview 20	36:15	And I think maybe that's part of having a small group	
Interview 20	36:20	that's been working together for a long time. And	
Interview 20	36:25	so, yeah, I would say it's similar to a consensus type model. Generally, if it if it has to come down, it would be the Pis making choices. And then you know, in some cases, our decisions are what we're working on is defined by the funding right? So you get a grant to pay for this. This is what you're going to be focusing some time on so so they the funders have kind of an indirect role in defining our priority list.	

Interview 20	36:54		So what are the challenges and difficulties in managing (platform) ?
Interview 20		Um, so kind of some of what I said there's kind of this distributed, you know, having having a bigger group and having a could, I could see the advantages of that you need more money to cover it. So like dealing with it limited limited resources is obviously something that we're all dealing with. For the funding opportunities. One, one, and this if I send you this link, this is kind of what this is focused on is, it's really hard to find funding to sustain existing infrastructure when your research platform so if you don't have this core funding from, you know, we are the ocean agency for this country, and we have money to manage the ocean data for this country. It is hard to find this kind of reliable funding for that.	
Interview 20		Just keeping it running. So so if you're a funding agency, you can get money to build a new database, you can get money to make a new, a new sharing platform, or you get money for research and piece of that will be developing your own platform. But to just keep something going, that's helping people, it doesn't meet the typical requirement for research funding to be doing something novel.	
Interview 20	37:02	And so really, when it's like we need to make these tools stronger to scale with the growth, you know, in data resolution. That's a straightforward development issue that might have some technical challenges or innovation, but it's not a research type innovation. So so. So it to find appropriate funding to even apply for is the challenge I'd say.	
Interview 20	38:55		Just Sorry to interrupt you. So how is the funding currently?
Interview 20	39:00	So the (platform) s society pays for most of it, the kind of the core funding. And then there's we have had we so we've always had that. And then there are like grants that that add pieces on to the funding. So we have fun have had funding from (institute) from the(institute)n from the (instiute) and in (country). From the (insitute). And then we are having recently also starting to get some private funding, there's some foundation funding for conservation stuff.	
Interview 20	39:53	So it's, yeah, it's the core funding coming from (institute) and then and then there's a funny button bernburg So we have a grant with now. And so then kind of keep trying with a, you know, maintaining a few additional grants running. Basically all the time.	
Interview 20	40:12		Yeah. Good. Thank you. So coming back now to the difficulties and challenges. Do you have more?
Interview 20	40:20	Sure. Let's see.	
Interview 20		So yeah, funding part of the kind of logistics for global and there is I would say that I mean, there's a bit there's a challenge and an opportunity and being not being anybody's mandate, but just kind of being this more open system. So you have it there is an issue of feeling like people are co owner in it. It's like people ask is moving open source? Well, not exactly. not open source that we can't send you the code for the database, but it's intended to be an open resource.	

Interview 20		And, and so finding the the amount of and as it grows, the amount of value we're providing to users is getting quite. Oh, there's another link, I can send you to a paper that that that (name) wrote enrolling, just put out that kind of get out. This is whose responsibility is it to keep these data? I mean, we're kind of doing it but but this would be traditionally something like what a museum would do, you know, maintaining specimens.	
Interview 20		And being the source of the public outreach, making exhibits. This is traditionally what would museums would do, but right now they're not really taking on that responsibility. And then the users are typically not paying for what is it becoming a really substantial data storage and transfer and maintenance. service. And they're used to not paying for it. And so how do we get some kind of payment model where, where there is a real it's where one organization is not kind of taking on the role for for everybody, both because, you know, and then having a more collaborative funding method would also then give, give the user some more ownership.	
Interview 20		So there's also that the users will ask for some new tool. But as we are limited in time and resources to implement the tool, and so they don't get the tool that they wanted, whereas if if they were a contributor, somehow, maybe there would be the resources to do that. We certainly like prioritize, you know, we keep hearing the same request for a long time. These policy changes are partly you know, things we've heard for a long time, which is I would like I would like to share the older data and Not the newer data, you know, this is something we've heard.	
Interview 20	40:23	And so I brought you bring them in, we certainly care about about the user feedback and requests. But we certainly can't get to everything always at the same time as quickly as, as they could in their own organization. Because for them, it's a priority. Yeah. So um, yeah, I think finding some more collaborative funding model is an important long term goal.	
Interview 20	43:29		And coming also to other goals. What specific goals do you want to achieve now? On a short term range?
Interview 20		Okay, so first, I want to release these changes that I was telling you about that we were like 99% finished shooting the testing. So it's very close, but so get those out. And then there will be probably I would say, like a one year process of just starting to work with those changes. So those changes will let us start to, for example, evaluate what is in the completely private studies and start encouraging people to either move them into the public discoverability or some of them are probably not needed anymore.	
Interview 20		And so some people, we can delete it if you don't want it is a seven to nine abuse and clean up. And then doing outreach, telling people about about some of the new features and documentation. And so some of that will be kind of curation projects. One of the new pieces is that a notification system so that we could efficient efficiently for example, identify all the owners of studies with a certain quality issue and contact them with a notification that tells them how to fix it.	

Interview 20		And so that we should be much more efficient and being able to do those kind of things. So yeah, we're just like creating a list now of like, once these changes are real, what, you know, what can we do to to help get it get more, we also will see what feedback we get. So the making the study summary public?	
Interview 20		Will the people will people will be fine with it once it's the default, or will they get upset and contact us and need an excuse and tell us they will delete all their data? I don't know. So so it'll be some like, see seeing what the responses.	
Interview 20		So far everybody we've spoken with is very excited about it. And we are also at a stage where we have enough users that and we also have a precedent. So a lot of our peer databases were kind of following. So we started in like (year/number) when there was Creative Commons licenses, I don't remember if they existed, but there were much less prevalent, the data sharing requirements were much less prevalent, and so on.	
Interview 20		So new databases that are starting have, they're saying, hey, if you want to be part of this, you will make the data public, eventually you will do that, you know, they're they're kind of making more demands on the users than we had the ability to at the beginning, because we really needed people to buy into the concept. And so now we can start to adopt some of these rules that that newer databases are having from the beginning. And we can refer to them we can say, Listen, if you're going to doubt make the data publicly downloadable use one of these licenses, this is what all this is what all these other biodiversity and tracking databases and ecology databases are doing so so we hope that most people are going to just they're kind of already used to it in other domains.	
Interview 20		So I think should go smoothly and most of the response will be positive. But so the next year will be kind of Yeah, leveraging this new opportunities we have with the changes that we've made. We also need to build the so I mentioned we offer date live data feeds like automated data feeds from different providers, which is quite tedious because there's no standardization in the data from these different providers.	
Interview 20		And even within one company, they, they're unlike this where we know basically, we all want this, you know, we all have the same size pocket about the same, and it's quite straightforward. And so you can build a sensor that goes in this that will do the same thing on every phone for every person.	
Interview 20	43:41	Well, for animals, it's quite trickier, it's trickier and they they want to take off the tag. They have different ways that you know how you attach the tag, what type of thing is attached to them that's going to minimally impact them and be minimally annoying to them and they will be minimally likely to drop it or rip it off.	

Interview 20		Or, you know, animals that are spending parts of their time in trees or in holes where a GPS cannot send a signal or a dirt that are very different sizes from the babbu, mit to Finch are, are, they must be much more flexible with the technology and the sensors and how they work. And so there is you can understand why it's not standardized. But so we've had a lot of requests for more companies to be sending the data.	
Interview 20	47:54	So so we're, that's another big goal of this year. Right now, most of the companies that for some reason I have found the companies that sends the data most easy to implement as a feed or tagging birds. And the large mammal like the large terrestrial mammal callers, somehow it's much more complicated. And so we're working with these companies and I would love to see this year one of the big terrestrial mammal Color companies start to have live feed. And I think a lot of people would be really excited if we can get that happening.	
Interview 20	49:12		You just mentioned also that you have like this vision in your mind. So how do you want to set a strategic position
Interview 20	49:27		for (platform) ? Or how do you strategically position (platform) bank on the data market?
Interview 20		Well, I think I think both of the both of the things I talked about just now so so having the kind of policy things to to encourage us encourage standard ization, encouraged discoverability. And then these these data feeds are kind of critical because It's there are still be the old data sets. So they older data rescue is kind of one of my middle things that I love because it's so it's so exciting to talk about the new stuff. But if we want a 50 year period of record, we must care about those, the radio tracking studies that were done in the 60s, because here we can get a real period of record.	
Interview 20		And so those are stored on the Excel files. They're very small, you know. And so the challenge of getting those is a totally different look for the new stuff we really needed coming in right away is that as I mentioned, this idea of like having a pulse of life on the planet, looking for triggers of tipping points or triggers of changes where we might we might want to notice when a migration shifts somehow differently for the first time or even concept of using animal movements to detect natural disasters. ahead of time. And so there we need this, we need as much as possible the data coming in automatically to the users.	

Interview 20		This also is great for data quality because we already talked with the data provider. We know that time zone in these things so there's fewer chances for user error also. So it has many benefits, but but getting those live feeds. Working at a big piece of like a sibling of (platform) would be the (name) project. And so this is what (name) (name) is working together a send the testing phase now but so there is a system on the International Space Station where you can take tags and relay messages from tags using the International Space Station, which is quite cool. One of the most popular satellites methods for tracking Is it are those satellites so they're polar orbiting satellites, two polar orbiting satellites.	
Interview 20		But they're quite high, the International Space Station is at about half of the altitude. So you don't have to send the messages far, which means you can have like smaller battery. And there's so there's that coming up. There's also an Argos and some other companies are experimenting with like micro satellites, where they could also be lower to the ground and require less power to transmit messages and be have more time more Passover. So pass over the tag more times per day.	
Interview 20		To get more estimates or more options for if you need, you might need to schedule a GPS to transmit during the time of day when you know the animal is going to be if it's nocturnal, you may need to send it at 2am. You wanted to send the message and so if the if the satellites coming more times, it gives you more chances To transmit the data with minimal power usage.	
Interview 20	49:37	So there's a lot of kind of exciting developments happening with how do we transmit these global movements and we need to be ready to have these feeds to bring them in automatically because its users love it, it's much easier for them is better for data quality. And it gives us this opportunity to, to have this post and be seeing things as they're happening.	
Interview 20	53:28		So how do you describe the user group? because you were just mentioned the user love it.
Interview 20		So it's really quite global. There's probably you know, probably a bias for you and and (region). Although those researchers are often researchers in the (region), and researchers in (region) often from other countries and doing research in other countries, so I think that maybe hard at Two parts out and then one would think. But so I think it's quite International. There's a huge range of users from like, kind of old like old school.	
Interview 20		You know biologists field biologists who are kind of have retired the earning or AB retire past retirement age now who are like storytellers and and know so much from having spent so many decades in the field and have meticulous records of the populations they've worked with. lower resolution data but it goes back a long time and it tells really amazing you know, has amazing information in it. But not technically savvy.	

Interview 20		To then you have younger people who are coming on it, they want to do everything and are they don't even go to the moon fake website. They just are going in our and then you have to work with Their data to run a workshop with their data during their postdoc. Then you have also what's one thing I'm starting to see more of is the data users. So the people who are interested in like big biodiversity questions, and how do we get our conservation? big scale conservation question.	
Interview 20		So the (institute) which is big. It's a nonprofit, they, they're like birds in (region), like Audubon is is a big thing, both for outreach, public outreach, but data collection, they're really big group and they are starting a whole program that's centered on building atlases for long term how all the bird species in North and (region) are migrating over time, and they're running it entirely through (platform) . And so I've been they have like 15 people on their staff, and they are like doing all this recruiting. And so I've been working very closely with them.	
Interview 20		And so sorry, To see more people who are are willing to rely on (platform) as their platform rather than building a new platform, and when we can work together, it's like it's been really exciting because they bring all these new resources and ideas. And then I can see what I imagined happening. Now it's happening because one thing I found is that the users or potential users, they need a reason to make the data public or put the date on moving it so when someone they can I can't just say, Hey, I saw your nice paper, it's great would you like put the date on move, but they don't have time.	
Interview 20		But if someone says, Hey, we're going to do the great conservation tool, if you will contribute, if you need help, but we you need to put the date on moving and then share it with us. This is enough of an incentive for people to really get excited and start using or sharing. And so seeing what I would have been hoping to see for a long time I'm starting to see more of that. You're also starting to see people who are interested in like movement trajectories in general that I don't really care if it's an airplane or an animal or a phone and somebody, you know, what we're all walking around doing or on the bus or whatever.	
Interview 20	53:35	So they. So that's a whole nother group, and making sure the data are understandable to them and not making trying to minimize assumptions about knowledge of, of this field, in potential data users, so the, I'd say the fields getting bigger of users,	
Interview 20	57:32		so their scientific background is mostly kind of biology and so on.
Interview 20	57:38	Yeah, definitely. Yeah. And the data, there's also like data owners and data users, so data owners, yes, absolutely. biology, ecology, all different ages, and career stages.	
Interview 20	57:54		And in the future, do you want to broaden the community size or do you want to have Intense community usage of (platform) .

Interview 20		Good question. I, I would say mostly we're going for broadening the size and getting more. because ideally, a user does not actually have to spend so much time in moving. They make their studies, they either upload their old data or they start up a feed, they come back occasionally to share with somebody or to to mark down that the animals which animals have died in the last few months to go ahead and do that to to make the next step to annotate some environmental information or to publish it.	
Interview 20		But they are goal is not for people to spend so much time on moving, but that that is where their data lie. And then they're going in and out of it with other tools. So it's a more it's getting, getting more users making sure that as new People are starting their careers that they're using.	
Interview 20	58:07	They're using (platform) from the beginning, so as easy as possible, and then bringing in more, helping more of these groups like Audubon, who want to query and and, and build their own kind of networks and tools that are just using (platform) as the data platform and discovery platform, and then doing their own thing with it. So,	
Interview 20	59:31		what difficulties can arise when exchanging that via (platform) ?
Interview 20		So some confusion can happen, as I mentioned, given that when people are putting their data and moving from the very beginning, and so sometimes people they look at the study and they say, why is there GPS points in (region), but it's in (region), and oh, it's just an outline. Well, why didn't the owner there? So there's an expectations thing of people who are expecting to see like the finished, complete, perfect clean data set. And then yeah, there might actually be some stuff in there, that still has to get fixed because they're still collecting the data.	
Interview 20		So there's also, as I mentioned, because for all the, if you want a data feed, you must really what is required to have at the time and the tag, it doesn't have to be on an animal. So to explain to somebody who's not doing the data collection, Oh, those points were just, they were from before the tag was put on, they were testing it, or it was after the animal died, and they're like, Why are they here? And so, um, so what I mean, in the data structure, it is very clear, every data row has an animal ID, and a tag ID and a time and a location or whatever the sensor measurement is, and if there is no animal Id just is not an animal.	
Interview 20		Or else you must ask the owner if they made a mistake or did not add the animal. Yes. So so there can be some confusion of people who are expecting it to be just completed full, cleaned up data sets. And so it would be, yeah, which data are associated with an animal and what is the species of the animal. But so, and one thing I've had to learn is with the the API documentation is make sure that is all explained right there because some people as I mentioned, like they never go to move thing.	

Interview 20	59:39	They don't realize that we are storing all these other details about which are males and females, which ones were juveniles, which ones were adults, it's all there. And so making sure that people who are only reading the API documentation can also find this key information. It's also something I built into The data policy User Agreement and Terms of Use that we have written is make sure those really key features are very clear. So any person downloading data for the first time read it, hopefully, also keep it short, very readable to a premium to the most common confusions.	
Interview 20	1:02:22		And how do you think is the reusability of the data?
Interview 20		It's good question. I think we have so we have a grant right now, if I'm working with some people at University of (town) to get our repository certified with this core trust seal certification for repositories. And this is really focused on the accessibility of reusability. I think, because we have our own standard attributes. This units are consistent We only have an API is really quite reusable. What it seems to be the biggest challenge is to work with if you want hundreds of data sets to contribute to something, it's really the communication with the data owners, that is the hardest part and get them to participate.	
Interview 20		And there are some limitations in that. And how do you attribute this many people? Do you have an author line with 300 people on it? The journals often are restricting how many references you can include. So if you will, if you are publishing an analysis with 100 datasets, and the maximum number of references for the whole paper, it's 50. And they want you to reuse the data so that the search engines can see the linkages between the data use well you've just so there are some issues I think still need to get.	
Interview 20		Another little note I'd like to write for a journal or something to The journalists find a way for the data to be decided to put you know, don't limit the citation of data if you want to encourage reuse So, so there are some kind of policy things that go outside of (platform) that that could I think, make the reuse work better.	
Interview 20		Another thing I wrote in the the website changes is a citation guidelines, which I realized we did not have. So help users with how they should be citing the data, make it very clear is not moving, not (platform) data, it is somebody data, find out who and how to cite data for there's the straightforward case of I tested my method on one data set from who bank. Okay, this is fine. But for Audubon, if they're building an atlas that's using data from hundreds of people and it's not in a paper, it's on a web application.	
Interview 20		Where do you put the credit what's reasonable? And if you're using a license, and it says you may use the data if you attribute to me, okay, well, where do we put the attribution for different types of media products, then that is traditional. So beyond is a journal article.	

Interview 20		And so trying to provide more guidance for that and trying also now that we're seeing some examples of this happen on (platform) , I'm can be interacting directly with, with the people who are building that and see, talk with them, and then, you know, get their input and see what they're going to do and then, you know, update the citation guidelines as we start to see more examples of this happening.	
Interview 20	1:02:28	So yeah, I think technically we really are great for for enabling reuse. It would be more the making sure that attribution making people comfortable also keep more people will make their data public. Once they see there are clear citation guidelines and they see examples of people giving them a credit for what they've done so so	
Interview 20	1:06:03		imagine I want to upload something into (platform) . So what requirements do you have for the submission of data.
Interview 20		So to just put data on (platform) , really you need some kind of a file with the time and to tag and some kind of measurement. It's okay if the measurement You know, sometimes you have a you are unable to make take the measurements at the time. So even the measurements can be blank we need to time and the tag. And so this allows people to put their own data in be very flexible, but then you must also then to make it usable, add an animal and add a species.	
Interview 20		And so we have different ways to so if you go to download the data, if you have not added the animal and species, then technically it's not required because we have some points lots of points when There is not an animal in the species. But you will not download anything. And you'll get an empty download and say Why? Well, you have no animals. There's no animals, none of the data or to do with an animal. So why would you download it?	
Interview 20		So you can still download it, but you have to push button saying, I would like to include the undeployed data. And so if you look on the map, you will also not see it until you have put an animal so and then we also have the summary of the study is going to show how many animals and it was a none. And then it will show how many records of animals do you have? None. And so there's lots of ways to notice I think I'm not quite done here.	
Interview 20	1:06:13	And so yeah, it's, but it's a, it can be very simple a file with times dates, location, animals. It can be very complicated. You can have multiple tables. So if you're collecting, you can. The other the other requirement is a sensor type. So this would be GPS, radio signals, geolocation, an acceleration.	
Interview 20	1:08:17	What type of a sensor is it has to be assigned. So this lets you store all kinds of different sensor information about the same animals in the same study together and keep it clear.	
Interview 20	1:08:32		Good, and then how do you ensure the quality of the data?
Interview 20	1:08:38	So when I'm, so like I said, there's these little kind of hints at people who are managing their own data to get them get a complete data set. But so for example, we cannot make the assigning a mandatory because sometimes you don't know.	

Interview 20	1:08:58	But when you go to publish the data, I will always have them added if it's available. There's a set of kind of minimum attributes that must be filled out to publish. Also, if it's kind of what I use anytime I just review a study, I know I'm also on the new documentation on the website, I have basically written up the procedure I use for myself, to share with people. So here is our minimum set of suggested information. And more. Yeah, so providing more guidance, because there's like what technically Can you put in here and then what would what will complete it what you should do to prepare it to be reused? So we're putting more guidance is that	
Interview 20	1:09:53		related with the data quality is trust in the data? So how do you ensure that Trust.
Interview 20		And so I think the making it easy for people to contact the owner if they have a question. I'm making it clear whose data it is. Which so you don't go to (platform) and just look at all the data. The data must come is with a study and the study is with the person. So you can always see where that data came from. Having the documentation, being responsive to questions and then having things like a published vocabulary. The idea is to make this as easy as possible for people to know exactly what these each column of the data set should mean. And then trusted is also when you see it.	
Interview 20	1:10:02	When other researchers who I do not work with are publishing a paper with our assessing (platform) with other believers The programs, then this is kind of showing that there is a trust, collectively in the field for this database. And it's been running now for, you know, 1010 years. So I think also people who are in the field have, we've been able to build up trust. And people see that, you know, their data have not been stolen. And (name) is not publishing lots of papers with their data.	
Interview 20		The / Yeah, so having good documentation, being responsive to questions and then trying to make things as clear as possible. So one day sometimes people will want more fancy sharing options so like, I want to share this animal with this this person design with the other person. And I've always said, if you can show me how I can tell you what you have done very clearly I can do this but otherwise you become like Facebook Are you great, so many options and then everybody They can't figure out who they share with what they're confused, the people they're trying to share with are confused and then they get pissed off at you, you are definitely I would be the one in trouble. So.	
Interview 20		So trying to keep the core functionalities, especially for the sharing, really transparent and straightforward. So as which is at the expense of providing more flexibility in this case, because the trust for the sharing is really, really critical. And if we make it too complicated, we can lose that trust because, you know, even if someone must be able to see very quickly who they exactly they have shared with and be confident that it is very clear what they've done so so even adding these embargo option, it was a lot of discussion to make sure we were all clear.	

Interview 20	1:11:30	How do we make exactly clear what we're letting people do? It has been even just to add to it seemingly simple choices. So, so keeping the sharing options.	
Interview 20	1:13:08	Very, very clear and transparent, I think is is pretty key.	
Interview 20	1:13:13		You just mentioned those positive examples to show other researchers. And how do you ensure that scientists use the data scientifically correct?
Interview 20		Yeah, that's a good question. I think having those requirements to make sure. So for example, when we're publishing data, there is a paper that's describing this study, we had to add as much information as possible that they're likely to need. We've defined all the data attributes, we've given you the contact for the person, if you have questions, we have said you really should contact them about reusing the data. They might have more data.	
Interview 20		They will have feedback, you know, so really encouraging the communication. And then I mean, one thing that we have added, and this was also looking at every other database like this in their user agreement, they say, you know, we didn't, your use of this data does not represent our, you know, agreement with how you're using the data, you are responsible to use it correctly. So in the end, we can we can do, we can have, we can do a lot of steps that we cannot guarantee that, that people will reuse the data appropriately. And so, and one thing I saw as I was looking at other people's data policies is they all say that, that, you know, really, if you're using the data, it's your responsibility to make sure you're doing something sensible.	
Interview 20		Here's how you can ask questions to find out. But, yeah, and I have I mean, I haven't seen examples of, of something really, you know, very wrong conclusion being drawn. I think this is also people are worried about this sometimes but they also be published papers. Well, people miss cite papers to you know, they say, I make this statement that's true based on your paper. And people look at that, no, my paper does not say that that's not what my papers so this happens as well. And so all we can do is be as clear as we can, you know, when it's famous with our papers, the bright and very clear abstract and you minimize the chance of somebody misunderstanding your results, but somebody might still misunderstand them.	
Interview 20	1:13:28	So. So I do think a lot about that. But in the end I if we do everything we can to document and to create good best practices and help people talk with each other. And I don't worry about it too much beyond that. I also want like a third grader to be able to download some data and do something and you know, she might get the wrong answer, and that's okay. But she explored the data. It's great. So	
Interview 20	1:15:56		yeah. Coming also to the user. How do you encourage researchers and also institutions or project to use (platform) ?

Interview 20		So what we used to do I mean, I used to like look at research and contact authors of recent papers and invite them. It kind of self, it kind of does it itself now, so I think we must stay relevant when you know, so we have a social media feed, we are different people involved with moving guard giving presentations, I have it.	
Interview 20	1:16:09	I just updated my set of slides, presentation slides. So, because quite often someone will say, Hey, I'm talking about my study, I use (platform) . I would like to give two minutes to talk about how I use (platform) for my research, do you have some slides so I have it always ready. So I have other people who are presenting at different conferences in (country), in in (country) and other places in (country), where they can talk about it, talk about their experience. So, so trying to be as helpful as I can to get other people. And that's even a better recommendation, right is if they're telling what they're doing.	
Interview 20	1:17:11	So, so it's big enough now it's kind of working on its own, I'm finding. Also, if you can invite somebody to either use (platform) share data, or make the data more public, with some very specific project or incentive, you will be on this paper, you will be part of this conservation tool. People are quite responsive, but just asking them to do it is much less effective. So so it's really finding these groups that want us moving and helping them every way I can. And then yeah, we have kind of people, the poi and myself and other people who are kind of out there, you know, in the field. at conferences and making sure that we are presence. So,	
Interview 20	1:18:04		yeah, one special aspect about (platform) is that it's within the scientific community. So how do you think that's the scientific culture influence the use of the platform?
Interview 20		So certainly, yes. So because I think from the beginning, it's very been a researcher driven platform, it is quite different than it might be if it were a public outreach driven platform will be support public outreach, definitely the development is centered around what the researchers need. And I think if we did the other way, the website would be more user friendly, but it would have a lot less data. This is what I suspect so. So, yeah, it means that that, for example, the way we we built We don't have when you look at the tracks on the map I'm moving is not passing, can I change the color to the data, there's all kinds of wonderful visualizations we might be able to offer. However, we have studies with millions of points in them.	
Interview 20		And the traditional ways of showing a trajectory on it within a web application gets really constrained once you have millions of points. And so we first deal with making sure that they can put those millions of points and moving before we, you know, have the data is prioritized over making the most fancy visualization tools.	

Interview 20	1:18:19	Then you have the API and you have other people who make those those tools separately, so making sure we can accommodate the most state of the art. research studies definitely impact how we're spending spending our money in it. Yeah, it means that the website is less user friendly for for a high school student?	
Interview 20	1:20:06		Yeah, sure.
Interview 20	1:20:09		So coming to the two last questions, what development Do you see for (platform) ? In the next 10 years?
Interview 20		Good question. Um, yeah, so there's just like keeping keeping it running is so that the sides of moving doubled last year. And so it's like looking starting to look like an exponential curve. So that's a bit scary. And so it's just having the servers and the storage. We had the database was identified by an agency in Germany, I don't remember that. Basically, the German government has identified it as a like a critical source of data that will be backed up and in (town).	
Interview 20		So there's the geographic backup location. More than 200 kilometers apart or something. So, yeah, I mean, just keeping everything going as it's growing this quickly as a challenge, I think adding adding those data feeds. And then there's Yeah, AP, there's kind of API developments and improving the website interface, the that also need to happen so.	
Interview 20	1:20:19	And in 10 years, I think we would hope that if there is a, a more like, as I mentioned with this kind of collaborative funding that we also have a funding and hosting model, where it's either collaboratively hosted or hosted by a museum where that that has become a piece of that museums. Core kind of duties of what what they're what they're keeping With the protecting and so yeah, I think more collaborative funding mechanisms.	
Interview 20	1:22:08		And what does the success depend on then?
Interview 20	1:22:25	Good question. Finding resources, finding people finding ways to get people to, to take ownership like this to become co owners and finding ways for you know, (institut) then also be willing and able to distribute that ownership.	
Interview 20	1:22:53	And then yeah, I mean, kind of continued outreach and and, and maintaining the relevance of globally. And then, which also is kind of participating being being a piece of what the biodiversity community and other databases having us be one of those, you know, the list of databases that are communicating with each other and trying to coordinate our developments on data standardization and transfer protocols and things.	
Interview 20	1:23:25		Great. Thank you. Do you have anything else you want to tell me about success factors challenges, so whatever comes to your mind?
Interview 20	1:23:35	That's all I've got right now. Yeah.	
Interview 21	Time	Interviewee 21	Interviewer
Interview 21	00:00		
Interview 21	06:47		Und was ist jetzt das Kernangebot von (platform)?

Interview 21		Also das sind diese zwei Sachen. Ich kann nicht sagen, was jetzt der Kern ist. Im Prinzip ist es zum einen das elektronische Laborjournal und das Repositorium für Forschungsdaten. Wobei man sagen muss, dass das immer so ein bisschen - also es variiert, was stärker von Interesse ist.	
Interview 21		Für die Forschenden ist eigentlich das elektronische Laborjournal eher interessant, weil es dann (unv.) Wege bietet, die aktuell - wo der Schuh drückt, ne, dieses Forschungsdatenmanagement im Labor direkt, wobei das da niemand Forschungsdatenmanagement nennt, sondern einfach nur die Dokumentation der Arbeit und (-) ja (unv.) sind eher an uns und auch die Industrie eher an den Datenbanken daran interessiert, also das, was (unv.) nachher zugänglich ist.	
Interview 21	07:41	Und genau, also diese zwei Systeme stehen nebeneinander, sollen eigentlich auch zusammen benutzt werden, damit sie möglich effizient sind. Bis jetzt kenne ich aber keine Gruppe außer uns, die es wirklich systematisch auch parallel nutzt.	
Interview 21	07:53		Welche Vision wird denn damit verfolgt?
Interview 21		Also eigentlich möchten wir - das Ziel der Datenbank ist Unabhängigkeit von kommerziellen Systemen, würde ich mal so grob sagen. Wobei das natürlich ein sehr langfristiges Ziel ist. Für die (Chemiker?) - ich weiß nicht, ob Sie das auch mitbekommen haben - sind ja eigentlich zwei Datenbanken hauptsächlich relevant. Das ist (platform) und die sind halt sehr teuer und der Zugang ist auch sehr eingeschränkt, das merken wir jetzt, wo maschinelles Lernen mehr und mehr ein Thema wird, mit dem sich auch viele Leute beschäftigen.	
Interview 21	08:31	Eigentlich haben wir ja den Zugang zu den Daten gar nicht, den wir brauchen. Das bedeutet, wir hatten lange Zeit - hat es vielen (unv.) ausgereicht, immer den Zugang zu Datenbanken zu haben und dort nach Strukturen und Reaktionen zu suchen und das hat sich ja über die letzten - würd ich mal sagen - Monate oder Jahre gezeigt, dass das eigentlich nicht ausreicht, sondern wir wollen Datensammlungen haben, die wir dann auch genau so exportieren können und möglichst auch den gesamten Datensatz dann für maschinelles Lernen verwenden können.	
Interview 21		Nur ein Beispiel ja, andere Analysemethden natürlich auch. Und da wir den Zugang nicht haben, müsste jemand anfangen, die Initiative zu ergreifen genau das zu entwickeln. Und das war das Ziel. Wir stellen Datenbanken auf die alternativ zu kommerziellen Systemen genutzt werden können, die natürlich die Informationen, die jetzt zum Beispiel für sehr viele Chemiker wichtig sind, wie die Spektren oder die genauen Versuchsvorschriften dann auch enthalten. (--).	

Interview 21	08:58	Genau, das war so das Ziel. Also es ist auch schon eigentlich ein langfristiges Ziel von mir gewesen - ich war früher schon immer sehr frustriert wenn ich keine Spektren gefunden habe und dann sind Daten publiziert, die man eigentlich nicht nachprüfen kann genau, weil die Spektren nicht auffindbar sind, teilweise sind noch nicht mal Kopien der Spektren vorhanden und dann sind die Professoren meistens auch noch emeritiert, dann bekommt man keinen Zugang mehr, auch zu denjenigen, die da auch vielleicht noch Hilfestellung geben könnten und das Problem wollten wir mal grundsätzlich angehen.	
Interview 21	09:55		Nun handelt meine Arbeit ja von Erfolg, beziehungsweise Erfolgsfaktoren, wie definieren Sie denn Erfolg oder eine erfolgreiche Plattform?
Interview 21		Ja, das ist - das kann man glaube ich unterschiedlich sehen. Es ist glaub ich immer so die Frage, wer nutzt die Plattform nach oder wer nutzt die Daten der Plattform nach. Also wer ist passiver User und kann mit den Daten, die eingestellt sind, was anfangen. Das würde ich so als Schritt eins sehen. Darum wird es wahrscheinlich die ersten Nutzer relativ schenll geben, weil die denken, okay, die Datenbank ist, wenn vielleicht auch nicht umfangreich, hat sie vielleicht trotzdem einen sehr speziellen Datensatz, den man sucht und dann auch anwählen kann und vergleichen kann.	
Interview 21		Aber so der Level 2 wäre natürlich, dass die Datenbank, oder dass die (unv.) in dem Fall auch systematisch genutzt wird um Forschungsdaten zu speichern, parallel zu den Publikationen, die (submitted?) werden. Das wäre so unser Ziel. Dass es eine internationale Nutzung der Datenbank gibt.	
Interview 21	10:58	(unv., schlechte Verbindung)… sich daran beteiligen, neue Daten einzustellen. Und, wenn wir - also wenn wir unsere Systeme aktuell ansehen, dann sind wir genau da noch nicht. Also das ist ein Punkt, den haben wir bisher nicht erreicht, weil wir die aktuelle Datenlage immer noch mit Wissenschaftlern generieren, die aus der eigenen Instiution kommen und die internationale Beteiligung ist einfach noch nicht da.	
Interview 21	11:30		Kommen wir mal zu einem ganz anderem Thema. Wie ist der organisatorische Aufbau hinter (platform)?
Interview 21		Bezüglich des Repositoriums oder des elektronischen Laborjournals?	
Interview 21			Des Repositoriums.
Interview 21	11:47	Das Repositorium (--). Mit organisatorisch meinen Sie, wer das betreibt? Und/	
Interview 21			Genau, so die Personalstruktur
Interview 21		Also wir haben / Der Betreiber ist eigentlich das Rechenzentrum. Formal - also die Server stehen bei uns im Rechenzentrum in (town) und werden dort auch, was die Backups betrifft, durch das Rechenzentrum betreut. Und die Organisation, aber eigentlich der Software, das Codemergen, die Updates und solche Dinge, das wird von uns gemacht, also von unsrer Gruppe.	

Interview 21		Wir haben jetzt für das Repositorium - oder hatten zumindest - Förderung von der DFG und in dem Rahmen haben wir die Stelle auch im Haus weitergeführt, es wurden ja auch Nachhaltigkeitsversprechungen von unserer Seite gemacht und die haben wir in dem Maße erfüllt, dass wir den Hauptprogrammierer eigens weiterfinanziert haben und der managed so das organisatorische.	
Interview 21	12:27	Grob geschätzt würde ich mal sagen, für das Repositorium, Updates, Bugfixes, und solche Dinge, aber auch kleine Weiterentwicklungen. Wir supporten das noch mit Entwicklerstunden parallel, wenn es verfügbar ist. Das können wir nicht dauerhaft, nicht ausschließlich machen, aber dennoch, das kommt auch vor. Und dann haben wir noch ein Revue-System hintenan stehen, das ist natürlich das aufwendigste an der ganzen Geschichte, das mache meistens ich.	
Interview 21	13:17	Also das grob ansehen der eingestellten Submissions auf Plausibilität und Vollständigkeit und wir prüfen auch das Formating. Also das mache ich mit Studenten zusammen, also beziehungsweise Doktoranden. Da wechseln wir uns ein bisschen ab, sprechen das intern ab, wer was macht, aber prinzipiell läuft das über unsere Gruppe und das manuelle Prüfen auch der gesamten Einträge.	
Interview 21		Wir machen, also supported ist es ein bisschen durch einen kleinen Automatismus, also dass es gewisse Dinge und für Chemiker übliche Datensätze prüfen wir auch automatisch. Das ist natürlich nur bedingt auf alles gleichermaßen anwendbar. Da haben wir gute Indikatoren, welche Datensätze gut sind, welche schlecht sind, wo Unstimmigkeiten da sind und zu einem gewissen Anteil filtern wir auch die Submissions schon gleich raus, die wirklich vom Datensatz her nicht passend sind, also Unstimmigkeiten aufweisen, die auf jeden Fall offensichtlich sind.	
Interview 21		Aber dennoch ist es nicht so verlässlich als dass man dann sagen kann, man kann komplett auf die manuelle Prüfung verzichten. Es ist eine Kombination.	
Interview 21	14:25	Und das werden wir wahrscheinlich auf Dauer immer mehr in die Richtung der automatisierten Prüfung auch treiben, mit jeder Verbesserung des Codes und der automatischen Modelle, die da hinterlegt sind. Das ist natürlich ein bisschen besser, einfacher und auch treffsicherer. Aber ganz ohne manuelles (unv.) wird es wahrscheinlich nicht gehen. Einfach weil wir die Datenqualität hochhalten wollen und das geht sonst nicht.	
Interview 21			Wie viele Mitarbeiter mit welchen Kompetenzen arbeiten insgesamt für (platform)? Also auch für das Repositorium.
Interview 21		Insgesamt, also ELN und Repositorium sind wir im Moment (-) Sekunde, 5 Entwickler und, also ich als fachliche Betreuerin für das Repositorium, für ELN ist da weniger zu tun plus ein Postdoc, der macht Einweisungen für beide Systeme und trägt auch die Systeme, also stellt sie häufiger vor in Seminaren extern oder auch Vorträgen. Und dann haben wir kontinuierlich 2 bis 3 Doktoranden, die Submissions prüfen und die Qualität mit checken.	

Interview 21	15:41		Und was für Kompetenzen weisen die dann auf?
Interview 21		Also das sind - die Entiwckler sind entweder Softwareentwickler mit einem Engineering-Hintergrund oder tatsächlich Informatiker. Der Leiter der Programmierarbeiten ist Naturwissenschaftler aber hat auch umgeschult auf Informatik und alle anderen sind Chemiker von der Grundausbildung her und bringen eigentlich die Kenntnisse von Chemikern mit um die Daten prüfen zu können. Also mit einer abgeschlossenen Ausbildung dann. Entweder Masterabschluss, wenn es Doktoranden sind oder dann die Doktorarbeit, für denjenigen, den wir als Postdoc einsetzen.	
Interview 21	16:31		Wie sind denn jetzt die unterschiedlichen Verantwortlichkeiten für das (platform) Repositorium verteilt?
Interview 21		Im Prinzip gibt es zwei parallele Organisationsstrukturen, das eine ist der Entwicklungsteil und der wird von unserem Hauptentwickler geleitet und er gibt die - im Prinzip ist er verantwortlich für das Code mergen, was übergreifende Elemente betrifft, also Zusammenführung des Codes, Hauptweiterentwicklungen und natürlich auch die Verteilung der Arbeit auf die einzelnen Leute - wer wo seinen Fokus hat, was die Programmierarbeiten betrifft und dann gibt es den zweiten Teil, die Verteilung des Revue Systems und das ist dann meine Aufgabe. Also alles, was mit Submissions prüfen und Qualitätschecks zu tun hat, das verteile ich.	
Interview 21	17:30		Und wenn jetzt Entscheidungen getroffen werden müssen, wie findet das statt und wie sind dann die Entscheidungswege?
Interview 21		Das ist eigentlich - das sprechen wir mit den Kollegen ab. Die Entscheidung, die wir aktuell treffen müssen, ist ja eigentlich nur, in welche Richtung entwickelt sich das Repositorium weiter, was sind die Hauptfeatures, die dazukommen, beziehungsweise was das Bugfixing angeht, was hat Priorität. Und das sprechen wir eigentlich unter uns ab. Also die Hauptentwickler, ich und dann natürlich unser Chef Stefan Bräse.	
Interview 21		In der Regel ist er aber - also er betreut zwar das Projekt, aber ist natürlich nicht in den einzelnen Bereichen, also was mit welcher Priorität abzuarbeiten ist, nicht so tief drin, dass er das nicht mitentscheidet oder zumindest nur nochmal drüber sieht über die Entscheidungen. Was natürlich aber wesentlich ist, ist dann die Anwendung der zwei Systeme dann auch intern. Also welche Regeln das Repositorium, die vielleicht eher offen sind, sind für unsere Gruppe dann bindend, an was muss sich jeder halten, was ist so das Minimum an Datensätzen, die beigesteuert werden müssen, solche Sachen.	
Interview 21		In welchen Zeiten muss man das Repositorium bedienen und mit Inhalt füllen, wenn Publikationen rausgegangen sind, solche Sachen. Aber eigentlich hat das nichts mit dem Repositorium und der Organisation zu tun, sondern eher den Umgang der Gruppe mit diesem Tool, das verwendet werden kann.	

Interview 21	18:50		Nun haben Sie ja gerade gesagt, dass Sie sich austauschen in die Richtung, in die das weiterentwickelt werden soll. Wie soll den (platform) strategisch positioniert werden?
Interview 21		(platform) ist aktuell sehr eingeschränkt auf den organisch chemischen Bereich und das auch mit gutem Grund. Genau so war das beantragt und sollte entwickelt werden, demnach haben wir das genau so aufgesetzt. Und in Zukunft soll es etwas breiter werden. Also wir haben verschiedene Anwendungsbereiche identifiziert, wo Rückmeldungen der Kollegen kamen, dass sie gerne (platform) nutzen möchten, weil sie in Randbereichen arbeiten oder in Kooperationsprojekten mitarbeiten und aber gewisse Bedarfe haben, die wir noch nicht abdecken können.	
Interview 21	19:34	So zum Beispiel in der Polymerchemie oder Anorganik und da wissen wir, dass wir ein relativ übersichtliches Featureportfolio haben und dann können Kollegen dazukommen und können ihre Forschungsdaten auch speichern und das ist natürlich bei uns auf der Prioriätenliste sehr hoch, weil wir dann wissen, dass wir weitere Gruppen zur Nutzung gewinnen können.	
Interview 21	19:57		Gerade haben Sie ja schon die Finanzierung angesprochen und dass Sie das ja auch extra das so entwickelt haben, wie Sie es beantragt haben. Wie erfolgt denn die Finanzierung?
Interview 21		Wir hatten DFG Förderung und die ist ausgelaufen dieses Jahr - oder nein letztes Jahr, sorry, wir haben ja schon (year/number) - letztes Jahr ausgelaufen und hatten aber ein Nachfolgeprojekt direkt angestoßen und das ist bei uns das (institute) in (town), es ist also ein Landesprojekt.	
Interview 21		Da ging es genau auch um diese Weiterentwicklung, die ich beschrieben habe. Das heißt eine Weiterentwicklung in andere Anwendungsgebiete und Entwicklung der Randbereiche, das man (unv.).. schlagen kann und das wird jetzt - das heißt Data Science - entwickelt, auch mit Kollegen zusammen. Auch mit Kollegen, die die gleiche Softwarebasis benutzen und darauf aufbauend andere Systeme entwickeln.	
Interview 21	20:56	Also es ist nicht - sagen wir so - es ist, obwohl wir tatsächlich nicht so viele aktive Nutzer haben aktuell, das System wurde ja auch in der Projektlaufzeit erst voll entwickelt, haben wir doch schon sehr viele Kollegen, die auf der Codebasis aufbauen. Und das wird in diesem (institute) auch vor allen Dingen getan. Also die Weiterfinanzierung dann über dieses (institute) und überbrückt haben wir das mit Institutsmitteln. Das muss man glaub ich auch machen, also da muss man zu bereit sein. Sonst wird es wahrscheinlich schwierig. Die Programmierer muss man ja auch auf Dauer halten. Da kann man nicht ständig wechseln, das wär dann, glaub ich, einfach nicht gut.	
Interview 21	21:32		Ist das dann auch wieder projektbasiert oder dann eine länderfristige Finanzierung?

Interview 21		Ja, das ist eine spannende Frage. Die (institute) sollen eigentlich übergehen in die nationale Forschungsdaten Infrastruktur, das haben Sie bestimmt auch schon gehört oder?	
Interview 21			NFDI?
Interview 21		Ja, genau. Genau, so war es geplant auch. Die (institute) sind als Vorbereitungsprojekt eigentlich gedacht, für die NFDI, sodass Gruppen, die schon Entwicklungen im Haus haben, sich weiter gut aufstellen können, um an der NFDI beteiligt sein zu können. Wir sind ja mit der NFDI (unv.) mit unseren Systemen vertreten, sowohl das ELN, als auch das Repositorium bilden eigentlich den Hauptechnologiepart in der NFDI (unv.) und - genau, also das ist der Plan. Der Übergang von (institute) dann in NFDI.	
Interview 21	22:30	Das wäre dann natürlich, also das (institute) ist jetzt terminiert auf vier Jahre und NFDI dann wieder auf fünf, wobei die NFDI ja dann die Option bieten würde auf 10 Jahre ausgewählt zu werden, um dann in diese längerfristife Förderung überzugehen und das ist natürlich die Hoffnung dahinter. Wenn das nicht funktionieren würde, müssten wir uns ein Nutzermodell überlegen, also im Prinzip ein Kostenmodell, das auf die Nutzer übertragen wird auf Dauer, wenn man keine Förderung mehr hat. Also nach dieser Laufzeit vom (institute) und NFDI.	
Interview 21	23:04		Kommen wir mal zu einem anderen Thema. Nun ist es ja jetzt schon sehr weit in der Zukunft gesprochen, wie ist denn gerade im Jetzt? Welche konkreten Ziele sollen mit dem / also welche konkreten Ziele setzen Sie sich mit dem Repositorium?
Interview 21		Wir haben ein sehr konkretes Ziel. Und zwar, ist es / haben wir zwei Bereiche aktuell, also das Repositorium ist dazu da um Forschungsdaten zu speichern, das hatte ich ja gesagt. Das ist ja auch irgendwie so ein allgemeines Ziel aller Repositorien und wir speichern Originaldaten von Reaktionen. Dazu gehören hauptsächlich natürlich die Beschreibungen und Schemata der Reaktionen, die Produkte, Kennzeichnungen, Produkte, Nebenprodukte, Lösungsmittel und so etwas, aber es gehört auch die Analytic der Produkte am Ende dazu.	
Interview 21		Und die Reaktionsbeschreibung plus Analytic, das was man eigentlich so als Neuheitswert benötigt, wenn man / oder belegen muss, wenn man publiziert. Und das war so der Ausgangspunkt. Sonst sind wir eigentlich so ein bisschen weiter, dass wir uns sagen, okay, wir wollen nicht nur diese Core Repositorium Funktion implementiert sehen, sondern auch diese Datenbank parallel dazu aufbauen, weil wenn wir schon Reaktionsdaten speichern, dann können wir das natürlich auch / also und die nötigen (unv.) implementiert haben, dann können wir das natürlich auch für einfache Reaktionen, wo man vielleicht nicht darauf aus ist, gleich die Analytic mit zu speichern.	

Interview 21		Man hat sie ja aus verschiedenen Gründen vielleicht gar nicht - je nachdem, welche Datenquelle man verwendet - und aber die Verfügbarmachung dieser Daten als Reaktionsdaten an sich ohne Analytisches halt, hat auch einen gewissen Wert und ich glaube das ist eigentlich für Wissenschaftler so ein in Datenbanken (unv.) suchen. Das haben wir jetzt angefangen, das ist auch jetzt schon seit ein paar Tagen verfügbar, das (Scheme only?) auf dem Repositorium und jetzt ist natürlich so das mittelfristige Ziel das Scheme Only mit Datensätzen so zu bestücken, dass auch tatsächlich die Suche da realistisch erfolgreich ist.	
Interview 21	24:32:00	Das schaffen wir nicht mit (year/number), Datensätzen, sondern da müssen wir mit relativ viel Daten losgehen und das ist aktuell eigentlich noch (unv.) Patentdatensätze, die oft verfügbar sind. Und das würden wir gerne machen. Also, das konkrete nächste Ziel ist die offen verfügbaren Patentdaten, die man zur Verfügung hat, an der auch andere arbeiten, die auf dem Gebiet gearbeitet haben und die dann extrahiert haben, die dann im Repositorium zur Verfügung zu stellen. Dann hätten wir eine ganz gute (Anbindung?).	
Interview 21	25:41:00	Dann könnten auch (unv.) und Forscher Reaktionen und Produkte suchen und haben auch eine wirklich gute Chance, auch was zu finden. Also was an Datenbanken, die wirklich auch aktuell bekannt sind, herankommt.	
Interview 21			Und wie kontrollieren Sie dann jetzt die gesteckten Ziele?
Interview 21		Ja, das ist relativ einfach. Also (lachen) bei so einem doch relativ (unv.) Vorhaben, wie wir das haben, ist es ja / also gibt es dann ja immer das / sind die Ziele ja immer so zweigeteilt. Das erste ist ein ein verlässlicher Code mit einem ordentlichen Bugfixing hintendran und das prüfen wir Schritt für Schritt. Da haben wir natürlich zunächst den Code, den wir auf unseren (Tech Servern?) laufen haben, dann spielen wir sie auf die interne Version, da wird das geprüft und dann kommt der Code dann auf den allgemein zugänglichen Code im Repositorium, sodass die Anwendung sichtbar ist und dann sind wir natürlich mit wenigen Teilschritten da, dass wir einen verlässlichen Code stehen haben und ihn verwenden können, das heißt, die Daten einstellen können und mit unserem Workflow dann auch überprüfen können und wenn das Ziel erreicht ist, dann ist das nächste immer dann der Datenumfang.	
Interview 21		Also, dass man dahinkommt, dass man nicht nur zwei, drei Reaktionen die Woche hat, sondern in diesem Fall mit guten (unv.) an Reaktionen, die dazu kommen und das sind für uns dann / ich glaube, es wird einfach ein monatliches / eine monatliche Anzahl sein an Reaktionen, die wir zur Verfügung stellen möchten, von diesem Reaktionsdatensatz und ja, das ist einfach zu prüfen, da müssen wir sehen, ob wir unsere Zahlen erreicht haben.	
Interview 21	27:21:00	Also wenn wir dann (unv.) Reaktionen pro Woche oder pro Monat planen, das weiß ich noch nicht, wie wir den (unv.) zählen wollen, das ist einfach nur die Zahl praktisch abhacken oder sehen, dass wir noch ein bisschen im Rückstand sind und das nacharbeiten.	

Interview 21			Welche Schwierigkeiten und Herausforderungen gibt es denn beim Management von (platform)?
Interview 21		Die Wissenschaftler sind die Herausforderung. Das ist ganz klar. Also eigentlich ist es / das ist ein ganz schwieriges Feld, da wir keine systematische Unterstützung bisher haben von den Fördergeldgebern, es gibt keinen - negativ ausgedrückt - Zwang, Forschungsdaten zu speichern und zugänglich zu machen, das wird alles sehr sehr weich ausgedrückt, immer noch. Es wird nicht konsequent kommuniziert, dass das tatsächlich ein Hauptrequirement ist, wenn man publiziert oder wenn man Arbeiten publiziert, die öffentlich gefördert waren.	
Interview 21		Und damit ist es den Wissenschaftlern aktuell nicht wirklich klar, die nehmen das nicht ernst und demnach werden natürlich solche Systeme wie unseres nicht systematisch genutzt und auch andere nicht - ja, also das ist / es ist ja wohin man blickt eigentlich dasselbe, es sei denn man hat eine Community, die tatsächlich da schon sehr viele Jahre reingewachsen ist und das als normales Prozedere akzeptiert hat, also wie in der Biochemie zum Beispiel, in der Biologie gibt es einige Beispiele, zur (unv.) gibt es die (name), da läuft es super gut, aber für alle anderen Bereiche ist es ja echt noch sehr sehr rudimentär.	
Interview 21		Und die Wissenschaftler sind das Problem, weil die natürlich / was heißt das Problem, aber jeder Mehraufwand, den man leisten muss - nicht gewollt - und wird vehement abgelehnt und das sind die Herausforderungen vor denen wir stehen.	
Interview 21		Wie etablieren wir Syteme, die natürlich einen gewissen Mehraufwand bedeuten, weil immer, wenn man Forschungsdaten aufbereiten muss oder die Qualität halten muss, dass die andere ansehen können, ist das ein Mehraufwand - ganz klar. Und der wird vom einzelnen vermieden wo immer das geht.	
Interview 21	29:06:00	Natürlich ist es schon noch so, dass man sagt okay, es gibt sehr sehr viele Vorteile von solchen Systemen und gerade die Nachnutzer sehen die Vorteile natürlich auch. Nur der einzelne Wissenschaftler, der jetzt seinen Beitrag leisten muss und seine Daten aufbereiten muss, vielleicht auch gar nicht mehr weiß, wo er jetzt jeden Datensatz gespeichert hat, für den ist das natürlich eine Last. (-) Ja, aber daran kann man auch nicht viel ändern.	
Interview 21			(Lachen) Haben Sie denn noch weitere Schwierigkeiten oder Herausforderungen beim Management?

Interview 21		Eigentlich würde ich sagen nein, also wenn wir diesen Punkt / die fehlende Akzeptanz der Wissenschaftler oder das fehlende Verständnis dafür, dass natürlich Forschungsdatenmanagement auch Aufwand ist, dagegen ist alles andere wirklich nicht bedeutend. Natürlich haben wir Probleme, dass wir Bugs haben in unserem Code und das muss gelöst werden und natürlich sind wir immer zu wenig Leute für die Herausforderungen, die wir da haben, aber das sind alles Sachen, die bekommt man in den Griff, das sind keine Dinge, die uns langfristig beschäftigen. Das kriegt man gelöst und wenn man da den Zeitplan nicht einhält, dann ist man später, das ist so, das ist immer ärgerlich und das mag man nicht, aber das sind Sachen, die löst man.	
Interview 21	30:39:00	Nur die Begeisterung der Wissenschaftler, beziehungsweise die Akzeptanz der Wissenschaftler, das ist eine ganz andere Geschichte. Das können wir auch nicht alleine lösen.	
Interview 21			Damit habe ich jetzt auch den ersten Block über die Organisation hinter (platform) schon beendet. Jetzt möchte ich zu meinem zweiten Block über gehen. Da geht es dann vor allem so um die Plattformnutzung. Meine erste Frage dazu ist, welche Funktionalitäten und Möglichkeiten bietet (platform) den Nutzern?
Interview 21		(platform) hat ja, wie ich schon sagte, eigentlich zwei Arten der Nutzung. Also die Aktiven, die die Daten beitragen zum speichern, also diejenigen, die publizieren und müssen dann die Daten offenlegen oder begleitend andere zur Verfügung stellen, vielleicht auch schon im Revue-Verfahren. Und dann die passiven Nutzer, die den Datensatz ansehen wollen, vergleichen wollen, die eine (Synthese?) gemacht haben die ähnlich ist oder gleich ist und die dann ansehen wollen, ob sie tatsächlich das Mollekül erhalten haben und dann die analytischen Daten zum Beispiel vergleichen.	
Interview 21		Der Vorteil für Letztere ist klar, also die Datenbanken gibt es eigentlich nicht. Also das, was man bisher hat ist zum Beispiel (name). Die (name) Datenbank / also die (synthetic pages?) von (name), die haben auch einen Datensatz oder häufig Datensätze, die auch analytische Datenfiles beinhalten, aber in der Regel immer Bilder. Und das reicht ja eigentlich nicht. Also wenn ich ein Mollekül ansehe und es ist ein bisschen komplexer und ich will wissen wie die Multiplizität innerhalb des Spektrums aussieht, dann bringt mich in der Regel das Bild nicht weiter, weil genau das, was ich ansehen will, kann ich dann aufgrund der Auflösung nicht mehr sehen.	
Interview 21		Genau so kann ich eben keine Berechnungen durchführen, ich kann ja keine Integrale bestimmen oder Multiplizitäten, die anders angegeben sind nicht prüfen und genau das bietet einfach ein Repositorium. Wenn man die Datenfiles vorliegen hat, kann man genau das prüfen, kann ich Daten übereinanderlegen, kann, wenn Fehler passiert sind, das neu analysieren und genau das hat man.	

Interview 21	32:42:00	Das hat man bisher in Datenbanken, wie (name) nicht, die können ja nur auf die Originalliteratur verweisen. Die Originalliteratur hat dann wieder keine original Datenfiles, sondern auch wieder nur Bilder maximalst und dieses Problem wird damit gelöst. Was man auch hat / (platform) ist ja kein Repositorium im generischen Sinne, wir arbeiten ja die Daten im Hintergrund auf. Das bedeutet auf der einen Seite, dass es ein bisschen länger für das Processing braucht, aber auf der anderen Seite bedeutet es auch, dass jede Struktur, die dann zu finden ist, man wieder exportieren kann und die Identifier bereitgestellt werden. Das ist zum Beispiel (Inchety?) oder (InChe Smiles?) (unv.) und man könnte im Prinzip die ganze Datenbank und die Daten dann wieder nutzen auch im chemischen Sinne, ohne, dass man neu zeichnen muss oder irgendwelche anderen Formate generieren muss.	
Interview 21		Und das gilt eigentlich für den gesamten Inhalt. Also man kann die Reaktionen komplett exportieren, man könnte sie zum Beispiel auch in das eigene Laborjournal wieder importieren, also man hat eine sehr hohe Nachnutzbarkeit der gesamten Daten. Für diejenigen, die das Repositorium zum Daten einstellen nutzen, hat man, wie die Nutzer das empfinden große Nachteile, weil man sehr gut darauf achten muss, dass man tatsächlich vollständige Daten abgibt, das wird halt durch das Revueing gesichert und da achten wir auch drauf. Das ist natürlich / wird als unangenehm empfunden, weil wenn ich etwas nicht habe und werde darauf hingewiesen, das ist es natürlich ein bisschen / das ist immer unangenehm und das mag keiner.	
Interview 21		Auf der anderen Seite müssen alle Metadaten eingegeben werden und da achten wir auch drauf. Teilweise ist / ohne den entsprechenden Metadatensatz wird auch die Weiterleitung zur Offenlegung gar nicht erst freigeschaltet. Das empfindet der Einzelne dann meistens auch als etwas, was er nicht mag, was einfach zusätzlicher Aufwand bedeutet. Aber nun gut, ich meine für Datenbanken musst du nicht drüber diskutieren, dass halt einfach Metadaten da sein müssen und dass sie dann vom Nutzer beigegeben werden müssen ist auch irgendwo klar.	
Interview 21	33:31:00	Auf der anderen Seite ist natürlich so ein Datensatz, wenn er vorhanden ist und derjenige den Datensatz vollständig abgegeben hat und das freigegeben wurde / hat eine gewisse Aussagekraft für dich, weil er zum einen auf Vollständigkeit geprüft wurde, zum anderen aber auch auf eine gewisse Plausibilität, das heißt, das ist nicht einfach ein Datensatz der irgendwo abgelegt wurde und man weiß nicht, was da drin ist und ob es stimmt, sondern so ein gewisses Level an Prüfung ist schon durchlaufen und das gibt dem einzelnen auch Sicherheit.	

Interview 21		Das hatten wir in der Vergangenheit schon häufiger, dass der einzelne vielleicht gewisse Bedenken hat, nicht weiß, ist es jetzt sauber genug, also reicht die Qualität, hab ich das richtig aufgewertet. Wenn die Daten von uns zurückkommen und geprüft sind weiß man das in der Regel. Ne, das ist auf jeden Fall schon mal / hat es eine Hürde genommen. Und auf der anderen Seite hat man vielleicht sich perfekt vorbereitet für entweder andere Datenbanken oder die Journale.	
Interview 21		Das heißt, wenn man sich die Mühe gemacht hat und alles ordentlich aufbereitet hat, dann ist man nachher perfekt vorbereitet für alles, was dann danach noch kommt und hat eine gewisse Sicherheit noch, dass der Datensatz wirklich gut ist. Die Kombination von beidem, ELN und Repositorium, ist eine Sache, die ist super hilfreich. Allerdings man dafür beide Systeme natürlich auch nutzen und das ist so der Punkt.	
Interview 21	34:46:00	Also für unser / für unsere Universität, das KIT, sind die Systeme alle frei installiert, das kann jeder benutzen, da ist die Hürde natürlich gering. Für andere Institute, die beide Systeme, wenn sie wenn die Vorteile auch nutzen wollen, auch installieren wollen, ist es immer eine gewisse Hürde. Deswegen ist es so der Punkt, dass wir aktuell sehen, dass die Nutzung noch nicht perfekt ist, weil man diese Hürde nehmen muss, beide Dinge zu installieren oder zumindest das ELN zu installieren und dann das Repo mit zu bedienen.	
Interview 21	36:26:00	Aber dann ist es natürlich perfekt. Dann kann man parallel dokumentieren zu der normalen Arbeit und hat im Prinzip, durch einen Transfer der Forschungsdaten keine Arbeit mehr mit dem Verfügbarmachen der Forschungsdaten in einem Repositorium.	
Interview 21			Eine Nachfrage habe ich zu der Funktionalität der Plattform. Das heißt, wie ich das jetzt verstanden habe, kann man ja seine Daten hochladen oder auch andere Daten runterladen. Kann man auf der Plattform direkt auch Berechnungen durchführen oder sich das angucken lassen oder muss man das dann immer downloaden?
Interview 21		Ja ja, genau. Also das kann man machen, es gibt verschiedenste Funktionen für Chemiker, also das könnten wir / ohne könnten wir das auch gar nicht machen, weil ja sonst die ELN Nutzer komplett unzufrieden wären. Also so etwas wie eine Reaktionstabelle wird automatisch berechnet, genau so werden die Mollekularmassen berechnet, wir haben auch andere Datenbanken, entweder über eine Schnittstelle angebunden oder die Daten auch schon integriert, dass zum Beispiel parallel zu der Erstellung von Mollekülen in sowohl ELN als auch Repositorium, werden die Daten in (organisation) abgefragt und wenn die Daten, die in (organisation) sind für Chemiker relevant sind auch gespiegelt, sodass man sie direkt zur Verfügung hat und der Link für (organisation) wird erstellt.	

Interview 21		Genau so könnte man gleichzeitig in SciFinder die Daten abfragen, wenn man nach einer Reaktion sucht, müsste man die in SciFinder nicht noch einmal neu zeichnen und suchen, sondern könnte das gleich über das ELN oder Repositorium machen. Dann haben wir einen eigenen Sprektrenviewer eingebaut. Das ist eine Eigenentwicklung, die natürlich auf anderen Open Source Produkten aufbaut, aber trotzdem für uns / also für das Repositorium und das ELN neu aufgesetzt und integriert wurde. Das ermöglicht dann die Auswertung von zum Beispiel Massenspektren, NMR Spektren direkt in dem Tool. Das heißt ich brauche für verschiedenste Anwendungen überhaupt keine externe Software mehr, sondern kann das direkt im System machen.	
Interview 21		Und ja, was wir auch noch anbieten ist so etwas wie eine Reportingfunktion, das mögen auch viele, dass man im Prinzip eine Zusammenfassung der Daten sich generieren lassen kann direkt im System, das wird aber eigentlich eher für das ELN als für das Repositorium verwendet. Das ist dann so etwas wie Projektberichte oder supporting information, dass man das direkt aus den vorhandenen Daten generieren kann. Das ist natürlich recht geschickt, wenn man / also ich benutze das, aber ich glaube auch viele von den Kollegen nicht, weil sie da auch noch zu unsicher im Umgang sind.	
Interview 21	37:45:00	Man kann zum Beispiel, wenn man die Forschungsdaten eingestellt hat ins Repositorium, sich die entsprechenden Daten auswählen und aus den Metadaten und den Daten selbst dann die Supporting Information generieren mit den Links zu den DOI`s und dann hat man gleich im Prinzip genau das fertig, was man nachher einreichen muss. Und das ist natürlich maximal komfortabel. Ist nur die Frage / ja, also da muss man sich auch ein bisschen einarbeiten, es ist nicht alles selbsterklärend, da muss man schon drüber nachdenken, welche Daten möchte ich da in der Supporting Information haben, wie strukturiere ich das denn und muss sich dann gut Gedanken machen, wie man das ganze auch aufbaut.	
Interview 21	39:48:00	Die Systeme sind dann halt schon komplex, das kann man ohne Einweisungen / kann man das glaube ich nicht zielführend machen.	
Interview 21			Gerade sagten Sie ja auch, dass Sie die Nachnutzbarkeit der Daten als hoch einschätzen. Da wollte ich nochmal nachfragen, warum genau?
Interview 21		(--) Das ist eine gute Frage. Wenn man sich so die normalen Nachnutzungsszenarien ansieht, wie man Forschungsdaten nachnutzt - jetzt als Chemiker - dann gibt es ja den Chemiker, der sucht eine Synthese und möchte die wieder durchführen und wie macht er normalerweise? Da sucht er einen SciFinder, in der Regel, oder (unv.), findet eine Reaktion, dann findet er die Versuchsdetails dazu nicht, das heißt, da geht er auf einen Link und sucht sich dann die Originalpublikation, dann muss er gucken, dass er Zugang hat und dann sucht er sich die Supporting Information aus dieser Publikation und wenn er Glück hat, findet er dann das, was er sucht.	

Interview 21		Weiß er aber von Anfang an nicht, weil häufig ist es ja einfach auch nicht in der Supporting Info, häufig ist es auch so, dass es auf eine andere Publikation verlinkt wird oder es wird eine Arbeit referenziert und man muss es dann nochmal suchen. Also es ist in der Regel schon recht aufwendig und wenn man viele Sachen muss, kostet es auch enorm viel Zeit. Und dann man natürlich noch keinen Zugang zu den Originalspektren, die man dann vergleichen würde.	
Interview 21		Das heißt, wenn man ein Produkt raus hat, muss man es auch nochmal extra analysieren, ob es auch das Produkt ist, anhand der Peek-Listen, die gegeben sind und für uns ist es / oder im Repo ist es so, man findet die Reaktionen, man wählt die Reaktion aus und lässt sie sich exportieren, zum Beispiel als Word Format und hat die Reaktionsvorschrift direkt vorliegen.	
Interview 21		Dann sieht man sich die Orginialfiles an und kann direkt vergleichen, ob das Produkt übereinstimmt oder nicht. Das heißt, ich brauche keine externen Systeme, ich wähle mir einfach das, was ich brauche aus, exportiere es im Zweifelsfall, wenn ich das genau so nachmachen will und habe direkt alles vorliegen, was ich brauche.	
Interview 21	41:35:00	Und wenn ich dann noch eine ähnliche Reaktion machen will, exportiere ich die Daten, die ich haben möchte und dann kann ich auch direkt die Files schon wieder verwenden. Das heißt, ich könnte zum Beispiel das Wordexportformat verwenden und könnte das Datenschema in (ChemDo?) wieder öffnen und könnte dann die Molleküle verändern, die Daten speichern und dann kann ich mir das gleich wieder berechnet ausgeben lassen. Das heißt, ich habe eigentlich keinen Aufwand in dieser Wiederverwertung von wem, was da mal eingegeben wurde. Ich kann es direkt nachnutzen.	
Interview 21			Wie beschreiben Sie denn die Nutzergruppe von (platform)?
Interview 21		Das sind / wie meinen Sie Nutzergruppe? Von dem Level der Ausbildung oder von der Fachrichtung?	
Interview 21			Fachrichtung, genau.
Interview 21		Das sind Chemiker. Das sind meistens organische Chemiker. Auch so ein bisschen / Materialwissenschaftler auch, die da so ein bisschen interessiert sind, wie sie Materialien beschaffen können, die organische Chemiker herstellen, aber in der Regel Organiker. Wenige Anorganiker haben wir auch, die sind aber meistens sehr auf die Kristallstruktur Datenbanken ausgerichtet und brauchen daher (platform) weniger.	
Interview 21			Welche Schwierigkeiten können denn auftreten beim Austauschen der Daten über (platform)?
Interview 21	43:16:00	Sie meinen austauschen ...?	
Interview 21			Naja, zum Beispiel beim Upload oder beim Download der Daten, Verständnisprobleme, was so an Probleme auftauchen.

Interview 21		Gute Frage. Beim Upload haben wir / oder beziehungsweise beim Auffüllen der Daten, also beim Upload gibt es eigentlich keine Probleme, das sind Prozesse, die sind ja also eigentlich Routine, der Datenfile Upload stellt kein Problem dar. Was manchmal ein Problem ist, sind die Metadaten abfragen, weil die muss man ja auch sehr gut bestimmen, welchen Metadatensatz man zur Auswahl stellt, damit nachher auch eine gewisse Standardisierung möglich ist und das ist von unserer Seite immer so ein bisschen eine Herausforderung. Zum Beispiel, welche (Ontologiebäume?) erlaubt man und decken die alles ab?	
Interview 21		Also das ist von unserer Seite ein Problem, was bietet man an, damit die Wissenschaftler auch die Auswahlmöglichkeiten haben, die sie brauchen und das ist bei den Ontoglogien einfach noch nicht umfangreich da. Da muss man sich mit rudimentären Diensten zurecht finden. Große Datenmengen spielen für uns eine / also sind ein Problem, wenn jemand sehr große Datenmengen auf einmal exportieren möchte.	
Interview 21		Zum Beispiel, wenn die Bildformate gleich mit exportiert werden sollen und nicht Identifier oder Strings, da haben wir schon gewisse Probleme mit den Servern, die uns das alles verarbeiten, die Bildformate erstellen und das braucht dann eine Weile. Also im Prinzip ist es nicht ein so großes Problem aber es braucht halt einfach eine Weile und da muss der Nutzer Geduld mitbringen.	
Interview 21			Wenn ich jetzt nun einen Datensatz hochladen möchte, welche Anforderungen gibt es, außer, dass ich die ganzen Metadaten ausfüllen müsste?
Interview 21	45:09:00	Also sie müssen Struktur zeichnen können, also einen Struktureditor, dann / gut die Metadaten nehmen wir an, hätten wir, also es gibt empfohlene File Formate, die wir gerne im Repo sehen würden, weil sie offene File Formate sind für die entsprechenden Messmethoden. Auch nur die können prozessiert werden, sodass man automatische Bilderzeugung hat. Also das Repositorium erlaubt beim Hochladen der richtigen Dateiformate eine paralle Erstellung von einem PNG Format und das kann dann verwendet werden, um zum Beispiel eine schnelle Preview zu ermöglichen.	
Interview 21		Das funktioniert aber natürlich nur, wenn wir nicht die propretären Dateiformate haben, weil die können wir dann nicht prozessieren, können wir zwar speichern und vorhalten, aber nicht prozessieren. Und das ist so ein Handycap, das heißt, wenn man eine Preview haben will, dann muss ein offenes Dateiformat einfügen. Die geben wir in den How To Pages an, das ist ein JCAMP in der Regel, Formate, wenn man das nicht hat, hat man natürlich auch kein Preview.	
Interview 21	46:19:00		Und als Nutzer der Plattform, welchen Regeln unterliege ich da?

Interview 21		Im Prinzip sind das die Regeln, auf die sich die meisten Plattformen berufen. Also man muss gewisse Zusicherungen unter den Nutzungsbedingungen bestätigen, das es die eigenen Daten sind, dass man die Daten nicht mit Schadsoftware erstellt hat oder Schadsoftware in das System bringt, dass die Daten die Datenschutzrichtlinien erfüllen, solche allgemeinen Dinge, also das muss man bestätigen, das muss man auch bei der Anmeldung soweit ich weiß ein mal bestätigen und dann werden Teile nochmal währen der Submissions abgefragt.	
Interview 21		Man muss als Nutzer auch bestätigen, dass man zum Beispiel die Daten mit dem Wissen anderer einstellt, wenn das Daten anderer sind. Das ist natürlich für analytische Files zum Beispiel immmer ein Punkt der wichtig ist. Was muss ich noch? / registriert sein muss man zum Daten hochladen, also das ist so eine prinzipielle Voraussetzung. Zum Ansehen der Daten muss man nicht registriert sein, das kann man so machen, zum runterladen der Dateien aber muss man auch registriert sein. Genau, sonst fällt mir spontan nichts ein, aber da könnten Sie theoretisch auch die Nutzungsbedingungen ansehen, da steht das auch alles drin.	
Interview 21	47:50:00		Vorhin haben Sie ja schon erzählt von dem Revue Prozess und wie Sie die Qualität der Daten sicherstellen wollen. Wie stellen Sie denn die Qualität der Daten sicher?
Interview 21		Also eine Garantie natürlich gibt es nie. Also ich kann nicht zum Beispiel garantieren, dass eine Struktur / oder dass der Datensatz perfekt zur Struktur passt und das einwandfrei belegt wird, da bin ich also zwar Wissenschaftler und kann das auch für die meisten Strukturen ganz gut abschätzen, aber eine Garantie gibt es nicht, das heißt der einzelne Wissenschaftler kann das jetzt halt auch nicht 100 Prozent belegen. Aber, wir sichern zum Beispiel / also es gibt ein mehrstufiges Verfahren. Ich versuche es jetzt kurz darzustellen, weil es relativ umfassend ist.	
Interview 21		Wir checken zum Beispiel / also Sie müssen, wenn Sie ein Dateiformat hochladen - nehmen wir mal an es ist ein offenes, weil es ja auch eigentlich gefordert wird oder zumindest gewünscht wird, dann wird das ja ausgewertet und die Analyse dazu gespeichert, also sowas wie (unv.), NMR und dann kommen die einzelnen Signale, die gelistet werden mit der Multiplizität - Duplet, Triplet, was auch immer - und die Sachen werden gezählt.	
Interview 21		Das ist so unser erstes Level. Das heißt, jeder, der seine Signale angibt / die werden gezählt und werden abgeglichen mit der eingegebenen Struktur. Wenn das nicht übereinstimmt, muss derjenige nachprüfen und dann muss derjenige auch angeben, warum das so ist. Also wenn zum Beispiel ein Proton fehlt, muss er angeben, ob das ein Austausch sein kann mit dem (unv.) Lösungsmittel oder was sonst der Grund sein kann.	

Interview 21		Genau, so gibt es eine (unv.)-Prüfung. Es gibt ja die Prüfung bei der Masse, die gegebene Masse, angegebene Masse, in der Analyse wird geprüft, was die Struktur der theoretischen Berechnung hergibt. Das einzige, wo wir es nicht prüfen ist das IR, weil es häufig zu schwierig ist und häufig auch einfach wissenschaftlich betrachtet nicht gemacht werden kann. Das ist dann einfach ein Indiz und so werten wir das auch.	
Interview 21	49:36:00	Es gibt dann eine zweite Prüfung, ein anderes Level, auf Prüfung der offenen Dateiformate und Prozessierbarkeit. Hier wird geprüft, ob jeder Datensatz ein offenes Dateiformat enthält und ob der verarbeitet werden kann von unserer Software. Das ist nicht unbedingt ein Kriterium, sodass der Datensatz auch insgesamt akzeptiert wird, aber es gibt von uns einen Hinweis, dass da genauer hingesehen werden muss, wenn es nicht prozessierbar ist. Und dann gibt es noch ein drittes Level, das benutzen wir aber aktuell nicht systematisch, das kommt in Zukunft, ist auch etabliert, aber noch in der Testphase, da prüfen wir die Datenfiles, die prozessierbar sind und prüfen den automatisch gefundenen Inhalt mit dem was anhand der Struktur vorhergesagt wird.	
Interview 21	50:42:00	Und das gilt für NMR genau so wie für Masse und dann in diesem Falle auch für IR. Das bedeutet, bei IR haben wir zum Beispiel Maschinen Learning Tool im Hintergrund laufen, das mit den Daten, die wir bisher im Bereich IR bereits gesammelt haben gefüttert wurde, also das Netz trainiert wurde und hier prüfen wir, ob, zum Beispiel bei Anweseneheit von einer (unv.) in der Struktur die (unv.) auch im Spektrum gefunden wird. Dann prüfen wir bei (unv.) Spektren, in Kooperation mit der (unv.) in Köln, ob die vorhergesagte / das vorhergesagte Spektrum für (unv.) jeweils mit dem gefundenen Spektrum und den gefundenen Signalen im Spektrum übereinstimmt.	
Interview 21		Und bei der Masse ähnlich, also hier prüfen wir die Einzelscans von den einzelnen Messungen und prüfen, ob das Signal, beziehungsweise die Masse, die laut Mollekül vorhanden sein sollte, dann tatsächlich auch im Datenfile gefunden wird und nicht nur in der manuellen Auswertung, die ja der Wissenschaftler auch beeinflusst hat.	
Interview 21	51:27:00	Es gibt dann aufsummiert ein Ranking von minus, ich glaube 4 Punkte - gar nicht vorhanden, passt überhaupt nicht, Kalkulation nicht übereinstimmend mit Auswertung und Datenfiles nicht lesbar - bis 10 Punkte, Maximalpunktzahl - alles auswertbar, alles prozessierbar und passt mit Vorhersagen überein für die überhaupt analyisiert wurden. Ja, und das wird dann als Hilfestellung für das (unv.) Revue genutzt, sodass man da schneller vorankommt und da nicht alles, das was ich jetzt aufgezählt habe, einzeln prüfen muss.	
Interview 21			Verwandt mit der Datenqualität ist ja auch das Vertrauen. Wir wird denn das Vertrauen in die Daten sichergestellt?

Interview 21	52:33:00	Was sehen Sie denn als Vertrauen an? Also, dass es stimmig ist und der Datensatz im Nachhinein genau das beschreibt, was man möchte? Oder Vertrauen in dem Sinne, dass die Daten auf Dauer gesichert sind und zugänglich sind?	
Interview 21			Also Vertrauen, das der Nutzer (-) ja / der Plattform dann entgegenbringt.
Interview 21		Im Prinzip ist es so, der Nutzer, der die Daten auf der Plattform speichert, sieht ja glaube ich eher so den Vertrauenspunkt darin, dass Daten, die zum Beispiel einem Embarro unterliegen nicht offen gesehen werden können oder niemand die Daten stiehlt. Ich glaube, das ist so / das ist so der Hauptaspekt bei Vertrauen in die Datenbank. Ich habe jetzt noch nicht erlebt, dass jemand gesagt hat, mein Vertrauen in die Datenbank schwankt, wenn die Datenqualität nicht hoch ist, das ist so / weil das sieht man ja dem Datensatz schon an und die Sachen sind ja auch in der Regel publiziert, also es ist / mit dem Verfahren, was ich jetzt geschildert habe besteht eigentlich nicht der Gedanke, dass irgendwie schlechte Daten oder irgendetwas, das nicht geprüft wurde, wo man alles ganz kritisch ansehen muss.	
Interview 21		Das würde ich sagen, ist für uns einfach raus. Es ist eher so der Aspekt, wenn ich jetzt auf einer andere Datenbank oder auf einer andere Plattform Daten hochlade und das Repositorium ist noch nicht mal in meiner Universität, wie kann ich dann sicher sein, dass nicht jemand anderes die Daten verwendet und das die wirklich geheim sind und dass ich das offenlegen möchte und solche Dinge.	
Interview 21	54:09:00	Und da kann man einfach / also das, ja, kann ich nur aktuell beantworten damit, dass die Systeme aktuell natürlich von uns vom Rechenzentrum geschützt werden und wir so eine gute Sicherheit bieten für unsere eigene Daten, das man natürlich vor Hackerangriffen oder irgendwelchen Viren, die da kursieren, dass man / ja, da kann man gar nicht sagen, dass man da 100 prozentig geschützt ist, also tue ich das auch nicht.	
Interview 21			Und aufbauen auf die Frage, wie stellen Sie denn sicher, dass die Nutzer der Plattform wissenschaftlich korrekt mit der Datenverwertung umgehen?
Interview 21		Ach Sie meinen, dass mir jemand nicht, wenn er registriert ist, den Datensatz runterlädt und für sich verwendet?	
Interview 21			Möglich.
Interview 21		Das kann ich auch nicht sicherstellen. Das ist / im Prinzip kann das jeder tun und es ist immer die Frage, ob das gefunden wird oder nicht. Ich gehe da positiv ran, ich denke, das wird auf jeden Fall gefunden und dann ist natürlich so ein System wie unseres - witzigerweise haben wie die Diskussion relativ häufig - eigentlich das beste, was man machen kann, wie ich finde. Weil es ist / also wir haben mehrere Time Stamps, die wir zur Verfügung stellen können.	

Interview 21		Wir wissen, wann die Substanz eingegangen ist, wenn sie submitted wurde, wir haben das ganze supporting über den Revueprozess, wir können belegen, wer es eingestellt hat, zu welchem Zeitpunkt, wir können eigentlich auch tracken, das machen wir aktuell noch nicht systematisch, da müssen wir auch mal nachdenken, wie häufig welcher Datensatz runtergeladen wird, das könnte man theoretisch auch noch nachweisen, aber im Zweifelsfall haben wir immer auch parallel dazu das DOI Menting über Dataside.	
Interview 21		Das heißt, für so viele Time Stamps, die getrackt werden und dann mit dem Nutzer, der das Original eingestellt hat in Verbindung gebracht werden, dass man einwandfrei belegen kann, wer den Datensatz zuerst erzeugt hat. Und, wenn nachher wer auch immer kommt und diesen Datensatz neu verwendet und publizieren möchte, sobald der Verdacht nur aufkommt, dass du den verwendet hättest, ist es einwandfrei nachweisbar.	
Interview 21	56:00:00	Und das ist, wie ich finde, der beste Schutz, diese Einstellungen und Datenbanken in Repositorien zu relativ frühem Zeitpunkt, wenn der Datensatz von guter Qualität ist, schützt dann eigentlich davor, dass jemand die Daten zu einem Zeitpunkt, wo sie noch nicht öffentlich sind oder jemand anderes es noch nicht gesehen hat, stiehlt und für sich weiterverwendet.	
Interview 21		Und für mich ist es immer / ich würde solche Systeme auf jeden Fall immer sofort oder so schnell wie möglich nutzen, einfach weil sie garantieren, dass das, was man erarbeitet hat, öffentlich sichtbar ist, zu einem sehr sehr frühen Zeitpunkt und das schützt vor Missbrauch. Natürlich kann es verwendet werden durch andere, aber es ist sichtbar, dass es wiederverwendet wurde und es ist sichtbar, wer den Datensatz zuerst erzeugt hat.	
Interview 21	57:15:00		Komme ich nochmal zu einem ganz anderen Thema. Wie bewegen Sie denn Forscher dazu, (platform) zu nutzen?
Interview 21		Kann ich nur bis zu einem ganz / also bis zu einem ganz geringen Teil kann ich das beeinflussen. Ich kann es immer bewerben, aber je mehr ich es bewerbe, desto zurückhaltender sind die Leute. Ich glaube, man kann die Vorteile zeigen, muss es auch, man kann auch daran appelieren, dass natürlich jeder Datensätze braucht und Forschungsdaten benötigt und dass es wir es nur gemeinsam schaffen, wenn jeder bereit ist, sie preiszugeben, dann können wir auch insgesamt einen Status erhalten, wo die Verfügbarkeit von allen Daten sehr hoch ist. Im Endeffekt kann ich es nicht richten, sondern das muss die Gemeinschaft tun.	
Interview 21		Wir brauchen gute Regeln, sowohl von der DFG als auch von den Journalen, die genau das festlegen, was Wissenschaftler mit ihren Daten tun müssen, bis zu welchem Grad sie die freigeben müssen und dann können wir von unserer Seite die Systeme bereitstellen, die genau das bieten, die dafür ausgelegt sind, genau das zu lösen oder den Bedarf dann zu stillen, der dadurch entsteht. Was anderes / also viel weiter können wir das nicht machen, weil die eigene Gruppe können wir schon motivieren und hat auch einen gewissen Einfluss, aber natürlich externe Gruppen nicht.	

Interview 21	58:33:00		Sehen Sie noch weitere Möglichkeiten, Nutzer zu generieren?
Interview 21		Also im Kollegenkreis machen wir das natürlich und bewerben das auch bis zu einem gewissen Grad, also für sehr gute Kollegen, wo wir wissen, dass Sie das auch als Freundschaftsdienst ausprobieren würden und selbst wenn die so ein bisschen reserviert sind und davon noicht so richtig wissen, was sie davon halten sollen, von dem Forschungsdatenmanagement und Dateoffenlegung, ist doch die Zurückhaltung sehr groß.	
Interview 21		Also bei Kollegen machen wir das schon (Babygeräusche) aber ansonsten ist es sehr sehr schwierig. Also die NFDI schafft da glaube ich gute Möglichkeiten in Zukunft da / die Awareness für dieses Thema ist sehr gestiegen, aber das kann nur die Community insgesamt richten. Anders wird das nicht funktionieren. Aus dem Grund den ich auch vorher schon gesagt hatte. Es ist immer so ein bisschen Aufwand mit der Pflege von solchen Systemen verbunden und das machen die meisten einfach nicht freiwillig.	
Interview 21	59:44:00		Eine Besonderheit von (platform) ist ja auch, dass es in die Wissenschaft eingebettet ist. Wie schätzen Sie, beeinflusst die wissenschaftliche Kultur die Nutzung der Plattform?
Interview 21		Sehr. Also das ist überhaupt ein Hauptpunkt, wir als Betreiber natürlich können ganz wenig machen. Wir können anbieten und verbessern, aber wir können natürlich die Datenbasis nicht schaffen. Und das müssen die Wissenschaftler vor Ort machen. Die müssen nutzen, ausprobieren, aber auch testen, also die ersten (unv.) die wurden natürlich mit unseren Wissenschaftlern vor Ort gemacht und wenn wir die nicht hätten / ohne geht es natürlich nicht.	
Interview 21			Welche Entwicklung sehen Sie denn für (platform) in den nächsten 10 Jahren?
Interview 21		Ja, das ist eine gute Frage (lachen). Was wir natürlich gerne hätten, das ist einfach, dass in Zukunft Datensätze vollständig abgelegt werden. Das heißt, dass keine Publikationen mehr - ob das jetzt (platform) ist oder ein anderes Repositorium in der Chemie, dahingestellt / dass keine Publikation mehr erscheint ohne dass die geclaimten Daten oder die geclaimten Ergebnisse dann auch in Repositorien liegen. Und da wir jetzt mit (platform) ein System anbieten, das wir denken, dass sehr weit ist, hätten wir natürlich auch (platform) da vorne mit dabei und dass die Datenbasis wächst und auch für die Nutzer, die dann passive Nutzer sind maximalst attraktiv wird.	
Interview 21		Das in Zukunft / wo wir natürlich in 10 Jahren sein wollen, ist, dass das ganze viel viel einfacher für den Nutzer an sich wird. Dass wir von der Programmierpower nur so viel investieren können, dass nachher diese Datenbereitstellung tatsächlich nur noch minimaler Aufwand ist, auch wenn man die Daten von vorne hochladen muss, nicht über das ELN als Datensammlung geht, die schon vorhanden ist, sondern dass man wirklich sehr sehr einfachen Datenupload unterstützt, da braucht man einfach Zeit für.	

Interview 21	61:09:00	Also da muss man verschiedene Features noch implementieren, da müssen wir die Modelle noch einbinden / da haben wir einen guten Plan dafür, was man alles machen kann, aber da brauchen wir einfach sehr viel Zeit. Und das würden wir gerne machen. Also dass nachher der Nutzer sagt: oh ja, das ist nicht nur Datenablage oder irgendetwas, was ich aufbereiten muss, das total aufwendig ist, sondern sagt: ja, das mache ich so nebenher. Das wäre natürlich ein Traum.	
Interview 21			Und wovon hängt dann der Erfolg ab?
Interview 21		Von den Nutzern. Der Erfolg hängt immer von den / natürlich die Datenqualität, das ist ganz klar, ja, aber das ist nichts, was uns weiterbringt, das ist etwas, das können wir nur gut kontrollieren und gute Mechanismen schaffen, aber eigentlich ist der Erfolg von so einer Plattform / hängt davon ab, ob es genutzt wird. Aktiv und passiv, beide Seiten. Und dann aber natürlich auch / das wäre aber dann nur eine Folge davon, wie ich sehe, dass es Empfehlungen gibt in Journalen zum Beispiel, die sagen: okay, dieses Repositorium können wir empfehlen, das hat sich bewährt, da könnt ihr eure Daten ablegen. Das wäre auch so ein Erfolgsindikator zumindest am Ende.	
Interview 21	62:48:00		Ja, sehr schön, damit habe ich auch schon 26 Fragen um. Gibt es sonst noch etwas, was Ihnen zu Erfolgsfaktoren einfällt oder zu Herausforderungen oder was Ihnen sonst noch so auf der Zunge liegt?
Interview 21		Also, Erfolgsfaktoren gibt es natürlich viele / oder Erfolgsindikatoren, ja. Was immer genannt wird, aber das ist eher so eine Community Sache, dass das Datensätze in den Repositoroien nachher einen gewissen Beitrag zu dem H-Index haben zum Beispiel, ja. Das sind immer so Sachen, die werden / nachher wäre das natürlich eine Sache, die für uns insgesamt für die Community sehr wichtig wäre, aber das schafft man nicht mit einem Repositorium.	
Interview 21		Die Nennung und Akzeptanz durch Journale ist ein großer Punkt, aber auch da ist immer so die Frage, ja, die Verlage bringen höhere Eigeninteressen mit, man kann es auch nicht alleine daran messen, manchmal ist das Eigeninteresse der Verlage so groß, dass man gerade, wenn man auf große Abneigung stößt weiß, dass man erfolgreich war. (Lachen)	
Interview 21		Das ist so eine Sache / dennoch, auch zum Beispiel, wenn Fördergeldgeber nachher sagen, wir nehmen zum Beispiel (platform) in die Liste derjenigen Repositorien auf, die bei uns in den Leitlinien für beispielhaftes Forschungsdatenmanagement genannt werden, das wäre auch ein ganz toller Indikator für uns von Erfolg. Aber das sind natürlich so Wunschvorstellungen, die wir für die Zukunft haben, wo wir erstmal sehen müssen, ob das alles realistisch ist und kommen wird.	
Interview 21	64:25:00	Ich hätte noch eine Frage. Wie ist das denn bei den Großen, zum Beispiel bei (platform). Welche Unterschiede gibt es denn da so in den Antworten, die von der Seite gegeben werden, zu jetzt meinen Antworten. Das würde mich mal interessieren. Also haben sie die gleichen Einschätzungen, was Erfolgsindikatoren angeht oder Erfolg an sich?	

Interview 21			Also tatsächlich ist der gesamte Erfolg von solchen Plattformen eigentlich immer in der Nutzung begründet. Das hat dann natürlich unterschiedliche Auswüchse, sei das jetzt eine bestimmte Anzahl an Daten, die rauf- und runtergeladen werden, an Zitationen, die über die Plattform dann generiert werden...
Interview 21		Ja, Zitationen ist immer so ein Punkt, das finde ich noch nicht einmal so. Also es ist / ja, bis mein Datensatz zitiert ist, dann ist es schon weit gekommen, das kann Jahre dauern und sagt gar nichts über die Plattform aus. Ich habe auch / viele Sachen, die ich als Chemiker nach(unv.) habe, die waren vielleicht schon 10 oder 20 Jahre alt. Hätte damals jemand den Erfolg dieses Datensatzes messen wollen oder eine Plattform, die den Datensatz hostet, hätte er lange warten können (lachen). Also es sind / und das ist ja üblich in der Chemie. Ja, also es dauert manchmal ein bisschen bis eine Synthese wieder für eine andere Anwendung wieder sehr attraktiv ist und man den Datensatz wieder benötigt.	
Interview 21		Es hat gar nichts damit zu tun, ob das toll oder weniger toll ist, es ist einfach, manches braucht Zeit. Und von daher bin ich da so ein bisschen / nenne ich dann auch extra nicht, weil das wirklich so ein bisschen / ich weiß nicht, ob das repräsentativ ist dann. Aber gut.	
Interview 21	66:04:00		Also Erfolg generell ist ja sehr multidimensional. Also ich kann ja auch nicht und das werde ich ja auch nicht im Rahmen meiner Arbeit bewerten, welche Plattform jetzt erfolgreich ist oder nicht, weil prinzipiell ist man ja dann erfolgreich, wenn man es schafft, die Ziele zu erreichen. Und dann gibt es ja ganz unterschiedliche Ziele.
Interview 21		Ja gut, aber man muss schon sagen, dass es ja viele erfolgreiche Plattformen gibt und von denen zum Beispiel können wir auch nur lernen. Allerdings ist es auch so, dass es auch viele Plattformen gibt, die erfolgreich sind, wo wir gar nichts von halten. Wo wir sagen, okay, die sind jetzt zwar erfolgreich, aber so richtig hilfreich sind sie auch nicht. (Lachen).	
Interview 21		Von daher / es ist halt immer so / es richtet sich natürlich auch danach, was man braucht, wenn man als Wissenschaftler / zum Beispiel (organisation) ist so ein Thema. (organisation) ist unglaublich erfolgreich, wir betätigen uns auch bei (organisation), also wir finden es auch nützlich in gewissem Rahmen, aber wenn uns jemand dann sagt, (organisation) ist eine super chemische Datenbank, dann können wir doch alles in (organisation) suchen, dass wir sagen: Hm, ne eigentlich nicht.	

Interview 21		Also das ist eine Datenbank, die uns schon hilft bei der Namensgenerierung und sie deckt gewisse Dinge ab, die man sonst suchen oder generieren müsste, also es sind hilfreiche Tools, aber sie ist nicht gut / sie ist (nicht so super gut geprüft?) und tatsächlich helfen uns die Datensätze nicht so wirklich weiter und jetzt Originaldatensätze findet man auch einfach gar nicht, also weil das einfach nicht Ziel von (organisation) ist, es zu speichern.	
Interview 21	67:30:00	Und so ist es natürlich immer sehr unterschiedlich, was erwartet man von einer Plattform auch und naja, deswegen interessiert mich das auch. Mich interessiert auch immer sehr brennend, weil wir dieses sehr kontrollierte System haben, welche Daten in die Datenbank aufgenommen werden und das einzeln prüfen, das ist natürlich für sehr große Datenmengen gar nicht machbar in dem Maße, wie wir das jetzt machen. Wir bilden so unseren Grunddatensatz, weil wir denken, wenn wir jetzt hier anfangen und sind irgendwie nicht genau, dann haben wir etwas falsch gemacht. Auf der anderen Seite müssen wir naütrlich in Zukunft gute Modelle entwickeln, die sich dann auch skalieren lassen. Aber das ist natürlich auch eine Sache, die rentiert sich dann mit den Nutzerzahlen.	
Interview 21			Ja, also bei den / sag ich mal bei den Großen, wie Sie meinten, ist es ja auch so, dass wirklich schon ziemlich viel Algorithmen gestützt auch abgeschätzt werden kann, ob das jetzt sinnvoll ist oder nicht. Auch da hängt das ganz stark vom Fachbereich drauf an. Wenn ich immer Maschinen habe, die mir immer das gleiche Format ausschmeißen und ich ganz genau weiß, da habe ich den Raum, in dem Daten liegen können mit der und der Struktur oder was auch immer / ist halt viel einfacher zu kontrollieren, als wenn ich eine Wissenschaft betreibe, die eben nicht so plausibel berechenbar ist.
Interview 21		Ja, es ist halt / also ich halte die Chemie eigentlich für eine sehr sehr gut strukturierbare Wissenschaft, dadurch, dass wir Strukturen haben, die uns mehr oder weniger die Richtlinie geben, was da sein muss, wie es aussehen muss, da haben wir schon einen großen Vorteil. Allerdings ist die Abschätzung dann wieder / welches Spektrum oder welcher Spektrendatensatz in Kombination von NMR Spektren machen, machen Spektren, IR Spektren / beweist mir denn, ob es das richtige Mollekül ist, wenn es nicht bekannt ist. Das ist ja dann schon wieder eine andere Nummer.	

Interview 21	68:47:00	Wie realistisch oder wie plausbibel ist, dass der Spektrendatensatz genau das beschreibt, was ich hier erhalten habe oder vorgebe, erhalten zu haben Das ist bei komplexen Strukturen natürlich nicht mehr so einfach, da braucht man auch als Wissenschaftler eine Weile, bis man das ordentlich geprüft hat. Das dann darzustellen in Modellen oder automatisch zu prüfen, ist eine Hausnummer, das vollständig abzudecken, das schaffen wir auch in den nächsten Jahren nicht. Also die Community insgesamt nicht, das ist einfach zu komplex. Aber natürlich kann man unterstützen und das muss man auch, sonst kommt man nicht durch.	
Interview 21			Ja, das ist halt auch genau der Punkt. Weil je größer eine Plattform wird und je mehr kuratiert werden muss oder auch überprüft werden muss, desto weniger skaliert es.
Interview 21		Ja ja, also das ist uns vollkommen klar und ist einer der Punkte, die in Zukunft dann den meisten Raum einnehmen werden. Also weg von dieser Hauptfunktionalitätsentwicklung hin zu mehr algorithmischen Prüfen und Modellen hinterlegen, sodass man auch durch die Kuration und natürlich die Prüfung wirklich mit einem guten Zeitaufwand durchkommt, aber / und das sind auch spannende Aufgaben, ja das ist dann auch die Frage, wer macht das dann am Ende, weil ich werde das dann bestimmt nicht mit meinem Aufgabenbereich, den ich aktuell habe, dann auf Dauer machen, ich meine, ich mache das jetzt in der Anfangsphase und da muss man sehen, wie wir das weiterbringen.	
Interview 21		Aber das sind Entscheidungen, die fallen einfach mit der Weiterentwicklung des Systems, das brauchen wir jetzt nicht für alle Eventualitäten abdecken, sondern das entscheiden wir genau dann, wenn wir wissen wie viele Nutzer wir haben, in welche Richtung geht das, welchen Bedarf haben wir genau und wie gut sind dann unsere Algortihmen zu der Zeit auch, wo wir das entscheiden müssen. Naja, aber das sind alles spannende Fragen.	
Interview 21		Wir sind immer ganz happy, dass wir bisher so weit gekommen sind mit unserem DFG Projekt, weil dafür ist es echt schon recht umfangreich geworden und auch so von dem Nachfolgefunding bis zum Hauptentwicklungstool der NFDI for Chem, da waren wir schon sehr zufrieden. Da denken wir, dass wir auf einem guten Weg sind.	
Interview 21	71:24:00		
Interview 22	Time	Interviewee 22	Interviewer
Interview 22	03:00		And what is the history behind (platform) ?
Interview 22		So the (platform) was concieve in (year). So it is over (years) years old. And the original vision is attributed to cristallographer and I think it is described to polymers as well. Because he had a lot of views of lots of different subjects. (name). He had this vision, if you collect together all the data you can generate new knowledge and maybe generate sufficient knowledge that you dont need to do any exxperiments to generate any new data anymore.	

Interview 22		I think certainly the (platform) is [unv.] the first part of that. If you do collect together the data on a proporiate platform with tools you are able to mine you can generate knowledge that you would not otherwise be able to get or at least either you know get out or you can get out the knowledge more efficiently because you dont have to undertake sort of more expensive experiments to generate it.	
Interview 22	04:04	the original manifastation of the data base was actually printed in decease. That allowed people to look up wether there was a structure that was published for particular chemical compound. But underpinning those books was always a electronic files that was used actually generating those that might envolved into a databse with software that you can search and mine. And you know, yeah, until this sort of like mid (year) sort of the licence, the database was primarly compolled by people abstracting data from the literature. And licence to both industrie and academia.	
Interview 22	04:40	With the introduction of the world wide web and also the development of standard file format people started to deposit their data directly with the (platform). The (platform) did in the meantime sprung up as the organisation has responsibility for maintaining the (platform) (platform) , appologies for the acronym, I hope it make sence when you play it back.	
Interview 22	05:05	And so, now rather than us going us and extracting the data from the literature pretty much the majority of the data is deposited directly by the researches where we sort of our costudiance of the data sets that are often associated with scientif ic outcourse. So that providing, so underpinnings of those scientific articles. But we continue to provide the (platform) where an editorial team have a look at all the structures that been published, make sure they have got a god chemical representations, various other meta data, that allow the structures to be found and published and then structure that into a data base that can be used via software.	
Interview 22	05:50		What is the core offering?
Interview 22		So, there is I am gonna say, there are / the core / well ok. I think a lot of people will access the (platform) through our scientific software which primarily have been developed to run on desktop machines so something to download and to run on there own machines. But we do also have web interfaces where people can come along and search the (platform) and do a sort of basic searches to look up particluar structures. Some of those services are free.	
Interview 22	06:30	The core, I would describe, Is the (platform) base but also the software that surrounds that. Ranging from basic software that allows people to search, analysie and visualise to more saffisticated applicatioins that have a sort of targeted tools for particular domains. So we have a discovery suit wich is used by people in drug discover, a material suit which is used by people wanting to assess the stability of solid forms and maybe optimise those.	

Interview 22	07:00	The other bit that is growing but perhaps doesnt contribute quite so much to the revenue at the moment is sort of any simply forms of contract services that we would do for industrie. But it is more essentially more like partnerships where we would have scientific stuff based at (platform) working on pronlems that industrie providing to us. I guess the offering there is the expertise is supposed to us.	
Interview 22	07:35		My work is about success factors. So how do you define success or a success fuk?
Interview 22	07:40	I wish I had paid more attantion to your brief in beforehand and than might I had some thoughts prepared. But I think it is an interesting question and I was thinking as you were describing your work in the beginning. So an obvious success factors is how many data set are we sharing. But I am not sure how good that success factor is because it is lot of dictate by the rate the wider community is generating this data set. So I think one good success factor is how quickly can we enable ppeople to deposit their data and how quckly can we publish it at that from the point that we know that we areble to publish it. So a typical cut of workflow is the research will generate the data and they might they deposit it straight away.	
Interview 22		But that doesnt mean to say that we can publish it straight away because often it got to be associated with an article and people dont want that data getting out until the article is published. So we have done a lot of work to try and make it as efficient as possible for people to desposit the data with us. but at the same time also run it through various validation checks, provide opportunities to enrich the data by themselves.	
Interview 22	08:35	So that / We put the possible we can get from the researcher. We also have done a lot of work to make sure that our workflows with the scientific publishers are sufficient. So that as soon that we know a data set can be published we can make sure it is out there and also linked to the article.	
Interview 22	09:20	Put a broader success factors for us and probably the most / probably as simple is the number of people who are using that core offering I just described where the core offering is not juist the data base but also the software that surrounds that. How many people in how many organisations are actually taking advantage that software to generate new knowledge. And again think about the efficiency, how easy they are able to do that. Is is it a draw that or commecialy to that and that is something I think interoperablility with other software packages and things can be quite important as well.	
Interview 22	10:00	And I suppose the final success factore that I would highlight and maybe is just the overall sustaibaility of the organisation. Are we generating sufficient revenue to be able to continue doing what is in our charitibly remad and be able to do that for another fiftiy years. Or that we are confident that we can do that for another fifty years.	
Interview 22	10:25		Coming to the next fifty years: what vision is the platform pursuing?

Interview 22		From the early days and you know and currently really our focus is been on organic and organometallic expermimentlly determing the cristal structeres. I think we are recognicing that there are other technices coming along that can enable you to experimetally determing you the 3d structure of molecules. we also recognise there is alot of advances that people computational predict cristal structures.	
Interview 22		So certainly one of our focus is recognicing that the platform needs to grow to be able to support data coming from different sources which will maybe sort of different forms, slightly different structures to what we are used to and potantially quite large. Because we can predict things using computers. And with enough compute power you might be generating a lot of data. I think some of this new technics rather expensive at the moment if they become cheaper can start generating lot of data.	
Interview 22	10:35	Participating grows but also diversiting. I think the (-) balance between how much we rely on software, how much of our revenue comes from licencing software and how much comes through being able to get people to pay for our expertise is something we are looking at. Can we get more of getting the center of expertise than just licencing software.	
Interview 22	12:15	And I think you mentioned, I think we are consciuos there, with this sort of uptaking interest in machine learning and AI, we are actually in a quite well position to offer a quality dfata platform that can people base things on. I think data is quite important when you are doing that sort of aplliing those technics. So it is kind of understanding how we can offer the value that we have in the data in all way that helps with people willing to develope method based on AI, machine learning.	
Interview 22	12:55		Coming to an organisational of my questionare. What is the organisational structure behind the patform?
Interview 22	13:05	We are 70 to 80 people. So. Do you want me to talk about people. Do you want to talk me about - can you just say more about what your expetations are to answer the question.	
Interview 22	13:20		With the organisation I mean, how are you structured - do you have several teams?
Interview 22		Yes. Now it make sense. So, we have a team of, we have a data base team which comprises (-) what we describe is scientific additors and these are typical people who have a backgorund in chemistry. And those are the one who are actually curating the data that comes in, adding the value but there are also involved in several other projects as well. They are the core people to maintaining and develping the data that is in the data base and running the processes that, the depostiion processes and maintaining the workflows, the link get published and things like that.	

Interview 22	13:30	We have a team of software developers who have two key focuses. One is develpoing the scientific software and services that get used externally by the wider community. Also maintainign platforms internally that enable data base group to do their work. And then we have teams of scientist who are sort of undertaking research in various different forms to either advance a product, provide services to industrie and generally support product development wthin the organisation.	
Interview 22	14:50	Then additionally to that will be teams of people who are responsible for the administrastion, finance, HR, the general managament. The majority - oh, one key team I forgot as well: Business Development. Which is i think being the one building up a little bit in the last couple of years. And these are the people who are actually going out into particular industrie because we do rely to 70% of our revenue coming in from industrie at the moment to be able to do what we do. To you know sell our services, our software, and they take care of the marketing as well. That is data software science business developpment and admin. That kind of key five teams. Majority are based in the (country) but we have about five people now who are based in various locations in the us.	
Interview 22	15:45		And what different competences do they have? Those emploxees?
Interview 22		Yeah, I think, they probably broadly represent those sort of / yeah, they probalby divide nicely accros those teams. In the data team we are looking primarily for people who have structural chemistry background. And obviously with aspiritions to curate data in a digital enviroment. We are not yet at a point where we need to go out and recruit neccessarily for data stuarts which I know is becoming sort of profession in the wider worls as well.	
Interview 22	15:52	But maybe in time this will happen. But at the moment the focus is sort of getting those people who do have structur chemistry background. Software developer - some of them do come with a scientific background but increasingly this are people who have experience of developing sotware in a range of different industries prior to us. So the key thing is that making sure they have skills in programming skills and platforms that we are needing to support. With the science team, they typically looking for people who have Phds and interest in strucutal chemistry in various forms.	
Interview 22	17:10	Business Development need people with experience of I guess commercial selling paritcular in scientific enviroment selling to scientific organisations. And I think we have with the admin team suit sort of competences that gonna fit the roles we are looking for, wether it be finance, HR or anything else that fits under that. Does this sufficiently answer your question.	
Interview 22	17:30		Yes of course. You also mentioned the team structure. Now I wonder: How are the different responsibilities distributed? Does the team have the responsibility for the topic or how does it work?

Interview 22		So, I think. The word what is typically used is matrix management. So I say, each of the team has a degree of authonomy and responsibility. Cutting across that is sort of adoption of agil software development methodologies which means that the teams will often bring in. So the software developers are primarly responsible for actually writing the code that makes the software work. But the way the projects run envolve others from the outside of the organisation to provide thatsort of domain experience and feedback of what is beeing developed.	
Interview 22	17:55	If you read up on agile methodoly you probalby be familiar with it but sort of doing sort of like iterations where you try and to deliver something in two week period, review, make sure you make acceptance testing, search etc. , make dummies done. And to make this effectly you need cross- disciplinary teams not just teams with software developers. And I, I guess that, that, that sort of mixing up of people happens mostly aware software development side of things.	
Interview 22	19:06	Probalby in the other teams they tend to undertake there responsibility more in isolation whilst also contributing sort of cross team discussions trough the agile process as well. Isolation is not quite the word, I used that / Autonomies might be better.	
Interview 22	19:28		And how are the decisions made?
Interview 22	19:35	So we have, what we call a senior leadership and management team, SLMT. Who are at the moment kind of main sort of decision making body at strategic level. And they are lead by an executive director or CEO. We have an operations management team which is sort of a cross section people from across the organisation looking at systems and policies and procedures and just general benefit, communication within the organisation, to take that as an example.	
Interview 22	20:00	And than another key team is the product management team who has oversight of the product portfolio. And (-) Responsibilites whether - that is a form as well with discussions particular around how to allocate the software development times goes to what the priorities are for our product. I think those are probably the key decision making (unv) in (platform) at the moment.	
Interview 22	20:40		And then you would say that you discuss the topic and then the majority decides what to do or?
Interview 22	20:52	I would say that in my experience you, (platform) usually decision by consenses. So a topc will be ad, there may be different views on how to proceed. I think, ultimetly, if this decense, someone need to make a decision but you can have always hope that you give ideas of fear hearing and I know from my experience sometimes I thought the right way forward might be path a but someone has chosen path b and its gonna - well that is not what i have chosen but well - but i dont think it is a terrible idea to go done that route. Often we are actualy, we recently sort of, we shared (unv) understanding of what the priorites are that is usualy not to much desense. The short summary is that we aim for consenes.	

Interview 22	22:00		How do you strategically position the platform?
Interview 22	22:05	How do we?	
Interview 22		In terms of what we promoting to the wider community, it is I think, insides is a good word - it is strutural chemistry insides. But also tools to allow you to apply those insides to help you to solve problems that you might be working on. I think we are not necessarlily go out there and say: we have got a platform that can solve all your world problems. I think we gonna got out and say that we have a unique body of knowledge that can help, that can be used in combination with a range of other computational methods and experimental methods.	
Interview 22	22:12	One particular roll of our platforms complace to help assess risk associated with any particular project so. I use the example of someone who is trying to work out how to make a drug product. A tablet which is trying to deliver something. That involves cristalicing something. We can sort of (-) the stability of the particular solid form, the sort of risk factors that might suggest someone needs to do more experments in order to make sure they are not gonna get problems with the manufacting with that - so risk reduction is a key thing to come forward that. Does that help?	
Interview 22	23:45		Do you want to broaden the community size of users or do you want to have a more intense usage in the existence community?
Interview 22	23:55	We definetly want to broaden the community size. And I think, if we go right back to the beginning of that story, when the data base was first being adopted it was by very much specialist people. As time is going on I think you know the become more computational platforms that are more accessible to more scientist across an organisation. That is one of the challenges we face is delivering the products that we have in a way that those who are not expert can very easily take advantage in the knowledge within there. A good example how we want to broaden - I give you a couple of examples.	
Interview 22	24:45:00	One is, so the traditional users in pharmaceutical companies have often been computational chemist, molecular modelist, there is a lot, medicinal chmist, bench chemist could benefit from it from having easy access to some of the insidethat we can provide, just to know what happens if the thing here desinging you know designing in different ways, what impact might it have. So that is one way in which we want to broaden. I think the other way in which we would like to broaden is that we do historically have a strong base in the pharmaceutical industrie. But there are potentialy a lots of chemical based industries out there that can benefit from the knowledge that is within there in sort of designing all sorts of materials. We would like to understand how other indutries might take advante of the knowledge in the data base.	
Interview 22	25:40:00		How is the platform funded?

Interview 22	25:25:00	(-) we are self-financing essentially. We are not in recieve of any governant grands or other funds in support of our core activites. In the very early day we were, but very quickly that revenue stream dried out because people felt we were established in what we are doing. Thanks to the industrie were paying industries into this. So if I describe how is finance today: 70%, well 60-70% of our revenue comes from industrie. Mainly for the licencing of software. Including in that will be also revenue in providing in expert services to industrie, And then 30-40% is coming through academia for licencing the value added software as well. Just one point, I would like to note, there is a, the current revenue makes a certain level of access free. So anybody can come in and download any in someone has deposited with us. We do get a small number of revenues from grands by being involved in research projects.	
Interview 22	27:11:00	But that is ussualy designed funding for specific project. It doesnt got into running the operation cost. From an operational point of view we are self supporting with majority of revenue coming from industry and not insignificant amount coming from academica, but smaller percentage.	
Interview 22	27:38:00		What are specific goals you set?
Interview 22		Sorry can you clarifiy that?	
Interview 22	27:42:00		So my question is what are specific goals of the platform are you setting. Do you have for example a number of licences, a number of data upload, download, a number of I dont know - if you have specific goals you want to achieve?
Interview 22		(unv.) It reamphesizes some of the things we discussed. Broadening usage in organisations and getting it used by morge organisations. I mentioned getting into new industries. But also I think you know there is lots of smaller start-up, sort of organisations even in pharmaceutical where we perhaps havent be able to getting people interested but there is a potential for them to take advantage of what we offer. I think ensuring that we continue to provide comprehensive collection of the data that has beeing published. I think it is easy to forget that but that is still very important to us.	
Interview 22	28:10:00	Ensuring that we maintain and develop the quality of the data there. So that it is fit for the purposes the researchers want to put it to. Ensuring that we are timely in getting the data out there as well. I think you, we sort of thing about this from a more organisational perspective - growing revenue is sort of a kind of consequence that when you are the first one that was talking there about getting more widely used to wider industries. It is quite important that we do grow the revenue. We have to be careful about growths for growths sake but it is important that is enough for a profit organisation that we are not for loss and that we are generating sufficient revenue not only cover our expensive but beeing able to investi n the development in the organisation as well.	
Interview 22	29:57:00	I think if there is any other.	

Interview 22	30:00:00		And how do you control the goals you set?
Interview 22	30:05:00	What do you mean by control in that context?	
Interview 22	30:10:00		Some companies or institutions use KPIs.
Interview 22		yeah, ok. So I think, This is certainly a vary in developing. Our CEO is being in place now for a year - coming for two years. He is very keen on having dashboards with KPIs and things like that. And as an organisations in some areas we do have those. I think in other areas we could probably do better. I can point to varoius - i mean this didnt neccessary manifest themselves in specific goals. I think I was talking a bit more broadly.	
Interview 22	30:20:00	Certainly within the software team there is a sort of dashnpards where you can generate a round, how well the projects is progressing against a sort of estimated(-) estimates how long it should take, number of defects, number of issues resolved, all those sort of things from the user support type of view, how many queries how we dealt with, how quickly have we dealt with them. From a data processing point of view, how quickly are we getting structures reviewed and curated and once when they become public.	
Interview 22		I guess the key ones that probalby relates ed to the broader goals is are we hitting our revenue targets, how we are doing against budget etc.,etc.etc. So, I would say the reality in terms of how we monitoring those goals is a combination of KPIs and dashboards but also then probably the reporting and something on those. Here in case it doesnt come up somewehere else. that it is a none for profict organisation.	
Interview 22	31:30:00	We have a non-executive board of trustees who are responsible for making sure that we aredoing what our missions says we should do. And that we are doing in a way that is financially sustainable. And they are also accountable sort of what the organisation is ultametly accountable to the charity commision to the (country) or (unv.) we have to follow accounts and stuff like that.	
Interview 22	32:30:00		And what are difficulties and challenges in managing the platform?
Interview 22	32:35:00	So. There is always to much to do. So prioritaization, I think is a real challenge. I think as an organisation even without espandind in new areas as I said we want / we do have a diverse range of activities. We have to worry about the data curation. We have to worry about the software development, we have to worry about sort of delivering on scientific cinsultancy and projects. We have to go out there and sell the product. I think just generally balancing the time and energy within the organisation accross all of those area can be a challenge. Making sure we thrive the right balance the kind of academic community who provides us with the data might need and what the sort of wider sort of community of users who provide us with the revenue might need is always a challenge.	

Interview 22	33:40:00	Getting people to park with the money to support it. I think, would not neccessarily quite that lost fund. Yeah. I think (-) just like (-) I suspect any organisation is dealing in software bases is sort of maintaining those and developing the, not letting to much technical (-) build up and what have you. Challenge is associated with it.	
Interview 22	34:15:00		Good. Now we have finished the first blog about the organisation. Coming to platform governance and usage. I know you touched it already a little but just for me: what functionalities and possibilities does the platform offer?
Interview 22	34:40:00	So. Number one one is to provide a comprehensive repository of published cristal structure data specificly small molecules data. Number two is to provide ways of beeing in people in the broadly senses try people many ways of being into access with that data - through free services or more saffisticated ones that we provide. Building on that it is providing, is providing a platform that enables people to access the knowledge that can be derived from that collection of data. And apply that to real world problems and now as well to offer services where we are using that platform to do for people, so they dont have to.	
Interview 22	35:30:00		How do you describe the user and participating institutions?
Interview 22	35:36:00	It is a, it is a mix of both academic and industrial research organisations. So I think we have use of the (platform) in quite a large, quite a large -worldwide basicly - particly in research industries have / are doing chemical research, chemistry research departments. And then in industry particulary in the pharma indistry which is alsways able to take advantage of the knowledge inside the data base. Some smaller usages in other areas (unv.).	
Interview 22	36:20:00		And what difficulties can arise when exchanging data via the (platform) ?
Interview 22	36:30:00	So I think (---) cristallography is a disciplin is quite blessed in / has developed over the years. They have standard formats for the exchange of cristallographic informations. Where the challenges really come is ewhen you want a sort of exchange chemical information there are a lot of different ways of representing a chemical strucutres and lot of different formats and they not always interconvert so easily. So to say that is probably the main area of challenge for us.	
Interview 22	37:05:00	I think there is perhaps also an opportunities which says neccessaraly / yeah, perhaps to advance to do that. But thinking back how we can expose the data that we have in ways that that can be more easily picked by other disciplines, other outsider chemistry chemistry and cirstallography. but this is an area for you know kind of thinking about particular with increasing conversations about interoperability of data across disiplines.	
Interview 22	37:36:00		How do you estimate the reusanility of the data?

Interview 22	37:43:00	So. (-). I think (-) we have a recently good track record of making cristallographic data readily reusably while people working in chemistry. It is easy to think cristallography and chemistry is the same but in a way there aslightly different disciplins, what you get out of the (unv). you want to get chemical insides from all tha there needs to be work that is done to that data.	
Interview 22	38:15:00	I dont know with your question how do you estimate - do you want me to put some number or name?	
Interview 22	38:25:00		No, I mean, I dont know if you have a process of evaluating it or if you dont
Interview 22	38:35:00	I think (-) I think it is something we keep under constant review. One of the (-) one of the sort of features of our data base and data platform is that the data we are dealing with is quite homogenious. That you kind you know the way you treat one data set is going to be the same you treat another data set. That is not neccessarily the case in other data platform where there are getting data from all sort of disciplines and every data set is different. Comparatively all our data sets are going to be pretty much as reusable as each other. So really what we are looking at what work can we do to make more of the information and data and knowledge captured in those available.	
Interview 22	39:25:00		And how do encourage researchers and organisation to actively use the platform?
Interview 22	39:32:00	So. We attend quite a lot of scientific meetings in the cristallography but also chemistry as well. We have recently started running webinars every couple of months - highlighting new features. We periodically release newsletter to tell people about what is coming out and obviously cultivate the list of people are interested in getting those. We are active on social media. (-) we occasionally publish blog posts and things like that to support all this. We recently published a couple of white papers that highlight how the (platform) can used in different ways.	
Interview 22	40:00:00	We have people who will or go out and talk to people in industrie potential new costumers etc.	
Interview 22	40:27:00	I have probably missed a few things. But I think, the whole active campaign across various sort of outlets - our marketing team will always say we can do more- I am sure.	
Interview 22	40:49:00		What other ways do you also see?
Interview 22	40:55:00	I think it is more about the volume of all of those things. I think we can always benefit from having more people with more time to do more visits to costumers, we can always benefit from more people with more time to go to more conferenes etc. So I dont know if I have any other specific things to add, sorry.	
Interview 22	41:15:00		Ok, good. Coming to the submission of data. What requierements do you have for the submission of data?

Interview 22	41:25:00	So. (---) I can probably send you a link that points to the specific - in case it helps (unv.) I can refer to. Essentially we require the data be in a standard file format. That I have refered to earlier. And I think there is a very basic minimum amount of data we need in order for us to usefully include it in a data base but that is as simple as cell parameters. Often or typically that are the coordinates the people are interested but if for some reason people dont have that i think we will accept a determination that is done / that has cell parameters that can sometimes be good enough for some purposes.	
Interview 22	42:10:00	So I think the key thing there is that it comes / beiing provided in a standard file format and that it has very basicm minumum amount of scientific data, (unv.) When someone comes along with a collection of data that didnt not exactly conform with (-) standard file format we probably think about what we can do to get it into the data base. That is the way.	
Interview 22	42:40:00		And how do you sure ensure that people upload data in sufficient number and quality.
Interview 22		So our deposition / we have a web based depostition process which requires people to provide promince meta data. It will encourage people not just derived data but also processed data. It will check the syntax of the data that is beeing uploaded. It will link out to service that is provided by the international union of cristallography called chesif which will check for the completness and consistency of the data and report back.	
Interview 22	42:45:00	And people can at that point either go back and correct that data or write explanations to why you know that might be a particular issue with that particular data set. Trying to capture any publication information that might be associated with the data set. And then (-) I think the final bit will try and capture additionaly scientific meta data items if there are known just, color of the cristal, metlting point things like that. That is a sort of the depostion process depositer goes through. Does that answer that answer the related question?	
Interview 22	44:10:00		Related to quality is trust. So how do you ensure trust in the data?
Interview 22	44:15:00	Yes. We have recently applied to be accrediated to the core trust scheme. So, that is one way in which by going through the application process it gives us the opportunity to reflect on our processes and think are we adopting you know what is considered best practise for a trusted repository. We have actually also recently appointed to some who is a post-doc who is looking at sort of aspects sort of scientific integrity of the data and think if they can be / they can find issues that may be suspectal or also developed various other checks that can be applied in real time as the data is being deposited.	

Interview 22	45:00:00	There have been incidence of fraud in cristallography. If you consider the number of structers that been published it is relatively small but at the same time it is not something that can be easily dismissed. We are trying to use community based checks and we also have our own in-house checks hat will hopefully identify anything that looks suspicious and couple that with sort of processes around good practices from managing data. Hopefully we areble to show the community that we can be trusted.	
Interview 22	45:40:00		And how do you ensure that the user of the platform deal scientificly correct with the use of the data?
Interview 22	45:50:00	I think that is kind of mostly captured in the sort of software that we provide. That is kind of the aims of the software products that sit on top of the data. They make it easy for people wanting to use the data to do the right thing. And I think the curation process that goes on before that that underpins that as we.. So you know when / trying to think / if something wants to know / if somebody wants to know the geogsomething about geometric preferences of a molecule . One of our software packages allows you to just load the molecule, 3D molecule. And you know say based on experimentall data in de (platform) , what is good or bad about that. We have kind of done all the hard work that makes sure that sort of foundation of the methodology there is using the most appropiate data and sort of subject to biases and things like.	
Interview 22	46:50:00	So I think my answer of question would be trough our software products making sure that you know the user gets what they want and doesnt get what isnt helpful.	
Interview 22	47:10:00		One impotrant aspect about (platform) is that it is in the scientific community. How does the culture, the scientific culture, influence the use of the paltform?
Interview 22	47:25:00	So, cristallographiers have been sharing that data from pretty much the very beginning in cristallography because what they would do is they publish an article and they put the coordiantes of the atoms, the atomic position that they determine in the paper. So I think that has that sort of that cultures been cultivate from a very early stage. I think through all organisations such as ourselves and I note here that there is an inorganic cristal data base, there is also the protein data bank you know.	
Interview 22	48:05:00	Between this we are covering from inorganic materials all the way through to biological molecules. I think we demonstrated that there is value in doing this by providing the platform and in some cases undertaking the researches ourselves that shows that you can generate insides through from this data. And I think this is sort of probalby a vicious circle there. You know people share that data, all the community shares that data, the community shows how it can be useful and that encourage people to keep sharing their data.	
Interview 22	48:42:00		What development do you see for the platform over the next ten years?

Interview 22		(---) I think this is gonna be more a personal view than possible an organisational view although I dont think people would disagree with me. I wont repeat what I said before. Ok, now, I will, maybe just three things I said before. Obviosuly differnt types of adfta. There will always be a linkt to 3D structure in there. And you know also anything that can help with structural chemistry. I think, this is something I didnt mentioned before as well, is well as looking at structures thinking about what physical properties it is useful to capture alongside the data as well.	
Interview 22	48:50:00	That is wuite important if you want to build models in a machine learning context. Something else? The move to you know looking at ways, software is a solution of were us using our expertise might be the best soluton to some one. That is more about an organisation thing than a platform. I think on the software front were it is a little more personal but it is more about working on how we can get the knowledge in the (platform) and maybe certain aspects of the functionality that we provide exposed in other platforms that people might be using on a more regular bases.	
Interview 22	50:20:00	So that the bariers to people actually be able to take advantage of this is much much lower. A collegue of mine refers to this (platform) in science thing and it should familiiar with the Intel side. And it got all our computers and all our computers have intel chips inside. Can we have it so that other scientific methodologies kind of have the (platform) inside because their, those methodologies are drawing on the knowledge that the experimental cristallographic data can provide. I think I guess I try and summarise that in concise ways just lowering barriers to access to knowledge that is embedded in the (platform) .	
Interview 22	51:03:00		What does the success of the platform depend on then?
Interview 22	51:10:00	What does the success of the platform depend on? It depends on people continuing willing to share data with the wider communities trough the platform. It depends on us being able to maintain the revenue that supports the platform. It depends on the scientific community understanding the value of cristallographic data and knowledge. That is quite an important thing. And it depends on us being able package this up in a way that is attractive to users particular those that paying you know contributing to the bulk of the revenue And again that comes down to reducing the barrier to access.	
Interview 22			Good. Do you have anything else you want to tell me about challenges, success factors - whatever comes to your mind?
Interview 22		(---) So. (---) Ithink one things I sometimes highlight in these sitations is that one of the benefit of the way that (platform) is constitute. There is an independent non for profit organisation. It doesnt benefit from direct funding for our own activities. Which on the one side means we have to work hard to generate our revenue but what it does mean as that we do have the opportunity to be / we do benefit from independence.	

Interview 22	52:21:00	That means that we can - as long as we are running the organisation in a senseful way - sort of being adobtable. So if circumstances change we can more easily I think sort of change what our focus ist. We also been lucky enough to build up some financial reserve over time - so if we need to invest a little bit of extra money in beeing able to sort of you know perhaps development one particular thing we can do that. I think it is / the thing what I am highlighting here is that the organisational structure that provides / that sourrounds the platform is important to reflect on. (-) That has challenges but it also provides opportunities.	
Interview 22	54:00:00		Thank you for your insight.
Interview 23	Time	Interviewee 23	Interviewer
Interview 23	06:03		Und was ist jjetzt das Kernangebot von der Plattform?
Interview 23		Das Kernangebot sind anorganische, kristallographische Daten. Mittlerweile noch ein bisschen breiter. Also früher rein anorganisch, mittlerweile nehmen wir eben die Metallorganik mit dazu und die sogenannten theoretischen Strukturen. Also die Strukturen, die sozusagen aus Berechnungen sich ergeben und Strukturen zu optimieren oder neue Materialien zu erforschen und so weiter.	
Interview 23			Und meine (work) handelt ja nun von Erfolgsfaktoren von Plattformen. Was ist denn für Sie Erfolg bezeihungsweise eine Erfolgreiche Plattform?
Interview 23	06:51	Das kann man natürlich unterschiedlich definieren. Das eine ist, wenn sie eine, eine, eine kommerzielle Plattform haben, wie es die (platform) ist, können Sie es natürlich auf Basis der Erlöse definieren. Das würde ich jetzt persönlich (lachen) nicht unbedingt nur als befriedigend ansehen, weil ich / es ist natürlich schön, wenn man Geld einnimmt, das ist auch eine Bestätigung, das muss man auch so sagen, also wenn Leute Geld in die Hand nehmen und für eine Plattform, für solche Daten bezahlen, dann weiß man natürlich auch, adsss Sie damit zufrieden sind. Aber letztendlich ist die Nutzung der Plattform natürlich, sage ich mal, für uns glaube ich das größere, der größere Erfolgsfaktor.	
Interview 23		Also je größer die Nutzung wird, desto mehr erkennt man eben, dass die / dass wir das, was wir anbieten, auch für den Kunden (-) interessant ist. Und das wiederum macht ja den Erfolg dann am Ende aus. Also wenn Sie fragen: kauft der Kunde ein.	
Interview 23	07:50		Welche Vision wird denn mit der Plattform verfolgt?
Interview 23		Vision. Also wir (3) Das ist ja eine interessante Angelegenheit, dadurch dass (platform) so alt ist. Man hat sich ja früher verstanden als reine Nachweisplattform. Also wir, genauso wie das Depot hier bei uns im Keller, hat man dann irgendwann angefangen, die Daten zu erfassen - und die wurden ja wirklich manuell erfasst. Mittlerweile gibt es da ja ganz andere Methoden, das passiert eher elektronisch, aber (-) aber man muss sich jetzt auch glaube ich / die Zeiten haben sich ja weiterentwicklet, wir müssen uns einfach weiterentwickeln, also wir sehen uns als (-) Tool für die Materialforschung.	

Interview 23		Das heißt, wir wollen uns dahingegend entwicklen, dass wir eben nicht nur Daten nachweisen, sondern dass wir die Daten auch so nachweisen, dass sie für zum Beispiel Data Mining Prozesse möglich optimal genutzt werden können. Und damit auch eben direkt für die Materialforschung, also für die Erforschung neuer Materialien, nicht nur für den Nachweis bestehnder MAterialien dienen, sondern wirklich für die Erforschung neuer Materialien mit neuen Eigenschaften, mit verbesserten Eigenschaften, günster - was auch immer - gibt ja alle möglichen Kriterien. Da wollen wir hinarbeiten, also das ist unsere Vision. Und so wollen wir uns positionieren.	
Interview 23	09:27		Damit haben wir schon den ersten, kleinen Frageblock beendet. Jetzt möchte ich nämlich eher auf die Richtung gehen, wie ist das organisatorische hinter der Plattform. Und Sie haben jetzt ja schon das Positionieren angesprochen. Wie wollen Sie sich denn Positionieren?
Interview 23		Also wie ich, also wie ich das sozusagen in die Kommnity trage, wie ich das machen möchte oder wie?	
Interview 23			Oder die Plattform im Verhältnis zum gesamten Datenmarkt, was es da so gibt.
Interview 23		Ok gut, jetzt hoffe ich, dass ich die Frage richtig verstanden habe. Ich meine (platform) , das hatten wir ja nun öfters, gibt es ja nun schon relativ lange, insofern ist (platform) auch schon sehr gut positioniert- erfreulicher weise.	
Interview 23	10:00	Also ich würde es in diesem Sinne dann auch als erfolgreiches Produkt bezeichnen und (-) das heißt, wenn ich jetzt sozusagen meine Vision ändere, oder (-) erweitere -sagen wir mal erweitern, ändern kann man da nicht sagen - dann muss ich das in Community trage.	
Interview 23	10:30	Das heißt, ich muss erstens natürlich meine Datenbank entsprechend ausbauen und zweitens muss ich dann anfangen, diese Themen auch mehr und mehr in die Community zu tragen. Das geht über Konferenzen und solche Sachen. Also vielleicht auch, bei (name) planen wir jetzt einen gemeinsamen Workshop mit (platform) genau zum Thema Data Mining. Wir gehen / wollen zusammen gehen, was habt ihr für bedarfe? Also die Community muss natrülich auch befragt werden und ich glaube auch, dass man innerhalb der Wissenschaftscommunity Erkenntnisse ergeben, wie man Daten auch nutzen kann. Weil es sind ja immer zwei Seiten: das eine ist, man nutzt ein Produkt immer irgendwie, ich sage mal, viele Leute nutzen es sehr konservativ.	
Interview 23		Man macht das schon mmer so - (platform) wird in der Messung genutzt und fertig. Und die Überlegung, dass man eigentlich diese Daten auch für andere Zwecke, gerade auch Data Mining nutzen kann, die muss ja auch in ddie Community getragen werden. Das / Ich glaube, das geht nur so durch eine Communication innerhalb dieser Wisenschaftscommunity und genau das müssen wir tun. Also wenn Sie das darunter verstanden haben, wie wir uns Positionieren wollen, dann würde ich das so sehen.	

Interview 23			Ist es denn eher so, dass sie dann die Community wesentlich vergrößern wollen oder in der Community stärker ausbauen?
Interview 23	12:00	Ich glaube beides. Ich glaube beides. Dadurch, dass man die Nutzungsformen erweitert und dass diese ganze Thematik Data Mining ja nun absolut gehypt wird - und es ist ja auch wichtig. Da muss man / Das ist jetzt ja nicht ein Hype wo man sagt, das ist Mode, das ist eine kurze Modeerscheinung. Das ist sicherlicht etwas, was die Arbeitswelt auch verändert. Und. Und dadurch wird sich auch der Nutzerkreis erweitern, weil es einfach andere Leute sind, die diese Daten nutzen also auch mit einer anderen Ausbildung möglicherweise. Sowas wie Chemieinformnatiker, solche Leute kommen ja plötzlich auch ins Spiel, das ist ja noch eine andere Gruppe.	
Interview 23	12:45	Also ob es sich letzendlich sage ich mal, wenn wir jetzt wieder über den monetären Ansatz nachdenken, ob es sich letzendlich durch eine irrsinnig hohe Zahl neuer Subskription auswirkt oder zeigt, das weiß ich garn icht, weil wir sind gut positioniert. Aber es wird glaube ich eine höhere Nutzung gehen. Also im Sinne von mehr Menschen. Mehr Wissenschaftler, der Kreis wird erweitert. Aber wenn sie eine Lizenz haben für eine Universität. Dann ist die Lizenz halt für die Universitäten - ob da zehn oder hundert Leute dran arbeiten ist dann erstmal egal. Von daher kann ich gar nicht sagen, ob wir viel mehr Lizenzen verkaufen werden, aber ich glaube, dass die Anzahl der Personen, die es nutzen, größer wird	
Interview 23	13:30		nun haben Sie ja schon die Lizenzen angesprochen. Wie wird die jetzt finanziert, die Datenplattform.
Interview 23		Es ist eine rein kommerzielle Plattform, dass heißt unsere Erlöse werden dazu verwendet, diese Datenbank zu erstellen. Also (institute) ist ja eine Non for Profit Einrichtung (-) und genauso ist es dann letzendlich auch. Also das, was wir erlösen, setzen wir tatsächlich auch wirklich für die Erstellung der Datenbank ein. Das ist jetzt nicht so, dass wir eine Million irgendwie Euro plus machen, aber wir können die Datenbank damit produzieren und das ist schonmal sehr gut.	
Interview 23			Und die Erlöse kommen dann von den Lizenzen, oder haben sie auch Partner, die da mitreinspielen?
Interview 23		Ne, das sind nur Lizenzen.	
Interview 23			Gut. Wie ist denn der organisatorische Aufbau hinter der Plattform?
Interview 23	14:32	Bei uns? Also innerhalb vom (institute)s?	
Interview 23			Ja
Interview 23		Also ich glaube, Sie können das Organigramm auch grundsätzlich auf unserer Website finden, sodass / ich habe ihnen ja schon geschrieben. Mein Bereich ist dafür zustädnig, diese Datenbank zu Produzieren und auch zu vertrieben. Also Marketing, Vertrieb und Produktion ist in einer Hand. Und wir haben einen Bereich IE. Das ist (3) Infor / (5) / das ist peinlich (lacht).	
Interview 23			Alles gut.

Interview 23		Das ist ein Entwicklungsbereich. IT-Infrastruktur und Entwcklung heißt das glaube ich - also das ist ein ganz langer /. IT- Infrastrauktur - ja egal, Sie können das bitte nachgucken oder ich schreibe es ihnen.	
Interview 23			Ich werde mal gucken, ob ich es finde, sonst melde ich mich noch bei Ihnen.
Interview 23	15:20	Es gibt nur einen Bereich, der mit Etnwcklung zu tun hat. Und in diesem Bereich wird / findet die Entwcklung statt. Also die Plattform sozusagen der technische Background von der Plattform.	
Interview 23			Wie viele arbeiten dann für das (institute)?
Interview 23		Fürs (institute)? Am (institute) selber sind wir ja 300, 320 Mitarbeiter und fürs (platform) - ist jetzt auch eine Frage (5) schwierig zu beantworten ist, adhioc. Ich würde mal sagen, um die zehn Personen arbeiten an der (platform) unterschiedlicher Koleur. Wenn Sie es genau wissen wollen, müsste ich jetzt nochmal nachgucken. Das sind solche Zahlen, die gucke ich, die habe ich nicht permanent parat. Es gibt eben auch Leute, die arbeiten teilweise dafür und so weiter.	
Interview 23		Das ist nicht so (-) ich kann das jetzt nicht so spontan sagen. Also gerade / es wechselt halt auch. Der eine macht mal das mal jenes. Gerade im Entwicklungsbereich gibt es eben mal Schwerpunkte, dass ein Mitarbeiter mehr dazu kommt oder einer weniger. DAs kann ich jetzt ganz spontan nicht sagen, aber ich würde jetzt schätzen, dass sind so zehn, zwölf Leute.	
Interview 23			Die dann die Plattform aus der technologischen Seite aufbauen oder komplett ebtreuen?
Interview 23	16:34	Ja komplett. Technologisch und eben auch Marketing, Vertrieb und Produktion - also der reine Dateninput muss ja auch gemacht werden, was auch der größere Input ist.	
Interview 23			Das heißt, was für Kompetenzen weisen die dann so auf?
Interview 23		Unsere Mitarbeiter sind / also wir haben im Grunde, im Grunde haben wir drei Grupen, würde ich sagen. Wir haben die Fachangestellten für Medien und Informationstechnologien heißen die- FaMi. Das sind die Leute, die sich um die Datenerfassung, um die Datenstandardiesierung, die Erfassung, die Beschaffung der Literatur, Erfassung bibliographischer Daten usw. kümmern. Dann haben wir eine ganze Reihe Leute, die sind Chemiker. Die machen den fachlichen Input und machen auch den Vertrieb. Der Vertrieb von solchen Produkten ist ja, da wir ja die wissenschaftscommunity ansprechen, sollte man auch Chemiker sein, der sich irgendwie damit auskennt.	
Interview 23		Von daher machen die zum Teil auch beides - also das ist eine Mischform an Tätigkeiten. Und wir haben auch Informatiker. Ich glaube, es ist auch ein Chemieinformatiker dabei. Die, die Datenbank dann / also die sozusagen die Plattform bereitstellen. Bei mir im TEam sind, sozusaghen in deR Produktion ist auch ein Kollege, der auch, sagen wir mal, der ist von Haus aus Chemiker, kann aber auch sehr gut entwicklen.	

Interview 23	18:00	Der entwickelt das Inputsystem. Also das ist auch noch / sie müssen ja die Daten ja irgendwie zwischenspeichern. Und der macht das auch noch nebenbei.	
Interview 23			Ok, also tendenziell naturwissenschaftler Hintergrund.
Interview 23		Ja, also der überwiegende Anteil ist naturwissenschaftlicher Hintergrund.	
Interview 23	18:20		Wie sind denn die verschiedenen Verantworlichkeiten verteilt?
Interview 23		Es ist im Endeffekt so, dass / es gibt mich (lacht). Ich bin eben sozusagen verantwortlich für den Vertrieb und die Produktion und es gibt einen Kollegen, der dann verantworlich ist für die Entwcklung. Die Planung, also wenn es jetzt um die Gesamtplanung angeht. Also letzendlich sind wir natprlich dafür verantwortlich aus fachlicher Sicht zu sagen, wir brauchen das oder jenes. Und die Entwicklung kann dann sagen, können Sie das, können Sie das nicht, oder in welcher Zeit. Und darüber diskutiert man halt. Und dann werden die Prioritäten festgesetzt und dann kümmert sich der Kollege um sienen TRupp und ich mich um meinen Trupp und die treffen sich auch unterwegs zwischendurch- das ist ganz klar - auf der fachlichen Ebene. Aber das ist dann die Verantwortlichkeit.	
Interview 23			Und wie werden
Interview 23		Über allem gibt es natürlich noch die Geschäftsführung	
Interview 23			Ja, ok.
Interview 23		Nicht vergessen (lacht)	
Interview 23			Und wie werden dann die Entscheidungen getroffen.
Interview 23	19:30	(4) Im Endeffekt / Ich habe ja gesagt, zwischen Entwicklung und (-9 und Produktion oder Marketing, Produktmanagement nenne ich das jetzt mal, werden sie gemeinsam getroffen. Und was jetzt rein Content angeht, wird die Entscheidung bei uns im Team getroffen im Ende. Also da ist es natülich wichtig, dass sie (3) also sie müssen ja abwägen, Aufwand - Nutzen. Also sprich, braucht der Kunde das, braucht er das nicht, wie hoch sind die Aufwände um bestimmte Inhalte darzustellen. Und dann müssen sie letztendlich entscheiden, lohnt sich das oder können wir uns das leisten oder geht es halt nicht	
Interview 23	20:00	Aber das erfolgt eigentlich im Team - dazu gehören dann natürlich auch das Produktionssystem, weil wir müssen das ja darstellen, weil wir müssen auf zwei Seiten sozusagen die Daten, die Funktionalität darstellen können. Einerseits eben im Produktionssystem und dann müssen Sie es hinterher auf der Plattform darstellen können, dass der Kunde das auch nutzen kann. Also nützt ihnen ja kein Inhalt was, der nicht suchbar. Und von daher, das sind so (-) Diskussionen im Team im Endeffekt.	
Interview 23	20:50		Welche konkreten werden denn jetzt mit Plattform verfolgt und wie erfolgt die Kontrolle der Ziele?

Interview 23		(5) Ich denke erst, nicht dass ich keine Ziele hätte, sondern nur wie ich die Frage zu verstehen habe. Ich habe es ja vorhin gesagt, wir wollen (platform) als DIE Plattform im Bereich der anorganischen Kristallstrukturen für die Materialforschung zu positionieren - das ist das übergeordnete Ziel.	
Interview 23	21:20	Dazu werden eben untergeordnete Ziele verfolgt. Das eine ist die Datenqualität, die vollständigkeit der Datenbank etc. Dann natürlich auch die, die (-) Nutzerfreundlichkeit. Also das Ding muss ja auch benutzbar sein. Und das (-) beides dient am Ende dann dazu, dieses Ziel zu erfüllen.	
Interview 23			Wie bewerten Sie das dann?
Interview 23		Wie ich das bewerte? Also ich meine, Datenqualität bzw. den Content der Datenbank kann ich realtiv einfach bewerten. Also wir haben (4) ne, es ist, ja, so einfach ist es natürlich nicht. Wenn ich den Content bewerte, also wenn ich die Vollständigkeit einer Datenbank bewerte, dann brauche ich natürlich eine gewissen Basis, auf derer ich bewerte, bin ich vollständig oder nicht. Ich kann das gar nicht beurteilen, weil ich weiss natürlich, letztendlich gibt mir keiner die Zahl, so undso viele Kristallstrukturen wurden in (year/number) veröffentlicht und ich kann gucken, bei mir habe ich sie alle oder nur 80%.	
Interview 23	22:25	Aber natürlich, dadurch dass man das schon dreißig Jahre macht, weiß man in etwa, welche Zeitschriften isnd da. Man kann natürlich in gewisser Weise kontrollieren, ist die Zahl, die man erfasst steigend oder sinkend, und wenn sie steigend ist, kann man advon ausgehen, wenn man davon ausgeht, wenn man möglich alles erfasst, was irgendwie sichtbar ist, dass man dann vollständig ist und wenn die Zahl sinken muss man sich überlegen, woran es liegt. Also das ist das, was wir tun. (-) Ansonsten, Datenqualität wird immer wieder auch durch automatisierte Prüfungen überwacht, was wahrscheinlich alle machen, irgendwie. Was war die andere Frage?	
Interview 23			Wie Sie die Erfolgskontrolle
Interview 23		Ansonsten gibt es solche Maßnahmen, also Erfolgskontrolle im Sinne von Erfolgreich am Markt hat was mit Erlösen und Lizenzen zu tun, das sind Erfolgkriterien im Endeffekt. Also, sind diese zwei Seiten: immer Content und Erfolg am Markt.	
Interview 23	23:30		Welche Schwierigkeiten und Herausforderungen sehen Sie denn für die Plattform? Sei es jetzt im organisatorischen oder auf der Plattform.
Interview 23		Also ich glaube, dass die Herausforderung generell im Bereich Datenbanken liegt in den kostenfreien Angeboten und der Akzeptanz (-) von Kostenplfichtigen angeobten und letzlich dann damit einhergehend Qualitätssicherung. Also wofür werden wir bezhalt? DAss wir genau diese Kontrollen machen, dass wir versuchen vollständig zu sein, dass wir versuchen, die Daten standardisiert und korrekt darzustellen.	

Interview 23		DAss wir das auch regelmäßig überprüfen. (platform) ist eine Datenbank, wo wir regelmäßig Daten korrigieren, wenn wir sehen, dass wir Fehler machen. Oder was versäumt haben oder das wir inhalte nicht erfasst haben. Wir verfasen auch immer weider Altdaten weil plötzlich ist irgendwas auffinbar. Und dafür muss der Kunde im Endeffekt bezahlen. Wenn sie das machen, wenn sie diese zehn Leute beschäftigen, dann müssen sie dafür bezahlen.	
Interview 23	24:38:00	Wenn sie kostenfreie Services haben, die dann mal mehr mal weniger vollständig und mal mehr und mal weniger Funktionalität anbieten, dann stehen sie mit denen in Konkurrenz und dann stellt sich die große Frage, wie sich diese Konkurrenz im Laufe der nächsten Jahrzehnte oder auch vielleicht nur einem Jahrzehnt entwickelt. Und wie hoch die Akzeptanz bleibt adfür zu bezahlen. Und das ist glaube ich die große Herausforderung. Und dafür muss man glaube ich auch was tun.	
Interview 23			Im Sinne von?
Interview 23		Im Sinne von Produktweiterentwicklung und Kommunikation. Man muss dem Nutzer auch verständlich machen, wofür er bezahlt. Gerade bei (platform) ist es so, dadurch, dass wir auch das Depot haben und Wiossenschaftler sozusagen in das Depot einstellen - freiwillig - und da auch wieder rausholen. Dann kommt natürlich gerne auch mal die Diskussion hoch, wo dann gesagt wird: ja, wir stellen ja unsere Daten bereit und warum wollt ihr dann auch noch Geld dafür?	
Interview 23		Das trifft im übrigen nicht nur uns sondern auch die Kollegen in (town) oder auch (institute), in den (country), die alle diesen Herausforderungen entgegenblicken. Und dann klar zu machen, dass da eine ganze Menge an QUalitätssicherung im Hintergrund steht oder eben auch Funktionalitäten, die man anbietet, die man entwickelt, dass sowas halt Geld kostet (lacht leicht).	
Interview 23	26:05:00	Das ist glaube ich eine Frage der Kommunikation und das ist auch eine Herausforderung in der Zukunft - die sehen wir auch alle gemeinsam, glaube ich. Dass man da auch was tun muss und Verständnis dafür entwicklent muss.	
Interview 23			Nun haben Sie ja schon die Funktionaltität angesprochen. Auf der Website kann ich natürlich auch schon viel sehen. Können Sie nochmal für mich kurz zusammenfassen: was bietet die Plattform für Funktionalität und Möglichkeiten?
Interview 23		Oh, jetzt erwischen sie mich auf dem ganz falschen Fuß. Weeil wie gesagt, also ich bin jetzt nicht die. Also ich bin die Fachfrau für die Produktion, aber nicht für die Funktionalität für die Funktionalität. Vor allem bin ich auch nicht sicher, ob das was ich sage, so vollstädnig ist. Sie haben natürlich bei (platform) diverse Suchfunktionen nach allen möglichen Datenelementen. Sie können die natürlich in jeglicher fast suchen.	

Interview 23	26:35:00	Sie können nnach Elementen suchen, sie können nach Elementzahlen suchen, sie können sowieso natürlich nach chemischen Formel suchen - obwohl die Suche nicht sonderlich gut ist- , sie können nach den bibliographischen suchen, wir haben Daten standardisiert. Also die Kristallstrukturdaten standardisierten. Sie können also auch nach den standardisierten Formen suchen.	
Interview 23	27:25:00	Wir haben die Kristallstrukturen in gewisse Klassen getan - das nennen wir Strukturtypen. Das heißt sie können auch nach Klassenkkonstruktionen suchen. Also sie haben wirklich alle möglichen Möglichkeiten nach Daten zu suchen. Sowieso natürlich Kristallstrulturspezifische Inforamtionen können gesucht werden. Sie können sich die Daten ausgeben, sie können sie zum Teil auch natürlich Kristalltrukturen graphisch darstellen. (4) Sie haben (-) ich sage mal, wenn wir jetzt wieder auf die Bibliographie gehen, sie haben natürlich dann auch die Möglichkeit direkt auf den Volltext zuzugreifem, wenn sie das Abo haben. Ich weiss nicht / Sie können Daten exportieren. Wir arbeiten daran, dass man mehr und mehr Formate exportieren kann - also solche Sachen.	
Interview 23			Ja, das war schon sehr umfangreich (lachen) sehr gut.
Interview 23			Was ist dann / wie ist denn die Nutzergruppe der Plattform? Wer nutzt sie?
Interview 23	28:35:00	Hochschulen. (-) Also es gibt natürlich auch Industriekunden auch. Es gibt natürlich auch Industriekunden, die im Forschungsbereich arbeiten, denken wir nur an Batteriehersteller und ähnliches. Aber der überwiegende Teil unserer Kunden sind Hochschulen und Forschungseinrichtungen.	
Interview 23			Welche Schwierigkeiten treten denn beim Datenaustausch auf? Sei es beim Hoch- oder Runterladen?
Interview 23		Jetzt für den Kunden oder in deR Produktion	
Interview 23			Für sie.
Interview 23	05:15	Für mich. Für uns. Also wir haben unterschiedliche daten, die wir in (platform) verwenden, also elektronische Daten, die wir in (platform) verwenden. Wir haben einerseits die Zip-files - also die Kristallstrukturfiles, die natürlich von den aus den Messungen generiert werden, da (-) die sind halt mal mehr mal weniger vollständig, da müssen sie Daten nochmal korriegieren oder bei den Prüfungen stellt sich heraus, dass (-) irgendein Datum in diesem Datensatz so nicht sein kann - dass da irgendwas verkehrt ist. Dann müssen wir halt gucken, einerseits, wir korrigieren sehr wenig, wir kommentieren mehr. Wir sehen uns nicht in der Lage, oder wir wollen es auch nicht, wir wollen die Originaldaten behalten. Ich glaube, wenn es ein ganz offensichtlicher Tippfehler ist oder so, dann wird es auch mal korrigiert. Aber eigentlich wird kommentiert.	

Interview 23	30:00:00	Und wir schreiben: das kann so nicht sein, aber das ist in dem original Datensatz enthalten. Das ist das eine. Also die Kristallstrukturdaten können Fehler enthalten und das muss geprüft werden und (-) sind manchmal auch im falschen Format. Und das andere sind, wir haben eben Verlagsdaten, weil wir die dazugehörige Publikation miterfassen und da ist es halt so, dass sie auch ihr Format ändern und dann haben sie plötzlich wieder ein Problem, das Zeug zu importieren, also das sind so die wesentlichen Probleme. Aber eigentlich ist das (husten) ist das mit den Jahren immer besser geworden.	
Interview 23	32:00:00		Wie schätzen sie denn die Nachnutzbarkeit der Daten auf der Plattform ein?
Interview 23		Sehr hoch.	
Interview 23			Im Sinne von?
Interview 23		Im Sinne von, wir haben, wenn die Daten auf der Plattform sind, sind sie in einem einheitlichen Format, sie sind struktueriert, sie sind (-) also absolut standardisiert. Das heißt, sie können mit diesen eigentlich, wenn sie sie exportieren können sie alles damit machen. Nachnutzbarkeit (2) im Sinne von (5) ja, also auch im Sinne von (5) wie soll ich das jetzt sagen, ja Vollständigkeit. wenn sie darüber nachdenken, gerade wenn wir über Data Mining Prozesse nachdenken, geht es ja auch um große Datenmengen, auch das ist gegeben. Ich glaube, die (platform) ist die größte Datensammlung im Anorganischen Bereich - also sehr hoch, ja.	
Interview 23			Welche Regeln unterliegen denn die Nutzer auf der Plattform?
Interview 23	38:05:00	(5) Sie, also, sie müssen die Nutzungsbedingungen von uns beachten. Und das ist eigentlich alles gut.	
Interview 23			Ist es denn so, dass auch direkt zitiert wird - also so bestimmte Vorgaben bei der Datennutzung?
Interview 23		Wir geben keine Vorgaben. In der Regel wird (platform) schon zitiert - ob das durchgängig so ist, aber da machen wir keine Vorgaben, nein.	
Interview 23			Das heißt, die rechtlichen Bedingungen, die sie stellen, ist dann die AGB?
Interview 23		Ja.	
Interview 23			Gut.
Interview 23			Wie wird denn das Vertrauen in die Daten sichergestellt?

Interview 23	33:01:00	Das Vertrauen der Kunden (5) das ist eine gute Frage. Ich meine, wie kann man sowas, wie kann man das Vertrauen in, von Kunden sicherstellen? Ich glaube nur dadurch, dass (2) also dadurch, dass man eine gewisse Kontinuität hat, also dass man auch sicherstellt, dass eben zum Beispiel Updates auch regelmäßig erfolgen und auch eben vergleichbare Größenordnungen. Also dass man nicht mal 2 Datensätze hat und mal (year/number) oder so. Also dass man daran erkennt, dass ein Kunde das einigermaßen, vertrauenswürdig, also die sind vollständig, also dass man relativ aktuell diese Datenbank haält. Also dass man auch schnell die Daten - so schnell wie es geht. Bei (platform) haben wir zwei Updates jährlich. Das ist nicht wie bei anderen Datenbanken, wo es tägliche Updates gibt.	
Interview 23	34:10:00	Aber trotzdem. Wenn ich im zweiten Halbjahr dann nur Daten, wenn ich über das zweite Halbjahr (year/number) rede und ich bringe nur Daten aus (year/number) rein, dann fragt sich der Kunde auhc: was soll das? Ansonsten glaubei ch, dass uns sehr viel hilft, dass wir das seit vielen, vielen Jahren machen und dass wir da auch bekannt sind. (platform) ist auch eine Hausnummer, also eine Marke. Also. Und solange sie das Vertrauen nicht verspielen, indem sie einfach eine schlechte Qualität liefern, behalten sie das auch. Das ist ein Ruf, den man verliert. Und ich glaube auch, dass imuss man eifnach sicherstellen, dass man da ordentlich arbeitet. Und das versuchen wir. Ist einfach so.	
Interview 23		Wir waren die ersten auf dem Markt und dann spricht sich, das ist eine Mund zu Mund Propaganda. Und solange sie dieses Vertrauen nicht erschüttern (2) behalten sie es - ich glaube, so ist das.	
Interview 23			Nun haben Sie genau perfekt auf meine nächste Frage übergeleitet. Undzwar mit der Qualität der Daten: wie stellen sie denn sicher, dass die Personen in ausreichender Zahl und Qualität Daten in ausreichender Plattform hochladen?
Interview 23		Ja, also wir haben natürlich. Das ist nun typisch Datenbankproduktionsthema. Das ist eine Frage der Quellen, die sie auswerten. Das heißt, über die Jahre - das ist das, was ich vorhin schonmal gesagt habe - sie kennen die Zeitschriften, in denen publiziert wird. Wir haben hier natürlich - nicht nur für die (platform) , das wäre am Ende Datenbankproduktion- auch für andere Themenbereiche, wir haben natürlich Strukturen, wo wir wissen: also wir überwachen regelmäßig Prospekte von Verlagen, wir/es gibt durchaus mal Newsletter oder ähnliches, wo man solche Informationen bekommt. Die Kollegen, die den IInput machen, tummeln (lachen) sich ja auch in den Bereichen.	

Interview 23		Das ist schonmal was, wo wir sagen, wir haben da Strukuren, wo wir davon ausgehen, dass wir recht zuverlässig Informationen zu neuen Publikationen erhalten. Und andersum ist es so, dass wir natürlich das Depot haben. Und im Depot lagern/ werden ja Strukturen abgelegt. Auch darüber bekommen Sie natürlich Informationen: es gibt diese Struktur und die Wahrscheinlichkeit, dass sie publiziert wird, ist ja auch nicht ganz gering. Und dann kriegt man ja darüber mit: bleibt da noch ein Bodensatz übrig wo man sagt: der wächst und wächst und wächst.	
Interview 23	36:00:00	Oder ist das irgendwie ein Bodensatz, der über die Jahre immer über die Jahre die gleiche Größenordnung hat, das ist ein Zeichen. Und wir machen Recherchen in Literaturdatenbanken und werten die aus. Also vor allem chemical Abstracts, also das ist die Literaturdatenbank, die - vor der wir ausgehen - dass die ziemlich vollständig ist. Und die bringt uns vor allem Dingen die Literatur, die eben aus Journalen, die eben nicht typisch kristallograpisch sind, wo wir nicht oder materialforschungslastig sind, wo eben vielleicht ein zwei mal im Jahr irgendwas publiziert wird und das kriegen sie über Literaturrecherchen MÜHSELIG, aber sie kriegen es raus.	
Interview 23	37:07:00	Aber es wird genügend Artikel geben, die wir nicht erfasst haben - leider. Leider 8lachen) und daas ist aber die Kunst auch bei der Datenbankproduktion, und das hat auch was mit Qualität zu tun, dass sie sich da sehr große Mühe geben. Und das ist auch etwas, was ich hier immer wieder propagiere, dass das eine Aufgabe ist, die wirklich nicht zu unterschätzen ist. Und das ist / wir geben einiges aus der Produktion machen wir auch mit externen Kräften, also mit freien Mitarbeitern. Die Relevanzentscheidung für (platform) würde ich nie rausgeben, weil das ist wirklich ganz wichtig, das ist der Entscheidende Punkt. Wenn sie einen Artikel erkennen, das ist relevant, dann wird der externe Mitarbeiter das draussen immer gut erfassen, aber wenn sie das nicht erkennen oder wenn sie nicht zu viel erkennen wollen, weil sie wollen sie ja viel Input machen, weil sie dann besser bezahlt werden sozusagen.	
Interview 23	37:50:00	Dann ist es auch nicht gut. Also das ist eigentlich der Kern der Qualitätssicherung, dass sie die Relevanz / also sie suchen nach Literatur und die Relevanzentscheidung treffen - würde ich, also so sehe ich es, ist meine persönliche Entscheidung. Einer der wichtigsten Schritte- nicht der spannendste- aber (lachen) einer der wichtigsten.	
Interview 23	38:10:00		Wie stellen sie denn sicher, dass die Nutzer der Plattform die Daten ethisch korrekt verwenden?
Interview 23		Ja, das ist für uns ja eigentlich / das stellen wir nicht sicher.	
Interview 23			Ok.

Interview 23		Das ist für uns eigentlich nicht die Frage, weil wir uns auch nicht im medizischen Bereich sehen. Ich glaube, das ist der Unterschied. Wenn ich mich damit auseinandersetzen würde, speziell, dann würde ich wahrscheinlich anders darüber nachdenken, aber wir sehen uns halt eher im Batteriebereich oder ähnlichem. Und da ist das einfach kein Thema. Aber grundsätzlich wüsst / glaube ich nicht, dass ich es sicherstellen kann. Wie soll ich das tun? (5)	
Interview 23		Gibt es dazu antworten (3)	
Interview 23			Ich bin auf der Suche (lachen)
Interview 23		(lachen)	
Interview 23	39:10:00	Ich weiß nicht, man kann es maximal könnte ich das wahrscheinlich über AGBs regeln. Also indem ich eben, wenn ich mich in diesem medizischen Bereich tummel, dass ich dann eben sage: für diese oder jene Zwecke dürfen diese Daten nicht verwendet werden. Aber ob / damit habe ich es nicht sichergestellt, ich habe es nur mal ausgeschlosen. Sie können den Kunden nicht überwachen, letzendlich. Von daher, sicherstellen ist von daher fast nicht möglich.	
Interview 23			Nun haben sie ja gesagt, dass die Nutzer der Plattform Hochschulen sind.
Interview 23		Ja	
Interview 23			Wie bewegen Sie denn Hochschulen dazu, eine Lizenz zu erwerben und die Plattform aktiv zu nutzen`?
Interview 23		Indem wir eben einerseits ab und zu - das ist ein bisschen zu selten - Publikationen zu (platform) veröffentlichen, indem wir versuchen zu beschreiben, was die Funktionalität ist, unsere Website versuchen wir auch jetzt doch vermehrt so Use Cases darzustellen.	
Interview 23	40:00:00	Das ist was, was wir in diesem Jahr sicherlich noch deutlich verstärken wollen. Also darüber den sozusagen, in dem wir beschreiben, was andere damit machen, sozusagen Neukunde zu zeigen, dass und das kannst du damit machen, das ist glaube ich die Lösung dieser Frage.	
Interview 23	40:30:00	Und das können Sie natürlich auch auf Konferenzen indem sie Produktvorstellungen machen oder eben einen Vortrag halten oder einen Workshop organisieren oder sowas in der Art. Das ist so, dass, was ich sehe, was möglich ist. Wir sind ein kleines Team, das ist alles bei uns ganz schwierig (lachen). Also, ne, ist wirklich so. Das hat natürlich was mit der Menge an Personal zu tun, die ihnen da zur Verfügung steht und auch mit den Finanzmitteln.	
Interview 23		Aber das sind glaube ich die Möglichkeiten, den man hat. Man muss einfach den Nutzen darstellen und dann kriegen sie auch die Kunden. Wir haben (-) sind jetzt mit Sicherheit nicht bekannt als aggresiver Marketing-Part aber trotzdem kriegen wir doch immer wieder Neukunden, die offensichtlich davon überzeugt sind, dass unsere Daten gut sind, ja. Ich glaube aber auch, dass ist eine Community, wo sie nicht aggressives Marketing machen dürfen (lachen)	

Interview 23			Nun haben Sie ja schon die Community angepsrochen. Das besondere dieser wissenschaftlichen Daten ist, dass sie in die Wissenschaft eingegliedert sind. Inwieweit glauben Sie, beeinträchtigt die wissenschaftliche Kultur die Nutzung der Plattform?
Interview 23	41:30:00	Das müssen Sie mir erklären.	
Interview 23		Was ist die wissenschaftliche Kultur.	Also die Wissenschaft / Es gibt ja Domänen,in denen werden sehr sehr viele Daten schon immer geteilt, auch die Kristallographie- Oder es gibt ja auch Bereiche, in denen wir immer alles eher zurück gehalten und so lange es geht unter Verschluss bleiben. Inwieweit sehen sie da die wissenschaftliche Kultur, die auf ihre Wissenschaftliche Datenbank einfließt?
Interview 23		Ja, das habei ch ja schon gesagt, im Endeffekt haben sie das jetzt selber beantwortet jetzt. Dadurch, dass es das (platform) schon so lange gibt und die Kristallographie offensichtlich - ich habe da auch irgendwann ein Artikel darüber gelesen- eine der ersten Wissenschaften waren, die dieses Thema Forschungsdaten und Nachnutzung von Forschungsdaten wirklich kultiviert geradezu, und das eben schon seit vierzig Jahre im Endefeffekt versucht - damals eben auf Papier, heute digital - das zu gewährleisten. Ist das glaube ich keine Fragestellung in der Kristallographie. Wie es aussieht im Pharmazeutischen Bereich oder industriellen Bereich, da glaube ich wird das schon eher unter Verschluss gehalten. Aber im reinen universitären Bereichen, was (platform) angeht, glaube ich, ist das sehr offen.	
Interview 23	43:15:00	Aber wie gesagt, dass was nicht veröffentlicht wird, werden wir nie erfahren, also.	
Interview 23			Welche Entwicklung sehen sie denn für die Plattform in den nächsten Jahren?
Interview 23		Ja, ist ja auch das, was ich vorhin gesagt habe. Wir wollen uns Positionieren, wir wollen in Richtung Text- und Datamining und wir wollen (-) die Inhalte, wenn es sich ergibt, weiter erweitern. Wir wollen möglichst viele Datenformate bereitstellen, sodass eben Text- und Data Mining gewährleistet ist, das ist eigentlich. Wir wollen im endeffekt genau das bleiben, was wir sind, nämlich DAS tool für die anorganische Materialforschung. Wir müssen diesen Status erhalten.	
Interview 23			Und wovon hängt dann ihr Erfolg ab?
Interview 23		Von der Zahl Nutzung oder der Zahl der Lizenzen - das ist auch das, was wir am Anfang gesagt haben. Das sind die zwei Sichten meines Erachtens.	
Interview 23	44:15:00		Haben Sie sonst noch irgnedetwas, was sie mir zur Plattform erzählen möchten ,sAchen die sie beschäftigen in dem Bereich. (3) Was Ihnen noch so spontan einfällt?
Interview 23	44:21:00	Ne. Glaube nicht, glaube sie haben ziemlich umfrangreich gefragt, ja. Ja. Fragen sie eigentlich rein kommerzielle Plattformanbieter oder auch freie? Auch freie?	

Interview 23			Ja, genau. Also das Hauptkrietrium ist halt, dass man Daten hochladen kann in irgendeiner Art und Weise.
Interview 23		Bei uns können Sie aber keine Daten hochladen.	
Interview 23			Ja, aber es gibt ja dieses Hochladeportal mit der (platform).
Interview 23		Das ist das, was ich vorhin sagte, das ist das Depot. (platform) wird aus dem Depot zum Teil gespeist, mittlerweile tatsächlich zum großen Teil. Aber es ist nicht nur das Depot. Also wir haben mehr Daten als im Depot bzw. besser aufbereitete Daten und eine wesentlich höhere Suchfunktionalität. Das Depot ist absolut frei, aber damit ist auch die Suche eingeschränkt	
Interview 23		Das Depot soll eigentlich nur dazu dienen (-) ich sage jetzt einfach mal- das ist eine sehr eingeschränkte Sicht und so wird es auch nicht nur genutzt - aber im Endeffekt soll das Depot dazu dienen, wenn sie eine Struktur in (platform) oder (platform) gefunden habnen, dass sie dann auch im Zweifel die Originalmessung finden. Oder wenn sie wissen, ich suche Struktur X aus einer Pubklikation, dann können Sie die darunter laden.	
Interview 23	45:50:00	Aber für die Suche in Kristallstrukturdaten sind eigentlich die beiden kostenpflichtigen Datenbanken gedacht - und so werden die auch genutzt.	
Interview 23			Was ist dann der wesentliche Unterschied zwischen den beiden? Also zwischen dem Depot
Interview 23		dem Depot und (platform) ? Ist einfach so, das Depot da (-) da lädt der User, also der Wissenschaftler selber seine Daten hoch. Diese Daten sind dann kostenfrei auch wieder durch andere Nutzer runterzuladen. Allerdings muss der Nutzer eben Zugang haben. Die Suchfunktionalität ist deutlich eingeschränkt. Sie können jetzt nicht sagen, ich suche (2) sie können glaube ich nach Literatur suchen, also wenn sie eine Literaturstelle haben, können sie suchen.	
Interview 23		Sie können glaube ich aber nicht sagen, ich suche mir alle Strukturen mit element X,Y aber nicht mit Element D. Das geht nicht. Also sie haben eine deutlich eingeschränkte suchfunktionalität. Inhaltlich ist es so, dass wir einige Daten noch dazu schreiben, bei der Generierung. Und die Daten natürlich generell prüfen. Das heißt, was wir vorhin hatten, vielleicht nicht unbedingt korrigieren aber zumindest mal kommentieren.	
Interview 23	47:10:00	Und auch (-) gut, wir haben mittlerweise gesagt, wir schieben auch Daten, die nicht im Depot abgelegt sind ikn reduzierter Form aus (platform) ins Depot. Jetzt ist es so 1:1. Früher war es wirklich nur das, was der Kunde da abgelegt hat oder der Nutzer. Jetzt haben wir gesagt, wir schieben auch Daten aus (platform) adrein. Das heißt, dass was wir in (platform) zusätzlich haben, was gar nicht im Depot (-) ist in (platform) wesentlich vollständiger, wesentlich umfrangreicher, aber im Depot zumindest mal in rudimentärer Form auch vorhanden. Also das ist schon ein Qualitätsunterschied. Das ist eigentlich die Qualität, für die wir Geld haben wollen, was da der Unterschied ist.	

Interview 23			Noch zu meinem Verständnis: Das Depot wird ja aber auch vom (institute) dann gemanaget, oder nicht?
Interview 23		Ne, das ist das, was ich vorhing sagte, wir gehen wieder mehr mit (platform) zusammen, also mit den (town)r. Das machen wir seit, ich glaube, seit Mitte letzten Jahres, ist es veröffentlicht. Also das Depot immer für die Anorganik, ganz am Anfang wie gesagt auch für die Organik. Und wir haben jetzt seit Mitte letztens Jahres sind die Depots zusammen gelegt, aber das ist auf der Plattform von (town), weil die einfach die technisch ausgereifter - wir hatten keine echte. Also wir haben die Daten abgelegt und sie konnten bei uns nachfragen und dann haben sie eben einen Datensatz bekommen. Die haben einfach ein automatisiertes System mit Prüfung und allem pipapo. Man muss das ja nicht doppelt entwicklen. Und dann habe nwir uns da beteiligt und (-) Ziel ist es tatsächlich, die zwei Datenbanken auf lange Sicht - ich will jetzzt nicht sagen zu vereinigen, das ist zu viel gesagt -aber eine gemeisname Suchoberfläche zu schaffen.	
Interview 23		Auch über (platform) und (platform) , sodass man da genau diese Schnittmengenkunden zwischen Anorganik und Organik besser bedienen kann, weil wir jetzt sehen, dass die Nutzer - gerade beim Depot auch - enorm positives Feedback gegeben haben und dass sie das ganz toll finden und genau ihren Bedürfnissen entspricht.	
Interview 23	48:40:00	Diese Trennung zwischen Anorganik und Organik, die man früher mal gemacht hat, was ich vorhin sagte, Handschlag - man sah sich da dann auch glaube ich als reine wissenscahft und weniger als kommerziellen Service. Ich meine, wir müssen alle unser Geld verdienen, damit wir das Zeug machen können. Aber letzendlich ist die (platform) auch ein non-for-profit institut, insofern passt es auch gut. Und wir wollen die Community bedienen, das ist das, was wir wollen.	
Interview 23	49:30:00	Und möglichst gut und von daher wollen wir weiter zusammen arbeiten und enger zusammen arbeiten.	
Interview 23			Die stehen auf jeden Fall auch auf meiner Interviewliste
Interview 23		Ja?	
Interview 23			Ja
Interview 23		Der CEO, ich weiß nicht, ob sie die Gelegenheit haben mit ihm zu sprechen, (name), der ist auch ein deutscher. Mit dem könnten Sie sogar deutsch sprechen. Ganz netter Mann! Sind sowieso alle nett, ganz nette Kollegen ja, Falls sie Probleme haben, da in Kontakt zu kommen, sagen Sie mir ruhig Bescheid. Dann kann ich ihnen da auch helfen.	
Interview 23	50:00:00		Ich werde mal gucken, wie es da weiter voran läuft. Zurück zu ihrer Frage, dementsprechend sind das kommerzielle oder auch nicht-kommerzielle Plattformen.
Interview 23		Ja. Ok, und, na gut, ja das frage ich jetzt nicht. (lachen)	
Interview 23			Gut.

Interview 23		Ne, aber nochmal, mich würde wirklich das Ergebniss interessieren, weil das ist natürlich was, was uns tagtäglich beschäftigt. Gibt es dafür Kriterien? Oder sind die Kriterein, die wir anlegen falsch, oder vielleicht kann man das besser machen? Man kann immer alles besser machen. Und von daher würde mich das schon interessieren.	
Interview 24	Time	Interviewee 24	Interviewer
Interview 24			
Interview 24	02:45		Haben Sie sonst noch irgendwas, was Sie im Vorhinein ansprechen wollen?
Interview 24		Erstmal nicht. Sie haben ja sozusagen auch angekündigt, dass, wenn Sie dann fertig sind mit Ihrer Evaluierung, dass man die / auch die Umfrageteilnehmer dran teilhaben lassen an den Ergebnissen. Das ist natürlich eine ganz spannende Geschichte auch für uns, die wir natürlich auch (unv.) im Hinterkopf haben, aber wir uns damit natürlich ein bisschen schwer tun, diese tatsächlich zu messen - es gibt ein paar Messwerte, das ist gar keine Frage. Aber dadurch, dass es ein communitygetriebenes Projekt ist, hängt man natürlich dann auch immer von der Community ab. (unv.). Aber gut, da können wir später nochmal drüber reden. Ok, ja, alles fein.	
Interview 24	03:53		Also Sie können das auch gleich erzählen, wenn Ihnen das gerade auf der Zunge liegt, ist ja auch gut.
Interview 24		Naja, ne es geht halt immer darum, wie man die Wissenschaftler dazu animiert an so einem Repository aktiv teilzunehmen - sprich, dass sie Daten einladen in das Repository. Wobei man sich natürlich immer überlegen muss, was ist die Karotte, die man den Wissenschaftlern hinhalten kann oder was ist der Benefit für die Leute daran teilzunehmen, weil gerade so eine datengetriebene Wissenschaft (unv.), da geht es halt um Publikationen, das ist das wichtigste. Mit den (unv.) kann man sich die (unv.) verdienen, aber mit Datenablage im Augenblick noch nicht.	
Interview 24		Und das ist dann halt ein Zusatzaufwand, der betrieben werden muss und der Nutzen aus diesem Zusatzaufwand, der erschließt sich vielen auf den ersten Blick nicht. Und das ist das Problem - eigentlich - der meisten Repositories, genau die (Situation?), es gibt kein Geld dafür, es gibt keine zusätzlichen Indexcoints dafür, es gibt kein gar nichts dafür, außer die Gewissheit, dass man dran teilgenommen hat, ein Repositori und damit eine vernünftige Datenbasis - hoffentlich - gemeinsam aufzubauen.	
Interview 24		Aber das ist alles sehr schwach als Argument, weil letztlich zählt dann immer das Argument: was bringt mir das - das kostet mich Zeit und in der Zeit könnte ich vielleicht etwas beseres machen.	
Interview 24	05:18		Genau, um dieses Problem kümmere ich mich, heute werde ich das auch noch ansprechen.
Interview 24		Ok, habe ich mir schon gedacht. Ok.	

Interview 24			Gut, dann fange ich gleich mal an mit der / also ich habe mich ja jetzt schon kurz vorgestellt. Ich würde auch gerne etwas über Sie erfahren. Was ist so Ihr Hintergrund und was sind so Ihre Tätigkeiten?
Interview 24		(personal)	
Interview 24	07:25		Nun haben Sie ja schon (platform) angesprochen. Wie ist denn die Geschichte dahinter?
Interview 24		Die Geschichte ist eingentlich ganz witzig, weil - ich bin selber Biologe - und als ich meine Doktorarbeit gemacht habe, habe ich auch selber Enzyme (unv.) oder Membranenzyme charakterisiert und bin dabei schon auf die Schwierigkeit gestoßen, dass ich meine Laborergebnisse nicht mit denen vergleichen kann, die publiziert sind, weil einfach die Publikationsdaten uneinheitlich sind, man kann die Daten nicht nachvollziehen, weil im Methodenteil vielleicht Informationen fehlen, weil die Ergebnisse sehr stark prozessiert sind,	
Interview 24		das heißt, man findet Zahlen, aber kann diese Zahlen nicht auf irgendwas zurück beziehen, auf Material, Methoden, Berechnung oder sonst irgendwas und dann stand ich dann erstmal da, was nicht weiter schlimm war für meine Doktorarbeit, aber es hat mich umgetrieben - als ich dann nachher beim (institute)-Institut war habe ich dann eine Konferenz mit organisiert, das ging um Chemie-Informatik und ich habe da halt festgestellt, dass die Chemiker genau das gleiche Problem haben.	
Interview 24		In der Chemie-Inforamtik wird Software produziert oder wird Software entwickelt und diese Software, die analysiert Daten - mit dieser Datenanalyse kann man neue Wirkstoffe, Wirkstoffziele und dergleichen bestimmen. Das Problem ist halt, diese Tools funktionieren nur dann gut, wenn die Daten gut sind und da haben wir halt auch wieder ein Problem.	
Interview 24		Die Datenqualität auch in der Chemie ist (unv.). Und das führte mich dann wieder auf mein eigenes Problem mit zurück und ich hatte dann die Gelegenheit, hier das Projekt aufzuziehen. Erstmal einen Workshop zu Enzymdatenstandardisierung zu iniziieren, da kamen dann plötzlich Wissenschaftler weltweit zusammen und aus diesem Workshop purzelte dann sozusagen mehr oder weniger eine Arbeitsgruppe heraus mit Mitgliedern, die das Problem auch so gesehen haben und das ist dann sozusagen die (platform) Komission geworden.	
Interview 24		(platform) steht ja für "anonymized" und eigentlich sind wir damit gestartet die Experimente, die experimentellen Bedingungen zu standardisieren und wir haben dann aber sehr schnell herausgefunden, das ist sehr schwer. Und dann haben wir gedacht ok - dann fangen wir von hinten an, dann standardisieren wir zumindest das Reporting der Daten. Dass man halt so eine vollständige Darstellung halt aller experimentellen Bedingungen und experimentellen Ergebnisse in Paper.	

Interview 24		Und das sind dann die (platform) Guidelines geworden, das hat ein paar Jahre gedauert, weil das ist ein langer Diskussionsprozess innerhalb der Komission und der Komission mit der Community.	
Interview 24	08:59	Wir haben dann im zwei Jahres Abstand immer Tagungen gehabt und haben da auch diese Guidelines vorgestellt und disskutiert und auch natürlich richtig wissenschaftliche Beiträge rund um die Enzymologie gehabt und so entstanden dann die (platform) Guidelines / ich glaube (year/number) oder so standen die dann soweit fest und das waren dann die ersten Zeitschriften mit an Bord, die diese Standards empfohlen haben und wir haben dann aber festgestellt, dass es sehr schön ist, dass diese Standards empfohlen werden, aber kein Mensch hat sich dafür interessiert.	
Interview 24		Weder die empfehlenen Zeitschriften, noch tatsächlich die Autoren oder Gutachter oder die Herausgeber der Zeitschriften. Das hat ein paar Gründe und deswegen haben wir gesagt: ok, dann machen wir doch Folgendes, dann gießen wir diese (platform) Guidelines sozusagen in ein Software-Tool, (platform).	
Interview 24	10:52	Dieses Tool, das überprüft die eingegebenen Daten, Manuskriptdaten, auf ihre Einstellung, Konformität mit den (platform) Guidelines und wenn die Daten einmal eingegeben sind und der Check - das ist kein Qualitätscheck, sondern Komformitätscheck - wenn der erfolgreich ist, dann schmeißen wir die Daten nicht weg, sondern speichern sie in der Datenbank.	
Interview 24		Und dann kriegen die Autoren, die diesen Test / oder deren Manuskriptdaten diesen Test erfolgreich bestanden haben, bekommen dann sozusagen -ja - eine (platform)-Registry Number und die Idee war, dass dann die Autoren die (platform)-Registry Number an ihre Zeitschrift weitergeben und die Zeitschrift freut sich darüber, weil sie sagen: ok, diese Daten sind vollständig im Sinne von (platform) und jetzt brauchen unsere Gutachter jetzt nur noch auf die wissenschaftliche Qualität zu gucken.	
Interview 24	11:41	Das ist nach wie vor noch die Idee. Und wir sind jetzt auch dabei, dass (unv.), dass auch die ersten Zeitschriften (platform) empfehlen und unser nächster Schritt ist, dass aus dieser Empfehlung tatsächlich ein verpflichtender Status wird, so wie es für eine Reihe von anderen Datenbanken oder biologischer Daten auch der Fall ist.	
Interview 24		Das ist so in Kurzform die Geschichte von (platform). Also das (platform)-Projekt ist jetzt - im Herbst (year/number) aufgesetzt - das wird jetzt bald (number/year) Jahre alt.	
Interview 24	12:47		Gerade sagen Sie, dass es diverse Gründe gibt, warum sich Journals und so weiter nicht dafür interessiert haben - welche sind denn das?

Interview 24		Also nicht interessiert ist vielleicht falsch ausgedrückt. Sie können es einfach nicht leisten. Weil, wenn man ein Paper hat mit Proteindaten oder Proteomdaten, wo dann auch ein Protein / wurde dann aufgereiningt, isoliert, wurde charakterisiert und wurde dann auch funktional untersucht, das ist ja die Enzymologie und für jeden dieser Schritte gibt es eigene Reporting-Guidelines, dann hätte so ein Zeitschriftenherausgeber 4 oder 5 Reportingguidelines nebeneinander legen müssen, um dann die Manuskriptdaten auf Guideline-Konformität zu überprüfen.
Interview 24		Und das ist einfach zu viel. Angesichts der Manuskriptsmasse, die Herausgeber bearbeiten müssen, ist das ein Arbeitsaufwand, den die Zeitschriften nicht leisten können und nicht leisten wollen. Und deswegen / das ist so der Hauptgrund eigentlich. Es ist nicht so, dass sich keiner dafür wirklich interessiert.
Interview 24		Alle Zeitschriften möchten ja eine hohe Qualität haben, aber diesem Anspruch sind Grenzen gesetzt durch Manpower Ressourcen und auch die Massen der hereinkommenden Manuskripte.
Interview 24		Was man natürlich intern machen könnte ist, dass man die Gutachter dann darauf ansetzt herauszufinden, ob diese Manuskripte guideline-konform sind, aber es gibt eigentlich keine echten Richtlinien für die Begutachtung von Zeitschriftenartikeln. Das macht dann jeder frei nach Schnauze und die Gutachten können sehr gut sein für einen Artikel, aber es gibt auch Gutachten, die sind lausig, die sind / da (schreibt?) der Begutachter: ich habe eh keine Lust und winkt das Manuskript dann durch, da wird keinem geholfen.
Interview 24	14:07	Da wird so ein bisschen auch dieses Gutachtungswesen zum Teil unterlaufen / gut, das sind andere Gründe. Aber im wesentlichen ist so, es ist einfach ein Ressourcenmangel bei den Zeitschriften und sie können das tatsächlich nicht leisten, deswegen brauchen sie auch die Freiwilligkeit, dass die Autoren selber Interesse daran haben, guideline-konform auch ihre Manuskripte zu verfassen und es gibt Autoren, die machen das, aber die größte Masse macht das eigentlich, die größte Masse der Autoren kommt in den Instructions for Authors eigentlich gar nicht so weit, so zu lesen, dass es tatsächlich dann Reporting-Guidelines gibt, sondern die interessieren sich im wesentlichen dafür, wie muss mein Manuskript eigentlich formatiert werden, dass es diesen (reformer check?) erstmal übersteht beim Einreichungsprozess.
Interview 24		Und deswegen war das auch unser Ansatzpunkt zu sagen, ok, wir stellen (platform) als Tool der Community zur Verfügung. Die Journals haben etwas davon, weil die können dann sagen, ok, jedes Manuskript, das mit einer (platform)-Registry Number kommt oder mit einer DOI, das ist (platform) Guideline konform.

Interview 24	15:36	Für die Autoren ist so das Ding / ok, sie müssen natürlich dann den Extraschritt gehen, dass sie sagen, ok, ich muss meine Manuskriptdaten in dieses Repository eingeben um dann nachher am Ende sicher zu gehen, dass meine Daten vollstädnig sind. Und für den Gutachter ist es eigentlich auch sehr schön, weil die wissen, hier kommt was auf den Tisch, was vollständig beschrieben ist und ich brauche mich nur noch um die Qualität zu kümmern.	
Interview 24	16:22		Das heißt, was ist jetzt das Kernangebot der Plattform?
Interview 24		Das Kernangebot sind zwei Kernangebote. Das eine ist die Validierung auf (platform) Guidelines und am Ende ist es der Bau einer Enzymdatendatenbank, was es auch bisher noch nicht gibt. Und zwar haben wir hier die experimentellen Bedingungen und die experimentellen Ergebnisse nebeneinander aufgeführt.	
Interview 24		Für Systemologen ist das sehr wichtig, wenn es hier / es geht um Aktivitätsdaten von Enzymen - ich weiß nicht, wie tief Sie in dem Thema drin sind - geht darum, zu messen, wie schnell setzt ein Enzym ein Substrat um, wie kann es gehemmt werden, wie schnell lässt sich das hemmen, wie hoch ist die Spezifität des Enyms an das Substrat, gibt es noch andere Substrate und dergleichen.	
Interview 24		Also Kennziffern, mit denen man die - sagen wir mal - die Effizienz von einem Enzym messen kann und Systembiologen, die nehmen gerne diese Kennziffern um komplette Stoffwechselwege zu modellieren. Funktioniert nur dann gut, wenn die Systembiologen auch wissen, unter welchen Bedingungen diese Kennziffern ermittelt worden sind, weil die sind variabel in Abhängigkeit der experimentellen Bedingungen.	
Interview 24	17:41	Und, wenn ich jetzt beides bereitstelle - die experimentellen Bedinungen und diese Kennziffern, dann habe ich als Nachnutzer dieser Daten gewonnen. Es gibt andere Datenbanken, (platform) beispielsweise und auch (unv.) das sind auch zwei Enzymdatenbanken und da finden Sie im wesentlichen nur die Kennziffern, ohne die experimentellen Daten und ohne die experimentellen Bedingungen und dann müssten Sie immer in die Originalliteratur hereingehen, um zu gucken, was hier eigentlich gemacht wurde. Und das macht (platform) /diese Schritte deckt (platform) ab.	
Interview 24	18:13	Also, was wir haben ist zwei Dinge in einem: Validierungstool und das andere ist langfristig gesehen ein sehr gutes Kompedium für Enzymkinetikdaten.	
Interview 24			Nun handelt meine Arbeit ja von Erfolgsfaktoren. Wie definieren Sie denn Erfolg, beziehungsweise eine erfolgreiche Plattform?
Interview 24		Im Wesentlichen zwei Dinge. Das eine ist halt die Durchdringung in die Community, das heißt, dass Zeitschriften diese Plattform hrer Kundschaft, ihren Autoren empfehlen und am besten dann auch verpflichtend zur Benutzung ausschreiben.	
Interview 24		Und die zweite ist eigentlich sehr prophan, durch das Zählen der Datensätze, die pro Zeitabschnitt in die Datenbank reinkommen.	

Interview 24		Und im Augenblick haben wir noch nicht so viele Daten. Der dritte Punkt wäre dann die Nutzung dieser Daten, das heißt Abrufen von Daten und Zitieren von Daten, das wäre dann auch nochmal ein Erfolgsfaktor.	
Interview 24			Welche Vision wird denn mit (platform) verfolgt?
Interview 24	19:23	Wir wollen - die Vision ist ganz klar - die Anhebung der Qualität von Ezymdaten in der Literatur und in Datenbanken.	
Interview 24			Kommen wir jetzt zu meinem ersten großen Block über den organisatorischen Aufbau hinter der Plattform und wie das aufgebaut ist. Ja, meine erste Frage ist sofort, wie ist der organisatorische Aufbau denn hinter der Plattform?
Interview 24		Technisch oder personell?	
Interview 24			Beides.
Interview 24		Also personell arbeiten wir auf sehr sparsamer Flamme, ich bin derjenige, der hier in der Stiftung zuständig ist für das komplette Projekt. Ich werde unterstützt durch die (platform) Kommission, die besteht aus, zur Zeit - lasse mich nicht lügen - 15 Wissenschaftlern, die sozusagen als, ja, in ihrer Freizeit ihren Beitrag für (platform) leisten - alles mögliche, was so anliegt.	
Interview 24		Die werden also nicht dafür bezahlt, dass sie was machen, sondern sie machen das aus eigener Überzeugung und wir treffen uns ein mal im Jahr zum (platform)-Meeting und besprechen da die wesentlichen Dinge und legen auch die Aufgaben für das kommende Jahr dann fest. Wir haben / das ist im Prinzip unsere organisatorische Form, wir haben natürlich ein großes Netzwerk in die / das ist einfach eine Akademie, also dazu zähle ich mich auch, dass das alles über Freiwilligkeit geht, dass man hofft, dass das irgendwann alles über Eigendynamik stattfindet,	
Interview 24		so funktionieren eigentlich alle diese Community angetriebenen Projekte, dass ein Projekt, das wirklich sinnvoll in der Community erscheint, dass das dann auch eine Eigendynamik erfährt und dass das dann auch freiwillig verwendet wird von den Leuten in den relevanten Systemen.	
Interview 24		Das ist mit (platform) nicht anders und wir sind im Augenblick sehr klein, aber was die Standards / als Standardinitiative sind wir recht erfolgreich, weil wir halt auch den Vorteil haben, mit dem (institute)-Institut im Rücken haben wir eine langfristige Perspektive, wir müssen nicht von Funding / von Drittmittelförderung zur Drittmittelförderung uns hangeln und alle zwei Jahre einen neuen Antrag schreiben oder so, solange die Stiftung sieht, dass dieses Projekt Fortschritte macht, wird dieses Projekt unterstützt.	
Interview 24	22:08	Technisch haben wir halt / das System ist von einem Kollegen von mir hier im Institut technisch umgesetzt worden, ich habe die Spezifikation geschrieben, er hat das technisch umgesetzt. Das System ist hier im Haus auf einem Server und das war es dann eigentlich.	

Interview 24			Sie haben jetzt gerade schon die Finanzierung angesprochen, deswegen möchte ich da noch weiter nachhaken. Erfolgt denn die hunderprozentige Finanzierung dann über die Stiftung?
Interview 24		Ja.	
Interview 24			Ok. Das ist ja eine kurze Antwort (lacht).
Interview 24		Ja, es ist so. Mehr brauche ich / ja.	
Interview 24			Ja. Wie / Zurück zu Ihnen und auch zu denen, die freiwillig mitarbeiten. Welche Kompetenzen und welche Tätigkeiten haben Sie dann?
Interview 24		Also die (platform) Kommission besteht aus Experten aus den diversen Ecken der Enymologie. Das sind Biochemiker, das sind Bioinformatiker, das sind Leute, die haben ausgewiesene Datenbankexpertise, das sind Leute, die haben Expertise in der industriellen Enzymologie, wir haben Systembiologen an Bord - habe ich jemanden vergessen?!	
Interview 24	23:01	Ne, das ist eigentlich so das Wesentliche. Also wir haben die Leute, also die Kommission besteht aus Expertise aus allen Bereichen der Enzymologie dabei und wenn wir auf ein Problem stoßen, wo wir vielleicht externe Expertise oder darüber hinausgehende Expertise brauchen sollten, ist unser Netzwerk groß, dass wir mal gerade jemanden fragen können und wenn es etwas größeres ist, dann können wir den auch zum (platform) Meeting einladen und dann gibt der uns so ein bisschen Hilfestellung, das ist auch / das ist alles sehr unkompliziert und auch unbürokratisch.	
Interview 24		Wir haben jetzt bisher noch nie aus der Community gehört, ne, euch helfe ich nicht, weil ihr blöd seid, sondern, ich kann euch nicht helfen, weil ich keine Zeit habe wenn überhaupt oder / aber zu 90 Prozent habe ich immer gehört, oh, das ist ein tolles Projekt, ja, da, wenn ihr Rat und Tat braucht, helfe ich euch gerne. Also das ist ein echtes Community driven project.	
Interview 24	24:32:00		Und wie sind dann die unterschiedlichen Verantwortlichkeiten für die Plattform verteilt?
Interview 24		Die liegen alle bei mir.	
Interview 24			Ok, das heißt, Sie treffen dann auch die ganzen Entscheidungen? Oder wie sind da so die Entscheidungswege?
Interview 24		Ich habe den Hut auf bei der ganzen Geschichte, weil als Repräsentant von (institute), das der Geldgeber ist, haben wir natürlich auch die letzte Entscheidung, aber wir haben natürlich die (platform) Kommission, in der bestimmte Dinge - oder in der alle Dinge disskutiert werden, es werden hier Richtungsentscheidungen getroffen und diese Richtungsentscheidungen / selbst wenn kein hundertprozentiger Konsens besteht, es gibt immer Bedenken bei bestimmten Dingen, aber wenn es eine Mehrheitsentscheidung gibt, dann wird diese Mehrheitsentscheidung umgesetzt und dafür stehe ich.	
Interview 24	25:24:00		Nun haben Sie ja schon die Richtung angesprochen und auch das Verhältnis zu (platform) und (unv.), wie positionieren Sie sich denn auf dem Datenmarkt?

Interview 24		Als / nicht als Konkurrenten. Das spiegelt sich schon darin wider, dass wir in der Kommission einen Repräsentanten von (unv.) und (platform) jeweils dabei haben und wir verstehen uns eigentlich als Dreieck im Enzym - wenn man so will - Enzymologie Datenmarkt. Weil jeder hat etwas anzubieten und wir sind nicht substetutiv unterwegs.	
Interview 24		Wir ergänzen - komplementär - wir ergänzen uns gegenseitig. Und das zeigt sich auch schon daran, dass wir auch zusammen / zumindest mal mit (unv.) zusammen auch ein Datenaustauschformat / an einem Datenaustauschformat arbeiten um dann ohne Probleme Daten von einer Datenbank zur anderen hin und her transferieren zu können.	
Interview 24		Das finde ich eigentlich eine sehr angenehme Situation, weil wir alle am gleichen Strang ziehen und wir befinden uns nicht im Wettbewerb, weil jeder eine eigene Nische belegt, aber - sagen wir mal - mit relativ ähnlichen Daten (unv.) - ja.	
Interview 24			Welche konkreten Ziele werden denn jetzt verfolgt?
Interview 24		Im Bezug auf (platform)?	
Interview 24			Ja.
Interview 24		Also ganz konkret sind wir gerade dabei, an einem (unv.) Datenaustauschformat arbeiten wir gerade. Das ist eine ganz wichtige Geschichte. Auch hinsichtlich unserer Bestrebung noch offener zu werden. Und ich bin / wir sind gerade dabei auch die Datenbank (platform) zu erweitern, um Felder, die benötigt werden.	
Interview 24	27:05:00	Wir haben gerade die Version 1, wir haben gesagt / immer gesat, Version 1 ist der Start, aber Version 1 ist nicht da Ende, das heißt, wir / es ist war immer klar, dass diese Datenbank erweitert werden muss. Und das sind so die nächsten Ziele, auch die Erweiterung.	
Interview 24		Und natürlich, das so das (unv.), Promotion in der Community, (platform), (platform) noch bekannter zu machen, die Journals noch stärker an uns zu binden und zu überzeugen und zu machen, das ist halt so die Daueraufgabe, die gemacht werden muss und das machen wir auch.	
Interview 24		Junge Wissenschaftler müssen vertraut gemacht werden mit dem System, dass es Standards gibt, dass sie sich da bitte gefälligst dran zu halten haben, dass es dafür ein Tool gibt, nämlich (platform), das ihnen da hilft und die junge Wissenschaftlergeneration ist da deutlich offener, als die alten Knochen und das ist eine sehr gute Erfahrung, die ich in den letzten Jahren gemacht habe.	
Interview 24			Nun haben Sie mir ja von einigen Zielen erzählt, wie kontrollieren Sie denn die gesteckten Ziele?

Interview 24		Ganz normal, mit Projektmanagementmitteln. Das wird als Projekt behandelt, es werden / die Ziele sind natürlich relativ weit, es ist nicht so, dass man sagt, bis zum Jahresende will ich jetzt 10 Zeitschriften mit an Bord haben, natürlich ist das ein Ziel, das ist aber ein Idealziel, weil man das nicht erzwingen kann, weil das sind unheimlich viele Dinge, die man nicht selber in der Hand hat, man ist von anderen Leuten abhängig, aber kann zumindest Ziele formulieren und versuchen, die zu verfolgen.	
Interview 24		Wenn man die Ziele jetzt verfehlt, wenn man jetzt statt 10 Zeitschriften meinetwegen nur 5 hat, ist das auch nicht dramatisch, das ist aber der Fortschritt, den das Projekt macht. Da sind wir relativ - ja, ich will nicht sagen frei - aber es gibt da ein gewisses Maß an Flexibilität.	
Interview 24		Und das wird natürlich nachgehalten, also auch beim (platform) Meeting, da werden viele Ziele und Verantwortlichkeiten festgelegt, also ich habe mich nicht für alles, was bei einem (platform) Meeting beschlossen wird, verantwortlich gemacht, sondern ich habe die Verantwortlichkeiten verteilt und auch die Ansprechpartner dann verteilt und die Verantwortlichen für kleine Teilprojekte, die können sich natürlich noch Hilfe aus der (platform) Kommossion noch dazu suchen, aber die sind letzlich dann erstmal verantwortlich, die Geschichte - ihr kleines Teilrpojekt voranzutreiben.	
Interview 24	30:00:00	Weil ich kann das nicht alles alleine machen, ich habe noch andere Jobs außer (platform) und das funktioniert eigentlich ganz gut. Und dann trägt man dann die Ergebnisse beim nächsten (platform) Meeting zusammen und schaut, was dabei herausgekommen ist.	
Interview 24			Welche Schwierigkeiten und Herausforderungen gibt es denn beim Management von (platform)?
Interview 24		Ressourcenknappheit, ganz klar. Also ich könnte locker noch mehr Leute gebrauchen, die diesen Outspread machen, die trommeln, die Marketing machen, die mit der Community viel mehr agiert und solche Geschichtchen, das ist so die Hauptherausforderung bei der ganzen Geschichte.	
Interview 24			Gut, super, wenn Sie da nichts mehr weiter zu sagen haben oder anzumerken haben, dann würde ich gerne mit dem nächsten Block fortfahren. Mit dem Bereich Plattform, Governence und Nutzung. So, das, ich hoffe, das wird sich jetzt nicht doppeln, weil manchmal ist es schwierig abzuschätzen, was schon alles so erzählt wurde in den Interviews. Ich fange mal mit der ersten Frage an, ich weiß, Sie haben das schon ein bisschen erläutert, vielleicht kommt gleich noch mehr. Welche Funktionalitäten und Möglichkeiten bietet (platform)?

Interview 24		Das ist ja, wie gesagt, das habe ich ja eben gesagt, dass die Validierung und die Datenvalidierung von Manuskriptdaten auf (platform)-Konformität und dann die Ablage dieser Daten in einer strukturierten Datenbank und natürlich dann nachher auch die Abfragemöglichkeit dieser Daten aus dieser Datenbank heraus, als Open-Access Datenbank.	
Interview 24	31:34:00	Funktionalitäten haben wir jede Menge in Bezug auf die Unterstützung, also User-Support, Usability der Datenbank, da ist eine ganze Menge implementiert, wobei wir hier noch nicht das Ende der Fahnenstange erreicht haben, aber ich weiß nicht, ob Sie da jetzt wirklich drauf abzielen.	
Interview 24		Ich glaube eines der wichtigsten Dinge, ist - ich weiß nicht, ob das in dem Zusammenhang passt - aber, was wir machen, ist, dass jeder Datensatz wird bei Data Side registriert, bekommt eine DOI und diese DOI ist auch dafür geeignet, für die Autoren, ihren eigenen Datensatz zu zitieren, so ähnlich wie bei (platform), bei Protein Database, da man die Identifiers verwenden kann, um dann direkt auf Strukturen in der Protein Database zu referenzieren	
Interview 24		und mit der DOI machen wir das genau so und wir haben im Augenblick jetzt schon Exportformate, das sind XML, also dieser Datensatz, der kann heruntergeladen werden im XML Format und auch woander hingeladen werden und der Datensatz kann auch komplett als PDF ebenfalls heruntergeladen werden, ausgedruckt werden	
Interview 24		und hier ist die besondere Charme, oder die Idee, was wir auch offensiv unterstützen, dass die Autoren dieses PDF in dem das Fact Sheet, in dem da alles drinsteht, als Datensatz drinsteht, dass dieses Fact Sheet zusammen mit einem Manuskript eingereicht wird beim Journal und dieses Fact Sheet kann dann tatsächlich auch von unseren Gutachtern verwendet werden, um die Qualität des Manuskripts zu begutachten ohne eigentlich tatsächlich das Manuskript lesen zu müssen.	
Interview 24		Einen Augenblick. Ich kriege hier gerade einen Anruf auf der anderen Leitung, darf ich Sie mal zur Seite legen?	
Interview 24	34:00:00		Ja.
Interview 24		Einen Moment. (Telefonat auf der anderen Leitung).	
Interview 24	35:24:00	Sorry, es war wichtig.	
Interview 24			Alles gut, kein Problem.
Interview 24		Jetzt bin ich wieder voll und ganz für Sie da.	
Interview 24			Sehr schön. Dann fahre ich gleich mal mit der nächsten Frage fort. Wie beschreiben Sie die Nutzergruppe von (platform)?
Interview 24		Es gibt / wir haben gelernt - lernen müssen - dass es im Grunde zwei Nutzergruppen gibt. Es gibt eine Gruppe, die gibt Daten ein und es gibt die Gruppe, die benutzt die Daten für weitere Forschung. Und diese beiden Gruppen sind eigentlich nicht deckungsgleich.	
Interview 24		Also, diejenigen, die Daten generieren, sagen wir mal der Enzymologie, der Biochemiker, der Bench, der Aktivitätstest von Enzymen macht, der diese Daten erhebt und eingibt, der nutzt die nicht nach und holt sich die Daten aus (platform) wieder heraus - in den seltensten Fällen.	

Interview 24		Diejenigen, die diese Daten nachnutzen, sind im Prinzip tatsächlich Systembiologen, die Interesse haben, an Kinetiken, das ist so die Hauptgruppe, natürlich auch die Leute, die an der Bench stehen wollen vielleicht auch mal gucken / wollen vielleicht Vergleichsdaten haben, aber das ist jetzt nicht die Hauptzielgruppe. Wir haben im Prinzip zwei - grob zwei verschiedene Gruppen, was im Prinzip auch nicht ungewöhnlich ist bei der Verwendung von Datenbanken, dass diejenige Gruppe, die Datein eingibt, eine andere ist, als diejenige, die Daten nutzt. Ich hoffe, das beantwortet relativ verwirrungsfrei Ihre Frage.	
Interview 24			Ja, sehr gut. Noch eine Nachfrage dazu. Welche fachlichen Disziplinen nutzt dann (platform) überhaupt?
Interview 24	37:12:00	Biochemiker.	
Interview 24			Ok.
Interview 24		Im weitesten Sinne. Also, wie gesagt, das können Enzymologen sein, das können Applied (unv.), also Leute, die Enzyme im Anwendungsbereich modifizieren oder untersuchen oder was auch immer, das können Systembiologen sein, aber das ist eigentlich alles / sind alles Subspezies im Bereich der Biochemie.	
Interview 24			Nun haben Sie ja gesagt, dass die Uploader andere Menschen sind als die Downloader. Welche Schwierigkeiten können denn genau bei diesem Datenaustausch über die Plattform auftreten?
Interview 24		Keine Ahnung, eigentlich / dadurch, dass wir beide Gruppen ja in der (platform) Kommission haben, hatten wir eigentlich dahingehend noch keine Schwierigkeit gehabt. Wichtig ist natürlich, dass man eine einheitliche Terminologie verwendet, die beide Nutzergruppen versthen und auch selber anwenden, das tun wir. Und von daher ist mir eigentlich von Schwierigkeiten zwischen diesen beiden Gruppen, was den Datenaustausch angeht, nichts bekannt.	
Interview 24			Nun ist das Schlagwort Nachnutzbarkeit auch schon häufiger gefallen. Wie schätzen Sie denn die Nachnutzbarkeit der Daten ein?
Interview 24	38:37:00	Hoch, absolut hoch und sie wird umso höher, je detaillierter oder je mehr Daten wir tatsächlich auch in (platform) / also je größer / je umfangreicher der Datensatz ist und je mehr Datensätze wir in (platform) haben.	
Interview 24			Wie ist es denn, wenn - sag ich mal - Forscherinnen und Forscher die Plattform nutzen wollen? Welche Regeln unterliegen sie?
Interview 24		Was meinen Sie jetzt mit Regeln? Lizenzen, oder rechtlichen Regeln oder technischen Regeln?	
Interview 24			Beides.
Interview 24		(Lacht). Jetzt machen Sie es sich aber leicht. Technischen Regeln: sie müssen einen Internetzugang haben, weil es eine Online-Datenbank ist, die / dem Eingabeformular ist es relativ Wurst, mit welchem Browser Sie ankommen, das ist / funktioniert mit allen Browsern sehr gut.	

Interview 24		Also technische Regeln haben wir absolut / ja, Sie müssen halt / ja, technische Regel ist auch, dass Sie natürlich alle verpflichtenden Daten eingeben müssen, ansonsten werden Sie von (platform) angemeckert, beziehungsweise drauf hingewiesen, dass Daten fehlen, Datenformate vielleicht falsch sind, das sind so formatvalidierende Geschichten, darauf beschränkt sich dann eigentlich schon das Technische.	
Interview 24		Und das rechtliche ist so, dass wir (-) wir haben auf der Webseite haben wir den Terms and Conditions (Serve?) veröffentlicht und wir / natürlich benutzt die Eingabemaske, wenn man sich registireren muss / benutzt die dann auch Cookies, aber wir sind so sparsam, wie es eigentlich nur geht, im Sinne der DSGVO, das sehen wir schon sehr streng.	
Interview 24	40:13:00	Und die Datensätze, die eingegeben sind, die sind in einer CC Buy Lizenz, also die sind jederzeit nachnutzbar von anderen und wäre schön, wenn man dann natürlich dann differenziert, auf (platform), entweder mit dem DOI oder zumindest später dann auf die (Cookie?) Originalpublikation. Aber das ist so im wesentlichen alles, was mir gerade so einfällt an Regeln.	
Interview 24			Wie stellen Sie denn sicher, dass Personen ausreichender Zahl und Qualität die Daten auf die Plattform hochladen?
Interview 24		In ausreichender Anzahl, was ist ausreichend?! Also so viel wie möglich ist gut. Ich / das ist im Augenblick noch ein kleines / oder ist tatsächlich ein Problem, weil noch nicht so viele Leute Daten eingeben, weil das auch ein Bekanntheitsproblem ist von (platform), da kommen wir dann auf die Ressourcenknappheit wieder zurück. Die Quatlität / es gibt zwei Qualitätsstufen, das ist ist / das ist das, was wir / was ich hier mache, ist die Plausbilitätskontrolle, also es wird in (platform) / wird nichts veröffentlicht, was nicht publiziert ist, ganz wichtig.	
Interview 24		Also die können Daten einladen in die Datenbank, aber diese Daten sind nicht sichtbar, solange ich sie nicht freigeschaltet habe. Und diese Daten werden erst dann freigeschaltet, wenn diese Daten im Originalmanuskript auch in einem Journal veröffentlicht worden sind. Und die Veröffentlichung im Journal bedeutet, dass dieses Manuskript durch einen (Perreview?) Prozess durchgegangen ist, was im besten Sinne hoffentlich eine vernünftige Qualitätskontrolle durchlaufen hat.	
Interview 24		Das ist das eine. Das andere ist, ich mache hier selber eine Plausibilitätskontrolle, das heißt, ich besorge mir die Publikationen und schaue nach, ob die Angaben, die in der Publikation übereinstimmen mit den Angaben in der Datenbank. Und wenn das alles gut ist, dann wird der Datensatz veröffentlicht.	
Interview 24			Und wie wird dann das Vertrauen in die Daten sichergestellt?

Interview 24		Das Vertrauen in diese Daten ist / kann nicht höher oder schlechter sein, als das Vertrauen in die Daten in der Publikation. Das, was wir nicht machen, ist Data (unv.), dass wir (nachexerpieren?), warum auch, weil der Autor hat ja die Daten eingegeben, hat die Manuskriptdaten eingegeben und wenn diese Manuskriptdaten durch den (Perreview?) Prozess durchgegangen ist, dann muss das / dann ist das in Ordnung.	
Interview 24		Data / ein (Datenkurator?) kann auch nicht viel mehr machen, als zu schauen, ob diese Daten - ja / der wird die Experimente nicht nachkochen können, das wäre der sicherste Weg, um zu schauen, ob die Daten richtig sind, er wird auch nicht mehr machen können, als Plausibilitätskontrollen und schauen, wie valide diese Daten sind, das ist das, was wir auch machen.	
Interview 24	42:58:00	Aber nicht als Kurator, Kuratoren, die exerpieren ja die Literatur und beziehen dann die Daten aus den publizierten Artikeln und was wir machen, ist, wir lassen die Autoren sozusagen für uns arbeiten und behandeln so die Daten schon.	
Interview 24			Wenn jetzt Nutzer die Daten runterladen, wie stellen Sie sicher, dass sie die Daten wissenschaftlich korrekt verwenden?
Interview 24		Das kann ich nicht sicherstellen. Was heißt wissenschaftlich korrekt, also mit den Daten des statistischen Bundesamtes können sie auch Schindluder, das kann man nicht kontrollieren, das kann man nicht verhindern und das fällt dann letztlich auf den Autor selber zurück, der diese Daten nutzt oder missbraucht, um irgendwelche schrägen Dinge daraus zu machen, das ist nicht im Verantwortungsbereich der Datenquelle.	
Interview 24	44:55:00		Um nochmal auf den Anfang von dem ganzen Interview zurück zu kommen, da hatten Sie auch schon erwähnt, wie motiviere ich die Forscher, dass sie Daten hochladen?! Meine Frage jetzt zurück an Sie erstmal, wie bewegen Sie denn Forscher, die Plattform zu nutzen?
Interview 24		Im Gespräch. Gespräche laufen immer über Datenqualität, sobald man merkt, dass die Forscher unzufrieden sind über die Datenqualität oder über die Verfügbarkeit von Daten, das ist schon ein prima Einstiegspunkt für mich. Darauf hinzuweisen, dass wir mit dem Projekt an dem Thema Datenqualität arbeiten und wir schwimmen mit (platform) und dem anderen Projekt (name) wunderbar oder (unv.) in dem Open Science (Fahrwasser?).	
Interview 24		Open Science ist auch eine der größen Stichworte, die wir in der Stiftung verfolgen und Open Science bedeutet tasächlich Forschungsdaten, Forschungsergebnisse offen zu machen und transparent zu gestalten. Und das ist eine ganz wichtige Geschichte, Transparenz in die Forschungsdatengenerierung reinzubringen. Da kann jeder Forscher für sich selber herausfinden, welche Qualität bestimmte Daten haben oder welche Qualität Datenquellen haben und können dann entsprechend darauf reagieren.	

Interview 24		Im Weiteren ist es so, dass wir auch hier sehr früh schon im (Fahrwasser?) sind, nämlich Datensätze mit einer DOI Ziffer sehen, Datensätze registrieren, weil ich davon ausgehe, dass früher oder später nicht nur Publikationen als wissenschaftlicher Beitrag gezählt werden und auch die Zitierung dazu, sondern auch die Publikation von Datensätzen und auch die Zitierung von Datensätzen eine Bedeutung hat	
Interview 24	46:23:00	und was wir auch machen, ist, mit dieser DOI, das ist eigentlich so das Goldstück, oder das (unv.), ist mit der DOI werden die Datensätze auffindbar. Für Forscher ist es eigentlich wichtig / das wichtigste für Forscher ist es gelesen zu werden und zitiert zu werden und wenn wir den Forschern helfen, dass ihre Datensätze gefunden werden - gesucht und gefunden werden - weil, Indexierungstools weltweit sich auf die DOIs stürzen, dann ist das eine wunderbare Sache und das ist so die Karotte, die wir hinhalten.	
Interview 24		Das ist auch ein Generationending, es sind eher die jüngeren Forscher, die wissen, was eine DOI ist und wie wichtig die DOI ist, um das in die (unv.) beispielsweise eingepflegt zu haben und die Älteren, die wissen eigentlich nicht viel darüber.	
Interview 24		Das ist für mich so die verlorene Generation, bei (einer Situation?) wie heute, die, was weiß ich, die seit 30 Jahren im Geschäft sind, die fast Mitte, Ende 50 sind, die wenigsten interessieren sich tasächlich für sowas und das ist für mich auch die verlorene Generation. Mit denen muss man eigentlich darüber (nicht?) sprechen und deswegen habe ich auch sehr viel, sehr positive Resonanz von Nachwuchsgruppenforschern, von Jungforschern und dergleichen, von Leuten, die gerade am Beginn ihrer akademischen Karriere sind.	
Interview 24	47:26:00	Weil die sind nämlich auch diejenigen - das ist eine ganz interessante Geschichte - die sind altruistisch unterwegs. Die sagen, das habe ich selber in einer Umfrage herausgekriegt, wenn ich Daten eingebe in (platform), dann trage ich dazu bei, dass andere meine Daten finden und wenn andere auch ihre Daten eingeben, dann kann ich dann auch Daten in (platform) finden und gerade diese Findbarkeit von Forschungsdaten ist für mich eine ganz wichtige Geschichte. Und das fande ich einen starken Ansatz.	
Interview 24	48:45:00		Welche weiteren Möglichkeiten sehen Sie denn noch, Nutzer zu generieren?
Interview 24		Nutzer generieren hört sich gut an, die fallen nicht vom Baum, die können wir auch nicht schnitzen, ich kann es einfach nur bekannt machen, dass es das System gibt, ich kann es dadurch machen, dass die Journale, die Zeitschriften ihre Autoren darauf aufmerksam machen,	
Interview 24		das passiert auch dadurch, dass (platform) in einer Reihe von Recommended Repositories Listen erscheint, wenn Sie auf (platform) / die Projektseite von (platform) gehen, finden Sie auch eine Liste von Zeitschriften, die (platform) empfehlen, das ist die ganze (journal?), also wirklich die großen Zeitschriften sind mit dabei.	

Interview 24		Das ist auch so ein Thema, Bekanntmachung und dann die Registrierung in Repositories wie (unv.) ist eine wichtige Geschichte bei Open Air registriert zu sein, das sind jetzt alles so - sagen wir mal - im Hintergrund organisatorische Dinge, die im Augenblick noch nicht so wahnsinnig ins Gewicht fallen, aber sobald dieser Open Science Zugriff richtig losgelegt hat, ist das sehr hilfreich.	
Interview 24		(platform) ist auch eine der ersten Projekte, die in (Fair Sharing?) registriert worden sind, das hieß damals noch (Medi?) und daraus ist dann (BioSharing?) geworden, das ist jetzt auch schon 10 Jahre her. Was auch hilft, wenn man eine (Fair Sharing?) ID hat, denn solche Projekte, die haben dann auch / kriegen dann auch entsprechende Anerkennug durch die Journale, die Journale fragen tatsächlich danach, haben Sie eine (Fair Sharing?) ID? - ja, habe ich - ja, super, dann hat man schon halb gewonnnen.	
Interview 24	49:56:00	Das sind viele kleine Minischritte und Prozesse, die im Hintergrund laufen und die dann so Stückchen für Stückchen das Projekt voranbringen und auch in die Community reinbringen. Das ist kein großer Schlag, das ist nur Trippelschritte, aber wie ein (unv.) praktisch irgendwo - ich würde nicht sagen, unterwandert - aber, ja, Bestandteil wird einer Community.	
Interview 24	51:05:00		Nun ist die Besonderheit von (platform) ja auch, dass sie in die wissenschaftliche Gemeinschaft eingebettet ist. Wie schätzen Sie den Einfluss der wissenschaftlichen Kultur auf die Nutzung der Plattform ein?
Interview 24		Also erstmal ist es so, dass die meisten Projekte dieser Art ist von der wissenschaftlichen Community selbst getragen. Der Bottom-Up Ansatz, es ist das wichtigste, ohne diesen Bottom-Up Ansatz funktioniert das nicht. Also, wenn zum Beispiel das (unv.) sagt, wir machen jetzt eine Datenbank und da sollen jetzt alle dran teilnehmen, das macht kein Mensch. Jetzt habe ich den zweiten Teil Ihrer Frage vergessen.	
Interview 24			Ne, wie die wissenschaftliche Kultur die Nutzung der Plattform beeinflusst.
Interview 24		Das ist ja ein Kulturwandel erstmal. Jetzt geht es darum, dass man bereit ist, seine Daten zu teilen, Daten zu nutzen und Nachnutzung und dass man aus seinem kleinen Kämmerlein herauskommt. Und in den Biochemiewissenschaften haben wir es eigentlich relativ leicht, weil, es hier / die meisten Projekte können nur in Kollaboration funktionieren, weil die Projekte / weil die Expertise zur Bearbeitung von Projekten ist nicht in einer einzelnen Hand.	

Interview 24	51:52:00	Das heißt, die Biochemiecommunity ist im wesentlichen sowieso schon gewöhnt, Daten zu teilen. Und (platform) ist eigentlich eine Plattform, um Daten zu teilen, wobei man hier die Einschränkung machen muss, wir / im Augenblick sammeln wir noch keine Rohdaten, sondern prozessierte Daten, also berechnete Daten, und / aber mit diesen berechneten Daten habe ich ja gesagt, wir haben zwei Gruppen von Nutzern, diejenigen, die Daten eingeben und die anderen, die Daten benutzen und die sozusagen einen Forschungskreis dann unter Umständen schließen und - ja - die wissenschaftliche Kultur ist einfach wissensgenerierend und datenteilend.	
Interview 24		Und da gibt es sehr viele. Es gibt Communities, die sind da ganz anders drauf, in der Chemie beispielsweise in der organischen Chemie ist es so, das Datenteilen wird da sehr skeptisch gesehen.	
Interview 24			So, nun komme ich zu den letzten drei Fragen.
Interview 24		Ok.	
Interview 24			Welche Entwicklung sehen Sie für die Plattform in den nächsten zehn Jahren?
Interview 24		Sagen wir mal so - mein Kaffee ist leer, ich kann es Ihnen nicht sagen - aber das Ziel ist, dass (platform) die gleiche Bedeutung kriegt wie (platform). Bei (platform) ist es so, dass sie ihre Protein- oder Kristallstrukturen abgelegt haben müssen in der Datenbank, bevor sie überhaupt ihren Artikel publizieren können.	
Interview 24		Die brauchen eine (platform) Identifier, um damit publizieren zu dürfen. Und das / oder auch (unv.) oder (unv.) Daten ist es ähnlich. Und das Ziel verfolge ich für (platform) auch. Die müssen eine (platform) DOI oder (platform) Registry number haben und dann können sie den Artikel erst veröffentlichen. Das hoffe ich, dass das schneller geht, als in 10 Jahren.	
Interview 24	54:14:00		Und wovon hängt der Erfolg der Plattform dann ab?
Interview 24		Von der Nutzung. Von der Nutzung sowohl was die Datenangabe eingeht, als auch von der Nutzung, von der Nachnutzung des (platform) / dass ich Anfragen habe in (platform), dass Daten genutzt werden und idealerweise dann auch Daten zitiert werden, wenn sie zitiert werden.	
Interview 24		Und das wird sich dann, da wir noch keine Metriken dafür haben, ist das noch ein bisschen Zukunftsmusik, aber wenn wir über 10 Jahre sprechen, gehe ich davon aus, dass (platform) DOIs ein wichtiger Beitrag sind, um Datensätze zu zitieren.	
Interview 24			Ja, wunderbar. Damit sind jetzt auch schon 25 Fragen um. Jetzt kommt meine letzte. Haben Sie sonst noch etwas, was Sie mir zu Erfolgsfaktoren mitgeben wollen? Zu Herausforderungen oder was Ihnen da noch einfällt?
Interview 24		Also, im Augenblick nicht, aber wahrscheinlich wenn wir aufgelegt haben, fällt mir bestimmt noch etwas ein, aber ich muss im Augenblick echt passen, also ich glaube, ich habe Ihnen soweit alles verraten, was mir jetzt so direkt einfällt. Ne, wüsste ich jetzt nichts.	
Interview 24	55:48:00		

Interview 25	Time	Interviewee 25	Interviewer
Interview 25	02:58		Bevor ich mit den Fragen starte, würde ich dich gerne nochmal ein bisschen kennelernen. Was ist so dein Hintegrund und was gehört so zu deinen Tätigkeiten?
Interview 25		(personal)	
Interview 25	03:58	Und habe damals zwei Bewerbungen noch stehen lassen und die eine davon war an der Vorläufer Organisation des (Fitzs?) und da hat man mich damals eingestellt als stellvertretenden Gruppenleiter. Die Gruppe gab es damals schon und (platform) gab es damals auch schon.	
Interview 25	04:51	Aber die die Gründerin der Gruppe war todkrank und ich bin da als Krankheitsvertretung reingekommen. Ich fand damals interessant in retrieval - also in retrieval Forschung - ist es häufig schwierig, wenn man etwas innovatives macht schwierig, an Testpersonen zu kommen. Und ich fand damals super-interessant etwas zu machen, was mit echten Nutzern zu tun hat und das finde ich auch immer noch interessant und darum bin ich auch immer noch hier, als inzwischen als Gruppenleiter. Meine Vorgängerin, die (name) ist leider verstorben (year/number), wir haben noch schön zusammen gearbeitet und ich habe dann progressiv, also (year/number) habe ich die Gruppe übernommen.	
Interview 25	05:39	Die Gruppe ist halbe halbe oder sagen (unv.) ein Großteil der Leute sind Biochemiker, Chemiker, Biologen - also mehr mehr aus der Bio-Ecke und quasi die andere Hälfte der Gruppe sind Entwickler, die ja - die Software schreiben. Also wir haben zwei große Richtungen in die das ganze geht. Das eine ist wir sind im (???) Projekt. Das ist ein Projekt, in dem wir versuchen Leute dazu zu kriegen, ihre ihre Daten selber aufzubereiten und selber zu sharen. Also es geht so in die Richtung, ja es ist ein zwischending zwischen einem Electronic Lab Journal und so etwas wie wie Open (Air/R??).	
Interview 25	06:38	Ja, also wo wo Projekte, gemeinsam Daten sammeln können und auch auch per DOI publizieren können.	
Interview 25			Nun hast du ja gerade gesagt, dass die Hälfte dann so Biochemiker, Biologen usw. sind und die andere Hälfte Entwickler. Wie viele sind denn dann insgesamt mitbeteiligt?
Interview 25	06:59	Also jetzt (unv.) - (15s) (Tastaturgeklapper) - also momentan fünf Entwickler - (17s) (Tastaturgeklapper) - eine Wissenschaftlerin, die so ein bisschen eher informatischen Background hat, und vier Leute, die Dienstleistungen machen im Bio-Bereich oder also die halt Biofachwissen haben, also fünf zu vier (currently?)/	
Interview 25	07:52		Danke, dann wollte ich auch nicht weiter unterbrechen.
Interview 25		Nee, ich habe gerade jemanden an der Tür stehen. - (Unterhaltung mit der Person an der Tür)- Ja okay, man kann bei mir ins Büro reingucken, ich hab da so ein Fensterchen und da war offensichtlich meine Gestik nicht ganz klar.	

Interview 25	08:21	So, - ja -/ Genau (unv.) ist ja der (unv.)/(name)beide promovierte / nein/ (name) ist glaube ich Biologin und Ulrike ist Biochemikerin. Die haben Hiwis, die Daten extrahieren aus Papers und sie schauen dann nochmal nach und bringen die Sachen noch Mal besser in Form für das ABAK. Und das ist natürlich eine komplett andere Vorgehensweise, als jetzt bei sowas wie dem (Sigs?)-System, was das (unv.) Projekt macht.	
Interview 25	09:12		Nun hast du ja erzählt, dass du als, als du dazugestoßen bist, dass damals schon (platform) gab. Wie ist denn die Geschichte hinter der Plattform?
Interview 25		Also -- ich glaube der älteste Code in (platform) ist aus den neunziger Jahren. Die (name) ist Datenbänkerin gewesen, war interessiert an der Aufbereitung von Pfadwegen, eine sehr wichtige Kollegin für sie war die Ursula Kummer, die jetzt Professorin an der Uni in Heidelberg ist (unv.).	
Interview 25	10:00	(platform) ist entwickelt worden als ein System, was Pfadwege speichern kann und zwar auch mit kinetischen Informationen, die jemand wie Ursula Kummer braucht. Wir machen ja quantitative mechanistische Modelle von von Pfadwegen und für die brauchen wir Parameter - und für und für diese Community ist die ganze Sache gestrickt. Und es wird jetzt aber auch von Leuten benutzt, die eigentlich ihren Überblick haben wollen, was sind denn zum Beispiel - typische - typische kinetische Eigenschaften von Enyzmen oder wie weit kann (unv.) welchen Einfluss hat pH oder so, also sozusagen, die sich die Daten an sich anschauen. Aber sozusagen unser Haupt use case waren eigentlich Leute die Modelle dann hinterher bauen wollen.	
Interview 25	11:13		Und was ist/
Interview 25		(unv.)	
Interview 25			Entschuldigung
Interview 25		Ich sage unser, aber sozusagen, aber das war vor meiner Zeit/	
Interview 25			Und was ist jetzt das Kernangebot der Platform?
Interview 25		da muss ich sagen immer noch/ -- Wir - Wir haben sehr viel Zeit reingesteckt, um die Nutzung zu vereinfachen. Wir sind jetzt gerade dabei / wir haben zwei zwei Erweiterungsprojekte die BMBF gefördert sind, die jetzt gerade am Laufen sind, die in zwei komplett unterschiedliche Richtungen gehen. Das eine ist (platform) Bis?, da geht es darum die komplexen Daten, die (platform) hat, möglichst sinnreich zu visualisieren und es so zu ermöglichen, dass die Leute mehr aus den Daten machen.	

Interview 25	12:10	Also im Moment ist es ja so, wir haben an verschiedenen Stellen bieten wir ja auch taxonomische Suche, also das man zum Beispiel sagt, okay ja, meine Nagerart kann ich jetzt gerade nicht finden, aber ich nehme irgendein Säugetier - in dem es gemessen worden ist zum Beispiel. Derartige Suchen kann man bei uns machen. Das man also in diesem taxonomischen Baum so ein bisschen höher geht, als man anfangs war. Aber --- da (platform) gibt momentan nur beschränkt einen Überblick über über zum Beispiel die Werte, die kinetische Parameter haben. Das heißt man bekommt eine Liste von Tabellen/ Hast du dir (platform) angeguckt? Hast du die möglich/	
Interview 25			Ja
Interview 25	13:04	Okay du hast ja diese Einträge, diese Eintröge sind in (unv.) Tabellen, wir haben auch an einer Stelle schon --- ich - gehe jetzt mal einfach kurz in die Suche -- (Tastaturgeklapper) ich such jetzt hier (unv.) nach Glukose, wir haben da dieses Virtual Search Tab, wenn du/	
Interview 25			Den dritten da ja/
Interview 25		Genau so ja - und in die Richtung gehen wir dann halt jetzt weiter, also da geht es ja eher, welche welche Werte gibt es überhaupt und wie viele davon, ja/ Was für Gewebe, was für Organismen und in die Richtung gehen wir halt weiter, dass wir versuchen eigentlich Überblicke zu geben. Weil diese Überblick hat zwei --- Funktionen, das eine wenn man ein Modell bauen möchte, um vielleicht und nicht ganz genau das findet, was man gesucht hat, möchte man eigentlich wissen, was sind denn die Extremwerte, die ich erwarten könnte.	
Interview 25	14:15	Sagen wir das mal so, ich hätte das gern für - für (unv.) und Körpertemperatur, aber das gibt es jetzt gerade nicht, dann möchte man natürlich schon wissen - zwischen zwischen welchen Möglichkeiten bewegt sich der Wert für die Kinetik. Also, das ist einerseits, für sozusagen, unsere Kernnutzer ist das gut und zur anderen Seite gibt uns das natürlich auch die Möglichkeit, das ein bisschen zu beurteilen, was halten wir denn von unseren eigenen Werten.	
Interview 25	14:55	Wir haben es durchaus schon gehabt, dass (unv.) Eine lachende Kuratorin im Büro stehen, die -- die nach einem Wert gefragt hatte und also/ und irgenwie hatte sie, sie konnte eine Kommastelle nicht lesen und in einem Paper. Und am Ende hat sich herausgestellt, dass es statt milli mikro sein musste, ja. Also sie hat durch reinen Zufall um einen Faktro tausend erfahren, der falsch war. Weil sie mit dem Autor vom Paper Kontakt aufgenommen hatte. Und solche Dinge würde man natürlich gerne, sehen können - und für solche Sachen ist natürlich wenn man eine Überblicks Visualisierung von den Werten machen kann sehr nützlich.	
Interview 25			Die Überblicksvisualisierung ist ja jetzt ein Projekt, was du angesprochen hast, was ist das Zweite dann?

Interview 25	15:58	Das Zweite da geht es darum, näher dranzukommen, oder mehr Sachen ---- allgemeiner ausgerückt, die Kuratorin besser automatisch zu unterstützen. -- Und die - und da ist halt das Problem, dass bei den Methoden, die wir kennen -- die Zahl der false Positives so groß ist, dass man dann eigentlich die Sachen nochmal selber lesen muss, ja. Und es gibt ja Systeme, die machen (unv. recognition?) oder - oder versuchen auch einzelne Werte zu extrahieren. Wenn die aber zu oft falsch liegen, dann kann man halt gerade auch dieses Problem haben, was auch die Piloten haben. Das/	
Interview 25	17:06	Dadurch dass die Piloten zu viel mit Autopilot fliegen, sie manche Fertigkeiten verlieren. Und bei uns es dann halt so, wenn man wenn man jetzt versucht Dinge automatisch zu extrahieren und man extrahiert sie falsch, dann übersieht man halt eventuell mehr Sachen und spart auch gar nicht so viel Zeit, als wenn man das ganze selber durchsieht. Und da haben wir jetzt ein Projekt, wo wir wo wir mit mit (name) dann zusammen ein System entwickeln wollen, was halt einserseits Vorschläge macht und auf der anderen Seite sich so verhält, dass man nicht in falscher Sicherheit gewiegt wird.	
Interview 25			Gerade hast du schon die Kernnutzer von (platform) angesprochen, wie beschreibst du denn die Nutzergruppe?
Interview 25	18:02	(7 s) - Ja, -- also die die meisten Leute, die ich treffe, sind schon aus dem Modellierungsbereich / --- Und das sind halt sehr - sehr computeraffine Leute --- die dann halt auch mit so Dingen wie Webservices was anfangen können. Also das ist ein großer Teil und was ich aber auch teilweise getroffen habe, sind Leute die die ---- in einem gewissen Gebiet kinetische Daten messen und ein Gefühl dafür entwickeln wollen, ob ihre Daten okay sind.	
Interview 25	18:57	Also das eine, ist ja eine Sache zu messen und sich sicher zu sein, ich mach das entsprechend dem State of the Art und habe sauber gearbeitet und dann möchte man ja aber eigentlich auch wissen, ist das jetzt, was ich gemessen habe, überraschend oder nicht. Und die schauen halt auch ab und zu in unser System rein.	
Interview 25			Nun handelt meine Arbeit ja von Erfolg bzw. Erfolgsfaktoren, wie definierst du denn Erfolg oder eine erfolgreiche Plattform?
Interview 25		(9 s) - Sind glaube ich zwei / es gibt zwei Arten von Erfolg, also das eine ist -- Nutzerzaheln -	

Interview 25	20:00	das andere ist -(5 s)- ja so dieses Kritikerlob, ja. Also ich denke es ist ein bisschen wie beim Theater, sie können volles Haus haben oder du kannst ein volles Haus haben / --- aber dann gibt es noch noch einen anderen Erfolg, nämlich das irgendwie/ einen seltenen Moment, die Hauptkritiker von der (Zeitschrift) sich alle einig sind, dass es ein tolles Stück war. Und und so ein bisschen ist es bei uns auch. Also, das eine ist, ist das wir natürlich versuchen Nutzer anzuziehen und das andere ist das Lob, das wir bekommen von den Nutzern. Also, dass gesagt wird, wir merken die Daten haben eine hohe Qualität, halt eben, weil sich die Leute selber anschauen (unv.) ist in dem Gebiet, in dem sie tätig sind, halt gibt es da viele Outlier, gibt es da viele Überaschung oder	
Interview 25	21:11	oder ist man, macht die Datenkollektion wie ist an sich Sinn, und dann gibt es natürlich. Die die ganzen technischen Dinge, kann ich die Resultate schnell finden, kann ich meine Suche einfach spezifizieren - kann ich die Daten so exportieren, dass ich hinterher mit denen etwas machen kann und so weiter und so fort. Und ja, also das sind die zwei Dimensionen, die ich (unv.).	
Interview 25			Welche Versio/ Welche Vision verfolgst du denn mit (platform) auf lange Sicht?
Interview 25		(Unv.) Ich bin immer so ein (unv.) -(4 s)-	
Interview 25	22:07	Langfristig - also es gibt wirklich, denke ich, eine Sache (unv.) (platform). Das ist, dass man pro Paper, was man analysiert, wir ungefähr einen Arbeitstag reinstecken und das würde ich gerne so drücken können, dass wir große Zahlen von Daten bekommen. Also unsere Kollektion sehr stark erweitern können und zwar ohne die Kompromisse (unv.) zur Datenqualität aufzugeben. Das ist ein großes Thema, weil hat eben dann dafür eigentlich (unv.) Unterstützung sehr viel besser werden muss.	
Interview 25	23:00	Wir arbeiten auch langfristig eigentlich schon - nicht sehr öffetlich, aber aber immer wieder in Richtung Kooperationen mit anderen Datenquellen. Also, unsere Daten sind sehr sehr reich strukturiert, es gibt manche, die haben mehr Daten aber weniger strukturiert und das man das man da zusammen arbeiten möchte, um diese strukturierten Daten aus, sagen wir mal, vorstrukturierten Daten zu erstellen. Also das ist ist, sagen wir mal, die die techinsche Seite eins, sozusagen, also die die Kuratierung so weiter treiben, dass wir unsere Datenmenge nicht um 10 Prozent steigern, sondern um Faktor 10.	

Interview 25	23:58	Die andere Richtung ist Suche, also ich denke, da gibt es auch ziemlich viel Musik, und wir sind gerade dabei uns diese Musik zu überlegen. Weil die Daten, es ist ja eigentlich nicht nicht trivial ja, man hat diese diese Pfadwege und man kann natürlich versuchen, so wie (platform) alles alles auf die Pfadwege zu (merken?). Aber ich denke, es gibt einige Eigenschaften, da ist gar nicht so klar, wie möchte man die eigentlich visualisieren, dass die Leute, die diese Daten suchen, etwas davon haben. Und daran möchten wir mehr arbeiten. Dann ist (unv.) natürlich immer schauen wir uns an wollen wir, wollen wir weiter gehen in, in Richtung zum Beispiel Reaktionmechanismen klarer auch zu schlüsseln.	
Interview 25	25:10	In die Richtung -(5 s)- zum Beispiel Pharmakokinetik da mit aufzunehmen. Und Ja, wäre eine Frage ist, glaube ich, momentan nicht ganz so das Thema.	
Interview 25			Dann komme ich mal zu einem ganz anderen Thema. Du hast ja schon angesprochen, du bist ja jetzt der Gruppenleiter von den Mitarbeitern. Wie ist denn der organisatorische Aufbau hinter (platform)?
Interview 25	25:50	Also die Kuratoren haben momentan eine eine Doppelrolle. Sie sind (unv.) Also einerseits halten sie den Kontakt zu unseren Nutzern - dann --- machen sie zu großen Teil die Requirements. Also durch den Kontakt mit den Nutzern bekommen wir Ideen für die Requirements, also was wollen wir ändern, an welchen Stellschrauben wollen wir drehen. Zum Beispiel diese Visualierungssache ist durchaus gefragt, die ist nicht im Detail gefragt ja. Aber wenn man dann mit fünf Leuten gesprochen hat, die sagen die Daten sind sehr toll, aber irgendwie würden wir gern mehr in diesen Daten intuitiv graben können. Dann überlegt man sich halt, wie man so etwas herstellen kann. Also die Kuratoren machen natürlich die Kuratierung und da ist es so	
Interview 25	27:06	Das wir hier, ein ein Pool von Hiwis haben, und (platform) hat neben dem was man sieht, hat es auch ein Kuratierungsinterface und diese Hiwis haben dann Zugang zu diesem Kurtierungsinterface und bekommen Aufgaben zugeteilt und bekommen Papers zugeteilt, die sie dann bearbeiten. Bearbeiten heißt, dass die Daten extrahiert werden aus dem Paper. Und dann gehen die Kuratoren nochmal drüber. Es gibt verschiedene Dinge, wo es Ermessensspielräume gibt, die man etwas abbildet innerhalb von (platform). Diese Ermessensspielräume werden geglättet, wenn man so will, dadurch das halt die Kuratoren, das halt seid Jahren machen und wissen wie sie das bei anderen derartigen Quellen gelöst haben und dann die Verlinkungen zu Onthologien typischerweise von den Kuratoren gemacht.	
Interview 25	28:13	Und dann wird halt ungefähr alle zwei Monate werden die Daten von dem von dem Kuratierungsinterface transferriert ins (platform) und dann hat man wieder eine neue Variante.	

Interview 25			Du sprichst ja gerade schon die Quallitätskontrolle der Daten an, wie wie genau stellt ihr die Qualität dann sicher? Also du hattest es ja schon ein bisschen angesprochen aber gibt es da noch weitere Faktoren?
Interview 25		Also was wir teilweise gemacht haben, ist das wir das wir zufällig Einträge ausgewählt haben, die dann zu prüfen waren. Das man also nach einer Zeit noch mal drüber geht mit einem frischen Auge, und dann schaut, ob die Sachen richtig sind.	
Interview 25	29:08	Da hatten wir sehr kleine Fehlerraten, im Moment machen wir das nicht. Aber, dass erinnert mich an etwas, ja/ Also und das haben wir halt voll zugänglich gemacht. Also einfach die Zahl der (platform) Eiinträge genommen und dann (unv.) und diese Einträge wurden dann halt (abgedruckt ?). Wir bekommen teilweise Rückmeldung, also wenn jemand einen Fehler findet, weitere Qualitätsprüfung machen wir nicht.	
Interview 25	30:00		Wie ist es denn überhaupt mit dem Data-Input? Also auf der Website steht ja auch, dass wird ja kuratiert über die Literatur und auch Forscher und Forscherinnen können ja auch automatisch etwas submission. Wie ist denn da so das Verhältnis zwischen den Einträgen?
Interview 25		Also wir, wir haben vielleicht zwei-dreistellig Entrys in (platform), die die von Wissenschaftlern gemacht worden sind, also die direkt gekommen sind/	
Interview 25			Wie viele waren das?
Interview 25		Das Gros ist wirklich, ich würden denken, dass es eine einstellige Prozentzahl ist.	
Interview 25			Okay
Interview 25		Ich kann das jetzt schnell live nicht nachschauen. Aber es ist wirklich wirklich wenig.	
Interview 25			Ne, so relevant ist die Zahl auch nicht, nur um da so ein Gefühl für zu bekommen. Nicht so ganz wichtig/
Interview 25		Also die Möglichkeit ist da, wir haben sie geschaffen, was wir gemacht haben ist, dass wir ein system gebaut haben das die Daten ins Kuratierungsinterface (reinlässt?). Das heißt es ist so, als ob da jemand/ als ob ein fixer Hiwi ganz viele Daten eingegeben hätte, ja. Und dann wird es genauso behandelt, wie von der Litertatur eingegeben, auch deswegen, weil ja ein Teil ist - den richtigen Onthologieterm zuzuordnen und (wir?) nicht erwarten können, dass die Wissenschaftler das für uns machen.	
Interview 25	31:32	(3 s)- Also die experimentiellen Wissenschaftler, die das gemessen haben. Bist du noch da?	
Interview 25			Ja, ich bin da. Ich höre dir gespannt zu und mache mir immer Notizen nebenbei.
Interview 25		Okay, ja genau, es ist schwer zu aber/	
Interview 25			Dann zurück wieder zur Plattform, wenn jetzt irgendwie Entscheidungen getroffen werden sollen, wie findet das statt? Also diskutiert ihr das, macht ihr E-Mails irgenwie mit (Konsens-Arbeiten?) ?

Interview 25	32:10	Wir sitzen alle an einem Ort. Wir/ natürlich haben die Entwickler auch etwas zu sagen. Ja, wir sind eine Weile Srum gefahren. Jetzt fahren wir haben wir momentan keinen Scrum, weil wir jetzt über Jahre hatten wir eine (unv.). Und es fühlt sich dann sehr komisch an, wenn da (unv.) seinen Srum macht. Oder sagen wir mal, man muss es (lieben?). Ich bin jemand, der so etwas nicht gerne macht (unv.) Haken am abend geile Sache, ja. und/ und der Entwickler mag das nicht so und muss er auch nicht/	
Interview 25	33:01	Und wir sprechen dann halt/ wir sind ja nicht komplett im luftleeren Raum. Sondern wir haben ja auch auch typischerweise Projekte, wo man dann sagt dieses und jenes, sollst du vielleicht gemacht haben, damit die beim nächsten Projektreview gut aussehen. Solche Entscheidungen kommen natürlich rein und dann überlegen wir uns, wie wir das am am besten umsetzten. Ja, also typischerweise Gruppendiskussion. Und da sind wir uns dann häufg einig ja (lacht)	
Interview 25			(lacht) Vorhin hast du ja auch schon angesprochen, der Vergleich mit zum Beispiel (platform). Wie soll oder soll denn wie positionierst du denn (platform) strategisch, jetzt auch gegenüber anderen?
Interview 25	34:04	Ja ich denke, dass das große Ding ist ist ((platform)?). ((platform)?) ist nicht so einfach, weil die einfach alles machen. Und es ist nicht leicht, uns gut zu positionieren ohne etwas Negatives über ((platform)?) zu sagen, ja.	
Interview 25			(mh, zustimmend)
Interview 25		Censiert auf Bitte. Verfügbar auf Nachfrage	
Interview 25	38:11	Ja, das ist glaube ich so, dass strategischte, was ich sagen kann. Also globaler gesagt, Innovation ich denke die Visulaierung, die wir bauen gerade, die kann ich natürlich gerade nicht zeigen (lacht), dass wird sehr schön. Das heißt, also weiter die Schiene laufen, dass man sagt, man möchte, dass wenn die Leute (platform) benutzen sie so viel (Mehrwert?) aus einer Session ziehen wie geht.	
Interview 25			Da komme ich mal zu einer anderen Frage. Wie erfolgt denn jetzt die Finanzierung von (platform)?
Interview 25	39:00	Also momentan ist (platform) hauptsächlich finanziert über über (institute) und über über dieses Visualisierungsprojekt. Wir haben über über weite Teile der Lebenszeit von (platform) haben wir - typischerweise - Eigenkurator intern finanziert. Ich habe zum Beispiel auch gar nicht so wenig (platform) entwickelt, in meiner Anfangszeit, das war alles interne Sache. Das heißt also momentan sind wir hauptsächlich extern finanziert, sind aber über/ also da ist sehr viel internes Geld auch drin.	
Interview 25			Ist sie/
Interview 25		(unv.) wie jetzt Basisförderung	
Interview 25	38:58		Ist die Finanzierung dann langfristig? - Oder eher kurzfristig?

Interview 25		(unv.) wenn wir das wüssten. Also (institute) arbeitet auf seine (Bestätigung?) hin, gewonnen ist noch nichts und verloren ist auch noch nichts. Es gibt sehr konkrete Bestätigungspläne, wir sind teil einiger dieser Bestätigungspläne und hoffen, dass wir, wenn es dann wirklich bestätigt wird, auch wirlich dabei sind. Aber es ist nichts, was man/ ich glaube nicht, dass es was (unv.) in der (work) lesen möchte (lacht)	
Interview 25			(lacht) Nein, also keine Sorge ich bringe das auch auf eine abstrakte Ebene und schreibe da auch nie was direkt über eine Plattform, also keine Sorge, da gehe ich vertraulich mit um.
Interview 25		Ja also, der Punkt ist halt wirklich diese Bestätigungskiste ist halt seitdem ich diesen Job mache immer ein Problem. Und es tun sich da Möglichkeiten auf und da sind wir jetzt mal alle gespannt. Also momentan bin ich sehr stolz, dass ich/ wir hatten vor zwei Jahren einen Überlegung mit externen Kollegen, die zu Besuch kamen.	
Interview 25	41:23	Und die waren erstaunt, wie wenig permanente Stellen ich hier habe in der Gruppe und und das heißt, wir wir schaffen es, den Eindruck von Kontinuität zu erwecken, dass wir ganz schnell einen Flickenteppich weben. Der der so dann aber dann doch so sehr Teppich ist, das man den Eindruck hat, da müsste was da sein, was das ganze zusammenhält. Aber momentan haben wir halt wie gesagt die Hoffnung, dass das es ja wirklich eine Bestätigung da ist, womit man dann den Leuten längerfristige Verträge geben kann.	
Interview 25	42:13		Welche weiteren Schwierigkeiten und Herausforderungen gibt es denn beim Management von (platform)?
Interview 25		(5 s) Also das ist mal schwierig mal weniger schwierig. Also - also zwei zwei komplett unterschiedliche Richtungen. Das eine ist, es ist eine sehr kleine Gruppe, die sehr lange sehr eng zusammenarbeitet und das heißt die Leute sind praktisch miteinander verheiratet. Das kann manchmal Probleme (town), wenn man wenn man irgendwo Doktorand ist, dann sind die ältesten Problem, die man mit sich rumschleppt, sind drei Jahre alt. Und jetzt die Leute machen das seit zwölf teilweise schon fünfzehn Jahren zusammen, ja.	
Interview 25	43:17	So, dass kann manchmal Probleme mit sich bringen, die man sonst nicht so hat in einem rein wissenschaftlichen Kontext. Ich denke, im Großen und ganzen kriegen wir da supergut hin. Ich merke das auch immer wenn wir Besuch haben, dass wir eigentlich/ (unv.) als Einheit rüberkommen. Aber ja aber ja, ich würde sagen, das ist (wenn man so eine Ressource baut) sicherlich was, was man im Auge behalten muss. Also die Langfristigkeit von dem ganzen. Ich hätte mir den zweiten Gedanken aufschreiben müssen/ (4 s).	
Interview 25			Soll ich warten?

Interview 25	44:18	Nein, er kommt mir gerade. Der andere ist, dass ist je nach Arbeitsmarkt ist es sehr unteschiedlich. Also meine erste Hiring Runde habe ich gemacht als als als gerade die (year/number)er Krise durch war. Da habe ich, in einer Bewerbungsrunde habe ich, lauter Top Leute bekommen. Und ohne mich jetzt groß anzustrengen und jetzt, zehn Jahre später ist eigentlich jede jede Bewerbung ein (unv.)-Spiel. Einfach deswegen, weil die Märkte so leer sind, dass die Leute, die nicht schon über den kleinen Dienstweg auf irgendeine Stelle gesetzt worden sind/ Ja da sind dann halt viele dabei, die halt gerade Zeit haben, weil sie keiner haben will. Und und das ist glaube ich vom Markt her etwas anderes als wenn man Doktoranden hat und ich denke nicht, dass es ehrlich ist eine Ressource zu machen wie (platform) und und jemanden (drauf?) promovieren lassen zu wollen, es sind zu viele Sachen dabei, die keine informatische Forschung sind, die keine biologische Forschung sind, die aber Zeit brauchen und ich habe keine Lust irgendjemanden zu sagen, ja ja du kannst auch promovieren. Und dann muss sie hier schauen, dass das System am Laufen bleibt. Und das ist Feature (A?), irgenwie ein Format hat sich geändert, dass er dieses Format auch ändert.	
Interview 25	46:11	Das finde ich nicht ehrlich, darum habe ich keine Lust Doktoranden auf (platform) einzustellen und das macht es natürlich schwer gute Leute zu bekommen gerade in der aktuellen Zeit.	
Interview 25			Ja, danke für die Einblicke. Das ist echt sehr wertvoll. Da würde ich auch gerne zum nächsten Teil übergehen, der sich eher so auf die Plattform Governance und Nutzung bezieht. Die erste Frage ist: wenn ich als Nutzer jetzt von (platform) die Plattform nutzen möchte, welche unter Regel liege ich da - welche Regeln unterliege ich da?
Interview 25	46:59	(Du sollst halt nicht?) unsere Daten abziehen und ein eigenes Ding aufmachen, ansonsten ist die Lizenz momentan nicht kommerziell. Das heißt, du sollst dich an uns wenden, wenn du die Daten kommerziell nutzen möchtest. -(6 s)-	
Interview 25			Und das war es?
Interview 25		Das war es. Du sollst uns nicht hacken.	
Interview 25			Und zu den paar, die Daten hochgeladen haben, welche Anforderungen an die Daten stellt ihr da?
Interview 25		Wir haben da ein Format und wenn die das mit mit dem Format hochladen, dann sind wir erstmal glücklich, ja. Und wir haben also die Leute, mit denen wir das gemacht haben, die haben wir so gut gekannt, dass da sozusagen da brauchten wir keinen Vertrag aufzumachen. Sondern die sagen uns, wir schicken euch diese Daten, könnt ihr die bei euch hosten. Also da das der Kontakt war informell.	
Interview 25	48:19		Welche Schwierigkeiten können denn auftreten, wenn Daten über die Plattform ausgetauscht werden?

Interview 25		(4 s) - Also die Sache ist immer mit der Sichtbarkeit, also da achten wir wahnsinnig drauf. Das nur Dinge sichtbar werden, die die sichtbar werden dürfen. Und ansonsten sehe ich keine Schwierigkeiten.	
Interview 25			Wie ist das mit der Nachnutzbarkeit der Daten, wie wie schätzt du die ein?
Interview 25	49:01	Also wir haben, alles gemacht, damit die möglichst nachnutzbar sind.	
Interview 25			Das heißt was konkret?
Interview 25		Verlinkung mit (Onthologie?), also das man nicht sagt, das hier ist was, sonder das (unv.)-Compound irgendwie 32 oder was. --- Bereitstellung in zwei Formaten, das eine ist sbml. Sbml ist Systems biology marker language. Das ist das / ein Austauschformat für mechanistische Modelle, das heißt also im Idealfall kann man sagen ich hätte gerne jetzt diese Daten mal (unv.)ein Modell. Da bekommt man ein ziemlich unsinniges Modell, aber man erstmal eins und könnte das in einem Werkzeug wie (???) einfach laden.	
Interview 25	50:07		Was war denn das zweite Format?
Interview 25		Das zweite Format ist Excel.	
Interview 25			Okay. Gut. Dannn habe ich noch eine Frage zu der Nutzergenerierung, wie bewegt ihr denn Forscher oder auch Organisationen (platform) aktiv zu nutzen?
Interview 25		Wir gehen auf Konferenzen. Wir haben mit mit verschiedenen Datenbanken Verlinkungen. Also das heißt, ich glaube bei z.B. ja auch dem berühmten ((platform)?) ist wenn Daten - wenn es zu ((platform))-Daten entsprechende Daten in (platform) gibt, dann ist da halt ein Link drin. Und das im Resultat angezeigt wird, dass man zu uns kommen kann, ähnliches haben wir auch in der anderen Richtung ebenso ((platform)?) und so.	
Interview 25	51:03	Wo man halt dann/ Also wenn ein Enzyme in ((platform)s?) ist, dann entsprechende Informationen bei uns abgreifen kann. Das sind so denke ich, die Hauptdinge. Wir sind natürlich auch gelistet, in Ressourcen wie wie (name) und so weiter und so fort.	
Interview 25			Siehst du denn noch weitere Möglichkeiten?
Interview 25		Ja klar. Also es gäbe weitere Möglichkeiten. Darüer denken wir nach momentan, sehen wir es nicht. Es ibt etwas das nennt sich (coredataressource?) von (Elixier?) Ich weiß nicht, ob du / kennst du (Elixier?)	
Interview 25			(Elixier?) ist ja das europäische Programm.

Interview 25	52:03	Ja genau super gut. Und die haben ein ein Programm, das nennt sich (coredataressource?). Da kann man sich bewerben und wenn man wichtig genung ist, dann wird man halt/ bekommt man diesen Stempel, dass man für die Bioinformatikstruktur (region)s sehr wichtig ist. Diesen Stempel haben wir nicht, diesen Stempel hat ((platform)?). Als eine von zwei deutschen Ressourcen, die die diesen Stempel haben. Also es ist schon eine tolle Sache und wir überlegen uns natürlich, ob wir was ähnliches machen könnten. Das ist eine Richtung. Die andere Richtung ist, also momentan sehe ich das nicht so ganz von wegen Größe. Dann gibt es Dinge wie wie (platform?).	
Interview 25	53:03	Das ist die Standardisierungs-App Initiative zu Enzymen der Arten und da sind wir sehr aktiv in dieser Standadisierung und unterstützen die Initiave, ein sogenanntes (platform?) zur Submission von Enzymdaten aufzuziehen und haben auch entsprechende Demodaten in (platform) und darüber hoffen wir natürlich auch Nutzer zu generieren. Wobei (platform?) momentan, also nach meinem Wissen schon ein bisschen das Problem hat, dass zu wenige Journal ihre Nutzer dazu verdonnern ihre Enzymdaten auf diese Art hochzuladen. Also führt dan ein weiterer Strom in (platform), wenn das zu pass kommt. Und gleichzeitig würden wir als einer der Unterstützer von (platform?) mehr Sichtbarkeit bekommen. Also derartige Dinge machen wir.	
Interview 25	54:10	Na ob man jetzt/ wir treffen schon ab und zu Leute, die für die wäre (platform) schön und sie wissen nichts von uns, ich weiß jetzt aber nicht was wir jetzt noch mehr machen könnten, um die zu erreichen.	
Interview 25			Vorhin haben wir ja auch schon über die Qualität der Daten gesprochen. Verwandt mit dem Thema Qualität finde ich ist das Vertrauen, finde ich ist aber auch irgendwie was anderes. Wie ist das bei euch, wie stellt ihr das Vertrauen in die Daten sicher?
Interview 25	54:47	(5 s) - naja wir machen schon so Sachen, wie wir offenlegen, was wir kuratieren und was wir machen. Dann können natürlich auch die Nutzer, also die meisten mit denen ich gesprochen habe, sagen, dass wenn sie Daten aus der Datenbank ziehen, sie diese Daten nochmal nachprüfen werden. Die benutzen die Datenbank, um um Hypothesen zu generien, sie schauen dann aber nochmal nach meistens und bei diesem Nachschauen ist natürlich interessant, sind die Daten gut oder sind sie nicht gut. Und auf diese Weise generieren halt auch die Nutzer ein Vertrauen.	
Interview 25			Apropro, Nachnutzung, wie stellt ihr sicher, dass die Nutzer von (platform) wissenschaftlich korrekt mit der Datenverwertung umgehen?

Interview 25	55:54	Not our business. Das können wir nicht sicher, dass können wir nicht -- wir haben ja keine Spur von den Leuten. Man könnte natürlich was machen, aber ich glaube, dann würde uns auch keiner mehr benutzen mehr, ja. Wenn wir jetzt irgendwie eifersüchtig draufschauen, ob jeder (platform)-Wert in dem richtigen Kontext benutzt wird. Dafür müssten wir ja erstmal alle Leute kennen, die (platform) benutzen. Und momentan ist es so, dass Datenbanken relativ wenig zitiert werden. Und wir hatten auch schon Fälle, wo wir wussten, wir werden benutzt, aber wir sind in dem Vortrag nicht benannt worden. Das heißt, also wir müssten dann schon wissen wer uns benutzt und dann müssten wir nachprüfen, ob die Folgerungen korrekt sind. Oder ob in diesem meist vielschrittigen Workflow unsere Daten auf die richtige Art und Weise benutzt worden sind. Ich glaube, dass ist aussichtslos. Ich glaube, dass ist ein bisschen so, als wenn man sagen würde, ja (unv.) wie stellen sie sicher, dass ihre Schweizer Taschenmesser nicht für schlechte Sachen missbraucht werden.	
Interview 25	57:31	Muss man dann halt die Firma man/ Man muss halt schon irgenwie Vertrauen haben, dass die meisten Leute diese Möglichkeiten, wie das Taschenemesser gescheit einsetzen.	
Interview 25			Eine Besonderheit von (platform) ist ja auch, dass es in der Wissenschaft fundiert ist, wie denkst du, beeinflusst die wissenschaftliche Kultur die Nutzung der Plattform?
Interview 25	58:04	(18 s)- Ja klar ist ein bisschen Kulturfrage ob man, ob man so etwas einsetzt wie eine Datenbank. Ich habe auch schon Leute getroffen, die mir erzählt haben, dass sie kein Bedarf haben, die Datenbank benutzen wollen. Schon einfach die Idee irgenwelche Werte ungefiltert, viel mehr gefiltert aus der Datenbank zu ziehen, anstatt die selber aus einem Paper zu ziehen, empfanden sie als etwas anrüchiges. Klar wir sind auch Teil eines Kulturwandels, ich meine, das heißt ja eigentlich eine Datenbank wie (platform) oder wie ((platform)?) heißt ja auf Deutsch, dass (Wissen?), das vorliegt ist so unstrukturiert, dass es Mittler braucht, die dieses Wissen strukturieren und nutzbar machen für moderne Anwendungen. In so fern sind wir Teil eines Kulturwechsels. Ist das /	
Interview 25	59:16	(ist das was du wissen wolltest?)	
Interview 25			Die Frage ist komplett offen. Also das ist kann von A bis Z alles sein, was bei der Antwort ist. Es ist immer super interessant, was auch dann mit der Wissenschaftlichen Kultur in Verbindung bis jetzt (unv.). Eine Rückfrage habe ich noch zu den Projekten, die ihr jetzt vorhabt also mit ((platform) Vis?) und auch der Kuration.
Interview 25		Die ??? Das Porjekt ja?	
Interview 25			Genau, ihr habt da jetzt diese Ziele gesetzt und seid da ja jetzt voll dabei. Wie kontrolliert ihr, dass ihr diese Ziele erreicht?

Interview 25	59:58	Mit der Kuratierung, also das Projekt hat zum Jahresbeginn angefangen. Die sehen sehr stark als Kontrollierbakeit dessen, was da automatisch gemacht wird, halten wir für sehr sehr wichtig. Wie genau wir das machen werden, also da gibt es ja schon schon Ansätze. Also - ((region)nC?) macht das auch im Rahmen von (elixier?), dass sie so eine Plattform haben, wo sie halt einfach -- leicht machen um vom - von der Region im Paper auf Daten zu kommen oder auch von Daten auf eine Region des Paper. Und in die die Richtung werden wir laufen, wir haben auch selber mal was gemacht vor-zehn Jahren noch mit Isabell noch zusammen.	
Interview 25	01:00:03	Wo wir wo wir halt uns angeschaut haben, wie kommt man von einem Datum in ein Paper rein und zurück. Und das das wird wohl ein wichtiger Teil sein.	
Interview 25			Und wenn ihr das jetzt durchführt, setzt ihr euch dann irgendwie konkrete Meilensteine oder wie geht ihr denn dann vor?
Interview 25		Ja, dann (Nähkästchen?) zum Thema Proposal schreiben. Natürlich haben Milestones im Proposal, und natürlich haben wir versucht, diese möglichst wenig konkret zu halten (lacht) also wir haben Meilensteine, ja. Und und wollen - ja, also das das ist ein BMBF-Projekt, also da wird man typischerweise gebeten, sagen wir mal man hat da so milestones im Halbjahresrhythmus,	
Interview 25	01:02:05	und die werden wir erfüllen. Und haben sie natürlich auch so formuliert, dass - auch Misserfolge eine Erfüllung sind, ja. Also ich denke, wie erfolgreich wir damit sein werden ist ziemlich offen, wir werden aber auf jeden Fall etwas lernen dabei. Und auch Dinge machen, wo andere Leute etwas lernen davon, das heißt es wird in dem Sinne weniger ein Serviceprojekt sein, als ein wissenschaftliches Projekt, wo dann/ wenn man herausfindet, wieso es momentan nicht geht eventuell auch schon ein Resultat ist.	
Interview 25			Dann komme ich auch schon zu meinen beiden letzten Fragen. Welche Entwicklung siehst du für (platform) in den nächsten zehn Jahren? Ganz allgemein gesprochen.
Interview 25	01:02:59	Ich hoffe mehr automatische Kuratierung, mehr Visualisierung, größere Intergration mit anderen Sytstemen.	
Interview 25			Und wovon hängt dann der Erfolg der Plattform ab?
Interview 25		(17 s)- Möglichst vielen Leuten klar zu machen, dass wir das gut machen - für die Nutzer.	
Interview 26	Time	Interviewee 26	Interviewer
Interview 26	00:25	I'll give you some more of my views.	
Interview 26	00:29	Okay, you're asking the question.	
Interview 26	00:32		Good. So my work is about success and success factors. I'm curious, how do you define a successful platform?

Interview 26	00:44	There's a number of factors that say, successful platform is I can speak to the (platform). In our case, it's because it has to have a global reach and and it has to be interesting to a wide scientific community. In our case, the structural chemistry community around the globe. So successful platform to me is one where people frequently come to from all around the world and whether it's a high adoption.	
Interview 26	01:12		And then what do you mean with high adoption factor?
Interview 26	01:16	I mean, people in our case, for example, for our content, they would want to come to us because it's the best structural chemistry content. So, you know, success is made, I think, because people can't really go to many other platforms, in this case, to get this type of information. So they come to the (platform). So there's a big drawing factor for people to come to us, because we've got high quality data. So that's another big factor. It has to be top quality, the kind of offering you have on the platform. In our case, it's top quality structural chemistry data	
Interview 26	01:50		will come. What do you mean by quality?
Interview 26	01:54	In our case, it's a several things it's precision of the data. So The way we store the molecules, we just have the highest precision on that, you know, atomic coordinates, lots of metadata data, everything extracted from the literature and then obviously post processed with semantic chemical checks. So it's a very high level of quality on molecular data. That's what the (platform) stands for. We really care about top quality, which is why we have an editorial team. And we have lots of semantic workflows and robotic checks and automation checks for integrity checks and data quality. And that raises the bar for quality. And I think any successful platform has to have top quality data in it. It can't be substandard.	
Interview 26	02:39		You just mentioned that there are not so many other platforms researchers can go to. So how do you strategically positioned (platform)?

Interview 26	02:53	it's a good question. I mean, now some other minor platforms have free platforms that people could go to like code For example, that's a free crystallography service. But it's only a third of the size and the quality is way, way poor. So I think the quality is a positioning factor for the (platform). We are more more of a closed platform, of course, because it's a subscription fee, although very cheap rates for academics, but it's much more expensive for industry partners. But our sort of, you know, stated trajectory for the next 10 years is to become more open and arguably, also more cheaper on the academic side, so that we can raise the adoption around the world even further. We're already kind of world leading with it. But obviously, there's more to go. And so in terms of positioning back to your question, you know, how do we position the success of the platform, I mean, it has to be at at the right price point for the end users for the academics, and obviously, it has to deliver this top quality, and so it has to be better than any of the competing platforms. And that's what we try and get across via our social media channels. In LinkedIn, and all the kind of marketing we do, we position it in that way, you know, high precise quality and comprehensive data on structural chemistry because we're we're the only data platform that has the biggest collection. Because we go for comprehensiveness of all crystal structures in the world. And that's another successful factor, it has to be comprehensive. Because if you only having 50%, and you know, people would come to the platform, and then half of it is missing. That's not good enough.	
Interview 26	04:30		When it comes to the management, What challenges do you see there, when it comes to the management of the platform in general,
Interview 26	04:41	biggest challenges database architecture. And basically, I mean, I'll give you an example. But it does cost us quite a lot of money with a with an architectural team to advance the database and evolve it and make sure it's future proof because you can appreciate if you have millions of molecules coming in with more than a million now, with all the surrounding data. You know, this kind of scales more and more. Now we want to store theoretical data as well, we want to store more metadata. So our database architecture needs to evolve in scale as well. And I think that adaptation to more flexibility for the future so that the database architecture can grow, and also then be disseminated via the cloud in a more flexible and are, you know, behind firewalls and for industry partners and all that sort of stuff. That's, that's quite an architectural challenge. And it needs quite some investment to get the database technology, right, and future proof and scalable.	
Interview 26	05:39		You just mentioned that you get so many data sets in how do you encourage researchers to upload data?

Interview 26	05:55	was it happens with the publication process? So we're a data repository? That's highly respected, so people would want their data to go in there. Anyway, I guess it's been built on the reputation from 60 years of our existence. And that's sort of got a head start in that sense. So if you're already that successful, and I think success builds further success, and people would want to submit the data to the (platform), because that's the best repository. But one other thing is I mean, we do things like CFD communications, and we allow people to publish more additional articles on their data. And then they can push out their data into our repository and then write a CFD communication. I guess they get additional chances to publish, rather than doing full articles and things like that. So there's a there's a few additional, you know, benefits you could have in that way. But I guess what we don't yet do and we should probably do this is some sort of additional incentives for people to publish the data maybe faster into our systems.	
Interview 26		But that's because we're charity, we have to be rather very careful. how one can incentivize. You know, I think the best incentive is that, you know, we're a comprehensive data source, it's top quality. If your data is in there, it's kept in the best place, please, please publish it in there. That's possibly still the best incentive. Rather than, you know, giving out Amazon vouchers for doing something that's not, you know, wouldn't be so good, I don't think	
Interview 26	07:24		and when it comes to future topics, what development Do you see for (platform) in the next 10 years,
Interview 26	07:34	big future topic is storing all the theoretically calculated data, so not just all the experimental data. So for example, at the moment is all X ray structures which are experimentally determined, but in the future, we would like to store crystal structure prediction landscapes and energy landscapes and everything that's been calculated by computers in bulk.	
Interview 26		So you could for example, you know, in visited in a number of years time, you might rerun a calculation on the database Which would recalculate all the hydrogen positions of all the molecules and then store the theoretically calculated result. So basically, the big thing is not only storing experimental data, but also supplemented it with computationally calculated theoretical data and bringing the two together.	
Interview 26		And that's a big sort of five year challenge, I think, going forward.	
Interview 26	08:21		And what does that this success depend on then?
Interview 26	08:27	execution needs to be executable in terms of workflows and running the algorithms to actually produce the results. And then again, it depends back on the database evolution of the architecture, because you need to then store the result in the modernized architecture, you haven't got that?	
Interview 26		You can't, whatever you calculate with the computers, you can't feed the results back into the database.	

Interview 26		So it's both execution in the sense of what the computers can do with the workflows and the algorithms and that you have the right protocol to achieve that. And then the results you need to be able to store them over wholistically back in the database,	
Interview 26	09:02		for my research is quite critical, you know, to motivate people to upload, as I asked before, and another part is the trust in the data. So how do you ensure that the researchers can trust your data?
Interview 26	09:18	So the concept of data trust is something that (platform) is is pushing, if not even pioneering going forward. I mean, that's something I care so much about. And I've talked about that for the last two years. And I want to say way more, the most of the keynotes, I give, I always talk about data trust and the factors for data trust. And for me, again, it's precision, its quality, its reputation of the platform and the repository. It's who's the community, you know, who are the scientists? Are they all you know, from around the world? And are they all already trusting the center, and a lot of these factors are already there for the (platform). But it's, I suppose it's never enough you can always improve and get better which is why we We do things like data quality checks in batch and data integrity checks with lots of workflows. And we hire people inside the editorial team to run analysis holistically on the data set.	
Interview 26		And we do even corrections from the past. Because obviously, we we only let the good stuff go in, in a sense, but some things slip through. And you need to kind of, you know, technologies advance so you can run better workflows or better algorithms in the future to check backwards for the last few decades. And you're always going to find some mistakes or some peculiarities which need fixing.	
Interview 26		So we play in all the fixes again, and then we do data updates, and we tell people that we've kind of you know, did all these integrity checks and here's, here's how it advanced even further towards even better quality. But again, quality is the top thing and precision.	
Interview 26	10:48		Yeah, thank you for your all insights and I think we have pretty much in time.
Interview 26	10:54	That's good. My pleasure. I hope it helps.	
Interview 26	13:23		and I think it's also very interesting for you because I also interviewed (platform) and (platform), so it's open. Yeah.
Interview 26	13:35	So obviously, you know that we collaborate with (platform) in cults with I know all of the fits people over there and cultural and I'm German myself, I know that quite well. And we probably will collaborate even deeper in going forward for the inorganic side. And we've just I don't know if you've seen the LinkedIn publication, but we've just struck a deal with the (institute) over in (town), us, not one that you've worked with, but they do all the diffraction patterns. And we've just done a collaboration deal with them. So we as a center, we do lots of international collaborations, I think that's also very, that's another success factor for a good repository.	

Interview 27	Time	Interviewee 27	Interviewer
Interview 27	0		Also, ich freue mich, dass das klappt. Vorher noch ein paar Worte zu mir: also ich (...) beschäftige mich(...) mit Technologien und Daten an der Grenze zur Wissenschaft. Und da sind Daten natürlich auch super wichtig und die auszutauschen auch sehr relevant. Bevor wir in die tieferen Fragen einsteigen, möchte ich gerne von Ihnen wissen, was Sie hier beruflich machen.
Interview 27		Angefangen habe ich mal als Biologie - Biologie studiert, promoviert auch im Basisbiologiebereich und bin hier ein Chameleon aus Wissenschaftler und Infrastrukturmaintaner.	
Interview 27		Meine augenblicklikche Funktion ist, ich leite den Bereich IT, Wissenschafts-IT und Datenmanagement. Das ist alles eng miteinander verwoben. Das ist ja ein relativ großes Institut an dem wir hier sind, (...) Personen, Technolgienplatformen für Durchssatztechnologien, da hängt sehr viel IT hinten dran an den Laboren. Durch unsere Wissenschaft sind wir auch international sehr verwoben.	
Interview 27		Also wir haben Forschungsprojekte mit den USA, wo dann Datenmengen, 100 TB oder so, ausgetauscht werden müssen. Das muss man organisieren, aber dafür braucht man auch Infrastrukturen. Ja und ich betreue hier zwei verschiedene Teams.	
Interview 27		Einmal das Datenmanagementteams, das sind hauptsächlich Wissenschaftler, die aus ganz verschiedenen Bereichen kommen, die sich gerne mit Datenmanagement beschäftigen. Momentan sind wir noch sehr infrastrukturell beschäftigt.	
Interview 27		Leider gibt es an der Uni nicht so viele gute Infrastrukturen für Datenmanagement, das baut sich im Moment auch alles erst auf. Und wir sind hier im Bereich der medizischen Daten, das ist noch ein ganz spezieller Fall, weil wir eben mit sensitiven Daten arbreiten und weil unsere gesamte Infrastruktur im Klinikbereich ist.	
Interview 27		Wir sind zwar hier auf dem Unigelände, aber unser Netz ist das Kliniknetz. Wir sind hier quasi in der Versorung und entsprechend gehen wir hier dann auch anders mit den Daten um als das irgendwo anders an der Universität der Fall ist.	
Interview 27		Das andere Team, was ich betreue, ist momentan leider sehr klein. Das ist die IT. Da geht es um Maintaining der eigentlichen Infrastrukturen vom Desktop PC bis hin zum HPC Cluster, wmot man dann große Datenmengen berechnen kann; sehr viele Datenspeicher.	
Interview 27		Wir haben momentan insgesamt 3 Petabyte an Daten, die zu verwalten sind, verschiedenste Arten von Daten natürlich, auf verschiedensten Infrastrukturen. Das sind so die beiden Schwerpunktsbereiche. Nebenbei habe ich noch ein Hobbie, das ist die Wissenschaft.	
Interview 27		Beschäftige mich mit Biomarkern für chronische entzündliche Darmerkrankungen, die man aus Blut gewinnen kann mit verschiedenen RNA Moleküle werden da verwendet.	

Interview 27		Und da entwickeln wir hoffentlich irgendwann eine diagnostische Methode, wie man verschiedene Entzündungserkrankungen characterisieren kann.	
Interview 27			Da bist du ja voll dabei in diversen Themen voll spannend.
Interview 27		Genau, überall mit drin.	
Interview 27			Das heißt welche Data Sharing Aktivitäten gibt es dann konkret?
Interview 27		Ganz unterschiedlich. Es gibt erstmal Data Sharing quasi institutsintern. Hier sind ja jede Menge Labore, die produzieren irgendwelche Daten und die müssen dann zum Wissenschaftler.	
Interview 27		Das sind aber sowzusagen weniger die Sharing Vorgänge, wo dann Kollaborationen und Authentifizierung eine wichtige Rolle spielen, sondern eher wie kriegt der Wissenschaftler schnell seine Daten.	
Interview 27		Das ist dann eher eine technologische Lösungsfrage, also wo packe ich die hin, damit der Wissenschaftler schnell dran kommt, damit er sie nicht erst noch zehn mal hin und herkopieren muss, damit wir nicht so viele Duplikationen der Daten im Netz haben und so weiter.	
Interview 27		Dann haben wir Technologienplattformen hier wie zum Beispiel das Sequenzierzentrum, das nicht nur Sequenziert für den Eigenbedarf sondern auch für andere Arbeitsgruppen am Standort aber aber national und international.	
Interview 27	5	Da haben wir dann, ja, Data Sharing in Form von: wir liefern Rohdaten aus an die Kunden. Das passiert - soll ich etwas sagen zur Infrastruktur?	
Interview 27			Ja, gerne, einfach offen drauf los.
Interview 27		Also das läuft im Moment alles über SFTP Server, also Secure FTP-Server, ja. Das ist eigentlich eher steinzeitlich, aber das ist so die beste Methode. Wir sind momentan dabei, noch andere Standards da zu verfolgen, die wir nicht selber entwickeln, aber andere entwickeln und die wir dann auch nutzen.	
Interview 27		Man muss ja immer gucken, dass der Kunde auch die gleichen Mittel hat um auch die Daten dann eben entgegen zu nehmen, die man dann zur Verfügung steht. Da geht es dann hauptsächlich darum, den Download der Daten zu beschleunigen.	
Interview 27		Weil so ein Datensatz von 3 TB über das Internet ist von Uni zu Uni recht einfach, weil die Anbindung entsprechend ist, aber wenn jetzt jemand extern sitzt, kann das schonmal ein etwas längeres Abenteuer werden.	
Interview 27		Dann haben wir noch Daten Sharing, da geht es gar nicht so um Daten ausliefern sondern Kollaborationen in nationalen und internationalen Projekten. Wir sind ja in diversen Forschungsverbünden hier, regional, überregional und international.	
Interview 27		Da werden Daten in Form von eigentlichen Rohdaten, Omicsdaten, Genomdaten, was auch immer fpür Daten geteilt, aber da werden auch sehr viele Ergebnisse oder eben auch prozessierte Daten geteilt. Da geht es mehr darum Excelshits, Airplot und solche Geschichten zu teilen und weniger die Forschungsdaten an sich.	

Interview 27	Es sind natürlich auch Forschungsdaten aber sind quasi die resultierenden Ergebnisse. Dazu nutzen wir auch unsere SFTP Infrastruktur, wenn es ein bsischen größer ist . Dazu nutzen wir alerdings auch Clouddinste, und zwar alle möglichen. Wir nutzen hier intern, vielleicht jetzt so als kleiner Einwurf, die Microsoft Cloud als Data Sharing Platform, weil wir haben ein Institutsaccount für Office 365 und da ist für jeden Nutzer ein Terrabyte OneDrive dabei.	
Interview 27	Und außerdem kann man dann die Sharing Funktionalitäten auch nutzen, dass man zusammen kollaborativ an Dokumenten arbeitet usw. Das ist da alles drin. Das heißt, intern wird daruüber auch sehr viel von den ganzen Office Geschcihten werden darüber geteilt.	
Interview 27	Das nutzen wir dann auch nach ausen, wenn das Gegenüber auch so ausgetattet ist, ist das der einfachste Weg. Bisher haben wir das große Problem, dass viele Daten da nicht hin dürfen in die Clouds, wegen des Datenschutzt. Weil da irgendwelche Daten drin stehen, die vielleicht Patienten betreffen oder sonst in irgendeiner weise sensitiv sind.	
Interview 27	Da fangen wir jetzt gerade an, das ist ganz neu quasi. Das ist gerade in der Testphase, Verschlüsselungssoftware zu verwenden, die uns die Cloud Inhalte verschlüsselt.	
Interview 27	Das Produkt heißt (name), ist eine in Deutschland geoutete Software, die dann im Team oder in der Companie oder Enterprise das ermöglicht, dass man eben Sachen verschlüsselt, und dann trotzdem kollaborativ	
Interview 27	auf diesen verschlüsselten Sachen arbeitet. Das testen wir im Moment gar nicht mit Forschungsdaten, sondern mit der Verwaltung. Weil die haben die ganzen Personaldaten und so weiteru nd die arbeiten auch ganz gerne mal von zuhause, aus dem Home Office.	
Interview 27	Das ist natürlich doofm weil man kann natürlich nicht die Personalakte von Hern sowieso irgendwie frei in die Cloud legen. Das geht darüber natürlich sehr gut, wenn das alles verschüsselt ist. Und dann ist es auch Datenschutzkonform.	
Interview 27	Und wenn es in der Verwaltung gut läuft - da sind die Dokumentgrö0en klein und die Fallzahlen nicht so hoch, dann können wir auch versuchen zu skalieren für die Wissenschaftler, genau. Was habe ich jetzt vergessen? Nö, also wenn es ums Sharing geht ist das glaube ich, ja.	
Interview 27		Darauf anknüpfend kann ich direkt fragen: welche Daten von anderen werden denn bei der Forschung hier verwendet.
Interview 27	Eigentlich das gleiche genau andersrum. Also wir haben Projekte mit anderen großen Instituten,	
Interview 27	wo auf der einen Seite, ja, Rohdaten hin und hergeschaufelt werde - immern och. Es gibt zwar den Trend, dass man jetzt die Daten einfach da liegen lässt wo sie sind und man bringt dann die Methoden zu den Daten.	

Interview 27		Und macht die Berechnung quasi da, wo sie liegen und holt sich nur noch die Ergebnisse. Das macht auch sinn, vor allem wenn man sich auch manche Projektgrößen anguckt. Wir haben zum Beispiel ein Forschungsprojekt mit einem Institut in den USA. Da geht es um 300 hundert - quatasch- um dreißigtauasend Teilnehmer einer Studie und für die wird jeweils das Genom sequenziert.	
Interview 27		30.000 Geniome, das ist ein Datenvolumen von sechs, siebenhundert terrabyte oder so. Das kopiert man nicht mehr in der Gegens rum. Das wird irgendwo strategisch hingelegt und dann wird halt der Zugang gewährt und dann ist dort,l wo die Daten liegen, ist dann auch die Mlgkichkeit, von Computerpower, dass man eben die Analysen, die man machen möchte, eben dort vor Ort machen kann.	
Interview 27		Das ist sicherlich was, was in Zukunft immer häufiger verwendet werden wird. Es gibt in zwischen auch Cloud provider, die wir allerdings nicht nutzen dürfen, weil sie in den USA sind, die darauf ausgelegt sind, die quasi nicht nur Speicherplatz zur Verfügung stellen sondern gleich auch den Computer daneben stellen, mit dem man dann die ausweertung macht.	
Interview 27		Bestes Beispiel ist die AmazonCloud, da gibt es jede Menge Webservices, die sehr gut funktuionieren, die wir aber nicht nutzen dürfen.	
Interview 27		Es gibt aber noch andere, die etwas spezialisierter sind, zum Beispiel auch unsere medizinische Daten, die haben dann spezielle Cloudumgebungen, aber das ist alles nicht vom Deutschen Datenschutz abgesegnet, das dürfen wir nicht nutzen.	
Interview 27		Und dann sharen wir ganz normal vom Antragsdraftdokument, das wir mit irgndwem schreiben, über Forschungsergebnisse bis hin zu irgendwelchen Publikationen, die man miteinander verfasst, und so weiter, da wird sehr viel und sehr rege hin und her geshared.	
Interview 27	10	Meistens über irgendwelche Clouds dann, Dropbox , OneDrive, GoogleDrive und was es da eben so gibt.	
Interview 27			Um jetzt in ein Zukunftsscenario zu gehen: auf welche Daten würdest du denn gerne noch zugreifen, wenn du könntest.
Interview 27		Als Wissenschaftler oder als Datenmanager? Als Datenmanager natürlich möglich auf alles, weil wer wird gefragt, wenn irgendwelche Daten nicht mehr im Zugriif sind?	
Interview 27		Dann wird der Datenmanager gefragt, der sollte wissen, wo die Daten sind. Als Wissenschaftler kommt das drauf an. Also. Vielleicht missverstehe ich die Frage auch ein bisschen.	
Interview 27			Ne, alles gut.
Interview 27		Ist das mehr auf lange Sicht gesehen? Also auf welche Daten möchte ich längerfristig zugreifen?	

Interview 27			Meine Idee dahinter ist zum Beispiel: Ich brauche die und die Asuwertungen von dem und dem, aber die habe ich momentan halt nicht, das würde ich gerne noch in Zukunft haben. Oder ist die Datenbasis, die man jetzt abgreift, ausreichend?
Interview 27		Sagen wir mal so, unsere Projekte sind eigentlich immer so aufgestellt, dass die Leute, die an einem Projekt teilnehmen, immer auf alle Daten zugreifen können, auch wenn die Daten irgendwo dann nochmal reanalysiert werden oder so, dann gibt es bei uns immer so, es gibt quasi, mehrere Phasen: die Rohdaten schlagen wirgendwo auf, dann werden sie analysiert, dann gibt es quasi den ersten Satz Ergebnisse, und dann kommt sowas - nennen wir Data Freeze - das heißt, dann wird sozusagen nicht mehr weitergearbeitet.	
Interview 27		Sondern das ist dann der erste Stand, der kommuniziert wrid. Und das kriegen dann alle zur Verfügung und alle können dann raufgucken und sagen: "nee, die Analsyse gefällt mir nicht, die müssen wir nochmal neu machen", oder " da ist irgendwas falsch gelaufen", oder " da muss noch was anderes gemacht werden" und dann geht es quasi in die zweite Runde.	
Interview 27		Und dann arbeitet man sich von Data Freeze zu Data Freeze. Gerade bei diesen Projekten, wo sehr sehr viele Daten drin sind, muss man das auch so machen, weil sonst gibt es tausende Versionen von irgendwas, wo man dann nicht mehr durchblichkt, sodass, ja also, wie gesagt, die Data Freezes sind immer das, was sozusagen, das man sich anguckt.	
Interview 27		Was dazwischen ist, das muss eigentlich nicht jeder sehen. Das möchte man vielleicht sehen um zu sehen wie ist ein Ergebis zustande gekommen, also wie ist die Analyse verlaufen.	
Interview 27		Aber diese ganzen intermediären Files, die werden dann auch längerfristig, also wenn ein Projekt dann abgeschlossen ist, man an dem finalen Data Freeze angekommen ist,	
Interview 27		, dann ist es auch so, dass man vielleicht so, dass man dann Data Freezes dazwischen auch einfach weglöscht weil man sagt, die brauchen wir nicht mehr, und dann wird quasi ein, ja, ein inforamtiver Datensatz zurückbehalten und die Rohdaten gehen sowieso anfangs direkt nach der Produktion ins Archiv.	
Interview 27		Sodass man da immer mal wieder drauf zurück greifen kann, wenn man das muss, dann holt man sie eben hervor und kann eine neue Analyse machen irgendwann.	
Interview 27	15	Letztendlich möchte man einen finalisierten Datensatz von Ergebnissen haben und vielleicht am besten eine sehr gute Dokumentation, wie es dazu ´gekommen ist, sodass man mit den Rohdaten wieder anfangen könnte. Das ist der Idealfall, aber leider nicht der Bormalfall.	
Interview 27			Darauf anschließend kommt gut die Frage: Welche Anforderungen an die Daten gestellt werden auf die geteilten Daten?

Interview 27	Genau, da gibt es ja die schönen FAIR Principles, die umschreiben das ziemlich gut. Für hier für den Standort ist es vielleicht ein ganz schönes Beispiel.	
Interview 27	Wir haben ja hier die medizinische Fakultät, dass sind so 50 Klinioken und Institute, die alle irgendwie an ähnlichen Fragestellungen arbeiten, natürlich jeder für seine Krankheit oder jeder für sein Spezialgebiet usw.	
Interview 27	Aber letzendlich haben wir alle ähnliche Arbeitsweisen und arbeiten mit ähnlichen DAten - oft auch mit den Gleichen Daten. Weil wir haben ja zum Beispiel die Biobank, wo die ganzen Bioproben drin sind zu einem Patienten gibt es dann auch vielleicht 5, 6 verschiedene Bioproben, die dann aus unterschiedlichen Blickwinkeln angeschaut und analysiert werden.	
Interview 27	Im moment ist das aber auch alles disconnected. Vielleicht kann man bei der Biobank nachfragen und sagen: wem hast du diese Bioprobe eigentlich alles gegeben? Also vieleicht habe ich zu dem Patienten auch das und das .	
Interview 27	In den seltensten Fällen klappt das aber wirklich. Idealerweise hätten wir für diesen ganzen Wust ein einheitliches ID Management, also dass man die Daten identifizieren kann.	
Interview 27	Dann ein vernünftigen Metadatensatz an dem Datensatz dran, dass man auch weiß	
Interview 27	an den Daten dran, dass man auch weiß, was sind das überhaupt für Daten, und wer hat die erzeugt und womit und wann und wie und überhaupt.	
Interview 27	Dann sollte das ganze idealerweise durchsuchbar sein, wenn die Daten selber nicht durchsurchbar sein können, wegen Binärformat oder sonst irgendwas, dann eben die Metadaten. Dann sollte die möglochkeit sein, an die Daten ranzukommen.	
Interview 27		
Interview 27	also Da sollte irgendwie ersichtlich sein, wem gehören die Daten denn und wen kann ich ansprechen - oder die Daten sind sowieso downladbar, das wäre natürlich das einfachste, was bei medizinischen Daten in der Regel meistens nicht der Fall ist, weil da muss ja auch immer mit Einverständniserklärung und so weiter gearbeutet werden, also da haben wir noch ein paar Fallstricke.	
Interview 27	Habe ich was vergessen? Das wars geigentlich, genau. Diese Grundvorsaussetzung sollte geschaffen werden. Das ist auch ein Grundatz für ein Projekt, was wir hier gerade versuchen zu etablieren an der medizinischen Fakultät. Wir etablieren hier ein zentralisiertes Datenmanagement, wo der Chemiker, der Laborzellbiologe, der Professor - egal wer - quasi, ein Fenster ins Datenmanagement bekommt und das möglichst benutzerfreundlich.	
Interview 27	Also über ein Grafisches Interface und so weiter, da hat er dann quasi eine Netzwerkfestplatte, wo er seine Daten hinspeichern kann und wenn er die Daten dann ordentlich mit Metadaten angereichert hat, dann kann er irgendwann den Submit knopf drücken und dann landen diese Daten in einem Archiv.	

Interview 27		Und in diesem Archiv sind die Daten nicht mehr veränderbar, die liegen dann da einfach, die sind durchsuchbar, die sind analysierbar und vor allem sind sie auch teilbar. Ich kann in diesem grafischen System kann ich dann sehen" dem gehören die Daten".	
Interview 27		Dann kann ich ihn einfach fragen: "kann icha uf deine Daten zugreifen?" Und dann kann der andere sagen: ja. Dann muss nicht irgendwie das Datenmanagement angesprochen werden oder der Admin oder sonst irgendwie sondern wie bei Dropbox, wenn ich selber entscheiden kann, der darf auf die Daten gucken - so soll das da auch werden.	
Interview 27		Das vielleicht für dich ganz interessant. Das wird schon gelegt, das System. Die Niederländer sind da sehr, sehr weit. Die haben ein nationales Datenmanagementsystem für ganz Holland - unglaublich.	
Interview 27		Und die haben für die unterschiedlichen Universitäten, haben die das auch als Schwerpunkt. Also die haben eine echte Datenmanagementgruppe.	
Interview 27		Da sitzen teilweise zwischen 5 und 15 Menschen dirnnen. Das sind It Leute, Entwickler	
Interview 27		das sitzen Professor für Datenmanagment, und die entwickeln für die Universitätsstandorte Datenmanagement. Das System, was wir uns quasi qbgucken wollen, das läuft im Moment in Utrecht und wird an neun weiteren Standorten gerade ausgerollt - nennt sich Yoda "Your DAta".	
Interview 27	20	und das bietet genau diese ganzen Funktionalitäten, also: grafisch, nutzerfreundlich, usermanagement, metadatenanreicherung, archivierunf, durchsuchbar, das ist alles drin.	
Interview 27			Das ist dann auch so die idealvorstellung für den zukünftigen Zugriff?
Interview 27		Jein, also ich war mitte letztens Jahres war ich auf einer Tagung in Brüssel von der EU. Da ging es um die Europäische Health _Cloud. Da gibt es irgendwie so eine Wahnvorstellung, dass alle gesundheitsdaten aus Europa in einer zentralen Cloud sozusagen gespeichert werden.	
Interview 27		Das wäre schön ,wenn das so wäre. Für die Forschung wäre das der Idealfall, aber das wird natürlich nicht so sein. Es gibt dann auch noch initiativen auch etwas niedrigerem Niveau. Wie zum Beispiel deutschlandweit. Da gibt es die medizininformatikinititative, da wird gerade an den deutschen Klinikstandorten, gibt es sozusagen Schwerpunktgruppen, die sich um die Datenintegration bemühen am Standort, also die quasi die ganzen Daten zusammenführen, zentralisieren, miteinander verknüpfen, weil wir haben in der gerade Medizin haben wir auch Daten zu einem Individium in sehr unterschiedlichen Systemen.	

Interview 27	Also wir haben einmal Daten aus dem Versorgungskontext, aus dem Krankenhausinformationssystem, der Patient hat aber auch bei Studien mitgemacht und der hat der auch Bioproben hinterlassen, wo andere dran forschen. Da haben wir auf einmal in fünf verschiedenen Arbeitsgruppen, in sechs verschiedenen Insituten, in zwanzig verschiedenen Systemen haben wir auf einmal Daten zu dieser Person.	
Interview 27	Und im moment sind hier alle zum großen Teil nicht miteinander verbunden. Und das soll jetzt qwuasi klinikstandort bezogen in Deutschland passieren. Da gibt es dann lokale Datenintegrationszentren.	
Interview 27	Und das ganze dann möglichst am besten so generisch und standardisiert, dass man dann die verschiedenen Datenintegrationszentren miteinander verknüpfen kann, dass man auch Daten tauschen kann zwischen den Standorten, das da dann keine komplizierten ETL Prozesse geben muss oder sonst irgendwas um überhuapt die Datenmiteinander reden zu lassen sondern dass das eben möglich ist.	
Interview 27	Hätte dann auch den Vorteil, dass wenn der Patient hier in Schleswig-Holstein nicht mehr leben möchte und dann nach Süddeutschland zieht, und da mit seiner chronischen Erkrankung wieder ins Krankenhaus geht, das man von dem quasi den gesamten Krankheitsverlauf weiterverfolgen könnte.	
Interview 27	In Skaninavien ist es ja so, da sind die ganzen Informationen an die Sozial ID geknüpoft und da hat man eine Krankenakte von der Geburt an mit allem drum und dran mit jedem Medikament, das er verschriebne kriegt, mit allem Pipapo. Die Dänen zeigen uns gerade, wie es geht.	
Interview 27	Die schreiben immer so, ein ganzes Land, eine ganze Kohorte. Also ideal für mich hier wäre, wenn wir das für diesen Standort hinbekömmen wprden, weil wir sind zweitgrößtes Klinkium in Deutschland, Und wenn wir das für diesen Standort hier hinbekämmen, da können wir schon genug Forschung mit machen.	
Interview 27	Idealerweise sind die Daten in einer Form vorhanden und accesierbar, dass man sie auch mit anderen Standorten teilen kann, mehr kann man glaube ich nicht machen.	
Interview 27		Welche Probleme treten denn beim Verwenden von den Daten auf?
Interview 27	Ich muss mal kurz überlegen, aus welcher Sicht ich das jetzt beantworte. Wenn ich als Bioinformatiker antowrten würde, dann ist es das Format. Weil, die Daten, die wir hier so verarbeiten in der medizischen Forschung, da gibt es standardisierte Formate, aber das heißt nicht immer, das Format X auch wirklich Format X ist.	
Interview 27	Das heißt, das wird dann so genannt, aber ees sieht dann doch ein bisschen anders aus. Und dann gibt es jede Menge Computerverfahren oder Programme, mit denen man dann rechnet, irgendwelche Bioinformatischen Tools.	
Interview 27	Und Die hat irgendwer programmiert, der das dann natprlich mit dem Fileformat X gemacht hat, aber das andere Fileformat X kann das dann nicht mehr lesen.	

Interview 27		50% der Zeit eines Bioinformatikers ist das Umformatieren der Daten - von einem Format ins nächste, damit das irgendwie	
Interview 27		das ist sicherlich, sozusagen auf der analytischen Ebene dasgrößte Problem. Das Problem aus der wissenschaftlichen Sicht generell ist die Verfügbarkeit der Daten, wie komme ich an die Datenüberhaupt ran und - was ich hier am Standort sehe- ist, dass nicht wissen über die Existenz von Daten.	
Interview 27		Hier werden unglaublich viele Daten doppelt und dreifach erzeugt. Quasi die gleiche Analyse von unterschiedlichen Arbeitsgruppen wirder gemacht, weil sieeinfach nicht wissen, dass es diese Daten schon gibt, weil sie eben nirgendwo liegen, wo sie finbar, durchsuchbar und so weiter sind.	
Interview 27		Das ist ein sehr großes Problem. Das verbraucht unglaublich viel Zeit und Geld, was eigentlich unnötig ist. Und ein anderes Problem, was es auch noch gibt, gerade in der medizischen FOrschung, ist der technologieFortschritt.	
Interview 27		Weil die Labortechnologien etnwickeln sich so schnell, die werden immer effektiver, da kann man immer mehr Patientenzahlen, immer mehr Daten in kürzester Zeit.	
Interview 27		an kommt eigentlich mit der Analyse nicht hinterher. Und Bevor man den alten Datensatz fertig analysiert hat, gibt es schon wieder eine neue Technolgie auf dem Markt, die noch besser ist, noch feiner und überhaupt.	
Interview 27		Das überschöägt sich immer so ein bisschen. Oft bleiben alte Daten dann auf der Strecke und werden eigentlich nie so richtig ausgewertet.	
Interview 27	25	Das ist natürlich sehr schade - sehe ich auch eibfach als Problem, weil es hat natürlich auch Geld gekostet, diese Daten zu erzeugen. Also viele Daten werden gar nicht wirklich bis zum Ende quasi ausgewertet. Man kännte da viel mehr rausholen.	
Interview 27			Wie ist das denn mit finanziellen Aspekten - gibt es da irgendwelche Barrieren?
Interview 27		Ja, natürlich, vor allem an Universitäten und mit der Deutschen Forschungsgemeinschaft. Wenn man jetzt mal aus der technologiebene guckt: Daten müssen irgendwo liegen und gerade in der medizischen Forschung sind Daten auch sehr voluminös.	
Interview 27		Habe vorhin schon gesagt, wir sind hier bei 3 Peta im Moment hier - das ist nicht viel aber für ein Institut schon einiges. Und dafür müssen natürlich Infrastruktur her.	
Interview 27		Und IT Infrastrukturen sind leider nicht billig, und sind auch nicht sehr langlebeig. Also der Zyklus ist immer so fünf Jahre. Nach fünf Jahren muss ich zusehen, dass ich das System quasi erneuere.	
Interview 27		Gerade bei Datenspeichersystemen ist das kritisch - ein Computer kann mal kaputt gehen, dann nehme ich eben den nächsten und rechne meine Daten damit.	

Interview 27		Aber wenn der Fileserver kaputt geht, ist es schwierig an die Daten ranzukommen. Natürlich haben wir unsere Daten hier alle in irgendeinerweise gesichert, die Systeme sind alle so redundant, dass wenn irgendwas kaputt geht, bicht die ganzen Daten weg sind, aber es ist schwierig sozusagen vernünftige Technologien aufzubauen - da kommen wir zu dem finanziellen Aspekt.	
Interview 27		Die DFG ist natürlich bestrebt, es sind ja alles steuergelder	
Interview 27		die wir hier ausgeben, dass wir hier nicht verschwenderisch sind und so weiter. Leider hat die DFG aber als eine der Geldgeber da in der Verwaltung Leute sitzen, die etwas eigenartige Ansichten davon haben, wie wir hier arbeiten.	
Interview 27		Die meinen, wir können unsere Daten auf externen Festplatten managen oder sowas. Das ist echt ein Problem. Das größte Problem ist: wie kommen wir dann an das Geld, dass wir neue Infrastruktur kaufen können?	
Interview 27		Wir können natürlich in Forschungsvorhaben immer etwas einpreisen, in Forschungsanträgen, wo wir sagen: "Ja, bei diesem Forschungsvorhaben kommen schätzungsweise 20, 30 Terrabyte an Daten zustande und dafür müssen wir dann eben gewissen Budget vorhalten damit wir Festplatten kaufen können oder die bestehenden Systeme erweiter und so weiter.	
Interview 27		Das ist immer sehr schwierig, weil so betreibt man keine IT Infrastruktur. Da kauft man eher für 3-400.000 Euro einen großen Klotz, der dann schön performant und redundant ist, aber nicht eben mit 25 Terrabyte sondern eher so: noch ein halbes Petabyte ran, nochmal für 100,000.	
Interview 27		Das ist sehr schwer abbildbar in der Art und Weise, wie das Funding funktioniert im Moment. Es gibt zwar von der DFG spezielle Verfahren für Großgeräte, Forschungsgroßgeräte, da kann man auch IT Budgets drüber bekommen- da habe ich zum Beispiel vor zwei Jahren ein Antrag eingereicht und bewilligt bekommen	
Interview 27		1,7 Millionen Euro. Damit bauen wir jetzt die Infrastruktur für das Datenmanagement hier für die medizische Fakultät.	
Interview 27		Allerdings reichen die 1,7 Millionen für die Anschaffung und für die ersten 5 Jahre Wartung und Support. Und ab dann läuft das Ding entweder von alleine oder - also aber ab fünf Jahren muss man ja schon dran denken: Moment, die Systeme müssen ja schon erneuert werden.	
Interview 27		Das werden ja nicht weniger Daten, das werden immer mehr Daten. Da muss dann Geld wieder irgendwo herkommen.	
Interview 27	30	Der Finazielle Aspekt ist da absoulut gegeben. Bei den kleinen Sachen, externe Festplatten, Clouspeicher, das ist alle Pillepalle. Da geht es wirklich um die IT Infrastruktur, man kann das nicht mit externen Festplatten, das geht nicht.	
Interview 27			Um mal auf einen ganz anderen Aspekt zu kommen: wie ist das denn mit der wissenschaftlichen Kultur. Hat die auch einen Einfluss darauf, wie man Daten miteinander austauscht?

Interview 27	Ja, absolut. Ganz generall gesehen: der Wissenschaftler ist ja ein freigeist, und so geht er auch mit seinen Daten um. Wissenschaftler gehen primär den Weg des geringsten Wiederstands.	
Interview 27	Und so wird auch das Datenmanagement gemacht: irgendwie, jedes Mittel ist recht. Es ibt unglaublich wichtig, dass man Vorschriften oder Policies zur Hand hat " so musst du deine Daten managen", Ansonsten ist das Wildwuchs?	
Interview 27		Das heißt, es muss auch vom Managemt kommen?
Interview 27	Ja natülich, dass muss absolut vom Management kommen. Also, wenn hier ein Mitarbeiter in die Abteilung kommen, dann gibt es ganz klare Anweisungen, wo sind hier welche Daten in welcher Form.	
Interview 27	Das wird noch schlimmer in Anführungszeichen, wenn wir jetzt das metadatengestütze	
Interview 27	Datenmanagement einführen, weil dann werden viele Daten nur noch abgelegt, wenn da auch Metadaten dran sind, sonst werden sie nicht abgelegt.	
Interview 27	Also Diese Datenmanagementressourcen sind quasi nicht bedinungslos sondern da gibt es immer die Bedingung, die Metadaten müssen stimmen.	
Interview 27	Da laufen dann auch die Routinen, die prüfen, ob die stimmen die Metadaten, ob die vollständig sind und so weiter.	
Interview 27	Kann vieles automatisieren natürlich auch. Gerade auch die Datenmanagemensachen, die hier von den Laboren kommen und so weiter, das ist ja alles sehr standardisiert und die LAborgeräte selber geben meistens Standardiserte Metadaten raus, da kann man sehr viel standardisieren, sobal der Faktor Mensch kommt, dann wird es messie.	
Interview 27		Hat denn der persönliche Faktor auch einen Einfluss darauf, inwieweit man Daten teilt?
Interview 27	Oh, ja. Inwieweit? Ja, auf jeden fall. Vor allem in der Medizin. Es gint ja hier so unsichtbare Königreiche, in der Medizin. Verschiedene Klinikdirektiren oder Bereiche denken ja, sie und kein anderer.	
Interview 27	Und entsprechend verhalten sie sich auch im Datenmanagement, dass sie sizusagen "die Daten, das sind meine Daten. Und die geben ich dir überhaupt nicht"	
Interview 27	Das ist auch noch ein Problem. Ich weiß nicht, ob das auch in anderen Dsziplinen auch so ist, in der Medizin ist das absolut so. Es ändert sich zum Glück im Moment ein bsischen, weil alle sind auf irgendwelche daten von anderen angewiesen mehr und mehr.	
Interview 27	Aber es gibt immer noch die alten Hasen, die sagen: "meine Daten kriegst du nicht". Das ist auch ein Problem, dem wir gegenüberstehen, in diesem medizinischen Datenmanagmentinfrastrukturpojekt,.	
Interview 27	Wir wissen noch nichtm wie gerne das genutzt werden wird. Immerhin, dadurch, dass wir dem Nutzer selber quasi nur die Benefits geben in dem wir sagen: "hier, hier ist quasi ein Sandkasten, indem du die Daten managenen kann.	

Interview 27		Wenn es keiner sehen soll, dann sieht es keiner. Du hast aber die Möglichkeit, den Schalter zu betätigen, und dann kann es jemand sehen, mit dem du die Daten teilen willst".	
Interview 27		Dadurch geben wir das so ein bisschen ab und ermuntern die Leute, dadurch trotzdem ihre Daten vernünftig zu managenen als sich dann doch wieder die Netzwerkfestplatte zu kaufen und ins Institut zu stellen.	
Interview 27		Ich denke mal Macht ist sicherlich ein Faktor, der beim Sharing von medizischen Daten absolut eine Rolle spielt.	
Interview 27		Und Konkurrent natürlich auch. Das ist zum Teil ein sehr kompetitives Feld, wo man auf Konferenzen echt aufpassen muss, dass man keine unpubulizerten Sachen präsentiert sonst werden	
Interview 27		sonst werden die sofort gescoopt und von jemand anders publiziert. Also das ist sicherlich auch ein Faktor, der da eine Rolle spielt.	
Interview 27		Da gibt es eben Menschen, die denken sehr kompetitiv und die passen da dann voll rein und sind da auch sehr restriktiv, Und dann gibt es andere, die sehen den Mehrwert von Sharing, das sind allerdings leider noch nicht die Mehrheit, das ist eher eine kleiner eGruppe.	
Interview 27		Und die sind sehr offen, die sagen einfach: hier nimm die Daten, tu was damit. Die vertrauen qusi darauf, dass wenn mir jemand die Daten gibt, dass ich dann falls ich dann eine Publikation daraus mache, dass ich dann eben als Coautor mit draufstehe, sowas in der Richtung.	
Interview 27	35	Aber rechnen sollte man damit nicht. Dementsprechend vorsichtig ist man dann auch.	
Interview 27			Und wie ist es dann - auch passend zu dem Thema - wie ist denn die Anerkennung von dem Data Sharing?
Interview 27		Also wie gesagt, es gibt eine Community, die ist sehr fokusiert auf das Data Sharing und so eine Community ist zum Beispiel die skandinavische Community.	
Interview 27		Also da sind die alle einigermaßen gleich: Norweger, Schweden, Dänen. Die gucken ein bisschen schräg, wenn man nicht shared, sagen wir mal so. Für die ist das selbstverstädnlich und die leben das einfach.	
Interview 27		Die leben das aber auch in ihrem staatlichen System. In Dänemark, wie gesagt, alle Daten hängen an dieser Sozialnnummer. Ich kann die Daten auch einsehen, ich kann zu der zentralen Behörde, ich weiß nicht wie die da heißt, hingehen und sagen "ich möchte gerne meine Daten sehen".	
Interview 27		Dann setzen sie dich an einen Computer und kannst du in deine Daten gucken was von deiner Geburt bis heute alles so mitgeschnitten wurde.	
Interview 27		Wenn du in so einer Gesellschaft groß wirst, dann hast du natürlich ein ganz anderes Verhältnis zum Sharen von Daten als wenn du hier in Deutschland , wo jedes mal wieder der Datenschützer kommt: "Äh äh, rote Lampe".	
Interview 27		Genua. Und dann gibt es die Leute, die nur ihren eigenen Mehrwehrt sehen, wenn jemand mit ihnen die Daten shared. Das ist XY da primär, aber hinterher hat man selber, wenn man seine Daten preisgegeben hat, nur der andere.	

Interview 27	Das ist dann natürlich eine Enttäuschung, für den, der geshared hat. Das ist nur so eine vorgeschobene Anerkennung, die tun immer nur so als wenn.	
Interview 27		Ein weiterer Aspekt, auf den wir noch gar nicht zu sprechen kamen, ist der Zeitaufwand? Beeinflusst das auch das Verhalten?
Interview 27	Ja, auf jeden Fall, kommt ein bisschen auf die Daten an. Also wie viel und vor allem um welches Volumen es da geht. Habe ich ja vorhin schon angespriochen, der Trend, Daten da zu lassen wo sie sind und dann die Methoden dahinzubringen, sind eben eine Lösung, wenn es um sehr große Datenmengen geht.	
Interview 27	Aber ich glaube beim Sharing eher der Release of Exit, also wie einfach komme ich an die Daten ran und wie gut kann ich mit den gesharten Daten arbeiten,.	
Interview 27	Da sind eben solche Enterprise Cloud Solutions wie Onedrive oder DropBox oder so mit entsprechenden Kollaboraionstools da schon drin, sind da natürlich sehr hilfreich.	
Interview 27	Ich glaube Google, waren die ersten mit Google docs wo man dann gemeinsam auf Dokumenten arbeiten konnte und so.	
Interview 27	Inzwischen machen das ja auch viele andere, wo das auch möglich ist. Zeitfaktor.	
Interview 27	Also, ich glaube die Zeit, die jemand aufwendet um Daten zu sharen in der Wissenschaft ist, die ist schon recht groß inzwischen, weil das eben ein Every Day Business ist.	
Interview 27	Und ja, je einfacher das ist, aber dass das jemand negativ empfinden könnte, ich brauche zu vielö Zeit um Data sharing zu machen, glaube ich nicht.	
Interview 27		Welchen von den Barrieren und Problemen, die wir jetzt besprochen haben, siehst du denn als größte Barriere?
Interview 27	Die größte Barriere sitzt vor dem Computer, das ist der Nutzer, der seine Daten nicht sharen möchte oder nicht managen möchte.	
Interview 27	Also die Technologie ist da, es gibt für alles Lösungen. Das Problem ist der Nutzer. Wie weit lässt er sich darauf ein, die Datenmanagementlösungen, die ihm angeboten werden, zu nutzen.	
Interview 27	Das hängt dann natürlich auch daran, wie gut sind diese Lösungen und wie einfach zu benutzen, das ist noch ein anderer Aspekt.	
Interview 27	Und dann eben dieser Machtaspekt "nö, das sind meine Daten".	
Interview 27	Ich publiziere, ich behalte das, und mein Schatz, mein Preciuos. Und, ja. Es gibt ein schönes Konstrukt, das versucht, oder das ist so ein Pseudo-versuch, wie man besser sharing macht, und das ist die Konsozialforschung.	
Interview 27	Das wird ja gerade in der Medizin sehr gerne gemacht, weil man dann größere Fallzahlen bekommt und so weiter, aber die Konkurrenz in den Konsotrium ist immer sehr groß.	

Interview 27		Letztendlich gibt es, ich kenne kein großen Forschungsverbund, der kompetitionslos läuft. Man hat immer noch die Competition dadrin und vor allen Dingen der Publikationsdruck, der das ganze auslöst, und dann.	
Interview 27		Da versteckt man sich quasi nur in dem Konsortium und sagt: "ja, wir sind alle eine große Familie, damit ich an die Daten der anderen komme, damit ich dann irgendwas machen kann.	
Interview 27	40	Darum geht es natürlich. Also das Problem ist tatsächlich der Mensch, der Nutzer, würde ich sagen.	
Interview 27			Ja, danke für die Einschätzung. Ich selber habe ja bereits mehrere Interviews geführt (...)
Interview 27			Ich gucke mal, welche Fragen jetzt schon alle abgehandelt wurden.
Interview 27			Bei den rechtlichen habe ich noch eine nachfrage: Du hast ja gesagt, es gibt die Datenschutzrichtlinien, die da viel verhindern. Welche anderen rechtlichen Aspekte beeinflusst noch das Data Sharing?
Interview 27		Also in der medizinischen Forschung sicherlicher der Konsent. Also das Einverständnis des Patienten, dass seine Daten für die Forschung genutzt werden dürfen und in welcher Form.	
Interview 27		Das ist in den letzten Jahren, also wenn man zurück schaut ist das immer sehr restriktiv gehandelt worden, also, wenn ein Patient sich entschließt, an einer Studie teilzunehemn, dann gibt es für diese Studie einen Konsent: "Ja, ich bin einverstanden, meine Daten dürfen für diese Studie genutzt werden.".	
Interview 27		Das ist eine direkte Zweckbindung. Das heißt, die Daten dürfen für diese Studie verwendet werden, für nichts anderes. Das heißt, danach stehen die Daten auch nicht für andere Sachen zur Verfügung, die müssen sogar gelösht werden.	
Interview 27		Das ist natürlich, ja, fatal für alle möglichen Forschungsrichtungen, die sozusagen über eine lange Zeit laufen wollen oder wenn die die Daten wieder benutzen wollen.	
Interview 27		Das ist ja gar nicht möglich. Deshalb nennt sich ein verfahren, das nennt sich Broad Konsent. Das ist das, was jetzt hier am UK,SH auch ausgerollt wurde vor 1,5 Jahren ungefähr. Das heißt, der Patient gibt am Anfang eine generelle Einverständniserklärung, dass alle seine Biomaterialien, die aus dem Versorgungskontext irgendwie übrig bleiben oder sonst irgendwie anfallen, dass damit Forschung gemacht werden darf. Punkt.	
Interview 27		Da gibt es keine Zweckbindung- die Zweckbindung ist die Forschung, das war es. Das heißt, die Materialien können eingelagert werden, die können da ganz lange liegen, die können irgendwann aus dem Bioprobenlager geholt werden, dann können damit eine Studie gemacht werden, die Daten einer Studie liegen irgendwo, die können wiederverwendet werden und so weiter.	
Interview 27		Das ist ein wesentlich offeneres Konstrukt. Ich glaube wir sind das einzige Klinikum in Deutschland, das diesen Broad Konsent i n dieser Form ausgerollt hat bisher.	
Interview 27		Da sind wir auch so ein bisschen Vorreiter.	

Interview 27		Alle anderen haben eben, ja, das Problem des Konsentmanagements. Die Frage ist immer, zu was hat der Patient sein OK gegeben und zu was nicht. Und die Urpsrungsfrage war?	
Interview 27			Welche rechtlichen Aspekte
Interview 27		Ja, genau. Und dann, welche Infrastrukturen darf ich nutzen? Das ist auch absolut. Also gerade welche Daten dürfen in welcher Form wo sein, das ist in der medizinischen Forschung immer die Frage, wobei es dafür auch Vorschriften gibt.	
Interview 27		Es gibt dann Vorgehen das nennt sich Pseudonymisierung, das heißt, wobei man dann bei einem Genomdatensatz überlegen muss, wann ist denn so ein Datensatz denn pseudonomisiert.	
Interview 27		Also welche Merkmale muss ich quasi aus dem Genom löschen, damit wirklich kein Bezug mehr zu der Person hergestellt wird. Darüber streiten sie sich bis heute, da gibt es auch keine wirklich gute Lösung für.	
Interview 27		Geltenes Recht ist, dann wenn die ID des Datensatzes, wenn die pseudonymisiert ist, dann gilt der Datensatz als Pseudonymisiert.	
Interview 27		Kann man überlegen, was man davon halten soll. Generell, Datensätze, wenn sie den Versorgungskontext verlassen, müssen pseudonymisiert sein.	
Interview 27		Wenn sie anonymisiert sind, dann kann man sie eben nicht mehr zurück auf den eigenen Patienten mappen, das gibt es auch in der Medizin. Diese Daten sind dann allerdings weniger gut, sind auch gut, aber weniger gut, weil man eben, ja, den Patient nicht mehr fragen kann.	
Interview 27		Viele Studien haben etwas mit Rekontakt der PAtienten zu tun. Die werden dann eben nach 5 Jahren Medikation gefragt: wie hat das Medikament denn gewirkt.	
Interview 27		Diese Sachen sind dann nicht möglich, weil man eben bei einem anonymisierten Datensatz nicht mehr auf das Individuum zurück kommt. Das sind glaube ich die wichtigsten,rechtlichen Aspekte.	
Interview 27		Dann gibt es hier die Europäische, sie GDP, die schreibt ja eigentlich genau vor, welche daten sind sensitiv, welche nicht, welche dürfen wie verwendet werden.	
Interview 27		Wenn man sich daran hält, ist alles gut. Dann gibt es eben die Probleme mit den Infrastrukturprovidern, die nicht in Deutschland sitzen. Und ich glaube, Das wird aberein Problem sein, das immer schlimmer wird in Deutschland.	
Interview 27		weil es immer weniger Infrastrukturprovider in Deutschland geben wird. Alles was die Bundesregierung zu stande bringt, wird nicht die Lösung des Problems sein, das ist leider viel zu langsam und vioel zu steinzeitlich, da muss irgendwann mal eine vernünftige Regelung geben.	
Interview 27		Es gibt ja den Trend quasi, dass die Amzaon und alle möglichen anderen, dass sie ihre Rechenzentren auch nach Deutschland packen - Frankfurt ist da so ein Hotspot.	
Interview 27	45	Da werden sie alle hingebaut, damit man dann sagen kann: "ja, meine Daten sind immerhin auf deutschen Boden.	

Interview 27	Wenn man mal etwas genauer in die Technologie guckt, dann arbeiten die auch über Datenreplikation über Standorte und dann ist das auch alles hinfällig.	
Interview 27	Außerdem ist der Vertrag außerdem mit einem amerikanischem Unternehmen und die NSA kann da immer drauf gucken.	
Interview 27		Gut, dann komme ich zu meinen letzten beiden Fragen: wir haben ja schon viel über Data Sharing gesprochen. Hast du denn Pläne, das weiter auszubauen?
Interview 27	Ja, natürlich, das ist mein Beruf. Das ist sicherlich eins der großen Ziele hier, das ich auch absolut vertrete. In zwei Wochen ist wieder der Retreat der medizinischen Fakultät.	
Interview 27	Da treffen sich die Dekane und der Dekan und die ganzen Professoren und Institutsdirektoren und da wird es auch wieder Thema sein.	
Interview 27	Es ist ein Thema, das kann man nur bewerben und dann hoffen, dass die Leute telnehmen. Also, auf jeden Fall.	
Interview 27		Das ist gut
Interview 27	Sharing ist die Zukunft. Und die anderen machen es.	
Interview 27		Was auch die Zukunft sein könnte, und das ist meine abschließende Frage: Ich habe ja eine Vorlesung zum Thema Künstliche Intelligenz und Big Data belegt. Mich würde jetzt noch interessieren, wie du den Einfluss von Big Data und Artificial Intelligence auf das Institut, auf dich, ...
Interview 27	auf die Welt - OK. Also es ist natürlch ein Thema, was ich auch von berufswegen schon lange verfolge. Wir haben am Anfang sogar schon bei IBM gesessen als sie mit Watson kamwn und sagen: hier, das kann alles.	
Interview 27	Inzwischen wissen wir, dass es nicht alles kann und das es auch sicherlich nicht alle Probleme lösen wird.	
Interview 27	Sagen wir mal: aus wissenschaftlicher Sicht finde ich das Problem, oder finde ich das Thema schwierig, weil die künstliche Intelligenz eine Black Box ist, wo ich viele Daten reinsteicken kann und dann kommt auch irgendwas raus, aber ich weiß nicht wie.	
Interview 27	Sondern es kommt einfach nur was raus. Und diese Black Box ist für einen wissenschaftler natürlich grausam.	
Interview 27	Und die Frage ist dann auch, replizieren muss man das ja auch irgendwie, das Finding.	
Interview 27	Und wie macht man das? Teilweise ist das wahrscheilich un,,öglich, das mit alternativen Technologien zu replizieren.	
Interview 27	Von der medizischen Ebene her, oder Versorgungsebene her, sind da glaube ich viele Chancen, da es viele Technologien gibt, die mit KI optimiert werden können - die dadrurch besser, schneller funtkionieren	
Interview 27	Gerade in der medizischen, gerade in der Medizintechnik, Geräte, profitieren davon. Ich glaube da, von der Ingenieursebene kommend kann man da viel machen. Und wofür die sicherlich auch gut geeignet sind, gerade der Bereich Big Data gut geeignet ist, Dinge zu finden, Pattern zu finden, die man einfach sonst nicht sehen würde.	

Interview 27		Sicherlich muss man dann auch hinterher hingehen und die Ergebnisse nochmal anders beleuchten. Schwierig wird es glaube ich immer dann, wenn nur mit KI zum Ergebnis X kommt.	
Interview 27		Weil man dann eben nicht belegen kann, wie es wirklich funktioniert.	
Interview 27		Aber eine Chance sehe ich da absolut. Es wurde in den letzten Jahren auch sehr gehypt als Thema, gerade von den Technoligekonzernen, IT-Konzernen. Am guten wissenschaften Arbeiten wird es nichts ändern, sondern das muss so bleiben.	
Interview 27		AI oder KI ist ein Tool für mich, finde ich absolut.	
Interview 27		Ob Deutschland nun da den Schwerpunkt drauf legen muss, wie das jetzt gerade passiert mit X Professuren und Schwerpunktthema Schleswig-Holstein KI weiß ich nicht, ob das so der richtige Angang ist, weil wir da auch schon sehr weit hinterher sind.	
Interview 27		Da gibt es andere Länder und Nationen, die uns da schon ein paar Jahrzehnte voraus sind, vor allem die Chinesen,	
Interview 27			Gut, hast du sonst noch was, was du mir zum Data Teilen oder Daten Sharing sagen möchtest?
Interview 27		Ich habe schon ziemlich viel gesagt.	
Interview 27			Muss auch nichts sein, aber vielleicht was, was noch nicht beleuchtet wurde?
Interview 27			Super, dann vielen Dank.
Interview 28	Time	Interviewee 28	Interviewer
Interview 28	0		Das Interview wird jetzt 15 offene Fragen beinhalten. Das heißt, Sie können einfach ganz offen drauf losreden - und erfahrungsgemäß eine dreiviertel Stunde.
Interview 28		Prima	
Interview 28			Haben Sie sonst irgendwas, was Sie von vornherein klären möchten?
Interview 28		Vielleicht läuft ab und zu eine Katze ins Bild, aber ansonsten ist alles in Ordnung.	
Interview 28			Ok, dann möchte ich Anfangen mit: was gehört denn zu Ihren täglichen Aufgaben?

Interview 28	Zunächst einmal bin ich Universitätsprofessor. Das heißt in der Vorlesungszeit bin ich mit Lehre beschäftigt in der Chemieinformatik, der analytischen Chemie und der Chemometrie. Ansonsten leite ich eine Arbeitsgruppe, die sich mit Chemieinformatik beschäftigt. also eine rein theoretische Arbeitsgruppe, zum Beisoiel, um beim Thema zu bleiben, die auf die Daten angewiesen sind, die andere Wissenschaftler produzieren. ICh bin sehr aktiv gewesen mein ganzes Leben lang im Bereich Infrastrukturaufbau für DAten. Das fing an mit kleinen Forschungsdatenbanken, die ich machen wollte und musste, weil es in der Chemie so wenige offene und freie Forschungsdaten gab und immer noch gibt. Und von dort bin ich dann mehr und mehr auch in große, globale und europäische Infrastrukturprojekte geraten und habe die am Ende auch geleitet. Zur Erstellung von Standards in der Wissenschaft, nicht nur in der Chemie, aner auch in den Biowissenschaften. Und eben auch für Infrastrukturprojekten, die dann mit den offenen Daten auch rechnen können. Meine ganze wissenschaftliche Arbeit beschäftigt sich damit, wissenschaftliches Material frei und offen zur Verfügung stellen zu können.	
Interview 28		In welchen Data Sharing Aktivitäten sind Sie denn involviert?
Interview 28	Ich habe, bevor ich nach (Ort) gekommen bin, zehn Jahre am europäischen Bioinformatikinstitut, die Abteilung Chemieinformatik und Metabolismus geleitet. Das EBI in Cambridge beschäftigt sich sozusagen hauptberuflich und im Auftrag seiner mittlerweile dreißig Mitgliedsstaaten damit, Daten, Biodaten, das reicht ja bis in die Chemie hinein, auch frei und offen zur Verfügung zu stellen in solchen Datenbanken.	
Interview 28	Und dort habe ich den Bereich Metabolismus aufgebaut. Da gibt es jetzt eine Chemiedatenbank Cheby und eine Metabolismusdatendatenbank Metebolits, die im Bereich im Bereich Metabolonics und Metabolismus und kleine Moleküle in der Biologie Daten frei und offen zur Verfügung stellt. Diese Daten werden bei Cheby kuratiert von, von, wie sagt man, Domainexperten sozusagen. Also Menschen, die das wirklich sitzen und Daten aus der Literatur kuratieren.	
Interview 28	Für Metabolonics ist das eine Datenbank, wo wir wirklich Forschungsdaten sammeln, die also von anderen bereitgestellt - freiwillig oder unter leichten Druck. Dadurh dass sie eben beispielsweise Paper publizieren wollen und die Instruktion für die Autoren, die sagen, sie anhalten oder sie sogar zwingen die Daten vor der Publikation in der Metabolits datenbank abzulegen.	
Interview 28	Ansonsten bin ich in einem SFB, leite ich das INF Projekt. Weiß nicht, ob sie damit vertraut sind. Die DFG hat irgendwann angefangen, für ihre Sonderforschungsbereiche sogennante INF Projekte zu	
Interview 28	5 bewilligen, die also mit der Informatik und den Informationen in den jeweligen Sonderforschungsbereichen zu tun haben und dafür sorgen sollen, dass die vernünftig gespeichert und archiviert werden und dann auch zur Verfügung stehen.	

Interview 28	Da gibt es in (Ort) den Sonderforschungsbereich Chembiosis, der sich mit Chemischen Interaktionen in Ökosystemen beschäftigt und da leite ich das INF Projekt, das eben genau mit diesem Data Sharing Aspekt zu tun. Dann haben wir ja, waren wir kürzlich erfolgreich mit einem Excellenzcluster in (Ort)	
Interview 28	das Bells of the Microverse. Da geht es auch eben um Interaktionen in Ökosystemen was die stört und was passiert wenn man die Stört und dort bin ich im Paket Data Synopsis	
Interview 28	federführend mittätig. Da geht es auch viel um Datenmanagement. Dieses Mal eben in einem wirklich großen Excellenzcluster. Ja und zu guter letzt, eigentlich gar nicht zuletzt, leite ich das (Name) Projekt, das (Name) Projekt, wo es eben um den Aufbau einer Nationalen Forschungsdateninfrastruktur in der Chemie geht.	
Interview 28	Und, naja, da wissen wir jetzt noch nicht, wie es weiter geht. Da findet die Begutachtung jetzt über den Jahreswechsel statt. Wir sind natürlich guten Mutes, aber wir wissen nie, was passiert. Wenn das wahr wird, dann wird das ein sehr aufregendes Projekt, das denke ich.	
Interview 28	Das Forschungsdatenmanagement in der Chemie in Deutschland und darüber hinaus auf jeden Fall revolutionieren wird. Und auf europäischer Ebene gibt es ja Projekte wie Elixir und GoFair, die sich ja auch mit Biodaten und Daten im allgemeinen beschäftigen. Und auch dort bin ich aktiv in verschiedenen Arbeitsgruppen um eben dafür zu sorgen, dass Forschungsdaten möglichst frei und offen zur Verfügung stehen.	
Interview 28		Nun haben Sie ja schon erwähnt, dass Sie auch Daten verwenden, die andere hochladen. Nun interessiert mich, inwieweit welche Daten von anderen bei der Forschung verwendet werdet.
Interview 28	Unser Forschungsinteresse liegt in der Strukturaufklärung von Naturstoffen und organischen Verbindungen - und zwar der automatischen Strukturaufklärung, wir sind ja Chemieinformatiker.	
Interview 28	Und da braucht man in vielen Bereichen eben Daten um Methoden zu validieren, also Algorithmen validieren oder eben auch um MEthoden des machinellen Lernens bis hin ins Deep learning zu trainieren und zu testen und zu entwickeln.	
Interview 28	Ein Beispiel wäre die Vorhersage von spektroskopischen DAten mit Hilfe von statistischen Methoden aber auch Neuronalen NEtzen. Da braucht man dann größere Datenbanken, in denen Strukturen von organischen Molekülen oder Naturstoffen und deren Spektren, Kernresonanzspektren typischerweise bei uns abgelegt sind.	
Interview 28	Und diese Datenbanken müssen kuratiert sein. Da geht es also darum, dass man für jedes Atom im Molekül auch weiß, welchen Kernresonanzshift das dann hat.	

Interview 28		Also es nützt nicht nur einfach das Spektrum und das Molekül abzulegen, es muss auch noch eine Zuordnung der Signale da sein. Also das sind aufwendige Datenbanken, die schwierig zu erstellen sind. Und mit solchen Datenbanken kann man dann eben Neuronale Netze zu trainieren um das Spektrum vorherzusagen. Und damit kann man dann am Ende Strukturaufklärung betreiben.	
Interview 28			Würden Sie denn noch gerne auf weitere Daten zugreifen?
Interview 28		Wir brauchen mehr und größere Daten von der gleichen Art in diesem Bereich. Es gibt andere Daten beispielsweise, das könnte man sozusagen als sekundäre Forschungsdaten bezeichnen, wir bräuchten Zugriff zu den Volltexten der kommerziellen Journale.	
Interview 28		In einer Art und Weise, dass wir sie machinell auswerten können. Das ist im Moment noch ein riesen Problem. In einigen europöischen Ländern gibt es Gesetzgebung, die das erlauben. In anderen europäischen nicht. In Deutschland bin ich mir da im Moment nicht so sicher, wo der Stand ist, da liest man verschiedene Dinge. Also es gibt diesen Slogan	
Interview 28		The ride to read ist the right to mine. Also wenn man es lesen kann, darf man das auch machinell auswerten. Aber die Verlage stehen da auf einem anderen Standpunkt, Ich würde also gerne auf die letzten 60 Jahre beispielsweise Journal of Natural Product der American Chemical Society, aber darf das eben in machineller Form nicht.	
Interview 28		Ich darf mich hinsetzen und als Mensch die einzeln lesen und kuratieren. Aber ich darf sie nicht machinell auswerten. Wenn ich das versuche, dann hat die ACS IT Systeme im Hintergrund, die solche Versuche detektieren und dann die ganze Universität radikal vom Zugriff auf dieses Journal oder auf gesamte ACS Journalangebot abschneiden - und das ist ein riesen Problem.	
Interview 28	10	Da scheint es aber, also ich glaube, es gibt im europöischen Rahmen eine Gesetzgebung, die das jetzt erlauben soll, aber ganz sicher bin ich mir da nicht. Und soweit ich weiß, brauchen die nationalen Gesetzgeber Zeit um diese eropäische Gesetzgebung in natinales Recht umzusetzen.	
Interview 28			Wie kommen Sie denn momentan an die geteilten Daten ran?
Interview 28		Naja, die Daten, die wir brauchen, die gibt es gar nicht. Da haben wir unsere eigene Datenbank mithilfe eben menschlichen Kuratoren erstellt. Eine kleine Kernresonanzdatenbank, NMR Shift DB Und die Daten, die wr gerne hätten, da sind gerade Bemühungen auch im Rahmen der NFDI4Chem in Gange, dass eben solche DAtenbanken für Kernresonanzspektren erzeugt werden.	

Interview 28	Aber das wäre dann ein paar Jahre in der Zukunft, dass sowas tatsächlich existiert. Also im Moment müssen wir die Daten selber manuell erstellen und das ist eigentlich, ich würde mal sagen, es ist wissenschaftlich ein Desaster, weil diese Daten seit 50 - 60 Jahren publiziert werden, aber eben in dieser zerstörten Form als gedruckte Information auf Papier und nicht in machinen lesbarer Form. Und dann müssen wir sie mühsam aus der Literatur zurück holen.	
Interview 28		Dann haben Sie jetzt schon die Zukunft erwähnt. Ich würde gerne in ein Zukunftsszenaro gehen, inde ich Sie frage: wie stellen Sie sich in Zukunft den idealen Zugriff auf die benötigten Daten vor?
Interview 28	Der ideale Zugriff ist frei und offen über Webdatenbanken basierend auf Qualitätsstandards, die die gesamte Community festlegen muss, diese Daten eben zur Verfüfung stellen.	
Interview 28	Das heißt, man braucht akzeptierte, offene Datenbanken.	
Interview 28	Die Wissenschaftler, wie sagt man, mit sanftem Druck dazu gebracht werden, dass sie ihre Daten dort ablegen. Da gibt es verschiedee Mechanismen.	
Interview 28	Und man braucht offene Standards.	
Interview 28	Das sind Datenstandards, mit denen man beispielsweise, ich bleibe mal bei den Kernresonanzspektren, die die Primärdaten darstellen kann, dass man von den Herstellerformaten unabhängig ist.	
Interview 28	Man braucht aber auch, in deutschem würde man vielleicht sagen Minimalinformationsstandard, also in welcher Form diese Daten auch mit Metadaten beschrieben werden.	
Interview 28	Da hängt noch eine ganze Menge an sozialen Fragen dahinter, wie man zu dieser Situation kommt, aber auf der technischen Ebenen lässt es sich relativ leicht lösen. Dann hängen da eben noch die Standards mit dran, die brauchen Zeit um die zu entwickeln. Da sind die Biowissenschaften deutlich weiter als die Chemie.	
Interview 28	Die brauchen deshalb Zeit, weil man sich eben nicht als Arbeitsgruppe hinsetzten kann und kann so ein Standard entwickeln. Sondern dafür muss man wirklich weltweit Experten in einen Raum bringen, die dann darauf einigen, was sind, was ist so die Minimalinformation, die man zu einem Datensatz ablegen muss. Wenn man das alleine macht, dann adobtiert das keiner.	
Interview 28	Deswegen sind das komplexe, internatonale Prozesse, die man da anstoßen muss. Und wir rechnen zum Beispiel ihm Rahmen der NFDI4Chem mit fünf bis zehn Jahren um sowas dann wirklich zu etablieren.	
Interview 28		Welche konkreten Anforderungen stellen Sie an die geteilten Daten?
Interview 28	Das sie offen sind, ist für mich das wichtigste.	
Interview 28	Da gibt es natürlich Einschränkungen. Also Patientendaten beispielsweise, da gibt es gesetzliche Regelungen um die Privatsphäre der Patienten zu schützen - an die muss man sich halten, da gibt es gar keine Frage.	
Interview 28	Dann gibt es ja dieses Akronym FAIR, über das sie sicherlich schon viel gestolptert sind.	

Interview 28		Daten müssen FAIR sein, also auffindbar, zugänglich, interoperable und wiederverwendbar und reproduzierbar. Also, das sind so die Grundlagen. FAIR alleine reicht mir nicht, weil FAIR nicht notwendigerweise bedeutet, dass sie offen sind. Man kann Daten auch hinter IT Mauern verstecken und trotzdem wären sie nach den FAIR Prinzipien, wären sie eben fair.	
Interview 28		Ansonsten müssen Sie gut - wenn Sie mit. Wenn offene Daten mit den FAIR Prinzipien übereinstimmen, dann sind eigentlich alle anderen Anforderungen auch schon gelöst.	
Interview 28		Also die Kodierung in offenen Standards und die übereinstimmung der Metadaten mit Minimalinformationsstandards, auf die sich die Kommunity geeinigt hat.	
Interview 28	15	Wenn das der Fall ist, dann hat man eigentlich ein Ökosystem von offenen Daten, mit denen man wirklich was anfangen kann	
Interview 28			Nun haben Sie ja gesagt, dass offen eine der Hauptgründe ist, Daten zu teilen. Welche Beweggründe stehen dahinter?
Interview 28		Offene Daten oder das Teilen von Daten ist einfach Teil der wissenschaftlichen Methode. Wir bauen auf den Erkenntnissen anderer auf und damit die wissenschafltiche Methode in ihrer Gänze funktioniert, müssen wir in der Lage sein zum einen die Ergebnisse anderer auch validieren können. das ist bekannt, dass es auch eine Validierungskrise gibt.	
Interview 28		Auch und vor allem in hoch angesehen Studien, die eben publiziert wurden, die dann nicht validiert werden konnten. Und zum anderen können wir eben mit den Daten, die andere publizieren, Wissenschaft betreiben, auf die die vielleicht nicht gekommen wären.	
Interview 28		Zum einen kann man einfach mit einem einzelnen Datensatz, den jemand publiziert, vielleicht etwas machen, dass dem eben nicht eingefallen ist.	
Interview 28		Man kann aber auch Datenseätze von mehreren Zusammentragen und dann Metastudien, Metaanalysen machen, die ansonsten die Arbeitsgruppe, die jetzt diesen einen Datensatz erzeugt hat auch nicht machen konnte, weil sie eben die Gesamtheit der Daten nicht zur Vergüung hatte. Ein Beispiel: vor 2, 3 Jahren gab es das Paper über das menschliche Proteom, also die Gesamtheit aller Proteine im menschlichen Organismus.	
Interview 28		Das war eine Arbeit, die nur möglich war, weil es eben zehn Jahre lang, die PRIDE Datenbank am europäischen Bioinformatikinstitut gab, die über zehn Jahre hinweg Proteomdaten von allen mölichen Studien gesammelt hat und eine Metaanalyse all dieser Daten hat dann ermögkicht, dass man dieses große Paper über das menschliche Geom schreibt. Da werden also Dinge möglich, die für eine einzelne Gruppe oder für kleine Gruppen nicht möglich sind, die also sozusagen weltweite Erfordern im Datenmanagement um so ein Paper dann am Ende schreiben zu können.	
Interview 28			Welche Probleme und Schwierigkeiten treten denn beim Data Sharing auf?

Interview 28		Es gibt natürlich technische Probleme, die auftreten. Die sind, die liegen meiner Ansicht nach vorwiegend in der Art und Weise wie wir Daten im Labor managen. Das ist alles noch sehr frühes 20. Jahrundert, spätes 19. Jahrhundert, wir schreiben Dinge mit einem Stift in Laborbücher und ansonsten liegen unsere elektronischen Daten an allen möglichen Stellen.	
Interview 28		Und wenn es dann zu dem Punkt kommt, dass wir etwas publizieren müssen, dann müssen wir oft Dinge vielleicht wieder abtippen oder wr kopieren ein NMR Spektrum von einem Ausdruck, den wir irgendwo rumliegen haben.	
Interview 28		Und dann müssen wir das ganze zum einen in die Form bringen, die das Journal wünsch.	
Interview 28		Und zum anderen, wenn das Journal sagt, bitte legt eure Daten auf einer Datenbank ab, dann muss sich der arme Student oder der arme Postdoc noch hinsetzten und das Ganze in diese elektronische Form wieder bringen, in der es ursprünglich mal war, aber die unter Umständen irgendwo verloren gegangen ist auf dem Weg vom sagen wir mal Spektrometer ins Institut bis jetzt zu dem Punkt wo die Daten in ein offenes Format gebracht werden müssen.	
Interview 28		Und an der Stelle streiken die meisten Wissenschaftler. Man kann sagen, vielleicht aus gutem Grund. Dieses System ist eben so zerbrochen und so kaputt, dass es für den einzelnen Wissenschaftler, der ja vorankommen möchte, der dann vielleicht seine nächste Stelle antreten möchte nachdem er das Paper geschrieben hat,	
Interview 28		das ist einfach zu aufwendig ist, in dieses Data Sharing sich zu engagieren. Man kann das dadurch lösen, dass man die, das akademische, das frühe akademische Data handling oder überhaupt die Arbeitsweise in akademischen Laboren dem anpasst, was die Industrie schon lange macht.	
Interview 28		Das man eben elektronische Laborbücher, elektroschine Laborinformationsmanagement und -datenbanksysteme etabliert, die es dann dem Forscher ermöglichen, gleich alle Dinge in elektronischer Form und wohl annotiert und mit guten Metadaten zu erfassen. Dann ist es am Ende nur noch ein Klick um beispielsweise das experimentelle, die experiemellte Sektion in dem Artikel zu schreiben.	
Interview 28	20	Das muss man dann nicht mehr schreiben, sondern ELN das elektronische Laborbuch ELN exportiert das automatisch. Und ein weiter Klick lädt den ganzen Kram dann in eine Datenbank hoch.	
Interview 28		Und nochmal: um das zu ermöglichen braucht es eben internationale Bemühungen um diese Standards zu erzeugen, indem diese Daten erstellt werden.	
Interview 28		Und wenn diese Hürden überwunden werden, dann gibt es am Ende noch die sozialen Hürden, die sind sehr schön zusammen gefasst - hatte ich Ihnen da dieses Paper geschickt über die Hürden des Data Sharings?	
Interview 28			Von Patientendaten

Interview 28	Es gab was, das waren keine. Es gibt im Journal F1000 gibt es ein schönes Paper, warum, was die sozialen Hürden sind vom Data Sharing. Das fängt also an: das wichtigste ist eigentlich, dass Menschen oder Wissenschaftler Angst haben - wie ist das deutsche Wort? - also im englischen heißt es scoop, dass sie sozusagen von anderen mit ihren eigenen Daten übertrumpft werden.	
Interview 28	Das ist so die Angst, ich teile meine Daten, der andere nimmt sie, analysiert sie schneller als ich und schreibt vor mir das Nature Paper.	
Interview 28	So eine sehr irrationale und dumme Angst, aber es ist die weit verbreiteste Angst. Und dann natürlich die üblichen Dinge, die an der menschlichen Natur so hängen, dass man gerne alles für sich behalten möchte um dann Vorteile anderen gegenüber bekommen. All diese Dinge spielen eine Rolle und sind auch für Data Sharing.	
Interview 28		Sehen Sie denn auch finanzielle Aspekte, die beim Data Sharing einspielen?
Interview 28	Also ich habe jetzt von Kollegen gehört, dass sie die Vision, die wir so haben, nicht notwendigerweise unterstützen könne, weil sie sagen, sie bräuchten eine Stelle im Labor, die sich nur mit den Aspekten des Data Sharings befasst und die dann den Rest der Gruppe, dem Rest der GRuppe ermöglichen in dieses moderne System des Data Sharings sich zu engagieren.	
Interview 28	Das ist vielleicht für eine Übergangszeit sogar wahr, aber in anderen Bereichen der Wissenschaft, andere Bereiche sind da schon weiter. Ich glaube wir können diese Situation relativ schnell auch in der Chemie übergehen.	
Interview 28	Im Moment steckt die Bundesregierung 85 Millionen jedes Jahr in den Aufbau der NFDI oder wird, wird 85 Millionen hinein stecken. Das alleine zeigt, dass man da eine Menge Geld investieren muss um zu einer guten Situation zu kommen. Aber wenn die erstmal etabliert ist, dann glaube ich, ist das relativ kostengünstig und gehört zum normalen Laboralltag.	
Interview 28		Wird denn das Teilen von Daten anerkannt in der wissenschaftlichen Community?
Interview 28	Das hängt sehr stark davon ab, in welcher Community man ist. In der Chemie habe ich das Gefühl, dass es so kleine, es gibt eine Bewegung, die dem sehr viel Gewicht beimisst.	
Interview 28	Also, weiß nicht ob sie vom Blue Obelisk gehört haben. Da gibt es einge Gruppe, die sich seit den frühen neunzigern Daten, Software, und Standards frei und offen zu machen, denen ist das natürlich sehr wichtig. Aber die meisten, den meisten ist es glaube ich tatsächlich sehr egal.	
Interview 28	Ist meine eigene Meinung. Das bringen die auch zum Ausdruck, also. Genickt wird immer wenn gesagt wird: ja, Daten müssen frei und offen geteilt werden, weil man dann bessere	
Interview 28	Wissenschaft macht. Aber, das ist, gibt es ein Lippenbekenntnis.	

Interview 28		Viele handeln anders. Da muss eben ein sanfter Zwang von Journalen und vor allem von Geldgebern da sein um - und dass muss auch später überprüft werden.	
Interview 28	25	Data Sharing Agreements schreibt heute jeder in seinen Antrag rein, seinen Forschungsantrag, aber das sind Lippenbekenntnisse. Getan wird dann am Ende doch nichts.	
Interview 28			Darauf aufbauen stellt sich mir die Frage, inwieweit rechtliche Aspekte das Data Sharing beeinflussen können?
Interview 28		Dass, der, meiner Ansicht nach der einzige Bereich, im akademischen Bereich, der rechtliche Aspekt. Wichtig sind, ist das Teilen von Patientendaten, weil die eben sensitiv sind und da gibt es eine klare Gesetzgebung.	
Interview 28		Generell eigentlich muss man sagen, vielleicht nicht nur Patientendaten sondern Daten, in denen menschliche Subjekte beim, in einer Studie beispielsweise beteiligt sind. Deren Daten müssen geschützt werden und die Art und Weise, wie das verarbeitet wird, muss in Einklang stehen mit dem, was die Probanden, ist vielleicht das bessere Wort, was die Probanden als Einwilligung gegeben haben.	
Interview 28		Da können sich Leute natürlich auch auf den Standpunkt stellen: ich möchte in der Lage sein, meine Forschung zu patentieren. Auch da spielt es sicherlich eine Rolle, wie diese Daten vor der Veröffentlichung gehandhabt werden. Das ist etwas, was mir persönlich nicht wichtig ist.	
Interview 28		Ich stehe immer auf dem Standpunkt, die Forschung wird vom Steuerzahler gezahlt und sollte dann auch allen frei und offen zur Verfügung stehen.	
Interview 28		Aber der Gesetzgeber möchte, oder, unsere Politik möchte ja auch, dass unsere Universitäten Intellectual Property generieren und Dinge patentieren.	
Interview 28		Und in diesen Bereichen spielen rechtliche Aspekte dann wahrscheinlich auch eine Rolle.	
Interview 28			Nun sind Sie ja Professor und im Managementbereich. Haben Sie dadurch auch Einfluss auf Data Sharing?
Interview 28		Dadurch, dass wir, also in meiner Gruppe wird natürlich alles, was wir machen, frei und offen geteilt. Das ist schon seit langer Zeit so. Die Software, die wir produzieren, sind ja im Grunde auch Forschungsdaten, weil wir eben theoretisch Daten.	
Interview 28		Und die Daten, die wir verwenden und bekommen sind auch immer alle offen, da legen wir großen Wert drauf. Und ich kann natürlich im Rahmen der Projekte, die ich leite, auch Menschen dazu anhalten ihre Daten doch frei und offen zu teilen. Das ist auch durchaus gelegentlich von Erfolg gekrönt.	
Interview 28		Aber der Einfluss hört dann eben da auf, wo andere PIs ihre Gruppen vorschreiben, dass ihre Daten eben nicht frei und offen sein sollen.	
Interview 28			Nun haben Sie gesagt, dass Sie Daten auch teilen. Haben Sie schon gesagt, worüber Sie das machen? Läuft das über Datenbanken oder wie teilen Sie die Daten?

Interview 28		Die, genau. Wenn wir richtige Forschungsdaten haben, also beispielsweise Metabolomdaten, dann laden wir die in Datenbanken hoch.	
Interview 28		Unsere anderen Ergebnisse werden über offene Codesharing Platformen wie Github, Sourceforce und andere, Gitlab, geteilt. Das sind so offene Communityportale, in denen beispielsweise Softwareentwickler ihren Quellcode teilen, im Grunde die Softwareentwicklungs, das Softwareentwicklungsanalogon zur freien und offenen Forschungsdatenbank. Dort teilen wir unsere Daten.	
Interview 28			Nun haben wir ja jetzt schon über einige Probleme und Barrieren gesprochen. Welche der genannten ist für Sie die größte Barriere beim Data Sharing?
Interview 28		Der soziale Aspekt. Die menschliche Komponente. Das dauert am längsten.	
Interview 28		Menschen ändern sich nur langsam und man muss ja auch immer berücksichtigen, der einzelne agiert in einem sozialen Ökosystem, man könnte auch soziale Blase nennen, und werden in solchen sozialen Blasen alle - oder wenn der einzelne glaubt, ich spreche jetzt von Forschungsgruppenleitern, weil die ihre Gruppe ja beeinflussen, wenn der einzelne glaubt, in seiner sozialen Blase nicht üblich ist und alle eigentlich gegen Data Sharing sind, dann wird sich das da nur sehr, sehr langsam ändern. Ich habe diesen schönen Spruch, der ist nicht von mir,	
Interview 28		das Fortschritt in der Wissenschaft eine Verentung nach der anderen erfolgt. Also eine Ruhestand nach dem anderen und man muss dann sehr lange warten, bis die alten Herren und Damen aus der Wissenschaft verschwunden sind, und dann ändert sich langsam was, wenn neue, neuer Geist nachströmt.	
Interview 28		Was ich auch bemerke, ist eben, dass diese Dinge aufgrund dessen sehr langsam passieren, aber es tut sich was. Ich habe vor dreißig Jahren angefangen und gleich von Anfang an die offene Attitude mit befreundeten Wissenschaftlern verfolgt.	
Interview 28	30	Und wir waren am Anfang natürlich sehr optimistisch, dass wir da sehr schnell was ändern können, aber es tut sich alles nur sehr langsam was, aber es ändert sich was. Die Welt wird, was das angeht, besser.	
Interview 28			Inwieweit möchten Sie denn Data sharing Aktivitäten ausbauen?
Interview 28		Ja, also mehr ausbauen kann ich Sie eigentlich gar nicht. Ich verwende fast mein gesamtes wissenschaftliches Leben darauf, da in dieser Hinsicht die Welt besser zu machen. Und ich glaube, wir sind da auch schon wirklich weit gekommen.	
Interview 28			Nun haben Sie am Anfang von Machine Learning Algorithmen gesprochen. (…) Deswegen ist für mich auch eine interessante Frage: inwieweit schätzen sie den Einfluss von Big Data und künstliche Intelligenz auf ihren Forschungsalltag ein?

Interview 28		Der ist potentiell gigantisch. Im Moment sind wir so ein bisschen begrenzt hinsichtlich der Rechenkapazität, die wir verwenden können. Wir haben im Moment ein interessantes Problem, wo wir versuchen tiefe neuronale Netze dazu zu bringen, die chemischen Daten aus Pubklikation wieder in elektronische Form zu überführen.	
Interview 28		Also im Grunde eine absurde Sache. Zuerst zerstören wir die Information, weil wir sie auf Papier drucken, dann wollen wir sie wieder rausholen, aber so ist die Situation nun. Wir haben nun 60 Jahre mindestens gute Information in diesen gedruckten Journalen, und die Originaldaten sind alle längst weg.	
Interview 28		Und das Problem ist, wir müssten - was haben wir im Moment ausgerechnet? - wir müssten also sechs Monate trainieren, so ein neuronales Netzt auf den Trainingsdaten, die wir haben, um zum Erfolg zu kommen. Und das würde auf den - das ist sehr teure Hardware, mit denen man das trainieren muss.	
Interview 28		Das würde also hunderte von tausenden von Euro kosten, Und man weiß dann auch nach sechs Monaten nicht, ob der Run erfolgreich ist.	
Interview 28		Unser Problem ist mehr im Bereich der Rechenkapazität, die nicht zur Verfügung steht. Ansonsten ist es nicht abschätzbar im Moment. Also man kann es nicht hoch genug einschätzen, welchen enormen Einfluss die Deep Learning auf die Wissenschaft haben wird.	
Interview 28			Ja, interessant. Danke für Ihren Input.
Interview 28			Dann bin ich auch schon durch mit all den Fragen. Haben Sie sonst noch etwas Sie mir mitgeben möchten zum Thema Data Sharing, was Ihnen wichtig ist?
Interview 28		Oh, ich glaube, ich habe alles gesagt, was ich sagen wollte. Ich wäre auf jeden Fall sehr interessiert daran, Ihre Arbeit zu sehen, wenn das möglich wäre. Und ansonsten wünsche ich Ihnen viel Glück beim Schreiben und beim Auswerten.	
Interview 29	Time	Interviewee 29	Interviewer
Interview 29	0		Das erste, was mich interessiert, ist: was gehört zu Ihren täglichen Aufgaben?
Interview 29		Was gehört zu meinen täglichen Aufgaben? Also ich bin der Leiter dieses Zentrums (…) Forschungsdatenmanagements. Deswegen gehört jetzt zu meinen täglichen Aufgaben, natürlich auch die Koorderdination all der Arbeiten in diesem Zentrum.	
Interview 29		Also neben auch solchen Dingen wie Personalgeschichten, und Plaung und so weiter. Aber letzlich die Hauptaufgabe besteht darin, dass ich die verschiedenen Fäden zwischen den verschiedenen Terminen zusammenhalte und dafür sorge, dass auch in der Universität diese Dienstleistungen bekannt werden.	
Interview 29		Und jetzt nicht einfach nur Werbung machen, sondern auch versuche insbesondere in strategischen großen Projekten, im Bereich der Forschung, zumindest so weit mitzumachen, dass dort darüber informiere, was Datenmanagement bedeutet, was man tun muss, dass sie das vieleicht nicht unbedingt bei uns machen müssen sondern dass es mehr um das generelle geht, dass man das überhaupt machen.	

Interview 29	Also ich bin nicht dogmatisch, dass ich sage, sie müssen das bei uns machen und sonst gilt das nicht. Sondern ich sage, sie müssen das überhaupt machen undzwar da, wo es für sie am besten ist. Das ist glaube ich das wichtigste. Das ist also insofern, also so eine wirklich typische Aufgabe, wo ich jetzt sagen könnte, jeden Tag bin ich so und so viele Stunden mit beschäftigt, das habe ich eigentlich gar nicht das.	
Interview 29	Das sind entweder ganz viele Gespräche, die ich führe, Anträge schreiben oder Anträge mitschreiben, an Gremiumsitzungen an der Universität teilnehmen auch in der Stadt an entsprechenden Sitzungen teilnehmen, die sich um dieses diesen Themenkreis letzlich drehen.	
Interview 29	Also es eigentlich ein sehr bunter Strauß [lacht]	
Interview 29		Und in welchen Data Sharing Aktivitäten sind sie dann beteiligt?
Interview 29	Also, bei uns geht es ja hauptsächlich nicht um Data sharing, es geht - also das ist natürlich auch ein Gebiet, aber das ist jetzt nicht so, dass das Ziel ist, dass wir nur Daten nehmen, die dann auch geshared werden soll oder können.	
Interview 29	Also erstes Ziel ist es, dass alle Daten die bei einem Forschungsprojekt entstehen, und die auch aufbewahrungswert sind, also nicht alle Daten muss man aufbewahren, aber, sagen wir mal: ihre Forschungsdaten wären beispielsweise diese Interviews.	
Interview 29	Und diese Interviews sollten Sie eine gewisse Zeit aufbewahren, falls jemand sagt, ach Sie haben sich das Ganze nur ausgedacht, ne ist klar. Das heißt aber nicht unbedingt, dass sie diese Daten sofort mit der ganzen Welt sharen und weitergeben würden, sondern möglicherweise würden sie sie weitergeben an jemanden, der Interesse daran hat.	
Interview 29	Aber sie würden die nicht gleich im Internet zum Sharing bereitstellen. Das gilt tatsächlich für viele Forschungsdaten. Also insbesondere muss man vielleicht so grundsätzlich unterscheiden.	
Interview 29	Im Bereich der Naturwissenschaften ist es immer einfacher, weil dort handelt es sich hauptsächlich um Messdaten, die jemand selber gemessen hat, und die kann er auch, da kann er selber entscheiden, werden due auch weitergeben.	
Interview 29	Es gibt Bereiche wie Klimaforschung oder Astronomie, da ist es sogar üblich quasi sein Daten in dem Moment, in dem man sie gemessen hat, zur Verfüfung stellt, damit andere damit auch was machen.	
Interview 29	Es gibt aber viele Bereiche im Bereich einer Universität, wo das nicht so einfach ist - also Psychology, Medizin beispielsweise. Wo Sie dann auch mit, mit Patientendaten und personenbezogenen Daten zu tun haben.	
Interview 29	Da können Sie nicht einfach diese Daten frei und offen zur Verfügung stellen und damit sharen. Das verbietet sich einfach. Es ist aber trotzdem wichtig, diese Daten zu behalten.	

Interview 29		Und das Sharing besteht dann darin, dass möglicherweise jemand anderes sagt: "ich will etwas ähnliches machen - da gibt es ja schon eine Studie". Dann muss ich halt fragen um, ob ich diese Daten benutzen kann.	
Interview 29		Das ist der Gedanke dabei. Weil Sharing, das ist jetzt die Frage, wie Sie das definieren, aber so wie ich es immer verstehe, wird ja eigentlich als: jeder kann alles frei verfügbar machen, ja.	
Interview 29			
Interview 29		Also, beispielsweise Netflix würde man nicht als Sharing Dienst bezeichnen, weil man	
Interview 29		ja dafür bezhalen muss, dass man das benutzen kann. Aber irgendwie so ein illegaler Filehoster, wo man die neusten Spielfilme runterladen kann, da würden alle sofort sagen, das ist ein Sharing dienst.	
Interview 29	5	Aber das ist eigentlich damit ja nicht gemeint. Es ist eigentlich nur gemeint, dass ich Daten austauschen kann. Die Bedinungen muss ich vielleicht aushandeln.	
Interview 29			Das heißt, inwieweit werden dann geteilte Daten von anderen in der Forschung verwendet, wissen Sie das?
Interview 29		Also wirklich wissen tue ich es im Moment nicht. Ich weiß aber, dass es viel passiert. Und das ist der erste Schritt, ist im Grunde genommen, dass man die Daten zur Verf - oder Informationen, dass es so einen Datensatz gibt zur Verfügung steht. Daten selber dann ja auch irgendwo gespeichert hat, aber das jemand das überhaupt finden kann.	
Interview 29		Dann kann jemand sagen: ok, da gibt es vielleicht doch was", findet es hoffentlich und kann entweder darauf zugreifen oder ich möchte gerne, darf ich das bentuzen?	
Interview 29		DAs passiert in vielen Bereichen, also Astronomie passiert das, in den Klimawissenschaften passiert es, überhaupt im Bereich der Naturwissenschaft passiert es sehr häugig schon, in den anderen Wissenschaften eher weniger.	
Interview 29		Da hat man eher so: das sind meine Daten, das ist mein geistiges Eigentum, ich gebe das nicht her. Also das ist jetzt sehr vereinfacht gesprochen.	
Interview 29		Aber, ja. Es gibt immer Ausnahmen in alle Richtungen, ganz klar. Wenn ich in der Chemie etwas entwickle, was vielleicht patentverdächtigt. Also aktuell einen neuen Kraftstoff für Autos oder sowas.	
Interview 29		Dann wäre ich, wäre es natürlich dumm: ja kann jeder haben. Ne, ich meine, das ist klar, solche Daten würde ich erstmal zurückhalten und dann villeicht ein Patent anmelden und so weiter.	
Interview 29		Da muss man dann sehr unterscheiden. Das ist glaube ich ein sehr wichtiger Punkt, dass man nicht von Forschenden verlangt, alles frei und offen zur Verfügung stellen müssen.	
Interview 29		Oder alles mit allen teilen müssen, sondern sie müssen halt mal hingucken, was sie machen. Es ist allerdings so, dass das ein Trend ist, dass das ein Trend ist, dass man schon vorhandene Daten benutzt.	

Interview 29	Entweder weil es sehr teuer ist, Dinge zu erzeugen, oder weil man damit möglicherweise auch Experimente spart. Typisches Beispiel sind zum Beispiel Tierversuche, wenn man (.) Medikamente testen will, braucht man in der Regel Tierversuche.	
Interview 29	Wenn man jetzt aber zum Beispiel sagt, dieses Medikament hilft gegen irgendeine bestimmte Krankheit und man hat dann schon Tierversuche gemacht und es ist rausgekommen: ne, das funktioniert nicht, das hilft überhaupt nicht, ja.	
Interview 29	Nach drei Jahren kommt jemand anders auf die Idee, dieses Medikament könnte doch gegen die Krankheit helfen, dann muss er das ja nicht nochmal machen.	
Interview 29	Das ist ein ganz wichtiger Punkt, dass man möglicherweise auch negative Ergebnisse, also in dem Sinne da ist gar nicht bei rausgekommen, veröffentlicht, sodass man das auch wieder benutzen kann.	
Interview 29	Das wird auch schon gemacht. Damit werden auch teilweise schon Experimente eingespart, also gerade so im Bereich Tierversuche beispielsweise.	
Interview 29	Aber auch in anderen Bereichen. Also in der Astronomie wird das auf jeden Fall sehr intensiv benutzt.	
Interview 29	Und in einigen anderen Bereichen auch. Also das interesse steigt, Angebot (-) folgt auf Nachfrage [lacht]	
Interview 29		Und wie kommen die dann momentan an die Daten ran?
Interview 29	Also wir haben das Prinzip der Freiwlligkeit. Es ist so, dass wir eine Plattform bereitstellen, wo jeder selber seine Daten hochladen kann, dementsprechend beschreiben kann und selber auch entscheiden kann, sind die offen oder sind sie geschlossen.	
Interview 29	Das ist also in der Hand derjenigen, die das machen. Das ist das, was ich vorhin meinte, man sollte das beste Tool für sich suchen.	
Interview 29	Es gibt in bestimmten Bereichen gibt es schon sehr gute, überregionale agierende Anbieter für soetwas. Also wieder Beispiel Klimaforschung: hier in (Ort) haben wir das (Ort) Klimarechenzentrum.	
Interview 29	Da macht es überhaupt keinen Sinn, wenn die Leute das bei uns im Archiv speichern. Da würde ich immer sagen, nein, sie sollen es lieber dort speichern.	
Interview 29	Es gibt aber viele Bereiche in der Wissenschaft, wo es sowas gar nicht gibt. Mh Da macht es dann wieder Sinn, dass Sie das bei uns speichern - also wichtig ist nur, dass sie es speichern.	
Interview 29	Und die. Letzlich ist es freiwillig, Der Druck, der da ein bisschen entsteht, ist dadurch, dass die Drittmittelgeber, sobald sie Geld geben, verlangen, dass die Leute das machen.	
Interview 29	Mh Man muss es eigentlich für zehn Jahre nach Projektende aufbewahren. Man vertraut im Moment noch darauf, wenn die Leute sagen: "ja ich mache das", dass sie das auch machen. Also es wird kein Zertifikat gefordert beispielsweise.	
Interview 29	Das könnte man ja auch machen. Man könnte sagen: ja ja, wenn du es irgendwo gespiechert hast, dann muss dir die Stelle, wo du es gespeichert hast, einen Nachweis geben.	

Interview 29		Möglicherweise kommt das noch. Also, Was schon teilweise gemacht wird, dass man im Abschlussberericht ein, einen Link oder Identifier anbieten muss, wie man denn auf die Daten zugreifen kann.	
Interview 29		Das ist ja schon. Dann könnte halt derjenige, der den Abschlussbericht liest, Drittmittelgeber, guckt dann mal nach, klappt das überhaupt,und dann ist das in Ordnung.	
Interview 29		Wenn es den Link dann gar nicht gibt, gibt es vielleicht Ärger. Also, der Druck steigt da schon, insofern ist es vielleicht ein selbstverstärkender Prozess. (-)	
Interview 29	10	Aber mehr können wir im Moment auch nicht machen. Freiheit der Forschung führt eben auch dazu, dass man leider keine Verpflichtung im dem Sinne einbauen kann, also keine gesetzliche Verpflichtung: sie müssen das speichern, und sie müssen das dort machen, ja (-) wenn nicht, dann passiert irgendwas.	
Interview 29			Wie stellen Sie sich denn in Zukunft den idealen Zugriff auf benötigte Daten vor?
Interview 29		Also auf jeden Fall natürlich über das Internet, klar. Der ideale Zugriff ist für mich, dass ich in jedem Fall erstmal die Daten finden kann. Das heißt, dass die Daten so geschrieben sind mit Metadaten, dass man sie finden, wiederfindet.	
Interview 29		Ob man dafür noch spezielle Suchmachinen braucht, die das machen, das ist (-) kann man lange drüber reden. Google macht das ja wahrscheinlich gar nicht so schlecht, aber da ist natürlich nicht sehr speziell, wobei Google da gerade dabei ist, so ein Dataset Search zu entwickeln, der eigenetlich genau das wahrscheinlich dann am Ende des Tages macht.	
Interview 29		Also das ist das wichtigste überhaupt. (-) Dann natürlich, dass die Leute es ablegen, speichern, entsprechend beschreiben sodass man das dann wiederfinden kann, und dann natürlich auch, wenn sie etwas machen, vielleicht bevor sie selber anfangen, erstmal gucken, was gibt es denn schon.	
Interview 29		Wie es ja in der Natur üblich ist. Also man macht immer erstmal so ein Check, was gibt es so an Literatur - also Stand der Forschung. Könnte man ja auch noch einbauen (-) Stand der Daten sozusagen, welche Daten gibt es schon, was kann ich villeicht benutzen, was kann ich selber noch dazu machen?	
Interview 29		Vielleicht ich ja auch einen Datensatz benutzen und ihn ergänzen. Mh Und so weiter.	
Interview 29		Vielleicht haben sie auch Ideen darüber, wie andere vor ihnen damit umgegangen sind oder welche Daten sie genutzt haben, was ich dann damit ja machen kann.	
Interview 29		Ja also sowas kann auch Ideengenerierung, das ist ja vielleicht auch noch eine Aufgabe.	
Interview 29		Also, die technische Seite ist glaube ich komplett gelöst. Also, da gibt es nicht wirklich viel zu etnwicklen. Ich glaube, es ist wieder ein Verhaltensproblem der Menschen, aber auch das wird sich ändern.	
Interview 29		So wie wir uns an Google gewöhnt haben und an viele andere tolle Dinge [lacht] werden wir uns auch daran gewöhnen und es wird zunehmend ein Prozess werden, der so in den Alltag übergeht.	
Interview 29		Und wir werden einfach viel viel XX andere Dinge benutzen	

Interview 29		Welche Anforderungen stellen Sie denn an die Daten?
Interview 29	Also sie müssen in jedem Fall (-) richtig sein. Das kann man natürlich quasi nicht überprüfen [lacht] und vor allen Dingen, haben wir, ja - was heißt auch richtig? Das ist schon wieder eine große Frage.	
Interview 29	Das wichtigste ist überhaupt, dass die Daten so geschrieben sind, dass auch jemand anderes etwas damit anfangen kann. Also ein schönes Beispiel, also ein Text ist relativ einfach.	
Interview 29	Also wenn ich eine PDF Datei hochlade, dann muss ich nicht noch eine Beschreibung machen, das ist eine PDF Datei mit einem Text, also das wäre jetzt Unsinn, weil das ergibt sich sofort. Wenn ich jetzt aber einfach irgendwelche Messungen habe, dann brauche ich ja schon Informationen: was ist das denn überhaupt, was sagen diese Werte aus, in welchen Einheiten sind die, wo sind die gemessen, womit sind die gemessen.	
Interview 29	Das macht ja ein riesen Unterschied (-) Temperaturmessungen ob ich die mit einem Küchentermometer gemessen habe oder ob mit einem mega genauen Spezielthermometer, wie ist das geeicht worden?	
Interview 29	Also man kann sich vieles ausdenken, ich denke, das kennen Sie auch aus der Chemie. Mh Das ist schon ganz wichtig, weil sonst können Sie mit diesen Werten ja überhaupt gar nichts anfangen. Das ist ja eigentlich auch übliche Praxis.	
Interview 29	Was oft vergessen wird, dass die Leute sagen: ich weiß das doch alle. Das mag ja auch so ein, mh nur jemand anderes weiß das ja nicht. Das heißt, die müssen ja eigenetlich, man muss das immer so beschreiben, dass auch jemand anderes, der relativ wenig Ahnung davon hat, das versteht.	
Interview 29	Das macht ein bisschen Mühe, ist ja aber eigentlich gute wissenschaftliche Praxis. Wenn das gegeben ist, dann kann man damit auch was anfangen.	
Interview 29	Ansonsten gibt, sollten Sie, wenn es irgendwelche Daten sind, also sagen wir mal speziellere Sachen mit spezielleren Formaten	
Interview 29	muss man sich überlegen, ob man sie entweder in üblichen Formaten (-) ablegt, eventuell auch zusätzlich, könnte zum Beispiel auch sinnvoll sein. Also klassische Beispiel bei, wenn ich Fotos gemacht habe, dann kommt aus üblichen, vernünftigen Kameras kommt ein Raw Format heraus also ein O Format des Kameraherstellers.	
Interview 29	Da hat Nikon ein anaders als Canon zum beispiel. Damit kann man nicht so richtig viel Anfangen, dafür braucht man wieder ein spezielles Programm. Diese Daten sollte man schon irgendwo ablegen, weil das sind ja immerhin die Rohdaten, die kann man mal umrechnen mal beispielsweise in JPEG Bild.	
Interview 29	Wenn ich die Rohdaten alleine ablege, dann kann der nächste im Moment noch nicht so viel damit anfangen, weil der muss wissen, welcher Kamerahersteller war das jetzt, vielleicht auch die Version, weil da gibt es ja manchmal auch Sprünge so alle paar Jahre.	

Interview 29		Ist also relativ schwierig, dass wäre in solchen Fällen immer gut, dass man nicht nur die Rohdaten ablegt sondern auch Daten, mit denen man was anfangen kann. Also zum Beispiel dann eben ein JPEG Bild oder ein TIF Bild, damit man sofort etwas sieht.	
Interview 29		Gilt auch für andere Dinge, also gerade (-) in den Naturwissenschaften bei vielen Messdaten, die werden mit Geräten von Firmen gemessen, die sehr, sehr eigensinnige Formate haben.	
Interview 29		Wo es manchmal so ist, dass nur auf diesem Gerät, diese Daten wieder eingelesen und verarbeitet werden können. Wenn sie diese speichern, dann brauchen sie eigentlich dieses Gerät dafür -das hilft ihnen ja nicht wirklich.	
Interview 29	15	Ich würde die immer speichern und würde dabei immer auch eine abgeleitete Version ablegen, sodass auch jemand anderes damit was anfangen kann, und sei es auch nur ein Bild der Messkurve oder soetwas.	
Interview 29		Aber auf jeden Fall so, dass man damit was anfangen kann. Weil das schlimmste ist, wenn sie etwas so ablegen, dass damit niemand jemals wieder etwas anfangen kann, das ist unsinn (lacht)	
Interview 29			Nun sind wir ja schon bei den Problemen und Schwierigkeiten.
Interview 29		Lacht*	
Interview 29			Welche weiteren Probleme und Schwierigkeiten beim Verwenden der Daten sehen Sie denn?
Interview 29		Also gerade in Deutschland, vielleicht auch noch in Europa, ist das Rechteproblem -also Urheberrecht, persönlichkeitsrecht. (-) Also viellerlei Zugriffsrechte sind ein großes Problem.	
Interview 29		Es ist auch ein großes Hinderungspotential. Also darf ich diese Daten überhaupt benutzen? Und wenn ich sie benutzen darf, was darf ich jetzt daran jetzt eigentlich machen, beispielsweise. Mh (-)	
Interview 29		Auch da wieder ein Beispiel, wirklich aus der Praxis, nicht ausgedacht. Für ein Forschungsprojekt aus den Geisteswissenschaften werden Abbilldungen von Gegenständen aus einer Bibliothek gekauft. Also das sind nicht die Originale, sondern man hat, hat, was weiß ich, ein Buch beispielsweise fotografiert.	
Interview 29		Mit dieser Fotografie macht man jetzt beispielsweise seine Forschung. Und, und man hat sogar was dafür bezahlt, dass man diese Fotografie bekommt. Und die Rechte lassen aber nur zu, dass diese Fotografie beispielsweise in einem wissenschaftlichen Artikel erscheinen, der gedruckt ist.	
Interview 29		Jettzt ist das zwar immer noch üblich, aber nicht richtig toll. Also man macht da heute eine PDF Datei raus. Aber dafür hat man aber die Rechte nicht. Das heißt, der selbe Artikel als PDF Datei hat dann an der Stelle, wo die Fotos über das Subjekt sind, und die Fotos braucht man ja, weil man braucht ja einen Bezug zur Beschreibung, sind einfach nur schwarze Kästen.	
Interview 29		Das ist jetzt vielleicht ein am Ende lösbares Problem, man hätte diese Rechte noch kaufen können. Aber das ist zum Beispiel ein Rechteproblem, was die Sache extrem verkompliziert.	

Interview 29		Oder es werden eben Schranken auferlegt, dass man soetwas nicht benutzen darf, wenn am Ende vielleicht eine kommerzielle Nutzung dabei rauskommt. Aber das wissen sie vorher vielleicht gar nicht. Also nehmen wir mal das Beispiel von vorhin mit dem künstlichen Benzin in der Chemie.	
Interview 29		Sie forschen vor sich hin, benutzen irgendwelche Daten, aber sie forschen jetzt nicht zielgerichtet, dass es irgendwann ein Treibstoff wird, der verkauft wird und eine kommerzielle Nutzung hat, sondern das Ziel it svielleicht ein ganz anderes. Und dann kommt, wie das oft so ist, sowas dabei raus.	
Interview 29		Aber dann haben Daten benutzt, die zum Beispiel diese kommerzielle Nutzung ausnutzen - geraten sie automatisch in ein Problem.	
Interview 29		Auch das kann dazu führen, dass Leute direkt am Anfang sagen: nein, ich benutze keine fremden Daten, weil am Ende habe ich vielleicht nach fünf Jahren ein Problem damit, ne ich mache nur meine Daten.	
Interview 29		Also, diese Rechteklärung ist tatsächlich ein großes Problem.	
Interview 29	20	Haben wir aber überwiegend in Europa und insbesondere in Deutschland. Also Europai st schon sehr rigide, aber Deutschland macht es nochmal schwieriger. Ist im asiatischen Raum aber auch USA wesentlich einfacher?	
Interview 29			Sehen Sie denn noch andere Probleme und Barrieren?
Interview 29		(-) Also technische sehe ich im Moment tatsächlich nicht. Die Frage ist, wenn wirklich alle ihre Daten speichern würden, wer finanziert dann eigentlich diese dann notwendigen riesigen Speichermengen und, und auch den dauerhaften Erhalt und so weiter.	
Interview 29		Aber das sind jetzt erstmal Dinge, wo ich sagen würden, das ist ähnlich wie bei der Entwicklung der Bibliotheken. Als man damit angefangen hat, hat man auch nicht darüber nachgedacht, dass man riesige Gebäude mit Hochregallager benötigt und so weiter.	
Interview 29		Das entwickelt sich dann. Ansonsten sehe ich eigentlich nichtwirklich mh großes Problem. Was noch ein Problem ist, ja, ist diese Interoperabilität, also.	
Interview 29		Gerade wenn Sie Daten haben, die sie mit - sind ja in der Regel digitale Daten, die Sie mit einem Computer bearbeiten wollen - dann müssen sie die ja auch noch in zehn Jahren bearbeiten können. Da haben wir bei gewissen Formaten zum Beispiel ein Problem.	
Interview 29		Wir haben ja alle schon erlebt, dass in den letzten zehn, fünfzehn Jahren, Formate auch wieder verschwunden sind oder Datenträger verschwunden sind. Und solche Formate verschwinden halt auch ständig.	
Interview 29		Mh Und (-) Je schweirger so ein Format ist, desto eher ist es vielleicht auch gefährdet. Das heißt es könnte sein, heute ist ein JPEG Bild überal lesbar, ist kein Problem.	
Interview 29		Persönlich gehe ich davon aus, dass wir auch in zwanzig Jahren noch sein, weil aufgrund der Verbreitung wird es immer jemanden geben, der das möglicht macht, dass man das lesen kann.	

Interview 29	Andererseits kann man sich auch vorstellen, dass plötzlich, warum auch immer, kein Interesse mehr dahingehened ist, das interessiert neimanden mehr. Und dann gibt es plötzlich keine Programme mehr, die JPEG Bilder lesen können.	
Interview 29	So. Und dann haben sie ein Problem. Was machen Sie dann? Wollen alle, die Sie haben, umformatieren? Dann sind eigentlich ihre Originaldaten weg.	
Interview 29	Wollen Sie eigentlich auch nicht, können Sie vielleicht auch gar nicht. Also, große Datenmengen, da brauchen Sie plötzlich Jahre oder Monate um das umzuformatieren , umzuspeichern.	
Interview 29	Das ist zum Beispiel auch so ein, das sind aber eher so technische PRobleme. Die man aber oft dadurch lösen kann, dass man es heuteverhältnismäßig verständlich macht.	
Interview 29	Also wenn man etwas heute macht, muss ich immer daran denken: kann ich es moren auch noch benutzen? Macht man im normalen Leben aber auch. WEnn ich einen Einkaufszettel schreibe, schreibe ich das mit irgendeinen Stift auf irgendein Papier, weil ich weiß, ich brauche das maximal eine Stunde.	
Interview 29	Wenn ich ein, eine Urkunde brauche, dass ich ein Haus kaufe und der Notar stellt eine Urkunde aus, dann nimmt er eben auch richtiges Urkundenpapier, was gewisse Qualität hat, was länger hält.	
Interview 29	Er nimmt auch einen stift, einen Fullfederhalter, nicht nur wegen des besonderen Moments, sondern weil es aus Erfahrung her einfach länger hält. Und so sollte man dann eben auch damit umgehen, jenachdem, was das für ein Zweck ist.	
Interview 29		Welche Beweggründee sehen Sie denn, Daten mit anderen zu teilen?
Interview 29	(---) Gute Frage. Naja, das eine ist, wenn ich selber Daten von anderen benutzen möchte, ist es vielleicht auch schlau, selber ein Angebot an andere zu machen.	
Interview 29	Also das ist ja der klassische Sharinggedanke. Nicht einer hat und der andere nimmt immer, sondern eigentlich geben beide Seiten etwas. Das wäre durchaus ein Beweggrund.	
Interview 29	Also wenn ich von anderen etwas benutze, muss ich auch selber etwas beitragen. Das führt (-) wenn es denn ideal läuft, zu einer Bewegung. Ansonsten hat es sicherlich ökonomische Gründe. Also es kann sein, dass ich dadurch entweder günstiger in meinem Forschungsprojekt werde oder schneller, weil ich vielleicht nicht nochmal drei Jahre messen muss, um das rauszubekommen.	
Interview 29	(-) Und es hat vielleicht auch den Grund, dass ich auch mal auf einen größeren Datenbestand zurückgreifen kann, den ich nir vielleicht selber gar nicht erarbeiten könnte.	
Interview 29	Das sind sicherlich so persönlche Beweggründe mh. Ich denke mal, dass viele (-), also gerade jüngere Forscherinnen und Forscher sind ja eher daraus aus, nochmal selber etwas zu messen, also wenn es um Messungen geht oder etwas selber zu generieren, so.	
Interview 29	Das ist auch verständlich, weil man ja irgendwie soetwas auch einmal gemacht haben muss und lernen muss. Da ist ja auch gar nichts gegen zu sagen.	

Interview 29		Nur vielleicht wird die Kombination daraus relativ gut. Also, ich habe in meinem eigenen Fach auch Messungen von anderen benutzt und meine eigenen Messungen, das habe ich miteinander verglichen und insofern glaube ich lernt man auch immer daraus.	
Interview 29		Das sind letzlich die Hauptbeweggründe. wenn man so will, ist es so ähnlich wie mit einer Publikation. Warum publiziere ich? Ich möchte der Welt irgendetwaas mitteilen.	
Interview 29		Wenn ich daran kein interesse dran hätte, würde ich das auch nur für mich schreiben und in den Schrank legen. Auch da teile ich ja mein Wissen mit anderen und andere teilen ihr Wissen mit mir.	
Interview 29	25	Und daraus entsteht dann vielleicht ein Mehrwert, Warum sollen wir das jetzt nicht auch auf Daten(-) anwenden, dieses Prinzip.	
Interview 29			Jetzt haben Sie schon die persönlichen Faktoren angesprochen. Wenn es jetzt wieder in Richtung Probleme und Barrieren geht. Sehen Sie da auch Gründe, warum Menschen nicht teilen? Oder Forscher ihre Daten nicht teilen?
Interview 29		Also Sie meinen eher auf technischer Ebene?	
Interview 29			Auf persönlicher Ebene.
Interview 29		Achso persönlich, entschuldigung. Also klar, natürlich. Es gibt von der Persönlichkeit her natürlich Menschen, die sagen, das ist meine Arbeit, das gehört nur mir, das gebe ich nicht her.	
Interview 29		Und im Zweifel auch die Angst hegen, die ich aber auch durchaus verstehen kann, die Angst hegen, ich habe das mit viel Mühe erzeugt und gemacht, vielleicht Befragungen gemacht mit viel Mühe, ich bin zu hundert Leuten gegangen und habe mit hundert Leuten gesprochen jeweils eine Stunde und ich habe mir ganz viel Mühe gegeben, mir das alles wieder angehört und Transkribiert und so weiter.	
Interview 29		Und dann kommt jemand anders und der hat das alles nicht gemacht und der benutzt das einfach und hat vielleicht eine tolle Idee, die ich nicht hatte.	
Interview 29		Diese Angst ist glaube ich urmenschlich, dass jemand anderes besser sein könnte als man selber und dass er dann auch noch besser ist mit Dingen, die man selber gemacht hat, also vielleicht erfogreicher.	
Interview 29		Und es gibt ja in der Wirtschaft und wicherlich auch in der Wissenschaft genügend Beispiele ´, wo das so ist.	
Interview 29		Wo man immer so denkt, die haben das selber erfunden, aber im Grunde ist das garnicht so.	
Interview 29		Also die Firma Apple mit ihrem Design, sicher schon in gewisser Weise selbst gemacht, aber 90% davon stammt aber ursprünglich von der Firma Braun. Nur braun war damit lange nicht so erfolgreihc.	
Interview 29		Könnte man jetzt auch sagen, eine nachnutzung, wenn man so will. Da gegen kann man ja aber nichts sagen, weil diese Ängste muss manja ernst nehmen, die kann man auch nicht zerstreuen, weil was soll man denjenigen sagen? Nein, das wird nicht passieren - und dann macht es morgen jemand.	

Interview 29	Was sollen wir da tun? Das geht nicht, so. Da kann man dann ja immerhin könnte man ja sagen: solange du mit den Daten wirklich arbeitest, musst du die auch nicht offen und frei zur Verfügung gestellt werden. Aber irgendwann kommt vielleicht der Punkt, wo man selber damit gar nichts macht, vielleicht auch nicht zukünftig, sondern abgeschlossen hat und sich einem anderen Thema zuwendet.	
Interview 29	Spätestens in dem Moment könnte man ja sagen, gut jetzt kann ich sie anderen zur Verfügung stellen. (-) Aber ich finde schon, dass man diese Ängste oder Befürchtungen auch ernst nehmen muss, weil das ist sicherlich auch durch die weltweite Vernetzung deutlich anders geworden.	
Interview 29	Also früher konnten halt Leute nicht aus Asien, aus den USA, oder Russland oder sonst woher, an irgendwelche Daten so einfach kommen. Sie waren halt in meinem Schreibtisch, da konnte man ja nicht ran.	
Interview 29	DAs ist natürlich heute überhaupt kein Problem und das führt schon zu ganz anderen Verzerrungen, Das kann ich schon in gewisserweise verstehen, dass man es nicht möchte. Und dann gibt es noch die Ängste in all denen Bereichen wo man wirtschaftlichen Nutzen erzeugen kann.	
Interview 29	Also, mh (-) das ist natürlich in der Wrtschaft, in der Industrie, besonders vertreten, das ist klar. Aber mh da gibt es ja immer wieder Mischungen mit der Forschung. Also Medikamentenforschung findet eben auch in den, in der medizinschen in den Krankenhäuser statt, in der Chemie oder in der, den Ingenieurswissenschaften werden ja auch Dinge gemacht, die sich finanziell verwerten lassen.	
Interview 29	Dass man die Angst hat, dass es ein Konkurrent nimmt und mit viel weniger Aufwand daraus Ergebnisse produziert und dann damit Erfolg hat, ist natürlich klar.	
Interview 29	Oder vielleicht auch nur günstiger sein kann, weil er nicht die 50 Millionen in die Forschung stecken musste. Das kann ich verstehen, und ich finde aber dass spricht ja nicht dagegen, dass die Daten, dass Daten oder alles nicht zutrifft und dass man die Sachen trotzdem weiter teilen kann.	
Interview 29		Wird das Teilen von Daten denn anerkannt?
Interview 29	Interessanterweise, ja. Das hängt an den Fächern. Interessanterweise im angelsächsischen Raum eher als in (-) Europa, würde ich jetzt so nicht sagen, aber viellleicht in Deutschland.	
Interview 29	Deutschland ist das noch nicht so anerkannt. Das zeigt sich unter anderem daran, dass gerade im angelsächsischen Raum gibt es extra zeitschriften, also sogenannte Data Paper, wo man auf 1,2,3 Seiten seinen Datensatz beschreibt.	
Interview 29	Also eine Publikation daraus macht und die auch anerkannt wird. Selbst in großen Zeitschriften, wo normalerweise nur wissenschaftliche Artikel erscheint, gibt es Sektionen, wo Leute so Kurzbeschreibungen ihrer Datensätze machen können. Und das zählt auch als Publikation.	

Interview 29		Das ist bei uns noch nicht so. Bei uns wird das vielleicht anerkannt wahrgenommen, aber das zählt nicht in ihrer Publikationsliste. Wenn sie 50 Journalartikel und vielleicht 20 Data Paper, dann würde bei uns nicht sagen: oh sie haben siebzig Publikationen.	
Interview 29		Sondern würden sie sagen fünzig. Das Rest, "ja, gut, nett", aber es ist noch nicht so anerkannt. Aber das kommt glaube ich auch wirklich. Weil auch da wachsen die Wissenschaften ja immer enger zusammen. Und wir werden uns in Deutschland auch daran orienteren müssen, was weltweit üblich ist.	
Interview 29		Und das wird hier auch kommen, da bin ich relativ sicher. Also ansätze dafür gibt es schon. Wir haben gerade hier bei uns letzte Woche, ist noch (-) Online Journal für Data Paper ins Leben gerufen, damit es einfacher wird von der UniHamburg	
Interview 29		. Da muss ich jetzt mal gucken, wie viel Interesse es am Ende gibt. Also man muss halt Angebote machen und gucken, ob es was wird. Ich kann jetzt nicht erwarten, dass ich gleich überrannt werde mit Artikeln und mh, sondern man muss wenn irgendjemanden einen Datensatz gespeichert hat, gibt es nicht auch eine Möglichkeit, eine Seite darüber zu schreiben und so.	
Interview 29		DAs kann man dann ja doch vielleicht angeben irgendwie. Vielleicht wird es dann so mittelfristig. Also das ist immer so meine Idee dahinter: durch Angebote und durch (-) Möglichkeiten, die man leuten bietet, und Informationen, etwas zu verändern.	
Interview 29	30	Geht nicht immer, schon klar, aber meistens ist es nachhaltiger, als wenn man es irgendwie vorschreibt.	
Interview 29			Inwieweit hat dann die Institutskultur einfluss auf das Data Sharing
Interview 29		Die, die, also ich würde sagen, die Fachkultur. Weil die Institute dann auch mit den Föchern zu tun haben, muss man sagen. Das hat auf jeden Fall einen großen Einfluss. Was ich ja auch schon gesagt habe, in den Naturwissenschaften ist es eher verbreitet als in denmeinetwegen Geisteswissenschaften.	
Interview 29		In den Sozialwissenscahften ist es auch shcon wieder mehr verbreitet. Es gibt Bereiche, da ist es es praktisch überhaupt noch nicht üblich, also gar nicht. Dann wird auch immer noch disktiert, was sind denn eigentlich Daten?	
Interview 29		Da denken dann natürlich alle nur an Zahlen, also. Ja, die berühmten Messwerte. Da höre ich auch immer wieder: ja, ja, die Naturwissenschaflter haben ja Daten, die haben Zahlen, klar.	
Interview 29		Aber das für jemanden, der vielleicht nur mit Texten arbeitet, einfach seine Texte seine Forschungsdaten sind oder jemand, der mit Bildern arbeitet oder mit Filmen, dann sind das eben die Forschungsdaten.	
Interview 29		Genausowie Sie jetzt mit Interviews, dann sind Ihre Interviews Ihre Forschungsdaten - muss ja so sein. Es ist ja nicht gebunden an Zahlen. Das ist eben häufig auch so ein Missverständnis.	
Interview 29		Muss man eben wieder auch sagen, wenn jemand öffentliche Güter analyisert, dann muss er das nicht wieder kopieren und abspeichern, weil davon gibt es bestimmt genügend Kopien.	

Interview 29		Das ist, das muss er ja nicht teilen, das wäre auch Unsinn. Da kann man auch Hinschreiben: Goethe, Ausgabe und so und so. Weil die wird man überall auf der Welt finden.	
Interview 29		Also auch da muss man wieder gucken. ODer wenn jemand Filmwissenschaft betreibt und über einen Spielfilm redet, dann muss er den sicherlich auch nicht speichern, weil diesen Film gibt es in (-) zig Auswertigen Archiven, Mediatheken und so weiter, das ist ja gar kein Problem.	
Interview 29		Also man muss immer gucken, was man wie benutzt und was man macht und wie sicher denn diese Dinge sind.	
Interview 29		Also wenn es zum Beispiel etwas ist, was nur eine gewisse Zeit aufbewahrt wird - also denke gerade an Rundfunkanstalten, die bewahren auch nicht alles auf, schon aufgrund der großen Menge - und wenn ich jetzt anfangen würde soetwas zu sammeln und vielleicht dann mal Nachrichten auszuwerten,	
Interview 29		und ich weiß aber dass diese Nachrichten maximal drei Monate bei den Rundfunkanastalten aufbewahrt werden, dann sollte ich aber vielleicht diese Nachrichten selber aufbewahren, die ich benutzt habe.	
Interview 29		Also auch das ist etwas, wo man genau hingucken muss. Aber das muss, das hat was mit der Fachkultur zu tun. Mittlerweile ist es tatsächlich so, dass mh (-) eigentlich weltweit in allen Fächern diese Themen diskutiert werden und in allen Fächern es auch angegangen wird, mehr oder weniger und unterschieldich intensiv.	
Interview 29		Man kann aber auch noch nicht mal generationsmäßig sagen, dass jüngere das besser machen als ältere - das stimmt so auch nicht. Also, es ist glaube ich sehr divers.	
Interview 29	35	Und wie immer gibt es Leute, die machen das von Anfang an und machen das auch sehr gut. Und dann gibt es Leute, die machen erstmal gar nichts, kommen vielleicht spöter dazu, Und es gibt eine große, breite Menge in der Mitte. Das ist einfach so.	
Interview 29			Nun haben Sie ja schon viele Probleme und Barrieren angesprochen. Welche glauben Sie denn ist die größte Barriere?
Interview 29		Also eigentlich ganz simple, das ist nicht wirklich eine Barriere. Die größte Barriere ist, den inneren Schweinehund zu überwinden es zu machen.	
Interview 29		(-) Weil, ja, es mag technische Probleme geben, es mag rechtliche Probleme geben, es mag alles geben und kann man auch alles irgendwie lösen.	
Interview 29		Ich glaube tatsächlich das größte Problem ist, wie immer XX man muss es einfach machen. Also man muss sagen: ich mache das jetzt. Und dann hat man vielleicht Herausforderungen, aber die wird man auch irgendwie gelöst bekommen.	
Interview 29		(-) Also, das ist aber ein Ding, das können wir ja niemandem abnehmen - ich wüsste auch nicht wie. Das muss jeder für sich selber entscheiden. An sich kann man es nicht jemandem abnehmen.	
Interview 29		Und letzlich muss man auch verstehen, da müssen die Leute selber drauf kommen. XX	

Interview 29	Ja, aber irgendwann entschliessen sie sich: ich will jetzt meine Masterarbeit schreiben. Und so glaube ich, ist das auch. Also die größte Barriere ist eigentlich bei einem selber.	
Interview 29	"Ich will das jetzt machen und dann mache ich es", so. Da habe ich aber keine Lösung, das muss ich ehrlich gestehen [lacht]	
Interview 29		Inwieweit wollen Sie denn die Data Sharing Aktivitäten in Zukunft aufbauen?
Interview 29	Also wir haben ja, wir sind tatsächlich kein Projekt sondern wir sind ein Zentrum, das für Dauer angelegt ist. Unser Service ist auch auf Dauer angelegt.	
Interview 29	Also insofern auf Wachstum. Und meine Idee ist, man muss das Angebot machen, das haben wir jetzt. Wir haben auch schon erste Daten eingesammelt, also Leute haben schon was bereit gestellt.	
Interview 29	So, jetzt XXX das Angebot verbessern, dass lernt man dann im Laufe der Zeit, welche Anforderungen gibt es. Und ab irgendeinem gewissen Zeitpunkt wird es auch dazu kommen, dasss wir feststellen können, und das ist sicherlich auch ein Problem: was dürfen wir überhaupt überwachen.	
Interview 29	Man könnte natürlich mit technischen Tools jegliche Aktivitäten überwachen. Also wer guckt das an, wer lädt es runter und so weiter. Da gibt es aber dann den Datenschutz, der dem weitestgehend widerspricht.	
Interview 29	Insofern ist es auch ein bisschen Schwierig, sind denn Datensätze wirkiich benutzt worden. Trotzdem hoffe ich - und ich animiere die Leute auch immer dazu, also, weil es ja meistens so ist, dass es dann über Zitate geht - also, wenn jemand so einen Datensatz benutzt, dann wird er ja wenn er ja wenn er ordentlich arbeitet, das zitieren bei seiner Arbeit.	
Interview 29	Wenn das jemand anderes, vielleicht wird er sogar angesprochen, wenn der das jemand mitkriegt, dass die uns auch noch Rückmeldung geben, ja, dann hat tatsächlich jemand über diese Platform die Daten gefunden und war damit erfolgreich und hat sie benutzt.	
Interview 29	(-) Das ist so ein bisschen das Ziel so für die nächsten, für die nächste Zeit, dass wir so tolle Beispiele haben. Aber die können wir nicht produzieren, daraus muss man warten. Dass wir solche tollen Beisoiele haben, die wir in gewisser Weise vermarkten können und das Thema positiv zu besetzen und voranzutreiben, so.	
Interview 29	Vielleicht auch mal ein negatives Beispiel finden, wäre ich auch, finde ich auch nicht schlimm wenn man mal sagt: ja, da hat jemand sowas benutzt und noch nichtmal zitiert, und das ja geht eigentlich gar nicht.	
Interview 29	Auch das, wollen wir ja nicht die negativen Seiten verschweigen müssen, macht ja auch ein schlechtes Bild. Und das würde ich gerne als Anregung benutzen, sodass	
Interview 29	man sagen kann: ja, es kann ja doch einen Effekt haben. Mh Leider habe dann, also, ja, ich kenne positive Beispiele, aber noch nicht mit den Daten, die wir konkret gespeichert haben und wenn kommen die woanders her.	

Interview 29		Aber, das heißt, es macht mir schon Hoffnung, dass wir dann schon irgendwann sowas haben werden und ja. und ja. Wir arbeiten gerade daran, im Rahmen dessen, was erlaubt ist, auch herauskriegen können wie oft sind beispielsweise solche Dinge angeguckt worden und wie oft sind sie auch wirklich runtergeladen geworden.	
Interview 29		Also, angucken kann man sagen: ok, Interesse. Runtergeladen, zumindestens soviel Interesse, dass jemand sichdie Mühe macht das runterzuladen.	
Interview 29	40	Was er damit macht, können wir dann nicht mehr ä ist ja klar. Aber wenigstens, dass wir das Wissen, so.	
Interview 29		Aber auch da muss man sich überlegen, was bedeutet das jetzt "erfolgreich". Das kann sein, dass es exakt einen Interesseten gibt, der das einmal runtergeladen hat und damit irgendetwas macht, wo er am Ende den Nobelpreis für bekommt.	
Interview 29		Es kann (-) ja, auf dem ersten Blick würde man sagen, ist ja nicht erfolgreich, weil nur einmal runtergeladen. Es kann aber auch Dinge geben, die werden hundert mal runtergeladen, da passiert aber gar nichts.	
Interview 29		(-) Da würde man aufgrund der Zahl sagen, super erfolgreich und nachgefragt, aber vielleicht ist das wirklich irrellevant. Es ist wirklich schwieirig daraus was zu machen.	
Interview 29		Es ist ja nicht wie bei Verkeraufszahlen, wo wir jetzt wirklich irgendwie (-) dann sofort einen Erfolg haben, also. es klingelt ja dann leider nicht die Kasse, das wäre schön [lacht].	
Interview 29		Das heißt dann noch nichts. Auch das sind aber Probleme, die man im Grunde genommen aus anderen Bereich kennt. Es gibt Filme, die gucken nur wenige Leute an, aber die würde jeder als Meilenstein der Filmgemschichte bezeichnen und ganz wichtig.	
Interview 29		Und Filme, die werden Millionfach angeguckt, aber eigentlich sagt dir ist eigentlich Quatsch, ist nette unterhaltung, aber hat keinen Wert.	
Interview 29		Und das ist, so ähnlich muss man das auch sehen. Die schiere Masse sagt eigentlich noch nichts aus.	
Interview 29	1,16		Um jetzt zu einem ganz anderen Thema zu kommen. Das sind auch schon meine letzten beiden fragen jetzt.
Interview 29		Wir sind auch gut in der Zeit [schmunzelt]	
Interview 29			XX Nun interessiert mich, wie schätzen Sie den Einfluss der Technologien auf den zukünftigen Alltag ein?
Interview 29		Also, ja. (-) Also ich stamme aus einer Generation, wo es dies alles noch gar nicht gab, das muss ich dazu sagen. Das ist in sofern ganz wichtig, ich kann mir auch immer vorstellen, dass man auch ohne all diese Dinge leben kann.	
Interview 29		Also, ich bin groß geworden ohne Internet, ohne Handy, und wie man sieht konnte man ja überleben. Alles hat sich erst entwickelt in der Zeit.	
Interview 29		(-) Das ist manchmal ganz interessant, weil diese Sichtweise dazu führt, dass ich vielleicht - oder meine Generation, einerseits ist sie die Generation, die diese Dinge entwickelt hat und vorangetrieben hat. Es sind ja nicht die ganz jungen, die das Internet entwickelt haben.	

Interview 29		Es ist ja eher so eine ältere Generation. Und gleichzeitig sind wir auch so ein bisschen skeptisch in all diesen Dingen. Natürlich wäre es, also ich glaube schon, dass KI und Big Data einen Einfluss haben wird und auch eine sinnvolle Anwendung in gewissen Bereichen.	
Interview 29		Ohne Frage. Wobei Big Data ist ja noch mal etwas anderes, das ist so ein schwammiger Begriff, finde ich. Bei dem geht man eher davon aus, dass man ähm sage mal, hauptsächlich Konsumentendaten hat. Also, entweder Daten, was haben Leute alles gekauft oder wie verhalten sich Menschen über Handyprofile oder irgend solche Sachen oder eben Daten aus sozialen Netzwerken oder von Twitter oder was auch immer.	
Interview 29		Man versucht dort ein Muster zu erkennen und die benutzt man eigentlich überwiegend dafür um dann wiederum wirtschaftlich erfolgreiche Angebote daraus zu generieren, beispielsweise. Das ist ja eigentlich der Sinn dahinter.	
Interview 29		Also, ja. Es wird auch genutzt um irgendwelche Erkenntnisse über gesellschaftliche Veränderungen zu erfahren, aber eigentlich geht es ja darum, irgendwas zu verkaufen. Dafür benutzt man dann diese Dinge.	
Interview 29		Dazu benutzt man auch so ein bisschen KI, aber KI ist eben (-) im Moment aller Munde und wird ja auch stark gefördert.	
Interview 29		Wo ich nicht so richtig dran glaube (-) sind wirklich diese vollkommen autonome Machinen, mmh meinetwegen komplett autonom fahrende Autos, die in jeglicher Situation, die man sich vorstellen kann (-)	
Interview 29		es kann sein, dass es das in dreißig, fünfzig Jahren gibt, will ich auch überhaupt nicht ausschliessen, aber, also zumindest in der nahen Zukunft sehe ich das überhaupt gar nicht.	
Interview 29		Und die Frage ist auch, ist das sinnvoll. Das muss man ja auch überlegen, Nur weil es technisch geht, heißt das ja noch lange nicht, dass es sinnvoll ist. Man kann ja viele Dinge machen, aber das heißt nicht unbedingt, dass sie sinnvoll sind.	
Interview 29		mmh (-) Also im Moment zumindestens sehe ich nicht wirklich intelligentes Verhalten von Machinen, sondern denen wird irgendetwas beigebracht und das versuchen sie halt mechanisch einfach immer wieder abzuarbeiten, nach einem gewissen Muster.	
Interview 29		Also, das beste Beispiel sind die Roboter in der Chemie, Fabriken.	
Interview 29	45	Man könnte den Eindruck haben, die sind intelligent und können auf alles mögliche reagieren, das können Sie aber gar nicht, sondern denen ist ja nur beigebrahct worden, welche Schritte sie machen müssen, und das ziemlich genau, ziemlich exakt und das machen sie auch.	
Interview 29		Aber das hat mit Intelligenz ja nicht wirklich was zu tun.	
Interview 29			Glauben Sie, das hat dann auch Einfluss dann auf die Forschungsgemeinschaft?
Interview 29		Ja, das auf jeden Fall. Weil, also der Trend wird sicherlich dahin gehen, dass man a größere Datenmengen benutzt, also nicht unbedingt nicht dieses Big Data, aber immer mehr Daten.	

Interview 29	Auch mit dem Argument, dass man ja (-) sagen wir mal, Methoden hat, nicht unbedingt KI, die es ermöglichen, diese Menge an Daten auch auszuwerten (-) ja (-) also, das gilt in allen Bereichen. Also, wenn Sie an die Chemie denken, da haben Sie vielleicht früher drei Versuche gemacht.	
Interview 29	Mehr konnten Sie in einer realistischen Zeit gar nicht auswerten, heute machen sie vielleicht hundert Versuche, weil sie da irgendein Sequenzer auf dem Tisch stehen, der Rund um die Uhr die Sequenzen dann macht und Sie kommen am nächsten Tag wieder und haben die Ergebnisse.	
Interview 29	So, wenn Sie das alles per Hand machen würden, bräuchten Sie vielleicht zehn Jahre dafür. Also würden Sie es nicht machen, dann würden Sie nur wenige Dinge machen.	
Interview 29	Man versucht ein bisschen durch die Masse, vielleicht auch Qualität zu erzeugen in der Annahme, wenn ich viele XXX mache, mitteln sich Fehler raus und so weiter, aber das muss vielleicht auch gar nicht so sein. Auch das geht wieder in diese Richtung, wie haben Sie das denn vor 40, 50 Jahren gemacht - wie konnte das eigentlich sein?	
Interview 29	(-) Auch da gibt es ganz praktische Beispiele, war ja nun gerade Mondladundung. Die Rechenkapazität dieser Rechner - jedes Handy ist, jedes Billighandy ist heute leistungsfähiger.	
Interview 29	Jetzt kann man sich fragen, wie konnte das überhaupt sein. Ja, weil Leute mit diesen beschränkten Ressourcen sich überlegt haben, sehr reflektiv, wie kann man das so programmieren, dass man wenig Speicherplatz braucht und trotzdem der Rechner das wirklich leisten kann.	
Interview 29	Das ist etwas, was wir heute permanent erleben. Sie kaufen einen neuen Rechner, oder ein neues Tablet. Das ist super leistungsfähig in dem Moment, wo sie es gekauft haben.	
Interview 29	Weil es so leistungsfähig ist, machen die Programmierer immer tolle XX sie brauchen immer mehr Platz und nach spätestens einem Jahr stehen Sie da und sagen: oh, was ist denn jetzt passiert, das kann doch gar nicht sein.	
Interview 29	Und warum? Weil man nicht mehr effizient zum Beispiel Software entwickeln muss. Das muss man gar nicht mehr. Man ha ja genug Leistung, dann soll sich der Kunde doch ein neues Gerät kaufen, das Leistungsfähiger ist.	
Interview 29	Wenn Sie (-) ich hatte einen der ersten Apple zuhause, wenn ich da einen Brief geschrieben, das ging auch damals schon, es gab eine Textverarbeitung, die hat alles gemacht um einen Brief zu schreiben, mehr brauchte man gar nicht. Dieser Brief war wenige Kilobyte groß, wenn der gespeichert wird.	
Interview 29	Ich habe mir mal den Spaß gemacht, dass ich so einen Brief, der wenige Kilobytes war, dass ich den dann immer im Laufe der Zeit immer so übernommen habe, da waren nicht mehr Informationen drin, also ja, der sah immer nochgenauso aus. Mh Der hatte dann irgendwann mehrere hundert Kilobyte.	

Interview 29		Der wurde immer größer, weil immer mehr Inforamtionen im Hintergrund gespeichert werden. Man kann das bestimmt alles begründen, aber für den eigentlichen Zweck, ich möchte am Ende einen Brief ausdrucken, ist das überhaupt alles gar nicht notwendig. Macht man aber, weil man iimmer mehr Speicherplatz hat und so weiter.	
Interview 29		Heißt, man erzeugt auch Daten, die man gar nicht braucht. Das ist jetzt ein Problem, wo ich glaube, also man hat ein Problem, viel zu viele Daten. Das will man beantworten durch KI. Und weil man dann KI hat, kann man dann noch mehr Daten haben, zum Beispiel. Ein autonom fahrendes Auto braucht so viele Daten, Messwerte und Zusatzdaten, das kann man sich eigentlich gar nicht wirklich vorstellen.	
Interview 29		Dafür braucht man super leistungsfähige Systeme, damit das überhaupt funktioniert. Nun kann man sich überlegen, ist das sinnvoll? Braucht man das wirklich nur weil ich von A nach B fahren will?	
Interview 29		(-) Also, weiß ich gar nicht. Also insofern: ja, ich bin gespannt. Ich würde es gut finden, wenn man sich, das ist aber auch meine persönliche Meinung, wenn man sich auf die Bereiche, wo es Sinn macht, konzentriert, und nicht auf Bereiche, wo es relativ wenig Sinn macht, wenn ich ehrlich bin.	
Interview 29		So, und wenn wir uns davon nicht zu sehr abhängig davon machen. Das ist auch so ein großes Problem.	
Interview 29	50		Vielen Dank für Ihre Einschätzung dazu.
Interview 29			Ja, ich bin auf jeden Fall auch schon voll gespannt, was die Technologien angeht.
Interview 29		Ja das ist so ein bisschen - also im Moment ist es getrieben, gerade im Bereich KI, das müssen Sie mal ein bisschen verfolgen. Die Bunderegierung, Europa, die ganze Welt setzt auf KI.	
Interview 29		Es gibt Förderprogramm ohne Ende. Sie brauchen eigentlich im Moment, egal was sie machen wollen, nur einen Förderantrag schreiben und irgendwas mit KI da rein schreiben.	
Interview 29		Eigentlich, kriegen Sie sofort Geld, gut wenn Sie ein bisschen auch die Hintergründe haben,klar. Aber letzlich ist es das. Das ist dieses typische Prinzip, das hatten wir schon immer, das kennen wir aus der Vergangenheit. Früher war eben was mit Kernforschung, da kriegte und so weiter, da war das nicht mehr in, dann gab es plötzlich Genforschung.	
Interview 29		Dann hieß es wir werden alle 1000 JAhre alt und werden nicht mehr krank und so weiter, dann war das jetzt auch nicht mehr der große Hit, jetzt ist es KI. Also, ja.	
Interview 29		Und die Alltagsprobleme, finde ich, werden damit überhaupt nicht gelöst, im Gegenteil. Das fällt hinten vor. Also, gucken Sie sich jeden Fahrkartenautomaten an oder andere Dinge. Das sind ganz relativ einfache Abläufe, hat mit Intelligenz praktisch gar nichts zu tun.	
Interview 29		Aber die funktionieren schon nicht richtig. Warum sollte ich jetzt Zutrauen haben, dass man irgendwie Intelligenz irgendwo nachbilden kann. Ich weiß auch gar nicht, ob das überhaupt interessant ist für uns.	

Interview 29		Ich möchte ja eigentlich nicht immer nur rumsitzen und gar nichts mehr zu tun haben, weil alles Machinen für mich erledigen. Das gilt für die Forschung natprlich auch	
Interview 29		Wenn man sich jetzt vorstellt, dass vielleicht irgenwann nur noch Algorithmen irgendwelche Analysen machen und ich selber gar nichts mehr machen muss, dann bin ich ja überflüssig.	
Interview 29		Was bleibt denn dann noch? Dann bin ich vielleicht noch der Handlanger, der die Flüssigkeiten anmischt oder irgendwie sowas. (-) Das ist leider schon in vielen, zum Beispiel in vielen Bereichen ist das schon Realität. Also wenn man in den Analysebereich geht oder große Labore oder so,also , Bluttest für die Medizin oder Lebensmittelkontrolle und so weiter, das ist ja unglaublich automatisiert schon.	
Interview 29		Kann man auch verstehen, weil es ja quasi millionenfach immer das gleiche ist. Aber da wo früher eben ganze Herrscharen von Laboranten und Laborantinnen standen, die das selber gemacht haben und beherrschten, sind häufig nur noch relativ wenige, die diese Machinen bedienen.	
Interview 29		Und das, ist das jetzt erstrebenswert? Das weiß ich eben auch nicht so richtig. Und ob das dadurch besser geworden ist, weiß ich auch nicht so richtig, ob die Analyse wirklich besser geworden. Also das ist eben auch so ein, also es ist auch ein gesellschaftliches Problem. Wollen wir das wirklich?	
Interview 29		XX Norwegen ist ja einerseits sehr auch Fortschrittsgäubig und durchaus modern, aber auch sehr sehr konservativ und auch langfristiges Denken.	
Interview 29	55		Irrelevant
Interview 29			Haben Sie sonst noch etwas, was Sie mir zum Thema Data Sharing mitteilen möchten?
Interview 29		[lacht] Mh (-) Was wir jetzt, also es ist zwischendurch mal angeklungen, ich will jetzt auch gar nicht sagen, dass das für den wissenschaftlichen Bereich so interessant ist.	
Interview 29		Generell kann man aber sich natürlich auch überlegen, ob das vielleicht auch Vergütungsmöglichkeiten gibt. Ich habe da jetzt auch keine Lösung und wüsste auch nicht, wie man sowas machen sollte und so weiter. Aber natürlich kann man sich das auch überlegen. Man kann sich ja natürlich auch immer überlegen, es muss immer alles offen und frei Verfügung sein, ich bin sehr dafür, man kann auch das komplette Geld abschaffen, aber dann muss ich auch das Brötchen umsonst kriegen, also, ja.	
Interview 29		Ich muss, darf nicht so sein, dass dann immer: ja, verdienen Sie ihr Geld doch irgendwie anders. DAs funktioniert ja nicht. Wenn man zum Beispiel die wissenschaftlichen Verlage kritierst. DAnn kann man sicherlich aufgrund der Preise. Aber das Prinzip, dass es jemand gibt, der Arbeit reinsteckt und dafür auch Geld bekommt und damit wiederum Menschen finanziert, das ist ja nicht das schlechteste unter den Prinzipien.	

Interview 29		Es ist ja auch nicht künstlich irgendwo erfunden worden, also es hat sich ja in der Menscheihtsgeschichte herausgebildet, damit dass man, ja, der eine hatte was gemacht und der andere und, und hat was anderes gemacht und das konnte man nicht so gleichwertig, so (-) alo brauchte man irgendeinen Austausch, und (-) also insofern könnte man auch darüber nachdenken, macht das Sinn, kann man ja als Hypothese aufstellen: macht es Sinn auch einen Vergütungsmechanismus vorzusehen zumindestens.	
Interview 29		Vielleicht ist es auch sinnfrei, kann ja auch sein.	
Interview 29		Aber Sharing heißt ja nicht automatisch kostenfrei, großes Miss- ich glaueb da sind wir wieder am Anfang- großes Missverständniss tatsächlich, ja, also. (-)	
Interview 29			Ja.
Interview 29		Also Car Sharing ist ja auch nicht automatisch kostenfrei, warum auch? Warum soll jetzt Daten Sharing kostenfrei sein? (-) Also. (-) Aber da müsste man ein Vergütungsmodell haben, so. Muss man drüber nachdenken. (-) Nö, Ansonsten wüsste ich sonst auch nichts, glaube habe schon viel zu viel gesprochen.	
Interview 30	Time	Interviewee 30	Interviewer
Interview 30	0		Davor möchte ich nochmal den Datenschutzrahmen abklären. Also (…) ich verwende es (das Interview) nur, ich werde es transkribieren. Das bedeutet - mit deinem Einverständnis natürlich - das Interview aufnehmen, abschreiben und mit dem geschrieben weiterarbeiten.
Interview 30			Hast du ansonsten noch im Vorhinein etwas, was du abklären möchtest?
Interview 30		Ich denke, da werden wir wiohl über das Interview noch dazu kommen, kann ich mir vorstellen.	
Interview 30			Gut, das heißt, den Fragebogen, den ich dir geschickt habe, da werde ich mich nicht zwangsläufig an die Reihenfolge halten, nur dass du nicht irritiert bist oder so.
Interview 30		Gut.	
Interview 30			Wenn du ihn offen hast, ist es auch gut.
Interview 30			Meine erste Frage, die ich an dich richte, die steht tatsächlich nicht in dem Fragebogen, weil es mich einfach interessiert, was über dich zu erfahren und was du bei deiner Arbeit machst. Meine Frage ist demnach: was gehört zu deinen täglichen Aufgaben bei (...)?
Interview 30		Also ich bin in mehrere Bereiche involviert. Einmal grundsätzlich alles was eben mit Screening zu tun hat. Ich starte dann bei der Entwicklung von Essays die eben halt Screening Format getestet werden können, die Durchführung der Screens, Datenanalyse, Auswertung, also eigentlich alle Schritte im Projekt durch bis zu den validierten Tips. Das mache ich im Moment sehr viel im Bereich Bindungskinetiken, was da untersucht wird. Und bin bei uns noch zusätzlich im Data Management involviert.	
Interview 30			Das heißt, was machst du dann da?

Interview 30		Im Data Management? Also wir sind halt ein Team und versuchen gerade den optimalen Data Management Plan für unser Institut aufzubauen bzw eigentlich für unseren Standort besser gesagt.	
Interview 30		Das heißt zu schauen, wie können wir bei uns sinnvoll unsere Daten ablegen, so dass sie möglicht dahingehend an den FAIR Principles zu folgen - das hast du wahrschienlich schon gehört, oder?	
Interview 30			Ja
Interview 30		Das klingt ja alles in der Theorie ganz schön, dass die Daten entsprechend annortiert sind, dass man sie auch hinterher mit diesen Daten arbeiten kann vor allem andere mit den Daten anderen arbeiten können.	
Interview 30		In der Praxis ist natürlich alles sehr viel schwieriger umszusetzen. Das entwickelt sich halt langsam davon weg, dass halt jeder in seinem eigenen Laborbuch arbeitet.	
Interview 30		Also davon sind wir mittlerweile seit 5 Jahren weg. Also seit dem haben wir ein elektronisches Labor Journal, das aber im Prinzip genauso aussieht wie das alte Laborbuch, das man auch geführt hat. Nur ist es eben elektronisch ist.	
Interview 30		Aber es ist immer noch Text mit Bildern, mit, ja, Verknüpfungen von Analysefiles, also die sind dann wieder darin eingebettet. Das heißt, es ist nicht direkt eine Datenbank, aus der Daten	
Interview 30		wieder extrahiert werden können, sondern es ist eben ein digitlaes Laborbuch, also, ja. Es ist besser als wenn man irgendwie Laborbucher eingescannt. Das war jetzt für uns erstmal die beste Lösung das zu realisieren, dass wir überhaupt von den handschriftlichen Laborbüchern einfach wegkommen.	
Interview 30	5	Und genau. Und da geht es eben darum wie bei uns dann auch die Server Daten abgelegt werden,. welche zusätzlichen Metadaten abgelegt werden sollen, wohin archiviert wird, was wir mit Langzeitarchivierung erreichen möchten und eben Schulungen in dem Bereich durchführen.	
Interview 30			Und in welchen Data Sharing Aktivitäten seid ihr involviert?
Interview 30		Data Sharing ist ja erstmal schwierig zu umreisen. Es gibt, denke ich, mehrere Arten des Data Sharings. Einmal ist es klassisch eben das Publizieren in einem Journal, das ist ja auch Data Sharing.	
Interview 30		Dann ist es aber auch das zu Verfügung stellen der eigenen Forschungsdaten innerhalb der ganzen Gruppe, was eben normalerweise im Laborbuch dann gemacht wird oder halt auch dann in Perm, das ist ein Institut, was halt eben auf den Servern abgelegt ist, was digital ist, was in eigenen Datenbanken hinterlegt ist.	
Interview 30		Und dann gibt es halt eben für verschiedene Forschungsnetzwerke dann auch verschiedene Lösungen wie Daten ausgetauscht werden - sei es über irgendwelche Cloudlösungen oder ob man tatsächlich Daten einfach nur per Email hin und herschickt oder so. Irgendwie involviert in Data Sharing ist man immer.	

Interview 30	Das man die Daten nur für sich behalt funtkioniert halt nicht Gott sei dank, aber es ist halt die Frage, über welche Form des Data Sharings man halt an der Stelle spricht. Und dann, genau, gibt es ja auch noch die öffentlichen Datenbanken, dass man jetzt Proteinstrukturen zur Verfügung stellt oder so, aber da bin ich jetzt selbst nicht drin involviert.	
Interview 30		Das heißt, ihr seid dann natürlich intern dabei, ihr seid in Forschungsnetzwerken dabei - also intern und extern.
Interview 30	Ja	
Interview 30		Und das in öffentliche Datenbanken zu stellen ist dann eher nicht so euer Thema.
Interview 30	Es ist jetzt nicht direkt mein Thema, sagen wir so. Es ist halt ein bsi	
Interview 30	bisschen von den Projekten abhängig. Viel wird es gefordert, wenn man an einem geförderten Projekt arbeitet, dass im Anschluss die Daten der breiten öffentlichkeit zur Verfügung gestellt werden.	
Interview 30	Dann bedeutet es halt schon, das dann auch in öffentliche Datenbanken eingestellt werden müssen. Die Daten, die von mir persönlich generiert werden, die eignen sich nicht dazu, bisher in etablierte Datenbanken eingestellt zu werden.	
Interview 30		Und warum dann nicht?
Interview 30	Weil jetzt zum Beispiel primäre Screeningdaten - dafür gibt es einfach keine Datenbanken. Und es wäre wahrscheinlich einfach zu groß und zu viel Datenmüll, tatsächlich.	
Interview 30	Vermutlich, wenn, für mich persönlich wäre es schon interessant, wenn diese Primärdaten auch erreichbar wären. Bisher, in dem Bereich Publiziert wird sind wirklich validierte Hits, aber die ganzen Substanzen, die durchgetestet wurden und innerhalb des Prjektes irgendwann hinten über fallen, die werden halt nicht publiziert.	
Interview 30	Das heißt, die Daten sind nicht zugänglich.	
Interview 30		Also auf die Daten würdest du dann gerne zusätzlich zugreifen wollen?
Interview 30	Ja, manchmal wären solche Sachen spannend, also zum Beispiel wenn wir ein Test haben, also greifen halt wir und auch größere Pharma unternehmen auf die eigene Datenbank zurück. Wenn ich ein Screen durchführe, bzw ich habe mehrere Screens, die alle verschiedene Targets untersuchen aber mit einem ähnlichen Experiment ablaufen, da kann ich diese Daten eben	
Interview 30	auch untereinander vergleichen um auch zu sehen: OK, ich habe jetzt hier eine Substanz in meinem Test drin, die ist in allen diesen Testverfahren positiv. Das heißt, die geht wahrscheinlich eher auf mein Detektionsverfahren als dass es tatsächlich mein Target trifft. Diese Daten sind für mich sehr interessant.	
Interview 30	Das sind aber dann nur die Daten, die wir hier am Standort erhoben haben, auf die ich dann Zugriff habe. Das sind keine, da gibt es keine Datenbanken, auf die man zurückgreifen kann. Jetzt große Pharmaunternehmen, die haben natürlich die größere Masse an solchen Daten und die vergleichen sie halt auch innerhalb.	

Interview 30	10	Das sind halt auch schöne Daten um Untersuchungen zu machen, welche dieser, welche bekannten Essay Formate möglichst geringe Anfälligkeit haben für solche falsch positiven Ergebnisse oder halt auch andersrum: welche Substanzklassen sind häufig in welchen Verfahren als falsch positiv auftauchen.	
Interview 30			Jetzt hast du ja schon die FAIR Principles erwähnt und gerade die Problematik mit dem faksch positiv: da stellt sich mir die Frage: welche Anforderungen stellst du an die geteilten Daten?
Interview 30		Also man muss halt in allererste Linie einmal nachvollziehen köännen, wo diese Daten und wie diese Daten generiert wurden. Und das ist glaube ich ganz schwierig zu gewährleisten. Also du musst das halt, also du musst einmal das ganze Protokoll hinterlegt haben, was halt meistens irgendwie in schriftlicher Form als Text vorliegt.	
Interview 30		Also wirklich wahrscheinlich Stichpunkte: Zugabe von Lösung A, Inkubation über Zeitraum X und, naja, diese einzelnen Schritte eben. Und die sind halt sehr schwieirig dann mit den Daten wieder vernünftig zu verknüpfen. Aber das ist halt normalerweise das, also es ist eigentlich Experimentprotokoll, was die maximale Information enthält.	
Interview 30		Das ist aber halt schwierig zu interpretieren in der Methode, die auch, ja, machinell erarbeitet werden kann. Das heißt, die Daten müssen eigentlich, die müssen annotiert werden, es muss ein maximum an Metdaten, die zur Verfüfung stehen, das aber auch in einer verknüpften Form dann liegt, damit diese Metadaten auch schnell rausziehen kann, die Metadaten, die mich dazu interessieren.	
Interview 30		Ohne die dazugehörigen Metadaten eist halt ein Datenpunkt relativ unbrauchbar, weil ich dann die Nachvollziehbarkeit nicht mehr habe. Weil ich nicht, welche genauen Schritte, welche Faktoren dazu gehören.	
Interview 30			Jetzt sind wir schon im Bereich und Schwierigkeiten: welche weiteren Probleme und Schwierigkeiten treten denn beim Verwenden der geteilten Daten auf?
Interview 30		Ja, eben, wenn es einfach ein einsamer Datenpunkt ist, der gegeben wurde, und ich nicht weiß wie er erhoben wurde, dann ist es schwierig, den zu interpretieren und wirklich zu sagen, dass das ein verlässlicher Datenpunkt ist.	
Interview 30		Man weiß häufig nicht, wo, welche, ich sage mal grob, welche Verfahren angesetzt wurden, aber man sieht einfach nicht genau, wo diese Daten jetzt herkommen. Dann ist es schwierig zu sagen, wie verlässlich der jetzt ist.	
Interview 30		Das ist halt die Verwendung von den geteilten Daten, ne. Das ist halt, aber ich denke, dass das im Moment nicht die große Hürde, das teilen von Daten. Es geht gar nicht um die Verwendung der Daten die da sind, weil es gibt einfach Datenbanken, auf die viele zugreifen, die sehr verlässlich sind, die sehr gepflegt werden, wo immer neue Daten eingespielt werden.	

Interview 30		Und je mehr Datenpunkten von unterschiedlichen Gruppen zu einer bestimmten Fragestellung eingestellt werden, um so verlässlicher kann nachher natürlich auch Aussagen darüber treffen. Die Schwieirigkeit ist halt die, die Leute zu motivieren ihre Daten auch wirklich zur Verfügung zu stellen.	
Interview 30	15		Genau, an dem Thema arbeite ich momentan.
Interview 30		Genau, weil es ist halt, es ist ein Aufwand, den man betriebt, der erstmal für einen selber keinen Nutzen generiert. Wenn ich meine Daten andere zur Verfügung stelle, dann habe ich halt einmal das Problem, dass einmal gesichtert werden muss, wo jetzt die IP liegt von diesen Daten, das muss halt klar sein, dass wenn jemand seine Daten zur Verfügung stellt.	
Interview 30		Es muss halt die Angst genommen werden, dass diese Daten dann genutzt, jemand anders darauf Profit schlägt und man selber leer ausgeht. Das ist einmal so eine große Hürde, warum Daten vielleicht nicht zur Verfügung gestellt werden.	
Interview 30		Und dann ist es ein riesen aufwand, die Daten aufzubereiten, so dass sie eben wiederverwendbar sind, dass sie ordentlich annortiert sind, dass die Metadaten da sind, dass sie in X reinpassen, dass das Format hat, dass für die Datenbank zuläsig ist. Das sind alles Schritte, die gegangen werden müssen, die Mehraufwand sind.	
Interview 30		Das ist das, was nicht alltäglich ist, wie mit den Daten umgegangen wird. Wenn man da eine Umgebung schafft, wie an einem Standort an sich die Daten schon geteilt werden, dass das in einem Format ist, dass das auch global genutzt werden kann, dann gehe ich hier einen Schritt weiter.	
Interview 30		Das ist einfach, das es vereinfacht, die Daten darszustellen, die Daten zu verknüpfen und die Daten wieder auffindbar zu machen.	
Interview 30			Das heißt welche Beweggründe siehst du jetzt konkret, Daten mit anderen zu teilen?
Interview 30		Das ist einfach der Schritt in die Zukunft. Das funktioniert heute nicht mehr, dass jeder im Labor und im stillen Kämmerlein seine Forschung für sich macht. Jeder versucht für sich selber die Fragen neu zu erfinden.	
Interview 30		Die Daten zu teilen, das ist der nächste große Schritt, weil ich meine Daten zur Verfüfung stelle und andere ihre Daten zur Verfügung stellen, dann erreichei ch darüber eine viel gesicherte Information über meine Fragestellung, kann viel mehr Rückschlüsse daraus ziehen.	
Interview 30		Man kann über Informationstechnologien die Daten besser auswerten, neue Rückschlüsse daraus ziehen.	
Interview 30		Das ist halt der neue Schritt, wohin die Forschung gehen muss - nicht mehr hier sein Protein auf seinem Ploten, GC davon machen und das Publizieren sndern wirklich schauen, was wird auf der Welt gemacht, was haben die Leute rausgefunden, neue Netzwerke zu knüpfen.	

Interview 30		Wie die ganzen Forschungsbereiche irgendwie zusammenhängen, und wie jeder Forschungsbereich von dem anderen lernen und Kooperatiionen daraus ziehen kann.	
Interview 30			Und inwieweit möchtet ihr die Data Sharing Aktivitäten ausbauen?
Interview 30		Ja, also einmal sind wir ja, wie gesagt, aktiv dabei, das ganze für uns sinnvoll auzubauen, weil wir halt einfach sehen, wenn wir intern keine Lösung haben, wie wir unsere Daten so aufbereiten können, dass sie auch nur im Ansatz der Fair Prinzipien folgen, können wir so auch nicht unsere Daten der breiten Masse zur Verfügung stellen, das ist im Moment unser erster Scritt, dass wir sagen, wir gehen jetzt Wege, wie wir innerhalb von unserem Standorts, dann später vom Gesamt IFG, da eine Schnittstelle finden können und versuchen auch zu verstehen, wie Programme arbeiten können, um diese Daten wieder lesbar zu machen.	
Interview 30		Das ist das eine. Dann eben unsere Arbeiten innerhalb des NFDI Konsortiums, wo wir halt eben schon mit anderen Instituten im Gespräch sind, wo wir das ganze Vernetzen möchten.	
Interview 30		Und das ist ja auch ein ganz wichtiger Schritt, dass hier eben versucht wird, Netzwerke aufzubauen, die völlig losgelöst sind von verschiedenen wissenschaftichen Disziplininen. Das da einfach etwas aufgebaut wird, das fachbereichsübergreifend funktionieren kann. Aber wir sind halt noch ganz am Anfang.	
Interview 30			Das heißt, welche Daten werden denn geteilt mit anderen? - sei es im Konsortium und in anderen Forschungsnetztwerken.
Interview 30		Das ist bei uns ziemlich Projektabhängig. Also wir arbeiten, ja, viel auch im Bereich Auftragsforschung. Da ist es eben so, dass diese Daten sowieso nicht uns gehören.	
Interview 30		Das heißt, wir machen hier unsere Untersuchungen. Diese Ergebnisse werden dann an die Arbeitsgruppen weitergeleitet. Das kann halt über verschiedene Wege	
Interview 30	20	funktionieren. Dann arbeiten wir halt mit verschiedenen EU geförderten Projekten, zum Beispiel, wo dann auch Anforderungen sind, dass diese Daten in welchen Formen auch immer, anschließend zur Verfügung gestellt wird. Wo dann Strukturen aufgebaut werden müssen, wo diese Daten geteilt werden - sei es jetzt innerhalb eines Konsortiums oder öffentlich.	
Interview 30			Das mit der Auftragsforschung finde ich interessant. Da frage ich mich, werden dann immer alle Daten ausgetauscht oder wann werden keine Daten geteilt?
Interview 30		Also innerhalb der Auftragsforschung entscheidet im Endeffekt ja der Kunde, welche Daten er haben möchte. Das sind seine Daten. Die können wir dann auch nicht weiter an anderer Stelle nutzen oder herausgeben.	

Interview 30	Wir behalten uns allerdings vor, eben unsere eigenen Rückschlüsse daraus zu ziehen aus diesen Daten. Also Sprich jetzt wie eben das Beispiel, dass ich mir eben angucke, ob die Substanz in einem anderen Testverfahren auch schon positiv war. Dann kann ich das auch aus diesen Daten, wenn diese Daten weitergereicht werden, auch herauslesen.	
Interview 30	Weil mich in dem Fall nicht das eigentliche Zielprotein hinterher interessiert sondern nur der Detektionsweg. DAs ist aber in den unterschiedlichen Verträgen mit den Auftraggebern abgeklärt, welche Daten wie von wem genutzt werden können. Und da ist es eben unterschiedlich, welche Daten der Auftraggeber hinterher haben möchte- ob er nur die prozessierten Daten haben möchte, ob er alle Daten haben möchte und halt auch in welcher Form diese Daten dann übergeben werden.	
Interview 30	Also wir hatten dann auch schon den Fall, dass jemand gesagt hat, dass jede Form von Weg über das Internet zu riskant ist und wir haben dann Festplatten mit der Post verschickt. Also das ist jetzt auch schon ein paar Jahre her, das sieht er vielleicht dann auch anders, aber es dann halt eben, es ist unterschiedlich.	
Interview 30	Andere sagen: ja schicke mir das als PDF in einer Email oder, ja. Das wird dann halt jeweils abgesprochen. Auf jeden Fall sind wir Auftragnehmer und machen eine Dienstleistung und dann wird das vorher ausgehandelt, wie das zu funktionieren hat.	
Interview 30	Es sind dann nicht unsere Daten. Das heißt, wir können nicht darüber entscheiden, ob diese Daten dann weiter an anderer Stelle zur Verfügung gestellt werden.	
Interview 30		Um in ein anderes Zukunftsszenario zu gehen: wie stellst du dir denn den idealen Zugriff auch benötigte Daten vor?
Interview 30	Idealerweise gibt es, denke ich, eine Art nennen wir es mal Portal, so grob versteht da auch jeder etwas anderes drunter unter diesem Begriff. Aber ich denke, dass es schön wäre, eine Möglichkeit zu haben, Daten zu sichten ohne jede, ohne hunderte von verschiedene Datenbanken zu kennen, diese Abfragen zu müssen und das ganze wieder miteinander verknüpfen zu kennen.	
Interview 30	Also das es halt über eine Cloud Computing Lösung funktioniert. Vielleicht muss ich mich da einloggen, vielleicht hat da aber auch jeder Zugriff drauf, dass ich da sagen kann: OK, ich interessiere mich für den und den Pathway und diese Substanz involviert ist und kann mir dann alle Informationen zusammensuchen, die da hinterlegt sind.	
Interview 30	Sei es Proteinstrukturen, sei es die Information, die über die Substanz vorhanden ist. Wenn das ein Medikament ist zum Beispiel wie das eingesetzt wird, wo es wirkt, welche Nebenwirkungen darüber auftreten und auch welche Kinetischen Daten bei der Interaktion entstanden und ob das irgendwie ein Umweltgift und da irgendwas beachtet werden muss.	

Interview 30		Also diese Daten alle miteinander verknüpfbar sind bzw nicht verknüpfbar sind sonder alle miteinander verknüoft sind, sorum. Weil es ist im Moment noch der Fall, dass man selber halt die einzelnen Daten, also einmal wissen muss, wo sind Daten hinterlegt, wie kann ich diese Daten für mich zugänglich machen, dann brauche ich	
Interview 30	25	verschiedene Softwarelösungen, um die Daten bearbeiten zu können. Und diese Daten dann wieder verknüpfen und daraus eben neue Rüchsclüsse ziehen zu können. Wenn mehr Daten zugänglich und ein Netzwerk diese Daten entstanden ist, denke ich sind wir da einen ganz großen SChritt weiter.	
Interview 30			Und welche Probleme und Barrieren sorgen dafür, dass es momentan nicht so ist?
Interview 30		Es ist wirklich schwierig, die Daten sinnvoll zu benennen, sprich ich muss ja für den Datenpunkt, den ich erhoben habe, dann im Endeffekt die zusätzlichen Informationen damit verknüpft haben.	
Interview 30		Und diese Verknüpfung aufzubauen ist gar nicht so einfach, wie man sich das vorstellt. Auch einmal die Begrifflichkeiten zu definieren, also wenn ich jetzt, also stell dir einfach ein einfaches experiment vor, zum Beispiel du machst eine PCR. Dann weisst du, OK ich muss irgendwie noch hinterlegen, welcher Primer dazu gemacht wurde, wie viele Zyklen du gemacht hast, bei welcher Temperatur du gearbeitet hast, welcher Puffer ladiladida, dann am besten welcher Pufferhersteller, welche Batch war es gewesen.	
Interview 30		Man hat halt einfach eine Reihe von zusätzlichen Informationen und muss daraus erstmal selektiveren, welche davon sind wirklich relevatnt, weil alles wäre vermutlih zu viel.	
Interview 30		Und dann: wie bennen ich diese einzelnen Punkte? Und dann braichst du eine Softwarelösung, die diese einzelnen Punkte erkennt, die diese dann zusammen bündelt und dann sagt: Ok, das war jetzt eine PCR, damit man das ganze wiederauffindbar machen kann, und sagen kann:hier ist die PCR, hier ist der Betaplot dazu und hier ist das Experiment. Das glaube ich ist noch eine große Schwieirigkeit	
Interview 30		. Und eben, dass da verschiedene Fachbereiche aufeinander treffen.	
Interview 30		Du hast halt einmal die Leute im Labor, die diese Daten erheben, und dann hast du die IT Leute, die versuchen eben Daten in eine computer lesbare Form umzuwandeln, die aber halt auch einfach wissen müssen, welches sind die relevanten Punkte.	
Interview 30			Welche weiteren Probleme und Barrieren und Probleme sieht du denn beim Data Sharing?
Interview 30		Ich denke, ein großer Punkt ist, dass viele vielleicht nicht wissen, was der eigene Vorteil daraus wäre die Daten zu teilen. Und eben das es, das es halt eben ein zusätzlicher Aufwand ist, der betrieben werden muss.	
Interview 30			Wie ist es denn mit finanziellen Aspekten - spielen die auch eine Rolle?

Interview 30		Joa, mit sicherheit. Also ich weiß halt nicht in wiefern es im Moment ein finanzieller Vorteil sein kann. Jetzt mal abgesehen von Publikationen in irgendwelchen Journals. Immer wenn man veröffentlicht, man möchte, dass es gelesen wird, man möchte es als Möglichkeit sein als Mittel einzuwerben, aber ja, ich weiß nicht, was stellst du dir darunter vor?	
Interview 30		ALso meinst du, dass es für jemanden ein finanzieller Nutzen sein kann, seine Daten zur Verfügung zu stellen - oder die Daten verkauft werden? Oder wie?	
Interview 30			Ich habe jetzt daran gedacht, dass es ja auch sein kann, dass man gar nicht die ganzen Zugänge zu den Datenbanken bekommt, weil es Geld kostet.
Interview 30		Ja, das ist ja aber weniger eine Hürde, die Daten zu teilen als mehr geteilte Daten zu nutzen.	
Interview 30			Inwiefern?
Interview 30		Naja, wenn ich keinen Zugang zu den Datenbanke habe, komme ich an die Daten nicht ran, die dort bereits hinterlegt sind.	
Interview 30	30		Prinzipiel ist beim Data Sharing auch beides wichtig: also Daten zu teilen aber auch Daten zu nutzen. Weil wenn niemand Daten nutzt, die geteilt werden.
Interview 30		Ja, es ist auch das Vertrauen noch nicht da, dass die Daten ihre richtigkeit haben. Das kann auch meines Erachtens wirklich erst entstehen, wenn die Datenmenge einen gewissen Schwellenwert überschreitet. Also wenn ich halt einen simplen Datennpunkt finde, wo es dann heißt, was weiß ich: das Protein XY hat ein Gewicht von 87 Kilodalton, dann kann das ja sein, aber vielleicht ist da auch jemandem einen Fehler unterlaufen - vielleicht kann ich dem auch nicht trauen	
Interview 30		Und dann glaube ich für viele Leute noch so eine Schwierigkeit. Aber das wird halt entstehen, wenn mehr Daten da sind, wenn mehr Leute ihre Daten teilen, dann weiß man halt auch, wann diese Daten dann auch verlässlich sind und welcher Datenpunkt - wird sich halt dann - Datenpunkt ist nicht valide, indem man veröffentlicht, Datenpunkt werden dadurch, dass das von vielen Seiten gestützt, wird, wird der dann valide.	
Interview 30		Und im Moment hat man das eben noch nicht. Aber vielleicht ist das auch so wie mit Wikipedia, Als Wikipedia in seinen Anfängen stand hat haöt auch jeder gesagt: aja, da kann ja jeder reinschreiben, was er will.	
Interview 30		Mittlerweise ist es aber schon eine vertrauenswürdige Quelle, weil viel daran gearbeitet wird unv viel Input geleistet wird und gepusht wird. Das ist einfach jetzt, das ist einfach ein Prozess.	
Interview 30			Wird das Data Sharing denn in der wissenschaftlichen Community anerkannt?
Interview 30		Also in meinem Umfeld ja.	
Interview 30			Trägst dann auch die Unternehmenskultur dazu bei oder wie ist das?

Interview 30	ich denke mal, wer auch so ein, ja ich denke schon. also wir haben halt ein Interesse daran, mit vielen Daten zu arbeiten, Daten zu also Ohne interesse an geteilten Daten arbeiten zu können.	
Interview 30	Und dafür müssen wir auch unsere Daten teilen wollen, sonst funktioniert das nicht.	
Interview 30		Hat das Management da auch ein Einfluss drauf, dass Sie sagen, ihr sollt das jetzt teilen, oder wo kommt das her?
Interview 30	Das kommt halt auch ein bisschen von den Porjekten, die wir haben. Also, ich meine, deshalb bemühen wir uns, am NFDI teilzuhaben.	
Interview 30		Und wenn du darüber nachdenkst, so die ganzen Probleme, die wir jetzt schon angesprochen haben. Welches hat deiner Meinung nach den größten Einfluss auf das Data Sharing? Warum das nicht so gelebt wird wie man sich das vorstellt?
Interview 30	Wahrscheinlich ist es einfach die Kenntnis darüber, wie man die Daten sinnvoll teilt. Da musst du auch ein bisschen besser definieren, was deine Vorstellung ist des Data Sharings.	
Interview 30	Also Daten werden geteit. Daten werden schon immer in Publikationen geteilt. Das ist die Form des Data Sharings, auf die wir alle seit Jahrhunderten zurckgreifen, dass wir eben in die Journals reingucken und schauen: was haben andere Leute erreicht - das ist ja auch Data Sharing.	
Interview 30	Ja, also. Es muss halt eine neue Größe erreicht werden, XX. Ich meine, es ist ja schon ein Schritt weiter, dass man jetzt nicht mehr in eine Bibliothek geht und das Heft, Hournal aus dem Regal rauszieht und es vor sich legt, und, oder eine Kopie davon macht, darin rummaltsondern es ist schonmal alles digital da.	
Interview 30	Das ist schonmal ganz nett. Viele Journals haben auch viele, da sind halt eben viele zusätzliche Information zu einem Artikel hinterlegt, wo dann Daten wiederverwendet werden können.	
Interview 30	Es ist ja alles da, aber es muss halt, meines Erachtens noch viel stärker ausgebaut werden.	
Interview 30	Also es gibt Data Sharing, es ist nocht so, dass es nicht stattfindet. Aber es ist eben so, denke ich, einer der großen Aufgabe der Wissenschaft, dass das eben viel besser ausgebaut und viel besser vernetzt wird vor allen Dingen.	
Interview 30		Zum Thema ausbauen habe ich auch nochmal eine ganz andere Frage.
Interview 30	35	Weil ich habe mich im vorletzten Semester mit Big Data Analysen und Künstliche Intelligenz beschäftigt. Wie schätzt du persönlich den Einfluss der beiden Technologien auf den Forschungsalltag ein?
Interview 30	Das ist das, was jetzt der, den nächste XX wenn auch YY könnte. Also da, wenn sie das schaffen, dass sie - das ist ja das, warum die ganzen Daten vernetzt werden sollen, das eben dann hinterher eben diese Daten auch insgesamt betrachtet werden können.	

Interview 30	Das ist Big Data. Dafür muss ich die Algorithmen schaffen, dass ich mit machinellen Lernverfahren daraus neue Erkentnisse ziehe, dass die DAten vorhanden sind, aus den ich neue Fragestellungen generieren kann, neue Hypothesen aufbauen kann. Das schafft ja ein Wissenschaftler heute nicht mehr, diese MAsse an Daten zu generieren und zu verarbeiten.	
Interview 30		Also glauben Sie dann, dass Big Data und Künstliche Intelligenz in den Forschungsalltag eintreten können.
Interview 30	Muss! Wird! Ja, das hoffe ich stark.	
Interview 30		Das klingt sehr überzeugt.
Interview 30		Ja, da bin ich auch noch voll gespannt, was da passieren wird.
Interview 30		Hast du denn noch weitere Aspekte zum Thema Data Sharing, Verwenden oder Daten zu teilen?
Interview 30	Ne, ich glaube, ich habe schon viel gesagt.	
Interview 30		Ja; du hast mir viele Einblicke ermöglicht und nochmal einen ganz anderen Blick darauf geworfen. Das fand ich sehr schön.
Interview 30	Ich denke, im Endeffekt wollen wir ja doch alle die Forschung vorantreiben. Und ich denke das ist halt der Punkt, wie wir das erreichen können. Klar, der rechtliche Punkt ist eine Schwieirigkeit, weshlab es schwierig ist, die Daten zu teilen.	
Interview 30	Es muss halt eben sichergestellt werden, dass immer nachvollziebar ist, wer diese Daten generiert hat. Weil sonst verlieren halt eben die Leute ihre Rechte an den Forschungsergebnissen oder den Gewinn, der daraus gezogen werden kann.	
Interview 30		Ja, da spielen viele Aspekte rein. Deswegen bin ich gerade auch auf einzelne Aspekte eingegangen weil ich wissen wollte, ob das für dich auch ein Thema ist oder gar nicht.
Interview 30	Ja, es ist halt eben dadurch, dass ich halt wirklich einfach schon von Anfang viel im Bereich Auftragsforschung gearbeitet habe, habe ich halt auch einfach nicht dieses persönliche Verhältnis zu den Daten, die ich generiere.	
Interview 30	Das ist halt, ich erhebe sie und verkaufe sie an dieser Stelle schon. Ich habe nicht die rechte an den Daten, die ich erhebe. Dadurch ist das für mich nicht so ein großer Aspekt, aber damit gehöre ich halt zu einem sehr kleinen Kreis in dem Bereich der naturwissenschaftlichen Forschung.	
Interview 30	Aber dadurch habe ich eben einen anderen Fokus vielleicht auch.	
Interview 30		Jetzt hast du ja das persönliche angesprochen.
Interview 30		Was glaubst du denn, welchen Einfluss das auf das Datenteilen hat?
Interview 30	Der persönliche Besitz, meinst du?	
Interview 30		Ja, genau. Oder welche persönlichen Einstellungen man dazu hat.

Interview 30		Ja, das ist ja wie in allen Bereichen, ist das das, was uns antreibt. Wenn ich halt denke, die Daten, die alle anderen erhoben haben, sind sowieso totaler Quatsch.	
Interview 30		Dann bin ich nicht ein Mensch, der seine eigenen Daten teilt oder die Daten den anderen Leuten zur Verfügung gestellt haben und nutzen möchte.	
Interview 30		Wenn man aber davon getrieben ist, dass irgendwann alle Daten miteinander verknüpft sind und vieles noch am Computer stattfindet, dann will man diesen Prozess auch unterstützen.	
Interview 30		Und dann ist man vielleicht auchein bisschen offener für die Wege, die es da gibt. Und möchte sich auch da mit einbringen. Also da gibt es eben von bis.	
Interview 30	40	Und, also, ja. Es gibt die Leute, die ihre Daten nur für sich haben wollen, die vielleicht auch schlechte Erfahrng gemacht haben, dass dann quasi DAten in anderer Hand weitergearbeitet wurden und dann leer ausgegangen ist - kommt vor. Dann ist man da halt etwas protektionistischer.	
Interview 31	Time	Interviewee 31	Interviewer
Interview 31			Ja, freut mich, dass das jetzt klappt mit dem Interview. Und dass Sie die Zeit dafür nehmen.
Interview 31		Jap.	
Interview 31			(...)
Interview 31		(...)	
Interview 31		Ich hoffe, ich darf mal eben. Ich mache jetzt auch ein Datenschutzthema. Also, ich sage Ihnen halt einfach was ich, was ich weiß oder was ich sagen kann und hoffe, dass das dann bei Ihnen von Interesse ist, ja?	
Interview 31		Ja, ja.	Ja, alles klar. Reden Sie da ganz frei und offen. Es werden 15 Fragen kommen.
Interview 31		Ok.	
Interview 31			Noch von vorne weg möchte ich einmal um ihre Einverstädnis bitten.
Interview 31		Sie nehmen es jetzt nicht akustisch auf, oder? Schreib... Oder wie machen Sie das? Schreiben Sie was? Wie geht das jetzt?	
Interview 31			Genau, dazu komme ich gerade. Ich würde es gerne aufzeichnen. Das kann man direkt auch hier über Skype für Business.Ich starte mal die Aufnahme unter ihre[m] Einverständnis, natürlich.
Interview 31		Ja, ja, gut. Alles klar. Das ist nur gut, dass wenn man das dann weiß. Ja genau. Sehr gut, ja.	
Interview 31			OK. [mh] Weil ich muss das ja alles transkriberen.
Interview 31		Ja, genau. Aber danach löschen sie es die Aufnahme, wenn Sie es transkribiert haben, oder wie ist das jetzt?	
Interview 31			(...) ja.
Interview 31		Ok, alles klar.	
Interview 31			Ok. Mal gucken: "Aufzeichnungen starten". Wo geht das denn? [...]
Interview 31			Also nochmal ganz kurz: Es werden 15 Fragen jetzt kommen. Es kann auch eine oder zwei mehr sein. Je nachdem was sie so antworten, was dazu passt. Die Dauer ist auf eine dreiviertel Stunde jetzt ausgelegt.

Interview 31		Ok, aber das schaffen wir. Weil ich habe auch ein paar Minuten danach noch Zeit. Also ich, bin, habe jetzt keinen (-)	
Interview 31			OK, sehr gut. Die Einstiegsfrage, mit der ich beginne, ist: Was gehört zu Ihren täglichen Aufgaben dann bei (Firma)?
Interview 31		Ok, mmh. Ja, ich bin hier im Grunde, wie man im deutschen Jargon sagt, mh Abteilungsleiter. Und mh ja, habe eine Abteilung.	
Interview 31		Und eine Abteilung besteht aus mehreren chemischen Laboren, die typischerweise aus Laborleiter, Laborleiterinnen und typischerweise so zwei bis drei Laboranten bestehen.	
Interview 31		Und dann gibt es auch noch 'ne Analytikabteilung. Und dann eine Abteilung, die sich mit 'ner, mit Up-Scaling oder eine Gruppe, die sich mit dem Up-Scaling beschäftigt.	
Interview 31		Und ich sage mal so, meine Tätigkeiten ist immer so in dem Spannungsfeld zwischen Personalmanagement - also da geht es natürlich um die Frage, wie wird jetzt so ein Laden gemanaged und geführt, auf der einen Seite. Und auf der anderen Seite natürlich auch auf der wissenschaftlichen Ebene Projekte mitbegleiten, zu coachen, mal Fragen zu stellen oder auch Anregungen zu geben wie Projektteams weiter arbeiten können.	
Interview 31		Also eigentlich immer so im Spannungsfeld Forschungsmanagement und und, und dann so auch immer so Seiten.	
Interview 31			Ok, super. Ich bin ja damals auf Sie zugekommen, weil sie meinten: "Data Sharing, das geht nur zusammen."
Interview 31		Genau!	
Interview 31	05:07		Jetzt interessiert mich: an welchem Data Sharing Aktivitäten sind Sie denn so involviert?
Interview 31	05:14	Wir haben verschiedene Sachen. Wir haben, sind und waren involviert muss man sagen.	
Interview 31		Wir arbeiten natürlich auch als pharmazeutische Industrie. Man muss immer mehr auch mit anderen zusammen in ganz unterschiedlichen Formaten.	
Interview 31		Das geht, sagen wir mal jetzt, von einer Contract Research Zusammenarbeit wo man Firmen hat, die Synthesen machen oder auch bestimmte Analytik Aufträge machen. Natürlich kann es auch in Richtung Biochemie oder Pharmakologie gehen, aber das ist dann vielleicht mal raus, weil hier geht es primär nur um die Chemie. Das ist so die eine Seite des Spektrums, sage ich mal.	
Interview 31		Das kann dann entweder in so einer Fee-for-Service Geschichte sein. Das heißt man sagt eigentlich "Ok, ihr synthetisiert mir dieses oder jenes Molekül und dafür kriegt ihr dann so und so viel". Oder da wie man so schön sagt FTE. Also Full Time Employer basierten Format wo man sagt: "OK für eine bestimmte Zeit arbeiten jetzt so und so viel Leute bei euch für uns". Und dann ist es natürlich immer ganz unterschiedlich, was die genau machen. Das nennt man dann FTE Modell, das es auch auf dieser CAO Seite und das geht natürlich dann bis zu, auf der anderen Seite, so hoch integrierten Kollaborationen, die wir haben.	

Interview 31		Zum Beispiel jetzt eine Kollaboration mit dem Broad Institut in (Ort), wo es eine Kollaboration dann auf allen Ebenen, der chemischen und biologischen pharmakologischen Ebene, stattfindet und man wirklich auch gemeinsame Projekte betreibt.	
Interview 31		Oder zum Beispiel, jetzt hier im deutschen Kontext, eine Kollaboration, die wir mit dem DKFZ im Deutschen Krebsforschungszentrum haben, wo wir auch gemeinsame Projekte mit dem Deutschen Krebsforschungszentrum vorantreiben.	
Interview 31		Und ich sage mal, eigentlich in allen diesen Fällen, also von diesen, ich sage mal, CAO Modellen bis hin zu diesen hoch integrierten Forschungskollaboration-Modellen, geht es natürlich darum auch Daten auszutauschen, Daten zu sharen. Das ist im Rahmen von Kollaboration.	
Interview 31	12:31		Und darauf aufbauend stellt sich die Frage: Auf welche geteilten Daten würden Sie noch gerne zusätzlich zugreifen, wenn sie es könnten?
Interview 31	12:40	Ja, das ist auch eine gute Frage. Ein Punkt ist ja auch - und da haben wir uns auch im AI Kontext getroffen - ist ja natürlich was wir auch machen.	
Interview 31		Wir haben jetzt ein Projekt der Pharmakovorinformatik, wo wir, ich sag mal, alle diese pharmakologischen, biologischen und chemischen, biologisch-chemischen oder VisChem Daten eben zusammengefasst haben in einer großen Datenbank, sodass im Grunde der Wissenschafter die Möglichkeit hat, zu den aktuellen Verbindungen immer den kompletten Datensatz, der früher erzeugt wurde und der jetzt ganz aktuell ist, herauszuziehen.	
Interview 31		Das ist so eine Art wie man die Daten nutzt. Man kann also direkt ansehen, was gibt es für aktuelle Daten. Dann werden aber natürlich diese Daten auch genutzt für, zum Beispiel, irgendwelche Machine Learning Algorithmen, die jetzt aus diesen Daten versuchen zu lernen, wie man jetzt vielleicht zukünftigte, also die Eigenschaften von zukünftigen Molekülen berechnen kann. Also solche Property-Predictions-Tools.	
Interview 31	12:51	Und das ist etwas, wo man sehr stark auf die eigenen Daten zugreift.	
Interview 31		Aber ich denke, das ist etwas worüber wir uns auch schon Gedanken gemacht haben - auch schon mit anderen, was natürlich interessant wäre, wenn man jetzt solche Lern-Algorithmen vielleicht auch in einem größeren Kontext betreiben könnte.	
Interview 31		Wo man vielleicht auch auf die Daten von anderen zugreifen kann, vielleicht auch in der Form, wo man dann gar nicht das direkt sieht, was vielleicht von der Intellectual Property Seite her vielleicht nicht sichtbar gemacht werden werden sollte aber wo man diesen Lerneffekt eben aus diesen externen Daten ziehen kann.	
Interview 31		Also ich sag mal so eine Art gemeinsamen Data-Lake bilden würde. Mit diesem Data Lake Dinge eben lernt und damit weiter arbeitet.	

Interview 31	13:54	Ich glaube, dass wäre aus meiner Sicht ein interessantes Modell, das man auf bestimmten Gebieten angehen könnte, so dass dann vielleicht die verschiedenen Parteien, die eben in den Data Lake Daten reingeben, die gar nicht Individual Daten sehen können sondern nur aggregierte Daten aus dem Lake, aber aus diesem Lake sozusagen lernen können. Also das wäre meiner Meinung nach noch anwendbar.	
Interview 31	14:57		Das wäre auf jeden Fall ein interessantes Konzept, da gebe ich Ihnen Recht. Jetzt haben wir ja schon viel darüber gesprochen wie viele Daten sie denn verwenden. Welche Anforderungen stellen Sie denn an die Daten die sie verwenden?
Interview 31		Ja, gut. Ich sag mal so, klar ist, dass man natürlich immer versucht Daten mit einer bestimmten natürlich einer möglichst hohen Qualität zu haben oder zu verwenden.	
Interview 31		Das ist natürlich ein Anspruch. Das heißt wenn man sagt: Okay ich will natürlich eigentlich nur Daten, die eine bestimmte Qualität zum Beispiel Pharmako, biochemische Daten, pharmakologische Daten, genetische Daten Drug Metabolism Daten haben, die ein bestimmtes Qualitätslevel erfüllen.	
Interview 31	15:17	Das ist natürlich ein ganz wichtiger Punkt, der übrigens gar nicht so einfach zu definieren ist. Ich denke, was ein genereller Punkt ist, das kam auch übrigens in der Diskussion vor, die wir da in Aachen hatten, ist was oft nicht so reported wird, sind eigentlich Sachen, die nicht geklappt haben.	
Interview 31		Ich finde, das ist doch eigentlich auch etwas, was jetzt im ganzen Deep Learning und AI Kontext etwas ist, was von Interesse sein könnte - dass man eben auch mal Sachen macht, die nicht funktioniert haben.	
Interview 31		Gerade auch bei beispielsweise irgendwelchen Reaktionen. Und das ist eigentlich etwas, was man typischerweise meistens bei den In-House Daten dann hat, welche Sachen nicht geklappt haben. Zum Beispiel jetzt bei so einem Laborjurnal hat man auch bei den In-House Daten auch die Dinge, die nicht funktioniert haben, aber bei den externen Daten hat man das nicht.	
Interview 31		Also da haben Sie vielleicht nochmal einen Rückgriff auf Ihre Frage von vorher. Ich glaube, es wäre auch interessant bei den externen Daten, wenn man da auch mehr Dinge hätte, die nicht geklappt haben. Und warum das natürlich nicht der Fall ist, weil oft es so empfunden wird dass wenn etwas nicht geklappt hat ist es dann ein schlechtes Datum.	
Interview 31		Aber ich glaube, da stellt sich jetzt zunehmend mehr die Frage, ob das wirklich so ist. Ob es dann wirklich ein schlechtes Datum ist, oder ob jetzt, ich sag mal, auch solche negativ Ergebnisse auch ein interessantes Datum sein könnten.	
Interview 31	16:06	Wobei man natürlich nie weiß, liegt es jetzt daran, ob es schlecht gemacht ist oder ob es einfach generell nicht geht.	

Interview 31	12:11		Für mich persönlich ist es immer wichtig, dass man sagt: Okay, es kann was nicht klappen und es wird auch alles nicht klappen - und das ist genauso Wissen, was man sich angeeignet hat wie Dinge, die halt funktioniert haben
Interview 31		Ja, genau.	
Interview 31	17:24		Dann haben Sie ja schon gesagt dass sie auch versuchen, die Daten von anderen mit ihren eigenen zu kombinieren. Welche Probleme treten denn auf, wenn Daten von anderen verwendet werden?
Interview 31		Andere ist natürlich ein weites Feld. Sagen wir mal so, da gibt es natürlich multiple Phänomene.	
Interview 31	17:40	Ich erinnere mich zum Beispiel, wir haben mal ein Projekt gehabt. Ein bisschen kann ich das glaube ich ja auch erwähnen, wo wir versucht haben auch jetzt zu gucken welche Moleküle sind eigentlich mit welchen Aktivitäten publiziert.	
Interview 31		Und ein Problem ist dann dass sie natürlich zum Teil mitunter sehr unterschiedliche Arten und Weisen haben wie jetzt biologische Aktivitäten reported werden.	
Interview 31	18:00	in manchen Publikationen steht da drin very activ /not activ oder so etwas. ja so ganz, Ich sage mal, wirklich so ganz qualitativ. In anderen Publikationen, das ist das eine Extrem, in anderen Publication ist es so ganz lege artis [Anmerkung: nach dem Stand der Wissenschaft].	
Interview 31		Da hat man die richtigen IC50 Werte, die richtigen IC50 Kurven, dann wahrscheinlich so einen Mittelpunkt ist oft, dass man sagt; active at, concentration from, oder not active at concentrationon from, also dass man dann so eine semi-quantitative Beschreibung hat.	
Interview 31		Übrigens ganz typischerweise auch oft die Sache mal ein Thema in Patenten, wo es sehr unterschiedliche Stile gibt. In manchen Patenten werden biologische Daten sehr genau reported, in anderen gar nicht so genau.	
Interview 31		Das heißt die unterschiedlichen Formate in denen zum Beispiel biologische Aktivitäten reported werden, das ist sicherlich ein Thema und eigentlich wenn man jetzt, sage ich mal, moderne Tools hat, das zu einem Gesamtbild zusammenzufügen ist das sicherlich etwas was hilfreich ist.	
Interview 31		Gut, Ich sage mal so was natürlich generell der Fall ist, ist, dass sehr viele Sachen sich nicht reproduzieren lassen können aber da haben wir auch ein Kollege von uns, der hat da einmal eine Arbeit drüber geschrieben vor fünf oder noch mehr Jahre her die wurde dann auch viel beachtete sogar mal im Economist irgendwie auch länger drüber abgeschrieben worden.	
Interview 31		Wo wir eben haben gesagt haben, wir können sehr, sehr viele Dinge, übrigens auch in High Impact Journalen, gar nicht reproduzieren biologischer Ergebnisse.	
Interview 31	18:36	Das ist das natürlich auch ein grundsätzliches Problem, was man hat. Aber das hat nicht unbedingt etwas mit Daten, aber sicherlich etwas mit der Qualität der Daten oder auch vielleicht mit der Genauigkeit mit der Dinge beschrieben werden, zu tun.	

Interview 31	19:59		Um in ein Zukunftsszenario zu gehen: Wie stellen Sie sich denn in Zukunft den idealen Zugriff auf benötigte Daten vor?
Interview 31	20:10	Meinen Sie jetzt intern oder extern, oder ganz generell?	
Interview 31	20:14		Generell
Interview 31		Oh, ja. Ich glaube dass wir insgesamt auf der einen Seite zu einer höheren, als wissenschaftlichen Community sag ich mal - und das gilt dann sicherlich auch innerhalb von Unternehmen, zu einer höheren Daten-Transparenz kommen sollten. Es muss aber dann natürlich mit einer größeren Genauigkeit, wer darf was sehen, verknüpft sein.	
Interview 31		Ich finde es klingt wie ein Gegensatz. Ich glaube, das ist aber kein Gegensatz. Ich glaube, wenn man klarer weiß, wann wer was sehen kann oder nicht - und ich meine "sehen" aus vielleicht patentrechtlich Gründen oder vertraglichen Gründen.	
Interview 31		Wenn man das genauer spezifizieren kann, dann kann man ja auch mit den anderen Daten freizügiger oder offener und transparenter umgehen. Ich glaube, dass das etwas ist, wo die modernen Daten-IT-Systeme einfach noch mehr beitragen können. Und das hat sicherlich auch ganz entscheidend etwas zu tun mit Interfaces.	
Interview 31		Wir haben sehr viele Daten in unterschiedlichen Daten-Silos, unterschiedlichen Datenbanken mit unterschiedlichen Formaten, unterschiedlichen Zielrechnungen.	
Interview 31		Ich glaube die wesentliche Aufgabe ist es, jetzt auch diese Dinge zusammenzubringen, sodass man da auch einen gesamtheitliche Blick auf diese Dinge entwickeln kann ohne allzu großen Aufwand betreiben zu müssen.	
Interview 31		Und ich glaube, wenn man so eine Situation hat, dann kann man auf der einen Seite in Kollaborationen auch noch mehr schneller austauschen. Auf der anderen Seite aber vielleicht auch noch mehr schneller mit Externen einfach teilen.	
Interview 31	20:14	Und dass wir da einfach, ich sag mal so, mehr Dinge teilen, auch das ist so ein Trend, aber natürlich auf einer rechtlich fundierten Grundlage.	
Interview 31	22:20		Und zwar mit den Daten teilen, das haben Sie auch gesagt in Aachen, das geht nur zusammen. Da würde ich gerne noch mal ihre Beweggründe erfahren, die sie haben, diese Daten zu teilen.
Interview 31		Gut, ich sag mal so, das ist jetzt auf unterschiedene Ebenen. Wenn ich eine Kooperation habe, dann ist es klar, wenn man so will in Anführungsstrichen, eine Schicksalsgemeinschaft oder Volksgemeinschaft.	
Interview 31		Man möchte gerne zusammen etwas erreichen und je mehr, je besser man sich austauscht und je mehr - so wie es halt ist in einem guten Team, wie bei einem guten Fußballteam oder beim Tennis Doppel oder so - je mehr man auch, ich sage mal, orchestriert aufeinander abgestimmt arbeitet, miteinander arbeitet umso besser wird im Zweifel ja auch das Ergebnis sein.	
Interview 31		Das heißt, in so einer Kooperation ist ganz klar, da ist natürlich ein intrinsischeInteresse daran, all die Daten, die ich brauche um gemeinsam erfolgreich zu sein, auch zu teilen.	

Interview 31	22:38	Da ist es auch oft einfacher. Wenn man natürlich jetzt den nächsten Schritt macht auf die nächste Ebene, also ich sag mal so, in der Public Domain daran denkt, gut.	
Interview 31		Da gibt es natürlich dann, wahrscheinlich zum einen, ähnliche Beweggründe. Das heißt, grundsätzlich ist es natürlich so, je mehr Leute dann auch mehr Daten teilen, desto mehr können alle auch lernen, ich sag mal so, das ist ja auch die Motivation von Patenten letzten Endes.	
Interview 31		Und das natürlich im Interesse aller, wobei das ist natürlich nicht ganz so direkt, deswegen ist es manchmal etwas schwerer diesen Schritt zu tun. Aber das hat sicherlich auch inzwischen was mit Branding zu tun.	
Interview 31		Ich glaube, dass - man sieht das einfach - dass Firmen [die jetzt Firmen] oder Unternehmungen oder Institute oder whatever, die in der Lage sind oder die bereit sind, Daten, die von Interesse sind, zu teilen.	
Interview 31	23:34	Das ist ja dann auch etwas, was sich sozusagen positiv auf ihr Bild in der Öffentlichkeit, ihr Bild in der wissenschaftlichen Community zurecht auswirkt	
Interview 31	24,19	Und das kann auch eine Motivation sein Daten zu teilen.	
Interview 31		Und Ich denke, zwischen diesen beiden Extremen, auf der einen Seite vielleicht so das öffentliche Bild, auf der anderen Seite auch noch der Erfolg in der wissenschaftlichen Community oder in einer Kollaborationen, spielt es immer mit diesen verschiedene Motivationen.	
Interview 31	24,24	Auf der einen Seite wissenschaftlicher Fortschritt und dann sicherlich irgendwo auch etwas zur Allgemeinheit beizutragen.	
Interview 31	24,44		Und jetzt darauf anknüpfen das Gegenteil: Welche Probleme und Barrieren sehen Sie da momentan gerade?
Interview 31		Gut, es gibt natürlich eine Menge. Es gibt natürlich "natürliche Barrieren" und es gibt natürlich auch potenzielle Probleme oder, sagen wir vielleicht mal, Risiken, ganz klar. Ein Thema ist, natürlich in bestimmten Bereichen, so auch zum Beispiel in der pharmazeutischen Industrie oder sagen wir mal in der Chemie, ist es natürlich so, dass die ganzen Businessmodellen letzten Endes darauf fußen - oder viele Business-Modelle, vielleicht nicht alle, dabei so typischerweise von gewinnorientierten Unternehmen, was ja zum Beispiel eine Aktiengesellschaft per Definition ist oder auch sein muss, auch wissen, - dass man eine gewisse Zeit mit einem Patentschutz arbeitet. Warum?	
Interview 31		Weil man natürlich am Anfang ein sehr sehr hohes Investment tätigt. Und das lässt sie nur dann wieder rein spielen - das ist sicherlich nochmal eine ganz andere Diskussion, aber grundsätzlich ist das die Überlegung - letztlich dann nur wieder rein spielen, wenn man eben auch eine Zeitlang mit einer gewissen Exklusivität arbeiten kann.	

Interview 31		Und das ist natürlich auch der (Anm: das), dann letzten Endes wie das im Patentrecht heißt Verbietungsrecht gegenüber den anderen da, zur Anwendung bringt, nämlich wenn man das Patent hat, hat man ja Verbietungsrecht den anderen gegenüber. Und das wäre die Motivation diese Dinge zu veröffentlichen und das ist die Grundlage für den Business Case.
Interview 31		Das heißt, wenn sich jetzt etwas [mmh] eine Grundvoraussetzung für Patent ist ja eben die Neuheit, dass es noch gewerbliche Nutzbarkeit, Neuheit - und Nutzbarkeit ist meistens nicht entscheidender Punkt.
Interview 31		Aber wenn ich Dinge veröffentliche, dann sind sie, wie der Patente Anwalt dann so schön sagt, Neuheit schädlich getroffen. Das heißt, ich kann das dann eben nicht mehr, oder eben nur noch sehr eingeschränkt, zumindest mal die Struktur, patentieren.
Interview 31	24,52	Das heißt, dadurch gebe ich mir die Option zur Patentierbarkeit. Und das ist natürlich etwas was jetzt gerade im Kontext mit chemischen Strukturen ganz ganz wichtig und evident ist.
Interview 31		Das gilt im übrigen auch für chemische Verfahren. Da geht es ja dann [mmh] sodass man da eben typischerweise und in traditioneller Weise vorsichtig ist Strukturen, chemische Strukturen, zu früh zu veröffentlichen.
Interview 31		Das ist das eine Thema, das ganze Thema Intellectual Property IP.
Interview 31	26,56	Ein anderes Thema ist das natürlich, dadurch dass wir - wie vorhin gesagt, jetzt von dieser ganzen z.B. pharmazeutischen Industrie, Biotech, sicherlich auch in anderen Bereichen - immer mehr jetzt auch kollaboriert zusammenarbeitet, ist man auch in 'nem zunehmend komplexeren, vertraglichen Netzwerk
Interview 31		Ja. Das heißt, man muss, wenn man Daten veröffentlicht, auch erstmal überlegen gehören, die mir überhaupt alleine, oder oder gehören die mir gemeinsam mit einer anderen Partei, einem anderen Kollaborationensteilnehmer?
Interview 31		und darf ich die überhaupt veröffentlichen? So ein bisschen so wie wenn man jetzt irgendwelche Fotos ins Netz stellt oder so, nicht. Da geht's auch immer um die Frage, ob ich das Copyright darauf oder nicht? Oder habe ich es alleine oder nicht?
Interview 31		Und unter welchen Voraussetzungen kann ich sie veröffentlichen? Das ist das nächste. Und es geht natürlich auch wieder um das Thema Reputation. Ja, ich glaube, da ist es wahrscheinlich so...
Interview 31		Zum Beispiel jetzt bei diesen toxikologischen Daten: Ich glaube, das zeigt es gut. Ich glaube, dass zum Beispiel die pharmazeutische Industrie - da hat glaube ich, wenn ich mich richtig erinnere, die GSK oder BMS, ich glaube es war GSK, die ersten, die angefangen haben irgendwelche Studienergebnis ins Netz zu stellen.
Interview 31		Da hat man sicherlich vorher auch immer überlegt, naja, auf der einen Seite ist es natürlich gut, wenn man jetzt solche Daten ins Netz stellt. Das sicherlich zeigt Transparenz.

Interview 31	27,36	Auf der anderen Seite hat sich sicherlich der eine oder andere auch Sorgen gemacht, OK, wie man so weiß, nicht alle Experimente funktionieren und wenn ich jetzt auch, ich sage mal, negativ Ergebnisse ins Netz stelle, das kann ja dann auch wieder auf der kritischen Seite den Ruf beeinflussen.	
Interview 31		Ich glaube im übrigen persönlich, dass meistens eher da die positive Seite überwiegt. Aber das ist auch so eine Abwägung, die man dann sicherlich treffen muss und das ist sicherlich auch etwas das man jetzt lange in der Pharma [mmh] im Pflanzenschutz gemacht hatte.	
Interview 31		Ich meine, wo man auch sich überlegt hat, OK, welchen Einfluss hat das dann auf die Reputation? Wenn sie Daten veröffentlichen es ist ja auch oft so dass sie die eigentlich nur als Experte in seiner Gesamtheit verstehen können. Also ein Beispiel: Wenn Sie jetzt eine toxikologische Studie machen, dann ist es immer so, dass in der höchsten Dosis Gruppe - also ich vereinfache jetzt - wahrscheinlich irgendwelche Ratten oder Mäuse dann bestimmte Effekte zeigen.	
Interview 31	28,52	Und das ist dann ... kann ja auch sein im Sinne einer Toxikologie eigentlich ganz normal - zum Teil übrigens von Toxikologen sogar gewünscht, weil sie dann wissen Okay jetzt bin ich an dem Punkt wo ich wirklich 'nen negativen Effekt der Substanz habe, dann kann ich eben auch den Sicherheitsabstand zum nicht negativen Effekt ausrechnen.	
Interview 31		Wenn das jetzt aber ein Laie sieht, dann kann er natürlich ganz schnell denken: oh Gott, das ist ja ganz giftig, da sind irgendwelche Ratten zu Schaden gekommen. Und das heißt, sie haben dann natürlich schnell das Thema: Was wird mit solchen Daten gemacht wenn sie, ich sage mal, mal aus dem Zusammenhang gerissen werden, nicht.	
Interview 31		Das haben sie dann natürlich nicht mehr in der Hand wenn Sie die veröffentlichen. Ich glaube, da hat sich jetzt aber auch die Kultur gewandelt, dass an eben sagt, das ist dann eben so - eine freie Diskussion, da muss man einfach mal offener und transparenter an das Thema rangehen.	
Interview 31	29,51	Und eben dann auch aushalten, wenn es dann vielleicht auch mal nicht so kompetente Diskussionen gibt.	
Interview 31	30,29		Jetzt haben Sie ja schon die Kultur angesprochen. Wie ist das denn bei der Unternehmenskultur? Hat die auch einen Einfluss darauf, wie sie die Daten teilen?
Interview 31		Davon bin ich überzeugt. Das ist natürlich ein hoch komplexes Thema. Da bin ich natürlich ... absolut. Ich habe ehrlich gesagt - ich bin ja jetzt seit 20 Jahren, naja, noch nicht ganz, aber, sagen wir mal, fast 20 Jahren bei (Firma) .	
Interview 31		Ich bin in meinem 20ten Jahr bei (Firma) , sagen wir mal so, im 20ten Jahr bei (Firma) , und ich habe, ganz klar, jetzt in den 20 Jahren da auch ein Wandel der Kultur, was dieses Thema angeht, gesehen. Am Anfang war das sicherlich noch - und das war bestimmt nicht nur so bei (Firma) - die Vorstellung, man sollte viele Dinge, sagen wir mal, immer, im Zweifel eher, geheimhalten.	
Interview 31		Ich sage mal so, eben aus... wegen der Patentierbarkeit. Das ist etwas.	

Interview 31	30,40	Und jetzt wo wir in der Zeit leben, wo wir jetzt immer mehr kollaborieren - ich habe zum Beispiel bei (Firma) jetzt auch zwei prominente Beispiele genannt, hier DKFZ und BROAD zum Beispiel, auch viele andere Kooperationen natürlich mit irgendwelchen Biotech-Unternehmen - das ändert dann auch die Kultur.	
Interview 31		Und das zeigt dann, dass man auch mehr teilen muss. Und man sieht dann auch mehr den positiven Effekt vom Teilen von Daten. Ich denke, generell ist das so -jetzt auch über dieses Structure Economics Consortium oder so eine europäische Initiative wie IMI, Innovative Medicin Initiative wobei er ja auch sehr aktiv also nicht allein aber mit anderen europäischen Firmen und Universitäten beteiligt ist.	
Interview 31		Das ist dann etwas, was dann einfach auch diesen Kulturwandel zeigt. Und, absolut, das hat dann auch etwas mit der Kultur des Unternehmens zu tun, die übrigens, und das halte ich für ganz wichtig, auch natürlich immer von der jeweiligen Branche abhängt.	
Interview 31		Es gibt eben Branchen, wo man, ich sage mal mehr alleine arbeitet, ich sag es mal so, da wird man wahrscheinlich weniger seine Datenteilungskultur haben. Es gibt andere Branchen, wo man eben mehr zusammenarbeitet, da wird man mehr Teilungskultur haben - ist dann so das Branchengrundmotiv.	
Interview 31		Auf der anderen Seite hat man aber eben auch eine Situation, dass dann jeweils ein Unternehmen sicherlich einen etwas anderen Stil hat und der spielt dann natürlich auch eine Rolle. Aber ich glaube, das ist etwas, was sehr stark im Fluss ist und ich bin auch davon überzeugt, dass sich das jetzt durch diese ganze aktuelle Deep Learning, Artificial Intelligence und so weiter Diskussion nochmal zu mehr Transparenz entwickeln wird.	
Interview 31		Auf der anderen Seite muss man auch sagen, sieht man ja auch durch diese Diskussion wieder den Wert von Daten und das führt dann eben auch dazu, dass man wahrscheinlich noch reflektierter an die Frage herangeht, welche Daten kann ich jetzt sharen, welche nicht.	
Interview 31		Übrigens der Effekte kann man jetzt ganz genau bei diesen ganzen Smartphones und oder so sieht, da hat man jetzt auch die ganze Diskussion im Kontext von Android, Google, Facebook und so weiter, wo man ja auch fragt, welche Daten dürfen jetzt abgegriffen werden, welche nicht, welche dürfen geshared werden.	
Interview 31	31,45	Also ich glaube, man teilt mehr Daten, hat hat mehr Daten, aber man geht auch zunehmend bewusster mit diesem Thema um, ist mein Gefühl.	
Interview 31	33,43		Welchen Einfluss haben Sie denn, z.B. wenn Sie jetzt im Management sind, auf das Datenteilen?
Interview 31		Ja, ich glaube schon. Ich sage mal so als Manager, wenn man im Management ist, hat man natürlich per se natürlich auch, also ich meine natürlich Einfluss auf Entscheidungen.	
Interview 31	33,51	Und ich glaube schon auch, dass da natürlich dann auch wieder die persönliche Haltung zu diesem Thema natürlich auch eine Rolle spielt.	

Interview 31		Und, ja, ich glaube, dass diejenigen, die schon mehr in Kollaborationen gearbeitet haben wahrscheinlich auch tendenziell eher - also das ist jetzt statistisch gesagt, Einzelfälle können immer unterschiedlich sein und Ausnahmen bestätigen die Regel - aber ich glaube, dass diejenigen dann tendenziell vielleicht auch eher bereit sind, den Wert darin zu sehen.	
Interview 31		Also ist ja immer die Frage, sehe ich dann mh ist das Glas halb voll, ist es halb leer. Sehe ich eher das Risiko, wenn ich jetzt etwas teile, dass irgendetwas schief geht und ich sozusagen vielleicht ein potenzielles zukünftiges Patent verliere oder, oder, oder so.	
Interview 31	34,17	Oder sehe ich eher die Chance, dass daraus jetzt eine neue Opportunities wächst.	
Interview 31		Und das (ist) hat natürlich auch sicherlich viel mit der persönlichen, ich sage mal, Grundhaltung zu solchen Themen zu tun. Also Ich glaube, das Management spielt da auch eine Rolle. Aber natürlich auch ganz stark die Unternehmenskultur.	
Interview 31		Ja, also und da spielt auch das Management eine Rolle. Also zum Beispiel bei (Firma) hatten wir natürlich, ich sage mal so, vor einigen Jahren, wahrscheinlich sind das jetzt weniger als man denkt, da war es noch undenkbar, da wurde man eher davor gewarnt, an irgendwelchem sozial Media teilzunehmen.	
Interview 31		So nach dem Motto, wenn man da irgendwas falsches schreibt, dann hat man hinterher wahrscheinlich viel Ärger. Und inzwischen ist es eher so, dass dann auch Leute vom Management, natürlich wirklich ein bisschen auch den Trend folgend, da jetzt anfangen bei Linkedin oder soetwas sich sehr aktiv zu bewegen.	
Interview 31		Und das hat dann natürlich auch einen Einfluss auf die anderen Mitarbeiter. So das wäre jetzt - zum Beispiel bei (Firma) haben wir inzwischen viele, die jetzt auch bei Twitter recht aktiv sind, wenn sie auf irgendwelchen Konferenzen und so sind, oder bei LinkedIn - ich mache das zum Beispiel auch ein bisschen.	
Interview 31	35,00	mmh und das ist dann natürlich auch so ein role model in so einem Unternehmenm klar.	
Interview 31	36,04		Nun haben wir ja schon viele Probleme und Barrieren besprochen. Interessanterweise haben Sie jetzt gar nicht erwähnt, dass der Zeitaufwand oder der Personalaufwand eine Barriere zum Datenteilen sein könnte. Welche Rolle spielt das bei Ihnen?
Interview 31		mhh. Das stimmt. Das ist mmh ja. Das ist eine natürliche Also ich glaube, den haben wir versteckt angesprochen.	
Interview 31		Natürlich ist es so: am Schluss ist es auch immer eine Frage von Aufwand. Und das wird sicherlich auch etwas sein, was in unterschiedlichen Branchen und bei unterschiedlichen Unternehmensgrößen auch unterschiedlich stark eine Rolle spielen wird.	
Interview 31	36,24	Ich denke, dass versteckte sich so ein bisschen in diesem Thema Interface. Ich glaube, (Teilen,) Teilen von Daten. Also ein großer Aufwand ist auch dieses ganze Interface-Thema.	

Interview 31		Ein weiteres Thema ist natürlich auch genau diese Wertung der Risiken. Das heißt, wenn ich Daten geteilt habe, habe ich sie geteilt. Dann gibt's in Anführungsstrichen kein zurück mehr. Und das muss ich mir natürlich gut überlegen, wie wir ja schon gesagt haben, was ich teile, was nicht.	
Interview 31		Und dieser Bewertungsaufwand, oder Diskussionsaufwand, der dann auch dahintersteht - was kann ich teilen, was nicht - der ist, glaube ich, auch größer als der rein technische Aufwand, es dann zu tun, ja.	
Interview 31		Und ich glaube, den halte ich, also sozusagen, diesen Diskussionsaufwand, was sind die technischen, was sind die IP, also Intellectual propertie, was sind die legal risks, hinter dem Teilen; diese (diese) nicht einfache Diskussion, das halte ich für eine der Hauptaufwände hinter dieser Daten, hinter dem Datenteilen.	
Interview 31	37,00	Und klar, der technische Punkt kommt auch noch dazu. Aber ich glaube, das ist gar nicht oft der (der) entscheidende, ehrlich gesagt. Ein großes Unternehmen, das mag für	
Interview 31	38,00	ein kleineres Unternehemn dann anders sein, ja.	
Interview 31			Und die finanziellen Aspekte? Müssen Sie diese berücksichtigen?
Interview 31		Klar, ich meine, im Schluss ist alles eine Frage von Arbeitszeit, auch, natürlich und (und), ja, das spielt natürlich auch eine Rolle.	
Interview 31		Aber ich glaube, wie gesagt, ich glaube, in so großen Unternehmen ist erstmal diese rechtlich mh, Patenthürde ist aus meiner Sicht oft erstmal die größere.	
Interview 31			Ok, gut. Das hat mir auf jeden Fall schon mal sehr viele Fragen beantwortet, gerade. Eine ganz andere Frage ist Folgende: Wie viele Personen kennen Sie, die Daten ihrer aktiv teilen - also über eine Forschungskooperation hinaus?
Interview 31		Über die Forschungskooperation hinaus? - das müssen Sie nochmal spezifizieren.	
Interview 31	39,00		Im Sinne halt von freiwillig in einer Datenbank hochladen. Wissen Sie was ich meine? Also zum Beispiel gibt es ja die (platform). Da werden ja auch immer wieder neue Moleküle aktiv hochgeladen.
Interview 31		Richtig, ja, ja, ja, genau. Also ich hatte jetzt auch an sowas gedacht, dass man auch irgendwas supporting information hochläft.	
Interview 31		Das ist jetzt echt schwer abzuschätzen, aber ich würde mal sagen, dass sind wahrscheinlich irgendwas zwischen 50 und 100.	
Interview 31			Ja, das ist sehr interessant, welche Antwort man bei der Frage bekommt. Also ich habe auch schon mal null gehört.
Interview 31		Die so Twitter oder so etwas aktiv machen? dann hätte ich jetzt wahrscheinlich. Ja gut, kennen und kennen.	
Interview 31		Dann hätte ich jetzt wahrscheinlich gesagt, naja, also 20, wenn ich wahrscheinlich länger überlegt hätte, wäre ich wahrscheinlich auf 50 gekommen, und jetzt habe ich mal gesagt, naja 50 bis 100, das liegt dann, also ich sage mal so	

Interview 31	40,00	sehr viele von unseren Wissenschaftlern machen das, natürlich mit sehr unterschiedlicher intensitöät auch, muss man sagen, das muss man auch ganz klar sagen, also da, ja.	
Interview 31			Inwiweit möchten sie denn diese Data Sharing Aktivitäten in Zukunft ausbauen?
Interview 31		Ja, ich glaube, ich glaube, wir müssen einfach noch mehr zu Modellen kommen, wo jetzt nicht einfach, ich sage mal, besinnungslos Daten irgendwo hochladen, sondern dass wir uns vor allem um die Frage Gedanken machen: wie können wir, ich sage mal, im gemeinsamen Interesse Teilen, ohne dass eben, diese, diese jetzt ja schon mehrfach besprochenenen rechtlichen und patentrechtlichen Risiken zu stark sind.	
Interview 31		Ich glaube, ich glaube, wenn man das schafft, und da sind ja auch genauso Institute wie dieses Structural Economics Consortium zum Beispiel mit dem Probes auch angetreten, um solche Dinge zu tun.	
Interview 31		Ich glaube, dann wird man auch	
Interview 31		automatisch mehr Data Sharing haben. Weil letztenendes hat ja jeder auch ein Interesse selber, auch vielleicht Zugriff auf mehr Daten zu bekommen.	
Interview 31		Also da gibt es natürlich noch ganz andere Bereiche, über die ich jetzt nicht sprechen will, weil ich mich da auch nicht gut auskenne, was für - irgendwelche Daten in der vorklinischen Forschung, also Patientendaten, genetische Daten, all solche Sachen, wo es einfach auch ein großes wissenschaftliches Interesse gibt.	
Interview 31		Es gibt ja auch immer mehr dieses Thema jetzt, wo wir jetzt gerade von den Smartphones gesprochen haben, irgendwelche Daten aus irgendwelchen Apps, in diesem medizisch pharmazeutisch Kontext - das ist natürlich auch noch ein spannendes gebiet, aber das ist jetzt nicht so grunsätzlich mit der Chemie verknüpft.	
Interview 31	41,00	Ich glaube, dass da einfach noch mehr Daten haben - ja, wie wir gesagt haben auch, negativ Ergebnisse, vielleicht auch Daten, die zeigen, wie Publikationen, ob das jetzt funktioniert hat oder nicht, soetwas halte ich auch für wichtig, ja. Dass man da auch noch mehr Feedback einfach kriegt. Dass nicht einfach Daten eben ins Netz gestellt	
Interview 31	42,00	sind oder Publikationen und dann ist es so, sondern dass auch das Feedback für diese Dinge, die Erfahrung der Leute für diese Dinge, mehr ins Netz gestellt wird. Ich glaube, das, das würde auch helfen, dass man einfach noch mehr die Learning daraus irgendwo vergemeinschaftet, sag ich mal.	
Interview 31			Haben Sie sonst noch ..
Interview 31		Wenn ich klar genug war?	
Interview 31			Ja, das war sehr gut, danke
Interview 31			Haben Sie sonst noch weitere Aspekte zum Thema Data Sharing, auf die ich bis jetzt noch nicht eingegangen bin, die sie mir noch sagen möchten?
Interview 31			Was Ihnen so spontan noch einfällt?

Interview 31		Ja, also ich sage mal so, ich glaube, ein Thema für mich ist auch, dass diese ganze, ich sage mal, Patentlandscape, also diese ganze Daten in Patenten, das ist aus meiner Sicht, immer noch zu Textlastig, sage ich mal, ja, und zu wenig wirklich, im Sinne von Daten, mit denen man arbeiten kann,	
Interview 31		ja. Also ich finde es ehrlich gesagt noch so ein bisschen	
Interview 31		anachronostisch zum Teil, ehrlich gesagt. Ja, dass man jetzt diese Patente, statt immer noch in dieser, in der Form, wie wahrscheinlich 50 oder vielleicht auch vor 100 Jahren, ja, dass das dann immer als Text da noch vorliegt.	
Interview 31		Ich glaube, dass gerade auch im Grenzbereich zu legal, ja also, dass wir immer noch zu wenig aus meiner Sicht, zu wenig digital sind. Und dass das eben, aus meiner Sicht, auch eine Hürde ist für das sharen von Daten.	
Interview 31	43,00	Das wäre noch so ein Aspekt. Sie haben gemerkt, dass ich auf diese Grenzfläche, mit der sie vielleicht nicht so gerechnet habe, sehr stark abgehoben habe, weil die eben aus Industriesicht eine wichtig ist.	
Interview 31			Ja, außer Frage. Also auch mit Patenten haben wir uns ja schon im Studium mit auseinander gesetzt, das ist da auch schon nicht einfach. Dann komme ich jetzt zu meiner alleletzten Frage. Wir haben uns ja nun zum Thema AI kennen gelernt. Meine Frage an Sie ist: mit zunehmendem technologischen Fortschritt
Interview 31	44,00		werden ja immer mehr Datenmengen benötigt. Wie schätzen Sie jetzt den Einfluss von Big Data oder künstlicher Intelligenz auf Ihren Forschungsalltag ein?
Interview 31		Mhh. Ja, also das ist natürlich eine gute Frage, nicht. Also wie sagt man jetzt? Wie war das? Prognosen sind schwierig, insbesondere, wenn sie sich auf die Zukunft beziehen. Mh, ja. Also ich glaube, ich mein, ich glaube, der Einfluss wird, auf der einen Seite, kann sehr groß werden.	
Interview 31		Ich meine das auch im positiven Sinne, das ist mir auch wichtig. Aber er muss nicht unbedingt den grundsätzlichen Charakter des Bereichs ändern. Was meine ich damit?	
Interview 31		Also ich meine, ich glaube, dass wir viele Tätigkeiten haben, das hatte ich ja auch im Vortrag versucht so ein bisschen zu erläutern, viele Tätigkeiten haben, die in unserem Bereich - sei es im Labor, da vor allen Dingen übrigens, und sei es auch bei der Planung von, sagen wir mal, Design neuer Molekule und so weiter und sofort - die noch zum Teil recht mechanisch oder, oder, in, in, in einzelnen Häppchen berarbeitet werden.	
Interview 31		Und das liegt, aus meiner Sicht, gar nicht so sehr daran, dass man es nicht besser machen könnte, sondern da kommt jetzt wirklich auch dieser Aufwandspunkt rein, den sie genannt haben, sondern weil wir einfach noch sehr viele unterschiedliche monolithische Systeme haben, die wie so, oder endemisch, müsste man schon fast sagen, die eben nicht miteinander kommunizieren.	

Interview 31		Und deswegen kann da sozusagen, der volle, volle Effizienzgewinn, oder, oder der volle, sagen wir mal, die Erkenntnis kann da nicht von einem System ins andere fliessen und andersrum. Und ich glaube, das ist ein Riesenproblem, dieses Interface Problem.
Interview 31		Also ich glaube, wenn die Artificial Intelligenz es schaffen würde, auch bei diesem Interface Problem zu helfen, da wird ja auch nicht oft so drüber geredet, ich glaube, dann würden wir massive Änderungen haben.
Interview 31		Ein Bereich, wo wir das jetzt schon am Anfang sehen - und das wird auch mit Sicherheit kommen, ich mein, das hat ja auch der Herr Sattler da gesagt - ist zum Beispiel bei der Syntheseplanung.
Interview 31		Das finde ich ein absoluter schon fast Nobrainer, ehrlich gesagt. Dass man jetzt eben, ich sage mal, Klammer auf zum Glück Klammer zu, dass man in Zukunft nicht mehr nur dann jetzt tausend Einzelreaktionen sich anguckt und dann hinterher eine Synthese zusammen bastelt, sondern dass man natürlich jetzt ein Computersystem hat, das schon in, im ,im Gänze macht, dass man nicht jetzt eine Einzelreaktion macht, guckt, sondern gleich eben eine ganze Syntheseroute vorschlägt.
Interview 31		Das ist ja letztenendes qualitativ gar nicht so eine Änderung, aber quantitativ eben massiver Schritt, Schritt nach vorne. Und das wird dann zum Beispiel Einfluss haben darauf, wie planen in Zukunft Chemiker ihre Synthesen - weil die werden sich dann einfach weniger in Einzelliteratur gucken, sondern die werden sich da halt von so einer Computersoftware dann eben Sachen selektiv angucken.
Interview 31		Sie werden trotzdem noch sich Gedanken machen über Synthesen oder die bewerten, da bin ich mir sicher, aber das wird natürlich jetzt effizienter. Im Labor könnten wir auch viele solche Dinge machen.
Interview 31		Da sehe ich sogar ehrlich gesagt, dass wir da irgendwann Richtung Roboter, Robotiks mehr gehen. Das dauert vielleicht noch ein bisschen länger, bin ich mir aber ziemlich sicher, dass das kommen wird.
Interview 31		Das wird natürlich dann den Alltag der technischen Tätigkeiten im Labor stark beeinflussen - das sehe ich, ja.
Interview 31	45,29	Aber ich glaube trotzdem, die Unsicherheit im Umgang mit biologischen System, ja, die, das sehe ich nicht, dass das sobald weggehen wird. also, ich meine, ich sage mal, wie der andere Kollege das auch gesagt hat, dass man jetzt alle toxikologischen
Interview 31		Ergebnis - oder nicht alle - toxikologischen Ergebnisse vorhersagen kann - da bin ich jetzt ehrlich gesagt etwas skeptischer, auch aufgrund meiner bisherigen Berufserfahrung; das glaube ich, das wird noch viel, viel länger dauern, weil ich glaube, die Komplexität dahinter steht, die ist auch mit solchen, ich sage mal, Deep Learning Ansätze nicht so leicht zu, zu beherrschen.
Interview 31		Also in sofern wird man immer noch sehr, glaube ich in unserer Branche, immer noch eher empirisch unterwegs sein, aber dabei kann natürlich diese mh können diese Deep learning und Artificial Intelligenz Ansätze die Sache trotzdem effizienter machen, aus meiner Sicht.

Interview 31			Ja, vielen, vielen Dank für diesen tollen Einblick. Ich bin da auch vollkommen Ihrer Meinung, dass wir auf jeden Fall großes Potential haben können mit der künstlichen Intelligenz. Zum Abschluss meines Interviews möchte ich noch ein paar Statistiken erheben, allgemein.
Interview 32	Time	Interviewee 32	Interviewer
Interview 32	0		"Aufzeichnung wird gestartet" angezeigt. Ok. Bei mir steht das zumindestens. Alles klar. Dann wollte ich da nochmal im vorhinein etwas zu der Datenschutzvereinabrung sagen. Also das, was wir jetzt alles sagen (...) wird auch anonymisiert und es ist nicht auf Sie zurück zu verfolgen.
Interview 32		Ok, auch das ist auf jeden Fall gut zu wissen.	
Interview 32		Wie lange dauert das jetzt ungefähr - dass ich hier eine grobe Vorstellung habe?	
Interview 32			Also ich schätze so eine dreiviertel Stunde. Also es sind 15 Fragen, 15 offene Fragen. Und jenachdem was da so kommt, können das auch mal ein, zwei Fragen mehr sein - wie das Gespräch so läuft.
Interview 32		Ja, ja,ja klar, Selbstverständlich. Jenachdem wie viel mir auch einfällt dazu zu sagen. Und was sie so wissen möchten, weiß ich gerade auch noch überhaupt noch gar nicht, muss ich sagen.	
Interview 32			Ist auch alles gut, es braucht auch keine Vorbereitung. (...)
Interview 32		Ah, Daten Sharing. Ich muss zugeben, ich habe Ihren Flyer leider verloren. Ich habe gerade nochmal gesucht, aber genau. Insofern, wenn sie noch zwei, drei mehr Worte zu ihrem Thema sagen würden, wäre das vielleicht hilfreich.	
Interview 32	1,3		Ja ok, also: Meine ganze Arbeit handelt davon
Interview 32		Ah, ok. Das klingt auf jeden Fall für mich auch wirklich mega spannend, genau. Weil, ja, genau das möchten wir durchaus in unserem Sonderforschungsbereich natürlich auch erreichen, dass die Leute möglichst viel Daten tauschen untereinander.	
Interview 32	2,21	Also momentan bin ich in der Wissenschaftskordination tätig. Das bedeutet, ich begleite dieses Verbundforschungsprojekt, Sonderforschungsbereich, eines Sonderforschungsbereichs in der Chemie, was alle Managementaufgaben angeht.	Ja, genau. Und dann möchte ich gleich mal anfangen mit der ersten Frage. Die ist: was gehört so zu ihren täglichen Aufgaben jetzt in dem Sonderforschungsbereich?
Interview 32		Das heißt: anfangen tut es mit den Finanzen. Das heißt, ich kenne die Budgets, die da sind. Ich habe einen Überblick über das Personal - wer ist bei uns derzeit angestellt, wer ist nicht angestellt, wo sind Budget übrig, die ungenutzt verfallen würden. Genau. Forschungsdaten, wo werden die abgelegt, wie werden die ausgetauscht, wie oft treffen die Menschen sich um ihre Forschungsdaten zu teilen, welche Veranstaltungen wären hilfreich, damit die Leute sich öfter treffen, wer ist Doktorandensprecher und organisiert regelmäßige Treffen auf Doktorandeebene.	

Interview 32	Welche Entscheidungen müssen im Vorstand getroffen werden hinsichtlich der nächsten Förderperiode. Wie muss, mit wem müssen die sich möglichst treffen, welche Vortragsgäste wären hilfreich, damit unsere wissenschaftliche Thematik für die nächsten vier Jahre aufgestellt wird.	
Interview 32	Was dürfen wir von der deutschen Forschungsgemeinschaft aus tun mit unserem Geld, aber auch was wünscht sich die Deutsche Forschungsgemeinschaft von uns - und das natürlich insbesondere im Hinblick auf Forschungsdaten.	
Interview 32	Genau, da bin ich immer im so Spannungsfeld unterwegs. Einerseits, Forschungsdaten, was möchte der Fördergeldgeber, die DFG, von uns was Forschungsdaten angeht. Andererseits, was ist praktisch wirklich hilfreich. Also es ist schon ein Spannungsfeld, was sieht gut aus und was ist in der Praxis auch umsetzbar und nutzbar beziehungsweise auch vom Arbeitsaufwand her vertretbar, was man eben an neuen Systemen einführen kann beziehungsweise was ist von unseren Ressourcen her machbar.	
Interview 32	Und das sind eben alles drei Bereiche, die ich durch meine derzeitige Tätigkeit abkläre und wo ich mich eben sehr bemühe im stetigen Gespräch, sowohl mit den Professoren zu sein als auch mit der DFG, als auch mit unseren Doktoranden.	
Interview 32	Und da würde ich behaupten bin ich nicht so schlecht für aufgestellt durch meien Hintergrund, weil ich eben selber Chemikerin bin, auch promoviert habe, auch einen Postdoc gemacht habe im Anschluss daran, in der medizischines Chemie habe ich Postdoc gemacht.	
Interview 32	Da ging es auch sehr viel darum, gerade interdisziplinär Forschungsdaten auszutauschen, weil man eben mit Biologen, mit Medizinern, mit Leuten, ja, die Mikrobiologie gemacht haben, eben die Daten austauschen musste und selber eben organischer Chemiker war.	
Interview 32	Und mein eigener Hintergrund eben von der Wissenschaft her ist die organische Synthesechemie, die eben auch in unserem Sonderforschungsbereich teilweise abgedeckt wird. Aber teilweise machen wir auch ganz andere Sachen im Sonderforschungsbereich also Physiker, Ingenieure.	
Interview 32		Ja, ich habe mir zur Vorbereitung auch schon den Zeitartikel durchgelesen. Es gab ja auch einen über den Sonderforschungsbereich. Ja, sehr spannend auf jeden Fall, was Sie da machen.
Interview 32	Ja, genau.	
Interview 32		Sie haben ja gesagt, Sie kümmern sich auch darum, wie sich die einzelnen autauschen untereinander mit den Daten. In was für bestimmten Datenaustauschaktivitäten sind Sie denn involviert?
Interview 32	Also grundsätzlich fängt das ganze an, oder sind die Sachen, in die ich am meisten involviert sind, dass ich Veranstaltungen organisiere, auf denen Daten vorgestellt.	

Interview 32	Genau. Also weil ich jetzt nicht weiß, ob Sie das mit Datenaustausch meinen. Da werden natürlich Forschungsergebnisse vorgestellt.	
Interview 32	Und Das ist jetzt weniger so dass ist dann weniger so, dass ein Datenfile weitergereicht wird, sondern man hält Vorträge, man macht Poster und bereichtet dann einander davon, was eben passiert ist. Also das ist der konservative Austausch, wie man ihn schon immer gemacht hat und wie wir ihn eben derzeit auch natürlich weiterhin vorantreiben.	
Interview 32	Darüber hinaus versuche ich, weil das ein sehr, der einzige Weg ist, der bisher etabliert ist um Daten auszutauschen.	
Interview 32	Zum einen - was heißt der einzige Weg? Selbstverständlich gibt es auch noch Artikel publikationen in Journals, über die Daten ausgetauscht werden. Selbstverständlich veröffentlichen unsere Leute ihre Daten in Artikeln, wo sie eben auch kollaborieren und da ist eben natürlich auch eine Supporting Information, sind die Daten enthalten. Genau.	
Interview 32	Das eben zu den Dingen, die sowieso schon exisiteren in der Chemie und die auch uns tendenziell immer noch die Grundlage bilden.	
Interview 32	Darüber hinaus versuchen wir eben jetzt verschiedene andere Tols zu etablieren, um Forschungsdaten auszutauschen. Und das ist eben zuallerst dieses (platform), wo wir eben die IT Infrastruktur aufgebaut haben.	
Interview 32	Wir sind jetzt gerade soweit, dass die IT soweit steht, dass wir sagen, wir trauen uns, den ersten Testnutzer damit arbeiten zu lassen. Und da hoffen wir natürlich eben auch, dass eben ein gewisser Datenaustausch darüber entsteht.	
Interview 32	Was jetzt gerade neu angestoßen wurde von unseren Doktoranden selber ist Slack als Kommunikationstool.	
Interview 32	Kennen Sie das? Das ist natürlich sehr, kein klassisches Forschungsdatenaustauschtool, nichtsdestotrotz, hoffen wir, dass wir da gewisses, einfach Kommunikation aufrecht erhalten können, zwischen Menschen, die nicht am gleichen Ort arbeiten, indem wir einfach verschiedene Chatrooms, eben Chats - Treads nennt sich das glaube ich, diese verschiedenen Themen, die man da aufmachen kann als Chat - die zum Beispiel schon auch mit Forschungsdaten insofern zu tun haben, dass man sagt, wir haben bestimmte Dinge, Ergebnisse, die ausgetauscht werden müssen, in unserem Sonderforschungsbereich.	

Interview 32		Das sind bei uns insbesondere Materialkomponenten, die in einem Projekt hergestellt werden und die dann weiter gegeben werden müssen, möglichst breit, über den den gesamten Sonderforschungsbereich. Und da hoffe ich eben, dass man da zumindest jetzt als Anfang, bevor das elektronische Laborbuch richtig läuft, die Leute dazubringen kann, dass man sagt: wenn man was zur Verfügung stellen kann, was dem anderen Projekten eben in ihrer Forschung, den anderen Projekten ihrer Forschung weiter bringen kann, dass man da reincshreibt: wir haben jetzt, wir waren jetzt in der Lage Material X herzustellen, das hat Prorengröße 5 nm und hier folgende Analytikparamter 1, 2, 3, Falls es jemand haben möchte, sagt Bescheid.	
Interview 32		Ist erstmal nur so ein kollaborationsding und natürlich nichts, was jetzt langfristig irgendwie die Forschung voran bringt, aber halt in unserem Verbundprojekt einfach die grundelgende Informationen auf einer gesicherten Plattform eben auch archiviert.	
Interview 32		Denn momentan läuft es viel so: die Leute unterhalten sich, der eine sagt: ich habe jetzt gestern geschafft das und das zu machen. Dann wissen es zwei Leute, aber in unserem Sonderforschungsleute sind 40 Leute. Da sind noch mindestens 5, die das eigentlich auch am besten wissen sollten und nächste Woche kommen vielleicht drei neue dazu, die das Zeug auch brauchen könnten.	
Interview 32		Aber weil das dann ja schon alt ist, denkt niemand mehr dran denen zu sagen: ach ürbigens, dieses Zeug gibt es auch. Also das ist halt dieser Archivierungsgedanke, ist da irgendwie erstmal ganz stark und soll erstmal ein ganz niedrig level Einstieg bringen, dieses Slack. Genau.	
Interview 32		Ja also wie gesagt, das ist jetzt ganz neu. Die Doktoranden wollten das selber. Also habe ich gesagt: ja klar gerne, macht das.	
Interview 32		Genau und als letzten Schritt ist eben dann dieses Datenrepositorium, das ich auch schon auf dem Wissenschaftsforum erwähnt habe, eben die dritte Aktivität in der ich momentan beteiligt bin.	
Interview 32	10	Wobei momentan die Motivation da bei uns die ist, wenn man Daten sowieso schon in einem Journal Artikel veröffentlicht hat, und da eben dieses normale Wordprotokoll, sage ich mal, also Textfile Protokoll des NMRs oder des IR-Spektrums oder was auch immer, veröffentlicht hat, dass man da eben auch noch die Rohdaten Files und den bearbeiteten Datenfile auch noch dazu abzulegt in Kombination mit der Information "mehr dazu steht in diesem Journal Artikel".	
Interview 32		Und, genau. Weil man momentan befürchtet, dass der Aufwand, da Daten zu hinterlegen, zumindest nach dem momentanen Stand, wie wir unsere Daten protokollieren, sehr groß wäre, ja genau, ist da nun die Motivation da, wenn etwas sowieso in einem Journal Artikel drinnen steht, dann klnnen wir dazu auch den Rohdatenfile dazu hinterlegen in dieser Form.	
Interview 32	10,53	Aber da hört es dann auch auf. Ja, also diese drei Aktivitäten, Slak, (platform) und eben das Date, Forschungsdatenrepositorium, das bei uns eben von der Universität (Ort), beruhend auf (platform)	
Interview 32			Ja, ich wollte dieses Thema an meiner Universität vorantreiben.

Interview 32		Ganz kurz gucke ich mal, dass ich das Rollo zumachen kann - mir scheint die Sonne so ins Gesicht.	
Interview 32	12,06	Ok, so ist besser.	
Interview 32			Ja, sehr schön. Dann fahre ich mal fort mit meiner nächsten Frage: Jetzt haben Sie ja schon erzählt, dass Sie auch untereinander austauschen. Wie ist das denn mit Verwenden von anderen Daten bei Ihrer Forschung. Wird das gemacht? Welche Daten werden denn von den Doktoranden oder den Forschenden verwendet?
Interview 32	12,32	Sie meinen jetzt, Daten innerhalb unseres Sonderforschungsbereiches oder grundsätzluich Forschungsfaten, die einfach schon existieren?	
Interview 32			Genau, die von anderen produziert wurden, die sie verwenden. Jetzt ganz grob gesagt, Spektren zum Abgleich oder so.
Interview 32		Ja, also davon würde ich definitiv ausgehen, dass man, definitiv, Synthesestrategien, sind ja auch Forschungsdaten, dass man die recherchiert und dann auch nutzt.	
Interview 32		Und dazu dann eben auch, wenn man bekannte Moleküle, was in unserem Fall meistens Referenzmoleküle sind und nicht der eigentliche Forschungsinhalt, ja, dass man die eben auch, die Spektren eben nachschaut und eben abgleicht ob eben das mit der Literatur übereinstimmt.	
Interview 32		Einfach weil man dann einfach weniger Daten produzieren muss zu einem Molekül, wenn man eben sagen kann: "hier,das ist Literaturbekannt, das hat schon jemand volstädnig characterisiert und insofern müssen wir im Prinzip nur noch ein NMR machen, auf jeden Fall keine Elementaranalyse mehr - ich weiß nicht, ob Sie das Problem kennen.	
Interview 32	13,47	Das sind definitiv Daten, die wieder genutzt werden. Ansonsten in unserem Theorieprojekten ist es definitiv Software, falls man das als Forschungsdaten bezeichnet, genau, die genutzt wird natürlich. Ansonsten, ja, ich muss sagen bei unseren physikalischen Chemikern habe ich weniger Einblick, was die tun.	
Interview 32			Ok, ja. alles gut, weil manchmal ist es so: Daten teilen ja. Daten teilen heißt aber auch Daten von anderen zu verwenden.
Interview 32		Sie meinen jetzt auch intern?	
Interview 32			Ja
Interview 32		Ich habe jetzt erstmal an die Recherchen gedacht, die man macht und die als Grundlagen für die eigenen Forschung dienen. Daten teilen, im Sinne von: dass man wirlich innerhalb des Projekts, also, man nutzt die Ergebnisse, die der andere hat, aber wirklich dass man jetzt wirklich den Datenfile von jemand anderem nochmal aufmacht, ne, also dass man den Rohdatenfile nimmt oder den bearbeiteten File sich selber anguckt und da irgendwelche Schlüsse rauszueht, das passiert definitiv nicht.	
Interview 32	15		Ok.

Interview 32	Also zumindest nicht in den meisten Fällen, weil es einfach komplexe Messungen, komplexe Simulationen, komplexe Zusammenhänge sind, wo wir der Meinung sind, dass man diese Ergebnisse auf jeden Fall von dem Experten in den Zusammenhang gesetzt bekommen muss und dass diese Ergebnisse auch wirklich nur in diesem Zusammenhang gültig sind, den man dann eben dargestellt bekommt.	
Interview 32	Und in sofern ist da, ist es sehr schwierig, also wirklich wieder nutzbar sind unsere Meinung nach nur Standarddaten. Und Standarddaten sind eben Charakterisierungsdaten vom Molekül zum Beispiel, aber alles, was darüber hinaus geht, da braucht man unserer Meinung nach entweder einen Artikel, den man dazuzgeschrieben hat, indem man das dann ein bisschen in den Zusammenhang setzt, oder b) man braucht den Forscher, mit dem man spricht wirklich.	
Interview 32	Insofern wieder dieses Format, Veranstaltung in dem man seine Ergebnisse präsentiert im Sinne eines Posters oder eines Vortrags, wo man eben dann wirklich im Zusammenhang sagt, welche Bedeutung diese Daten haben.	
Interview 32	Ansonsten nutzen höchstens eben, würde ich jetzt sagen, in dem Sinne, dass man eine Überblickstabelle bekommt. Zu was hat denn Projekt X schon alles was gemacht?	
Interview 32	Haben die schon Ergebnisse ganz grob zu, haben Sie schon ein poröses Material mit 5 nm Porengröße hergestellt? Das wäre eine Abfrage, die ich als Kollaborationspartner interessehalber vielleicht in einer Datenbank nachgucken würde. Und dann würde ich sehen: ja da ist irgendwas. Und dann würde ich dort hingehen und mir das erklären lassen - und dann damit arbeiten? Schwierig.	
Interview 32	Beziehungsweise im Endeffekt soll es natürlich schon so sein, dass die Leute, die physikalische Messungen machen und characterisieren wie ein Material aussieht in dem Menschen eben die Simulationen machen vorherzusagen wir ein Material aussieht - oder wo das Energieminimum eines bestimmten Materials oder Hybrids zwischen Katalysator und Material aussieht, dass die im Endeffekt übereinkommen, was eben die logischte Interpretation sowohl ihrer Messung als auch ihrer Simulation ist.	
Interview 32	Aber auch das ist auch das, es funktioniert eben nicht so, dass man die Simulationen in der Datenbank hinterlegt und dann der Spektroskopier sich einsam und allein in sein Kämmerlein hinsetzt und sich die Simulationsdaten angucjkt und schaut ob das mit dieser Spektroskopiedaten übereinstimmt und umgekehrt, sondern auch das ist, weil eben die Spektroskopiedaten und die Simulationsdaten eins zu eins vergleichbar sind, ist ein komplexer Prozess.	
Interview 32	Insofern ja, ist da irgendwo eine Nutzung da gegenseitig der Daten weil man eben versucht ein schlüssiges Bild aufzubauen aus verschiedenene Richtungen - Theorie und Spektroskopie - aber dieses wirklich: ich als selbstständiger Forscher in meinem Gebiet nutze die Daten von jemand anderem aus einem anderen Gebiet ist eben sehr schwierig, genau.	

Interview 32		Und da ist das beste Beispiel, wo man das wirklich gut machen kann, wirklich Standardanalytik von Molekülen.	
Interview 32			Da muss man das Rad ja nicht immer neu erfinden.
Interview 32		Genau, da ist es wirklich hilfreich.	
Interview 32			Jetzt haben Sie ja schon erwähnt, dass Sie bei Slack reinschreiben und sich austauschen, was sie so hergestellt haben. Wie stellen Sie sich in Zukunft den Zugriff auf benötigte Daten vor?
Interview 32	18:47	Ok, wie? Im technischnen Sinne? Oder wie?	
Interview 32			Also das es zum Beispiel eine große Datenbank gibt? Haben Sie sich dazu schon Gedanken gemacht?
Interview 32		Ja, also ich meine im Idealfall gibt es im Endeffekt einen Ort, das wäre schonmal hilfreich wie Sie schon festgestellt haben.	
Interview 32		Es ist gerade in der Testphase, wo man nicht so richtig weiß, was am besten funktioniert. Eher nicht, dass was wir jetzt als erstes anstreben, aber ja, im Endeffekt soll es ein Ort sein, wo jeder weiß, dass die Daten hinterlegt sind.	
Interview 32		Dieser Ort soll durchsuchbar sein - digital nach verschiedensten Parametern und zwar in der Chemie mindestens nach einer Struktur, aber selbstverständlich auch nach individuellen Parametern.	
Interview 32		Also wir können keine Datenbank gebrauchen, die total standadisiert ist, sondern müssen unbedingt auch selber einstellen können, welches die suchbaren Parametern für uns sein sollen, weil doie eben für uns anders sein können als f+ür andere.	
Interview 32		Das wäre auf jeden Fall wichtig. Und sollen eben möglichst niedriges Einstiegslevel bereitstellen, also sodass auch jemand, der sich nicht lange eingearbeitet hat, mit der Datenbank schon was anfangen kann.	
Interview 32		Aber trotzdem auch komplexere Daten hinterlegt haben, aber eben auf eine Art und Weise, dass die eben erst im zweiten Schritt sozusagen auffallen, also das heißt man braucht eine extreme gute Metadaten, aber eben auch nicht so viele, dass man den Überblick verliert.	
Interview 32		Also ich muss sagen dieses Metadatenthema finde ich ein extreme schwieriges, weil je mehr man verlangt von den Leuten, dass sie ihre Metadaten eintragen, desto besser auffindbar sind sie theoretisch, aber desto kleiner ist die Chance, dass es a gepflegt wird und b wenn du den Leuten immer alle Metadaten anzeigst zu den Datenfile, die theoretisch exoisieren, guckt da jeder nur rauf und sagt hä.	
Interview 32	20	Und hat dann auch wieder nichts davon. Also dass man einfach eine sinnvolle zusammenstellung von Metadaten für das derzeitige Projekt sich eben ausdenkt, wo man eben auf niedrigem Könnenslevel schnell den Überblick erlangt, was gibt, was gibt es nicht?	
Interview 32		Allerdings eben nicht unbedingt, das Verständnis vollkommen den Leuten gibt, also erstmal was existiert an Forschungsdaten, das ist eben wichtig. Und alles andere kann dann in der persönlichen Kommunikation folgen.	

Interview 32			Da kann ich ja gut anschliessen an meine nächste Frage: Inwieweit werden oder möchte Sie die Data Sharing Aktivitäten ausbauen?
Interview 32		Sie meinen so eine Zukunftsvision?	
Interview 32			Ja genau.
Interview 32		Also ich muss sagen, ich bin der Meinung, die Vision, dass wirklich jeder unserer Forscher im Alltag ein (platform) nutzt, ist schon eine extrem weitreichende.	
Interview 32		Die momentan fast unrealistisch erscheinend, einfach wegen der großen Diversität der verschiedenen Arten von Forschung, die die Leute betreiben.	
Interview 32	22,26	Das heißt, die Vision wäre, ein elektrosnisches Laborbuch haben, das möglichst Modular aufgebaut ist, sodass man jedem verscheidenen Forschern aus unterschiedlichen Disziplinen die Funktionalität bieten kann, die sie brauchen um sinnvoll und mit Zeitersparnis eben diese digitale Form der Protokollierung nutzen zu können.	
Interview 32		Da dann eine Schnittstelle zu haben zu Repositorien durch die leicht sämtliche Metadaten, die man im Alltag protokolliert hat, in strukturierter Form irgendwo hochgeladen werden, sodass sie durch andere Menschen aufwindbar sind, genau.	
Interview 32		Aber insbesondere das modulare, das ein digitales Laborbuch, dass dann eben auch als Teilungsplattform dienen kann für interdisziplinäre Forschungsprojekte ausreicht, halte ich für so eine sehr weitreichende Version, die momentan weit davon entfernt ist, das sie umgesetzt werden kann. Aber ja, das wäre die Vision für die Zukunft, genau.	
Interview 32		Und vor allem wichtig eben dabei, dass jeder einzelne dieser Forscher eben das Ganze als Vorteil empfindet. Das heißt a) Zeitersparnis im Alltag davon hat und b) Zeitersparnis am Ende seiner Forschungsarbeit, wenn er seine Ergebnisse zusammentragen möchte oder wenn ihn schlicht der Chef fragt, was haben Sie den letzten Monat gemacht?	
Interview 32	23,17	Das heißt irgendwo auf den Knopf drücken kann und sagen kann jüp und man hat zumindest schonmal alle Experimente überblicksmäßig zusammen gestellt und muss nur noch drei Sätze dazu schreiben warum man den einen oder den andere Versuch gemacht hat	
Interview 32			Jetzt haben Sie ja schon gesagt, Zeitersparnis ist auf jeden Fall ein Grund. Ich frage mich jetzt, welche Probleme oder Barrieren sehen beim Thema Datenaustausch mit anderen jetzt konkret? Warum ist das noch nicht etabliert oder was hindert einen daran Daten miteinander auszutauschen
Interview 32		Also ich meine zum einen defintiv die Tatsache, dass viele Daten nur analog vorliegen oder viele, ja, Überlegungen, die man anstellt und das deswegen Zeitaufwand bedeuten würden sie so aufzubereiten, dass sie auch jemand anders verstehen könnte, ideal auch analog sein Laborbuch so zu führen, dass jemand anders es versteht.	

Interview 32		Selbstverständlich schon immer existenz aber nicht unbedingt realistisch, ja, wenn die Leute Aufschriebe selbst verstehen, ist es sicherlich in vielen Fällen schon viel.	
Interview 32		Auch noch drei Monate oder ein Jahr später, genau. Also das hindert auf jeden Fall an Daten teilen oder Experimente teilen, weil wenn man sagt: ach cool, du machst sowas? Sowas habe ich vor einem Jahr auch schon gemacht - ich gucke mal.	
Interview 32		Und dann in sein Laborbuch guckt und feststellt "mmh ist vllt nicht so übersichtlich, wie ich es aufgeschrieben habe - ob er da was damit anfangen kann?" Dann gebe ich es tendenziell nicht weiter, aber wenn ich es hübsch in meinem digitalen Laborbuch habe und nur Datum März 2018 gucken muss, "achja stimmtn, da ist der Versuch, ich teile das mit dir" dann ist das natürlich sehr viel einfacher.	
Interview 32	25	Also das ist auf jeden Fall was, was die Leute am Teilen hindert weswegen Digitalisierung der Daten extrem wünschenswert wäre.	
Interview 32		Dazu kommt aber im Grunde, aber ist auch absolut nur auf dem internen Level gedacht, dazu kommt natürlich aber schon auch, dass man Angst davor hat, dass Leute was klauen von einem, dass man halt gar nicht so vielen Leuten seinen Zugang dazu verschaffen möchte, was ureigenste Gedanken sind.	
Interview 32	26,17	Denn Forschung ist auch viel Kreativität und viel harte Arbeit, und wenn man selber 100 Varianten von einer Reaktion durchgekocht hat, um endlich zu finden, wie es funktioniert, möchte ich sehr gerne möglichst lange für mich behalten, wie das jetzt funktioniert - nicht, dass das jemand anders plötzlich für seins verkauft.	
Interview 32		Und auch dass ich jetzt auch im größeren Maßstab gedacht, dass ich auch denken könnte, gerade wenn ich meine Rohdatenfiles teile, eben auch selbst in Repositorien mit - wie nennt sich das? diese ID? Persistent Identifier? Sie wissen was ich meine?	
Interview 32			DOI
Interview 32		DOI . Genau - ist es so, wenn jemand diese Rohdaten hat, dann kann er dann vielleicht auf Grundlage meiner Forschungsergebnisse was fälschen, weil er ja auch wirklich Zugriff auf die Rohdaten hat und die auch seinem Dokument hinzufügen kann, und dadurch sehr viel einfacher behaupten kann, er hätte eine ähnliche Messung gemacht wie ich und, ja.	
Interview 32		Das ist sicherlich auch was die Leute davon abhalten könnte zu sagen, sie wollen auch überhaupt ihre - selbst wenn sie ihre Journalartikel veröffentlicht haben - auch die Rohdaten files dazu nicht hergeben, weil sie sagen: "Das sind meine Daten und ja, es war viel Arbeit, die zu messen, und merkt ja keiner, wenn die jetzt jemand anderem, anders seinen Titel zu ähh, da dazu packt und behauptet, es wären seine.	

Interview 32			Ja, ich habe jetzt gerade daran gedacht: sie haben ja jetzt schon so technische Aspekte genannt, dass das ja auch eine Barriere sein könnte, dann hatten sie ja auch noch gesagt, dass es zeitaufwendig und es benötigt viele Ressourcen - und Personalaufwand? Ist das auch ein wesentlicher Faktor, dass Datasharing Probleme bereiten kann?
Interview 32		JA, also, ich meine selbstverständlich die IT-Systeme müssen bestehen und derzeit, ich meine es drängen immer mehr Anbieter auf diesen Markt, die das ganze kommerziell anbieten, die dann zwar einstiegspalete für akademische Einrichtungen anbieten, die kostenlos sind, aber die werden dann, wenn man sie für die gesamte Institution nutzen möchte, selbstverständlich auch nicht mehr kostenlos sein.	
Interview 32		Also da ist ein langfristiger Finanzierung selbstverständlich ein Problem, weil man keine langfristige Finanzierung dafür hat und kein System einsetzen möchte, was man sich im moment, wenn man es mit fünf Nutzern nutzt, leisten kann, aber wenn alle es nutzen würden nicht mehr, genau.	
Interview 32		Das ist die kommerzielle Variante, genau, das ist ein Argument warum die kommerzielle Variante fast nicht gangbar ist.	
Interview 32	28,4	Das wäre dann natürlich professionell gemacht und vielleicht auch stabiler. Stattdessen macht man dann die Open Access Lösung, die natürlich auch besser sind, weil man eben da selber eben in mehr individualisiren kann, was ich ja auch schon erwähnt hatte, ein wichtiger Punkt ist, aber dafür hat man eben auch nicht die IT Unterstützung im Sinne von: IT Administratoren an der Uni, die langfristig finanziert sind, die sich um die Systeme dann auch widerum	
Interview 32		Also insofern ist da der große Knackpunkt die Grundausstattung der Universitäten in Deutschland in der Breite in der IT ist nicht gegeben.	
Interview 32		Und die Fördergeldgeber, die eben solche IT Projekte momentan fördern, wie die DFG oder auch das Land (Ort) ist bei uns sehr stark, sind selbstverständlich dahinter auf Projektbasis, da inovative Ideen zu fördern.	
Interview 32		Aber diese innovativen Ideen zu verstätigen und da langfristig den Systemen eine Chance zu geben, dafür ist schlichtweg einfach die IT Infrastruktur nicht da beziehungsweise nur zu sehr geringem Maße da.	
Interview 32		Das heißt, man hat oft keinerlei Freiheit, verschiedene Systeme auszuprobieren, einfach weil genau für ein Projekt da ist, das ist an der Universität Stuttgart momentan dieses Forschungsdatenrepositorium Davus, das ist sicherlich nicht das schlechteste, aber ob es das beste ist, weiß man auch nicht, genau.	
Interview 32	30	Und das müssen wir jetzt alle nutzen, weil da wird irgendwie was reingesteckt und das ist jetzt aber auch das einzige Projekt, wo eben langfristig Geld für da ist. Die fehlende Grundausstattung in der Breite an den Universitäten in Deutschland, was IT Infrastruktur angeht, ist ein großes Problem, was das angeht	

Interview 32			Sind sie denn dadurch, dass das ein Forschungsbereich ist auch rechtlich gebunden an Datenaustauschaktivitäten?
Interview 32	31,33	Wie meinen Sie das? Ob wir dazu verpflichtet Daten zu teilen?	
Interview 32			Ja, bei einigen Journals ist es auch so, dass sie sagen, Daten müssen geteilt werden. Solche Data Sharing Policies - haben Sie da rechtliche Aspekte die sie einerseits dazu bringen Daten auszutauschen oder auch zurück zu halten?
Interview 32		Ja, also ich meine die DFG hält sich da sehr zurück was Sonderforschungsbereiche angeht. Nach meinen Informationen, die wir bekommen haben am Anfang unserer Förderperiode, es ist so, dass die DFG ermutigt, Daten auzutauschen und selbstverständlich müssen wir unsere Forschungsergebnisse veröffentlichen und wenn es geht auch Open Access und soll da zumindest eine gewisse Open Access Quote für unsere Journal Artikel erreichen und die freuen sich über alle Daten, also Forschungsdaten austauschaktivitäten, die wir machen.	
Interview 32		Reden momentan aber, haben momentan aber keine konkreten Forderungen. Soweit ich weiß, sind die einzigen wo man wirklich konkrete Bindungen hat, wie man seine Daten austauschen muss oder verlffentlichen muss, die EU geförderten Projekte Horizon 2020 und so diese Auschreibungen. Da gibt es sehr konkrete Anweisungen, was man tun kann.	
Interview 32		Die DFG lässt es momentan noch offen, wobei, gerade bei solchen Verbundsforschungsprojekten, die so langfristig finanziert werden wie so ein Sonderforschungsbereich, durchaus sagen, sie wollen Aktivitäten sehen. Aber das geht mehr in die kreative Richtung, dass man sagt: sie möchten gerne, dass wir Konzepte entwickeln, wie wir der Meinung sind, dass es für uns sinnvoll ist, damit sie dann daraus ersehen können, was vielleicht für die Zukunft gut wäre, genau.	
Interview 32			Ah, ok.
Interview 32		Genau, aber sie erwarten von uns, dass wir uns Lösungsansätze indivuell überlegen. Wenn wir überhaupt nichts tun würden in der Richtung, können wir sicher sein, dass wir in der nächsten Förderperiode nicht weiter gefördert werden, aber was wir tun, überlassen sie ziemlich uns.	
Interview 32			Das bringt mich zum Thema, zu der Frage: Inwieweit wird das Datenteilen denn Anerkannt in der wissenschaftlichen Gesellschaft?
Interview 32	33,54	Sie meinen, inwiefern ein wissenschaftliches Prestige der einzelnen Forscher draus entsteht?	
Interview 32			Ja, genau.
Interview 32		Ich kann am besten für die sage ich mal organische Synthesechemie sprechen, da das mein Heimatforschungsgebiet ist. Und da würde ich ganz klar sagen gar nicht.	

Interview 32		Es ist schön und gut, wenn jemand dies tut und, ja, es wird gerade bei solchen Standardanalytikdaten auch dann gerne genutzt, wenn es irgendwo ist, aber dass da in irgendeiner Weise wissenschaftliche Vorteile draus erwachsen, das ist tendenziell nicht der Fall.	
Interview 32		Vielleicht mit Ausnahme der Kristallographie, von der sie sicher auch schon gehört haben - der Cambridge Cristal Structure Database, die einfach das beste Paradebeispiel dafür ist, wo es eben ganz gut funktioniert.	
Interview 32		Und ich denke in der Kristallographie wenn	
Interview 32		man eben viele Strukturen veröffentlicht hat, dann hat es eine gewisse Beudeutung, aber auch nicht so viel wie man so viel wie wenn man zu den Daten noch zusätzlich Journal Artikel veröffentlicht hat, denn auch das ist auch so, nur weil man einen Datenfile in den Cristal Data Bank reinbringt, heißt es noch lange nicht, dass man dazu auch ein Journal Artikel veröffentlichen kann.	
Interview 32	35	Dazu muss schon wirklich was Besonderes einfach sein an der neuen Struktur, dass man eindeutig hervorheben kann. Also da habe ich schon viele Leute erlebt, die frustriert sind gerade im Bereich der Proteinkristallstrukturen, da gibt es ja sowas ähnliches wie die Cambridge Cristal Structure Database, da gibt es eben die Protein Data Bank die PDB, und da ist es eben so · meine Kollegin kommt gerade rein, sorry. Guten Morgen Tanja, ich habe ein Skype Interview, aber du kannst dich gerne hinsetzten und in Ruhe arbeiten... Muss nicht, wie du magst. Genau	
Interview 32		Also was diese Proteindatabank angeht, ja, da kenne ich viele Leute, die extrem viel abeit reingesteckt haben, bestimmte Proteine in ihrer Struktur aufzuklären, zu kristallisieren und dann eben röntgenstrukturen zu vermessen und dann super froh waren, dass sie es soweit hatten.	
Interview 32		Im Endeffekt keine wissenschaftliche Veröffentlichung im Sinne eines Artikels zu erreichen konnten, weil eben so eine ähnliche Struktur schon da war.	
Interview 32		Das ist natürlich ein Gewinn ist, der dass man das wusste, der durch eben die (platform) erreicht wurde, aber wissenschaftliches Prestige hat ihnen das nicht gebracht, dass sie da jetzt die gleiche Struktur nochmal reingestellt haben.	
Interview 32		Also, das, ja, ist jetzt nicht so, dass jetzt irgendjemand gemacht hat "boah cool, der kann krass Proteine kristalisieren" und ist deswegen, ja, irgendwie weiter gekommen in der wissenschaftlichen Welt - nicht der Fall.	
Interview 32		Und so würde ich momentan denken, ist es in der organischen Synthesewelt, die ich kenne, allgemein so. Wo es vllt ein bisschen anders ist, ist glaube ich in der Welt der theoretischen Chemie, wenn man da irgendwelche Sachen programmiert. Irgendwelche Programme dann auf Github hochlädt.	
Interview 32			In der Inforamtik ist das ja eher mit Informatik mit Open Access. Und auch in der Genomforschung werden die Genome immer hochgeladen, also die Sequenzen davon werden geteilt.

Interview 32		Genau, da ist es vielleicht ein bisschen anders. Aber in der Chemie sehr wenig, genau. Ja, und in der Spektroskopie, wo es wirklich um die Neuentwicklung von Analytikmethoden, was viele unserer Leute machen, ja, die sagen wirklich immer nur: da ist das Teilen von Daten sowieso irrelevant, weil wenn man eine neue physikalische Messmethode entwickelt, produziert man so viele Mülldaten und damit kann kein Mensch was anfangen.	
Interview 32		Und die eine Messung, die dann am Ende da ist, versteht nur derjenige, der sie gemessen hat und insofern ist es, ja, Data Sharing sowieso nicht von Relevanz.	
Interview 32	38,04		Jetzt haben wir ja bereits über einige Barrieren und Probleme besprochen. Haben Sie eine Einschätzung davon, welche Probleme den größten Einfluss auf das Datenteilen hat? Also was so die größte Barriere ist?
Interview 32		Ja, also die Gewohnheit. Also das ist wirklich, also wirklich wirklich, einfach die Gewohnheit, dass man, ja, ist das Format indem man wissenschaftlich sich austauscht einfach nicht so stark d+C137atenbasiert ist, historisch gesehen sondern dass man es eben gewohnt ist, seine Forschungsergebnisse als PowerPoint zusammen zu stellen und das eben dann, das ist was man eben mit der Community teil. Und da eben nicht die verschiedenen Datenfiles als Anhang mitverknüpft.	
Interview 32		Was aber eben auch daran liegt, genau, dass auch einfach nicht die Tools dazu, die Softwaretools dazu, noch zu wenig präsent, genau. Aber insofern würde ich denken, wenn man wirklich von Anfang an den Leuten im Studium schon beibringt, wie man diese Tools nutzt und wie man sie zu seinem Vorteil nutzt, dass man dadurch eben eine Menge erreichen kann, weil man eben dann, ja, neue Gewohnheiten schafft, genau.	
Interview 32		Und dazu muss man dann eben diese Softwaretools schon in den Praktika im Studium angeschafft werden, genau. Und halt die richtigen Tools, nicht?	
Interview 32			Ja, das stimmt. Geht das so mit dem Licht bei Ihnen?
Interview 32		Ja, ich habe mich gerade so ein bisschen umgesetzt, jetzt ist es besser.	
Interview 32			Es wanderte immer nur weiter Richtung Auge. Ok. Ich gucke mal kurz, ich habe nämlich wahrscheinlich nur noch zwei Fragen offen, welche ich jetzt alle abgehandelt habe.
Interview 32	40		Also dann habe ich noch eine Frage zu: wie viele Personen kennen Sie ungefähr, die Daten mit anderen teilen? Sind das alle Daten oder nur auf Anfrage oder wie ist da so die Verteilung?
Interview 32		Sie meinen die Leute, die es aktiv tun von allen Wissenschaftlern, die ich kenne, wie viele das denn auch wirklich tun. Ich würde sagen, ja, also man teilt Daten nur wenn man sich selber einen Vorteil davon verspricht. Und das ist eine allgemeingültige Regel.	

Interview 32	Das bedeutet, wissenschaftliches Prestiges, das bekommt man durch Journal Arikel, deswegen teilt man seine Daten in Journal Artikel. Man möchte eine Kollaboration mit jemandem eingehen, deswegen erzählt man ihnen von vorläufigen Ergebnissen zu Thema X, Y, Z.	
Interview 32	Und trifft sich mit den und stellt denen die Sachen vor. Innerhalb der Arbeitsgruppe hat man momentan eben, ja, regelmäßige Treffen, in denen man sich Daten vorstellt, weil man hofft, dass man voneinander profitieren kann, wenn man auf ähnlichen Gebieten arbeitet und sich gegenseitig Tipps geben kann.	
Interview 32	Aber, ja, ich habe momentan oft das Gefühl, dass dieses willkürliche Teilen von Daten angestrebt wird - so dieses Ich gebe alle meine Daten in ein Repositorium und jeder darf sie nutzen. Aber davon hat man ja nichts, also insofern.	
Interview 32	Entweder man schafft einen ganz konkreten Anreiz, warum die Leute das tun, und das muss dann teilweise auch von den Journals sein, dass die sagen wie veröffentlichen ihren Artikel nur, nur wenn ihre Daten in Repositorien hinterlegt sind in X, Y, Z oder, ja, oder, wenn halt wirklich die Forschung drauf beruht, dass man mit anderen, das eben Datenbanken existieren.	
Interview 32	Und insofern, ja, kenne ich eigentlich nur die Kristallographen, die wirklich freiwillig ihre Daten in Datenbanken reinladen ohne dass da jetzt dieser konkrete direkte Nutzen da sind, weil die es gewohnt sind eben mit Datenbanken zu Arbeiten in ihrer Forschung und wissen, dass es ohne nicht geht. Und, ja, Kristallographen kenne ich nicht so viele, ist es das eben so eine Sache.	
Interview 32	Und ansonsten, ja sind die einzigen Leute, die halt im Alltag Forschungsdaten ungefragt - also gefragt teilen die Leute selbstverständlich ihre Daten eben im konkreten Fall wenn sie wissen warum - aber ungefragt kenne ich es eben in Form eines elektronischen Laborbuchs, in meiner alten Arbeitsgruppe, wo ich Doktorarbeit geschrieben hab, wo man halt wirklich sämtliche Ergebnisse der letzten fünf Jahren, seit dem dieses elektronische, digitale Laborbuch existierte, per Struktursuche durchsuchen konnte und sehen konnte, hat schonmal jemand eine Reaktion gemacht mit diesem Molekül? Und das sind dann eben positive und negative Ergebnisse gewesen.	
Interview 32	Da hat man dann auch gesehen "hey, das hat schon jemand versucht zu machen, hat aber nicht geklappt auf Art X, Y, Z. Genau, aber das erfordert eben ein enormes Vertrauen und im Prinzip ein Vertrauensvorschuss, den nur der Chef beschliessen kann, dass die LEute einander geben müssen.	
Interview 32	Also das kann nicht auf dem Individuallevel sein, sondern dass muss auf jeden Fall von oben vorgegeben werden in diesem Fall vom Arbeitsgruppenleiter, dass das die allgemeine Policy ist, dass man das so tut.	

Interview 32		Beziehungsweise für die größere Community der Forschungsgeldgeber wie die DFG muss eben da eine allgemeine Kultur vorgeben, dass Daten X immer in Datenbank Y hinterlegt werden. Sonst wird es einfach niemand tun und die Situation wird genau die gleiche bleiben wie bisher, dass man eben sehr punktuell Forschungsdaten teilt.	
Interview 32	44,33		Dann habe ich eine ganz andere Frage: wie schätzen Sie den Einfluss von Big Data oder Künstliche Intelligenz auf Ihre Forschung ein?
Interview 32		Momentan null, weil einfach keine Datenbanken exisitieren - Grundlage für die Nutzung von Big Data und Künstlicher Intelligenz wären. Und bis so eine Datenbank aufgebaut ist, wird da einfach sehr lange Zeit brauchen.	
Interview 32		Grundsetzlich ist es so in unseren Projekten nicht vorgesehen, KI oder Big Data zu nutzen sondern wir sind sehr auf einem naturwissenschaftlichen Erklärungsansatz auf, der ja auch den Naturwissenschaftlern normalerweise treibt, dass er sagt: "ja, wir möchten gerne verstehen, die das System funktioniert und deswegen das ganze optimieren können und nicht einen Algorithmus drüber laufen lassen" Also wirklicch die physikalischen Hintergünde aufklären.	
Interview 32		Das ist definitiv der Lösungsansatz und die, gerade so die jetzt so ganz speziell auf unser Projekt, so viele Datenpunkte zu bekommen, die wirklich konkret für unsere Fragestellung sinnvoll wären, dass Künstliche Intelligenz dann trainieren könnte, übersteigt glaube ich die Kapazitäten in unserem Fach.	
Interview 32		Und, ja. Da diese Anzahl von Datenpunkten eben dadurch nicht generierbar ist, ja, denke ich, wird es auch in Zukunft nicht dazu kommen. Und genau die Fragestellung, die wir untersuchen, untersucht ja auch niemand anders.	
Interview 32		Es ist ja nicht sodass wir jetzt sagen, da könnten wir die Daten, wenn alle Leute ihre Daten online stellen sodass wir sie nutzen können, sodass wir darauf zugriff hätten, könnten wir sie nutzen um eine KI zu trainieren, aber das ist ja eben nicht der Fall. Sondern die sind immer in irgendeinerweise unter anderen Voraussetzungen geschaffen worden und deswegen nicht unbedingt vergleichbar mit dem was wir machen.	
Interview 32		Einfach weil, weil wir eben nicht in den Idealsystemen arbeiten, sondern eben, ja, hundert Parameter sich ändern können und das ist schon innerhalb unserers Projekts schon schwierig Daten zu produzieren die Vergleichbar sind um dann daraus Schluss zu ziehen und Korellationen und physikalische Zusammenhänge.	
Interview 32		Aber da dann auch noch fremde Daten miteinzupflegen ist, wäre so komplex und so fehleranfällig, dass es unserer Meinung nach nicht zum Ziel führen würde. Also nein, kein Interesse an so einem Vorgehen.	
Interview 32			Dann vielen Dank für Ihre Einschätzung.
Interview 32			Haben Sie dann noch irgendwelche Weiteren Aspekte, die Sie mir zum Thema Data Sharing auf dem Weg geben wollen? Was Ihnen da noch spontan einfällt?

Interview 32		Ja, also zum Einen ist mir dieser Aspekt von modulbasierter Software extreme wichtig, dass das eben notwendig ist um auf einem System möglichst viele Menschen zusammenzubringen und eben interdisziplinäre Forschung zu digitalisieren - als eines und nicht auf sieben verschiedenen Platformen und da interdiszplinäre immer wichtiger wird, denke ich, dass es die einzige Möglichkeit wie man sinnvoll Forschungsdaten digitalisieren kann. Und das ist eine Sache.	
Interview 32		Ja, und zum anderen das, ja man eben sehr pragmatisch sich die Arbeitsabläufe im Alltag der Forscher angucken muss und wo es zu Zeitersparnis oder Zeitaufwand kommen kann und eben entsprechend dessen, ja, die Software anapassen. Also das heitßt, einfach nah am Forscher Softwaretools zur Digitalisierung zu entwickeln, ja.	
Interview 32		Und dann die Infrastruktur bereitstellen, die IT Infrastruktur, und das möglichst in Form von wirklich erfahrenen Teams von KLeuten, die sich damit auskennen, die vielleicht in ganz Deutschland den Universitäten zu Verfügung stellenn, die dann kommen, das dann installieren und dann festzustellen, schaffen die sich, ja um die Systeme kümmern und diese weiterentwickeln - und zwar als Experten und nicht als Leute, die das irgendwie nebenbei machen, die aber eigentlich schon 100 % an was anderem arbeiten.	
Interview 32		Wenn das so weiter geht, wird unsere, ja, Digitalisierung von Forschungsdaten nur sehr langsam voranschreiten und vielleicht auch wieder zum erldiegen kommen, weil die Leute die Motivation verlieren. Einfach diese drei Aspekte.	
Interview 32			Ja, super, vielen lieben Dank.
Interview 33	Time	Interviewee 33	Interviewer
Interview 33	0		Gut, dann habe ich Ihr Einverständnis, dass ich das Aufzeichnen darf?
Interview 33		Jawoll! Das haben Sie	
Interview 33			Gut. In dem Interview werde ich 25 offene Fragen stellen. Das bedeutet, Sie können ganz frei antworten, das was zuerst kommt, gerne mitteilen. Bei Gegenfragen auch gerne fragen
Interview 33		Ja.	
Interview 33			Haben Sie sonst noch etwas, was Sie im Voraus klären möchten?
Interview 33		Nö, da kommen wir ja drauf, wenn wir uns unterhalten.	
Interview 33			Die erste Frage lautet: was gehört so zu ihren täglichen bei (…) und was für Wissensdatenbanken nutzen Sie dabei?
Interview 33		Also, ich Projektmanager im Bereich 3D Druck und kümmere mich um die Entwicklung von Druckmaterialien im Bereich Fotopolimere für das sogenannte SLA Stereodoidperfin Verfahren. Was es - wie spezifisch wollen Sie da schon die Antwort? Was jetzt genau einzelne Arbeitspakete sind?	
Interview 33			Also bei was für Tätigkeiten sie mit Wissensmanagementdatenbanken in Kontakt kommen.

Interview 33		Also zum Einen haben wir eine sehr zentrale Funktion. Das ist der sogenannte Eitropin-prozess "Idea to Profit" heißt das mal. Das ist eine Datenbank, was auf Notes basiert, selbst zusammengedengelt sage ich mal. Es ist jetzt mittlerweile der Support für Lotus Notes ist jetzt eine webbassierte Interface geschichte, nennt sich Accolate. A C C O L AT E. Sagt ihnen der STage Gate Prozess was?	
Interview 33			Ja
Interview 33		Ok, dann braiche ich das nicht erklären, der ist dabei abgebildet in der Software. Diese Software hat mehre Funktionen. Zum einen halte ich dort den Projektfortschritt nach.	
Interview 33		Das heißt ich lade irgendwelche Dokumente hoch, ich schreibe wenn ich mich mit Kunden getroffen habe, wir dokumentieren dort zum Teil unsere Ergebnisse, aber in einer Form, dass ich einmal im Jahr im Rahmen meines Stage Gate Processes einmal dem Managementteam vorstellog werden muss und muss dann ein gewisser Template Foliensatz vortragen, der wird dort auch hinterlegt.	
Interview 33		Aber das erspare ich Ihnen jetzt, aber in dieser Datenbank sind keine Versuchergebnisse oder Auswertingen oder sowas.	
Interview 33		Was ich aber auch habe sind Checklisten, die in der Software hinterlegt sind, die auch je nach dem welches Stage ich bin, unterschiedlich sind. Das heißt. die sind je nach Projektfortschritt angepasst, wo ich quasi Aussage treffen muss: wie hat sich jemand um REACH gekümmert?	
Interview 33		Wie sieht die Wettbewerber situation aus? Was ist mit den Rohstoffen, die ich brauche um das Material herstellen zu können? Ist das verfügbar? Wie sehen da die Preise aus? Also es ist tatsächlich quasi unser komplettes Wissensmanagement, spezifisch eines Projektes in einer Datenbank drin.	
Interview 33	03:53		Ist das denn das einzige, was sie verwenden? Oder haben Sie auch noch mehrere?
Interview 33		Ja, wenn sie einen Moment warten, ich komme da gleich dazu, das ist aber nicht das einzige, was ich mit der Datenbank mache. Und zwar ist es so, es gibt gewisse Pflichtfelder, die muss ich immer ausfüllen. Das heißt, unsere Forschung wird hier in verschiedene strategic Buckets eingeteilt.	
Interview 33		Da werden sie jetzt nicht viel anfangen können: create, defend, extent und so weiter. Was der Konzern macht, zwei mal im Jahr wird hier eine Abfrage gemacht, das ist der sogennannte strategische Planungsprozess, in dessen Rahmen man gewisse Fragestellungen beantworten möchte.	
Interview 33		Quasi wie viel Geld gebe ich aus für Forschung und Entwicklung, wie viel prognostizierten Umsatz habe ich in der Forschungspipelin in zehn Jahre.	
Interview 33		Also ich habe auch meine Business Cases wuasi hinterlegt in der Software. Das heißt, der Konzern macht gewisse statistische Abfragen mit dieser Software, die sage ich mal, über mein Projektmanagement hinausgehen, die aber der Konzern braucht um am Kapitalmarkt	

Interview 33	5	sage ich mal spruchfähig sein. Das ist quasi so wie die Eierlegende wollmichsau. Die macht es auch ein bisschen kompliziert, weil sie können sich vorstellen, da gibt es tausende Felder, die ich ausfüllen muss, und die Bereitschaft vieler Menschen diese Dinge alle auszufüllen, ist nicht so hoch.
Interview 33		Das ist das eine, was wir zusätzlich haben, ist quasi für bestimmte Aufgabenstellungen hats der Konzern zentral auch verschiedenste Datenbanksysteme im Hintergrund, auf die ich zugreifen kann, wenn ich zum Beispiel wenn ich einen Marktbericht ordere.
Interview 33		Bevor ich bei irgendeinem Anbieter auf die Homepage gehe und einfach mal willenlos irgendwelche Marktberichte kaufen, haben wir eine zentrale Stile, der sogenannte Business Scout, und in dem sind hinterlegt sämtliche von den Konzern gekauften Marktbereichten.
Interview 33		Es ist so, dass ich diese Marktberichte nicht einfach kaufen kann sondern die müssen über eine zentrale Stelle besorgt werden. Das heißt es gibt tatsächlich jemanden, der so ein bisschen tatsächlich den Überblick hat - nicht dass man da ran was vorbei schummeln kann, aber grundsätzlich ist es erstmal so, die werden zentral eingekauft und dann in der Datenbank hinterlegt und jenachdem was man für eine Lizenz gekauft hat, sind die für alle frei zugänglich oder ich kriege einen Ansprechpartner genannt und sage dann "darf ich mal kurz reingucken" oder so.
Interview 33		Das ist quasi eine Datenbank für die Marktberichtssituation.
Interview 33		Wir haben auch noch ein sogenannten Info-Scout. Dieser Info-Scout ist jetzt aber eher eine übergeordnete geschichte. Das ist eigentlich nur eine Interface geschichte, die mir sagt, auf welche Datenbanken wir im Bereich Wissen zugreifen können.
Interview 33		Das heißt, das ist so ein Portal, müssen sie sich vorstellen, mit verschiedneen Links, da gibt es dann Sifinder, Link zum Scopus, es gibt einen Link beispielsweise Literaturbeschaffungsgeschichte über die Uni oder die Bibliotheken in Karlsuhe.
Interview 33		Dass ich, wenn ich irgendwie keinen Zugang habe zu irgendwelchen Online journals, dann kann ich denen irgendwas hinschicken, dann schicken die mir ein PDF zu gegen gebühr. Das sind ja auch alles Datenbanken, die wir haben.
Interview 33		Zugriff zu so, sage ich mal Datenbanken, in denen DIN Normen hinterlegt sind, wir haben zusätzlich Datenbanken, in denen Patente drinnen sind, wir haben gerade was das Thema angeht, haben wir diverseste Datenbanken.
Interview 33		Das eine ist eine Datenbank, in der wir Suchbegriffehinterlegen können zu spezifischen Themen. Dann wird dann quasi ein Rechercheur aus unserer IT Abteilung wird ein, sage ich mal, Skritpt geschrieben, das regelmäßige Abfragen bei den Patentämtern, Datenbanken usw macht.

Interview 33		Dann sucht der mir, spezifisch wenn ich dann "Photopolymere 3D Druck" haben will, habe ich dann so eine Dauerabfrage und die bringt mir einmal im Monat die neuen Patente auf. Dann hat es die Datenbank, zeigt es mir erstmal auf, ich muss die quasi selber mit der Hand selber durchsortierten, ist relevant, ist nicht relevant, und das spannende ist, die Software lernt bei diesem Prozess dazu.	
Interview 33		Das heißt, jedes Mal wenn irgendein Professor was veröffentlicht, und ich sage, das ist aber nicht relevant, irgendwann wird diese Veröffentlichung nicht mehr vorschlagen.	
Interview 33		Also es ist zum einen ein statisches System, das ich eine Suchanfrage definieren, zum anderen habe ich aber auch ein intelligentes Auswertetool, das dann sagt, ja ist zwar in der Datenbank, wird mir aber nicht als relevant nach oben geschoben.	
Interview 33			Das sind ja sehr viele Datenbanken, die Sie da benutzen. Wie häufig nutzen Sie die so?
Interview 33		Puh, das ist jetzt auch Projektphasenabhängig. Ich habe jetzt nächsten Monat mein Quasi Gate Keeper Review Meeting, das heißt, vormittags bin ich da relativ fleißig drin, weil ich jetzt halt quasi bevor ich das Meeting da habe, muss quasi alles ausgefüllt und auf dem Neusten Stand sein - das wird auch vorher überprüft.	
Interview 33	10	Das heißt uzur Zeit bin ich dort relativ fleißig drin.	
Interview 33			Das heißt, täglich?
Interview 33		Ja, also ich würde sagen zweitätig, also eigentlich jeden Tag, bare ich habe es nicht dauer offen. Das ist jetzt schwierigi zu sagen ,wie viel Zeit ich dort verbringe.	
Interview 33		Was ich auch quasi noch nicht fesagt habe, ist, wir haben ja auch diverseste Sachen wie Teams, also Microsoft Teams, in dem wir unsere Projekte organisieren, wo quasi der Kommunikative Austausch stattfindet.	
Interview 33		Hinter jeden Teams hängt auch automatisch ein Sharepoint, der in dem Sinne meine Datenbank ist um quasi die Dokumente zu speichern, wenn ich Sicherheitsdatenblätter irgendwo ablegen will von der Konkurrenz, wenn ich irgewndwlche Präsentationen, die ich irgendwo gefunden habe, das wird dort auch da alles abgelegt.	
Interview 33		Das ist ja in dem Sinn eigentlich eher ein ARchivierungssystem aber schlussendlich wieder eine Datenbank, aber nicht in dem sinne, dass ich jetzt irgendwie dann über eine Maske Sachen suchen kann. Dann muss ich schon wissen, wo die Sachen sind.	
Interview 33			Welche allgemeine Vorteile sehen Sie bei der Nutzung der Datenbanksysteme?
Interview 33		Das ist tatsächlich jetzt eine komplexere Frage finde ich, weil es gibt viele Dinge zu berücksichtigen. Zum einen ist es für mich einfach, weil ich zentrale Stellen gehabe, in denen ich schnell was nachgucken kann, ohne dass ich mir die Mühe machen muss mich durch den Konzern zu klingen: sag mal hast du denn Marktbericht schon gekauft?	
Interview 33		Für die I2P Datenbank, das Accolate, sage ich mal, sage ich mal, ist es von Vorteil, dass quasi dort drin, das innovationssteuerungstool zentralisiert wurde.	

Interview 33	Das heißt zum einen kann ich erkennen, ob ich mit den Checkliste, wenn ich Sie ausgefüllt habe, dann bin ich mir zumindest sicher, dass das, was der Konzern denkt was man braucht um ein Projekt richtig zu managen, ich alles gemacht habe. Das ist das eine.	
Interview 33	Das ist so ein bisschen "das sind die Anforderungen vom Konzern an mich als Manager um das Projekt leiten". Das andere ist aber auch, im Prinzip immer hat bei uns jedes Teammitglied Zugriff auf alle Daten. Es gibt innerhalb des Teams selber keine Unterscheidung mehr, was darfst du sehen und was darfst du nicht sehen.	
Interview 33	Was halt tatsächlich auch den Vorteil hat, wir sind viel unterwegs, dass ich muss nicht warten, dass diejenige Person wieder und was auch noch der Fall ist, ich habe zum Teil Projekte, die sind über USA, Singapur, China und Deutschland verteilt.	
Interview 33	Das bekommen Sie überhaupt nicht hin, irgendwie alle mal so sauber für eine längere Zeit an den gleichen Tisch zu bringen und dementsprechend sind solche Datenbanken im Sinne des zeitunabhängigen Zugriffs eine feine Sache.	
Interview 33	Das nächste ist, sage ich mal, ich bin in der strategischen Forschung. Das heißt, wir gucken und Realisierungszeiträume von Projekten an, die weit über das hinaus gehen, was sage ich mal, im Operativen bereich so gemacht wird - wir haben zum Teil acht Jahre.	
Interview 33	Die wir für die Projekte von vornerein als Laufzeit haben. Und in den seltensten Fällen erlebt man bei solchen Projekten, dass man das anfängt und auch fertig macht.	
Interview 33	Also es gibt Leute, die auch länger bleiben, aber in der Regel ist die Verweildauer bei uns drei bis fünf Jahre. Und wenn Sie quasi gehen und der Nachfolger oder die Nachfolgerin kommt, haben Sie den Vorteil, dass sie mal an der zentralen Stelle alles abgelegt haben.	
Interview 33	Es gibt hier auch faktisch keine Schrankwand mit Leitzordnern, in denen irgendwie gefühlt das Wissen der letzten Jahrunderte aufgewahrt wird.	
Interview 33	Sondern es ist halt alles Spezifische der Projekte in den Datenbanken hinterlegt, sodass ich es a leicht finde und b wirklich - ich kenne es tatsächlich noch von meinem vorherigen Job, da hatten wir die Schrankwände voll, da sehen sie erst gar nicht durch, da machen sie sich gar nicht erst die Mühe und gucken da Seite für Seite jeden Ordner durch.	
Interview 33	Sondern da gehen Sie ans Internet, googlen irgendwas, oder suchen es neu um Scifinder aber sie machen sich nicht die Mühe und machen da 30 Leitzorder durch.	
Interview 33	Also das dauert weit länger, als das was sie brauchen, wenn sie die Datenbank durchwühlen.	
Interview 33	Der große Vorteil dieser Datenbanken sehe ich tatsächlich, man hat ein öffentliches Wissen, das zugreifbar ist, und was ich vorhin auch schon angedeutet hatte, der Konzern hat natürlich in seiner Kontrolling funktion es einfacher, wenn ich solche zentralen Datenbanken habe, wenn sie, sage ich mal, gut gepflegt sind, kann ich damit auch wirklich was anfangen.	

Interview 33		Also wie gesagt, früher, das kenne ich auch noch, ich bin jetzt Zseit fastneun Jahren in der Firma, der strategische Planungsprozess lief ganz am Anfang noch so, dass es eine Person gab, die beuaftragt war, die musste von Projektmanager von Projektmanager laufen und die DAten per Hand sammeln.	
Interview 33		Und ja, das ist jetzt wie gesagt keine Jahrzehnte her, das ist jetzt so sechs sieben Jahre her, und jetzt muss ich kurz überlegen.	
Interview 33	15	Und jetzt sagt halt einer bei uns im Corporate Center und ich möchte das jetzt abfragen. Und das ist dann auch zeitunabhängig, weil er kann es, wenn er will, fünf mal am Tag abfragen. Der drückt auf sein Knöpfchen und dann spuckt ihnen die Software automatisch aus die Zahl, die er haben will.	
Interview 33		Also was wie, was ich viorher beschrieben habe, Umsatz in 20 Jahren oder Umsatz in 10 Jahren entsprechend der Business Cases die hinterlegt sind in den Projekten. Das funktioniert halt aber nur, wenn die Leute es pflegen.	
Interview 33			Und damit komme ich zu einem weiteren Aspekt, sie haben ja jetzt schöne Vorteile aufgezählt. Welche Probleme und Barrieren treten denn bei der Nutzung der Systeme auf?
Interview 33		Also grundstätzlich, sage ich mal so, für mich persönlich treten häufig Probleme auf, wenn es mehrere reduntande Systeme gibt, die ich parallel pflegen muss. Das heißt zum Beispiel habe ich mein Teams mit dem Sharepoint, ich habe das Accolate für das I2P, ich habe dann meintewegen noch eine ID in der Connections gruppe. Ich weiss nicht, kennen Sie IBM Connections?	
Interview 33			Nein
Interview 33		Das haben wir nämlich auch noch. Das ist quasi ein Facebook für Firmen. Da hat jeder sein Profil und dann gibt es Gruppen zu allen möglichen Themen; da können sie sich austauschen.	
Interview 33		Da ist ein Blog dabei, da können Sie Daten hochladen und so weiter. Da haben wir dann auch für jedes große Thema auch eigene Gruppen. Das heißt am ende des Tages habe ich fünf Sachen parallel.	
Interview 33		Und dann gibt es noch Email, die ich auch bespaßen muss, weil es halt einfach Leute gibt, die weigern sich das eine zu nehmen. Dann muss ich ihnen trotzdem nochmal eine Email schreiben, obwohl ich das bereits an zwei Stellen plaziert habe.	
Interview 33		Dieses Problem des doppelt pflegens, und das führt glaube ich dann auch dazu, dass viele - da schließe ich mich mit ein, an vielen Stellen sich auch weigern in Anführungszeichen das alles auszufüllen, warum soll ich es denn zehn mal irgendwo eintippen. Andererseits, da lege ich jetzt gerade usner Accolate, ich mag es tatsächlich sehr, aber wo ich ein nachteil dirn sehen könnte, es ist zu komplex.	

Interview 33		Das heißt, wie gesagt, dieses System sammelt eigentlich alles. Theoretisch brauchen Sie gar nichts anderes mehr als dieses System. Da müssen sie auch keine Emails mehr schreiben, wenn die Leute da jeden tag reingucken. Aber es überfordert auch, wenn man da sage ich mal frisch drauf losgelassen wird, weil sie da auch wirklich alles machen.	
Interview 33		Sie müssen jeden Kram da rein pflegen, damit es hinterher funktioniert. Und das ist viel Arbeit. Das ist sage ich mal ähnlich wie wenn sie auf einen Messebesuch gehen und dann den Messebericht nicht schreiben, weil der ist lästig, bloß in drei Wochen können Sie sich leider nicht mehr dran erinnern, was sie alles mit wem besprochen haben.	
Interview 33		Dafür brauchen Sie dann ihren Messebericht. Genauso ist es dann mit ihren Datenbanken, wenn sie die nicht sauber pflegen - was viele nicht tun -dann ist eine Datenbank auch eher hinderlich, weil sie sich nicht darauf verlassen können, dass die Daten, die drin stehen, aktuell sind oder wer auch immer da reingeschrieben hat, wenn zu viele Leute Lese und schreibrechte haben, sind sie sich auch nie sicher ob nicht jemand irgendwas daran rumgemacht hat.	
Interview 33		Das heißt sie brauchen eine Nachhaltung, wer wann was mit ihrem Dokument gemacht hat - das sehe ich auch nicht in jeder Datenbank.	
Interview 33		Das ist wie gesagt ein Nachteil, den ich sehe, von solchen sage ich mal Eierlegende Wollmilchsäue, die so viel ineinander reingebastelt kriegen, dass sie nicht mehr spezifisch sind und ich dann nicht mehr funktional empfinde.	
Interview 33		Wo ich tatsächlich nur wenig Nachteile sind solche spezifischen WissensDatenbanken. Das heißt irgendwieso der Business Scouting geschrieben hat für die Marktberichte, die sind da llle hinterlegt.	
Interview 33		Klar, auch da ist es so, wenn ich jetzt heimlich und leise mir den Marktbericht an dieser Abteilung vorbei beschaffe, dann ist er da nicht drun, aber ich glaube zumindest, ist mein EMpfinden, bei uns in der Firma wird dieser Business Scout sehr stark und gut angenommen.	
Interview 33	20	Weil sie müssen halt auch sehen, so eine Marktberichtsdatei, die kostet nicht 3000-4000 Euro, die will keiner fünf mal ausgeben. Und dementsprechend ist da auch ein eigenes Interesse daran, daran mitzumachen.	
Interview 33			Sehen Sie denn noch weitere Probleme und Barrieren bei der Nutzung?
Interview 33		Wir hatten, das ist eigentlich ganz Lustig, das darf man keinen wirklich erzählen, aber ich glaube für ihre Forschung ist das relevant. Ich sage mal, jeder hat so eine Lieblingsbrowser.	
Interview 33		Und zumindest unsere IT hat bevor wir jetzt auf Windows 10 umgestellt wurden, den Chrome nicht gemocht. Das heißt alles lief über den Internet Explorer, den ich persönlich wiederum nicht mag, weil ich den sehr unkomfortable finde, aber wie gesagt, manche Datenbanken gingen halt nur auf Internet Explorer.	

Interview 33	Das heißt, da muss ich jetzt den Intenret Explorer aufmachen - da habe ich kein Bock drauf. Und dann habe ich einfach die Datenbank nicht genutzt.	
Interview 33	Das hört sich lustig an, aber ich will da offen sein mit Ihnen, das sind so die klassischen, täglichen Probleme, die man gar nicht mal wirklich an was fixem festmachen kann, aber wie gesagt, ich mag den Internet Explorer nicht, also versuche ich den zu vermeiden.	
Interview 33	Und wenn das dann heißt, ich kann auf eine Datenbank nicht zugriffen, ich aber sage ich mal über google mit ein bisschen mehraufwand auch woander herkriege, ja dann mache ich das	
Interview 33		Und welcher der genannten Barrieren hatte den größten Einfluss?
Interview 33	Können Sie die Frage bisschen spezifizieren?	
Interview 33		Also welcher der genannten Barriere hatte den größten Einfluss auf die Nutzung? Jetzt haben Sie ja erzählt mit dem Browswer, war das die größte Barriere, die sie hatten bisher?
Interview 33	Ne, tatsächlich ist meine größte Barriere bei, das was ich davor beschrieben hatte, dass es Leute gibt, die gewisse Tools nicht nutzen und ich dann doppelte Arbeit machen muss und ich dann irgendwann kein Bock mehr drauf habe.	
Interview 33	Das heißt, es liegt weniger in der Nutzungsfähigkeit der Datenbanken selber, sondern, sage ich mal, zu viele Baustellen gleichzeitig und dadurch macht man es nicht. Also wier gesagt, ich fülle es nicht fünf mal in fünf verschiedenen Datenbanken aus und dementsprechend ist das ein Hindernisgrund dass ich zum Beispiel Teams nicht so gerne nutzen, weil ich weiß, die Leute gucken dort eh nicht rein.	
Interview 33	DAs Ding ist, wissen Sie, da hat jemand drin gearbeitet. In den ganzen Datenbanken ist es eher darauf ausgelegt, dass man kollaboratives Arbeiten macht und dann funktioniert es nur, wenn jeder täglich das Ding dauer offen hat und sieht sofort "ah hier wurde ich erwähnt" oder "hier ist ein neues Dokument hochgeladen worden".	
Interview 33	Wenn sie aber, sage ich mal, von ihrem Arbeitsstil anders arbeiten und da irgendwie alle zwei Wochen reingucken, dann funktionieren diese neumodischen Digitalisierungswerkzeuge.	
Interview 33	Und ich glaube wir sind halt noch so in einer Phase, da haben wir Leute in einem Konzern arbeiten, die sehr technik affine sind und Leute, sage ich mal, alles in ihre Büchlein schreiben.	
Interview 33	Und solagen sie, sage ich mal, noch so uneinheitliche Arbeitsweise vorfinden, wird es immer Probleme geben, dass die Akzeptanz für solche Softwarelösungen nicht groß sind.	
Interview 33		Wenn Sie jetzt die Möglichkeit hätten, etwas an dem System zu ändern. Welche Aspekte würden Sie persönlich denn bei der Nutzung der Systeme ändern?
Interview 33	Ich würde die Nutzungsmöglichkeiten einschränken. Also ich würde zum Beispiel mal die Hälfte der Sachen abschaffen. Das heißt, es gibt quasi den Sharepoint nur noch mit Teams und nicht mehr ohne.	

Interview 33		Ich würde dann auch sagen, also persönlich nur noch mit externen Emails schreiben, intern würde ich nur noch über kollaborativen Datenbanken arbeiten und nicht den öLeuten tausend Möglichkeiten geben.	
Interview 33		Wie gesagt meistens, meiner Meinung nach, scheitert es nicht an der Programmierung selber sondern wie man mit diesen Datenbanken arbeitet. Also der Faktor Mensch ist glaube ich das größere Probleme als die IT?	
Interview 33	25		Also haben Sie denn technische Probleme?
Interview 33		Unser Internet ist langsam, aber das hat mit der Datenbank nichts zu tun. Das wäre, sage ich mal, auch so ein Punkt. Wenn Sie, also die Schwierig ist, ja ich kann von jedem Ort der Welt darauf zugreifen, aber nur wenn ich Internet habe.	
Interview 33		Das heißt, wenn ich nichts mehr lokal speichere sondern nur noch quasi in der Cloud ider auf einem Server oder wo auch immer, wenn sie nicht mal kein Internet haben, dann können Sie, dann brauchen Sie garn icht mehr arbeiten.	
Interview 33		Sie sitzen am Flughafen und warten zwei Stunden, weil es Verspätung gibt, und es gibt kein gescheites Internet, dann haben Sie gelitten. Das ist so ein bisschen auch ein Nachteil, den ich in den Datenbanken sehe.	
Interview 33		Wenn die Datenbank lokal bei mir auf meinem Rechner ist, dann ist das kein Thema, dann sind die Daten ja auch immer da.	
Interview 33		Sobald ich in einen Teilungsmodus gehe, dass ich quasi Laufwerke teile, das ich den Sharepoint teile, die Datenbank zentral irgendwo ist, dann brauchen Sie Internet und zwar gutes mittleriweile.	
Interview 33		Weil selbst so eine dumme PDF Datei mittlerweise Megabyte groß ist, das ist glaube ich auch eine Schwierigkeit an diesem Arbeiten.	
Interview 33			Und beeiflusst die interne Abteilung? Also inwieweit geht die interne Abteilung auf die Nutzer der Systeme ein und unterstützt diese?
Interview 33		Also wir haben eine unglaublich große IT-Abteilung. Ich glaube auch, es gibt da -also über IT kann man immer meckern. Ich glaube, das ist so ein grundsätzliches Thema, genauso wie die Personalabteilung, weil sie es halt tatsächlich persönlich den ganzen Tag betrifft, wenn das Ding zu langsam ist, das trifft sie täglich.	
Interview 33		Was es bei uns gibt, wie gesagt ich habe ja von diesem IBM Connections erzählt, da gibt es Foren zum Beispiel wie ich am besten die einzelnen Tools nutzen kann. Es gibt da Tutorials zum Herunterladen, es gibt PowerPointpräsentationen, es gibt quasi so FAQs dort zu den einzelnen Sachen, es gibt irgendwleche Workarounds oder Best Practices können Sie sich angucken in dem Blog.	
Interview 33		Also dam uss ich tatsächlich sagen, macht die Firma sehr viel um mir dieses Datenbarnksarbeiten so leicht wie möglich machen. Wenn Sie gleich wieder fragen, was könnte man da verbessern, würde ich sagen, ist an der Stelle auch hier ist es alles ein. ich muss es mir holen, also der Konzern stellt es zwar bereit, aber ich muss aktiv, sage ich mal so, da dann suchen.	

Interview 33		Mir würde persönlich, aber wie gesagt das ist eine persönliche Ansicht, besser gefallen, wenn es mal eine oder zwei Stunden Tutorial gibt, wo ich mich einloggen kann, dann zeigt mir mal so ein Trainer, wie ich das Tool zu benutzen habe am besten.	
Interview 33		Das ist für mich besserm wenn ich so eine 30 Seiten PowerPoint lesen muss. Also wie gesagt, da wird bei uns doch sehr viel wert auf selbst erarbeiten gelegt, von dem her, dass, hätte ich jetzt gesagt, wäre so ein bisschen der Nachteil.	
Interview 33	30	Aber an sich, wenn ich ein Problem habe, dann kann ich jemanden anrufen und es wird in der Regel gerichtet. Also da sehe ich jetzt wenig Probleme, dass die Firma quasi was anschafft und einen dann im Luftleehren raum zurück lässt.	
Interview 33			Sie haben jetzt gesagt, dass es eher so ein aktives holen, welche Prozesse müssen denn durchlaufen werden um Zugriffe auf die Systeme zu bekommen und wie wird der Vorgang dann empfunden?
Interview 33		Das ist jetzt ein umschichtiges, also das ist jetzt keine einfache Antwort, weil das von Datenbank zu Datenbank unterschiedlich ist.	
Interview 33		Es gibt Datenbanken, das heißt, wenn Sie hier anfangen zu arbeiten, dann bekommen Sie eine sogenannte Konzern ID. Also Sie haben eine Personalnummer für den ganzen Gehaltkram und so weiter und es gibt eine Konzern ID, die brauichen sie für die It.	
Interview 33		Und mit dieser Konzern ID kriegen sie eigentlich, da können sie allen möglichen Kram hinterlegen.	
Interview 33		Das heßt die Standardsachen wie das I2P und Accolate Datenbank, die sind bei uns automatisch freigeschaltet, weil damit jeder arbeiten muss und soll. Es gibt aber auch Datenbanken,	
Interview 33		Datenbanken, da muss ich vorher regisitrieren, da ist es jetzt so ein bsischen abhängig, ist das ein esxterner Anbieter oder ist das intern ein Anbieter für die internen Sachen.	
Interview 33		Habe ich ein IT Tool, bei dem ich mir Services freischalten lassen kann? - da ist es nur ein Klick und dann schick ich es weg und dann habe ich irgendwie, geführt nach dem der Server durchgelaufen ist, am nächsten Tag den Zugang.	
Interview 33	35	Also es ist relativ einfach diese Zugänge zu kriegen. Es ist manchmal nicht ganz so einfach zu finden, wo ich den Zugang beantrage.	
Interview 33			Darauf dann tiefergehend: inweiweit beeinträchtigen denn die rechtliche Aspekte, also zum Beispiel auch die Lizenzen, die Nutzung der Systeme?
Interview 33		Da muss ich mir keine Gedanken machen, das ist alles im Hintegrund geklärt. Wir haben Konzernlizenzen für die allermeisten Sachen. Also Zugang habe ich eigentlich grundsätzlich überall.	
Interview 33		Manchmal ist es nur eine Frage, wie wird abgerechnet. Bei Reaxys ist es so, da wird abgerechnet pro Suchanfrage, dich ich dann stelle, aber ich komme grundsätzlich erstmal rein in die Datenbank. Beim Scifinder haben wir eine Flatrate.	

Interview 33		Wie gesasgt, dass ich so ein bisschen Datenbanksabhängig, aber Lizenzprobleme habe ich eigentlich nie, da muss ich mich nicht drum kümmern.	
Interview 33		Also wenn ich irgendwann in diesem IT Portal gesagt habe, ich möchte diese Datenbank haben, dann ist automatisch auch die Lizenz geklärt.	
Interview 33	36:12:00		Und dann gibt es dann finanzielle Barrieren bei der Nutzung? Sie meinten das kostet dementsprechend pro Suchanfrage. Oder ist das für Sie als Nutzer egal?
Interview 33		Ja, das ist eine gute Frage. Ich würde sagen, der Punkt kann man so entscheiden. Normalerweise ist eigentlich, man hat ja nicht wirklich einen Vergleich, was so einen Zugang kostet im Jahr. Das ist den Chefs, die dann Budget verantwortung haben, dann klar.	
Interview 33		ICh weiß nicht, wie so ein Scifinder - das haben Sie nicht automatisch bei uns. SOndern den muss ich beantragen, mein Chef muss zustimmen.	
Interview 33		Das heißt, wenn mein Chef der Meinung wäre, dass es die Kosten nicht Wert ist, weil ich da eh nur alle zwei Wochen al reingucken oder weiss nicht wie noch länger. Dann würde ich gar nicht erst den Zugang kriegen.	
Interview 33			Das ist passend auf meine nächste Frage: Welchen Einfluss hat das Management auf die Verwendung der Systeme?
Interview 33		Auch da sage ich mal, gibt es gewisse Vorgaben. Es gibt Kontraktionszwänge, dass ich zum Beispiel das Accolate nutzen muss. Ich kann nicht einfach entscheiden "für mein Projekt hier hätte ich jetzt aber gerne eine andere Softwarelösung, sondern das ist eine Koponzerneinheitliche Geschichte.	
Interview 33		Dadurch, wie gesagt da der Konzern auch diese Abfragen fährt, ist ihm auch sehr daran gelegen, dass es quasi Vorgaben gibt, dass es dieses System einheitlich gibt, und dass auch jeder Zugang. Das geht dann Hand in Hand an der Stelle. Was die anderen Sachen angeht, wie gesagt, es gibt auch eigentlich eine Verpflichtung, zum Beispiel, diesen Marktreport datenbank zu nutzen.	
Interview 33		Damit halt quasi über alle Köpfe hinweg die Kosten so klein wie möglich sind. Was wir noch gar nicht berücksichtigt hatten, es gibt noch zwei Datenbanklösungen, die eher was fürs Labor. Zum einen ist es quasi so ein elektronisches Laborjournal, gibt es bei uns, in dem wirklich die ganzen Versuche dokumentiert sind.	
Interview 33		Auch hier gab es einen Kontraktionszwang, dass wir jetzt in der neusten Version, Konzerneinheitlich eine Software verwenden sollen. Also das heißt jetzt tatsächlich nicht, dass es auch schlussendlich so ist, aber in den, sage ich mal, 90 %iger Wahrscheinlichkeit werden die Leute diese Datenbank dann nutzen.	
Interview 33		Und es gibt eine sogenannte Berichtsdatenbank. Das heißt, wenn die Forschung berichte schreibt, werden die auch zentral verwaltet. Allerdings kann ich dort sehr stark den Nutzerkreis einschränken.	

Interview 33		Das heißt, man findet vielleicht noch den Titel, aber Sie finden mal ein Deckblatt oder irgendwie den Bericht an sich, den kriegen sie dann nur wenn sie wiederum mit dem Autor des Berichts in Kontakt treten.	
Interview 33		Das ist dazu. Wie gesagt, Datenbanken laufen Ihnen hier zahlreich über den Weg. Ich sage jetzt mal so, die Frage ist auch, was ist eine Datenbank?	Ok
Interview 33		Ab wann ist ein Laufwerk, auf dem ich Dokumente ablege, ist das schon eine Datenbank oder muss eine Datenbank durchsuchbar sein?	
Interview 33	35		Ich selber beschäftige mich dann ja auch mit solchen Wissensmanagementsystemen, und da haben Sie mir auch einige genannt. Und da bin ich wirklich erstaunt, wie viele unterschiedliche Lösungen es gint. Sodass mich jetzt interessieren würde, welche Kultur in Ihrem Unternehmen vorliegt? Würden Sie sagen, es ist eher so Prozess-, Ergebnis-, oder Mitarbeiterorientiert?
Interview 33		Das ist ein Prozessorientiertes System, würde ich sagen. Also es ist wirklich, wir fahren hier ganz strikt den I2P Prozess nach. Beziehungsweise was wir zuzeit implementieren, es gibt neben dem I2P auch den sogenannten IEA Dicovery Incubation Exaleration.	
Interview 33		Ist aber in Prinzip ein Softwaregestütztes System. Das wird auch noch etabliert für die sage ich mal disruptive Projekte, die, also ein Stage Gate Prozess ist Ok, ich glaube aber auch, man kann disruptive Dinge damit steuern.	
Interview 33		Aber manchmal ist es einfach, sage ich mal, da ist das I2P, das passt hervorrangend für einen großen, deutschen Großkonzern- mit den Strukturen, Entscheidungswegen und so weiter. Wenn sie abertatsächlich lean, agil und wie alle fancy Wörtlein heißen, wenn Sie das sein wollen, dann funktioniert I2P, weil ich muss ja theoretisch, bis ich eine Entscheidung kriege, warten bis ich mein nächstes Gate Meeting oder mein nächstes Review meeting habe.	
Interview 33		Das kann ich nicht einfach festlegen sondern diese Termine sind quasi ein Jahrlang im Vorraus schon festgelegt. Dementsprechend ist dann quasi manchmal es besser, wenn man eine andere SoftwareLösung habe.	
Interview 33		Jetzt stellen Sie vermutlich die Frage, wer entscheidet jetzt, ist mein Projekt schon fancy genug, das es ein disruptiv Projekt wird oder ist das ein I2P Projekt? DAs sind dann so ein bisschen die schwierigkeiten, da kommt dann so der Mensch dann wieder rein.	
Interview 33		Aber grundsätzlich ist auch agil oder Scrum oder wie sie alle heißen, das sind sehr prozuale Dinge, weil man muss sich schon an gewisse Abläufe halten, dementsprechend sind wir hier eher prozessual unterwegs was das Management angeht, aber mit der Einschränkung, dass es sehr	

Interview 33		menschelt, weil wie gesasgt, es steht und fällt bei diesen Datenbanekn, ob die Leute es annehmen, ob sie es betreiben, ob sie da drin sich wiederfinden und die Sachen eintragen. Und das, wenn die Leute ihr system nicht füttern, aus welchen Gründen auch immer, sei es sie wollen nicht teil - alles meins - so Leute gibt es ja auch, die würden nie eine Datenbank benutzen. Das sind halt die Nachteile.	
Interview 33			Sie haben jetzt angesprochen: "nichts teilen". Inwieweit wird denn Wissen im Unternehmen anerkannt? Also neues zu generieren und zu nutzen?
Interview 33		Ja, also das ist spannend. Da kann man, kommt es ein bisschen glaube ich auf die Sichtweise drauf an. Zum einen ist es nach außen die Darstellung ist häufig eine andere als nach innen.	
Interview 33		Das heißt quasi nach außen, werden da wirklich nur die shiny, shine, shine sachen gezeigt und nach innen sind es halt auch die failures. Von dem her ist die Anerkennung von Forschung so ein bisschen geteilt.	
Interview 33		Extern sieht man, gerade uns als strategische Forschung, weil wir halt da nach extern, gehe ich wirklich nur und zeige eins von hundert Projekten, die dann wirklich mal gelungen sind,	
Interview 33		Dann sehen sie natürlich immer nur irgendwelche Erfolgsgeschichte, die auch wirklich fancy sind. Da ist die Anerkennung relativ groß.	
Interview 33		Intern, sage ich mal, also das ist bei Forschung allgemein so,	
Interview 33		müssen Sie sich immer rechtfertigen, dass das ganze Geld, was sie ja ausgeben, irgendwann auch wieder reinkommen muss, dass das sehr viele Leute immer anzweifeln.	
Interview 33		Das heißt, das ist das eine. Zum anderen hat aber die Firma sich bei uns jetzt auch, fragen Sie mal, wie viel XY, sie können wenn sie nicht in der Forschung Karriere machen wollen, gibt es tausende Stufen, sage ich jetzt mal	
Interview 33		Mittlerweile gibt es aber auch für die, ausgewählte Forscher	
Interview 33		sogenannte Expert Carriers, die dann nochmal anderweitig honoriert wird.	
Interview 33	40	Also quasi jemand, der fleißig forscht, und das publiziert, hat auch nun mittlerweise innerhalb der Firma eine höhere Sichtbarkeit zu kriegen und auch, sage ich mal, man macht zwar weiterhin sein Job, aber kriegt dann mehr Gehalt als die Stelle eigentlich hergibt.	
Interview 33			Also schon zwiegespaltene Anerkennung in den Bereichen.
Interview 33		Ja, ich glaube innerhalb der Forschung haben Sie überhaupt kein Problem mit der Anerkennung.	
Interview 33		Schwierig wird es so ein bisschen, wie gesagt, innesicht-, außensicht, ist glaube ich dann der Unterschied. Weiß nicht, wie häufig wird man von extern gelobt: "oh ihr habt ja so einen schönen Prozess und ihr fahrt ihn so stringend durch und das gefällt und sehr gut und ich würde das ja auch gerne machne" aber naja, sind wir mal ehrlich , wenn man mal extern geht es sind halt wirklich alles pole shows und die Realität sieht dann doch auch anders aus.	

Interview 33		Zum Thema Innen- und Außensicht: Sie sind ja jetzt sehr positiv den ganzen Systemen gegenüber. Inwieweit denken Sie, betrachten auch andere diese Systeme als sinnvoll?
Interview 33	Definieren Sie mal bitte andere.	
Interview 33		Also Sie sagen ja, sie nutzen es sehr gerne. Haben Sie auch mitbekommen, dass auch andere diese Wissensmanagementsysteme als nicht sonnvoll erachten?
Interview 33	Ja, ich sage mal so: die Forschung nimmt sowas eigentlich sehr gerne auf. Wo es hapert ist, und das ist tatsächlich eine gelebte, reale Erfahrung von mir, das Marketing macht da meistens nicht mit. Also füttern tun diese Datenbanken meistens nur die Forscher, aber wenn da irgendwer dran geht: ja wie viele Tonnen verkaufen wir denn jetzt in der Zukunft zu welchem Preis?	
Interview 33	Das ist die Bereitschaft des Marketings normalerweise nicht so hoch das zu pflegen. Das hat diverste Gründe, unter anderem ist es so, wenn mal eine Zahl irgendwo steht, dann wird man immer darauf festgenagelt. Ob die jetzt dann jetzt danach mit gutem Grund revidiert wird, ist vollkommen egal, oder wenn da mal steht "5 Euro pro Kilo verkaufe ich das"- dann ist diese Zahl da.	
Interview 33	Und deshlab traut sich eigentlich nie irgendjemand von der Marketingabteilung irgendwie Preise zu nennen, weil man danach darauf festgenagelt wird. Das ist so ein bisschen, glaube ich, in der XY Plattens Form erklärt. Also wie gesagt, die Forcsher habe ich noch nie erlebt, dass da groß Leute nicht die Ergebnisse ihrer Experimente dokumentieren wollten.	
Interview 33	Es ist eher auf der anderen Seite, dass wenn es dann halt mal ernst wird - wissen Sie, solange Sie forschen, ist noch nichts kaputt, da geben Sie ein bisschen Geld aus, .. aber real ist da erstmal nichts. Wenn ich jetzt aber quasi nicht diese fünf Euro nicht birnge sondern nur vier, dann sagt mir aber einer: aber hier vor fünf Jahren hast du doch mal in der Datenbank hinterlegt, ich glaube du kannst nicht verkaufen, warum hast du die 5 nicht?	
Interview 33	Das ist ein ganz anderer Stress, den die Leute kriegen, als wenn ich hier zehn Experimente mehr braiche.	
Interview 33		Das heißt unter welchen Umständen würden Sie kein Wissen mit anderen teilen?
Interview 33	Also ich bin da tatsächlich, glaube ich, einer der, ich geb lieber fünf mal, also, fünf mal Sachen zu viel als zu wenig. Wobei ich persönlich die Erfahrung gemacht habe, dass man durch das Teilen von Informationen viel mehr zurück kriege, als wenn ich es nur autrage.	
Interview 33	Von dem her hätte ich gesagt, es gibt tatsächlich nur sehr wenige Sachen, die ich nicht teilen würde. Das eine sind personlle Dinge. Also, ich habe ein Mitarbeitergespräch gehabt und da hat mir der Mitarbeiter irgendwie erzählt, ihm geht es nicht gut und er will eine andere Stelle.	

Interview 33		Sowas würde ich niemals in einer Datenbank hinterlegen. Und das andere ist, sage ich mal, ein bisschen abhängig davon, wie die Systeme gestrickt sind.	
Interview 33		Es gibt ja hier, also gerade bei uns ist es extreme häufig der Fall, ich habe Forschungskooperationen, von denen kein Mensch irgendwas wissen darf, weil die so Front End gelagert sind, dass wir auf Teufel komm raus, dass wir verhindern wollen, dass was ich,wie (Firma) oder (Firma) oder sonst wer davon Wind bekommt.	
Interview 33		Problem bei Solchen Datenbank, auf die jeder Zugriff, auf die jeder Zugriff hat oder haben kann, weil es ja eigentlich nur ein Häkchen in der Software ob jemand darauf kommt oder nicht, ist dass ich halt den Kreis, der Informationshaber einschränken kann, wenn ich es nicht in die Datenbank mache.	
Interview 33		Sobald sie in der Datenbank ist kann irgendjemand im Konzern die Abfrage machen und dann hat er die Daten. Das ist so ein bisschen glaube ich tatsächlich so ein Punkt wo ich sagen würde, aber ich kann ihnen da nichtmal ein konkretes Beispiel nennen, das würde ich niemals irgendwo hinschreiben, weil ich nicht möchte, dass es geteilt wird.	
Interview 33	45	Das ist dann eher eine Frage, ab welchem Zeitpunkt teile ich. Ich glaube grundsätzlich würde ich alles teilen nur manchmal ist es die Frage, schreibe ich es direkt sobald ich es mitgekriegt habe oder warte ich damit noch drei Monate bis ich mir dazu ein bisschen mehr Gedanken gemacht habe und dann ein besseres Bild reintragen.	
Interview 33			Um nochmal auf die Kultur zurückzukommen. Nun haben Sie ja gesagt, dass sie ein sehr prozessgetriebens Unternehmen sind. Inwieweit glauben Sie, dass das die Kultur im Unternehmen beeinflusst wie Wissensdatenbanken verwendet werden?
Interview 33		Ich glaube wenig dass man als Konzern das verordnen kann. Die Leute müssen selber den Vorteil drin sehen. Von dem her, Kultur ist wichtig, ich glaube aber auch, wie gesagt, das ist keine zentrale Geschichte ist, sondern das muss der Chef oder die Kollegen müssen es vorleben.	
Interview 33		Das heißt, wenn jetzt neue Leute ankommen in der Abteilung, um Gottes willen, trage es nicht ein, das wird gegen einen verwendet, dann wird derjenige es auch nie eintragen - und auch später wahrscheinlich nicht, weil er es nie gelernt hat damit zu arbeiten.	
Interview 33		Kulturtechnisch ja, defintiv, ob das eine von oben verordnete Kultur sein muss, das bezweifle ich.	
Interview 33			Jetzt kommen wir ja auch schon so langsam zu den letzten Fragen. Ich weiß nicht wie Sie danach noch Zeit haben.
Interview 33		Wir müssen nicht sofort aufhören, aber ich glaube um halb spätestens ist der nächste Termin.	
Interview 33			Nein, alles gut, das sind wirklich nur noch zwei Fragen soweit.

Interview 33	Ne, wir gesagt, wenn es drei sind ist es auch Ok. Ich glaube es ist wichtiger, dass Sie mit ihrem Fragebogen durchkommen, weil sonst haben Sie am Schluss eine Problematik, wenn nicht jeeder alle Fragen beantortet hat, dann können Sie niciht mehr auswerten. Die Zeit haben wir.	
Interview 33		Gut. Zurückkommend auf Zwischenmenschliche- und Teamaspekte: Welche Teamsfaktoren sprechen dafür oder dagegen, ein Wissensmanagementsstem zu verwenden?
Interview 33	Können Sie ein Beispiel für ein Teamfaktor nennen?	
Interview 33		Ein Teamfaktor in dem Sinne ist vielleicht, dass jemand damit nicht umgehen kann. Er muss es halt, aber irgendwie muss man die ja ausgleichen.
Interview 33	Was ich sehe ist tatsächlich, für solche, so kollaborative Sachen, ist es zwingend nötig, dass jeder Zugriff hat erstmal. Sowas funktioniert nie wenn ich sage: ne, nur der Gruppenleiter hat zugriff.	
Interview 33	Das bringt es nicht. Das heißt ich muss quasi sicherstellen dass eine ausreichende Anzahl an Lizenzen verfügbar ist für diese Datenbanken und ich glaube auch, man darf das nicht zu sehr über das Geld argumentieren, sondern den Mehrwert, den diese Datenbanken ergeben, berechnet sich nicht in EPID, oder auch nicht in Ersparnis, in Geld, weil ich in der Regel Zeit als Währung hier haben.	
Interview 33	Und ich glaube, man muss einfach den Leuten sehr gut erklären, dass wenn Sie diese Datenbanken tapfer nutzen, dass Sie über alles einen Mehrwert haben, und das wird glaube ich zu wenig gemacht.	
Interview 33		Nun gibt es ja auch noch weitere Kommunikationswege als über solche Systeme. Inwieweit werden auch alternative Kommunikationswege genutzt anstatt sie in die Datenbank zu schreiben. Sie haben ja schon angesprochen, dass sie Emails.
Interview 33	Emails, ja genau. Wir sind da momentan zumindest bei und in der Firma in der Phase, das wir viele Dinge doppelt haben, das weiß ich. Das schlimme ist dann auch ich weiß gar nicht mehr: habe ich das jetzt per Email gezeigt, hat mich jemand angeskypt, liegt es im Sharepoint, im Teams?	
Interview 33	Bei IBM Connections? Das ist manchmal tatsächlich nervig und schwierig. Ich persönlich auch an manchen Stellen, ich würde es nicht als altmodisch bezeichnen, aber ein klassisches, in angesicht zu angesicht meeting hat schon mehr geholfen, als acht email hin und her zu schreiben.	
Interview 33	Da ist man nur sauer, weil der andere nicht versteht, was ich möchte. Hat aber nicht zwanghaft was am datenbank arbeiten zu tun. Ja, ich glaube der Punkt ist, man muss versuchen sich zu fokussieren, dass wenn man eine gute Datenbank hat, dass die Leute dann, sage ich mal, ausshchliesslich über diese Datenbanken dann arbeiten und nicht mehr alle anderen Möglichkeiten auch nutzen.	

Interview 33		Und was macht dann für Sie eine Datenbank benutzerfreundlich oder gerade nicht?
Interview 33	Ja, das ist jetzt glaube ich wie ein sozial Network. Je mehr Leute die gleiche Datenbank nehmen, desto attraktiver macht die sich. Und wenn ich dann weiß, die Leute nutzen sie nicht, dann ist auch bei mir die Bereitschaft nicht so gro´ß, die auch zu nutzen.	
Interview 33	Ich glaube das ist auch so ein bisschen ein Punkt: Sie müssenso eine kritische Masse generieren, die diese Datenbanken tapfer benutzen und dann klappt die von ganz alleine und dann wird es auch immer mehr.	
Interview 33	Wenn sie da aber irgendwo eine Stiefmütterlich behandelte Datenbank haben, die irgendiwe einmal im Jahr wird die abgedated und dann werden die neuen Business Cases hochgeladen, das funktioniert nicht. Das Ding muss eigentlich wenn ich es aufmache, den aktuellen Stand wiederspiegeln, weil sonst kann ich mir nie sicher sein, ob die Daten, die da drin sind, überhaupt noch OK sind.	
Interview 33	Ich glaube, das ist ein wichtiger Punkt, wenn es eine Datenbank gibt, dann muss sie up to date sein, sie muss von möglichst vielen genutzt werden, ansonsten funktioniert sie nicht.	
Interview 33		So meine letzte Frage für heute ist: welche Möglichkeiten sehen sie für zukünftige Verbesserungen vor allem auch durch die technologische Entwicklung wie künstliche Intelligenz?
Interview 33	Oh, das ist eine spannende, sehr spannende Frage. Ich glaube, im Bereich der Datenauswertung und Experimten ist Künstliche Intelligenz, kann was sein. Ich muss ihnen aber gestehen, ich glaube nicht dran, weil sie müssen sie erstmal füttern, damit sie selber denken kann. Und das ist ein sehr aufwendiger Prozess.	
Interview 33	Das heißt, ich glaube bis ich für mein normales Projekt hier alle Versuche druchgemacht habe, damit die künstliche Intelligenz quasi jetzt weiß, was sie in Zukunft machen soll, bin ich mit meinem Projekt fertig. Also quasi da, wo sie unglaublich viele Daten generieren, da glaube ich ist künstliche Intelligenz was sinnhaftes, auch weil sie das internet um irgendwelche Trends zu finden.	
Interview 33	Ich glaube für sowas können Sie künstliche Intelligenz einsetzen. Für das Management, das konkrete Management eines Porjektes, glaube ich ist Artificial intelligenz, zumindest in der Ausprägung, über die wir uns momentan unterhalten, nicht geeignet.	
Interview 33		Haben Sie sonst noch irgendwelche Barrieren oder Treiber oder irgendwas, was Sie mir mitgeben wollen zu dem Thema?
Interview 33	Also geredet habe ich viel.	
Interview 33		Ja, es war echt ein sehr schöner Einblick, danke
Interview 33	Ja, ich überlege gerade tatsächlich. Also ich mein, es gibt, wenn man wahrscheinlich noch länger ein bisschen länger nachdenkt, noch diverste Datenbanken, die wir nutzen.	

Interview 33		Es sind wirklich unglaublich viel. Ne, ich glaube, das wichtigste habe ich gesagt von wegen: es muss halt angenommen werden, wenn die Leute nicht mitarbeiten, dann hilft Ihnen die schönste Datenbank nichts, genau. Ich glaube damit lassen wir es.	
Interview 34	Time	Interviewee 34	Interviewer
Interview 34	01:00		Meine Einstiegsfrage ist: Was gehört so zu deinen täglichen Aufgaben (...)?
Interview 34	01:05	Ich bin jetzt seit März hier Laborleiter und da habe ich mehr oder weniger zwei Grundbereiche. Einerseits betreue ich natürlich wissenschaftliche Fragestellunge. Das heißt, ich gucke mir komplett neue Foschrungsfelder, aalso auch für (...) komplett neue Forschungsfelder an und gucke, ob die was für uns sind oder nicht. Das ganze nennt sich scientific pioneering. Und ist in dem gesamtkonzept relativ neu, also dass man einfach mal exotische Technologien sich angucket und da die machbarkeit für die Kosmetik abschätzt. Und auf der adneren seite bin ich antprlich auch Gruppenleiter. Das heißtl, ich habe Leute unter mir, die ich auf unterscheidliche Art und weisen anleiten. Und hier probieren wir gerade auch ja neuartige Methoden der Zusammenarbeit. Diese New Wok geschichte, die hast du ja hier mitbekommen. Das was da diskutiert wurde, das leben wir schon eine ganze Zeit.	
Interview 34	02:11		Und in welchen Data Sharing Aktivitäten bist du denn involviert?
Interview 34	02:18	Momentan Data Sharing - ich meine, meine direkte Teamkollegin (Name) macht die ganzen Claim Reoprts hier. Ich meine, das ist ein sehr offensives Data Sharing. Ich meine , da wird all das , was hier an wissenschaftlichen Daten produziert wurde - auf jeden Fall auf Claim Seite wird in Reports umgeschrieben. Mit denne wir dann auch vor Gericht uns verteidigen müssen oder können. Also das ist ja schon sehr offenes Teilen von Daten. Die ganze (-) Institution mehr oder weniger. Was sonst Data Sharing angeht - ich habe vorher relativ viel in die Richtung gearbeitet. Da war ich unten im Active Scouting bereich. Und da war es für uns halt wichtig, dass die Informationen zu wirkstoffen halt auf eine vernünftige Art und weise abgelegt wird. Und da wird beispielsweise zu dem Zeitpunkt auch schon rausgefunden haben, dass die Ablage methoden, die wir da verwendet haben, relativ veraltet waren oder auch sehr heterogen, haben wir jetzt primär, primär ahmed - den du villeicht auch kennst - ahmed kaamal, ja auch den Ersatz des klassischen Chemfinders durch neue MEthoden angelegt. Um halt viel einheitlicher ein Teil der Ablegen von Daten zu ermöglichen.	

Interview 34	03:46	Hier ist dann beispielsweise wichtig, dass (-) halt die art und weise wie das abgelegt wird, irgendwie genormt ist, damit man die daten dann auch im endeffekt vernünftigt auswerten kann. Weil Natürlich ist das schön, wenn irgendein Labor das irgendwie bewertet und dann irgendwelche Kreuze, sternchen und sonst irgendwas da reinpackt. Viel wertvoller für die Firma ist das dann aber,, wenn das dann wirklich, wenn das im großen stil und für jeden verständlich ablegt wird. Und man dann beisoielsweise, wenn man über die Daten jahre akkumuliert, dass an da auch noch was herausziehen kann. Und da würde dann der AI ansatz fundiert auch endlich mal sinn machen. Momentan ist das so, klar man kann AI machen, aber dazu müssen die Datenaufbereitung halt - super langer weg. WEil du halt wie gesagt ganz unterschiedliche Formate, DImensionen usw hier mit einbeziehen musst. Das wollten wir vereinfachen.	
Interview 34	04:42		Und inwieweit werden dann Daten von anderen im Arbeitsalltag verwendet?
Interview 34	04:50	In meinem Fall, ich meine da wir alle auf Funktionen wie Chemfinder zurückgreifen, nutzen wir natürlich die ganze Zeit die Daten anderer leute. Wenn wir beispielsweise uns die ersten invitro Ergebnisse für irgendwelche Wirkstoffe, bei uns sind es primär durch (...) schon evaluierte und auch als sicher bewertet wirkstoffe, aber wir müssen uns da die Daten angucken, dann müssen die auf eine entsprechende Art und Weise abgelegt sein - sind sie normalerweise auch. Das sind die Daten, die wir so im täglichen Alltag so abgreifen, genau.	
Interview 34	05:29		Und auf welche Daten würdest du gerne noch zusätzlich zugreifen?
Interview 34	05:33	Also ich habe eigentlich die Luxussituation, dass ich auf alle daten, die für mich interessant sind, soweit zugreifen kann. Also das ist jetzt nicht irgendwie so, dass mir irgendetwas fehlt. Ich habe Zugriffsrechte durch unterschiedlichste Sachen, weil ich habe vorher bei der Wirkstoffgruppe gearbeitet habe, dass ich da sehen kann. was da gemacht wurde. Was natprlich insgesagmt schöner wöre, wenn die Labore generell etwas offener ablegen würde. Das geht jetzt nicht nur in die richtung von numerischen Daten ,als oZahle, Molekülargewichte oder sonst irgendwas, sondern beispielsweise auch in Richtung eines offenen Labornotebooks. Das man einfach sehen kannn, ok die Leute haben einen bestimmten - wirkstoff oder an einer bestimmten Fragestellung gearbeitet und ich kann wirklich die Überarbeitung einsehen, ich kann die Ergebnisse sehen, ich kann auch sehen ,was die Leute gemacht haben oder vieleicht auch nicht gemacsht haben. Dabei geht es ja nicht darum zu bewerten, ob leute irgendetwas falsch machen oder irgendwie zur Selbstbereichung zum Vorteil Daten abzugreifen sondern vielleicht aus Fehlern lernen oder aus dem gelernten anderer lernen, genau.	
Interview 34	06:55		Welche Anforderungen stellst du denn an die geteilten Daten?

Interview 34	07:00	Wenn die Daten geteilt werden, dann muss die Datenqualität hoch sein. Wenn wir über numerische Daten reden, beispielsweise bio assays, in biologischen Laboren, dann erwarte ich Messungen und Replikate mindestens - also wenn es um so in vitro versuche geht. Technische Triplikate oder biologische triplikate idealerweise um wirklich sicher sagen zu können, und dann in dem Fall auch nicht nur eine statistik, die dann da mit drei Sternchen dokumentiert, dass da irgendein effekt ist, denn man sich da fast reindenken muss, also niur ein statistischer Effekt, sondern im idealfall auch etwas größere Effekte, also echte effekte, die dann dokumentiert werden, damit die Daten / es sollen einfach valide Daten sein, also nicht einfach nur irgendwas, sondern die solen über unterschiedliche Methoden verifiziert werden und dann abgelegt werden.	
Interview 34	08:00		Gibt es noch weitere Anforderungen an Daten?
Interview 34	08:02	ne, da bin ich eigentlich sehr neutral. Ich mein, das was der assay ab, wirft der assay ab. Die muss man einfach vernünftig aufarbeiten. Eine Interpretation dazu wäre natürlich immer ganz schön. Aber Rohdaten sind natürlich erstmal, nicht Rohdaten, sondern soweit prozessierte Daten, das man damit arbeiten kann ohne dass sie verwässert sind.	
Interview 34	080:30		Nun hast du ja schon erwähnt, du hast die Luxussituation, dass du auf alles zugreifen kannst, was du so brauhcst. Wie kommst du denn momentan an die Daten ran?
Interview 34	08:38	Also momentan gibt es mehrere Quellen. Manche Aspekte wie die unser Chemfinder, die lagen auf separaten Laufwerken. Da hat man dann zugriff über ein Programm oder über Programme hat man Zugriff auf die Datenbanken. Dann haben wir unsere klassischen Systeme, die alle über ZEUS angesteuert werden können. Das ist so der klassische weg. DAs ändert sich ja jetzt, das sollen neue Methoden, neue Datenbanken hinterlegt werden, die das ganze vereinfachen und uniformisieren sollen. Also dass weniger Datenbanken ganz einfach nötig sind um alle Daten abgreifen zu können. Es kommt immer auf die Fragestellung drauf an. Wenn es Daten in Richtung - es müssen ja nicht biologische Daten sein oder chemische - es kann auch in Richtung Vertragsdaten gehen. es kann in Richtung PAtent, IP Daten gehen. Da hast du halt nterschiedlichste Plattformen für. Das, wenn du hier über Verträge nachdenkst, hast du eine Plattform, die wir direkt ansteuern können mit SPider, einem Programm, das intern genutzt wird. Ja, es gibt für jede fragestelung mehr oder weniger ein spezielles Tool.	
Interview 34	09:55		Und wie stellst du dir dann den idealen Zugriff in Zukunft vor?

Interview 34	10:00	Also ich finde, für sehr spezielle Fragestellungen ist es immer noch sinnvoll eine Software zu haben, die darauf zu geschnitten ist. (-) Da kann es keine uniforme Antwort geben. Du willst keine Vertragsdaten in einem eher chemie oder raw material basierten Tool sehen. Also das macht keinen Sinn. Was aber- und das ist auch gerade schon in arbei - was sinnvoll ist, dass alle Daten, die irgendwie mit raw material, mit wirkstoffen zu tun haben und auch mit den Ergebnissen zu tun - das sollte schon im idealfall alles zusammen fließen. Also dass man dann auch auf einem Blick sagen kann: ok, das ist der Wirkstoff, so wsieht er aus, so wirkt er aus, da können wir ihn kaufen, das kostet er, und dann ist er bioabbaubar, die ganzen Faktoren, die dann auch für uns wichtig sind. Das sollte eigentlich im idealfall in so einem Tool alles hinterlegt sein oder hinterlegbar sein. Ob ich das immer alles direkt brauchee, ist natürlich die Frage, weil es kommt imer auf den Status des Projektes an ob ich das immer alles anfragen muss in dem Fall. Aber der ideale Zustand wäre wirklich zu einem Molekül hast du alle Infroamtionen wirklich parat und musst jetzt nicht über (-) über Umwege zu Informationen kommen. Weil was was heutzutage immer noch ist, ist dass ein Wrikstoff, den du dir anguckst, und du gehst in den Chemfinder, findest dort einen kleinen Satz der Ergebnisse dazu, dann findest du einen anderen Satz der Ergebnisse dazu über Nero oder Business flow, und den Rest der Informationen, der eigentlich wirklich interessant ist, findest du eventuell über die Suche im damals BluePlanet R&D search und sonst irgendwas. DAs gibt es so nicht. So kann man nicht sinnvoll sich irgendwie ein Bild über die Historie von einem Wirkstoff einer Firma machen. Es wäre schöner, wenn das irgendwie konzentriert in einem Tool vorleigen würde, in dem man sagt: das ist das Molekül, ja kennen wir, das haben wir damit gemacht usw.	
Interview 34	12:20		Welche Probleme und Schwierigkeiten treten denn auf beim Verwenden Daten anderer?
Interview 34	12:23	Was oft ist, oft stört mich die Datenqualität, eindeutig. Bei vielen Bioassays ist mir die Qualität der produzierten Daten nicht hoch genug. Es kommt, es sind unterschiedlichste Faktoren. Wie gesagt, Datenqualität ist ein Faktor, Komplettheit der Daten, wie oft wurden ein Assay beispielsweise, wie wurde der Assay gemacht, womit wurde der Assa gemacht usw. Solche Sachen.	
Interview 34	13:05		Das sind dann Probleme in welchem Sinne?
Interview 34	13:10	Es geht da mehr oder weniger um die Datenqualität. Biologische Datenqualität. Das ist halt. Man kann es nicht immer bewerten. Das ist ein bisschen - man muss sich darauf verlassen, dass die anderen das gut machen. Aer oft bin ich - da geht es generell darm, dass es oft halt, die / man sollte die Wissenschaftlichkeit etwas höher legen lassen. Genau.	
Interview 34	13:30		Und welche Probleme und Barrieren treten dann auf, wenn es um das Thema Datensharing geht?

Interview 34	13:42	Was ein klassisches Problem bei unterschiedlichen Forschungsgruppen ist, ist die Tatsache, dass Forschungsgruppe relativ ungerne zu einem frühen Zeitpunkt - oder generell - ihre Daten rausgeben. Das kann daran liegen, dass die einfach wissen, dass die Qualität nicht gut genug ist, obwohl das auch wichtig. ALso beispieslweise die Tatsache, dass irgendetwas nicht funktioniert, ist ja eine wichtige erkenntnis. Und das wird oft nicht kommuniziert. Entweder weil die Plattform nicht da ist oder weil die Plattform für einen Teil der Daten nicht genutzt wird. Weißt du wo ich hin will? DAs sind so die Hauptprobleme. Also Daten werden ganz einfach häufig abgelegt, dass sie für alle abgegriffen werden können. Und die qualität der daten ist halt auch immer wieder (unv)	
Interview 34	14:44		Gibt es deiner Meinung nach auch finanzielle Aspekte, die das Data shraing beeinflussen?
Interview 34	14:55	Finanziele Aspekte? Sollte so nicht sein, sehe ich nicht.	
Interview 34	15:01		Rechtliche Aspekte?
Interview 34	15:04	Bei Daten, die innerhalb einer Firma produziert werden, nein.	
Interview 34	15:06		Und nach außen?
Interview 34	15:10	Ja nacah außen, dass ist natürlich der klassische Faktor Daten teilen. Ich muss natürlich IP technisch abgeklärt sein, aber ich denke, wir haben da ein relativ klares Regelwerk von Verträgen, die wir normalerweise aufsetzten mit externen. Die dann auch einen austausch mit Daten ermöglicht. Und auch relativ klar geregelt.	
Interview 34	15:35		Und wie ist das dann mit Aufwänden, die dazu getrieben werden Daten zu teilen? Ist das ein Problem?
Interview 34	15:50	Wie meinst du die Frage?	
Interview 34	15:52		Das es einfach zeitaufwendig oder personalaufwendig ist, die Daten dann für andere Bereit zu stellen.
Interview 34	16:04	Eigentlich ist es nicht zeitaufwendig. Natürlich, jeder Workflow, der neu etabliert wird, muss einmal gelernt werden, aber wenn der gelernt ist, dann sind normalerweise abalgeprozesse kein Problem. Ich meine, wenn du beispielsweise an ein elektronisches Labnotebook denkst. Du schreibst da ja sowieso rein. Und wenn du das einfach mit allen teilen, ist das ja in Ordnung. dann kann da jeder reingucken. Ob man da natürlich - da gibt es ja auch verschiedene Formate. Ob man da jetzt immer die abgeschlossen Projekte drin haben muss, wo dann am Ende dann auch eine Unterschrift drunter muss: ja ich habe dieses Experiment beendet und sonst irgendwas. Das stelle ich in Frage. Das finde ich etwas übertrieben. Das funktioniert vielleicht in der Hardcore produzierenden Industrie - also nicht wir in der Forschung. Aber sonst, sollte das kein Problem sein, daa einfach offene Notebooks zu haben. Wo man einfach zu einem bestimmten Zeitpunkt sagt, ok, ich habe die zu einem bestimmten Zeitpunkt abgeshclossen, ich kann die jetzt freischalten, das sollte schon gehen.	

Interview 34	17:20		Apropos freischalten. Welche Daten werden dann von (…) geteilt - oder von deiner Seite?
Interview 34	17:25	Das kommt auf das Projekt, das kannst du nicht pauschal sagen. Das können (-) also normalerweise teilen wir primär irgendwelche experimentellen Daten. Wenn jetzt hier ein klassisches Projekt hast, dann bekommst du normalerweise eine Technologie von außen und evaluierst die und dannteilst du diese Daten mit deinem Hersteller oder deinem Kooperationspartner um dann zu entschieden, ok kann das was für uns können wir das weiter treiben, oder warum klappt das ganze auch nicht, was stört uns, was können wir besser machen. Genau.	
Interview 34	18:05		Und soll der Austausch oder das Datensharing mit anderen ausgebaut werden in Zukunft?
Interview 34	18:15	Ich glaube nicht, dass es da wirklich strategien oder vorhaben gibt umd da irgendwie das Data sharing nach außen zu erhöhen. Bei uns ist das sehr projektabhängig. Also. Wenn wir hier jetzt hier unsere kleinen Projekte haben, rausgeben, ist das eine ganz andere Nummer, wenn man sich andere Aspekte angucken würde, wir geben jetzt alle Formel Daten nach außen um die mit einer AI analysieren zu lassen. Das sind projekte, die hier laufen um da zu lernen, ok, wenn wir diese Formelbestnadteile zusammen werfen, dann bekommen wir eine viel bessere Formel, die wir vorher hatten. DA wären wir nie drauf gekommen. Da hat uns zum Beispiel eine Künstliche Intelligenz drauf gebracht. dAAs ist so ein Projekt, das hier läuft. WIe gesagt, das kann man so generell nicht benatworten, genau, nicht generalisiert beantworten.	
Interview 34	19:18		Welche Beweggründe stehen denn dahinter, Daten mit anderen zu teilen?
Interview 34	19:20	Also im Prinzip, Datenaustausch kann vertraglich geregelt sein, dass dieser Datenautausch stattfindet. Und Datenaustausch muss natürlich stattfinden, wenn man sich einem Problem, also einem wissenschasftlich Problem nähert. Der erste Datenaustuasch ist ja schon, ok wir haben hier ein Problem und das ist unser genaues Problem. Dait gibt man ja Daten preis, Informationen preis. Das Gegenüber muss dann auch Daten rüber geben, wie dieses Problem eventuell gelöst werden kann. Ich glaube, das ist ein bisschen an der Frage vorbei.	
Interview 34	20:00		Welche Beweggründe gibt es Daten zu teilen?
Interview 34	20:05	Zusammen voran schreiten in Projeke, das ist glaube ich die einfache antwort.	
Interview 34	20:10		Gibt es noch weitere Gründe dafür?

Interview 34	20:30	Wenn ich jetzt in der Alademie sitzen würde, im Prinzip sollte Wissen geteilt, da es natprlich dem Allgemein wohl dient und diese Daten von anderen genutzt werden können um neue Erfindungen zu machen. Das kann man jetzt generell für die Industrie nicht so sagen, weil wir wolen natürlich unser Wissen schüten. Aber im Mikrokosmos (...) sollte es genau so sein, dass man asgt, ok, wir teilen die daten, damit andere damit arbeiten. Damit neue Erknenntise auf basis dieser Daten gemacht werden können. Weil sonst ist das Geld, was wir für die Generation für Daten heraufbewendet hat., im Prinzip verschwendet. Da hat man eine Wuste an Informationen geparkt, keiner hat Zugriff drauf und keiner kann es gebrauchen.	
Interview 34	21:20		Wie steht es denn um technische Apskete? Nun hast du ja schon gesagt, es gibt ganz viele unterschiedliche Plattformen. Haben denn jetzt auch technische Aspekte darauf, wie Daten geteilt werden?
Interview 34	21:30	Natürlich, wenn das Tool schlecht ist, wird es weniger genutzt und dementsprechend werden weniger Daten geteilt. Das kann aber nur subjektiv sein. Teilweise ist es auch nur die Tatsache, dass Leute es vorher nicht gemacaht haben, sich erstmal an einen neuen Workflow gewöhnen müssen, daten auf vernünftige Art und weise zu teilen Genau, du musst diese Aktivierungsenergie so ein bisschen überwinden. Aber je schlechter das Tool, desto höher die Schwelle, soetwas zu nutzen.	
Interview 34	32:08:00		Wird denn das Teilen von Daten auch anerkannt?
Interview 34	22:14	Von mir schon. Also ich finde (--) ja von vielen wird eigentlich, genau, die Tatsache sehr, sehr positiv aufgenommen, wenn man Daten teilt. Im Prinzip ist ja auch die Präsentation der Daten intern ist ja auch ein Teilen von Daten. Das wird normalerweise sehr positiv aufgenommen, weil es führt zu Diskussion, richtig.	
Interview 34	22:43		Und wenn du jetzt an die Unternehmeskultur hier denkst, glaubst du dass hat auch einen Einfluss darauf, wie man mit dem Datenteilen umgeht?
Interview 34	22:53	Eindeutig, ich meine, wenn du eine sehr offene und kommunikative Unternehmenskultur hast, wird auch das Datenteilen ganz einfach offener sein. Aber wenn man porjektteams von vornherein so aufstellt., dass man sagt, hier mach mal und behalts am besten für dich und (unv) in Konkurrenz setzt, dann wiwrd natürlich auch keine teilende Kultur daraus entstehen.	
Interview 34	23:25		Jetzt mal eine ganz andere Frage. Ichhabe ja am Anfang erzählt, dass ich eine Vorlesung zum Thema AI und Big-Data gehört habe. Wie schätzt du denn den Einfluss der Technologien auf den Forschungsalltag ein?

Interview 34	23:40	Technologien können den Forschungsalltag vereinfachen. Technologien können aber auch einfach nur einen riesigen Wust von Daten produzieren, die keiner versteht. Die Tatsache, dass eine Technologie da ist und auch dass wir uns die Leisten, bdedeutet nicht, dass wir da mit gut Daten produzieren. Es ist immer noch so, am Anfang von jeder Forschungs, von jedem Forschungsprogramm sollte eine Hypothese da sein. Wenn man dann Daten produziert muss man damit seine Hypothese verifizieren oder falsiz´fizieren können. Wenn man das nicht kann, sind die Daten nichts wert. Also: -technologie generell gut, aber sie muss richtig eingesetzt werden. Und dann zum Thema AI, AI ist für mich gerade ein Buzzword. Es gibt ein AI für alles - oder eine AI für alles. Aber AI kann auch in ganz einfachen Satz beschrieben werden, das ist nichts anderes als Machine Learning. Bulshit in - Bullshit out. Wenn dann Datenqualität müll ist, wird auch die Antwort an das System müll sein. Also generell, ich bin nicht gegen Technologien, aber sie müssen richtig benutzt sein. Und Formeln in Richtung in-Silico Technologien denkt, deswegen gucke ich gerade zum Computer rüber - ein Rechner it abhängig schon wieder von der Qualität deier Daten Ein rechner kann nur vorhersagen machen, auf basis von dem, was er vom eingegeben lernt. Merh kann er nicht, er kann selbst nicht ein Problem lösen. Genau.	
Interview 34	25:25:00	Deswegen muss man das jetzt etwas differenziert sehen, was ist jetzt eine Technologie - ai ist eine Art von Technologie, eine in.silico Technologie. Es gibt jetzt auch sehr viele Technologien im Labo alltag, die uns natprlich das Leben einfacher machen. Also der Fortschritt der Tehcnologien ist wichtig, damit wir prozesse, die vor vielleicht langer zeit gebraucgt habne, in kürzer Zeit machen können. Wir sollten dabei aber nie vergessen oder verlieren, as wir auch verstehen, was darin passsiert. Und was dann auch die Antwort der Technologie dann wirklich. ALso ich sage das Leuten immer. Man kann jetzt hier im LAbor (-) man sollte imme rverstehen, was der Kit eigentlich machen. Es soll nicht nur eine Black box sein, sondern man muss diese Blackbox verstehen, weil dann kann man das Ergebnis auch ganz anders interpretieren.	
Interview 34	26:30:00		Und der potentielle Einfluss der Technologien, wie schätzt du den ein?

Interview 34	6:36:00	Technologie wird uns immer beeinflussen. Vor allem je mächtiger sie ist, desto mehr wird sie uns natürlich beeinflussen. Man muss natürlich neuartige und multitasking technologien nutzen um weiterhin compettetive zu sein. Wenn ein Competitor die Technologie nutzen kann, damit neues Wissen oder bessere Produkte oder waas auch immer zu generieren, dann haben die natürlich einen Vorteil. Dann wollen wir den Vorteil auch haben oder nicht verlieren. Also riesiger Einfluss der Technologien auf alles, was wir machen. Sollte da sich auch nicht sperren. Ich sage mir mal, dass ma nda so ein bisschen, man sollte vorsichtig mit dem den Technologien umgehen. man sollte sie ansehen, man sollte sie bewerten, aber jetzt nicht direkt in den Himmel erheben. Vor allem nicht Nutzen der Technologien der Technologien willen. ALso das ist - das habe ih letztens bei mir auch wieder abgestellt. Da geht es um simple sachen. Die tatsache, dass ich tools wie planner, teams und ein Telefon habe, heißt nicht, dass ich das haben muss. WEil ich habe nichts daavon, wenn ich die ganze zeit checke, was da passiert und dadurch eventuelle lebenswualität verloren geht, weil ich die ganze zeit darauf gucke. DAs heißt ich stresse mich die ganze zeit mit arbeit, obwohl das nicht nötig ist. genau. genau.	
Interview 34	8:18:00	Die Tatsache, dass an ein handy hat, heißt auch nicht, dass man die ganze zeit erreichbar sein muss.	
Interview 34	8:30:00		Vielen Dank für deine Eindrücke. Ich gucke mal kurz, welche Fragen noch übrig geblieben sind.
Interview 34			Da haben wir schon echt einige geschafft.
Interview 34	28:52:00		Achja, genau. Nun hast du ja schon einige Probleme und Barrieren angesprochen, die beim Data sharing auftreten können. Welches Probelm glaubst du denn ist das größte, wenn es darum geht Daten zu teilen.
Interview 34	29:11:00	Es kommt drauf an mit wem. Also nach extern natürlich IP. Das muss klar geregelt sein. Intern ist es die Kultur. Ist da der Wille, die Daten zu teilen? J und dann auch die Leichtigkeit die Daten zu teilen. Genau, das sind glaube ich die Punkte.	
Interview 34	29:40:00		Inwieweit hat dann das Management darauf einfluss?
Interview 34	29:45:00	seufz. Das Management bestimmt, ja. Du wirst da nur halt die klaren Entscheidung ganz einfach bekommen. DasManagement hat aber auch auf der Seite enfluss, dass sie einfach den Anwendern, den Personen, die halt die Daten teilen sollen (--) fange ich nochmal andersrum an. Im Prinzip, bestimmt das MAnagement natprlich, dass bestimmte Daten auf eine bestimmte Art geteilt werden sollen oder nicht. Sie können es aber auch negativ beeinflussen. Es gibt halt auch Menschen, die beispielsweise ein nicht-teilen befürworten und damit einen negativen Einfluss auf die ganze Kultur haben. Managmeent in der doch noch hierahcisch organisation hat natürlich einen weiten Einfluss. Positiv oder negativ - das sind die Etnscheidungsgeber, die sind noch impilsgeber, genau.	

Interview 34	29:50:00		Hast du denn sonst noch irgendetwas weiteres, was du mir zum Thema Data Sharing mit auf den Weg geben möchtest?
Interview 34	29:52:00	Aso ich finde, Data Sharing ist eigentlich, das Fundament eines Wissenschlich arbeitenden oder forschenden Unternehmens, weil nur geteilte Daten können halt als Fundament für neue Studie mehr oder weniger herhalten. Und keiner hier wird ewig auf seiner Position sein. Das ist jetzt nicht negativ, das heit nicht, dass die Leute hier rausfliegen oder sonst irgendwas. SOndern Leute entwicklen sich weiter und orientieren sich um, werden befördert, werden - in Rente - und sow weiter. Deswegen müssen die Daten, die produzierrt werden, auch vernünftig und nachvollziebare Art und weise abgelegt werden, damit sie für alle nutzbar vorliegen. Und auch, für alle verstehbar vorliegen. Genau. Das ist wirklich, für ich ist es eindeutig das Fundament, das ist das, was man immer wieder unterstreichen sollte, weil - es mehrere Gründe da. Es sind die Kronjuwelen, die sich ein Unternehmen mehr oder weniger generiert. Das sollte von allen auch verstanden werden.	
Interview 34	32:16:00		Ja, vielen Dank. Hast du sonst noch etwas weiteres?
Interview 34	32:18:00	Ne, das wäre es glaube ich	
Interview 35	Time	Interviewee 35	Interviewer
Interview 35	00:18		Meine erste ist: Was gehört hier am Institut zu Ihren täglichen Aufgaben?
Interview 35		Ich bin Direktor am Institut und ich leite das Institut und (-) leite mehrere große Forschungsverbünde, (Ort) Life Sciences, ein Sonderforschungsbereich, (-) DFG-Projekte. Das ist meine tägliche Aufgabe.	
Interview 35			Und in welchen Data Sharing Aktivitäten sind Sie involviert?
Interview 35		Wir produzieren große Mengen an molekularen Daten, sequenzierdaten, genomische Daten vom, von vielzahl von Organismen einschließlich des Menschens und versuchen mit diesen Daten zu arbeiten - oder arbeiten mit diesen Daten und (-) tauschen diese Daten natürlich auch aus. Und das ist nicht nur am Institut sondern das geht eigentlich weltweit.	
Interview 35			Und worüber tauschen Sie Daten dann aus?
Interview 35		Wie meinen Sie? Mit welchem Medium?	
Interview 35			Genau.
Interview 35	01:11	Das sind / Wir haben Datenbanken. Diese Datenbanken haben ein bestimmtes Profil muss eben, muss man dann entsprechend bedienen. Und dann kann mein Kollege oder meine Kollegin ebenfalls auf diese Daten zurückgreifen. Das können Sie zum Teil auch mit unseren biomedizinisch arbetieten Patienten natürlich anonymisiert. Das sind zum Teil Patientendaten, die wir dann da vergleichen. Also so tauschen wir uns aus. Der Zugang; der codierte und begrenzte und geschütze Zugang zu Datenbanken - das wäre dann vielleicht die Antwort.	
Interview 35			Inwieweit verwenden Sie denn Daten von anderen bei Ihrer Forschung?

Interview 35		Ganz viel. Wir haben ganz viel dieser Art von quantitativen Daten zu molekularen Prozessen, zu evolutionären Prozessen (piept kurz im Hintergrund) werden natürlich (-) nicht nur von uns sondern auch von anderen Arbeitsgruppen erzeugt und dann ist es eben guter Stil und Pflicht in der Wissenschaft solche Daten publiziert. Das sie dann auch zugänglich sind für jedermann. Und so greifen wir zur Zeit zum Beispiel sehr intensiv auf ein Datensatz zu von der Gruppe aus Californien, die dann eben auch diese Daten auf eine Datenbank legt, die haben die erzeugt aber wir haben Zugang dazu und wir benutzen diese Daten und vergleichen Sie mit unseren Daten und ziehen aus diesem Vergleich dann Schlussfolgerungen wie bestimmte Strukturen zum Beispiel sich in der Stammesgeschichte evaluiert haben.	
Interview 35	02:47		Auf welche Daten würden Sie denn gerne noch zusätzlich zugreifen?
Interview 35		Alle Daten, die ich haben möchte, kann ich eigentlich / habe ich Zugang dazu. Also andere Daten erzeugen wir dann selber. Es gibt zur Zeit / Auf die Frage kann ich nur antworten (-) wir haben zur Zeit kein Bedürfnis, das nicht erfüllt werden kann.	
Interview 35			Und welche Anforderungen stellen Sie denn an die geteilten Daten von anderen?
Interview 35		Das sie in einer Sprache geschrieben sind und das sie in einem Format sind, sodass wir tatsächlich Zugang haben. Das ist auch das große Problem in der Daten - jetzt werde ich so langsam warm und weiß, was sie eigentlich wollen - ein großes Problem im Data Sharing ist natürlich, dass man die gleiche Sprache spricht.	
Interview 35		Also das macht kein Sinn, wenn eine Arbeitsgruppe etwas erzeugt, was dann niemand anderes lesen kann und abgreifen kann. Aus diesem sind wir dabei, auch professionell, mit Mitarbeiterinnen und Mitarbeitern, dass man Daten (-) dass wir einen systematischen Zugang dazu hat und auch schon bei der Erzeugung der Daten dafür sorgt, dass das in einer Art und weise gemacht wird, sodass eben auch andere dabei Zugang haben. Große Publikationsorgane, führende Journale, haben dazu auch bestimmte Formate, wie diese Daten eingepflegt werden müssen, damit eben dann die ganze wissenschaftliche Community darauf Zugang hat.	
Interview 35	04:18		Und die bestimmen dann die Anforderungen?
Interview 35		Das Format ist vorgegeben. Wenn Sie bei PMES, bei Proceeding National Academy, sich ein Paper publizieren, dann MÜSSEN Sie ihre Daten in DEREN Maske einpflegen. Und das gilt genauso für viele andere. Das heißt, die Formate sind vorgegeben und damit sind sie dann auch weltweit vergleichbar.	
Interview 35			Um jetzt mal in so ein Zukunftszenario zu gehen: Wie stellen Sie sich denn in Zukunft den optimalen Zugriff auf Daten vor?

Interview 35		Daten sollten möglichst für jeden Mann und jede Frau zugänglich sein. Das heißt, dieses (-) dieses strukturieren und systematische Ablegen von Daten (-) darf eigentlich nicht nur die Spitzenjournale und Spitzengruppen betreffen, was zurzeit passiert, sondern es gibt natürlich ganz viel in der Breite von Arbeitsgruppen, die irgendwelche Daten erzeugen und die dann häufig vergessen sind. Die zum Teil dann eben auch / die werden dann in der Wissenschaft nicht genutzt. Das ist schade. Und man sollte versuchen diese Daten mitzubenutzen. Das gilt auch übrigens in bestimmten Aspekten, das ist jetzt mal bei den Stichwort Citizen Science, es gibt viele Daten, die werden von Layen (-) erhoben. Klassische Be /Bereich ist seit 200 Jahren die Onotologie, die Vogelkunde, die in England zum Teil Professionalisiert ist, aber von Layen gemacht wird. Und die Herausforderung für die Daten, dür die Daten, Data Sharing Community ist, dass eben auch diese Daten in die Formate reinpassen müssen, damit sie gemeinsam genutzt werden können. Das ist die, denke ich / das wäre eine wunderbare Vision, wenn wir alle Daten, die zu bestimmten Fragen erzeugt werden, auch gemeinsam benutzen könnten.	
Interview 35		Das Kaviat dabei ist, dass es zum Teil dann sensible Daten sind - Patienten habe ich schon angesprochen. Es gibt auch andere sensible Daten. Wie mache ich eine Atombombe, das möchte ich wahrscheinlich nicht unbedingt öffentlich machen obwohl ich weiß, wie es geht. Das heißt, da müssen bestimmte Schutz- und Zugangs- / trotz aller Veröffentlichung und gemeinsamer Nutzung müssen bestimmte Schutz- und Zugangsbeschränkungen dabei sein.	
Interview 35			Nun haben Sie ja schon Herausforderungen angesprochen. Welche Probleme und Schwierigkeiten treten denn beim Verwenden von anderen Daten auf?
Interview 35	07:00	Das man nicht versteht, was die Daten sind. Ich erwarte, dass Nutzerinnen und Nutzer von diesen Daten, die auf solche Daten zurückgerifen auch wissen, was diese Daten eigentlich sind. Und es kommt manchmal, aber nicht so häufig zu Misinterpretationen von solchen Daten (5) Wie war die Frage nochmal?	
Interview 35			Welche Probleme und Schwierigkeiten auftreten beim Verwenden der Daten von anderen.
Interview 35		Es kommt zu Misinterpretationen von Daten, das ist glaube ich das / das ist unvermeidbar. Manchmal wird auch, werden auch beim Erstellen von solchen Daten handwerkliche Fehler gemacht - das Algorithmen nicht richtig genutzt werden. Und (-) man dann zu Schlussfolgerungen kommt aufgrund der Daten, die aber auf handwerklichen Fehlern beruhen und die dann nachfolgende Generationen wieder ausbügeln müssen.	
Interview 35		Und (-) möglich, was die Auswahl (unv) der Daten getroffen hat und dann eben nicht nachhaltig und nicht gültig ist.	

Interview 35	08:00		Am Anfang haben Sie schon erwähnt, dass Sie jetzt gerade auf eine Datenbank zugreifen, die von anderen auch erstelt wird. Welche Daten teilen Sie denn mit anderen und worüber dann?
Interview 35		Alle Daten, die wir erzeugen, teile ich, teilen wir dann mit anderen. Das ist Pflicht und (-) es gibt keinen Grund bei uns in der Grundlagenforschung vor dem Berg zu halten - das dürfen wir auch gar nicht. Wir publizieren in hochkarätigen Journalen. Und werden und müssen auch alle Daten, die wir erzeugen, auch mit anderen Teilen und das ist für uns tagtägliche Praxis. Es gibt hier keinen Datensatz, der irgendwo nicht zu einem bestimmten Zeitpunkt dann an die öffentlichkeit kommt. In solche öffentlich Zugänglichen Datenbanken.	
Interview 35			Welche Probleme und Barrieren sehen Sie denn beim Data Sharing allgemein?
Interview 35		Da habe n wir das alles angesprochen. Ich, Probleme. Da muss ich mich jetzt einfach wiederholen. Probleme sind, wenn die Daten nicht in eine, bestimmten Muster und nicht nach einer gemeinsamen Struktur erzeugt sind, dann sind sie eigentlich nicht verwertbar. Das heißt, man muss eine (-) eine Form oder eine Struktur in ein Format schaffen, in dem man von den verschiedenen Quellen der Daten her, die Daten irgendwo wo alle dann vereinheitlicht werden, so vereinheitlicht werden, dass sie dann auch von jeder man gelesen werden können. Das ist die Herausforderung, das ist / dem kann man sich aber sehr einfach stellen heute. Das ist die Hauptschwieirigkeit, die man lernen muss, wenn man mit solchen großen Datenbanken vergleichend umgeht, müssen die für jedermann auch tatsächlich lesbar sein - das ist die Hauptschwierigkeit.	
Interview 35	10:00		Und jetzt nochmal ganz andere Aspekte anzusprechen: Nun ist es nunmal so, dass Daten zu produzieren und auch die Forschung allgemein finanziell aufwendig ist bzw. es kostet auch Geld Forschung zu betreiben. Wie ist es denn bei finanziellen Aspekten? Beeiflussen die irgendwie das Daten Austauschen?
Interview 35		Nein, das tun sie eigentlich nicht. Diese Forschungsgruppe hier, mit der Sie hier reden, ist finanziell sehr gut unterstützt, wir haben ganz viele Drittmittelprojekte in hoher höhe, die uns diese Arbeiten ermöglichen. Das sehe ich auch in meiner Verantwortung, dass wir dann diese Daten dann auch tatsächlich auch allen zugänglich machen. Das Problem sehe ich bei Gruppen, die keine / die nicht solche finanziellen Mittel haben. Da mag es dann schon an die Grenzen gehen. Erstens ich kann solche quantitativen ohne Mittel gar nicht erzeugen und (-) das heißt, da tut man dann gut daran, sich mit Verbündeun zusammen zu tun, um solche großen Datenmengen dann auch wenn man selber eine kleine Arbeitsgruppe ist, das dann zu schaffen.	

Interview 35	11:06		Wie ist es denn mit technischen Aspekte? Haben Sie da alle Lösungen, die sie so benötigen oder gibt es da auch Probleme?
Interview 35		Datentransfer ist immer ein Problem. Datentransfer besonders natüprlich im Internetbereich. Die Wege sind oft zu langsam für solche großen Datenmengen. Das muss ein Datenloser Transfer möglich sein. Da hapert es natürlich noch immer. Aber so hier am Campus (-) und da muss dann kreative Wege finden, aber (-) ich würde sagen, da ist noch Verbesserungsmöglichkeiten da, dass der Datentransfer von solchen riesigen Datenmengen, die man almählich erzeugen kann und erzeugt, tatsächlich auch problemlos vom physischen Ort A nach physischen Ort B kommt. Wir planen mometan ein Projekt mit dem Rechenzentrum in (Ort). Und die Herausforderung wird sein, wie kommen diese Daten nach (Ort) und wie kommen diese Daten nach (OrT). Das sind so die täglichen Probleme.	
Interview 35			Wird denn das Data Sharing in der wissenschaftlichen Community anerkannt?
Interview 35		Natürlich wird das anerkannt, das ist grundvoraussetzung. Wenn ich keine Daten Share, dann bin ich eigentlich ein Outcast, dann bin ich ein Außenseiter und niemand wird mich ernst nehmen.	
Interview 35	12:25		OK. Haben Sie denn noch weitere Apskte, die wichtig sind - die sie mir mit auf dem Weg geben wollen zum Thema Data Sharing?
Interview 35		Ich glaube wir haben alles gesagt, oder?	
Interview 35			Ja, das war auf jeden Fall sehr intensiv.
Interview 35			Meine allerletzte Frage jetzt noch, die ist ein bisschen out of topic. Wie schätzen Sie denn den zukünftigen Einfluss der technologien auf Ihren Forschungsalltag ein?
Interview 35		Sie meinen jetzt konkret Künstliche Intelligenz?	
Interview 35			Oder auch Big Data Analysen.

Interview 35		Das ist zentral. Unsere Techniken werden immer besser und werden immer tiefer. Aus diesem Grund werden auch die Datenmengen immer größer. Und da kommt aber dann ein ganz anderes Schlagwort, was sie auch in den Raum geworfen haben Artificial Intelligence dazu. Und das benutzen wir auch. Wir machen Machine Learning mit unseren Datensätzen. Und zum Teil verstehen wir nicht, was die Daten eigentlich sind. Und dann haben wir einen Kollgen in Los Angeles, dem schicken wir diese Daten. Der hat ein Machinenprogramm, Machine learning programm. Und der kann dann vorhersagen treffen, das könnte das und das sein, die Daten könnten das und das meinen. Und dann können wir hier im LAbor das sozusagen überprüfen. Und das wird, glaube ich, wird in Zukunft immer mehr werden. Das heißt, ich würde das als eine intelligente Verwendung von DAten bezeichnen, die ein intelligentes Ausnutzen von Daten, die häufig auf dem ersten Blick ganz schwierig zu interpretieren sind. Aber wir brauchen dann diese berühmte Künstliche Intelligenz um uns dabei zu helfen. Die wird nicht uns ersetzten beim Nachdenken, sondern sie wird uns helfen bestimmte Strukturen oder Foramte zu verstehen, mit denen wir dann weiterarbeiten können. DAs wird in Zukunft immer mehr zunehmen und wir müssen uns rüsten für die Erzeugung, für die Speicherung und die Weitergabe von solchen Big Data.	
Interview 35			Danke
Interview 35	13:50	Prima	
Interview 36	Time	Interviewee 36	Interviewer
Interview 36			Um sie jetzt nochmal ein bsischen kennenzulernen, würde ich gerne erfarhren, was gehört so zu ihren täglichen Aufgaben - sei es hier oder auch als (Beruf).
Interview 36		Also, hier in der Fraktion bin ich zuständig für drei bis vier Themen. Das eine ist Hochschulpolitik. Das ist nicht so originell, weil ich ja viele Jahre an der Hochschule war. Mit der Hochschule verbunden ist, sind zwei weitere Themen nämlich Berufsfelder neue Arbeit, Arbeit 4.0 zuständig und Industriepolitik. Ich war an der Hochschule Professor für Arbeits- und Orgnaisationspsychologie, also insofern ist das auch relativ naheliegend. Und das dritte Thema ist auch relativ nahe gelegen ist die Digitalisierung. Hat was mit Arbeit 4.0 zu tun und zur Digitalisierung gehört sowas wie Künstliche Intelligenz, das hat wieder was mit Psychologie zu tun. Das sind so Themen, die sehr stark entweder was mit Bildungssystem Schule/Hochschule zu tun haben. Das erklärt sich unter anderem daraus, dass ich acht Jahre Rektor der Uni war in (Ort) und auch Hochschulpolitik gemacht habe, das andere sind inhaltliche Themen.	
Interview 36		Und in sofern wirkt das auch ein bisschen prägend, sind da immer zwei Seelen in meiner Brust. Einerseits bin ich dann doch noch Wissenschaftler und Hochschullehrer und in dieser Funktion sehr stark politisch engagiert und auf der anderen Seite Politiker. Welche Jacke ich jetzt gerade anhabe, weiß ich nicht so genau.	

Interview 36			In welche Data Sharing Aktivitäten / Welche Data Sharing Aktivitäten sind Ihnen denn bekannt?
Interview 36	01:53	Also was ich kenne ist die ganze Open Access Diskussion. Die an den hochschulen geführt wird, sehr weit geführt. Und die für mich auch am deutlichsten das Problem klarmachen, aber auch im politischen Raum merken wir das alltäglich.	
Interview 36		Also wenn Sie einfach mal simple, also wir müssen ja reden halten und ich beauftrage jetzt meine Mitarbeiter: sage mal, ich brauche mal ganz kurz von euch, die Information wie ist das in anderen Bundesländern. Das ist eine irrwitzige Suche und man findet es zum Teil nicht. Ich mache es mal ganz onkret. Morgen haben wir ein Thema, wir haben eine große Anfrage gemacht zzum Arbeitsgesundheitsschutz und ich wollte einfach nur wissen, wie viel geben wir in (Ort) aus und was geben andere bundesländer aus (2) Praktisch nicht zu kriegen. Das heißt, sie müssen extreme recherchieren können, das so ein bisschen versuchen rauszukriegen, aber das es jetzt so ein Portal gibt, wo ich sage, ich gucke einfach bestimmte Dinge nach. Heißt, da müssen wir telefonieren, da kennt man einen , der jemanden kennt, der was weiß. Das heißt, wir haben auch im politischen Raum gar nicht hinreichend öffentliche Informationsquellen.	
Interview 36		Die muss man alle recherchieren. Aber das es jetzt ein Portal wo ich sage mal, da sind die ganzen Daten drin, und die kann man dann auch mit einer Abfrage relativ schnell prüfen. Ich kann natürlich in die Haushalte aller Bundesländer gucken, die sind aber so unterschiedlich aufgebaut. Insofern ist das sehr schwer in der Tat an die Daten ranzukommen. Das eine ist die ganze Open Access Veröffentlichungsdiksussion, das andere ist die Frage, wie komme ich im politischen Raum schnell zu Informationen über andere Bundesländer oder zu Projekten.	
Interview 36			Welchen Einfluss hat denn dann die Politik auf das Data Sharing?
Interview 36	03:40	Naja, sie muss die Rahmenbedingungen verändern. Wir können es ja relativ leicht / das ist dann Hochschulpolitik, das Open Access Dikussion machen. Also wenn sie mal angucken, wie jemand (2) eine Karriere sich entwickelt, dann muss er höhlisch aufpassen, dass seine Daten nicht einfach verloren gehen. Es gab früher sogar eine Regel, wenn sie eine Doktorarbeit gemacht haben, dann hieß es ganz simple, die darf nicht ein zweites Mal veröffentlicht sein vor deiner Arbeit. Das heißt, wenn ich jetzt die Daten rausgebe, jemand nutzt die und veröffentlicht die vor mir, ist meine Promotion quasi den Bach runter, weil sie ist dann ja schon veröffentlicht. Das heißt, da brauchen wir hochschulpolitisch Regeln, wie man damit umgeht.	

Interview 36	04:12	Also, Nachweispflicht, wir müssen wahrscheinlich auch das Urheberrecht ändern. Das heißt, ich habe Daten produiert, gebe sie irgendwo hin, wo ist die Kontrolle, dass sie nicht irgendjemand nutzt, irgendwie nutzt, irgendwie verändert? Das heißt, da glaube ich brauchen wir noch sehr viel mehr Kontrollen, das ist das System von Urheberrecht und auch ein Nachvollzugsurheberrechts. Ich kann tausend mal behauptet, ich habe die Daten / ich habe in (Ort) KiTas untersucht, fünfzig Stück. Das ist vom Datenschutzrecht auch nicht ganz ohne. Ich veröffentliche die jetzt, gebe die in ein Open Access Forum Portal. Jetzt sind die Daten weg	
Interview 36	04:58	Jetzt macht jeder damit, was er möchte. Und ich behaupte sagen wir meine Daten, das könnte ich quasi nicht nachweisen. Das heißt, wir reden über Urheberrecht und auch den Nachvollzug, ist das eine Thema. Wir reden aber auch Datenschutz. Also ich bin ja Psychologe, sozialwisenschaftler. Und wenn sie Untersuchungen machen, in der Regel geben sie ja in der Arbeitspsychologie den Betriben die Zusicherung, dass man die Daten entsprechend schützt und nutz / also veröffentlichen nur wenn der Betrieb oder die Organisation das akzeptiert. Das geben Sie alles aus der Hand. Ich kann ja zig mal einem Betrieb sagen: natürlich benutze ich deine Daten nur für wissenschaftliche Zwecke, wenn ich die aber veröffentliche verlieren ich aber die Kontrolle. Das heißt, meine Zusicherung, die ich persönlich getroffen habe, spielen da kgar keine Rolle mehr.	
Interview 36		Oder ich weiß nicht ob es in ihrem Fachgebiet so ist, ich habe viele Masterarbeiten betreut, wo ich zusichern musste, dass diese Daten nicht veröffentlicht werden. Da sehen sie, wir haben viele Datenschutzprobleme, Datenurheberrechtsprobleme, wir haben Urheberrechtsprobleme, und dadrüber reden wir immer, dass Gesetze gemacht werden müssen, Gesetze verändert werden müssen. Das heitß, die Politik muss die Rahmenbedingungen schaffen.	
Interview 36	06:05	Weil sonst wäre mir das als Wissenschaftler oder auch als Autor zu unsicher.	
Interview 36			Und sind denn Diskussionen darüber in Gange?
Interview 36		Nicht wirklich	
Interview 36			Nicht wirklich

Interview 36	06:22	Also es gibt die Diskussionen an Hochschulen ein bisschen, im politischen Raum gar nicht. Also, es gibt keine wirklich ernstzunehmende Diskussion. Wenn überhaupt ist es eine Datenschutz, Datensicherheitsdikussion. Es ist so ein bisschen so (unv.) von den Grünen thematisiert wird: Open Access ist ganz wichtig ist und ganz gut. Aber das ist eher so eine ideologische diksussion: wir finden das gut. Wer ein bisschen länger - und da bin ich glaube ich einer der einzigen, die bisschen Erfahrung mit sowas haben - sieht halt, dass es nicht so simple ist. Man könnte ja sagen, es sind öfentliche Daten, da muss jeder drüber verfügen. Halt. Also wir haben, da sage ich dann als Psychologe, relativ viele Schutzbedürftige Daten. Wenn ich eine große Untersuchung mache über psychatrische Erkrankungen, dann kann ich die nicht locker in die Welt raus posaunen.	
Interview 36		Und damit spiele ich. Wer hat welche Rechte? Das ist die Schwierigkeit - wer hat welche Rechte an den Daten? Habe ich als Wissenschaftler die Rechte oder was ist dann mit den Menschen, die ich befrage, untersuche oder sonst was. Was ist mit deren Rechten?	
Interview 36	07:33		Wie stellen Sie sich in Zukunft den optimalen Zugriff auf solche Daten vor?
Interview 36		Naja, wir müssen im Prinzip / also ich bin grundsätzich ein Freund davon. Weil es hat- wenn sie die Wissenschaft nehmen - weil es hat einen extremen Reiz zum Beispiel meine eigene Untersuchung an einer anderen Untersuchung zu validieren. Wir haben ein großes Problem in den Wissenschaften, kennen Sie wahrscheinlich auch, nämlich das Reproduktionsproblem. Sie machen einmal eine Untersuchung, kriegen was raus, ob das auf Dauer stabil ist weiß man nicht. Also das heißt, ich untersuche jetzt einen Betrieb und finde, was ich häufiger gemacht habe, dass Zeitdruck meinetwegen zu koronaren Herzerkanken führt. Das scheint sich auch zu bestätigen. Ob das stabil ist, ob das in anderen Betrieben so ist oder in anderen Branchen weiß ich garnicht so genau. Das heißt, so eine Replikationsstudie, wo ich gucke, OK, der (Name) hat doch mal was weiß ich, bei mir schon etwas länger her, solche Untersuchungen gemacht. Ich habe aktuell in einer anderen Branche solche Untersuchungen gemacht, stimmen denn die Daten()? sind die stabil? Das nennt man Replikation.	
Interview 36		Das heißt, das ist schon ein risenreiz, also die eigenen Daten an anderen Daten zu prüfen, sie zu verallgemeinern, eine größere Datenbasis zu haben, das ist von großem Vorteil. Aber was finde ich genau sichern müssen, stelle ich mir vor, es muss klar sein, wie die Zugangsrechte sind.	

Interview 36	08:54	Ich glaube nicht, dass es sinnvoll ist zu sagen, die kommen in ein Portal und jeder kann damit machen, was er will - also die Zugangsrechte klären. Wir müssen unbedingt klären, wie das mit dem Datenschutz ist. Also mittlerweile ist ja, wenn sie mal das / die Datenschutzgesetzte nehmen, bin ich ja verpflichtet, bei jeder Untersuchung verpflichtet, dem Betrieb, den Menschen zu sagen, was mache ich denn mit deinen Daten.	
Interview 36		Ganz schwierig. Das heißt, wenn ich sage / Also wenn ich eine Untersuchung mache - haben wir mal gemacht in einer KiTa. Sage ich: passe mal auf, wir machen eine Untersuchung zur Gefährdung - nennt man das, ganz egal, was das ist- und wir machen das und das damit und die und die Auswirkung. Und dann ist auch gut. Und dann werden die Daten veröffentlicht und dann werden die nach zwei Jahren gelöscht. (-) Die Idee von Open Access ist aber ganz anders, die Daten sollen immer da sein und jeder darf damit nutzen, wie er will. Das heißt, da kommen wir mit dem Datenschutz in erhebliche Probleme, wo ja auch Leute widersprechen können, auch das ist ja eine (Möglichkeit): ach Mensch, ich gucke mir das gerade an, aber eigentlich gefällt mir das gar nicht mehr, ich möchte nicht mehr, dass sie meine Daten nutzen. Open Access sind sie irgendwo, irgendwo im Netz. Das heißt, wir müssen glaube ich da, sehr viele Gedanken verschwenden, wie wir das organisieren.	
Interview 36	10:00	Andersrum gedreht, was wir uns nicht leisten können ist, wenn man sagt, jeder ist verpflichtet - könnte man ja auch so rum drehen als Wissenschaftler - seine Daten irgendwo hinzugeben und jeder darf die benutzen.	
Interview 36		Zugriffsrechte, Datenschutz ist auch das, was wir regeln müssen	
Interview 36			Welche Beweggründe sehen Sie denn Daten mit anderen zu teilen?
Interview 36	10:30	Also wenn ich jetzt (unv.) im Prinzip genau das, was ich gesagt habe: Replikation, Generalisierung, die eigene Daten an anderen Daten prüfen. Das hat einen riesen Vorteil. Man könnte natprlich auch sagen, die meisten Forschungsprojekte sind mit öffentlichen Mitteln finanziert. Insofern hat natpürlich auch die Gesellschaft ein Recht, meine Daten zu nutzen. Das sind alles (.-) Dinge, die ich vernünftig finde, die sinnvoll sind. Dagegen spricht das große Problem, dass ich als Wissenschaftler die Schwierigkeit habe, was machen die anderen mit meinen Daten? Ideenklau? Mit einer Promotion deutlich gemacht. Ich gebe die raus und jemand nutzt die viel schneller. Dann natürlich auch diese ganze Schwierigkeit von Publikationskartellen. Komme ich da überhaupt ran? Werden die auch richtig wahrgenommen? Für mich als Wissenschaftler ist / deswegen gibt es ja auch diese Review Zeitschriften. Ich muss in bestimmte Zeitschriften kommen, weil nur die werden zur Kenntnis genommen. DAs führt, also in meinem Fach an bestimmten Instituten dazu, dass sie gar nicht mehr die Artikel sondern immer nur gucken, wo hat der veröffentlicht - Impact haben sie mal gehört	

Interview 36	11:43	Die ganzen Impact Factoren sozusagen: aha, der hat in so einem Journal drei mal veröffentlicht wunderbar. Das ganze fällt ja weg, dann. Das heißt, ich veröffentliche, ich gebe meine Daten irgendwo in die Öffentlichkeit. Welche Qualität das in Anführungsstrichen hat (-) stellt sich nicht jeder.	
Interview 36			Sehen Sie denn noch weitere Probleme und Barrieren beim Data Sharign?
Interview 36	12:11	Ein großes Problem ist ganz sicherlich auch, sie (unv.)hier im Landtag, wenn ich recherchiere, dass wir natürlich in den Fächern, in den Wissenschaften oder generell überhaupt keine Qualitätsstandards haben. Soll heißen, jeder packt seine Daten da irgendwie rein. Und (-) das macht ein Problem. Ich bräuchte ja idealerweise auch ein (-)ver, vernünftiges Muster die Daten irgendow dann auch kolectiv gehalten werden. Also sie machen jetzt eine qualitative Befrageung und schreiben da Text darein. Jemand anderes nimmt diese qualitativen Texte und macht da irgendwas draus (unv.)Und entsprechend dann Bewertungssystem. Das heißt wir haben dann wieder ein Sammelsorium von locker nebeneinander exisiterenden Daten - das würde uns nicht helfen. Das große Problem it, wir brauchen irgendwelche Qualitätsstandards zumindest in den Fächern. Wie müssen Daten aussehen, damit die da überhaupt reingebracht werden?	
Interview 36		Da gehören auch solche Sachen - das kennen Sie vielleicht in Sozialwissenschaften braucht man Mindestangaben über die Stichprobe, über die Population, über Teilpunkte oder sowas weiß nicht was. Weil mir hilft ein Datensatz gar nichts, wo ich nicht weiß wann der entstanden ist und woher der kommt und ähnliches. Also wire brauchen solche Qualitätsstandards und wir brauchen sicherlich sowas wie (-) eine Ressourcenabschätzung.	
Interview 36	13:30	Meint einfach, wenn das für mich als Wissenschaftler oder als Nutzer einen irsinnigen Aufwand bedeutet die Daten irgendwo reinzukriegen, mache ich das überhaupt nicht. Ich mache es ja zusätzlich. Ich habe die ja schon und jetzt verpflichte ich / werde ich verpflichtet die Daten auch noch irgendwo hinzugeben und muss da jetzt ein riesen Aufriss betrieben - das zahlt mir eh keiner. Das dritte ist dann - anderes Thema - wir brauchen dann natürlich eine Anreizstruktur weil ich muss mich ja fragen, warum sollte ich das eigentlichtun?	
Interview 36	14:03	Ich habe jetzt mit ganz viel aufwand - sie vielleicht später auch mal - mit ganz viel Aufwand eine Promotion gemacht und habe nächtelang nicht geschlafen oder was weiß ich was, und jetzt am Ende: jetzt gebe mir mal deine Daten. Also irgendwie weiß ich nicht (lacht). Ich habe den Aufwand getrieben und es gibt ja solche untersuchungen auch, da nutzt jemand anders - nennt man dann metaanalysendaten- nimmt die Daten von sechs Promotionen und macht da eine siebte raus. Irgendwo weiß ich nicht. Den Aufwand habe ich getrieben und der macht sich jetzt ein leichtes Leben in Anführungsstrichen und macht eine Metaanalyse mit meinen Daten.	

Interview 36	14:40	Auch da haben wir noch das Problem von Anreiz, von Vertauen, auch von Transparenz	
Interview 36			Sehen sie denn noch Probleme in der jetzigen technischen Ausstattung?
Interview 36	15:00	(--) Das ist eine gute Frage, ich muss mal darüber nachdenken. (5) Ich weiß es nicht wirklich, ich vermute ja. Das heißt, es ist einfach eine Frage des Zugangs auch da die Frage wer hält die Daten wo. Auch da bin ich wieder bei Datenschutz. Da kann man sagen, gibt bestimmt große Rechner in Amerika oder in Taiwan. (-) Die das wunderbar / oder die ganze Diskussion um Google und Co. Also möchte ich denn die Daten haben? Also auch da müssen wir uns glaube ich Gedanken machen, wie kriege ich ein öffentliche Struktur, einen öffentlichen Server hin, der auch demokratisch kontrolliert bleibt. Also ich kann nicht einfach sagen, ich nehme einen Anbieter - irgendeinen.	
Interview 36		Und was weiß ich, der beschließt nächstes Jahr: eigentlich möchte ich eure Daten nicht mehr rausgeben, die nutzen wir jetzt selbst - das ist schwierig. Auch da müssen wir uns Gedanken machen. Das ist ja eine Infrastrukturfrage. Wo werden solche Daten vorgehalten, wo werden solche Daten demokratisch kontrolliert?	
Interview 36	16:07		Nun haben Sie ja schon den Nutzen-aSpekt angesprochen: Wird denn Data Ihrer Meinung nach in der wissenschaftlichen Community anerkannt?
Interview 36		Immer mehr, immer mehr. Das hat was mit ich sage mal auch schon was mit den jüngeren Wissenschaftlern zu tun. Auch kritische müssen einfach feststellen, dass sie bestimmte Fragestellungen in klassischen Publikationsorganen nicht unterkriegen. In der Psychologie kann ich das ganz gut beurteilen, da gibt es eine sehr atarke Amerikanisierung und damit auch ein bestimmtes Format, wie so nein ARtikel in einem Journal auszusehen hat. Und einige sagen: ich habe eine qualitative Untersuchung - in der Psychologie können sie qualitative Untersuchungen eigentlich vergessen.	
Interview 36		Also in klassischen, richtigen Journals Apllied Psychologie. Haben / Da machen sie eine quantitative Untersuchung oder gar keine. Und jetzt haben wir viele, die sagen: ich habe eine gute Untersuchung gemacht, spannende Ergebnisse, würde die gerne veröffentlichen. Die kriegen sie aber nicht in diese Review Zeitschriften, weil da auch bestimmte STandards gelten und so weiter. Und da gibt es schon ein bestreben zu sagen: ich möchte sdas aber trotzdem veröffentlkichen. Das heißt von da kommt der Druck.	
Interview 36		Dann kommt der Druck natürlich auch der, das ist dann auch eine Frage der Finanzierung. Das wird immer teurer auch für Bibliotheken (-) gewisse Zeitschriften zu kaufen und auch vorzuhalten. Und Zeitschriften machen nur Sinn, wenn ich sie immer dauerhaft. das heißt also, wenn sie mal überlegen, mal so einen Artikel zu schreiben kostet aufwand, ihn irgendwie unterzukriegen kostet ein riesen Aufwand	

Interview 36		Sie schreiben den Sie zwei drei mal durch weil es durch Review Processe geht und dann plus kostet so eine Zeitschrift auch noch 280 Euro. Soll heißen, wir haben auch noch einen finanziellen Druck, wo man sagt, es macht keinen Sinn mehr. Und es macht auch desweegn keinen Sinn , weil es oft zu lange dauert. Wir bewegen uns ja in einer relativ schnellen, dynamischen Zeit. Und wenn mein Artikel von Untersuchung bis Veröffentlichung drei Jahre dauert, dann ist das zum Teil einfach zu lange.	
Interview 36		Wenn die ganzen Prozsse - soll nur sagen, es gibt schon gute Grunde zu sagen, wir brauchen schnelle, transparentere Publikationsorgane oder -portale, wie auch immer sie das nennen wollen, da gibt es schon Druck, glaube ich	
Interview 36			Nun haben Sie ja gesagt, dass die Politik den Rahmen schaffen kann für dieses Data Sharing (-) Es setzen sich ja jetzt nicht so viele dafür ein, wie ich gehört habe. Wer ist dafür denn zuständig und wie kann man das unterstützen?
Interview 36		Also ich glaube der Stärkste Impuls wird aus der Hochschulpolitik kommen. DA ist auch das größte Interesse und da wird auch Wissen geschaffen. Es ist ja auch vernünftig, dass es da herkommt. Und enn ich das richtig erinnere, nicht nur an der CAU, die haben glaube ich auch leitlinien für Open Access, wenn ich (unv) nicht vergessen habe. Und da sieht man ja auch schon, die wollen das ja unterstützen, dass eben die Wissenschaftlerinnen und Wissenschaftler entsprechend veröffentliche.	
Interview 36	19:10	Das heißt, es wird aus der Hochschulpolitik kommen und da ist auch der Motor nach neuen Formen der veröffentlichungen, nach neuer Organisation, nach schnelleren Veröfentlichungsformen. Und da bin ich ganz zuversichtlich. Also wenn man da mal jetzt mal so aus der (unv), dann sehe ich in vielen Hochschulen Bestrebungen, Leitlinien, die Bibliotheken die sich damit beschäftigen. Da glaube ich gibt es einen starken Impuls. wenn sie andere Organisationen nehmen, Sicherheit, Polizei, die beschäftigen sich damit gar nicht - also nicht als öffentliches Medium.	
Interview 36	19:38	Also nicht als öffentliches Medium, natürlich Polizei beschäftigt sich dmit der Frage, wie bekomme ich von anderen Polizei(-)einheiten Informationen, aber es hat nichts mit Data Sharing zu tun - schon, natürlich nicht ganz, weil Data Sharing findet bei denen statt, weil ich natürlich relativ schnell Länderübergreifend wissen muss, ob jemand für mich auffällig ist oder nicht, da machen die das, aber das ist was anderes als wir meinen.	
Interview 36	20:00	Wir meinen ja / also das gibt es schon, das gibt's glaube ich auch ganz gut bei denen, das weiß ich nicht genau. Wir meinen ja, also zumindest habe ich die ganze Zeit als Data Sharing als öffentliches Gut, Data Sharing geredet.	
Interview 36			Ja
Interview 36		Also Gehemindienste werden es auch haben, das wieß ich nicht.	

Interview 36			Nun haben Sie ja schon von Open Access gesprochen und auch Journale thematisiert, welche Verantwortung sehe / haben denn Journale im Sinne von Data Sharing?
Interview 36	20:43	Das ist eine Frage ob die in Zukunft noch so eine Plattform sein können. Ich bin durchaus der Meinung, dass diese Review Strukturen durchaus sinnvoll sind und auch erhalten werden müssen. Weil wir haben ja das große Problem, das fängt bei den Kids in der Schule schon an, wir leiden unter Informationsflut und woher weiß ich noch, dass das irgendwie Sinn macht? Insofern ist die Idee dieser Review Journals in der Tat, dass ich eine Struktur habe, (unv) Artikel, und dann prüfen Menschen, die sich fachlich auskenne, macht das ganze Sinn? ist das eine vernünftige Fragestellung? MAcht die Methode sinn? ist das nachvollziehbar? kann das so sein? Über solche Journal / Review Processe sind ja auch die entsprechenden Sachen aufgeflogen - koreanische Senior oder was weiß ich. Wo man einfach gesagt hat, das kann nicht sein, ich habe das im Labor selber gemacht, das funktioniert schlicht nicht. Das ist auch eine Sache, die ich wichtig finde. Ob das die Journals jetzt machen, ob die sagen wir bieten so ein Portal haben aber auch eine Review Struktur oder ob wir das irgendwie übertragen - das wäre mir schon ganz wichtig.	
Interview 36	22:00	Also wenn ich mir so vorstelle- das ist ja meine Kritik an Wikipedia - zu sagen, ich habe so ein Format und jeder kann, wenn das irgendwie einigermßen stimmt, kann das darein schieben. Dann hätte ich ein riesen Problem. Weil ich nutze natürlich - vielleicht ist das noch sehr kkonservativ, das weiß ich jetzt gar nicht so genau - aber ich nutze bestimmte Artikel mehr weil ich sage, dass ist halt Journal of Applied Psychologie, aber das weiß ich. Das mag mir nicht immer gefallen, was die einen oder anderen schreiben, aber ich weiß zumindest, dass die durch einen Review Prozess gelaufen sind. Und denn würde ich gerne erhalten und würde da eigentlich im Prinzip die klassischen Journals ermutigen: öffnet das, aber erhaltet diese Prüfroutinen.	
Interview 36	22:40		Nun wurden jetzt schon ganz viele Probleme und Barrieren angesprochen beim Data Sharing. Welche davon sehen Sie denn als größten Faktor momentan?
Interview 36	23:00	(überlegt) Also ich vermute, das ist eher eine behauptung, der größte Faktor ist, dass ich nicht weiß, was mit meinen Daten passiert - ich verliere völlig die Kontrolle. Das wäre für mich ein Problem.	
Interview 36			Inwieweit möchten Sie denn genau die Richtung Open Access, Open Data ausbauen oder unterstüzen.
Interview 36		Ich glaube, wir laufen ja fast den Hochschulen hinterher. Die haben die Leitlinien und sie verlangen von uns, dass wir das Urheberrecht verändern - wissenschaftliches Urheberrecht ist ein Stichwort. Sie verlangen von uns zurecht, dass wir uns dem Datenschutz nochmal widmen um das zu gewährleisten. In soweit ist das die Richtung, die wir auch gehen werden, das müssen wir auch tun.	

Interview 36			Gut. Haben Sie sonst noch weitere Aspekte, die sie mir zu dem Thema mitgeben wollen?
Interview 36		Ja, ich finde es ein spannendes Thema - gar keine Frage. Wenn man das jetzt auch eher wieder hochschulpolitisch sieht man ja auch, ist ein relevantes Thema. Es beschäftigt uns, aber ich glaube, und das schon fast zusammenfassen: solange wir nicht die Strukturen schaffen, werden wir dem hinterher schicken. Da gibt es eine Reihe an untersuchen, dass (-) zawr alle Wissenschaftler sagen: ja, das finde ich eine gute Idee, aber nur ein Bruchteil macht das. Und die Gründe sind genau die zu sagen, es gibt keinen Anreiz, ich weiß nicht was für ein Vorteil ich habe, Es überwiegend die Nachteile von Ideenklau bis Intransparenz. Und das ist im Prinzip die politische oder auch die gesellschaftliche Frage das zu ändern.	
Interview 36	24:47:00		Schön, vielen lieben Dank dafür.
Interview 36			Ich habe jetzt nochmal eine ganz andere Frage. Weil in Oslo habe ich mich mit dem Thema künstliche Intelligenz und Big data auseinander gesetzt (…). Wie schätzen Sie die technologische Entwicklung aud den Forschungsalltag ein?
Interview 36		Naja wir sehen es ja woanders. In Schleswig.-Holstein ist das eher stiefmütterlich. Natürlich kritiesieren wir das heftig. Es gibt jketzt hier einen Fond, KI Fond. Der hat glaube ich momentan drei Million. Das ist zum Vergleich zu den, was America und China ausgibt, ein Bruchteil. Alleine um Shanghai werden glaube ich 200 Million ausgegeben. Also in der Region Shanghai. (-) Und es wird uns im hohen Maße bestimmt - muss man ein bisschen auf dem Teppich bleiebn, nicht alles was KI genannt wird ist KI. Also es sind einfach nur Big Data. Big Data konnten wir immer schon. Das heißt, wenn Sie / ich habe früher auch mal praktisch gearbeitet bei den Berliner Stadtreinigungsbetrieben, die haben 10.000 Beschäftige und 1,5 Millionen Nutzer. Und da wird jeden Monat die Rechnung (unv) gemacht. DAs ist nicht KI, das ist einfach nur 1,5 Millionen Datensätze. Das heißt, da muss man aufpassen und wenn die jetzt die 1,5 Mill. Datensätze auswerten ist es immer noch kein KI. Dann stellen sie fest wie viele Kunden, was weiß ich, eine große, kleine, runde Tonne habenn. DAs ist alles kein KI. KI wird es ja erst, wenn ich es wirklich (-) ein lernendes System habe. Das heißt, wenn ich im Prinzip eine Software auf einen Datensatz setze und diese Software entdeckt neue Strukturen indem sie die Algorithmen optimieren	
Interview 36	06:38	Dann wird es KI. Und das ist schon sinnvoll. Und das wird auch schon massenhaft genutzt. Ich bin fest davon überzeugt, dass wird auch zunehmend von Bedeutung werden. In der Diagnostik kennen das schon, in der Medizin, da ist die ganzen bilderkennenden Verfahren, das heißt, wo eben ein Mustererkungsprogramm DInge erkennt, die ich nicht mehr sehe. Also, das läuft, also in (ort) ist das schon sehr schön zu sehen, wie das funktioniert.	

Interview 36	07:02	Und das wird eine zunehmend große Bedeutung kriegen, weil einfachquasi zu lernen aus großen Mustern irgendwas zu machen und (unv) mein Sohn beim (-) bei der GIZ, der Gesellschaft für internationale Zusammenarbeit, die haben gerade aktuell - was machen die? - ein riesiges KI Projekt. Wozu? Zusammen mit afrikanischen Ländern, weil auch da die Frage genau umgedreht ist. Die Afrikanern sagen: ok, wir wissen, überall ist KI. Wir würden da auch gerne was amchen. Das heißt, die GIZ überlegt gerade: wie kann ich denn KI in den afrikanischen Ländern von bedeutung machen. Das ist politisch hoch kompliziert. Weil (-) so ein Diktator könnte auf die Idee kommen zu sagen: MEnsch, ich würde gerne ganz genau wissen, was meine Leute machen. Baut mir doch mal ein System, ein Überwachungssystem, wo ich jederzeit weiß, wo sich (Name) befindet und was er macht. Das fände ich keine tolle Anwendung.
Interview 36	28:08:00	Aber es könnte eine KI-Anwendung zu sein, weil es hat was mit Bilderkennung zu tun, mit Raumbewegung und ähnliches. Und das können sie nur mit KI machen, weil solche Algorithmen können sie garn icht schreiben. Insofern, die Entwicklung wird kommen und sie wird schneller kommen als wir uns das Denke. Weil natürlich auch, wenn sich Programme erstmal selbst optimieren, optimieren sie sich immer schneller selbst.
Interview 36		Und das ist ja schon faszinierend, wenn sie mal sehen, ich weiß nicht ob die das bei einer Bilderkennung schon gesehen haben, wenn irgendeine Software lernen soll eine Kuh zu erkennen. Das dauert zuerst wahnsiniig lange. Und irgendwann in der lage, eine Kuh in der Entfernung bei Hundert Giraffen und zweihundert Elefanten zu erkennen. DAs heißt, was wir dann nicht mehr können. Und insofern muss man sagen, es wird schnell gehen und als Gesellschaft, das ja ein Teil was wir hier machen, wie viel wir davon mitmachen wollen (-) also es gibt durchaus die Diskussion zu sagen, irgendwann sind die besser in der Erkennung.
Interview 36	29:05:00	Will ich dann das System dem überlassen? Das ist beim Autonomen Fahren ein rieseinproblem, womit wir uns beschäftigen. Das heißt. Es gibt einen Punkt, wo man sagt, im Prinzip / das kennen Sie, diese Dilemmata. Das heitß, ich fahre mit dem Auto, Zebrastreifen, eine Mutter mit zwei Kindern geht über den Zebrastreifen und sie hat es nicht gesehen und rechts ist eine ältere Dame - einen von beiden fahre ich um. Wen ich fahre ich um? Gott bewahre, dass mir sowas jemanls passiert. Und jetzt ist die Frage, überlässt du das einem Alhgorithmus, der KI, zu sagen: ja ok, drei Kinder vs. ein, Lebenserwartung und was weiß ich - das ist doch ganz einfach, die alte Dame wird umgefahren. DAs geht ethisch nicht (lacht). Man kann nicht irgendwie Jahre aufrechnen. DAs wird die spannende Frage bleiben. SAgen wir: im Prinzip entscheidet der Rechner?

Interview 36	30:10:00	Und in der tendenz gibt es ja durchaus gute Gründe zu sagen, er wird irgendwann besser entscheiden .- in der Summe. Der wird den Abstand besser berechnen als wird und sagen: ich bremse, auch wenn es dir nicht gefällt. Weil ich weiß, bei diesem Abstand, bei diesen Witterungsverhältnissen, und wie der sich vor dier Verhalten hat, ist es vernünftig jetzt abzubremsen. Da ist es (unv.) fünf Minuten späer, ist nicht spo schlimm. Aber wir werden häufig in die Situation kommen, wo wir sagen, ne (-) und es gibt ethische Probleme und es gibt rechtliche Probleme. Was machen sie denn? / also ich habe da eine Funktion in meinem Auto, wenn das bald soweit, aktuell haben wir sowas nochg arnicht, ich habe eine Funktion in meinem Auto, die heißt, ich kann noch die Entsheidung des Rechners (-) kontrollieren. Ich bremse nicht und fahre dem hinten drauf. Und jetzt kann man juristisch sagen, dann zahlst du doppelt, weil der Rechner - das haben wir geprüft - hat gesagt, der zahlt, bremsen. Sie merken, es wird kommen (-) und wir werden sehr schnell Gedanken machen müssen, was wir advon machen wollen.	
Interview 36	31:00:00	Und wie wir damit umgehen müssen. Das sind ethische Probleme, das sind soziale Probleme. Vvon Teilhabe über wer verfügt darüber, wer verfügt darüber nicht. Die Entwickung und so weiter. Kennen Sie in Kaiserslaiter Deutsches Zentrum für Künstliche Intelligenz.	
Interview 36			Habe ich schonmal gehört, ja.
Interview 36		Gehen Sie da mal hin. Da kann man sehen, was da alles passiert, das ist schon spannend. Kann man auf der Homepage auch sehen. DA sieht man wirklich, was als Entwicklung schon da ist. Und was mich immer rumtreibt, ist ja gerade das, was wir hier machen. Das eine ist die technische Entwicklung und unser Job ist die gesellschaftliche zu kontrollieren und auch zu sagen: nicht alles, was technisch machbar ist, werden wir auch tun.	
Interview 36		Aber dafür brauchen wir mehr stimmen.	
Interview 36			Mal gucken, wie das mit Data Sharing dann aussehen wird.
Interview 36	32:10:00	Das ist das gleiche. Man muss eben wissen, was man damit machen will. Sonst geht das berühmte Kind den Bach runter.	
Interview 37	Time	Interviewee 37	Interviewer
Interview 37	01:40		Meine erste Frage ist: Was gehört so zu ihren täglichen Aufgaben?
Interview 37		Meinetäglichen Aufgaben umfassen hauptsächlich administrative Tätigkeiten. Emails schreiben, viel telefonieren, Anträge schreibben, Meetings leiten und Forschungsprojekte organisieren, ja. Das was ich machen muss, auch die Labortätigkeit organisieren und die Diagnostik vom (Unternehmen). Und/ Also zum Forschen kommt man jetzt nicht so häufig wie man sich das wünschen würde.	

Interview 37	02:26	Das sind dann die Tätigkeiten. Wir sind drei Direktoren am Institut. Das Institut hat etwas über 100 Mitarbeiter und eigentlich sind es strwng genommen auch nur zwei Direktoren, die da für das Tagesgeschäft. Herr Schreiber ist ja mehr an der Klinik. Insofern das ganze Tagesgeschäft, was an so einem institut hier anfällt. Gelder ranoholen. Ganz einfach	
Interview 37	02:51		In welchen Data Sharing Aktivitäten sind Sie denn involviert?
Interview 37	02:57	In diversen - das wird Herr (Name) sie auch erzählt haben. Wir leiten das Daten Managment des Exzelenzclusters. Precision medicin inflamation -a slo PMI. Da gibt es ein großes Teilprojekt Datenmanagement wo wir die Stützstelle zwischen Exzellenzcluster und Medizininformatik im moment abbilden - zu Professor (Name). Dann haben wir einen DFG Antrag speziell nur zum Daten Management wo wir gerade ein Fortsetzungsantrag stellen,. Da geht es auch um Omics Daten, also daten die hier in den Laboren gewonnen, dass wir die hier managemen vernünftig. Und das soll irgendwann mit den medizinischen Daten aus dem Patientensystem verschnitten werdem. Dann leiten wir hier ein Teilprojekt im SFB, Sonderforschungsbereich F82 zum Thema Datenmanagement - also das ist der Bosch SFB in der Zoologie. Jetzt bekommen wir eine Forschergruppe von der DFG bewilligt diese Woche. DAs sieht ziemlich gut aus, da haben wir auch ein Datenmanagement dirn. usw. Da gibt es noch andere Drittmittelaktivitäten mit Hamburg zusammen mit dem UKE. Da betreuen wir auch einen TEil des klinischen Datenmanagements mit den Internisten und so weite.r	
Interview 37	04:18		Und inwieweit verwenden Sie dann Daten von anderen bei ihrer Forschung?
Interview 37	04:23	Tagtäglich. Also man kann in unserem Bereich kann man ja super Artikel auch sehr hochrangige Artikel in Nature Genetics, usw kann man mit existierenden Daten publizieren. Bestes Beispiel wir arbeiten ja mit Patientedaten, mit Daten vom Menschen hauptsächlich hier, weil wir sind ja hier in der Medizinfakultät. Es gibt in England eine Biobank, die nennt sich UK Biobank. Die haben von 500.000 Leuten haben die verschiedene Daten erhoben. Also die ganzen PAtientendaten sind verfügbar, Genetische Daten. Und diese DAten kann man beantragen. Man bezhalt eine kleine Gebühr für administrative Tätigkeiten, aber dann bbekommt man Daten von 500.000 Personen. Im Vergleich: Deutschland baut gerade eine Kohorte mit 200.000 Leute auf. Die sie noch nicht ganz recrutiert haben. Ich glaube ,deie sind so bei mindestens 140.000. Die NAtionale Kohorte - die werden 30 Jahre nachbeobachet. DA gibt es noch kaum Daten. Das heißt, die werden jetzt erhoben und generiert. Das ist / zeigt einfach dass wir da ein bisschen zurück sind, aber international, also UK biobank ist da Vorreiter und das nutzen wir zum Beispiel, ja, aber da gibt es noch ganz viele andere Resourcen auf die wir zurückgreifen.	
Interview 37	05:47		Und welche Probleme treten dann auf, wenn Sie die Daten von anderen verwenden?

Interview 37	05:52	Welche Probleme? Das größte Problem ist wirklich, wenn die Person in den Artikeln sagen, die Daten sind verfügbar auf Anfrage - haben wir gerade erst wieder einen Fall. Renommiertes Journal - ich meine Nature Medizin. Und Kollegen aus Stanford, die geschrieben haben , die Software und die Daten sind verfügbar. Und dann haben sie uns die Software geschickt, aber nicht die Daten, die man braucht für Auswertungen. Und, diverse Nachfrage, wir kriegen die Daten nicht. Das heißt, man kann das nicht reproduzieren, was sie gemacht haben, man kann das nicht anderweitig verwenden. Das sind leider so Einzelfälle, hoffe ich mal. In vielen Fällen muss man als Gutachterselber darauf auchten, dass die Leute gezwungen werden, ihre Omics Daten in entsprechende Archive zu bringen. Jeder Artikel sollte reproduzierbar und nachvollziehbar sein und da muss man nunmal als Gutachter darauf achten. Genau. Das ist im Wesentlichen das Problem. Aber wie gesagt, in der Genetik sind wir ja quasi gezwungen worden zusammen zu arbeiten, weil man muss mit großen Kohorten uund Fallzahlen arbeiten. Weil gerade wenn man sich seltene Mutationen im Erbgut anguckt, seltene Varianten, dann brauche ich 100.000 und mehr Datensätze um überhaupt statistisch signifankte Befunde zu generieren. Gerade für seltene Erkrankungen. Und da muss man zusammen arbeiten. Das geht nur international. Auch wenn man sich nicht mag, -also die Leute haben sichz usogenannten Krankheitskonsorten zusammengeschlossen inder Genetik und dafunktioniert das dann wunderbar.	
Interview 37	07:43		Wie kommen Sie denn momentan an die Daten ran?
Interview 37	07:46	Es gibt verschiedenste Quellen. Entweder die sind direkt herunterladbar auf den Homepages als sogenannte Summery Statistics, also das sind dann keine identifizierbaren Daten sondern zusammenfassende Statistiken. Mit denne kann man eigentlich die allermeisten analysen auch abdecken. Und das ist auch so der goldstandard, dass man diese summary statistics ohne auflagen teilt. Oder man trägt anträge bei den Biobanken. Es gibt ein europäisches Biobankennetzwerk BBMEI, da kann man rüber gehen. Und dann gibt es entsprehcend beim NIH und beim EBI in Europa gibt es die netpsrechenden Archiven, da kann man das beantragen. Entweder man kann sich das direkt herunterladen. Wenn es genotyp und phänotyp daten zusammen sind dann muss man häufig noch die Schleife über so ein Begutachtungsgremium gehen. Auch in (Ort). Wenn sie hier in (Ort) daten beantragen,gehen sie immer über die Biodatenbank Popgen oder das P2N nNetzwerk und dann gibt es immer noch so eine Evalutaion, weil die Ethikkomission dem auch zusstimmen muss.	
Interview 37	08:58		Welche Anforderungen stellen Sie an die Daten?

Interview 37	09:00	Welche Anforderungen an die daten stellen? Da gibt es je nachdem was für analysen wir machen möchten. Wenn es genetische Daten sind und summery statistics, das ist standardisiert wie das auszusehen hat und was man da braucht. Also da geben wir dann vor wie die Spalten heißen sollen. Klar, je mehr Metadaten man hat, desto besser, gerade wenn es andere Omics analysen sind wie Expressionsdaten oder Mikrobiomdaten. Beim Mikrobiom möchte ich auch nochg erne wissen, was haben die Leute gegessen - weil Ernährung einen großen Einfluss hat. Also das ist ganz heterogen, das kann man nicht so pauschal sagen, aber klar. Ein Datensatz ist umso mehr wert, je mehr Metadaten man noch dazu hat. Das merken wir hier am Institut auch immer mehr.	
Interview 37	09:50		Und wie stellen Sie sich dann den zukünftigen optimalen Zugriff auf benötigte daten vor?
Interview 37	00:10:00	Ja also, Wir nehmen da eigentlich eine extreme Position ein.Also ich würde das unterstützen was Herr (Name) da im Moment vorwärts treibt von der CDU, dass er, sagt dass eigentlich jeder dazu verpflichtet sein sollte, seine Daten auch zu teilen seine medizinischen Daten für Forschung, dass man nicht nur einen Organspendeausweis hat, sondern auch ein Schattenspender Ausweis. Ganz nach dem Vorbild Skandinavien, Dänemark. Da gibt es nur ein Opt out System.	
Interview 37	00:10:30	Also erstmal ist pauschal jeder dabei von Geburt an werden Proben und Daten gesammelt für Forschungszwecke. Und damit kann auch dann jeder arbeiten man muss das allerdings beantragen und natürlich bestimmte Dinge beachten. Es ist klar dass man nicht die Klartext Namen sieht und dass das hinter geschützten geschützten Bereichen stattfinden muss auch hier findet das ja hinter der Klinik ja wohl statt. Es muss schon ja. Also es muss wirklich ein einfaches Verfahren geben aber es muss auch verfahren sein was sicher ist und was welches dann die Bevölkerung natürlich auch das entsprechende Vertrauen hat. In Deutschland gibt es ja leider ein sehr großes Misstrauen und damit muss man jetzt einfach mal auf warum auch immer Die Skandinavier sind dafür viel entspannter.	
Interview 37	00:11:27		Jetzt haben wir ja schon viel darüber gesprochen welche anderen sie welche Daten teilen sie mit anderen Wir teilen mit anderen.
Interview 37	00:11:39	Also erst einmal viele der Proben die wir benutzen liegen in der Biobanken. Und wenn wir Daten für diese Proben benutzen, dann geben wir diese Daten an Pop-Gen zurück, und Pop-Gen bietet die dann an und kann sie verteilen. diese sind im Prinzip sodass Daten Verteilzentrum das Datenmanagement Zentrum dann für die Popgen-Kohorte wir selber teilen natürlich die Daten wenn wir veröffentlichen wenn wir Peer-Reviewed Artikel veröffentlichen dann sind wir dazu verpflichtet die Daten zugänglich zu machen.	
Interview 37	00:12:19	Ganz einfach Es wird nicht immer passieren.	
Interview 37	00:12:25	Aber wir achten darauf dass wir das machen.	

Interview 37	00:12:27		Das heißt, welche Beweggründe sehen Sie dann die Daten zu teilen?
Interview 37	00:12:32	Beweggründe? Transparenz. Transparenz und Reproduzierbarkeit das sind die wichtigsten Punkte und ein anderer Grund ist auch das sehr viel Geld gekostet die Daten zu generieren und auch die Proben zu sammeln. Umso schöner ist es wenn andere die Daten dann nochmal benutzen. Ich sage Das war so eine Eintagsfliege. Wir haben das jetzt nur für unser eigenes Paper benutzt sondern ganz viele andere nutzen das auch noch.	
Interview 37	00:13:02	Wir sehen das wirklich ganz liberal und entspannt. Je mehr Leute die Daten dann nutzen desto besser.	
Interview 37	00:13:08	Also gerade von diesen gesunden Kontrollgruppe soll jeder benutzen für ihre Krankheit bezogenen Analysen. Das heißt Die Kontrollen sind ja unkritisch die kann eigentlich jeder gebrauchen.	
Interview 37	00:13:22		Welche Probleme treten dann auf beim Teilen?
Interview 37	00:13:29	Das einzige was mir da einfällt ist der Zeitverzug. Es gibt dieses Antragsverfahren und das kann manchmal recht lange dauern mehrere Wochen weil auch die Ethikkommission häufig rein guckt. Es dauert zu lange das ist ein Problem. Das zweite Problem ist schafften Diskussionen das übliche. Da gibt es häufig auch gerne mal Diskussionen.	
Interview 37	00:14:01	Muss man die Daten jetzt einfach verfügbar machen so dass die Leute machen können was sie wollen oder gibt es eine Auflage dass man sagt Ihr kriegt die Daten aber ihr müsst dann nachher auf der Publikation ein zwei Leute aufführen weil die Daten einfach sehr wertvoll sind und weil es auch Arbeit ist die Daten zu teilen und aufzubereiten und wir von den Artikeln widerlegen weil das unsere Werbung ist.	
Interview 37	00:14:29	Das gibt es ab und zu. Meistens ist das unkritisch beeinflussen denn finanzielle Aspekte beim geringsten Fall. Es ist eher ein bisschen die Bequemlichkeit dass die Wissenschaftler für die Mehrarbeit die Daten zugänglich zu machen oder wenn ein Doktorand uns verlässt dass er seine Daten zugänglich macht und eine übergabe macht. Das ist eher Bequemlichkeit auf beiden Seiten.	
Interview 37	00:15:01	Man muss ja selber auch als Chef darauf achten was ein gutes Datenmanagement erfolgt und eine übergabe erfolgt. Aber finanzielle Aspekte werden bei uns weniger.	
Interview 37	00:15:13	Daran scheitert es nicht.	
Interview 37	00:15:17		Ist es denn mit technischen Aspekten beeinflusst. Gibt es da Probleme?
Interview 37	00:15:25	Das hat schon am besten formuliert. Wir haben im Moment Probleme Cardinale Datensätze zu managen.	
Interview 37	00:15:35	Wenn wir zu einer Person Daten von mehreren Zeitpunkten haben da gibt es jetzt hoffentlich endlich mal ein IT-System mit dem man das vernünftig machen kann.	
Interview 37	00:15:49	Ansonsten ist das technisch gelöst werden die rechtlichen Punkte rechtlich verkehrten.	
Interview 37	00:16:01	Das interessiert uns weniger da hören wir auf die Experten.	

Interview 37	00:16:06	Wir sind dazu verpflichtet unsere Projekte mit der Ethik-Kommission über Ethik Anträge abzusprechen mittlerweile auch müssen wir auch Datenschutz Konzepte formulieren das heißt da guckt der Datenschutzbeauftragte drauf.	
Interview 37	00:16:23	Und wenn es irgendwo Schwierigkeiten gibt lassen wir uns hier von Professor (Name)) auch gerne mal beraten und unterstützen der unterstützt auch die Ethikkommission der Medizin. Das sind so unsere Experten. Ich bin da auf ganz dünnem Eis.	
Interview 37	00:16:40	Aber ich weiß was ich machen darf und nicht machen darf mit anonymen Daten darf man eigentlich fast alles machen auch Firmen dürfen mit anonymen Daten arbeiten.	
Interview 37	00:16:51	Aber natürlich Genetik und andere Sachen hinzukommen. Da gibt es jetzt keine großen Hindernisse im Moment.	
Interview 37	00:17:02	Denn das Teilen von Daten in der wissenschaftlichen Community anerkannt ja es gibt ganz viele tolle große Projekte wie es sie auch bei den Physikern gibt die eigentlich nur kollaborieren international und solche Großprojekte gibt es hier auch. Und wo man die Daten direkt über den Web-Browser abfragen kann analysieren kann. Das sind sehr sichtbare Projekte das sind aber wirklich auch sehr teure Projekte große Datensätze sehr sichtbare Dinge jetzt so klein ist es einfach gut dass gute wissenschaftliche Praxis eher.	
Interview 37	00:17:52	Man bekommt ja eher Probleme als es nicht tut. Viele publizieren ohne ihre Daten zu veröffentlichen dann haben die Gutachter geschlafen. Man muss einfach als Gutachter Leute zwingen sich nicht um gute wissenschaftliche Praxis geht es weniger geht es mehr um Stil.	
Interview 37	00:18:27		Welche der genannten Probleme haben ja jetzt schon viel darüber gesprochen was alles das Sharing Verhalten beeinflusst. Welche der Probleme oder Barrieren wirkt denn am stärksten Ihrer Meinung nach.
Interview 37	00:18:43	Ich bin nicht für unser Institut hier spreche ich der extra Aufwand pro Person. Das muss einfach so komfortabel wie möglich gehalten werden dass man als Wissenschaftler seine Daten irgendwo abkippen kann. Deswegen haben wir ja auch extra Daten Manager eingestellt die Arbeit abnehmen sollen und das zweite sind die Timss. Wenn wir Daten Biobanken beantragen kann es nicht sein dass man manchmal zwei Monate warten muss weil gerade für eine Doktorarbeit oder wenn man will die Daten ja beantragen und dann möglichst schnell zurück kriegen dass man weiterarbeiten kann.	
Interview 37	00:19:25		Das habe ich noch eine Frage aus einem ganz ganz anderen Thema und zwar habe ich mich in Oslo mit künstlicher Intelligenz und Big Data beschäftigt. Da würde mich interessieren, wie schätzen Sie denn den Einfluss dieser Technologien ein?(…)
Interview 37	00:20:02	Auf den Forschungsalltag auf den Forschungsleiter auf jeden Fall.	

Interview 37	00:20:10	Wir machen hier ja viel Bioinformatik im Institut. und für die Bioinformatik ist der Bereich nichts Neues. Maschinelles Lernen das ist ein Feld das gibt es schon seit mehreren Dekaden in der Bioinformatik und benutzen die Methoden hier regelmäßig. Klassische Classification Analysen werden häufig genutzt um Biomarker zu finden. Mit Support Vector Machines.	
Interview 37	00:20:46	Da gibt es verschiedenste Verfahren künstliche Intelligenz hingegen das ist ja sozusagen die nächste Generation dieser neuronalen Netze.	
Interview 37	00:20:57	Und das nutzen wir gerade auch in einem Forschungsprojekt. Ist unheimlich performant weil man das auf speziellen IT Architekturen laufen lassen kann Grafikkarten Cluster.	
Interview 37	00:21:11	Es geht schnell, viel schneller als klassische neuronale Netze oder andere maschinelle Lernen verfahren und es ist sehr präzise und gut. Je mehr Daten man da rein gibt desto besser.	
Interview 37	00:21:28	Und daran hängt es noch ein bisschen in der Medizin, dass wir in Deutschland erst einmal aufholen müssen und die ganzen Patientendaten verfügbar gemacht werden müssen und strukturiert werden müssen. Wir hängen Jahrzehnte zurück hinter den anderen Ländern. Deswegen sage ich immer erst mal unsere Hausaufgaben machen bevor wir dann über künstliche Intelligenz reden. Trotzdem nutzen wir das schon jetzt für uns nicht so etwas Besonderes ist wie man so schön sagt für etwas was es schon lange gibt. Aber klar dadurch dass es jetzt auf größeren Rechnern laufen kann parallel kann man viel größere Datensätze prozessieren.	
Interview 37	00:22:18		X Da wollte ich mal fragen ob sie welche kennen die am Anfang eine Gruppe festlegen wollten eine Datenbank aufsetzen also eine Plattform zum Datenaustausch und die dann im Laufe der Zeit Collaboration Partner dazugewinnen wollen mit einem wachsenden Netzwerk.
Interview 37	00:22:57	Es setzt sich ein Team zusammen aus verschiedenen Unternehmen oder Instituten ob ich euch ein Netzwerk kenne oder ob sie eine Datenbank kenne oder ein Netzwerk das so gemacht hat.	
Interview 37	00:23:08	Am Anfang rein gesetzt und dann wachsen die immer mit mehreren Partnern.	
Interview 37	00:23:13	Ach so ja das findet ja gerade in Deutschland statt im Bereich Medizin und Informatik ist diese vom BMBF die große Medizin Informatik Initiative Heimat. Das ist gut aber ich suche genau diese.	
Interview 37	00:23:32	Hier. Das. Wird jetzt gefördert.	
Interview 37	00:23:37	Ist die Medizin Informatik Initiative da geht es nur ums Datenmanagement in der Medizin. Und jetzt befinden wir uns in der Aufbauphase Verletzungspause und Thiel ist im sogenannten heimatverbunden. Da gibt's verschiedene Verbünde.	
Interview 37	00:23:55	Das ist jetzt (Ort) genau hier.	
Interview 37	00:24:05	Das heißt im Rahmen des Konsortiums wird das immer wieder erweitert.	

Interview 37	00:24:14	Das ist schon mal die Neuerung also jeder der Standorte muss natürlich selbst auf Vordermann bringen aber die haben gemeinsame Standards.	
Interview 37	00:24:23	Und dann irgendwann wird es natürlich soweit kommen dass man sagt es wolle die verschiedenen Hubs auch mal zusammenschalten bis man dann deutschlandweit auch alle Patienten an den Universitätskliniken abgreifen kann Medizin Informatik Initiative oder Heimeroth hat auch eine eigene Seite dabei.	
Interview 37	00:24:50		Kennen Sie denn sonst noch andere Datenbanken oder Plattformen die auch Partner überzeugen müssen teilzunehmen oder wollen stetig wächst in Deutschland in Deutschland kann Europa sein. In Deutschland gibt es jetzt hier die.
Interview 37	00:25:20	Initiative.	
Interview 37	00:25:25	Da gibt's von der DFG diese Ausschreibung und da sind wir auch bei zwei Initiativen dabei. Es startet jetzt aber werden erst mal die Anträge abgegeben aber da geht es genau darum unsere Spezialgebiete zu definieren wie zum Beispiel Gilo oder Mikrobiom. Bei den beiden sind wir dabei. Und da bildet sich jetzt so ein Nukleus. Das ist schon die Idee dass das dann auch national sichtbar zentrale Datenmanagement und Netzwerke werden abseits vom Datenmanagement in den Forschungsbereichen.	
Interview 37	00:26:12	Lokale Netzwerke das läuft ja über Verbund Projekte die wir selber beantragen. Aber das ist wenig dabei was verstetigt wird. Ganz wichtig für unseren Bereich Deutsches Netzwerk für Bioinformatik Infrastruktur. Das ist jetzt verstetigt und da sind wir assoziierter Partner. Das ist eine ganz tolle Sache für die Bioinformatik Training und das Elixier das gibt's länger. Deutschland hat sich leider nie an Elixier angehängt damals wurde erst mal das DNB gegründet das ist gewachsen. Jetzt hat das sind die wichtigsten Netzwerke alles andere sind für uns eher kleinere Sachen.	
Interview 37	00:27:11	Oftmals ist es ja so dass eine Gruppe von Instituten oder sagt wir eine Datenbank aufbauen und dann bleibt dabei ja von vornherein schon festgelegt haben wir sind der Kreis erweitern.	
Interview 37	00:27:25	Es gibt bei uns leider schon sehr viele gute die sich einfach etabliert haben. Da sehe ich wenig was da neu wächst.	
Interview 37	00:27:40	Auf jeden Fall bieten ganz viele Services an dem Rahmen finden Sie auch diese Datenbanken Borstel in Schleswig-Holstein mit Lipide Comics die haben eine ganz tolle Datenbank Schwerpunkte gebildet der Schwerpunkt analytischer Natur gelungen.	
Interview 37	00:28:07		Haben Sie denn sonst noch irgendwas mitgehen wollen.
Interview 37	00:28:13	Nein ganz wichtig. Jede Universität muss sich diesen Luxus leisten weil es einfach genau wie ein Rechenzentrum ist das Herzstück einer Universität ist das Wissen sind die Daten und das muss vernünftig gemanagt werden.	

Interview 37	00:28:36	Da würde ich mir wünschen dass da noch ein bisschen mehr passiert. Vieles ist ja auch über die Fakultäten hinweg betrifft nicht nur die Lebenswissenschaften. Da können wir auch gut zusammenarbeiten. Das ist sicherlich auch etwas Es gibt hier einen kleinen Nukleus einer Datenmanagement Team um Paul herum aber das muss man einfach ausbauen und vor allen Dingen auch weiter fördern und das geht nicht nur mit Forschungsmitteln.	
Interview 37	00:29:10	Ansonsten ein wichtiges Thema. In jedem Verbund Projekt sollte man immer ein Projekt mit beantragen.	
Interview 37	00:29:25		
Interview 37			Das sind dann noch ein paar Punkte für die Statistik der höchste Abschluss (...)
Interview 38	Time	Interviewee 38	Interviewer
Interview 38	00:00:01		Möchten Sie dass ich von vornherein noch eine kurze Einführung zu dem Thema gebe.
Interview 38	00:00:07	NFDI kenne ich gut. Ist halt die Frage was sie selber erreichen wollen.	
Interview 38	00:00:10		(...) und im ersten Schritt geht es um Motivationsfaktoren und Barrieren diese (Data Sharing Platforms) zu nutzen. Das heißt in dem Interview habe ich jetzt 15 offene Fragen auf die Sie ganz frei und offen antworten können. Zu dem Thema Data Sharing Datenbanken Nutzung Probleme.
Interview 38	00:00:39	Ich bin nicht nur der große Spezialist Nutzung in dem Bereich gearbeitet.	
Interview 38	00:00:45		Wir müssen ja genau gucken was da so kommt. Haben Sie denn von vornherein irgendwelche Fragen Anmerkungen.
Interview 38	00:00:55	Nö eigentlich nicht. Sie wissen ja dass sie angesprochen.	
Interview 38	00:01:06		Das Interview wird transkribiert und auch nur für meine Arbeit verwendet (...). Das wird dann alles anonym verwendet. Meine erste Frage an Sie lautet Was gehört zu ihren täglichen Aufgaben?
Interview 38	00:01:27	Sie wissen ja, dass ich eine klassische Bibliothek leite. Und diese Bibliothek ist noch eine klassische Bibliothek. Und die entwickelt sich gerade in ein moderneres Informationszentrum und jetzt habe ich zwei oder drei wichtige Aufgaben die eine Sache ist natürlich die Videothek am Laufen halte. Das Zweite ist dass sie sich auf den digitalen Wandel einrichten. Das Dritte ist dass sie Forschung aufbaue. und das soll natürlich alles zusammenpassen. Also als Gesamtausgabe kann man sagen dass wir uns von der klassischen zur digitalen Bibliothek bewegen und uns dort.	
Interview 38	00:02:01	Von Verwaltung von Literatur hin bewegen zur Begleitung von Forschern bezügig ihrer Literatur Daten. Also dass wir die auch anbieten einsammeln, dass wir halt Qualitätskontrolle machen.	
Interview 38	00:02:16		Und in welchen Data Sharing Aktivitäten sind sie so involviert?
Interview 38	00:02:21	Das ist eine gute Frage. Überlegen wir mal kurz, was Sharing ist. Sie meinen praktisch dass man Open Access Daten anbietet, richtig	

Interview 38	00:02:31		Die Frage zielt eigentlich darauf ab was man allgemein macht. also im Rahmen von Kollaboration oder freiwillig irgendwo Daten Open Access zur Verfügung stellen. Solche Aktivitäten
Interview 38	00:02:45	Unser Angebot ist eine Plattform. Diese Plattform ist so dass wir dort Daten und Literatur anbieten. Das ist alles noch im Aufbau. Aber da gibt es schon. Da gibt es im Grunde drei verschiedene Dinge, die gleichzeitig angeboten werden.	
Interview 38	00:03:03	Das ist einmal Normale Literatur, so im Open Access Journal. Das zweite ist etwas, das wir Living Handbuchs nennen. Das heißt Wir erstellen Bücher mit Editoren zusammen online Open Access und die sind dann verfügbar während sie noch geschrieben werden.	
Interview 38	00:03:19	Das Dritte ist dass wir ein Daten Portal anbieten. Jetzt überlege ich gerade, in welche Aktivitäten wir sind. Wir sind Im Allgemeinen in der semantischen Webcommunity Open Data aktiv. Wir haben jetzt hier konkret bei ZMED noch keine Datenquellen aufgebaut.	
Interview 38	00:03:37	Allerdings war ich In der Vergangenheit war ich schon involviert in Datenbanken die Open Access angeboten wurden.	
Interview 38	00:03:46		Jetzt kann ich mal weg von den Daten selber anbieten. Meine Frage zielt jetzt ab in die Richtung. Inwieweit verwenden sie Daten von anderen in der Forschung?
Interview 38	00:03:59	Wir machen ja nicht nur Forschung sondern wir haben ja auch. Wir bieten ja auch Inhalte an die Literatur und jetzt mache ich mal die klassische Bibliothek dort werden viele Daten zur Katalogisierung von Büchern wiederverwendet. Dort gibt auch offene Datenquellen. Aber im Forschungsbereich verwenden wir viele Ontologien Terminologie. Und dann auch Software oder Tools die Open Access angeboten werden also wirklich Softwarelösung.	
Interview 38	00:04:30		Welche Anforderungen stellen Sie dann an die geteilten Daten und Informationen?
Interview 38	00:04:35	Welche Anforderungen.? Wichtig ist dass Daten nicht zensiert werden oder dass eine Lizenz brauchen, selbst dann wenn sie Open Access sind. Un da versuchen wir natürlich möglichst Open zu sein. Wir versuchen möglichst das oberste Niveau zu erreichen CCB.	
Interview 38	00:04:55	Wir versuchen auftaten aufzusetzen die CCB sind und Daten anzubieten die CCB sind. Aber weil wir auch als Institut auftreten, die Inhalte lizenzieren dürfen können wir das auch kombinieren. Wir brauchen nicht nur CCB Daten.	
Interview 38	00:05:10	Wir können auch andere Inhalte lizenzieren und wir haben schon den Anspruch möglichst offen zu sein. Aber wir ziehen uns keine strikte Grenze sondern wir werden auch mit anderen Anbietern zusammenarbeiten.	
Interview 38	00:05:26		Und wie kommen sie momentan an geteilte Daten heran?

Interview 38	00:05:31	hauptsächlich über Zusammenarbeit mit anderen Instituten. Die die wichtigen Datenquellen die sind natürlich an größere oder an an bestimmten Instituten verfügbar, ich würde mal sagen an CBI. Und ich würde mal sagen Wir haben zwei große Kanäle. der eine große Kanal ist die BioInformatik und DENBI, das Deutsche Netzwerk für Bionformatik und Elixir, das ist das Deutsche Zentrum für Bioinformatik. Dort gibt es verschiedene Zentren die Daten anbieten genauso wie wir auch Daten anbieten, also wir bieten zum Beispiel Open Access Literatur Daten (unv.). Das ist der eine große Kanal und der zweite große Kanal ist zusammen. Die Konsortien mit der NFDI, die jetzt gerade in der Vorbereitung sind. und dort haben wir ein Konsortium aufgebaut was sich um klinische epidemiologische Daten und klinische Studien orientiert und diese Daten sind natürlich proprietär.	
Interview 38	00:06:37	Aber was wir machen können ist, oder wo wir beitragen ist dass wir. Wenn man diese Daten ihrer Datenbank hat. Dann haben die natürlich mit Beschreibungen und Beschreibungen sagt was in den Daten sozusagen drin ist, das nenne wir Metadaten.	
Interview 38	00:06:55	Und Metadaten auf die man zugreifen. Und die versuchen wir dann auch untereinander zu teilen so dass man über die Metadaten dann auf die richtigen Daten Zugriff bekommen wobei man dann natürlich eine Lizenz braucht.	
Interview 38	00:07:10	Das war jetzt eine lange Erklärungen da müssen Sie wahrscheinlich ein bisschen nachlesen was das alles ist aber wenn Sie noch Fragen haben, sagen Sie bescheid.	
Interview 38	00:07:15		Ja gerne, werde ich machen. Auf welche Daten würden Sie denn gerne zusätzlich zugreifen?
Interview 38	00:07:25	Wir sind Lebenswissenschaftlich orientiert und in den Lebenswissenschaften gibt es schon viele Daten, die aus der experimentellen Biologie stammen, das ist sehr wertvoll. und daraus können wir ableiten wie man Krankheiten versteht die Medikamente werden welche Risiken Patienten haben. Und wo wir gerne Zugreifen und offener mit umgehen würden, ist Patientendaten, was natürlich nicht so einfach ist. Da muss man schon drauf achten dass die Patientendaten geschützt bleiben aber trotzdem der Forschung zur Verfügung stehen aber sie auch zum Nutzen der Patienten genutzt werden können. Das wären wichtige Daten. Das ist eigentlich das wesentliche für uns. Also das sind Krankenhausdaten im Endeffekt.	
Interview 38	00:08:12		Um jetzt in ein Zukunftsszenario zu gehen. Wie stellen Sie sich in Zukunft den idealen Zugriff auf benötigte Daten vor.

Interview 38	00:08:22	Der ideale Zustand ist natürlich. dass man hat erstmal Daten auf einem Rechner liegen. Dann gibt es eine Beschreibung, die zu diesen Daten gehört. Die kann man dann gesondert aufrufen und nutzen. Sowohl die Daten als auch diese Metadaten befolgen Standards. Die sind dann praktisch so notiert oder erhalten dass man ohne großen Aufwand auf sie zugreifen kann. Und jetzt möchte man sie natürlich gerne analysieren. Man möchte dann gerne diese Daten auch in der Infrastruktur also auf Computern. Mit denen man dann die Daten analysieren kann. Wenn man jetzt die Analyse hat. Dann sind das ja wieder Ergebnisse, wieder Daten. Diese Daten kann man auch gleich wieder anbieten. Wenn man die Daten anbietet dann möchte ich auch gerne gleich noch eine Veröffentlichung anbieten. Und jetzt kann man schon erkennen dass aus den Daten, die vorhanden, sind leicht angreifbar sind die analysiert und dann sofort neue Ergebnisse kommen und man sofort veröffentlichen kann. wenn das alles in einer Infrastruktur offen zugänglich möglich wäre. Dann wären wir einen großen Schritt weiter.	
Interview 38	00:09:36		Welche Probleme und Schwierigkeiten treten denn momentan beim verwenden geteilter Daten auf?
Interview 38	00:09:45	Ich denke mal, also, denke ich mal. Allgemein über alle Daten rede, es gibt natürlich viele Daten, die nicht geteilt werden. Wir achen ja Forschung nd generieren Daten. und machen uns erstmal keine Gedanken dadrüber ob die auch für jemand anders nützlich sein könnten.	
Interview 38	00:10:00	Es gibt auch Beispiele wo man erkennen konnte, dass eine klinische Studie oder aus anderen Untersuchungen Daten öffentlich gemacht wurden während man die Fehler in der Studie gefunden hätte und dann genau verstanden hätte was richtig oder falsch ist. Im Grunde müssen wir erreichen wenn man mal genau hinschaut dann haben wir jetzt mal in den letzten zehn Jahren einen Wandel gemacht von.	
Interview 38	00:10:26	Lizensierter Literatur zu Open Access Literatur was allen forschenden hilft das funktioniert einigermaßen OK. Und jetzt muss man mal langsam den Wechsel schaffen wenn man Forschung betreibt.	
Interview 38	00:10:39	Dass man Kandidaten auch anbietet. Also zum Beispiels auf Rechnern oder in Infrastruktur oder in der Datenbank oder über Institute und dann diese Daten zur Nachnutzung erlauben kann. Es muss ein Köpferollen bekommen wenn wir das haben dann schaffen wir auch die Infrastruktur datu.	
Interview 38	00:11:00		Welche Daten teilen Sie denn jetzt worüber? wir schon gesagt Sie sind ja jetzt nicht so aktiv aber was machen Sie denn in der Hinsicht?
Interview 38	00:11:09	Wir haben unsere eigenen Server, über die wir Daten anbieten. Die anderen Repositorien müssen wir immer nachhacken, unseres nennt sich Publisso, aber ich meine wir sind auch ein Open Air aktiv und in der Wiki foundation sind wiraktiv Open Source ist. Wir machen allerdings keine experimentelle Forschung, da gibt es verschiedende Kanäle für die Analyse.	

Interview 38	00:11:52		Welche Beweggründe sehen Sie denn daran Daten mit anderen auszutauschen?
Interview 38	00:11:56	Der wichtigste Grund ist, wennn man gemeinsam Forschung betriebt um auch Daten herum, dass man damit sein akademisches Potential in die höhe schraubt.	
Interview 38	00:12:07	Damit auch sein akademisches Potenzial in die Höhe schraubt. Je mehr man Inhalte generiert. Die andere auch nutzen können. Und die anderen auch zitieren. Desto höher ist der verletzungsgrad und desto höher ist auch der Gewinn. Der akademische Gewinn. Das Zeigt sich schon bei Open Access in der Literatur aber das überträgt sich auf Daten eins zu eins.	
Interview 38	00:12:33		Welche Probleme und Barrieren sehen Sie denn dann beim Data Sharing. Allgemein.?
Interview 38	00:12:40	Das habe ich vorhin schon gesagt, dass Forscher selber. Zu unsicher sind oder daran zweifeln dass das Data Sharing was bringt. Aber dann ist natürlich Aufwand damit verbunden die Daten entsprechend standardisieren und erklären was sie zu bedeuten haben und wie sie genutzt werden können. Und da muss man natürlich Infrastruktur aufbauen, dass wenn man nicht entsprechend die eigene Infrastruktur hat dann kann man natürlich auch keien Daten anbieten. DAs sind so die Hürden, die ich sehen, aber das sind so die einzigen.	
Interview 38	00:13:07		Wie ist das denn mit finanziellen Aspekten. Und langfrstger Förderung von der Infrastruktur?
Interview 38	00:13:22	Der Infrastruktur.	
Interview 38	00:13:25	Ist klar dass man sich immer wünscht dass man solche Infrastrukturen öffentlich fördert. Die Gelder zur Verfügung gestellt werden. Ist auch klar dass wir dort Grenzen haben. Aber man muss natürlich auch umgekehrt sehen dass jede nationale Regierung auch haushalten muss. die kann jetzt nicht einfach hingehen und sagen wir mal einen anderen Bereich runter wie z.B. Physik Physik Forschung oder klinische Forschung machen wir dann mal größere Daten Infrastrukturen.	
Interview 38	00:13:53	Ich könnte mir vorstellen dass im Rahmen von Forschungsprojekten eine Quote ausgewiesen wird, ein bestimmter Prozentsatz in dem Projekt der für die Daten Nachnutzung gebraucht wird. Das müsste man sich überlegen. So ähnlich wie die Reisekosten in den FörderProjekten die Publikationsskosten in FörderProjekten . Genauso könnte man sagen es kosten die in jedem Projekt sind. Gleichzeitig. aber man würde dann auch gleichzeitig Erwarten dass man prüft welches Ergebnis sich aus diesen Daten ergibt dass man eine Vorstellung gewinnt. Ob das Ergebnis was abgeliefert wurde auch durch die Daten. Ob das langfristig einen impact hat.	
Interview 38	00:14:41		Jetzt sind wir ja schon beim Thema prüfen.
Interview 38	00:14:44		Inwieweit Denken Sie an rechtliche Aspekte Einfluss darauf inwieweit Daten geteilt werden.

Interview 38	00:14:52	Ich denke mal dass das auch ein interessantes Thema weil in den Köpfen natürlich immer auftaucht dass was für Grenzen wir haben. Aber. Es gibt Grenzen, die sind natürlich sehr enggesteckt. Ich habe schon personenbezogene Daten erwähnt, das ist auch wichtig.	
Interview 38	00:15:10	Da braucht man auch gewisse Regeln nach denen man das regelt. Aber ich habe irgendwie den Eindruck dass wir den ganzen Spielraum, wie wir Daten nutzen können, noch gar nicht ausgelotet haben. Es gibt sicher Daten die unkritisch sind es gibt andere Daten die sind kommerziell relevant, es gibt andere Daten die müssen geschützt werden weil sie nicht personenbezogen sind aber doch dann sensibel sind wie zum Beispiel. Dinge die kritische Infrastrukturen betreffen. Es gibt verschiedene Ebenen was man schützen muss. Wir werden natürlich bei dem offenen Bereich anfangen.	
Interview 38	00:15:47	Bei dem Bereich der unkritischer ist aber den sollte man möglichst weit ausdehnen und ob da rechtliche Grenzen also wir werden immer gegen die rechtlichen Grenzen anarbeiten. Wir werden uns genau an dem orientieren was wir anbieten können was nicht.	
Interview 38	00:16:01	Also ich werde da jetzt nicht groß drüber diskutieren, ob man da die rechtlichen Dinge wegnehmen kann, die kann man nicht wegnehmen sondern wir müssen uns daran orientieren was die Bedingungen aufkommen, also momentan ist im gespräch, dass sämtliche Informationen die auf nationaler Ebene gewonnen werden, wie zum Beispiel in Afrika und in Asien, die sollen kommerzielle Beschränkungen, dass dort, eingebracht werden sodass man die Nutzung kommerziell vergüten muss und zum Beispiel Dinge wo man zwar kann aber auf der einen Seite Schranken aufzieht und da muss man halt herausfinden wie man solche Probleme löst.	
Interview 38	00:16:42		Nun haben Sie ja eine sehr wichtige und einflussreiche Position. Inwieweit glauben Sie denn dass das Management einen Einfluss darauf hat wie Daten geteilt werden?
Interview 38	00:16:54	Wir haben einen großen Einfluss. Wir können, aber mal geteilt werden müssen. Wir müssen uns überlegen ob wir Open Access Policies betreiben und das auch formulieren. Und.	
Interview 38	00:17:11	Dann hat man auf der anderen Seite die individuelle Einstellung ob man jetzt Daten teilt oder nicht. Es sind zwei verschiedene Aspekte.	
Interview 38	00:17:21	Ich glaube am besten kann man den Erfolg von offenen Daten an Beispielen erkennen wenn Mitarbeitende Daten selber öffentlich machen wenn man sich zeigt welche Erfolge sich daraus ergibt. Das ist die beste Vorgehensweise aber als Management muss man trotzdem darlegen ob manOpen Access befürwortet oder nicht.	
Interview 38	00:17:42	Ein Institut muss eine Open Access Policy haben, muss darlegen wie man die Open Access anbieten sollte muss aber auch Richtlinien geben unter welchen Bedingungen die Daten anbieten kann und teilen kann.	
Interview 38	00:17:59		Wird denn das Teilen von Daten in der wissenschaftlichen Community anerkannt?

Interview 38	00:18:04	Ja, auf jeden Fall dadurch dass man sie zitierter macht. Das ist das höchste Gut, dadruh kriegt man Impact Faktoren.	
Interview 38	00:18:08	Das ist zwar noch nicht so dass wir das in bester Form umsetzen. aber es gibt schon genug Journals die Daten veröffentlichen und dann die auch zitierfähig machen und das Maunskript dazu das Gegenstück dazu ist.	
Interview 38	00:18:15		Nun haben wir ja schon über ganz viele Probleme und Barrieren gesprochen. Welche glauben Sie ist denn das größte Problem beim Data Sharing.?
Interview 38	00:18:36	Wir haben von mehreren Dingen geredet, Wir haben vom Indivium geredet, wir haben vom Geld geredet was Infrastrukturen darstellt. Wir haben von rechtlichen Bedinungen geredet. Das sind so die drei, die man vergleichen kann.	
Interview 38	00:18:49	Was sehe ich als die größte Hürde an? im medizinischen klinischen Bereich. Sind es die rechtlichen Bedingungen. Im sagen wir mal im experimentellen Bereich Chemie Physik und so weiter ist es eher dass die Individuen und die Infrastruktur,	
Interview 38	00:19:06	Also das Gegenteil, nicht die rechtlichen.	
Interview 38	00:19:14		Vielen Dank für die Ausführungen. Ist sehr interessant. Dann habe ich noch eine ganz andere Frage an Sie. (…) Wie schätzen Sie den Einfluss dieser Technologien (Big Data und künstliche Intelligenz) auf den Forschungsalltag ein?
Interview 38	00:19:39	Also das ist, für mich ist der Abstand zwischen dem, was wir - Big Data macht einen große Unterscheid, das ist eine ganz klare Sache. Da haben wir enorm dazu gelernt über die letzten Jahre wie wir mit großen Daten umgehen. -und das, was wir vor 10, 15 Jahre bezeichhnet ahben und was wir heute als große Daten bezeichnen, das ist schon beeindruckend. Also ein sehr großer Unterschied.	

Interview 38	00:20:00	Der große Unterschied ist dass wir große Daten sammeln und sie nutzen können. Das ist schon sehr beeindruckend. Und da braucht man bloß Das Beispiel zu nennen, dass es mittlerweile Plattformen gibt wie zum Beispiel Facebook oder Google die dann eine Milliarde oder zwei Milliarden Menschen auf der Erde sage ich mal kennen. Und dann daraus Schlussfolgerungen ziehen können kann man positiv negativ drüber sein. Aber diese Informationsmenge, das hätte man sich vor 50 Jahren nicht vorstellen können. Künstliche Intelligenz ist momentan sehr modern. Aber wird auch ein bisschen überbewertet für meine Begriffe. Im Grunde tun wir das Gleiche was wir früher gemacht haben jetzt noch besser. Und das natürlich durch viel größere Daten, was auch gut ist. nur künstliche Intelligenz hat immer sehr.,Isolierte Entscheidungen die gefällt werden. das kann die künstliche Intelligenz sehr gut, manchmal in Bereichen die für uns schwer zugänglich sind. Aber diese Entscheidungen sind sehr punktuell da kommt ein bisschen die Begrenzung rein. Wir sind noch nicht dort. Wenn man sagen würde künstliche Intelligenz ist etwas ähnliches wie der Mensch macht dass sie sich einer Umgebung anschaut die versteht lernt und dann damit umgehen kann. Da sind wir noch nicht, Das kann sie noch nicht. Sie kann komplizierte Situationen unterscheiden. Aber wenn dann eben die Situation sich verändert andere Entscheidung entsteht, dann reichen häufig doch die Daten nicht aus. Das sieht man dann beim automatischen Autofahren. Das geht gut bei Straßen die alle gleich aussehen. Und es sieht gut aus wenn die Straßen mal ein bisschen komisch aussehen.	
Interview 38	00:21:36		Ja das stimmt. Da gibt es auf jeden Fall auch Dinge die man verbessern kann.
Interview 38	00:21:43		Dann gucke ich kurz auf meinen Zettel ob ich noch Fragen offen habe einen Moment.
Interview 38	00:21:58	Die letzte Frage Mehr oder minder aus einem Fragebogen. Sie haben mal gesagt Sie sind jetzt nicht so stark aktiv beim Data Sharing. Haben Sie irgendwelche Intentionen diese Aktivitäten weiter auszubauen?	
Interview 38	00:22:18	Jap, NFDI. Da sind wir voll dabei.	
Interview 38	00:22:19		Inwie weit soll der Ausbau erfolgen?
Interview 38	00:22:21	Wir haben drei Richtungen die wir uns orientieren. Das eine ist, dass wir versuchen epidemiologische Daten und klinische Studien zusammenzuführen und dadurch. dann Die Möglichkeit schaffen dass wir, Auch über die Studie hinweg. Beurteilen können.	

Interview 38	00:22:43	Was für Gefährungen, Probleme wir haben. Das ist eine Sache. die zweite Sache ist dass wir uns auf Bakterien konzentrieren. Und zwar Bakterien die in Gemeinschaften leben. das ist ein sehr interessantes Forschungsfeld, weil man so ähnlich wie Menschen in einer Stadt sich anschauen kann warum die zusammenpassen. bei Bakterien ist es so, da geht halt viel über chemische Substanzen und /. Auf jeden Fall. Man kann es gar nicht so genau herausfiltern und das ist medizinisch relevant. Weil Bei uns auf der Haut und im Darm Kolonien leben, die man gerne verstehen will. Das ist die zweite Sache. Und die dritte Sache ist wenn man sich die Lebenswissenschaften anschaut, dann sind das viele verschiedene Bereiche. Aber die gehören zusammen. Da ist dann irgendwie Biologie in der Mitte, das sind dann Tiere und dann sind es Menschen. Und rein theoretisch können wir - aus dER Forschung wissen wir, das können wir bei Schweinen beobachten oder bei Affen oder bei Mäusen, dann hat es natürlich Relevanz für den Menschen und wir überlegen uns ob wir hier, wo wir aktiv werden wollen, ist dass wir helfen diese Daten kompatibel zu machen. Dass man Daten aus der Biodiversitätsforschung nehmen kann und dann dort Rückschlüsse ziehen ob Beispielsweise diese Bakterien zusammenleben und dann wieder auf den Menschen. Und dafür muss man halt die Standardisierung und die Metadaten zusammenbringen. Und das ist die Vorgehensweise.	
Interview 38	00:24:26		Gut weil ich für meine Statistik gern noch ein paar abschließende Punkte. Frage Was ist Ihr größter Abschluss.
Interview 38		(...)	
Interview 39	Time	Interviewee 39	Interviewer
Interview 39			Meine erste Frage an Sie, um sie auch ein bisschen besser kennen zu lernen : was ist ihre aufgabe hier an diesem Institut?
Interview 39	00:20	Also ich leite dieses Institut. Das ist ein Institut in der medizinisches fakultät, das heißt Biochemisches Instiut. Das heißt unsere Hauptaufgabe ist es, Medizinstudenten in der Vorklinik das Fach Biochemie zu unterrichten. Das muss ich organisieren. Und den Rest der Zeit habe ich sozusagen die Möglichkeit, wenn ich das will, zu forschen oder mich auf irgendwelche Sachen vorzubereiten, auf meine Lehre vorzubereiten oder über meine Forschung nachzudenken oder mich weiterzubilden.	
Interview 39	00:50		Und in welchen Data Sharing Aktivitäten sind sie involviert?
Interview 39	01:00	Das Wort Data Sharing-Aktivität sagt mir nichts.	
Interview 39	01:02		Also welche Daten tauschen sie mit anderen aus?
Interview 39		Was heißt denn eigentlich mit anderen`? Also wir sind sozusagen ja hier ein Institut, wir sind knapp hundert leute. Und wir haben natürlich bestimmte Seminarformate, bei denen wir Daten austauschen. Das wäre dann im Institut, aber schon über Arbeitsgruppen hinweg. An dem Institu gibt es mindestens drei, vielleicht vier Arbeitsgruppen und die tauschen auch schon in diesen Seminaren untereinander aus.	

Interview 39	01:34		Und werden auch Daten mit Externen ausgetauscht?
Interview 39	01:37	Ja natürlich, das wäre dann eher sozusagen mit Kollegen, mit denen ich so richtig zusammen arbeite. Und die können dann aber auch weltweit sein. Und dann je nachdem wie hoch sdas vertrauen ist, sagt man sich alles ,oder fast, oder nur die Hälfte, oder nur ganz wenig. So funktioniert, ja.	
Interview 39	02:00		Inwieweit verwenden sie denn Daten von anderen bei Ihrer Forschung?
Interview 39	02:05	Sehr häufig, es ist sozusagen natürlich in Absprache, aber wir arbeiten mit vielen Modellen, die wir entwickelt haben und andere arbeiten mit vielen Modellen, die auch wir entwickelt haben und vice versa. Und dann sozusagen das so ein Modell zu entwickeln, zum Beispiel so ein Tiermodell dauert viele Jahre. Und wenn wir mit vielen Tiermodellen arbeiten wollen, dann entwickeln wir nicht alle, sondern wir schicken dann sozusagen unsere Werkzeuge irgendwo hin und dann werden die dort verwendet. Um umgekehrt sozusagen, das tun andere auch. Das ist dann eine Internationale Zusammenarbeit	
Interview 39	02:40		Auf welche Daten würden Sie denn gerne noch zusätzlich zugreifen?
Interview 39	02:45	Das reicht mir eigentlich, ja.	
Interview 39		Das ist eine vertrauenssache in unserem Fall. Ich weis nicht wie dasb ei ihnen ist, aber in meinem Miteu kann man alles, was man tut und was noch nicht veröffentlicht ist, kann man patentieren. So ein Patent ist in der Regel / schreibt man das und dann steht das irgendwo. Aber Unter Umständen kann das unglaublich viel Geld wert sein. Und eine information, fast jede Information, die mir jemand erzählt, oder die ich jemandem erzählen, könnte der andere in der Tat unter seinem Namen patentieren- das ist eine Vertrauenssache. Ich tausche mich nur mit jemandem aus, von dem ich weiß,. dass er mich hintergeht, oder von dem ichh offe, oder von dem ich glaube, dass er mich nicht hintergeht. und das ist eignetlich sozusagen der Punkt. Also ich tausche mich gerne mit vielen Leuten aus, aber ich muss zu denen auch vertrauen haben.	
Interview 39	03:43		Und wie bauen Sie dann das Vertrauen auf?

Interview 39	03:45	Also in meinem Fall, also das ist so eher meine persönliche Sache, ich muss die treffen. Also ich kann das nicht per email machen, schon gar nicht / oder ich kann das auch nicht per skype machen, obwohl skype ist schon besser als Email - obwohl eigentlich muss ich die treffen. und muss die mal, ja mit denen drei Tage auf einer Tagung gewesen sein, da gehört auch zu dass man Abends mal ein Bier trinken geht. Also ich muss die einfach sehen. Und möglichst muss ich auch mit denen schon mal so ein bisschen zusammen gearbeitet haben und erste Erfahrungen gemacht haben wie es ist, wenn man mit denen gemeinsam mit denen eine Publikation schreibt, ob es da Probleme gibt, ob die sehr empfindlich sind, ob die umbedingt alles für sich reklamieren oder eben so - das muss man kennen lernen. Und dann hat man so, oder ich habe dann so, so gewisse (-) auch Resontiments, manche verhaltensweisen mag ich nicht so und das würde mich dann davor davon abhalten mit diesen Leuten mehr zu machen als vielleicht mit anderen, wo ich sagen würde: ja doch, das ist so, dass es ungefähr so, wie ich das auch machen würde.	
Interview 39	04:45		Welche Anforderungen stellen sie denn an die geteilten Daten?
Interview 39	04:50	Die erste Anforderung ist, dass sie transparent sind und stimmen müssen. Auch hinterfragbar sind. Also wenn mir irgendjemand irgendwelche Daten schickt und ich habe damit Probleme, dann muss ich Fragen können, also das verstehe ich nicht. Sozusagen wie ist denn dieser Datenpunkt entstanden. Und nur wenn da sozusagen wirklich transparenz herrscht, dann habe ich auch vertrauen.	
Interview 39	05:15		Wie kommen Sie denn dann momentan an die Daten ran?
Interview 39	05:20	Ich meine, unsere Daten sind ja veröffentlicht in der Regel, aber spannend ist eigentlich, dass vor der Veröffentlichung, also weil, wenn etwas veröffentlicht ist, dann weiß es ja jeder. Wenn ich aber jetzt zu jedem ein gutes Verhältnis habe, dann weiß ich schon zwei Jahre bevor die Daten veröffentlicht sind, kenne ich die schon. Und kann mit den auch schon arbeiten, kann, weiß dann sozusagen ja darauf kann ich aufbauen und kann vielleicht wieder andere Sachen machen, die dann sozusagen darauf schon wieder aufbauen,. Und da habe ich dann zwei Jahre vorsprung vor anderen Leuten, die vielleicht so etwas ähnliches wollen. Das ist ganz wichtig in unserem Miteu. Bei BWL vielleicht nicht so, aber (-) also bei uns ist das sehr sehr wichtig.	
Interview 39	06:03		Um jetzt mal un so ein Zukunftsszenario zu gehen: wie stellen sie sich denn Zugriff auf die benötigten in der Zukunft vor - also den optimalen Zugriff?

Interview 39	06:15	Ich finde, der ist optimal. Also für mich ist nicht wichtig, ist nicht wichtig wie die Daten jetzt an mich rankommen, ob die jetzt aus die Cloud oder ob da ein versiegelter Brief kommt, das ist vollkommen egal. Damit kann ich immer was anfangen. Das wichtigste ist, also für mich ist das wirklich das vertrauen zu meinen, zu den Kollegen, mit denne ich solche Daten auch vertrauliche Daten, austausche und da muss vertrauen da sein. Wenn dieses Vertrauen da ist, dann ist eigentlich der Weg, wie der austausch ist, vollkommen egal.	
Interview 39	06:46		Treten denn sonstige Probleme oder Herausforderungen beim Datenaustausch auf?
Interview 39	06:53	Also neben dem, was ich jetzt gerade erklärt habe, eigentlich nicht.	
Interview 39	07:00		Welche Beweggründe haben Sie denn dann, Daten mit anderen zu teilen?
Interview 39	07:03	Weil mich das Thema interessiert und weil, weil ich will, dass das Thema weiter kommt. Und weil sozusagen die Hilfe von anderen auch sozusagen mir selber hilft also. Unsere Ideen entstehen meistens gemeinsam, also die hat nicht einer sozusagen an seinem stilen Schreibtisch. Sondern eigentlich in dem Moment, wo wir uns austauschen, agerade so vielleicht drei, vier leute an einem Tisch sitzen und über ein Thema reden, da kommen wir weiter. Alleine kommt man eigentlich nicht so gut weiter.	
Interview 39	07:40		Sie haben ja jetzt gesagt, dass sie die anderen tauschen, wenn sie ein Vertrauenverhältnis haben und natürlich auch die Daten mit anderen teilen, wenn sie die veröffentlichen.
Interview 39	07:53	Ja, wobei, das müssen sie sehen. Veröffentlichen ist was anderes, wenn ich Daten veröffentliche, dann kann das jeder lesen, jeder chinese, jeder sonst wo. Das ist eigentlich wichtig was man macht mit den Daten vor der Veröffentlichung, was ungefähr so manchmal dauert das so ein bis zwei Jahre bis man sie veröffentlichen kann. Und die Leute, denen man in dieser Zeit schon die Daten gibt oder austauscht, die sind sehr priviliegiert, weil die schon lange vor, bevor eben der Rest der Welt sie bekommt, diese Daten schon kennt. Und das ist auch für das Geben und nehmen. ALso sozusagen wenn ich jetzt einem Kollegen vertrauliche geben von mir gebe, dann ist gar nicht unwahrscheinlich, dass er das dann auch macht oder sie das auch macht. Und das ist halt so ein, das ist wie ein Freundschaftsverhältnis. Sie würden ja auch nicht mit jedem Wildfremden ich weiß nicht was machen. Pullover oder was immer sie ihnen so einfällt. Aber in dem moment in dem sie jetzt wissen, der oder die mit der bin ich shcon seit Jahren sehr sehr gut befreundet und dann tut man da andere Sachen zusammen. Das ist ja so, ist das bei uns Wissenschaftler auch.	
Interview 39	09:06		Stellen Sie dann die Daten nachdem sie die verwendet haben und eventuell publiziert haben in öffentliche Repositorien?

Interview 39	09:15	Wenn sie publiziert sind muss ich das ja nicht mehr tun. Was wir wohl machen, ist also wir schreiben manchmal also sozusagen die allerletzten Details, die würden nochmal sehr viel Platz wegnehmen und dann schrieben wir manchmal, das kann sozusagen auf Anfrage beim Autor erfragt werden. Aber da würde ich dann jedem also wirklich egal wer die sch / wer mir dann schreibt, der schreibt mir, ich würde gerne das und das wissen, dann kriegt er eine Email und dann kriegt er diese daten. Also so da gibt es eigentlich keine Restriktionen. Also so .	
Interview 39	09:50	Bei uns, also wie gesagt ist wichtig vor der Veröffentolichung, danach, also wenn die Daten mal raus sind, dann sind sie raus. Dann, ja.	
Interview 39	10:03		Dann hat man ja auch keine Verfügungsgewalt mehr darüber.
Interview 39	10:05	Ja und Sie können sie ja auch nicht mehr patentieren. In dem moment wo das veröffetnlicht ist, ist ein Patent vorbei. Also sie müssen wenn sie patentieren wollen, und wenn sie daten irgendwie verwenden wollen um zum Beispiel eine Firma zu gründen oder von eine Firma geld zu bekommen damit die das entwickeln, dann muss ein Patent da sein, sonst geht das nicht. Also Paten wird Ihnen nie eine Firma auch nur einen Euro auf den Tisch legen. Weil es ja nicht geschützt ist, dann kann ja jeder / dann stecken die Millionen rein in die Entwicklung und dann wenn es funkltioniert hat, sagt eine andere Firma prima, das nehme ich mir jetzt und mache damit jetzt auch ein Produkt. Das tut eine Firma nicht, das muss egeschützt sein für Jahre und dann lohnt sich das vielleicht. Und das ist alles vor der Veröffentlichung.	
Interview 39	10:50		Sie haben ja gesagt, sie teilen die Daten cloud oder email oder was auch immer, es sei ihnen ja relativ egal. Gibt es da denn auch technische Probleme?
Interview 39	11:00	Ne, wirklich keine.	
Interview 39	11:03		Ok, gut zu hören. Beeinflussen irgendwie /
Interview 39	11:08	Außer das Internet bricht zusammen, was ja manchmal auch ist, aber das ist hja ganz selten. Und ja, es gibt keine Probleme.	
Interview 39	11:17		Wird denn dann das Daten austauschen in dem Wissenschaftsfeld anerkannt?
Interview 39	11:25	Was heißt anerkannt?	
Interview 39	11:28		Man bekommt ja beispielsweise Anerkennung in dem man ein Journal Artikel veröffentlicht und so weiter. Wie ist das dann beim Daten austauschen?

Interview 39	11:37	Das sind doch sehr, sehr kleine Communitites sage ich mal. Das also ich tausche vielleicht so richtig frei aus Daten mit (-) also maximal zehn Arbeitsgruppen - maximal, eher vielleicht ein paar weniger. Und, glaube das ist auch so ungefähr das , was jeder macht. Das heißt nicht, dass ich nicht Material rausgebe, also ich verschicke sehr viel Material. Aber das ist anders sozusagen, da kriege ich halt eine offizielle Anfrage, dann bekommt er das und das ist unter bestimmten Spielregeln, aber so richtig frei austauschen tut man nur mit relativ wenigen. So wie man eben auch wenige oder nur manche so richtig enge Freunde hat. Das ist so, kann man das glaube ich ganz gut vergleichen. Also einem Freund vertrauen sie und einem Kollgeen, mit dem ich mich frei eben austausche, dem vertrauche ja auch.	
Interview 39	12:30		Also ist schon sehr vertrauensbasierter Austausch.
Interview 39	12:35	Ja, ja.	
Interview 39			Durch Patent und durch Kennenlernen und so
Interview 39	12:39	Vor dem Patent ja, wenn es mal patentier t ist, ist alles gut. Dann können sie machen was sie wollen. Aber bis dahin ist es ja, sie haben eine Idee. Stellen sie sich, vor sie haben eine Idee und die ist eine Milliarde euro wert. In dem moment, in dem sie die idee mir schon sagen, da könnte ich dem moment in dem sie draussen sind, ich schreibe jetzt heute ein patent und ich sage, habe ich erfunden - können nicht sagengen machen. Dann haben sie eine Milliarde euro verlorn nur weil sie geplaudert haben und zwar dem falschen gegenüber - so ist das. und das heißt, wenn sie jetzt so eine idee hätte, die würden sie wirklich nur dem oder die erzählen bei der sie sagen, der zieht mich nicht über sohr sondern dem kann ich vertrauen.	
Interview 39	13:25		Haben sie sonst noch aspekte zum Data Sharing oder Daten austausch, die sie so einfällt?
Interview 39	13:30	Ne.	
Interview 39	13:33		Gut.
