# Supplementary material for: Data platforms for open life sciences–A systematic analysis of management instruments
Source: PLoS One. 2022 Oct 25;17(10):e0276204. doi: 10.1371/journal.pone.0276204 (PMC9595524; doi:10.1371/journal.pone.0276204)
Supplement: S4 File — (DOCX) [file pone.0276204.s008.docx]

# S8. File. Detailed information on data platform types

While all platforms within the three platform types are aiming for providing common structures they substantially differ in their governance and management structure.

**Enabler platforms** offer both standardised and interlinked data for the scientific community as well as tools for direct analysis of the data on the platform. In addition to the technological functions to store, publish, and access data, the Enabler platforms also provide the possibility to discover data with tools directly on the platform. The special value of tools on data sharing platforms is highlighted by Richter and Slowinski (2019): “competitive advantage also stems from the extent of the integration of additional functions and services which are directly related to the semantic dimension of data that is traded on the platforms”. The statement also illustrates that tools are only possible if data is available in a standardised form. By offering tools, Enabler platforms depend on corresponding data quality. To achieve this, Enabler platforms offer curation of data and can also extract additional data from the literature. The latter makes Enabler platforms still very personnel intensive, because automatic data mining is not yet fully possible. On the one hand, this may be due to the lack of tools and, on the other hand, there are limited access possibilities. While the licence agreements of Enabler platforms vary, all of them are characterised by extensive data upload requirements. These strict requirements not only determine the quality of data and possibilities of tools on the platform, but also ensure that the platform incorporates scarce resources that are also needed by other users (Boudreau and Hagiu, 2009). Enabler platforms mainly encourage the use of the platforms through external requirements, the ability to store data in a citable form, websites with information for the entire community and the additional benefit of the tools. The community of the Enabler platform consists mainly of researchers working in universities, research institutes and also companies that access both the data set and the tools. Acting as intermediary, Enabler platforms offer 2 potential benefits: creating the centralised place for standardised data and lowering various transaction costs. Furthermore, when writing blog articles or spreading news and events, Enabler platforms act as a social space for the scientific community and can offer the opportunity for communication between researchers. When Enabler platforms cover a wide range of tools and want to transit the platform category, they can expand on the scientific domains which are covered. Enabler platforms needs to keep clear positioning within the covered scientific domain and not to try to become a one fits all solution. Considering the large number of domain-specific domains, umbrella solutions are also possible, such as the European Open Science Cloud (Ayris et al., 2016) or the National Research Data Infrastructure (Nationale Datenforschungsinfrastruktur (NFDI)) (Deutsche Forschungsgesellschaft, 2020).

**Backbone platforms** provide domain-specific communities with a central location for domain specific data. This includes both the standardisation of meta data and data as well as the interlinking of data from other platforms or resources. The platform governance of Backbone platforms mainly comprises CC BY and CC BY-NC licences. It follows that the data suppliers and at best the data platform are cited by researchers when reusing them. Since the Backbone platforms have particularly low FTE values and thus have the lowest average resources compared to the other platforms, they are dependent on the platforms being cited in the literature. Although there is no specific data platform citation index (e.g Peters et al., 2017), citations of used data promote the work of the platform in the scientific community. The standard practice to incentivise platform use of Backbone platforms are platform outreaches in the usual scientific manner. The domain-specific scientific community of Backbone platforms comprises researchers, working groups, museums, federal institutions covering certain scientific niches, whereby specific needs of the community can be addressed. To facilitate data exchange, Backbone platforms must take decisions regarding the technological framework and thus the degree of openness (Eisenmann et al., 2008). Parker et al. (2016) state that “platforms that operate in similar areas may choose to differentiate themselves by adapting different levels and kinds of openness”. Backbone platform facilitates, if not already done, the standardisation of domain specific data, which is linked to the perceived quality of the data. This quality can be ensured by manual curation, automatic checks, and outsourcing. However, manual curation does not scale and becomes time consuming with increasing data supplier. When transitioning to another platform category, Backbone platforms have two development possibilities: covering more domains or aiming for higher ETI. This strategic decision depends on the goals of the platform with the scientific community behind and the extent of available resources.

**Generalist platforms** can provide organisations a solution that prevents the multi-homing of different platforms while enabling cross-domain data sharing with low ETI. Regarding the platform governance of the Generalist platforms, it is noticeable that either the users themselves can determine the CC-licence or the entire platform is subject to the CC0 licence. Generalist platforms incentivising platform usage through external requirements, platform outreaches, emphasising the possibility to make data citable and disclose information on the platform. We show that generalist platforms follow a strong platform disclosure strategy by publishing the name of the data provider, posting it on the main page, and showing statistics for individual datasets. The effects of data sets statistic have been investigated by He and Han (2017) on Dryad (also in this study), show that “high positive correlations between usage counts of data and citation counts of associated articles” in WoS exists. In comparison, Thelwall and Kousha (2017) show on the same platform “low correlations [...] between data downloads and article citation counts for articles” in 2 biology journals. The user group of the Generalist platforms includes users from organisations covering various scientific disciplines such as universities, research institutes and journals, if these organisations have a need for data platforms that cover cross-domain data. The technologies of the Generalist platforms are designed for metadata and data of many or even all domains, whereby the platform can be linked to other organisations. Thus, the architecture must be designed to link the “scattered components of the scholarly infrastructure” like researchers, libraries and journals which makes APIs the key gateway technology for Generalist platforms (Plantin et al., 2018). As the platform does not offer any facilitating analysis tools for the user, mainly easy usability and accessibility of the platform is considered beneficial for the users (Cennamo, 2019). Generalist platforms are characterised by cross-domain content and low ETI, which results in challenges with regard to the data quality offered. Richter and Slowinski (2019) in the context of B2B data sharing platforms state that “a major challenge for matching, however, is the obscure quality of the traded goods, i.e. data itself”. This emphasises that we are in line with existing literature regarding the importance of data quality. It is particularly noticeable that platforms with no domain restriction enforce low metadata standards and no data standards. A data platform does not necessarily have to offer high-quality data if this is not the decisive criterion for the user group. For the transition between platform categories, Generalist platforms have got the possibility to focus on specific scientific communities. Within specific focused domains, certain use cases can be addressed.
